# The genus *Cephaloleia* Chevrolat, 1836 (Coleoptera, Chrysomelidae, Cassidinae)

**DOI:** 10.3897/zookeys.436.5766

**Published:** 2014-08-22

**Authors:** Charles L. Staines, Carlos García-Robledo

**Affiliations:** 1 Department of Entomology, MRC 187, National Museum of Natural History, Smithsonian Institution, Washington, DC 20013-7012, U.S.A.; 2 Department of Botany, MRC-166, National Museum of Natural History, Smithsonian Institution, Washington, DC 20013-7012, USA; 3 Primary affiliation: Departamento de Interacciones Multitróficas, Instituto de Ecología, A.C., Xalapa, Veracruz 91070, México

**Keywords:** *Cephaloleia*, key to species, new species, biology, Neotropics

## Abstract

The species of the Neotropical genus *Cephaloleia* Chevrolat, 1836 are revised. We present a key to the known larvae of *Cephaloleia* (8 species), a key to the 95 species known to occur in Mexico, Central America and the West Indies, and a key to the 138 species known to occur in South America. All identification keys were translated to Spanish. Descriptions for the 214 known species of *Cephaloleia* as well as illustrations for 212 species are presented. The following species are removed from *Cephaloleia*: *C. bipartita* Pic, 1926c is transferred to *Hybosispa* Weise, 1910; *C. minasensis* Pic, 1931 and *C. viridis* Pic, 1931 are transferred to *Stenispa* Baly, 1858. The following species are described as new: *C. abdita* **sp. n.** from Brazil; *C. amba* **sp. n.** from Colombia, Ecuador, and Peru; *C. angustacollis* **sp. n.** from Ecuador; *C. brevis* **sp. n.** from French Guiana; *C. calathae* **sp. n.** from Costa Rica; *C. chica* **sp. n.** from Peru; *C. conforma* **sp. n.** from Costa Rica; *C. crenulata* **sp. n.** from Ecuador; *C. gemma* **sp. n.** from Bolivia and Brazil; *C. horvitzae* **sp. n.** from French Guiana; *C. interrupta* **sp. n.** from Costa Rica; *C. kressi* **sp. n.** from Costa Rica; *C. lenticula* **sp. n.** from Ecuador, French Guiana, Peru, and Suriname; *C. nana* **sp. n.** from Ecuador; *C. ochra* **sp. n.** from Ecuador; *C. stainesi* **sp. n.** from Costa Rica; and *C. susanae* **sp. n.** from Brazil and Ecuador. *Cephaloleia simoni* Pic, 1934 is treated as *Incertae sedis*. The larvae of *C. erichsonii* Baly, 1858 and *C. puncticollis* Baly, 1885 are described and illustrated.

## Introduction

The Neotropical genus *Cephaloleia* was erected by Chevrolat (1836) for the species *Hispa metallica* Fabricius, 1801 and *Hispa nigricornis* Fabricius, 1792. The type species is *Cephaloleia nigricornis* (Fabricius) designated by Staines (1991(1992)). *Cephaloleia* is distributed from the south of Mexico to Argentina.

The entire genus has never been revised and identifications to species are challenging. There have been four published keys to *Cephaloleia*- Uhmann (1930a) for 22 species from Costa Rica, Uhmann (1936a) for 31 species, Staines (1996) for 88 species from Central America and the West Indies, and Staines (2009b) for five species known from the Caribbean. In this revision we include 214 species.

In addition to a general overview of the literature on the taxonomy, ecology, and evolution of *Cephaloleia*, we present species descriptions for all species and images for all but two species (i.e. *Cephaloleia vittata* Staines, 1996 and *Cephaloleia amblys* Staines, 1996 whose type specimens were not available for this revision). We have been unable to locate the type of *Cephaloleia simoni* Pic, 1934, any specimen identified as *Cephaloleia simoni*, or any specimen which can be assigned to this species based on the short original description. We are treating this species as *incertae sedis*. We also included three identification keys: first a key to the known larvae of *Cephaloleia*. Then a key to the *Cephaloleia* of Mexico, Central America and the West Indies and finally a key to the *Cephaloleia* of South America. All keys were translated to Spanish.

The taxonomy of *Cephaloleia* species in Central America is very stable as a result of decades of research. Having a key only for the Mexican and Central American species will facilitate species identification for researchers working on this region. The identification of South American species is more challenging because of the high diversity of *Cephaloleia* in this continent. Although our key to the South American *Cephaloleia* includes all known species, researchers must remember that there are still many species to be described in this region. This last key is therefore a tool for both the identification and discovery of species in South America.

## Materials and methods

**Adult descriptions.** For this study, measurements were taken with an ocular micrometer. Pronotal length and width were taken along the midlines. Elytral width was measured at the humerus. Elytral length was measured from the base to the apex. Total length was measured from the base of the antennae to the apex of the elytra. In recording label data from type specimens, a slash (/) divides data on different labels; brackets ([]) contain explanatory information. Data from other specimens are reported nearly as they appear on labels, but with some standardization in the format of dates, punctation, or sequence of information. In specimens examined a question mark (?) indicates unknown province or state.

### Material was studied from the following collections

AJGC A. J. Gilbert, Fresno, CA, USA

AMNH American Museum of Natural History, New York, NY, USA

ANSP Academy of Natural Sciences, Philadelphia, PA, USA

BMNH The Natural History Museum, London, United Kingdom

BYUC Brigham Young University, Provo, UT, USA

CASC California Academy of Sciences, San Francisco, CA, USA

CDFA California Department of Food and Agriculture, Sacremento, CA, USA

CMNC Canadian Museum of Nature, Ottawa, Ontario, Canada

CNC Canadian National Collection, Ottawa, Ontario, Canada

DEIC Deutsches Entomologisches Institut, Müncheburg, Germany

DWC D. Windsor, Cuidad de Panama, Panama

EGRC E. G. Riley, College Station, TX, USA

EMEC Essig Museum of Entomology, Berkeley, CA, USA

FMNH Field Museum of Natural History, Chicago, IL, USA

FSCA Florida State Collection of Arthropods, Gainesville, FL, USA

INBIO Instituto Nacional de Biodiversidad, Santo Domingo de Jeredia, Costa Rica

INHS Illinois Natural History Survey, Champaign, IL, USA

ISNB Institut Royal des Science Naturelle de Belgique, Brussels, Belgium

LSC L. Sekerka, Liberec, Czech Republic

MACN Museo Argentina de Ciencias Naturales “Bernardino Rivadavia”, Buenos Aires, Argentina

MNHN Museum National d'Histoire Naturelle, Paris, France

MUCR University of Costa Rica, San Jose, Costa Rica

NMW Naturhistorisches Museum in Wien, Vienna, Austria

SEMC University of Kansas, Snow Entomological Museum, Lawrence, KS, USA

STMD Museum für Tierkunde, Dresden, Germany

TAMU Texas A and M University, College Station, TX USA

UMMZ University of Michigan, Ann Arbor, MI, USA

USNM National Museum of Natural History, Smithsonian Instution, Washington, DC, USA

ZMHB Museum für Naturkunde de Humboldt-Universität, Berlin, Germany

**Larva descriptions.** Measurements were taken with an ocular micrometer or from scanning electron microscope images. Total larval length was measured from the anterior to the posterior margins. Total width was measured at the widest point.

**Diet records.** We performed a comprehensive summary of diet records in published ecological studies. Although the diets of some populations of *Cephaloleia* are thoroughly studied, our knowledge on the full diet breadth of *Cephaloleia* species at broad geographic ranges is still fragmentary. We recommend to researchers interested in understanding the diet breadth of *Cephaloleia* species at a broad geographic scale, to be cautious when combining literature records from different populations. One potential source of error when combining records from different studies is that several species of *Cephaloleia* display similar morphology, leading to potential misidentifications. Because we did not have access to all specimens used in ecological studies, we could not confirm that such identifications are correct.

Another potential issue in the study of *Cephaloleia* diets using published records is that we don't understand to which extent putative *Cephaloleia* species might include several cryptic species. It is possible that species assumed to locally feed on a broad range of hosts might be a complex of sympatric cryptic species. It is also possible that populations with different diets are allopatric cryptic species. We suggest to researchers interested in studying *Cephaloleia* host associations to review the original literature and if possible the *Cephaloleia* and plant specimens used in each study. An ideal approach to address these issues is to combine morphological, ecological and molecular information to delimit *Cephaloleia* species (García–Robledo et al. 2013a).

## Systematic account

### Cephaloleia

Taxon classificationAnimaliaColeopteraChrysomelidae

Chevrolat, 1836

Cephaloleia Chevrolat 1836: 30. Type species: *Hispa nigricornis* Fabricius, designated by Staines 1991(1992): 247. Chevrolat 1843: 350 (noted); Blanchard 1845: 182 (redescription); Orbigny 1845: 60 (noted); Erichson 1847: 151 (noted); Guérin–Méneville 1855: 601 (faunal list); Baly 1858: 39 (redescription), 1869: 367 (noted), 1875: 74 (noted), 1885: 8 (distribution); Chenu and Desmarest 1870: 341 (noted); Chapuis 1875: 277 (redescription); Chenu 1884: 341 (noted); Sharp and Muir 1912: 567 (male genitalia); Maulik 1916: 568 (museum list), 1932: 294 (larva), 1933: 935 (host plants); Uhmann 1930a: 232 (Costa Rica species), 1936a: 109 (noted), 1948a: 217 (noted), 1957a: 14 (catalog), 1964a: 402 (catalog); Lepesme 1947: 529 (host plants); Guérin 1953: 97 (faunal list); Buck 1958: 146 (museum list); Beutelspacher and Batze 1975: 159 (host plants); Wilcox 1983: 136 (catalog); Gilbert and Smiley 1978: 90 (noted); Seeno and Wilcox 1982: 159 (genera); Machado–Allison et al. 1983: 248 (noted); Jolivet 1988: 14 (host plants), 1989: 303 (host plants); Strauss 1988: 95 (noted); Naeem 1990: 31 (ecology); Staines 1991(1992): 247 (type species), 1996: 4 (Central America species), 1996(1997): 13 (Nicaragua species), 1997: 413 (Uhmann species list), 1999: 240 (mimicry), 2002: 731 (key to genera), 2004: 311 (host plants), 2009a: 21 (redescription); Mariau 1994: 254 (noted); Jolivet and Hawkeswood 1995: 143 (host plants); Mexzón 1997: 28 (host plant); Staines and Staines 1999: 523 (Baly species list); Jolivet and Verma 2002: 61 (noted); Erwin and Medina 2003: 13 (predator); Arroyo et al. 2004: 203 (host plants); Farrell and Sequeira 2004: 175 (evolution); McKenna and Farrell 2005: 118 (phylogeny), 2006: 10949 (phylogeny); Strong and Sanderson 2006: 10827 (phylogeny); Williams 2006: 201 (noted); Chaboo 2007: 44 (noted); Frank and Barreta 2010: 8 (predator); García–Robledo et al. 2010: 50 (noted); Lawrence et al. 2011: 13 (phylogeny); Sekerka et al. 2013: 303 (noted); Schmitt and Frank 2013: 57 (biology).
Cephalolia Blanchard 1845: 162 (misspelling). Gemminger and Harold 1876: 3601 (catalog); Donckier 1899: 547 (catalog); Weise 1910: 82 (redescription), 1911a: 7 (catalog), 1911b: 9 (catalog); Bruch 1915: 375 (faunal list); Handlirsch 1925: 666 (classification); Uhmann 1936b: 481 (key), 1942: 94 (morphology); Bryant 1942: 205 (faunal list); Monrós and Viana 1947: 162 (Argentina species); Guérin 1953: 97 (faunal list).
Uhmannispa Monrós and Viana 1947: 172. Type species: *Uhmannispa maculata*Monrós and Viana 1947, by monotypy. Uhmann 1957a: 14 (synonymy); Staines 1995: 863 (Monrós species list).

#### Description.

Body elongate, rather subparallel (rarely oval), flat or moderately convex. Head: small; eyes oval, convex, finely faceted, slightly prominent (Figs 19–20); labrum rather large (Fig. 19), anterior margin rounded; maxillary palps with palpomere 1 short, 2 oblong conic, 3 shorter than 1 or 2, 4 subequal in length to 2, truncate at apex (Fig. 19). Antenna: filiform, slightly thickened at apex. Pronotum: quadrangular, square or transverse; frequently widest just behind apical angle; usually margined laterally, sometimes canaliculate; basal margin bisinuate or occasionally biangulate. Scutellum: short; pentagonal or triangular (Fig. 21). Elytron: variable in form and color; with 10 puncture rows plus a short scutellar row; very narrowly margined (Fig. 22); one segment of abdomen exposed. Venter: prosternum strongly contracted between coxae, truncate at base; mesosternum short, transverse; metasternum larger; suture between abdominal sterna 1 and 2 often obsolete (at least in middle). Leg: short; femur dilated in middle; tibia short, dilated toward apex, obliquely truncate at apex; tarsi wide, short; claws divaricate (Figs 25–26).

#### Larval morphology.

In general, larvae of *Cephaloleia* Chevrolat are rounded oval, longer than wide, with even, regular margins formed by wide expansion of all segments from pronotum to caudal abdominal segment forming a scale-like shield (Figs 27–54); head and legs concealed by broadly flattened margins (Figs 27–54); margins can display setae (Figs 14–18); expansions extending far forward in front of the head for a distance much greater than the width or length of the head (Figs 27–54), beyond the thorax at the sides to a width greater than ½ the width of the body proper and beyond the abdomen at the sides to a width wider on each side than the width of abdomen proper, width at caudal end nearly as great as at anterior end; expansions narrowly laminate; segments more or less distinct with spiracles (Fig. 13), sides plicate; elevated along central longitudinal medial line which is wider after the middle to the prothorax and narrows on tergites 7–9. Divisions between the head and the prosternum and abdominal tergites 7–9 are not clearly defined. Dorsal surface convex. Head retracted (Figs 11, 15); antenna with three antennomeres (Fig. 17). Legs consist of two distinct segments plus base; ending with a single strong recurved claw (Figs 12, 16).

#### Taxonomic position.

Historically *Cephaloleia* has been placed in the tribe Cephaloleiini Chapuis, 1875 (Staines 2002). The tribe Cephaloleiini has been synonymized with the tribe Imatidiini Chapuis, 1875 (Monrós and Viana 1947, Borowiec 1995, Staines 2002). However, Lawrence et al. (2011) demonstrated that the true author of Imatidiini is Hope (1840). This makes Imatidiini a senior subjective synonym of Cephaloleiini (ICZN 1999, Art. 23.1). The tribe Imatidiini contains 17 genera (Staines 2002). *Cephaloleia* can be distinguished from the other genera by the following combination of characters: antennae with 11 antennomeres; mouth not projecting forward; elytra subparallel; body not cylindrical; apical margin of pronotum truncate or weakly rounded in middle; base of elytra without carina; last three abdominal sterna not hirsute; and pygidium generally exposed.

#### Species excluded from *Cephaloleia*.

Three species currently in *Cephaloleia* need to be assigned to other genera. *Cephaloleia bipartita* Pic, 1926c belongs to *Hybosispa* Weise, 1910 due to the pronotum lacking a seta in any angle, the antennae being inserted into pits and the deep excavation of the frons. *Cephaloleia minasensis* Pic, 1931d and *Cephaloleia viridis* Pic, 1931d belong to *Stenispa* Baly, 1858 due to the antennae being inserted into shallow pits which are divided by a longitudinal keel, the shape of the basal two antennomeres, and the cylindrical body shape. The species *Cephaloleia lalli* (cited in McKenna and Farrell 2006) is not a valid name (ICZN Art. 15). Requests for this specimen or additional information were not responded to.

#### Remarks.

Most *Cephaloleia* species are generally similar in appearance. Some species are easily recognized by the body shape or color pattern. Other species can only be distinguished by the sculpture of the head. Important sculpturing is the degree and strength of punctation on the vertex (Fig. 20) and the presence, absence or shape of sulci or carinae (Fig. 20). The sulci or carinae sometimes continue between the antennal bases and onto the frons. Characters on the antennae are also important. The relative lengths of the first three antennomeres and the presence or absence of triangular projections on antennomeres two to four distinguish a number of species. Antennal projections are not used in the key for some species since the presence or absence of projections is a sexual character in these species. If the pronotal margin is canaliculate (channeled or grooved) or not is extremely useful with some species. Another useful character is whether the elytra have a declivity from behind the humerus at puncture row 7. Also on the elytra the arrangement of the apical punctures is useful in species determinations. Male and female genitalia were examined and found not useful for species determinations but the shape of the last sternite is useful for gender identification (Figs 23–24).

There are three groups of species which differ from the general pattern of the genus but do not clearly belong to other genera or justify erecting a new genus so are retained in *Cephaloleia*. The *barroi*-group (*Cephaloleia barroi* Uhmann, 1959c and *Cephaloleia sandersoni* Staines, 1996) have a convex, rounded body similar to the genera *Demotispa* Baly, 1858 and *Stilnapsis* Weise, 1905b. The *gracilis*-group (*Cephaloleia gracilis* Baly, 1878, *Cephaloleia formosus* Staines, 1996, and *Cephaloleia vagelineata* Pic, 1926c) are much more flattened than other *Cephaloleia* and have the elytral apex truncate. The *humeralis*-group (*Cephaloleia humeralis* Weise, 1910, *Cephaloleia obsoleta* Weise, 1910, and *Cephaloleia uhmanni* Staines, 1996) resembles members of *Stenispa* but differ in several characters.

Species hypotheses included in this revision are based on similarities with morphological characters of type specimens. However, molecular analyses suggest that some *Cephaloleia* species are not monophyletic but a complex of cryptic species (McKenna and Farrell 2005). Therefore, future studies will need to combine traditional taxonomy with ecological and molecular data to elucidate species boundaries.

**Figures 1–10.**
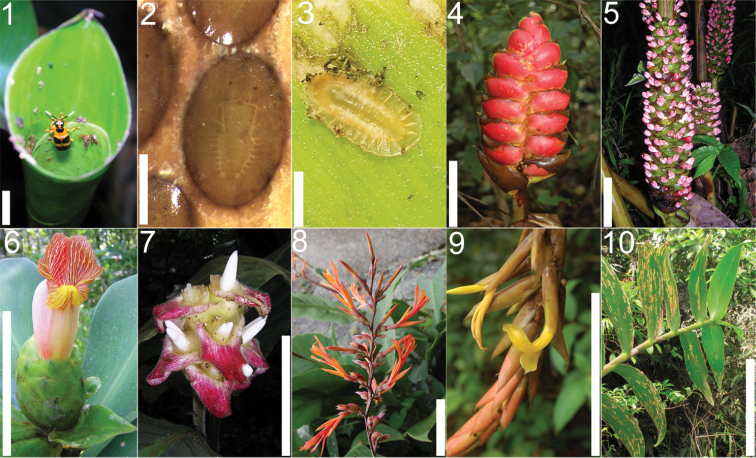
*Cephaloleia* beetles and their host plants **1** *Cephaloleia alternans* in a *Calathea* (Marantaceae) rolled leaf. Madre Selva Station, Dto Loreto, Peruvian Amazon Scale bar equals 1 cm **2** *Cephaloleia placida* eggs attached to a *Renealmia alpinia* (Zingiberaceae) leaf. La Selva Biological Station, Costa Rica. Scale bar equals 1 mm **3** *Cephaloleia dilaticollis* first instar larva feeding on *Renealmia alpinia* (Zingiberaceae). La Selva Biological Station, Costa Rica. Scale bar equals 3 mm **4–10** Examples of *Cephaloleia* host plant families, Scale bars equal 10 cm: **4** Heliconiaceae (*Heliconia imbricata*). La Selva Biological Station, Costa Rica **5** Zingiberaceae (*Renealmia costaricensis*). La Selva Biological Station, Costa Rica **6** Costaceae (*Costus malortieanus*) La Selva Biological Station, Costa Rica **7** Marantaceae (*Calathea leucostachys*). Braulio Carrillo National Park, Costa Rica **8** Cannaceae (*Canna bangii*). Machu Picchu pueblo, Dto Cuzco, Peru **9** Bromeliaceae (*Pitcairnia arcuata*) Braulio Carrillo National Park, Costa Rica **10** Orchidaceae (*Oerstedella exasperata*) Quijada del Diablo, Prov. Chiriquí, Panama. (Figure **10** from Sekerka et al. 2013).

**Figures 11–14.**
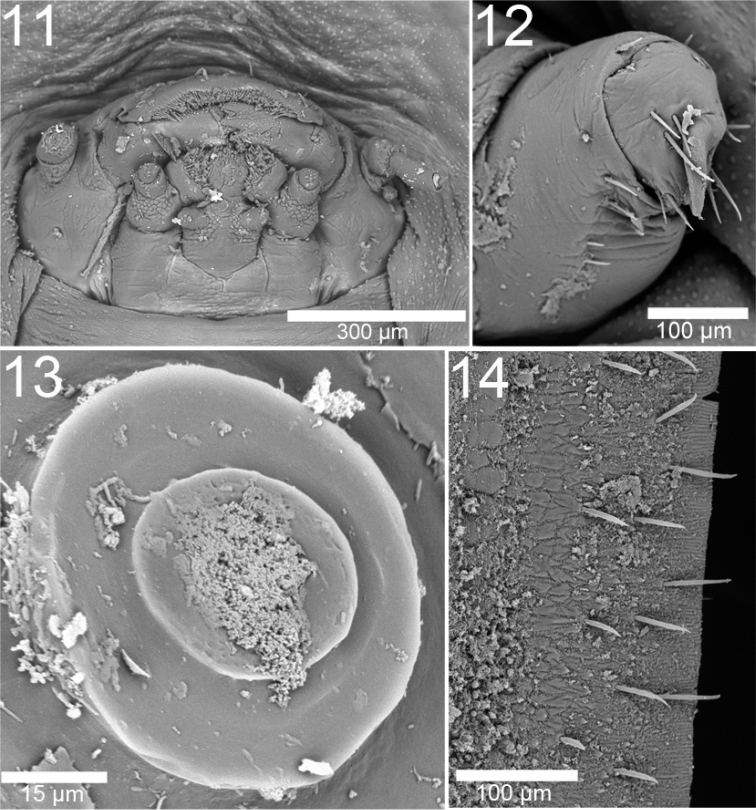
Larva of *Cephaloleia erichsonii*. (La Selva Biological Station, Costa Rica) **11** Head **12** Leg **13** Spiracle **14** dorsal view of setae in the lateral margin.

**Figures 15–18.**
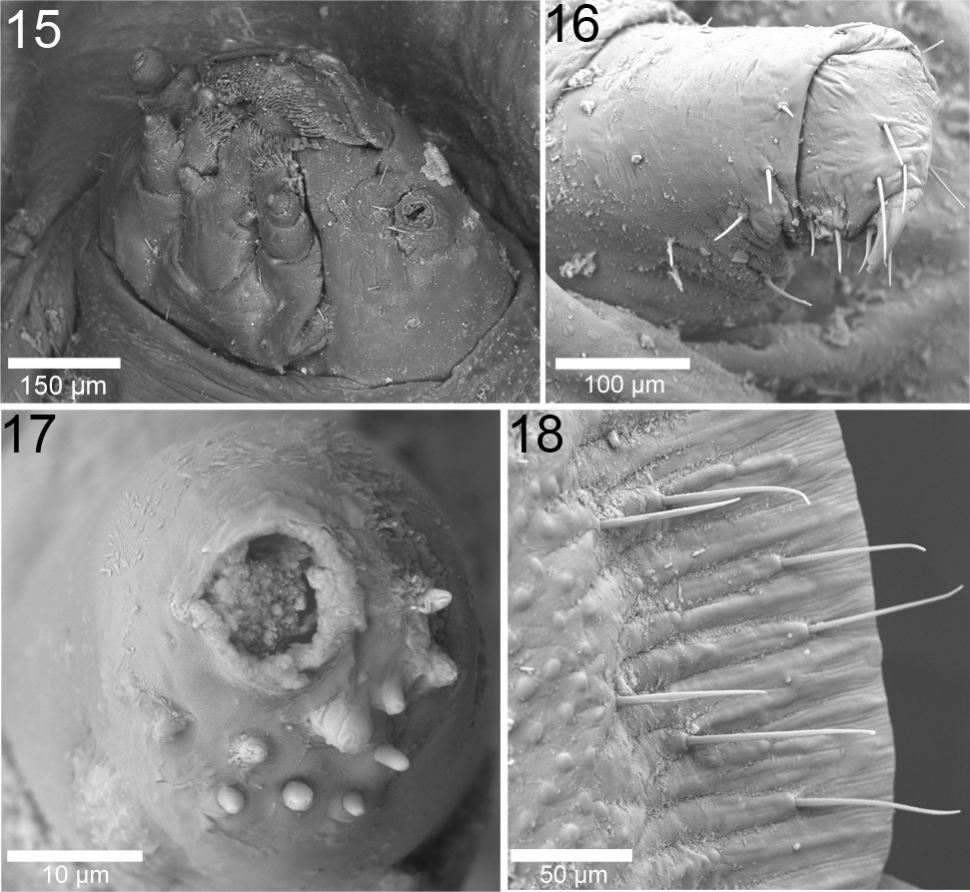
Larva of *Cephaloleia puncticollis*. (Corcovado National Park, Costa Rica) **15** Head **16** Leg **17** Antenna **18** dorsal view of setae in the lateral margin.

**Figures 19–26.**
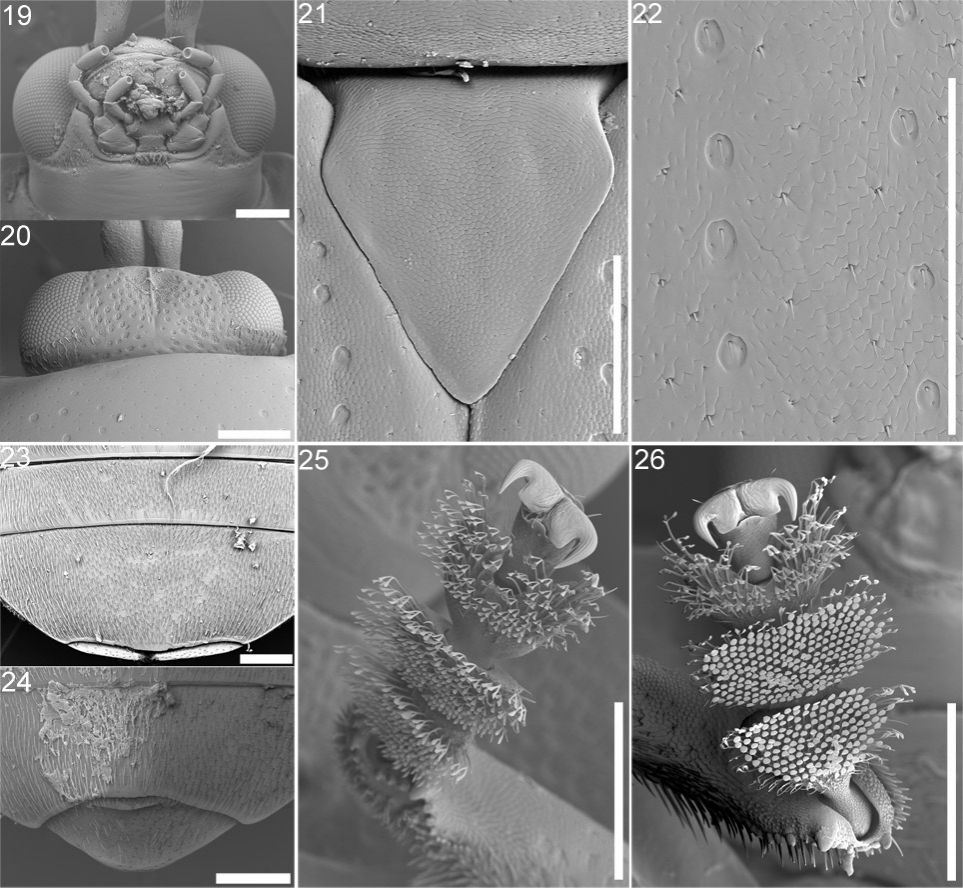
Adult structures and sexual dimorphism in *Cephaloleia* beetles **19** Head and mouth parts (*Cephaloleia belti*) **20** Head, dorsal view showing carina (*Cephaloleia dilaticollis*) **21** Scutellum (*Cephaloleia belti*) **22** impressions and setae on elytron (*Cephaloleia belti*) **23–24** typical sexual dymorphism in last abdominal segment (*Cephaloleia dilaticollis*): **23** Female **24** Male **25–26** Sexual dymorphism in setose attachment pads (*Cephaloleia dilaticollis*) **25** Female tarsa with bifurcate setal tips **26** Male tarsa with bifurcate and discoidal setal tips. All specimens were collected at La Selva Biological Station, Costa Rica. Scale bar equals in all panels equal 200 μm.

**Figures 27–34.**
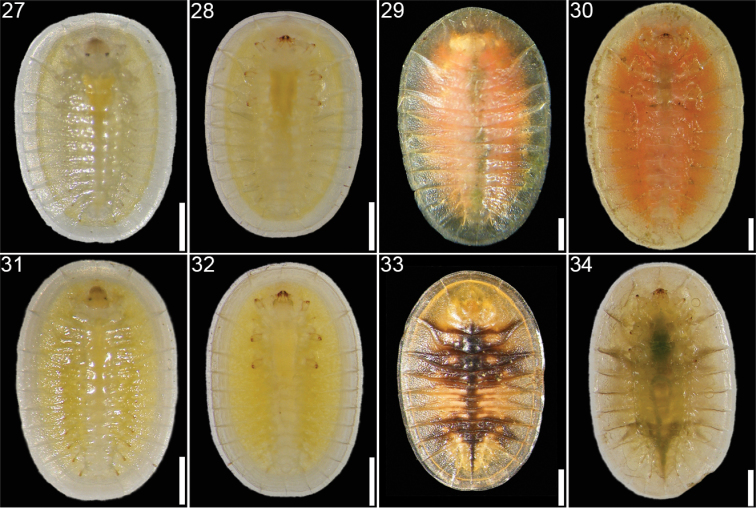
*Cephaloleia* larval stages **27–28** *Cephaloleia belti*, first instar, dorsal and ventral views **29–30** *Cephaloleia belti*, second instar, dorsal and ventral views **31–32** *Cephaloleia dilaticollis*, first instar, dorsal and ventral views **33–34** *Cephaloleia dilaticollis*, second instar, dorsal and ventral views. All specimens collected at La Selva Biological Station, Costa Rica. Scale bars in all panels equal 1 mm.

**Figures 35–42.**
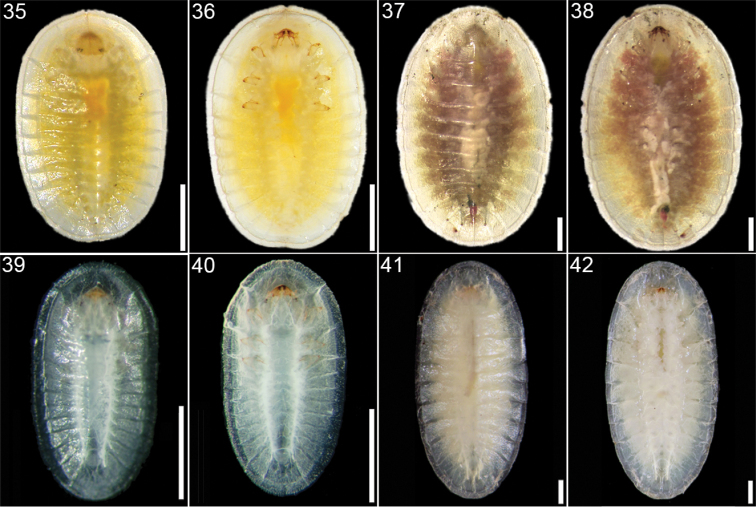
*Cephaloleia* larval stages **35–36** *Cephaloleia dorsalis* at La Selva Biological Station, Costa Rica, first instar, dorsal and ventral views **37–38** *Cephaloleia dorsalis*, second instar, dorsal and ventral views **39–40** *Cephaloleia histrionica* at Braulio Carrillo National Park, 1500 m. elevation, Costa Rica, first instar, dorsal and ventral views **41–42** *Cephaloleia histrionica*, second instar, dorsal and ventral views. Scale bars in all panels equal 1 mm.

**Figures 43–50.**
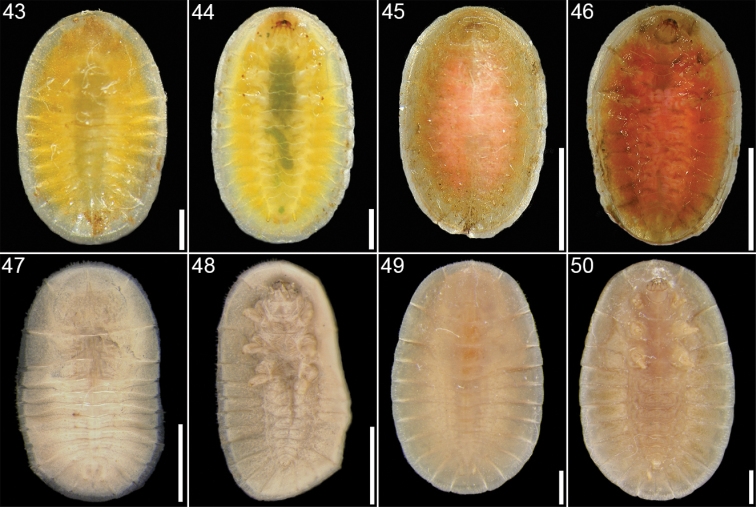
*Cephaloleia* larval stages **43–44** *Cephaloleia placida* at La Selva Biological Station, Costa Rica, first instar, dorsal and ventral views **45–46** *Cephaloleia placida*, second instar, dorsal and ventral views **47–48** *Cephaloleia puncticollis* at Corcovado National Park, Costa Rica, first instar, dorsal and ventral views, specimen preserved in alcohol **49–50** *Cephaloleia puncticollis*, second instar, dorsal and ventral views, specimen preserved in alcohol. Scale bars in all panels equal 1 mm.

**Figures 51–54.**
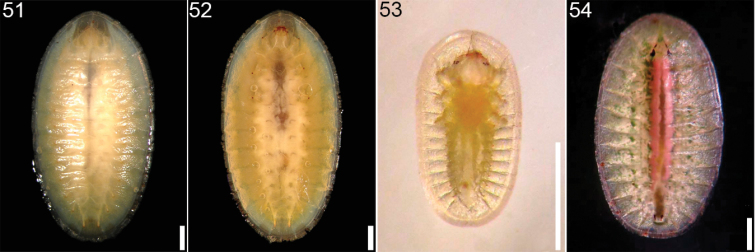
*Cephaloleia* larvae **51–52** *Cephaloleia erichsonii* at La Selva Biological Station, Costa Rica, second instar, dorsal and ventral views **53–54** *Cephaloleia orchideivora* at Quijada del Diablo, Prov. Chiriquí, Panama **53** first instar, dorsal view **54** second instar, dorsal view (Figures **53–54** from Sekerka et al. 2013).

**Figures 55–59.**
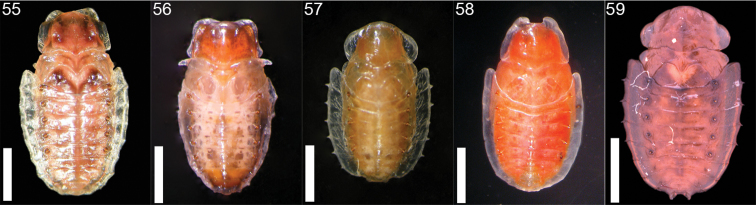
*Cephaloleia* pupae **55** *Cephaloleia belti* (La Selva Biological Station, Costa Rica) **56** *Cephaloleia dilaticollis* (La Selva Biological Station, Costa Rica) **57** *Cephaloleia dorsalis* (La Selva Biological Station, Costa Rica) **58** *Cephaloleia placida* (La Selva Biological Station, Costa Rica) **59** *Cephaloleia puncticollis* (Corcovado National Park, Costa Rica). Scale bars in all panels equal 2 mm.

### Phylogeny and the tempo in the diversification of *Cephaloleia*

A molecular phylogeny that included sequences from the mtDNA CO1 gene, the entire tRNA-Leu, and a portion of the mtDNA COII gene for 98 taxa suggests that *Cephaloleia* as a monophyletic genus (McKenna and Farrell 2005). Although molecular and morphological evidence suggests that *Cephaloleia* is a monophyletic genus, the tempo in diversification of *Cephaloleia* beetles is still in debate.

**Figure 60.**
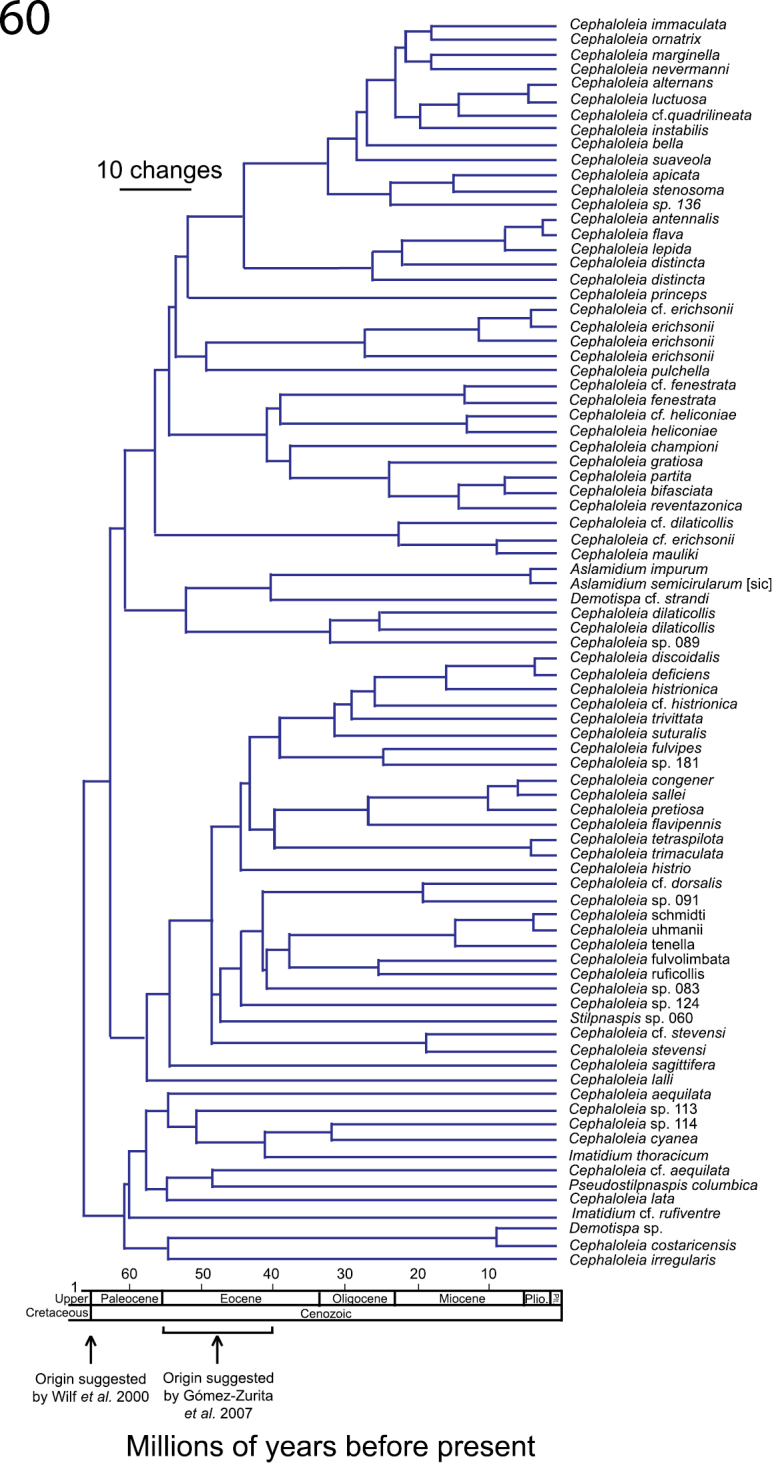
Phylogenetic relationships and two hypotheses in the tempo of evolutionary radiation of *Cephaloleia*. Note in the timeline the proposed origin of *Cephaloleia* by McKenna and Farrell 2006 and Gómez–Zurita et al. 2007. Chronogram modified from McKenna and Farrell 2006.

McKenna and Farrell (2006) proposed an early origin of *Cephaloleia*, i.e., during the Cretaceous – Paleogene boundary event (ca. 65.5 Ma) and a subsequent adaptive radiation during the latest Paleocene – early/middle Eocene (ca. 54.97–43.47) (Fig. 60). Under this scenario, a co-diversification of *Cephaloleia* and their host plants is proposed.

This early origin is controversial, as the evidence used to time early interactions between Zingiberales and ancestors of *Cephaloleia* was the herbivory damage found on fossil leaves of *Zingiberopsis*, an extinct genus of Zingiberaceae (Wilf et al. 2000). To honor the seminal work by D. R. Strong Jr on rolled-leaf beetles, this feeding damage was proposed as an ichnotaxon and named *Cephaloleichnites strongi*.

The assumption that these fossil herbivore bites can be only attributed to an ancestor of *Cephaloleia* beetles is problematic for the following reasons. First, in the study by Wilf et al. (2000), it was assumed that the damage could be the product of ancestors of *Cephaloleia* or *Chelobasis* beetles. However, molecular studies demonstrated that *Chelobasis* is not closely related to the genus *Cephaloleia* (McKenna and Farrell 2006).

Second, herbivory damage similar to the one described on *Zingiberopsis* fossils can be produced by other insects. In a study that predated Wilf et al. (2000), D. R. Strong described in Zingiberales leaves herbivory damage by Coleoptera and Lepidoptera that resembled rolled-leaf beetle herbivory (Auerbach and Strong 1981). In a more recent study we described similar herbivory patterns by larvae of Pyralidae and Choreutidae (Lepidoptera) and *Anopsilus* weevils (Curculionidae, Coleoptera) in four families of extant Zingiberales (García–Robledo and Staines 2008).

Estimates of the tempo of initial diversification of *Cephaloleia* based only on molecular data suggests a more recent origin between 40–55 Ma (Gómez–Zurita et al. 2007) (Fig. 60). This scenario challenges previous hypothesis of co-diversification between *Cephaloleia* and Zingiberales as the diversification of *Cephaloleia* occurred millions of years after the diversification of their Zingiberales host plants (Fig. 60).

### Identification of *Cephaloleia* species using DNA barcodes

DNA barcodes are short DNA sequences (ca. 600 bp) that can be used to identify organisms to the species-level. This technique compares the appropriate DNA barcode loci (e.g., the mitochondrial gene cytochrome oxidase, CO1 in insects) of an unidentified specimen to a known DNA barcode library.

**Figure 61.**
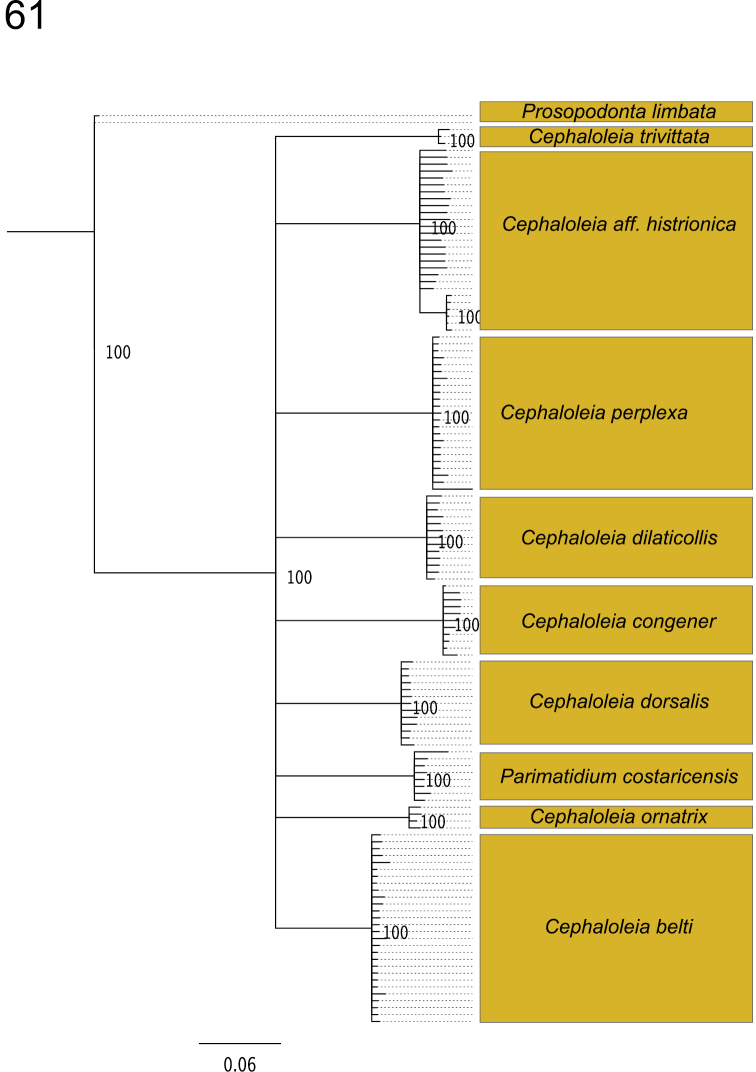
Identification of *Cephaloleia* species using the DNA barcode COI. All specimens were obtained between 600–700 m.a.s.l. in a tropical premontane rain forest in Braulio Carrillo National Park, Costa Rica. Neighbor-joining tree includes bootstrap values (%) supporting species identifications. Boxes group individuals within each species (Modified from García-Robledo et al. in press).

In a study testing the accuracy of DNA barcodes to identify *Cephaloleia* species, we obtained DNA barcodes (CO1 sequences) for multiple individuals representing a whole community of *Cephaloleia* beetles in a tropical montane forest in Costa Rica (García–Robledo et al. 2013a). In this community, the DNA barcode CO1 was able to identify individuals to the species level with 100% accuracy (Figure 61). Species identification of immature *Cephaloleia* species is challenging. However, using the DNA barcode CO1 we successfully identified to the species level immature stages such as eggs and larvae, linking these life stages to their host plant species (García–Robledo et al. 2013a). Future studies must address if the DNA barcode CO1 is also a reliable identification tool in studies at broader geographic scales.

We expect that as the DNA barcode library for *Cephaloleia* beetles becomes more comprehensive, DNA barcodes will play a fundamental role in the discovery of cryptic species and the delimitation of species boundaries. This molecular tool will have a great impact in our understanding of patterns of host plant use, and the geographic distribution of *Cephaloleia* species (García–Robledo et al. 2013a).

### Summary of the biology

The biology of various species of *Cephaloleia* have been studied by Strong (1977a, b, 1982a, b, 1983), Seifert and Seifert (1976a, b, 1979a), Strong and Wang (1977), Auerbach and Strong (1981), Morrison and Strong (1981), Seifert (1982, 1984), Johnson (2004a, b, 2005), Johnson and Horvitz (2005), García–Robledo and Horvitz (2009, 2011), García–Robledo et al. (2010, 2013a, 2013b). Many of the identifications made by earlier authors were incorrect to species but now have been assigned to the correct species (Staines 2004).

Most *Cephaloleia* species feed on the scroll formed by the young rolled leaves of various Zingiberales (Heliconiaceae, Zingiberaceae, Costaceae, Marantaceae, and Cannaceae) (Figs 1, 4–10). This feeding habit gave rise to the name "rolled-leaf Hispinae" (Fig. 1). Rolled leaves are tender and wet, but dry and toughen quickly after unfurling. In small Zingiberales the leaves remain rolled for less than a week but large Zingiberales may have leaves which remain rolled for as long as 25 days. In some species, adult and larvae of *Cephaloleia* also feed on the petioles and inflorescence bracts of their host plants.

*Cephaloleia* beetles evolved in the Neotropics. After the introduction of exotic Zingiberales to America, several species were recorded expanding their diets and completing their life cycle on invasive Zingiberales from India and the Pacific Islands. Paleotropical Zingiberales currently used as hosts by *Cephaloleia* include *Cheilocostus speciosus* (Costaceae), *Musa velutina* (Musaceae), *Alpinia purpurata* and *Alpinia zerumbet* (Zingiberaceae) (García–Robledo and Horvitz 2012). When Neotropical Zingiberales are introduced to a new locality, the local species of *Cephaloleia* expand their diets to such novel hosts (García–Robledo and Horvitz 2012). In this monograph we included several records of Central American *Cephaloleia* feeding on Zingiberales recently introduced from South America.

*Cephaloleia* species also feed on non-Zingiberaceous host plants. Adult *Cephaloleia* were recorded feeding on plants from the families Arecaceae, Bromeliaceae, Orchidaceae, and Poaceae (García–Robledo et al. 2013a; Sekerka et al. 2013). In a tropical montane forest at Las Alturas Biological Station in the Coto Brus province of Costa Rica, near the Panama border, we recorded adults of *Cephaloleia stevensi* feeding on *Tradescantia zanonia* (Commelinaceae). Larvae of *Cephaloleia* were recorded feeding on Arecaceae, Bromeliaceae, and Orchidaceae (Fig. 9) (García–Robledo et al. 2013a; Sekerka et al. 2013).

*Cephaloleia* eggs are flat (Fig. 2), with a thin chorion so they are subject to desiccation. Eggs are laid on host surfaces. Oviposition sites vary among beetle species and host plant. The most common oviposition sites are leaf surfaces, petioles of immature leaves or inflorescence bracts. Eggs hatch after 10 to 20 days.

Larvae begin feeding immediately upon the part of the plant on which the egg was laid. *Cephaloleia* larvae have a water penny-like appearance (Fig. 3). They are flat and well adapted to moving between the wet surfaces of Zingiberales leaves and stems. Larvae grow very slowly and have two instars (Figs 27–54). Time from larval eclosion to pupation is about 30 to 60 days (Figs 55–59). During their development, larvae of leaf and stem-feeding species utilize several leaves. Inflorescence-feeding larvae are restricted to a single inflorescence. The larvae of *Cephaloleia neglecta* and *Cephaloleia puncticollis* have a setose venter which may be an adaptation to life in water inside the bracts. They do not possess the abdominal gills or anal filaments of Psephenidae (Coleoptera, water penny beetles) which they resemble. *Cephaloleia* species are not leaf-miners; they feed on the plant by dragging their mandibles across the plant surface while they crawl forward. This leaves an irregularly shaped feeding scar and a trail of frass. Pupation occurs above ground usually on the stalk of the host plant and lasts about 20 days.

Adult *Cephaloleia* species are found in the same habitat as larvae. Females are usually larger than males. *Cephaloleia* frequently display sexual dimorphism in the last abdominal segment (Figs 23–24). Tarsal papillae are also sexually dimorphic. Females display bifurcated tarsal papillae (Fig. 25). Males display a combination of bifurcated and discoid papillae (Fig. 26).

### Key to the described larvae of *Cephaloleia*

**Table:** 

1	Dorsum with medial ridge from anterior to posterior margins	2
–	Dorsum without medial ridge from anterior to posterior margins; Costa Rica, Guatemala, Panama	*Cephaloleia histrionica* Baly (Figs 39–42)
2(1)	Dorsal medial ridge setose	6
–	Dorsal medial ridge non-setose	3
3(2)	Dorsum with surface shallowly rugose; Panama	*Cephaloleia orchideivora* Sekerka et al. (Figs 53–54)
–	Dorum with surface punctate	4
4(3)	Metanotum without sulcus or carina	5
–	Metanotum with transverse sulcus near base; Costa Rica, Nicaragua, Panama	*Cephaloleia puncticollis* Baly (Figs 47–50, 15–18)
5(4)	Pronotum with two diagonal carinae laterally; abdominal tergites 7–9 with two diagonal carinae; Costa Rica, Guatemala, Panama	*Cephaloleia dorsalis* Baly (Figs 35–38)
–	Pronotum without two diagonal carinae laterally; abdominal tergites 7–9 without two diagonal carinae; Brazil, Colombia, Costa Rica, Panama, Peru	*Cephaloleia erichsonsii* Baly (Figs 51–52, 11–14)
6(2)	Mesonotum without carina; Colombia, Costa Rica, Panama	*Cephaloleia placida* Baly (Figs 43–46)
–	Mesonotum with carina	7
7(6)	Mesonotum with medial V-shaped carina; metanotum irregularly plicate medially; Costa Rica, Guatemala, Honduras, Mexico, Nicaragua, Panama	*Cephaloleia belti* Baly (Figs 27–30)
–	Mesonotum with carina on outer margin; metanotum with diagonal carina on each side of middle; Bolivia, Brazil, Colombia, Costa Rica, Ecuador, Mexico, Nicaragua, Panama, Peru, Venezuela	*Cephaloleia dilaticollis* Baly (Figs 31–34)

### Clave para las larvas descritas de *Cephaloleia*

**Table:** 

1	Dorso con cresta medial desde el margen anterior hasta el margen posterior	2
–	Dorso sin cresta desde el margen anterior hasta el margen posterior; Costa Rica, Guatemala, Panamá	*Cephaloleia histrionica* Baly (Figs 39–42)
2(1)	Cresta medial dorsal setosa	6
–	Cresta medial dorsal no setosa	3
3(2)	Dorso con rugosidades poco profundas; Panamá	*Cephaloleia orchideivora* Sekerka et al. (Figs 53–54)
–	Dorso puntuado	4
4(3)	Metanoto sin sulco o carina	5
–	Metanoto con sulco transversal cerca de la base; Costa Rica, Nicaragua, Panamá	*Cephaloleia puncticollis* Baly (Figs 47–50, 15–18)
5(4)	Pronoto con dos carinas diagonales en los flancos laterales; terguitos abdominales 7–9 con dos carinas diagonales; Costa Rica, Guatemala, Panamá	*Cephaloleia dorsalis* Baly (Figs 35–38)
–	Pronoto sin dos carinas diagonales en los flancos laterales; terguitos abdominales 7–9 sin dos carinas diagonales; Brasil, Colombia, Costa Rica, Panamá, Perú.	*Cephaloleia erichsonii* Baly (Figs 51–52, 11–14)
6(2)	Metanoto sin carina; Colombia, Costa Rica, Panamá	*Cephaloleia placida* Baly (Figs 43–46)
–	Metanoto con carina	7
7(6)	Mesonoto con carina medial en forma de V: metanoto con pliegues mediales irregulares; Costa Rica, Guatemala, Honduras, México, Nicaragua, Panamá	*Cephaloleia belti* Baly (Figs 27–30)
–	Mesonoto con carina sobre el margen externo; metanoto con carina diagonal en los flancos medios; Bolivia, Brasil, Colombia, Costa Rica, Ecuador, México, Nicaragua, Panamá, Perú, Venezuela	*Cephaloleia dilaticollis* Baly (Figs 31–34)

### Key to the *Cephaloleia* species of Mexico, Central America and the West Indies

**Table:** 

1	Elytral punctures in regular rows (at least on basal ½)	2
–	Elytral punctures irregular; Costa Rica	*Cephaloleia irregularis* Uhmann (Fig. 169)
2(1)	Body convex, rounded	3
–	Body more or less flattened	4
3(2)	Vertex of head densely punctate; pronotum with lateral margin evenly arcuate; antennomere 1 clavate, 2 ½ length of 1; Cuba	*Cephaloleia barroi* Uhmann (Fig. 82)
–	Vertex of head sparsely punctate; pronotum with lateral margin straight for basal ⅓ then rounding to anterior angle; antennomeres 1–2 transverse, subequal in length; Jamaica	*Cephaloleia sandersoni* Staines (Fig. 230)
4(2)	Apex of elytra truncate; lateral margin of pronotum serrate; smaller species (<5.5 mm); elytral puncture rows converge and unite apically; Belize, Colombia, Panama	*Cephaloleia formosus* Staines (Fig. 145)
–	Apex of elytra rounded; lateral margin of pronotum smooth	5
5(4)	Additional punctures present between elytral puncture rows 7 and 8; Panama	*Cephaloleia orchideivora* Sekerka et al. (Fig. 201)
–	Additional punctures absent between elytral puncture rows 7 and 8	6
6(5)	Lateral margin of elytra smooth	7
–	Lateral margin of elytra denticulate; Colombia, Costa Rica, Venezuela	*Cephaloleia cyanea* Staines (Fig. 109)
7(6)	Apical margin of elytra denticulate	8
–	Apical margin of elytra smooth	13
8(7)	Dorsum unicolorous	9
–	Dorsum bicolorous	10
9(8)	Entirely black; elytral puncture rows converging and uniting apically; Mexico	*Cephaloleia punctatissima* Weise (Fig. 218)
–	Dorsum metallic blue; elytral puncture rows obsolete apically; Costa Rica	*Cephaloleia gilvipes* Uhmann (Fig. 152)
10(8)	Pronotum red	11
–	Pronotum yellow with black longitudinal vitta; Costa Rica	*Cephaloleia deficiens* Baly (Fig. 112)
11(10)	Vertex of head concave between eyes	12
–	Vertex of head flat between eyes; Costa Rica, Mexico	*Cephaloleia atriceps* Pic (Fig. 80)
12(11)	Suture between abdominal sterna 1 and 2 obsolete medially; Belize, Costa Rica, El Salvador, Guatemala, Honduras, Mexico, Nicaragua	*Cephaloleia ruficollis* Baly (Fig. 226)
–	Suture between abdominal sterna 1 and 2 complete; Costa Rica, Panama	*Cephaloleia schmidti* Uhmann (Fig. 232)
13(7)	Elytra with declivity beginning just behind humerus at puncture row 7	14
–	Elytra without declivity beginning just behind humerus at puncture row 7	41
14(13)	Vertex of head concave between eyes	15
–	Vertex of head flat between eyes	27
15(14)	Elytral puncture rows obsolete apically	16
–	Elytral puncture rows distinct apically	18
16(15)	Elytra expanding apically; Costa Rica, Panama	*Cephaloleia quadrilineata* Baly (Fig. 220)
–	Elytra parallel-sided	17
17(16)	Antennomeres 1 and 2 elongate; Guatemala, Mexico	*Cephaloleia suaveola* Baly (Fig. 243)
–	Antennomere 1 incrassate, 2 transverse; Costa Rica, Panama	*Cephaloleia nevermanni* Uhmann (Fig. 191)
18(15)	Elytra vittate	19
–	Elytra unicolorous or maculate	20
19(18)	Pronotum impunctate; antennomeres 3–4 triangular; Colombia, Costa Rica, Mexico, Panama	*Cephaloleia bella* Baly (Fig. 84)
–	Pronotum punctate laterally; antennomere 2 triangular; Costa Rica, Guatemala, Mexico, Panama	*Cephaloleia vicina* Baly (Fig. 266)
20(18)	Dorsum unicolorous	21
–	Dorsum bicolorous	23
21(20)	Pronotum with disc impunctate, punctate laterally; large (>7.0 mm)	22
–	Pronotum densely punctate; smaller (<5.0 mm); Belize, Guatemala, Honduras, Mexico	*Cephaloleia fulvolimbata* Baly (Fig. 149)
22(21)	Elytral puncture rows confused at apex; Costa Rica, Panama	*Cephaloleia flava* Uhmann (Fig. 141)
–	Elytral puncture rows distinct at apex; Costa Rica, Mexico, Panama	*Cephaloleia gratiosa* Baly (in part) (Fig. 154)
23(20)	Pronotum and elytra same color	24
–	Pronotum and elytra different colors	25
24(23)	Suture between abdominal sterna 1 and 2 obsolete medially; Costa Rica, Panama	*Cephaloleia nigropicta* Baly (Fig. 195)
–	Suture between abdominal sterna 1 and 2 complete; Bolivia, Ecuador, Panama, Peru	*Cephaloleia laeta* Waterhouse (Fig. 171)
25(23)	Suture between abdominal sterna 1 and 2 obsolete medially; Bolivia, Brazil, Colombia, Costa Rica, Panama, Ecuador, Peru, Venezuela	*Cephaloleia pretiosa* Baly (Fig. 214)
–	Suture between abdominal sterna 1 and 2 complete	26
26(25)	Pronotum impunctate medially, punctate laterally; antennomere 1 clavate; Costa Rica, Mexico, Panama	*Cephaloleia gratiosa* Baly (in part) (Fig. 154)
–	Pronotum sparsely punctate; antennomere 1 elongate; Panama	*Cephaloleia lepida* Staines (Fig. 175)
27(14)	Dorsum unicolorous	28
–	Dorsum bicolorous	30
28(27)	Elytra with sutural angle without tooth; pronotum without impression laterally	29
–	Elytra with sutural angle with tooth; pronotum with impression laterally; Costa Rica, Guatemala, Mexico, Panama	*Cephaloleia instabilis* Baly (in part) (Fig. 166)
29(28)	Pronotum finely punctate; suture between abdominal sterna 1 and 2 complete; antennomere 1 subequal in length to 3; Costa Rica, Guatemala, Nicaragua, Panama	*Cephaloleia congener* Baly (Fig. 103)
–	Pronotum sparsely punctate; suture between abdominal sterna 1 and 2 obsolete medially; antennomere 1 subequal in length to 2–4; Costa Rica	*Cephaloleia immaculata* Staines (Fig. 163)
30(27)	Elytron immaculate	31
–	Elytron maculate or vittate	32
31(30)	Pronotum with impression laterally; antennomeres 1–4 compressed laterally; Costa Rica, Guatemala, Mexico, Panama	*Cephaloleia instabilis* Baly (in part) (Fig. 166)
–	Pronotum without impression laterally; antennomere 1 compressed laterally; Costa Rica, Mexico	*Cephaloleia fulvicollis* Weise (Fig. 147)
32(30)	Elytron with longitudinal vitta on disc or lateral margin	33
–	Elytron without longitudinal vitta on disc or lateral margin	36
33(32)	Elytron with longitudinal vitta on lateral margin; Costa Rica, Panama	*Cephaloleia marginella* Uhmann (Fig. 184)
–	Elytron with longitudinal vitta on disc	34
34(33)	Suture between abdominal sterna 1 and 2 obsolete medially; Costa Rica, Panama	*Cephaloleia heliconiae* Uhmann (Fig. 157)
–	Suture between abdominal sterna 1 and 2 complete	35
35(34)	Pronotum punctate; antennomeres 2–4 triangular; Costa Rica, Panama	*Cephaloleia championi* Baly (Fig. 95)
–	Pronotum sparsely punctate; antennomeres 2–3 triangular; Colombia, Panama	*Cephaloleia luctuosa* Guérin-Méneville (Fig. 179)
36(32)	No antennomeres triangular	37
–	Some antennomeres triangular	40
37(36)	Suture between abdominal sterna 1 and 2 obsolete medially; Costa Rica, Panama	*Cephaloleia fenestrata* Weise (Fig. 139)
–	Suture between abdominal sterna 1 and 2 complete	38
38(37)	Pronotum nearly impunctate	39
–	Pronotum with disc impunctate, punctate laterally; elytral punctures larger on disc than laterally; puncture row 10 distant from lateral margin; Costa Rica, Guatemala, Panama	*Cephaloleia histrionica* Baly (Fig. 159)
39(38)	Vertex of head punctate; antennomeres 1–4 compressed laterally; pronotum immaculate; Costa Rica	*Cephaloleia stainesi* García-Robledo, sp. n. (Fig. 273)
–	Vertex of head impunctate; antennomeres 1–4 not compressed laterally; pronotum maculate; Costa Rica, Nicaragua	*Cephaloleia reventazonica* Uhmann (Fig. 223)
40(36)	Antennomere 3 triangular; Panama	*Cephaloleia leucoxantha* Baly (Fig. 176)
–	Antennomeres 2–4 triangular; Costa Rica, Guatemala, Mexico, Panama	*Cephaloleia instabilis* Baly (in part) (Fig. 166)
41(13)	Dorsum unicolorous	42
–	Dorsum bicolorous	75
42(41)	Elytra maculate or vittate	43
–	Elytra unicolorous	54
43(42)	Vertex of head concave between eyes	44
–	Vertex of head flat between eyes	47
44(43)	Pronotum strongly densely punctate; suture between abdominal sterna 1 and 2 obsolete medially; Costa Rica, Panama	*Cephaloleia stevensi* Baly (Fig. 240)
–	Pronotum moderately punctate; suture between abdominal sterna 1 and 2 complete	45
45(44)	Antennomere 1 longer than 2	46
–	Antennomere 1 subequal to 2; pronotum without V-shaped depression basally; Costa Rica	*Cephaloleia interrupta* García-Robledo & Staines, sp. n. (Fig. 167)
46(45)	Antennomere 1 subequal in length to 3; basic body color metallic green; Panama	*Cephaloleia eumorpha* Staines (Fig. 134)
–	Antennomere 1 subequal to 2–4 combined; basic body color black; Mexico	*Cephaloleia postuma* Weise (Fig. 212)
47(43)	Suture between abdominal sterna 1 and 2 complete	48
–	Suture between abdominal sterna 1 and 2 obsolete medially	51
48(47)	Antennomere 1 subequal in length to 2; Costa Rica, Guatemala, Panama	*Cephaloleia dorsalis* Baly (Fig. 127)
–	Antennomere 1 more than length of 2	49
49(48)	Antennomere 1 as long as 2–4 combined	50
–	Antennomere 1 shorter than 2–4 combined; Costa Rica, Guatemala, Nicaragua	*Cephaloleia suturalis* Baly (Fig. 248)
50(49)	Elytral puncture rows obsolete after middle; vertex of head without medial sulcus; pro-, meso-, and metasterna impunctate; Costa Rica	*Cephaloleia adusta* Uhmann (Fig. 64)
–	Elytral puncture rows complete; vertex of head with medial sulcus; pro-, meso-, and metasterna impunctate medially, punctate laterally; Costa Rica	*Cephaloleia kressi* García-Robledo, sp. n. (Fig. 272)
51(47)	Antennomere 1 transverse; Mexico	*Cephaloleia chevrolatii* Baly (Fig. 96)
–	Antennomere 1 elongate or clavate	52
52(51)	Antennomere 1 clavate; elytra with humerus impunctate	53
–	Antennomere 1 elongate; elytra with humerus punctate; Brazil, Costa Rica, Panama	*Cephaloleia elegantula* Baly (Fig. 129)
53(51)	Antennomere 1 as long as 2 and 3 combined; elytra with sutural angle without tooth; Costa Rica, Guatemala, Panama	*Cephaloleia stenosoma* Baly (in part) (Fig. 239)
–	Antennomere 1 as long 3; elytra with sutural angle with minute tooth; Bolivia, Colombia, Panama, Peru, Venezuela	*Cephaloleia partita* Weise (Fig. 206)
54(42)	Dorsum metallic blue, pronotum with lateral margin paler; Costa Rica, Guatemala, Nicaragua, Panama	*Cephaloleia metallescens* Baly (Fig. 188)
–	Dorsum black or reddish-brown	55
55(54)	Vertex of head concave between eyes	56
–	Vertex of head flat between eyes	63
56(55)	Dorsum black; Mexico	*Cephaloleia punctatissima* Weise (Fig. 218)
–	Dorsum reddish-brown	57
57(56)	Suture between abdominal sterna 1 and 2 complete	58
–	Suture between abdominal sterna 1 and 2 obsolete medially	59
58(57)	Larger species (more than 5.0 mm)	60
–	Smaller species (less than 4.0 mm); Dominica, Grenada	*Cephaloleia simplex* Staines (Fig. 236)
59(58)	Pronotum with disc punctate; Colombia, Costa Rica, Panama	*Cephaloleia distincta* Baly (Fig. 125)
–	Pronotum with disc impunctate; Colombia, Costa Rica, Panama	*Cephaloleia placida* Baly (Fig. 210)
60(58)	Antennomere 1 subequal in length to 2; Costa Rica, Panama	*Cephaloleia sulciceps* Baly (Fig. 246)
–	Antennomere 1 longer than 2	61
61(60)	Antennomere 1 subequal in length to 2–3 combined; vertex of head with medial carina; size larger (8.0 mm); Costa Rica, Panama	*Cephaloleia mauliki* Uhmann (Fig. 169)
–	Antennomere 1 not subequal in length to 2–3 combined; vertex of head without medial carina; size smaller (<6.0 mm)	62
62(61)	Antennomere 1 subequal in length to 3; pronotum densely punctate; Trinidad	*Cephaloleia brunnea* Staines (Fig. 90)
–	Antennomere 1 not subequal in length to 3; pronotum sparsely punctate; Trinidad	*Cephaloleia rubra* Staines (Fig. 225)
63(55)	Dorsum black; Costa Rica, El Salvador, Guatemala, Mexico, Nicaragua, Panama	*Cephaloleia tenella* Baly (Fig. 250)
–	Dorsum reddish-brown	64
64(63)	Body elongate, rounded	66
–	Body nearly rectangular in outline	65
65(64)	Elytra with puncture rows 6–9 obsolete on humerus; suture between abdominal sterna 1 and 2 complete; Costa Rica, Guatemala	*Cephaloleia aequilata* Uhmann (Fig. 66)
–	Elytra with puncture rows 6–9 present on humerus; suture between abdominal sterna 1 and 2 obsolete medially; Bolivia, Brazil, Colombia, Costa Rica, Ecuador, Mexico, Nicaragua, Panama, Peru, Venezuela	*Cephaloleia dilaticollis* Baly (Fig. 119)
66(65)	Suture between abdominal sterna 1 and 2 complete	67
–	Suture between abdominal sterna 1 and 2 obsolete medially	70
67(66)	Antennomeres 1–2 subglobose; Costa Rica, Panama	*Cephaloleia cylindrica* Staines (Fig. 110)
–	Antennomeres 1–2 elongate	68
68(66)	Antennomere 1 subequal to 2–4 combined, clavate; Costa Rica, Guatemala, Panama	*Cephaloleia antennalis* Donckier (Fig. 72)
–	Antennomere 1 shorter than 2–4 combined, elongate	69
69(68)	Pronotum with disc punctate; pro-, meso-, and metasterna impunctate medially, punctate laterally; Costa Rica, Nicaragua, Panama	*Cephaloleia puncticollis* Baly (Fig. 219)
–	Pronotum with disc impunctate; pro-, meso-, and metasterna impunctate; Costa Rica, Guatemala, Mexico, Panama	*Cephaloleia sallei* Baly (Fig. 229)
70(66)	Elytra with puncture rows confused basally; Belize, Costa Rica, Guatemala, Mexico	*Cephaloleia perplexa* Baly (Fig. 208)
–	Elytra with puncture rows distinct basally	71
71(70)	Antennomere 1 at least 2× length of 2	72
–	Antennomere 1 less than 2× length of 2	74
72(71)	Elytra with sulcus on humeral callus; Brazil, Colombia, Costa Rica, Panama, Peru	*Cephaloleia erichsonii* Baly (Fig. 132)
–	Elytra without sulcus on humeral callus	73
73(72)	Antennomere 1 clavate, 2× length of 2; elytral punctures obovate, not larger on disc; Costa Rica	*Cephaloleia conforma* García-Robledo & Staines, sp. n. (Fig. 102)
–	Antennomere 1 elongate, 3× length of 2; elytral punctures rounded, larger on disc; Costa Rica	*Cephaloleia calathae* García-Robledo & Staines, sp. n. (Fig. 93)
74(71)	Elytra with puncture rows confused apically; antennomere 1 longer than 2; Bolivia, Brazil, Colombia, Costa Rica, Ecuador, French Guiana, Guatemala, Honduras, Mexico, Panama, Peru, Venezuela	*Cephaloleia nigricornis* Fabricius (Fig. 193)
–	Elytra with puncture rows distinct apically; antennomere 1 subequal in length to 2; Mexico	*Cephaloleia delectabilis* Staines (Fig. 114)
75(41)	Elytra maculate or vittate	78
–	Elytra unicolorous	76
76(75)	Vertex of head concave between eyes; Bolivia, Brazil, Colombia, Ecuador, Panama, Peru, Venezuela	*Cephaloleia neglecta* Weise (Fig. 190)
–	Vertex of head flat between eyes	77
77(76)	Pronotum darker than elytra; antennomere 1 longer than 2; Panama	*Cephaloleia amblys* Staines (Image not available)
–	Pronotum paler than elytra; antennomere 1 subequal in length to 2; Panama	*Cephaloleia facetus* Staines (Fig. 136)
78(75)	Elytra maculate	80
–	Elytra vittate	84
79(78)	Vertex of head concave between eyes	81
–	Vertex of head flat between eyes	82
80(79)	Color black with reddish humeral macula; Costa Rica, Nicaragua, Panama	*Cephaloleia uhmanni* Staines (Fig. 261)
–	Color different	81
81(80)	Antennomere 1 longer than 2; pronotal punctures more dense laterally; elytral puncture rows confused apically; Colombia, Costa Rica, Ecuador, French Guiana, Panama, Venezuela	*Cephaloleia trimaculata* Baly (Fig. 256)
–	Antennomere 1 subequal to 2; pronotal punctures uniformly distributed; elytral puncture rows obsolete apically; Costa Rica, Panama	*Cephaloleia weisei* Staines (Fig. 269)
82(80)	Pronotum with disc impunctate, punctate laterally; antennomere 1 clavate, longer than 2 and 3 combined; Costa Rica, Guatemala, Panama	*Cephaloleia stenosoma* Baly (in part) (Fig. 239)
–	Pronotum punctate; antennomere 1 elongate, not longer than 2 and 3 combined	83
83(82)	Antennomere 1 subequal in length to 2; pronotum with basal impression; Costa Rica, Nicaragua, Panama	*Cephaloleia splendida* Staines (Fig. 237)
–	Antennomere 1 twice length of 2; pronotum with lateral impression; Costa Rica, Panama	*Cephaloleia turrialbana* Uhmann (Fig. 260)
84(79)	Vertex of head concave between eyes	85
–	Vertex of head flat between eyes	91
85(84)	Humerus nearly impunctate	86
–	Humerus punctate	87
86(85)	Suture between abdominal sterna 1 and 2 obsolete medially; sutural angle of elytra with small tooth; Panama	*Cephaloleia scitulus* Staines (Fig. 233)
–	Suture between abdominal sterna 1 and 2 complete; sutural angle of elytra without small tooth; Mexico, Peru, Venezuela	*Cephaloleia parenthesis* Weise (Fig. 205)
87(85)	Elytral puncture rows nearly obsolete apically	88
–	Elytral puncture rows distinct apically	89
88(87)	Antennomere 1 incrassate, subequal in length to 2–4 combined; Costa Rica, Nicaragua, Panama	*Cephaloleia ornatrix* Donckier (Fig. 203)
–	Antennomere 1 elongate, shorter than 2–4 combined; Mexico	*Cephaloleia presignis* Staines (Fig. 213)
89(87)	Antennomere 1 subequal in length to 2–4 combined; Costa Rica, Mexico	*Cephaloleia separata* Baly (Fig. 235)
–	Antennomere 1 ⅓ length of 2–4 combined	90
90(89)	Elytra with additional row of punctures after row 7; sutural angle of elytra notched; suture between abdominal sterna 1 and 2 complete; Costa Rica, Panama	*Cephaloleia apicata* Uhmann (Fig. 75)
–	Elytra without additional row of punctures after row 7; sutural angle of elytra rounded; suture between abdominal sterna 1 and 2 obsolete medially; Costa Rica	*Cephaloleia disjuncta* Staines (Fig. 124)
91(84)	Elytra with sutural angle emarginate; Guatemala	*Cephaloleia lateralis* Baly (Fig. 172)
–	Elytra with sutural angle rounded	92
92(91)	Elytra with humerus impunctate	94
–	Elytra with humerus punctate	93
93(92)	Antennomere 1 as long as 2–3 combined; Guatemala, Mexico Honduras, Panama	*Cephaloleia discoidalis* Baly (Fig. 123)
–	Antennomere 1 shorter than 2–3 combined; Costa Rica, Guatemala, Panama	*Cephaloleia stenosoma* Baly (in part) (Fig. 239)
94(92)	Elytra with puncture rows obsolete apically	95
–	Elytra with puncture rows distinct apically	97
95(94)	Antennomere 1 subequal to 3; suture between abdominal sterna 1 and 2 obsolete medially; Costa Rica, Nicaragua, Panama	*Cephaloleia trivittata* Baly (Fig. 257)
–	Antennomere 1 3× length of 3; suture between abdominal sterna 1 and 2 complete	96
96(95)	Pronotum impunctate; pro-, meso-, and metasterna punctate laterally; Costa Rica	*Cephaloleia triangularis* Staines (Fig. 254)
–	Pronotum punctate laterally; pro-, meso-, and metasterna impunctate; Panama	*Cephaloleia erugatus* Staines (Fig. 133)
97(94)	Antennomere 1 subequal in length to 2	98
–	Antennomere 1 much longer than 2	99
98(97)	Antennomere 1 subequal to 3; 2 transverse; pronotal punctures dense, uniform; Costa Rica, Panama	*Cephaloleia semivittata* Baly (Fig. 234)
–	Antennomere 1 subequal in length to 2; 2 elongate; pronotum irregularly punctate; Costa Rica	*Cephaloleia vittata* Staines (Image not available)
99(97)	Antennomere 1 as long as 2–4 combined; pro-, meso-, and metasterna impunctate medially, punctate laterally	100
–	Antennomere 1 as long as 2–3 combined; pro-, meso-, and metasterna impunctate; Belize, Costa Rica, Guatemala, Panama	*Cephaloleia consanguinea* Baly (Fig. 104)
100(99)	Sutural angle of elytra with small tooth; puncture rows distinct apically; Costa Rica, Guatemala, Honduras, Mexico, Nicaragua, Panama	*Cephaloleia belti* Baly (Fig. 85)
–	Sutural angle of elytra without small tooth; puncture rows converge and unite apically; Colombia, Panama	*Cephaloleia variabilis* Staines (Fig. 265)

### Clave para las especies de *Cephaloleia* de México, Centro América y las Indias Occidentales

**Table:** 

1	Puntuaciones elitrales en filas regulares (al menos en la mitad basal)	2
–	Puntuaciones elitrales irregulares; Costa Rica	*Cephaloleia irregularis* Uhmann (Fig. 169)
2(1)	Cuerpo convexo, redondeado	3
–	Cuerpo más o menos aplanado	4
3(2)	Vértice de la cabeza densamente puntuado; pronoto con márgenes laterales homogéneamente recurvadas; antenómero 1 clavado, 2 ½ veces la longitud de 1; Cuba	*Cephaloleia barroi* Uhmann (Fig. 82)
–	Vértice de la cabeza escasamente puntuado; pronoto con márgenes laterales rectas desde ⅓ de la base, luego redondeados hacia el ángulo anterior; antenómeros 1–2 transversos, subiguales en longitud; Jamaica	*Cephaloleia sandersoni* Staines (Fig. 230)
4(2)	Apice de los élitros truncados; márgen lateral del pronoto aserrado; especie más pequeña (<5.5 mm); puntuaciones elitrales en filas que convergen y se unen apicalmente; Belice, Colombia, Panamá	*Cephaloleia formosus* Staines (Fig. 145)
–	Apice de los élitros redondeados; márgenes laterales del pronoto liso	5
5(4)	Puntuaciones elitrales adicionales presentes entre las filas de filas de puntuaciones elitrales 7 y 8; Panamá	*Cephaloleia orchideivora* Sekerka et al. (Fig. 201)
–	Puntuaciones adicionales ausentes entre filas de puntuaciones elitrales 7 y 8	6
6(5)	Márgenes laterales de los élitros lisos	7
–	Márgenes laterales de los élitros denticuladas; Colombia, Costa Rica, Venezuela	*Cephaloleia cyanea* Staines (Fig. 109)
7(6)	Márgenes apicales de los élitros denticuladas	8
–	Márgenes apicales de los élitros lisas	13
8(7)	Dorso unicolor	9
–	Dorso bicolor	10
9(8)	Completamente negro; puntuaciones de los élitros en filas que convergen y se unen en el ápice; México	*Cephaloleia punctatissima* Weise (Fig. 218)
–	Dorso azul metálico; filas de puntuaciones de los élitros obsoletas apicálmente; Costa Rica	*Cephaloleia gilvipes* Uhmann (Fig. 152)
10(8)	Pronoto rojo	11
–	Pronoto amarillo con líneas longitudinales negras; Costa Rica	*Cephaloleia deficiens* Baly (Fig. 112)
11(10)	Vértice de la cabeza cóncavo entre los ojos	12
–	Vértice de la cabeza aplanado entre los ojos; Costa Rica, México	*Cephaloleia atriceps* Pic (Fig. 80)
12(11)	Sutura entre el esterno 1 y 2 obsoleta medialmente; Belice, Costa Rica, El Salvador, Guatemala, Honduras, México, Nicaragua	*Cephaloleia ruficollis* Baly (Fig. 226)
–	Sutura entre el esterno 1 y 2 completa; Costa Rica, Panamá	*Cephaloleia schmidti* Uhmann (Fig. 232)
13(7)	Elitros con declive comenzando justo detrás del húmero en la fila de puntuaciones 7	14
–	Elitros sin declive comenzando justo detrás del húmero en la fila de puntuaciones 7	41
14(13)	Vértice de la cabeza cóncavo entre los ojos	15
–	Vértice de la cabeza aplanado entre los ojos	27
15(14)	Puntuaciones elitrales en filas obsoletas apicálmente	16
–	Puntuaciones elitrales en filas diferenciadas apicálmente	18
16(15)	Elitros se expanden apicalmente; Costa Rica, Panamá	*Cephaloleia quadrilineata* Baly (Fig. 220)
–	Elitros con bordes paralelos	17
17(16)	Antenómeros 1 y 2 alargados; Guatemala, México	*Cephaloleia suaveola* Baly (Fig. 243)
–	Antenómero 1 engrosado, 2 transverso; Costa Rica, Panamá	*Cephaloleia nevermanni* Uhmann (Fig. 191)
18(15)	Elitros con líneas	19
–	Elitros de un solo color o con mancha	20
19(18)	Pronoto sin puntuaciones; antenómeros 3–4 triangulares; Colombia, Costa Rica, México, Panamá	*Cephaloleia bella* Baly (Fig. 84)
–	Pronoto puntuado lateralmente; antenómero 2 triangular; Costa Rica, Guatemala, México, Panamá	*Cephaloleia vicina* Baly (Fig. 266)
20(18)	Dorso unicolor	21
–	Dorso bicolor	23
21(20)	Pronoto con disco sin puntuaciones, puntuado lateralmente; grande (>7.0 mm)	22
–	Pronoto densamente puntuado; pequeño (<5.0 mm); Belice, Guatemala, Honduras, México	*Cephaloleia fulvolimbata* Baly (Fig. 149)
22(21)	Líneas de puntuaciones elitrales confusas en el ápice; Costa Rica, Panamá	*Cephaloleia flava* Uhmann (Fig. 141)
–	Líneas de puntuaciones elitrales diferenciables en el ápice; Costa Rica, México, Panamá	*Cephaloleia gratiosa* Baly (en parte) (Fig. 154)
23(20)	Pronoto y élitros del mismo color	24
–	Pronoto y élitros con colores diferentes	25
24(23)	Sutura entre los esternos abodominales 1 y 2 obsoleta medialmente; Costa Rica, Panamá	*Cephaloleia nigropicta* Baly (Fig. 195)
–	Sutura entre esternos 1 y 2 completa; Bolivia, Ecuador, Panamá, Perú	*Cephaloleia laeta* Waterhouse (Fig. 171)
25(23)	Sutura entre los esternos abdominales 1 y 2 obsoleta medialmente; Bolivia, Brasil, Colombia, Costa Rica, Panamá, Ecuador, Perú, Venezuela	*Cephaloleia pretiosa* Baly (Fig. 214)
–	Sutura entre los esternos abdominales 1 y 2 completa	26
26(25)	Pronoto sin puntuaciones en la zona medial, puntuado lateralmente; antenómero 1 clavado; Costa Rica, México, Panamá	*Cephaloleia gratiosa* Baly (en parte) (Fig. 154)
–	Pronoto escasamente puntuado; antenómero 1 alargado; Panamá	*Cephaloleia lepida* Staines (Fig. 175)
27(14)	Dorso unicoloro	28
–	Dorso bicolor	30
28(27)	Elitros con sutura angulada sin diente; pronoto sin impresiones laterales	29
–	Elitros con sutura angulada con diente; pronoto con impresiones laterales; Costa Rica, Guatemala, México, Panamá	*Cephaloleia instabilis* Baly (en parte) (Fig. 166)
29(28)	Pronoto finamente puntuado; sutura entre esternos abdominales 1 y 2 completa; antenómero 1 subigual en longitud a 3; Costa Rica, Guatemala, Nicaragua, Panamá	*Cephaloleia congener* Baly (Fig. 103)
–	Pronoto escasamente puntuado; sutura entre los esternos abdominales 1 y 2 obsoletas medialmente; antenómero 1 subigual en longitud a 2 y 4; Costa Rica	*Cephaloleia immaculata* Staines (Fig. 163)
30(27)	Elitros sin manchas	31
–	Elitros con manchas o líneas	32
31(30)	Pronoto con impresiones laterales; antenómeros 1–4 comprimidos lateralmente; Costa Rica, Guatemala, México, Panamá	*Cephaloleia instabilis* Baly (en parte) (Fig. 166)
–	Pronoto sin impresiones laterales; antenómero 1 comprimido lateralmente; Costa Rica, México	*Cephaloleia fulvicollis* Weise (Fig. 147)
32(30)	Elitros con líneas longitudinales sobre el disco ó las márgenes laterales	33
–	Elitros sin líneas longitudinales sobre el disco ó las márgenes laterales	36
33(32)	Elitros con líneas longitudinales en las márgenes laterales; Costa Rica, Panamá	*Cephaloleia marginella* Uhmann (Fig. 184)
–	Elitros con líneas longitudinales sobre el disco	34
34(33)	Sutura entre esternos abdominales 1 y 2 ausente medialmente; Costa Rica, Panamá	*Cephaloleia heliconiae* Uhmann (Fig. 157)
–	Sutura entre esternos abdominales 1 y 2 completa	35
35(34)	Pronoto puntuado; antenómero 2–4 triangular; Costa Rica, Panamá	*Cephaloleia championi* Baly (Fig. 95)
–	Pronoto escasamente puntuado; antenómero 2–3 triangular; Colombia, Panamá	*Cephaloleia luctuosa* Guérin-Méneville (Fig. 179)
36(32)	Ningún antenómero triangular	37
–	Algunos antenómeros triangulares	40
37(36)	Sutura entre esternos abdominales 1 y 2 obsoletos medialmente; Costa Rica, Panamá	*Cephaloleia fenestrata* Weise (Fig. 139)
–	Sutura entre esternos abdominales 1 y 2 completa	38
38(37)	Pronoto casi sin puntuaciones	39
–	Pronoto con disco sin puntuaciones, puntuaciones laterales presentes; puntuaciones en los élitros más grandes en el disco que lateralmente; puntuaciones de la fila 10 distantes del margen lateral; Costa Rica, Guatemala, Panamá	*Cephaloleia histrionica* Baly (Fig. 159)
39(38)	Vértice de la cabeza puntuada; antenómeros 1–4 comprimidos lateralmente; pronoto sin mancha Costa Rica	*Cephaloleia stainesi* García-Robledo, sp. n. (Fig. 273)
–	Vértice de la cabeza sin puntuaciones; antenómeros 1–4 no comprimidos lateralmente; pronoto sin manchas; Costa Rica, Nicaragua	*Cephaloleia reventazonica* Uhmann (Fig. 223)
40(36)	Antenómero 3 triangular; Panamá	*Cephaloleia leucoxantha* Baly (Fig. 176)
–	Antenómero 2–4 triangular; Costa Rica, Guatemala, México, Panamá	*Cephaloleia instabilis* Baly (en parte) (Fig. 166)
41(13)	Dorso unicolor	42
–	Dorso bicolor	75
42(41)	Elitros de un solo color	43
–	Elitros no manchados ni con líneas	54
43(42)	Vértice de la cabeza cóncavo entre los ojos	44
–	Vértice de la cabeza aplanado entre los ojos	47
44(43)	Pronoto fuerte y densamente puntuado; sutura entre los esternos abdominales 1 y 2 obsoleta medialmente; Costa Rica, Panamá	*Cephaloleia stevensi* Baly (Fig. 240)
–	Pronoto moderadamente puntuado; sutura entre los esternos abdominales 1 y 2 completa	45
45(44)	Antenómero 1 es más largo que 2	46
–	Antenómero 1 subigual a 2; pronoto sin depresión basal en forma de V; Costa Rica	*Cephaloleia interrupta* García-Robledo & Staines, sp. n. (Fig. 167)
46(45)	Antenómero 1 subigual en longitud a 3; color del cuerpo es verde metálico; Panamá	*Cephaloleia eumorpha* Staines (Fig. 134)
–	Antenómero 1 subigual a 2–4 combinados; color del cuerpo es negro; México	*Cephaloleia postuma* Weise (Fig. 212)
47(43)	Sutura entre esternos abdominales 1 y 2 completa	48
–	Sutura entre esternos abdominales 1 y 2 obsoleta medialmente	51
48(47)	Antenómero 1 subigual en longitud a 2; Costa Rica, Guatemala, Panamá	*Cephaloleia dorsalis* Baly (Fig. 127)
–	Antenómero 1 más largo que 2	49
49(48)	Antenómero 1 tan largo como 2–4 combinados;	50
–	Antenómero 1 más corto que 2–4 combinados; Costa Rica, Guatemala, Nicaragua	*Cephaloleia suturalis* Baly (Fig. 248)
50(49)	Filas de puntuaciones elitrales obsoletas en la mitad posterior; vértice de la cabeza sin sulco; pro-, meso-, y metaesternos sin puntuaciones; Costa Rica Costa Rica	*Cephaloleia adusta* Uhmann (Fig. 64)
–	Filas de puntuaciones elitrales completas; vértice de la cabeza con sulco medial; pro-, meso-, and metaesternos sin puntuaciones mediales, puntuados lateralmente punctate laterally; Costa Rica	*Cephaloleia kressi* García-Robledo, sp. n. (Fig. 272)
51(47)	Antenómero 1 transverso; México	*Cephaloleia chevrolatii* Baly (Fig. 96)
–	Antenómero 1 alargado o clavado	52
52(51)	Antenómero 1 clavado; élitros con húmeros sin puntuaciones	53
–	Antenómero 1 alargado; élitros con húmeros puntuados; Brasil, Costa Rica, Panamá	*Cephaloleia elegantula* Baly (Fig. 129)
53(51)	Antenómero 1 tan largo como 2 y 3 combinados; élitros con ángulo de la sutura sin diente; Costa Rica, Guatemala, Panamá	*Cephaloleia stenosoma* Baly (en parte) (Fig. 239)
–	Antenómero 1 tan largo como 3; élitros con ángulo de la sutura con diente diminuto; Bolivia, Colombia, Panamá, Perú, Venezuela	*Cephaloleia partita* Weise (Fig. 206)
54(42)	Dorso azul metálico, pronoto con márgenes laterales pálidas; Costa Rica, Guatemala, Nicaragua, Panamá	*Cephaloleia metallescens* Baly (Fig. 188)
–	Dorso negro o café rojizo	55
55(54)	Vértice de la cabeza cóncavo entre los ojos	56
–	Vértice de la cabeza aplanado entre los ojos	63
56(55)	Dorso negro; México	*Cephaloleia punctatissima* Weise (Fig. 218)
–	Dorso rojizo-café	57
57(56)	Sutura entre los esternos abdominales 1 y 2 completa	58
–	Sutura entre los esternos abdominales 1 y 2 obsoleta	59
58(57)	Especie más grande (más de 5.0 mm)	60
–	Especie más pequeña (menos de 4.0 mm); Dominica, Grenada	*Cephaloleia simplex* Staines (Fig. 236)
59(58)	Pronoto con disco puntuado; Colombia, Costa Rica, Panamá	*Cephaloleia distincta* Baly (Fig. 125)
–	Pronoto con disco sin puntuaciones; Colombia, Costa Rica, Panamá	*Cephaloleia placida* Baly (Fig. 210)
60(58)	Antenómero 1 subigual en longitud que 2; Costa Rica, Panamá	*Cephaloleia sulciceps* Baly (Fig. 246)
–	Antenómero 1 más largo que 2	61
61(60)	Antenómero 1 subigual en longitud que 2–3 combinados; vértice de la cabeza con carina medial; grande (8.0 mm); Costa Rica, Panamá	*Cephaloleia mauliki* Uhmann (Fig. 169)
–	Antenómero 1 no es subigual en longitud que 2–3 combinados; vértice de la cabeza sin carina medial; pequeño (<6.0 mm)	62
62(61)	Antenómero 1 subigual en longitud a 3; pronoto densamente puntuado; Trinidad	*Cephaloleia brunnea* Staines (Fig. 90)
–	Antenómero 1 no es subigual en longitud que 3; pronoto es escasamente puntuado; Trinidad	*Cephaloleia rubra* Staines (Fig. 225)
63(55)	Dorso negro; Costa Rica, El Salvador, Guatemala, México, Nicaragua, Panamá	*Cephaloleia tenella* Baly (Fig. 250)
–	Dorso café-rojizo	64
64(63)	Cuerpo alargado, redondeado	66
–	Cuerpo casi rectangular	65
65(64)	Elitros con fila de puntuaciones 6–9 obsoletas en el húmero; sutura entre los esternos abdominales 1 y 2 completas; Costa Rica, Guatemala	*Cephaloleia aequilata* Uhmann (Fig. 66)
–	Elitros con filas de puntuaciones 6–9 presentes en el húmero; sutura entre los esternos abdominales 1 y 2 obsoleta medialmente; Bolivia, Brasil, Colombia, Costa Rica, Ecuador, México, Nicaragua, Panamá, Perú, Venezuela	*Cephaloleia dilaticollis* Baly (Fig. 119)
66(65)	Sutura entre los esternos abdominales 1 y 2 completa	67
–	Sutura entre los esternos abdominales 1 y 2 obsoleta medialmente	70
67(66)	Antenómero 1–2 alargados; Costa Rica, Panamá	*Cephaloleia cylindrica* Staines (Fig. 110)
–	Antenómero 1–2 no subgloboso	68
68(66)	Antenómero 1 subigual a 2–4 combinados, clavado; Costa Rica, Guatemala, Panamá	*Cephaloleia antennalis* Donckier (Fig. 72)
–	Antenómero 1 más corto que 2–4 combinados, alargados	69
69(68)	Pronoto con disco puntuado; pro-, meso-, y metaesternos sin puntuaciones mediales, puntuados lateralmente; Costa Rica, Nicaragua, Panamá	*Cephaloleia puncticollis* Baly (Fig. 219)
–	Pronoto con disco sin puntuaciones; pro-, meso-, y metaesterno sin puntuaciones; Costa Rica, Guatemala, México, Panamá	*Cephaloleia sallei* Baly (Fig. 229)
70(66)	Elitros con líneas de puntuaciones confusas basalmente; Belice, Costa Rica, Guatemala, México	*Cephaloleia perplexa* Baly (Fig. 208)
–	Elitros con puntuaciones diferenciables basalmente	71
71(70)	Antenómero 1 al menos dos veces el largo de 2	72
–	Antenómero 1 menos de dos veces el largo de 2	74
72(71)	Elitros con sulco en el callo humeral; Brasil, Colombia, Costa Rica, Panamá, Perú	*Cephaloleia erichsonii* Baly (Fig. 132)
–	Elitros sin sulco en el callo humeral	73
73(72)	Antenómero 1 clavado, dos veces la longitud de 2; Puntuaciones elitrales obovadas, no son grandes en el disco; Costa Rica	*Cephaloleia conforma* García-Robledo & Staines, sp. n. (Fig. 102)
–	Antenómero 1 alargado, tres veces la longitud de 2; puntuaciones elitrales redondeadas, grandes en el disco; Costa Rica	*Cephaloleia calathae* García-Robledo & Staines, sp. n. (Fig. 93)
74(71)	Elitros con filas de puntuaciones confusas en el ápice; antenómero 1 más largo que 2; Bolivia, Brasil, Colombia, Costa Rica, Ecuador, Guayana Francesa, Guatemala, Honduras, México, Panamá, Perú, Venezuela	*Cephaloleia nigricornis* Fabricius (Fig. 193)
–	Elitros con filas de puntuaciones diferenciables apicalmente; antenómero 1 subigual a 2; México	*Cephaloleia delectabilis* Staines (Fig. 114)
75(41)	Elitros con mancha o línea	79
–	Elitros de un solo color	77
76(75)	Vértice de la cabeza comprimido entre los ojos; Bolivia, Brasíl, Colombia, Ecuador, Panamá, Perú, Venezuela	*Cephaloleia neglecta* Weise (Fig. 190)
–	Vértice de la cabeza aplanado entre los ojos	77
77(76)	Pronoto más oscuro que los élitros; antenómero 1 más largo que 2; Panamá	*Cephaloleia amblys* Staines (Image not available)
–	Pronoto más pálido que los élitros; antenómero 1 subigual en longitud que 2; Panamá	*Cephaloleia facetus* Staines (Fig. 136)
78(75)	Elitros con mancha	80
–	Elitros con líneas	84
79(78)	Vértice de la cabeza cóncavo entre los ojos	81
–	Vértice de la cabeza aplanado entre los ojos	82
80(79)	Color negro con mancha humeral rojiza; Costa Rica, Nicaragua, Panamá	*Cephaloleia uhmanni* Staines (Fig. 261)
–	Otro color	81
81(80)	Antenómero 1 más largo que 2; puntuaciones en el pronoto más densas lateralmente; puntuaciones elitrales en filas confusas apicalmente; Colombia, Costa Rica, Ecuador, Guayana Francesa, Panamá, Venezuela	*Cephaloleia trimaculata* Baly (Fig. 256)
–	Antenómero 1 subigual que 2; puntuaciones pronotales uniformemente distribuidas; filas de puntuaciones elitrales obsoletas apicalmente; Costa Rica, Panamá	*Cephaloleia weisei* Staines (Fig. 269)
82(80)	Pronoto con discos sin puntuaciones, puntuado lateralmente; antenómero 1 clavado, más largo que 2 y 3 combinados; Costa Rica, Guatemala, Panamá	*Cephaloleia stenosoma* Baly (en parte) (Fig. 239)
–	Pronoto puntuado; antenómero 1 alargado, no más largo que 2 y 3 combinados	83
83(82)	Antenómero 1 subigual en longitud a 2; pronoto con impresiones basales; Costa Rica, Nicaragua, Panamá	*Cephaloleia splendida* Staines (Fig. 237)
–	Antenómero 1 dos veces la longitud de 2; pronoto con impresiones laterales; Costa Rica, Panamá	*Cephaloleia turrialbana* Uhmann (Fig. 260)
84(79)	Vértice de la cabeza cóncavo entre los ojos	85
–	Vértice de la cabeza aplanado entre los ojos	91
85(84)	Húmero aproximadamente sin punturas	86
–	Húmero no es aproximadamente sin punturas	87
86(85)	Sutura entre los esternos abdominales 1 y 2 obsoletos medialmente; ángulo sutural de los élitros con un pequeño diente; Panamá	*Cephaloleia scitulus* Staines (Fig. 233)
–	Sutura entre los esternos abdominales 1 y 2 completa; ángulo sutural de los élitros sin un pequeño diente; México, Perú, Venezuela	*Cephaloleia parenthesis* Weise (Fig. 205)
87(85)	Puntuaciones de la fila de los élitros casi obsoletas apicalmente	88
–	Puntuaciones de la fila de los élitros diferenciables apicalmente	89
88(87)	Antenómero 1 engrosado, subigual en longitud a 2–4 combinados; Costa Rica, Nicaragua, Panamá	*Cephaloleia ornatrix* Donckier (Fig. 203)
–	Antenómero 1 alargado, más corto que 2–4 combinados; México	*Cephaloleia presignis* Staines (Fig. 213)
89(87)	Antenómero 1 subigual en longitud a 2–4 combinados; Costa Rica, México	*Cephaloleia separata* Baly (Fig. 235)
–	Antenómero 1 ⅓ la longitud de 2–4 combinados	90
90(89)	Elitros con fila adicional de puntuaciones luego de fila 7; ángulo sutural de los élitros con muescas; sutura entre los esternos abdominales 1 y 2 completa; Costa Rica, Panamá	*Cephaloleia apicata* Uhmann (Fig. 75)
–	Elitros sin fila adicional de puntuaciones luego de fila 7; ángulo sutural de los élitros redondeado; sutura entre los esternos abdominales 1 y 2 obsoleta medialmente; pronoto comprimido lateralmente; Costa Rica	*Cephaloleia disjuncta* Staines (Fig. 124)
91(84)	Elitros con ángulo sutural sin margen; Guatemala	*Cephaloleia lateralis* Baly (Fig. 172)
–	Elitros con ángulo sutural redondeado	92
92(91)	Elitros sin puntuaciones en los húmeros	94
–	Elitros con puntuaciones en los húmeros	93
93(92)	Antenómero 1 tan largo como 2–3 combinados; Guatemala, México Honduras, Panamá	*Cephaloleia discoidalis* Baly (Fig. 123)
–	Antenómero 1 más corto que 2–3 combinados; Costa Rica, Guatemala, Panamá	*Cephaloleia stenosoma* Baly (en parte) (Fig. 239)
94(92)	Elitros con filas de puntuaciones apicalmente obsoletas	95
–	Elitros con filas de puntuaciones apicalmente diferenciables	97
95(94)	Antenómero 1 subigual a 3; sutura entre esternos abdominales 1 y 2 obsoleta medialmente; Costa Rica, Nicaragua, Panamá	*Cephaloleia trivittata* Baly (Fig. 257)
–	Antenómero 1 tres veces la longitud de 3; sutura entre los esternos abdominales 1 y 2 completa	96
96(95)	Pronoto sin puntuaciones; pro-, meso y metaesternos puntuados lateralmente; Costa Rica	*Cephaloleia triangularis* Staines (Fig. 254)
–	Pronoto puntuado lateralmente; pro-, meso-, y metaesternos sin puntuaciones; Panamá	*Cephaloleia erugatus* Staines (Fig. 133)
97(94)	Antenómero 1 subigual en longitud a 2	98
–	Antenómero 1 mucho más largo que 2	99
98(97)	Antenómero 1 subigual a 3; 2 transverso; puntuaciones pronotales densas, uniformes; Costa Rica, Panamá	*Cephaloleia semivittata* Baly (Fig. 234)
–	Antenómero 1 subigual en longitud a 2; 2 alargado; pronoto irregularmente puntuado; Costa Rica	*Cephaloleia vittata* Staines (Imagen no disponible)
99(97)	Antenómero 1 tan largo como 2–4 combinados; pro-, meso-, y metaesternos sin puntuaciones medialmente, puntuados lateralmente	100
–	Antenómero 1 tan largo como 2–3 combinados; pro-, meso-, y metaesternos sin puntuaciones; Belice, Costa Rica, Guatemala, Panamá	*Cephaloleia consanguinea* Baly (Fig. 104)
100(99)	Angulo sutural de los élitros con pequeño diente; filas de puntuaciones diferenciables apicalmente; Costa Rica, Guatemala, Honduras, México, Nicaragua, Panamá	*Cephaloleia belti* Baly (Fig. 85)
–	Angulo sutural de los élitros sin diente pequeño; filas de puntuaciones convergen y se unen apicalmente; Colombia, Panamá	*Cephaloleia variabilis* Staines (Fig. 265)

### Key to the *Cephaloleia* known to occur in South America

**Table:** 

1	Elytra with exterior apical angle angulate	2
–	Elytra with exterior apical angle rounded	4
2(1)	Pronotum with lateral margin serrulate	3
–	Pronotum with lateral margin smooth; “Amazonas”	*Cephaloleia gracilis* Baly (Fig. 153)
3(2)	Pronotum with anterior angle angulate; suture between abdominal sterna 1 & 2 complete; Brazil	*Cephaloleia vagelineata* Pic (Fig. 264)
–	Pronotum with anterior angle rounded; suture between abdominal sterna 1 & 2 obsolete medially; Belize, Colombia, Panamá	*Cephaloleia formosus* Staines (Fig. 145)
4(1)	Dorsum unicolorous	5
–	Dorsum at least bicolorous	55
5(4)	Dorsum metallic blue or with metallic sheen	6
–	Dorsum black, brown or yellowish-brown	13
6(5)	Pronotum with distinct sulcus parallel to lateral margin; Colombia, Venezuela	*Cephaloleia aeneipennis* Baly (Fig. 65)
–	Pronotum without distinct sulcus parallel to lateral margin	7
7(6)	Lateral margin of pronotum serrulate; Colombia	*Cephaloleia tarsata* Baly (in part) (Fig. 249)
–	Lateral margin of pronotum smooth	8
8(7)	Lateral margin of elytra serrulate; Colombia, Costa Rica, Venezuela	*Cephaloleia cyanea* Staines (Fig. 109)
–	Lateral margin of elytra smooth	9
9(8)	Apical margin of elytra serrulate; Brazil, Ecuador	*Cephaloleia emarginata* Baly (Fig. 130)
–	Apical margin of elytra smooth	10
10(9)	Pronotum with transverse basal impression; Argentina, Brazil	*Cephaloleia nitida* Uhmann (Fig. 196)
–	Pronotum without transverse basal impression	11
11(10)	Antennomere 1 cylindrical	12
–	Antennomere 1 subglobose; Brazil	*Cephaloleia diplothemium* Uhmann (Fig. 122)
12(11)	Antennomere 1 shorter than 3; elytral punctures larger laterally; prosternum rugose laterally; Brazil, Ecuador	*Cephaloleia caeruleata* Baly (Fig. 92)
–	Antennomere 1 longer than 3; elytral punctures same size laterally; prosternum alutaceous laterally; Brazil	*Cephaloleia dilatata* Uhmann (Fig. 118)
13(5)	Dorsum black	14
–	Dorsum brown or yellowish-brown	27
14(13)	Vertex of head with medial fovea; Brazil	*Cephaloleia zikani* Uhmann (Fig. 271)
–	Vertex of head with medial carina or sulcus	15
15(14)	Lateral margin of pronotum serrulate	16
–	Lateral margin of pronotum not serrulate	19
16(14)	Apical margin of elytra smooth	17
–	Apical margin of elytra serrate; Brazil, Ecuador	*Cephaloleia elaeidis* Maulik (Fig. 128)
17(16)	Antennomere 2 obconic	18
–	Antennomere 2 cylindrical; French Guiana, Suriname	*Cephaloleia donckieri* Pic (Fig. 126)
18(17)	Elytral punctures in regular rows to apex; Brazil	*Cephaloleia depressa* Baly (Fig. 116)
–	Elytral puncture rows converging and uniting apically; Colombia	*Cephaloleia tarsata* Baly (in part) (Fig. 249)
19(15)	Lateral margin of pronotum canaliculate	20
–	Lateral margin of pronotum margined	24
20(19)	Pronotum with basal impression present	21
–	Pronotum with basal impression absent; Bolivia, Brazil	*Cephaloleia coroicoana* Uhmann (Fig. 107)
21(20)	Elytra with punctures nearly obsolete basally; Brazil	*Cephaloleia rufipes* Pic (Fig. 227)
–	Elytra with punctures distinct basally	22
22(21)	Elytra with punctures nearly obsolete apically; Brazil, Paraguay	*Cephaloleia fiebrigi* Uhmann (Fig. 140)
–	Elytral with punctures distinct apically	23
23(22)	Elytra with puncture rows distinct to apex; pronotum with disc impunctate; Brazil, Suriname	*Cephaloleia deplanata* Uhmann (Fig. 115)
–	Elytra with puncture rows converging and uniting apically; pronotum with disc punctate; Argentina, Bolivia, Brazil, Paraguay	*Cephaloleia marantae* Uhmann (Fig. 183)
24(19)	Vertex of head without medial sulcus or carina; Colombia, Ecuador, Venezuela	*Cephaloleia nigrithorax* Pic (Fig. 194)
–	Vertex of head with medial sulcus or carina	25
25(24)	Vertex of head with medial sulcus	26
–	Vertex of head with medial carina; Brazil	*Cephaloleia impressa* Baly (Fig. 164)
26(24)	Elytra with declivity beginning at puncture row 7; antennomere 1 longer than 3; Argentina, Bolivia, Brazil, Paraguay	*Cephaloleia funesta* Baly (Fig. 150)
–	Elytra without declivity beginning at puncture row 7; antennomere 1 shorter than 3; Brazil	*Cephaloleia obsoleta* Weise (Fig. 198)
27(13)	Lateral margin of pronotum with a lens-like swelling toward anterior angle; Ecuador, French Guiana, Peru, Suriname	*Cephaloleia lenticula* Staines, sp. n. (Fig. 174)
–	Lateral margin of pronotum without a lens-like swelling toward anterior angle	28
28(27)	Anterior angle of pronotum angulate	29
–	Anterior angle of pronotum rounded	30
29(27)	Vertex of head with medial sulcus; antennomeres 1 and 3 subequal in length; Brazil, Venezuela	*Cephaloleia cognata* Baly (Fig. 100)
–	Vertex of head with medial carina; antennomeres 1 and 2 subequal in length; Brazil, Colombia, Costa Rica, Ecuador, Mexico, Nicaragua, Panama, Peru, and Venezuela	*Cephaloleia dilaticollis* Baly (Fig. 119)
30(28)	Vertex of head with fovea; Brazil, Colombia, Ecuador, Peru, Venezuela	*Cephaloleia affinis* Baly (Fig. 67)
–	Vertex of head without fovea	31
31(30)	Pronotum with lateral margin canaliculate	32
–	Pronotum with lateral margin margined	48
32(30)	Vertex of head without sulcus or carina	33
–	Vertex of head with sulcus or carina	39
33(32)	Pronotum with transverse basal impression medially	34
–	Pronotum without transverse basal impression medially	35
34(33)	Elytra with punctures nearly obsolete at apex; Brazil, Peru	*Cephaloleia nigriceps* Baly (Fig. 192)
–	Elytra with punctures distinct at apex; Ecuador	*Cephaloleia lojaensis* Pic (Fig. 178)
35(33)	Elytral punctures confluent; Ecuador, Peru	*Cephaloleia chimboana* Uhmann (Fig. 98)
–	Elytral punctures distinct	36
36(35)	Elytral punctures nearly obsolete apically; Brazil, Colombia, Costa Rica, Panama, Peru	*Cephaloleia erichsonii* Baly (Fig. 132)
–	Elytral punctures distinct to apex	37
37(36)	Elytra with puncture rows converging and uniting apically; Bolivia	*Cephaloleia latipennis* Pic (Fig. 173)
–	Elytra with puncture rows distinct apically	38
38(37)	Antennomere 1 incrassate, longest; Colombia	*Cephaloleia polita* Weise (Fig. 211)
–	Antennomere 1 transverse, not longest; Brazil, Peru	*Cephaloleia dimidiaticornis* Baly (Fig. 121)
39(32)	Vertex of head with medial sulcus	40
–	Vertex of head with medial carina	44
40(39)	Pronotum with transverse basal impression; Bolivia, Brazil, Colombia, Ecuador, French Guiana, Peru	*Cephaloleia corallina* Erichson (Fig. 106)
–	Pronotum without transverse basal impression	41
41(40)	Antennae (except basal antennomere) black; Brazil, French Guiana, Peru	*Cephaloleia proxima* Baly (Fig. 216)
–	Antennal color different	42
42(41)	Meso- and metasterna impunctate	43
–	Meso- and metasterna punctate laterally; Brazil	*Cephaloleia apicicornis* Baly (Fig. 77)
43(42)	Vertex of head concave between eyes; antennomere 2 longer than 4; anterior angle of pronotum angulate; Brazil, Ecuador	*Cephaloleia halli* Uhmann (Fig. 156)
–	Vertex of head flat between eyes; antennomeres 2 and 4 subequal in length; anterior angle of pronotum rounded; Ecuador	*Cephaloleia ochra* Staines, sp. n. (Fig. 199)
44(42)	Pronotum without transverse basal impression	45
–	Pronotum with transverse basal impression	46
45(44)	Antennomere 1 longer than 2 and 3 combined; Colombia, Ecuador, Peru	*Cephaloleia unctula* Pic (Fig. 262)
–	Antennomere 1 subequal in length to 2; Brazil	*Cephaloleia subdepressa* Baly (Fig. 244)
46(44)	Antennomere 2 longer than 1; Brazil, Peru	*Cephaloleia interstitialis* Weise (Fig. 168)
–	Antennomere 1 longer than 2	47
47(46)	Antennomere 1 longer than 2 and 3 combined; Brazil, Ecuador	*Cephaloleia striata* Weise (Fig. 242)
–	Antennomere 1 shorter than 2 and 3 combined; Brazil	*Cephaloleia truncatipennis* Baly (Fig. 258)
48(31)	Pronotum with basal impression	49
–	Pronotum without basal impression	50
49(48)	Vertex of head with medial carina; elytra slightly costate apically; Brazil, Peru	*Cephaloleia approximata* Baly (Fig. 79)
–	Vertex of head without medial carina or sulcus; elytra not slightly costate apically; Brazil	*Cephaloleia bucki* Uhmann (Fig. 91)
50(48)	Vertex of head without sulcus or carina	51
–	Vertex of head with sulcus or carina	52
51(50)	Lateral margin of pronotum crenulate; vertex of head punctate; antennomeres 1 and 2 subequal in length; Ecuador	*Cephaloleia crenulata* Staines, sp. n. (Fig. 108)
–	Lateral margin of elytra smooth; vertex of head impunctate; antennomere 1 2× length of 2; Brazil, Colombia	*Cephaloleia steinhauseni* Uhmann (Fig. 238) (in part)
52(50)	Vertex of head with medial carina; Brazil, Peru, Venezuela	*Cephaloleia opaca* Baly (Fig. 200)
–	Vertex of head with medial sulcus	53
53(52)	Vertex of head punctate	54
–	Vertex of head impunctate; Bolivia, Brazil, Colombia, Costa Rica, Ecuador, French Guiana, Guatemala, Panama, Peru, Venezuela	*Cephaloleia nigricornis* (Fabricius) (Fig. 193)
54(53)	Elytral puncture rows regular to apex; antennomere 3 longer than 1; smaller species (5.0 mm); Brazil	*Cephaloleia castanea* Pic (Fig. 94)
–	Elytral puncture rows converging and uniting apically; antennomere 3 shorter than 1; larger species (7.6–9.6 mm); Colombia, Costa Rica, Panamá	*Cephaloleia distincta* Baly (Fig. 125)
55(4)	Elytra unicolorous	56
–	Elytra with more than one color	68
56(55)	Pronotum black	57
–	Pronotum reddish or yellowish	63
57(56)	Lateral margin of pronotum canaliculate	58
–	Lateral margin of pronotum margined	59
58(57)	Pronotum without transverse basal impression; antennomere 1 longer than 2; Bolivia, Brazil, Ecuador, Peru	*Cephaloleia clarkella* Baly (Fig. 99)
–	Pronotum with transverse basal impression; antennomere 1 subequal in length to 2; Brazil, Peru, Venezuela	*Cephaloleia luridipennis* (Weise) (Fig. 180)
59(57)	Vertex of head with medial sulcus	60
–	Vertex of head without medial sulcus	61
60(59)	Apical margin of elytra smooth; Bolivia, Brazil, Costa Rica, Panamá, Colombia, Ecuador, Peru, Venezuela	*Cephaloleia pretiosa* Baly (Fig. 214)
–	Apical margin of elytra finely serrulate; French Guiana	*Cephaloleia brevis* Staines, sp. n. (Fig. 89)
61(59)	Pronotum with transverse basal impression	62
–	Pronotum without transverse basal impression; Brazil, Colombia, Ecuador, Peru	*Cephaloleia flavipennis* Baly (Fig. 142)
62(61)	Lateral margin of pronotum canaliculate; Bolivia, Brazil, Colombia, Ecuador, Panama, Peru, Venezuela	*Cephaloleia neglecta* Weise (Fig. 190)
–	Lateral margin of pronotum margined; Colombia, Venezuela	*Cephaloleia collaris* Weise (Fig. 101)
63(56)	Lateral margin of pronotum finely serrulate	64
–	Lateral margin of pronotum smooth	65
64(63)	Pronotum immaculate, reddish; Peru	*Cephaloleia abdominalis* Pic (Fig. 63)
–	Pronotum with medial black longitudinal vitta; Brazil, Ecuador	*Cephaloleia susanae* Staines, sp. n. (Fig. 247)
65(63)	Vertex of head with fovea; Colombia, Ecuador, Peru	*Cephaloleia princeps* Baly (Fig. 215)
–	Vertex of head without fovea	66
66(63)	Vertex of head with medial sulcus	67
–	Vertex of head without medial sulcus; Brazil, Colombia	*Cephaloleia steinhauseni* Uhmann (Fig. 238) (in part)
67(65)	Antennomere 2 longer than 3; Brazil, Peru	*Cephaloleia amazona* Baly (Fig. 69)
–	Antennomere 3 longer than 2; Argentina, Brazil	*Cephaloleia teutonica* Uhmann (Fig. 252)
68(55)	Dorsum black with reddish macula at humeri	69
–	Dorsum colored differently	70
69(68)	Antennomere 2 obconic; pronotum with medial basal impression; Brazil, Peru	*Cephaloleia humeralis* Weise (Fig. 162)
–	Antennomere 2 subglobose; pronotum without medial basal impression; Argentina	*Cephaloleia tucumana* Weise (Fig. 259)
70(68)	Elytra with apical part darker than basal	71
–	Elytra with transverse or longitudinal vitta(e) or with macula(e) or fascia(e)	82
71(70)	Pronotum with medial longitudinal vitta; Ecuador	*Cephaloleia rosenbergi* Weise (Fig. 224)
–	Pronotum without medial longitudinal vitta	72
72(71)	Vertex of head with medial sulcus	73
–	Vertex of head without medial sulcus	76
73(72)	Pronotum with transverse basal impression	74
–	Pronotum without transverse basal impression	75
74(73)	Antennomere 1 longer than 3; Bolivia, Colombia, Peru, Venezuela	*Cephaloleia histrio* Guérin-Méneville (Fig. 158)
–	Antennomere 1 subequal in length to 3; Colombia, French Guiana	*Cephaloleia forestieri* Pic (Fig. 144)
75(73)	Antennomere 2 longer than 3; elytral puncture rows distinct to apex; Brazil	*Cephaloleia basalis* Pic (Fig. 83)
–	Antennomere 2 subequal in length to 3; elytral puncture rows converging and uniting apically; Brazil	*Cephaloleia waterhousei* Baly (Fig. 268)
76(72)	Pronotum with transverse basal impression; Bolivia, Brazil, Ecuador, Peru	*Cephaloleia grayei* Baly (Fig. 155)
–	Pronotum without transverse basal impression	77
77(76)	Head flat between eyes	78
–	Head concave between eyes	80
78(77)	Elytral puncture rows distinct along suture near apex; Colombia	*Cephaloleia apicalis* Baly (Fig. 74)
–	Elytral puncture rows obsolete along suture near apex; Argentina, Brazil, Colombia, Ecuador	*Cephaloleia fulvipes* Baly (Fig. 148)
80(77)	Lateral margin of pronotum straight	81
–	Lateral margin of pronotum sinuate; Brazil	*Cephaloleia apicenotata* Uhmann (Fig. 76)
81(80)	Antennomere 2 oval; elytral punctures obsolete apically; Bolivia, Brazil, Colombia, Peru, Venezuela	*Cephaloleia bicolor* Uhmann (Fig. 86)
–	Antennomere 2 elongate; elytral punctures not obsolete apically; Bolivia, Brazil, Colombia, Ecuador, Peru (?)	*Cephaloleia bicoloriceps* Pic (Fig. 87)
82(71)	Elytra with one or two transverse bands	83
–	Elytra without transverse bands	93
83(80)	Lateral margin of elytra with black markings extending to puncture row 6	84
–	Lateral margin of elytra not as above	86
84(83)	Lateral margin of pronotum canaliculate; Ecuador	*Cephaloleia bifasciata* Weise (Fig. 88)
–	Lateral margin of pronotum margined	85
85(84)	Vertex of head without medial sulcus; Ecuador	*Cephaloleia hnigrum* Pic (Fig. 160)
–	Vertex of head with medial sulcus; Colombia, Ecuador	*Cephaloleia recondita* Pic (Fig. 222)
86(83)	Vertex of head with medial fovea present; Ecuador	*Cephaloleia angustacollis* Staines, sp. n. (Fig. 71)
–	Vertex of head with medial fovea absent	87
87(86)	Vertex of head with medial sulcus absent	90
–	Vertex of head with medial sulcus	88
88(87)	Lateral margin of pronotum canaliculate	89
–	Lateral margin of pronotum margined; Colombia	*Cephaloleia succincta* Guérin-Méneville (Fig. 245)
89(88)	Antennomere 2 elongate; lateral margin of pronotum sinuate at base; larger species, > 8.0 mm; Ecuador, Peru	*Cephaloleia alternans* Waterhouse (Fig. 68)
–	Antennomere 2 subglobose; lateral margin of pronotum straight; smaller species, <4.0 mm; Ecuador	*Cephaloleia nana* Staines, sp. n. (Fig. 189)
90(87)	Antennomere 3 triangular	91
–	Antennomere 3 cylindrical	92
91(90)	Antennomeres 1 and 2 subequal in length; lateral margin of pronotum serrulate; Colombia, Ecuador, Peru	*Cephaloleia amba* Staines, sp. n. (Fig. 70)
–	Antennomere 1 2× length of 2; lateral margin of pronotum smooth; Ecuador, Peru	*Cephaloleia degandei* Baly (Fig. 113)
92(90)	Pro-, meso-, and metasterna punctate laterally; antennomeres 4–10 decreasing in length; Ecuador	*Cephaloleia applicata* Pic (Fig. 78)
–	Pro-, meso-, and metasterna impunctate; antennomeres 4–10 subequal in length; Brazil, Ecuador	*Cephaloleia nubila* Weise (Fig. 197)
93(82)	Elytra with longitudinal stripes or vittae	94
–	Elytra with spots or maculae	113
94(93)	Elytra with only sutural area darkened	95
–	Elytra different	98
95(94)	Pronotum totally black; Ecuador	*Cephaloleia balyi* Duvivier (Fig. 81)
–	Pronotum yellow with or without black markings	96
96(95)	Pronotum with black longitudinal vitta from base to apex; yellow; Ecuador	*Cephaloleia marshalli* Uhmann (Fig. 185)
–	Pronotum unicolorous	97
97(96)	Vertex of head with medial sulcus; lateral margin of pronotum straight; Brazil	*Cephaloleia fryella* Baly (Fig. 146)
–	Vertex of head with medial carina; lateral margin of pronotum sinuate; Brazil	*Cephaloleia lydiae* Uhmann (Fig. 181)
98(94)	Elytra predominately yellow with black vittae	99
–	Elytra not predominately yellow with black vittae	105
99(98)	Elytra with lateral margin all or partly darkened	100
–	Elytra with lateral margin not darkened	101
100(99)	Lateral margin of pronotum straight; smaller species (6.2 mm); Ecuador	*Cephaloleia felix* Waterhouse (Fig. 138)
–	Lateral margin of pronotum rounded, sinuate at base; larger species (9.0 mm); Colombia	*Cephaloleia whitei* Baly (Fig. 270)
101(99)	Pronotum without dark markings	102
–	Pronotum with dark markings	104
102(101)	Vertex of head without medial carina; antennomere 3 shorter than 1	103
–	Vertex of head with medial carina; antennomere 3 longer than 1; Brazil	*Cephaloleia abdita* Staines, sp. n. (Fig. 62)
103(102)	Antennomeres 1–2 paler; elytra with puncture rows 3–4 confused at base; Brazil	*Cephaloleia trilineata* Uhmann (Fig. 255)
–	Antennomeres all dark; elytra with puncture rows 3–4 not confused at base; Brazil	*Cephaloleia gemma* Staines, sp. n. (Fig. 151)
104(102)	Pronotum with medial longitudinal vitta; larger species (5.9–6.1 mm); Bolivia, Peru	*Cephaloleia convexifrons* Pic (Fig. 105)
–	Pronotum with triangular macula behind head; smaller species (<4.0 mm); Peru	*Cephaloleia chica* Staines, sp. n. (Fig. 97)
105(98)	Pronotum black with pale margins	106
–	Pronotum different	107
106(105)	Antennomeres 1 and 2 transverse; Brazil	*Cephaloleia flavovittata* Baly (Fig. 143)
–	Antennomere 1 elongate, 2 transverse; Bolivia, Brazil, Ecuador, French Guiana	*Cephaloleia deyrollei* Baly (Fig. 117)
107(105)	Pronotum unicolorous	108
–	Pronotum with black markings	109
108(107)	Vertex of head with medial fovea; pronotum without transverse basal impression; Argentina, Brazil	*Cephaloleia picta* Baly (Fig. 209)
–	Vertex of head with medial sulcus; pronotum with transverse basal impression; Argentina, Brazil, Peru, Venezuela	*Cephaloleia vittipennis* Weise (Fig. 267)
109(107)	Vertex of head without sulcus or carina; Colombia, Panama	*Cephaloleia variabilis* Staines (Fig. 265)
–	Vertex of head with sulcus or carina	110
110(109)	Vertex of head flat between eyes; Colombia, Panama	*Cephaloleia luctuosa* Guérin-Méneville (Fig. 179)
–	Vertex of head concave between eyes	111
111(110)	Suture between abdominal sterna 1 and 2 obsolete	112
–	Suture between abdominal sterna 1 and 2 complete; French Guiana, “Amazonas”	*Cephaloleia eximia* Baly (Fig. 135)
112(111)	Pro-, meso, and metasterna impunctate; Brazil, Ecuador, Peru	*Cephaloleia saundersii* Baly (Fig. 231)
–	Pro, meso-, and metasterna punctate; Peru	*Cephaloleia pulchella* Baly (Fig. 217)
113(93)	Elytra black with 2 yellowish humeral maculae and 2 apical maculae; Colombia, Ecuador, Peru	*Cephaloleia tetraspilota* Guérin-Méneville (Fig. 251)
–	Elytral markings different	114
114(113)	Pronotum different color than elytra	115
–	Pronotum same color as elytra	123
115(114)	Pronotum with transverse basal impression medially	120
–	Pronotum without transverse basal impression	116
116(115)	Vertex of head impunctate; Brazil	*Cephaloleia maculipennis* Baly (Fig. 182)
–	Vertex of head punctate	117
117(116)	Lateral margin of pronotum rounded	118
–	Lateral margin of pronotum straight	119
118(117)	Vertex of head flat between eyes; Brazil	*Cephaloleia emdeni* Uhmann (Fig. 131)
–	Vertex of head concave between eyes; Bolivia, Brazil	*Cephaloleia parvula* Weise (Fig. 207)
119(117)	Vertex of head with faint medial sulcus; “Amazonas”	*Cephaloleia thiemei* Weise (Fig. 253)
–	Vertex of head without medial sulcus; Colombia, Costa Rica, Ecuador, Panama, Venezuela	*Cephaloleia trimaculata* Baly (Fig. 256)
120(115)	Lateral margin of pronotum canaliculate	121
–	Lateral margin of pronotum margined	122
121(120)	Vertex of head concave between eyes, densely punctate; Bolivia, Brazil, Colombia, Ecuador, Peru, Venezuela	*Cephaloleia ornata* Waterhouse (Fig. 202)
–	Vertex of head flat between eyes, finely punctate; Colombia, Venezuela	*Cephaloleia fasciata* Weise (Fig. 137)
122(120)	Elytra with ovoid black medial macula on apical ½; pronotum red laterally; Peru	*Cephaloleia uniguttata* Pic (Fig. 263)
–	Elytra with black humeral and scutellar maculae basally and irregular transverse band from suture to lateral margin; pronotum black; Ecuador	*Cephaloleia insidiosa* Pic (Fig. 165)
123(114)	Pronotum with longitudinal black vitta	124
–	Pronotum without longitudinal black vitta	127
122(121)	Vertex of head with small tubercle; Colombia, Ecuador	*Cephaloleia daguana* Uhmann (Fig. 111)
–	Vertex of head without small tubercle	122
123(122)	Vertex of head impunctate; lateral margin of pronotum canaliculate; Bolivia, Ecuador, Panama, Peru	*Cephaloleia laeta* Waterhouse (Fig. 171)
–	Vertex of head irregularly punctate; lateral margin of pronotum margined	126
124(123)	Vertex of head with medial carina; pronotum without oblique impression laterally reaching to basal margin; smaller species (<5.0 mm); French Guiana	*Cephaloleia horvitzae* Staines, sp. n. (Fig. 161)
–	Vertex of head without medial carina; pronotum with oblique impression laterally reaching to basal margin; larger species (>6.0 mm); Brazil, Ecuador, Peru	*Cephaloleia antennata* Waterhouse (Fig. 73)
125(121)	Vertex of head with medial carina	126
–	Vertex of head without medial carina	129
126(125)	Pronotum with impression	127
–	Pronotum without impression; Ecuador, Peru	*Cephaloleia dilectans* Pic (Fig. 120)
127(125)	Pronotum with oblique impression on each side; Brazil	*Cephaloleia ornatula* Donckier (Fig. 204)
–	Pronotum without oblique impression on each side	128
128(127)	Lateral margins of pronotum straight, parallel; scutellum alutaceous; pronotum much narrower than base of elytra; Brazil	*Cephaloleia strandi* Uhmann (Fig. 241)
–	Lateral margins of pronotum straight, divergent; scutellum punctate; pronotum nearly as wide as the base of the elytra; Argentina	*Cephaloleia maxima* Uhmann (Fig. 187)
129(125)	Vertex of head with medial sulcus; Argentina, Brazil	*Cephaloleia linkei* Uhmann (Fig. 177)
–	Vertex of head without medial sulcus	130
130(129)	Lateral margin of pronotum sinuate at base, then rounded to apex; Argentina, Brazil	*Cephaloleia sagittifera* Uhmann (Fig. 228)
–	Lateral margin of pronotum straight at base	131
131(130)	Lateral margin of pronotum not divergent, canaliculate; Peru	*Cephaloleia quinquemaculata* Weise (Fig. 221)
–	Lateral margin of pronotum divergent, margined; Bolivia, Brazil, Colombia, Ecuador, Peru	*Cephaloleia kolbei* Weise (Fig. 170)

### Clave para las especies de *Cephaloleia* en Sur América

**Table:** 

1	Elitros con ápice exterior angulado	2
–	Elitros con ángulo externo apical redondeado	4
2(1)	Pronoto con márgenes laterales aserradas	3
–	Pronoto con márgenes laterales lisas; “Amazonas”	*Cephaloleia gracilis* Baly (Fig. 153)
3(2)	Pronoto con ángulo anterior angulado; sutura entre esternos abdominales 1 & 2 completos; Brasil	*Cephaloleia vagelineata* Pic (Fig. 264)
–	Pronoto con ángulo anterior redondeado; sutura entre esternos abdominales 1 & 2 medialmente obsoleta; Belice, Colombia, Panamá	*Cephaloleia formosus* Staines (Fig. 145)
4(1)	Dorso unicolor	5
–	Dorso como mínimo bicolor	55
5(4)	Dorso azul metálico o con brillo metálico	6
–	Dorso negro, amarillo o café-amarillento	13
6(5)	Pronoto con sulco evidente paralelo a las márgenes laterales; Colombia, Venezuela	*Cephaloleia aeneipennis* Baly (Fig. 65)
–	Pronoto sin sulco evidente paralelo a las márgenes laterales	7
7(6)	Márgenes laterales aserradas; Colombia	*Cephaloleia tarsata* Baly (en parte) (Fig. 249)
–	Márgenes laterales del pronoto lisas	8
8(7)	Márgenes laterales de los élitros aserradas; Colombia, Costa Rica, Venezuela	*Cephaloleia cyanea* Staines (Fig. 109)
–	Márgenes laterales de los élitros lisas	9
9(8)	Márgenes apicales de los élitros aserradas; Brasil, Ecuador	*Cephaloleia emarginata* Baly (Fig. 130)
–	Márgenes apicales de los élitros lisas	10
10(9)	Pronoto con impresiones basales transversales; Argentina, Brasil	*Cephaloleia nitida* Uhmann (Fig. 196)
–	Pronoto sin impresiones basales transversales	11
11(10)	Antenómero 1 cilíndrico	12
–	Antenómero 1 subgloboso; Brasil	*Cephaloleia diplothemium* Uhmann (Fig. 122)
12(11)	Antenómero 1 más corto que 3; puntuaciones de los élitros más grandes lateralmente; proesterno rugoso lateralmente; Brasil, Ecuador	*Cephaloleia caeruleata* Baly (Fig. 92)
–	Antenómero 1 más largo que 3; puntuaciones de los élitros de igual tamaño lateralmente; proesterno alutaceo lateralmente; Brasil	*Cephaloleia dilatata* Uhmann (Fig. 118)
13(5)	Dorso negro	14
–	Dorso café o café-amarillento	27
14(13)	Vértice de la cabeza con fovea medial; Brasil	*Cephaloleia zikani* Uhmann (Fig. 271)
–	Vértice de la cabeza con carina o sulco medial	15
15(14)	Márgenes laterales del pronoto aserradas	16
–	Márgenes laterales del pronoto no aserradas	19
16(14)	Margen apical de los élitros lisa	17
–	Margen apical de los élitros aserrada; Brasil, Ecuador	*Cephaloleia elaeidis* Maulik (Fig. 128)
17(16)	Antenómero 2 obocónico	18
–	Antenómero 2 cilíndrico; Guayana Francesa, Surinam	*Cephaloleia donckieri* Pic (Fig. 126)
18(17)	Puntuaciones elitrales en filas regulares hacia el ápice; Brasil	*Cephaloleia depressa* Baly (Fig. 116)
–	Puntuaciones elitrales en filas que convergen y se unen apicálmente; Colombia	*Cephaloleia tarsata* Baly (Fig. 249) (En parte)
19(15)	Márgenes laterales del pronoto caniculadas	20
–	Márgenes laterales del pronoto con márgen	24
20(19)	Pronoto con impresiones basales	21
–	Pronoto sin impresiones basales; Bolivia, Brasil	*Cephaloleia coroicoana* Uhmann (Fig. 107)
21(20)	Elitros con puntuaciones basales casi obsoletas; Brasil	*Cephaloleia rufipes* Pic (Fig. 227)
–	Elitros con puntuaciones basalmente diferenciables	22
22(21)	Elitros con puntuaciones apicales basales casi obsoletas; Brasil, Paraguay	*Cephaloleia fiebrigi* Uhmann (Fig. 140)
–	Elitros con puntuaciones apicales diferenciables	23
23(22)	Elitros con filas de puntuaciones diferenciables en el ápice; pronoto con disco sin puntuaciones; Brasil, Surinam	*Cephaloleia deplanata* Uhmann (Fig. 115)
–	Elitros con filas de puntuaciones convergentes y unidas apicalmente; pronoto con disco puntuado; Argentina, Bolivia, Brasil, Paraguay	*Cephaloleia marantae* Uhmann (Fig. 183)
24(19)	Vértice de la cabeza sin sulco medial o carina; Colombia, Ecuador, Venezuela	*Cephaloleia nigrithorax* Pic (Fig. 194)
–	Vértice de la cabeza con sulco medial o carina	25
25(24)	Vértice de la cabeza con sulco medial	26
–	Vértice de la cabeza con carina medial; Brasil	*Cephaloleia impressa* Baly (Fig. 164)
26(24)	Elitros con declive iniciándose en la fila de puntuaciones 7; antenómero 1 más largo que 3; Argentina, Bolivia, Brasil, Paraguay	*Cephaloleia funesta* Baly (Fig. 150)
–	Elitros sin declive iniciándose en la fila de puntuaciones; antenómero 1 más corto que 3; Brasil	*Cephaloleia obsoleta* Weise (Fig. 198)
27(13)	Márgenes laterales del pronoto con un engrosamiento similar a un lente hacia el ángulo anterior; Ecuador, Guayana Francesa, Perú, Surinam	*Cephaloleia lenticula* Staines, sp. n. (Fig. 174)
–	Márgenes laterales del pronoto sin engrosamiento similar a un lente hacia el ángulo anterior	28
28(27)	Angulo anterior del pronoto angulado	29
–	Angulo anterior del pronoto redondeado	30
29(27)	Vértice de la cabeza con sulco medial; antenómero 1 y 3 subiguales en longitud; Brasil, Venezuela	*Cephaloleia cognata* Baly (Fig. 100)
–	Vértice de la cabeza con carina medial; antenómero 1 y 2 subiguales en longitud; Brasil, Colombia, Costa Rica, Ecuador, México, Nicaragua, Panamá, Perú, and Venezuela	*Cephaloleia dilaticollis* Baly (Fig. 119)
30(28)	Vértice de la cabeza con fovea; Brasil, Colombia, Ecuador, Perú, Venezuela	*Cephaloleia affinis* Baly (Fig. 67)
–	Vértice de la cabeza sin fovea	31
31(30)	Pronoto con márgenes laterales caniculadas	32
–	Pronoto con márgenes laterales	48
32(30)	Vértice de la cabeza sin sulco o carina	33
–	Vértice de la cabeza con sulco o carina	39
33(32)	Pronoto con impresiones basales mediales transversales	34
–	Pronoto sin impresiones basales mediales transversales	35
34(33)	Elitros con puntuaciones casi obsoletas en el ápice; Brasil, Perú	*Cephaloleia nigriceps* Baly (Fig. 192)
–	Elitros con puntuaciones diferenciables en el ápice; Ecuador	*Cephaloleia lojaensis* Pic (Fig. 178)
35(33)	Puntuaciones en los élitros confluentes; Ecuador, Perú	*Cephaloleia chimboana* Uhmann (Fig. 98)
–	Puntuaciones en los élitros diferenciables	36
36(35)	Puntuaciones elitrales casi obsoletas en el ápice; Brasil, Colombia, Costa Rica, Panamá, Perú	*Cephaloleia erichsonii* Baly (Fig. 132)
–	Puntuaciones elitrales diferenciables en el ápice	37
37(36)	Elitros con filas de puntuaciones convergiendo y uniéndose apicálmente; Bolivia	*Cephaloleia latipennis* Pic (Fig. 173)
–	Elitros con filas de puntuaciones diferenciables apicálmente	38
38(37)	Antenómero 1 engrosado y es el más largo; Colombia	*Cephaloleia polita* Weise (Fig. 211)
–	Antenómero 1 no es el más largo; Brasil, Perú	*Cephaloleia dimidiaticornis* Baly (Fig. 121)
39(32)	Vértice de la cabeza con sulco medial	40
–	Vértice de la cabeza con carina medial	44
40(39)	Pronoto con impresiones basales transversales; Bolivia, Brasil, Colombia, Ecuador, Guayana Francesa, Perú	*Cephaloleia corallina* Erichson (Fig. 106)
–	Pronoto sin impresiones basales transversales	41
41(40)	Antenas (excepto el antenómero basal) negras; Brasil, Guayana Francesa, Perú	*Cephaloleia proxima* Baly (Fig. 216)
–	Color de las antenas diferente	42
42(41)	Meso- y metaesternos sin puntuaciones	43
–	Meso- y metaesternos puntuados lateralmente; Brasil	*Cephaloleia apicicornis* Baly (Fig. 77)
43(42)	Vértice de la cabeza cóncavo; antenómero 2 más largo que 4; ángulo anterior del pronoto angulado; Brasil, Ecuador	*Cephaloleia halli* Uhmann (Fig. 156)
–	Vértice de la cabeza aplanado entre los ojos; antenómeros 2 y 4 subiguales en longitud; ángulo anterior del pronoto redondeado; Ecuador	*Cephaloleia ochra* Staines, sp. n. (Fig. 199)
44(42)	Pronoto sin impresiones basales transversales	45
–	Pronoto con impresiones basales transversales	46
45(44)	Antenómero 1 más largo que 2 y 3 combinados; Colombia, Ecuador, Perú	*Cephaloleia unctula* Pic (Fig. 262)
–	Antenómero 1 subigual en longitud a 2; Brasil	*Cephaloleia subdepressa* Baly (Fig. 244)
46(44)	Antenómero 2 más largo que 1; Brasil, Perú	*Cephaloleia interstitialis* Weise (Fig. 168)
–	Antenómero 1 más largo que 2	47
47(46)	Antenómero 1 más largo que 2 y 3 combinados; Brasil, Ecuador	*Cephaloleia striata* Weise (Fig. 242)
–	Antenómero 1 más corto que 2 y 3 combinados; Brasil	*Cephaloleia truncatipennis* Baly (Fig. 258)
48(31)	Pronoto con impresiones basales	49
–	Pronoto sin impresiones basales	50
49(48)	Vértice de la cabeza con carina medial; élitros levemente costados apicalmente; Brasil, Perú	*Cephaloleia approximata* Baly (Fig. 79)
–	Vértice de la cabeza sin carina medial o sulco; élitros no son levemente costados apicalmente; Brasil	*Cephaloleia bucki* Uhmann (Fig. 91)
50(48)	Vértice de la cabeza sin sulco o carina	51
–	Vértice de la cabeza con sulco o carina	52
51(50)	Márgenes laterales del pronoto crenuladas; vértice de la cabeza puntuado; antenómeros 1 y 2 subiguales en longitud; Ecuador	*Cephaloleia crenulata* Staines, sp. n. (Fig. 108)
–	Márgenes laterales lisas; vértice de la cabeza sin puntuaciones; antenómero 1 2× el largo de 2; Brasil, Colombia	*Cephaloleia steinhauseni* Uhmann (Fig. 238) (en parte)
52(50)	Vértice de la cabeza con carina medial; Brasil, Perú, Venezuela	*Cephaloleia opaca* Baly (Fig. 200)
–	Vértice de la cabeza con sulco medial	53
53(52)	Vértice de la cabeza puntuados	54
–	Vértice de la cabeza no puntuada; Bolivia, Brasil, Colombia, Costa Rica, Ecuador, Guayana Francesa, Guatemala, Panamá, Perú, Venezuela	*Cephaloleia nigricornis* (Fabricius) (Fig. 193)
54(53)	Puntuaciones elitrales en líneas regulares hacia el ápice; antenómero 3 más largo que 1; Especie más pequeña (5.0 mm); Brasil	*Cephaloleia castanea* Pic (Fig. 94)
–	Puntuaciones elitrales en líneas que convergen y se unen apicálmente; antenómero 3 más corto que 1; especie más grande (7.6–9.6 mm); Colombia, Costa Rica, Panamá	*Cephaloleia distincta* Baly (Fig. 125)
55(4)	Elitros unicoloros	56
–	Elitros con más de un color	68
56(55)	Pronoto negro	57
–	Pronoto rojizo o amarillento	63
57(56)	Márgenes laterales del pronoto caniculadas	58
–	Márgenes laterales del pronoto con margen evidente	59
58(57)	Pronoto sin impresiones basales transversales; antenómero 1 más largo que 2; Bolivia, Brasil, Ecuador, Perú	*Cephaloleia clarkella* Baly (Fig. 99)
–	Pronoto con impresión basal transversal; antenómero 1 subigual en longitud a 2; Brasil, Perú, Venezuela	*Cephaloleia luridipennis* (Weise) (Fig. 180)
59(57)	Vértice de la cabeza con sulco medial	60
–	Vértice de la cabeza sin sulco medial	61
60(59)	Márgenes apicales de los élitros lisas; Bolivia, Brasil, Costa Rica, Panamá, Colombia, Ecuador, Perú, Venezuela	*Cephaloleia pretiosa* Baly (Fig. 214)
–	Márgenes apicales de los élitros finamente aserradas; Guayana Francesa	*Cephaloleia brevis* Staines, sp. n. (Fig. 89)
61(59)	Pronoto con impresión basal transversal	62
–	Pronoto sin impresión basal transversal; Brasil, Colombia, Ecuador, Perú	*Cephaloleia flavipennis* Baly (Fig. 142)
62(61)	Márgenes laterales del pronoto caniculadas; Bolivia, Brasil, Colombia, Ecuador, Panamá, Perú, Venezuela	*Cephaloleia neglecta* Weise (Fig. 190)
–	Margen lateral del pronoto con margen evidente; Colombia, Venezuela	*Cephaloleia collaris* Weise (Fig. 101)
63(56)	Márgenes laterales del pronoto finamente aserradas	64
–	Márgenes laterales del pronoto lisas	65
64(63)	Pronoto sin mancha, rojizo; Perú	*Cephaloleia abdominalis* Pic (Fig. 63)
–	Pronoto con línea medial negra longitudinal; Brasil, Ecuador	*Cephaloleia susanae* Staines, sp. n. (Fig. 247)
65(63)	Vértice de la cabeza con fovea; Colombia, Ecuador, Perú	*Cephaloleia princeps* Baly (Fig. 215)
–	Vértice de la cabeza sin fovea	66
66(63)	Vértice de la cabeza con sulco medial	67
–	Vértice de la cabeza sin sulco medial; Brasil, Colombia	*Cephaloleia steinhauseni* Uhmann (en parte) (Fig. 238)
67(65)	Antenómero 2 más largo que 3; Brasil, Perú	*Cephaloleia amazona* Baly (Fig. 69)
–	Antenómero 3 más largo que 2; Argentina, Brasil	*Cephaloleia teutonica* Uhmann (Fig. 252)
68(55)	Dorso negro con mancha rojiza en los húmeros	69
–	Dorso de color diferente	70
69(68)	Antenómero 2 obcónico; pronoto con impresión basal medial; Brasil, Perú	*Cephaloleia humeralis* Weise (Fig. 162)
–	Antenómero 2 subgloboso; pronoto sin impresión medial basal; Argentina	*Cephaloleia tucumana* Weise (Fig. 259)
70(68)	Elitros con parte apical más oscura que la parte basal	71
–	Elitros con linea(s) transversal o longitudinal o con mancha(s) o banda(s)	82
71(70)	Pronoto con línea medial longitudinal; Ecuador	*Cephaloleia rosenbergi* Weise (Fig. 224)
–	Pronoto sin línea medial longitudinal	72
72(71)	Vértice de la cabeza con sulco medial	73
–	Vértice de la cabeza sin sulco medial	76
73(72)	Pronoto con impresión basal transversal	74
–	Pronoto sin impresión basal transversal	75
74(73)	Antenómero 1 más largo que 3; Bolivia, Colombia, Perú, Venezuela	*Cephaloleia histrio* Guérin-Méneville (Fig. 158)
–	Antenómero 1 subigual en longitud a 3; Colombia, Guayana Francesa	*Cephaloleia forestieri* Pic (Fig. 144)
75(73)	Antenómero 2 más largo que 3; puntuaciones en los élitros en filas diferenciables en el ápice; Brasil	basalis Pic (Fig. 83)
–	Antenómero 2 subigual en longitud a 3; puntuaciones elitrales en filas que convergen y se unen apicalmente; Brasil	*Cephaloleia waterhousei* Baly (Fig. 268)
76(72)	Pronoto con impresión basal transversal; Bolivia, Brasil, Ecuador, Perú	*Cephaloleia grayei* Baly (Fig. 155)
–	Pronoto sin impresión basal transversal	77
77(76)	Cabeza aplanada entre los ojos	78
–	Cabeza cóncava entre los ojos	80
78(77)	Puntuaciones elitrales en filas que se diferencian a lo largo de la sutura y cerca al ápice; Colombia	*Cephaloleia apicalis* Baly (Fig. 74)
–	Puntuaciones elitrales en filas obsoletas a lo largo de la sutura y cerca al ápice; Argentina, Brasil, Colombia, Ecuador	*Cephaloleia fulvipes* Baly (Fig. 148)
80(77)	Márgenes laterales del pronoto rectas	81
–	Márgenes laterales del pronoto sinuosas; Brasil	*Cephaloleia apicenotata* Uhmann (Fig. 76)
81(80)	Antenómero 2 ovalado; puntuaciones elitrales obsoletas apicalmente; Bolivia, Brasil, Colombia, Perú, Venezuela	*Cephaloleia bicolor* Uhmann (Fig. 86)
–	Antenómero 2 alargado; puntuaciones elitrales no son obsoletas apicalmente; Bolivia, Brasil, Colombia, Ecuador, Perú (?)	*Cephaloleia bicoloriceps* Pic (Fig. 87)
82(71)	Elitros con una o dos bandas transversales	83
–	Elitros sin bandas transversales	93
83(80)	Margenes laterales de los élitros con marcas negras extendiéndose hasta la fila de puntuaciones 6	84
–	Margen lateral de los élitros no es como descrita anteriormente	86
84(83)	Margen lateral del pronoto caniculada; Ecuador	*Cephaloleia bifasciata* Weise (Fig. 88)
–	Margen lateral del pronoto con margen evidente	85
85(84)	Vértice de la cabeza sin sulco medial; Ecuador	*Cephaloleia hnigrum* Pic (Fig. 160)
–	Vértice de la cabeza con sulco medial; Colombia, Ecuador	*Cephaloleia recondita* Pic (Fig. 222)
86(83)	Vértice de la cabeza con fovea medial; Ecuador	*Cephaloleia angustacollis* Staines, sp. n. (Fig. 71)
–	Vértice de la cabeza sin fovea medial	87
87(86)	Vértice de la cabeza sin sulco medial	90
–	Vértice de la cabeza con sulco medial	88
88(87)	Margen lateral del pronoto caniculada	89
–	Margen lateral del pronoto con margen evidente; Colombia	*Cephaloleia succincta* Guérin-Méneville (Fig. 245)
89(88)	Antenómero 2 alargado; margen lateral del pronoto sinuada en la base; especie grande, > 8.0 mm; Ecuador, Perú	*Cephaloleia alternans* Waterhouse (Fig. 68)
–	Antenómero 2 subgloboso; margen lateral del pronoto recta; especie más pequeña, <4.0 mm; Ecuador	*Cephaloleia nana* Staines, sp. n. (Fig. 189)
90(87)	Antenómero 3 triangular	91
–	Antenómero 3 cilíndrico	92
91(90)	Antenómero 1 y 2 subiguales en longitud; márgenes laterales del pronoto aserrados; Colombia, Ecuador, Perú	*Cephaloleia amba* Staines, sp. n. (Fig. 70)
–	Antenómero 1 dos veces el largo de 2; márgenes laterales del pronoto lisas; Ecuador, Perú	*Cephaloleia degandei* Baly (Fig. 113)
92(90)	Pro-, meso-, y metaesternos puntuados laterálmente; antenómero 4–10 decrece en longitud; Ecuador	*Cephaloleia applicata* Pic (Fig. 78)
–	Pro-, meso-, y metaesternos sin puntuaciones; antenómeros 4–10 subiguales en longitud; Brasil, Ecuador	*Cephaloleia nubila* Weise (Fig. 197)
93(82)	Elitros con barras o líneas	94
–	Elitros con lunares o manchas	113
94(93)	Elitros con solo el área sutural oscura	95
–	Elitros diferentes a lo descrito anteriormente	98
95(94)	Pronoto totalmente negro; Ecuador	*Cephaloleia balyi* Duvivier (Fig. 81)
–	Pronoto amarillo con o sin marcas negras	96
96(95)	Pronoto con líneas longitudinales desde la base al ápice; amarillo; Ecuador	*Cephaloleia marshalli* Uhmann (Fig. 185)
–	Pronoto unicolor	97
97(96)	Vértice de la cabeza con sulco medial; margen lateral del pronoto recto; Brasil	*Cephaloleia fryella* Baly (Fig. 146)
–	Vértice de la cabeza con carina medial; margen lateral del pronoto sinuoso; Brasil	*Cephaloleia lydiae* Uhmann (Fig. 181)
98(94)	Elitros predominantemente amarillos con líneas negras	99
–	Elitros no son predominantemente amarillos ni tienen líneas negras	105
99(98)	Elitros con márgenes laterales total o parcialmente oscuros	100
–	Elitros con márgenes laterales no oscuros	101
100(99)	Margen lateral del pronoto recto; especie más pequeña (6.2 mm); Ecuador	*Cephaloleia felix* Waterhouse (Fig. 138)
–	Margen lateral del pronoto redondeado, sinuoso en la base; especie más grande (9.0 mm); Colombia	*Cephaloleia whitei* Baly (Fig. 270)
101(99)	Pronoto sin marcas oscuras	102
–	Pronoto con marcas oscuras	104
102(101)	Vértice de la cabeza sin carina medial; antenómero 3 más corto que 1	103
–	Vértice de la cabeza con carina medial; antenómero 3 más largo que 1; Brasil	*Cephaloleia abdita* Staines, sp. n. (Fig. 62)
103(102)	Antenómero 1–2 pálidos; élitros con puntuaciones en las filas 3–4 confusas en la base; Brasil	*Cephaloleia trilineata* Uhmann (Fig. 255)
–	Todos los antenómeros oscuros; élitros con filas de puntuaciones 3–4 no confusas en la base; Brasil	gemma Staines, sp. n. (Fig. 151)
104(102)	Pronoto con línea medial longitudinal; especie más grande (5.9–6.1 mm); Bolivia, Perú	*Cephaloleia convexifrons* Pic (Fig. 105)
–	Pronoto con mácula triangular detrás de la cabeza; especie más pequeña (<4.0 mm); Perú	*Cephaloleia chica* Staines, sp. n. (Fig. 97)
105(98)	Pronoto negro con márgenes pálidas	106
–	Pronoto diferente	107
106(105)	Antenómeros 1 y 2 transversos; Brasil	*Cephaloleia flavovittata* Baly (Fig. 143)
–	Antenómeros 1 alargado, 2 transverso; Bolivia, Brasil, Ecuador, Guayana Francesa	*Cephaloleia deyrollei* Baly (Fig. 117)
107(105)	Pronoto unicolor	108
–	Pronoto con marcas negras	109
108(107)	Vértice de la cabeza con fovea medial; pronoto sin impresión basal transversal; Argentina, Brasil	*Cephaloleia picta* Baly (Fig. 209)
–	Vértice de la cabeza con sulco medial; pronoto con impresión basal transversal; Argentina, Brasil, Perú, Venezuela	*Cephaloleia vittipennis* Weise (Fig. 267)
109(107)	Vértice de la cabeza sin sulco o carina; Colombia, Panamá	*Cephaloleia variabilis* Staines (Fig. 265)
–	Vértice de la cabeza con sulco o carina	110
110(109)	Vértice de la cabeza aplanado entre los ojos; Colombia, Panamá	*Cephaloleia luctuosa* Guérin-Méneville (Fig. 179)
–	Vértice de la cabeza cóncavo entre los ojos	111
111(110)	Sutura entre los esternitos abdominales 1 y 2 obsoletos	112
–	Sutura entre los esternitos abdominales 1 y 2 completas; Guayana Francesa, “Amazonas”	*Cephaloleia eximia* Baly (Fig. 135)
112(111)	Pro-, meso, y metaesternos sin puntuaciones; Brasil, Ecuador, Perú	*Cephaloleia saundersii* Baly (Fig. 231)
–	Pro, meso-, y metasternos con puntuaciones; Perú	*Cephaloleia pulchella* Baly (Fig. 217)
113(93)	Elitros negros con dos manchas humerales amarillentas y dos manchas apicales; Colombia, Ecuador, Perú	*Cephaloleia tetraspilota* Guérin-Méneville (Fig. 251)
–	Marcas en los élitros diferentes a las descritas anteriormente	114
114(113)	Pronoto de diferente color que los élitros	115
–	Pronoto del mismo color que los élitros	123
115(114)	Pronoto con impresiones basales mediales transversales	120
–	Pronoto sin impresiones basales transversales	116
116(115)	Vértice de la cabeza sin puntuaciones; Brasil	*Cephaloleia maculipennis* Baly (Fig. 182)
–	Vértice de la cabeza puntuado	117
117(116)	Márgenes laterales del pronoto redondeadas	118
–	Márgenes laterales del pronoto rectas	119
118(117)	Vértice de la cabeza aplanado entre los ojos; Brasil	*Cephaloleia emdeni* Uhmann (Fig. 131)
–	Vértice de la cabeza cóncavo entre los ojos; Bolivia, Brasil	*Cephaloleia parvula* Weise (Fig. 207)
119(117)	Vértice de la cabeza con un sulco medial débil; “Amazonas”	*Cephaloleia thiemei* Weise (Fig. 253)
–	Vértice de la cabeza sin sulco medial; Colombia, Costa Rica, Ecuador, Panamá, Venezuela	*Cephaloleia trimaculata* Baly (Fig. 256)
120(115)	Márgenes laterales del pronoto caniculadas	121
–	Márgenes laterales del pronoto con margenes evidentes	122
121(120)	Vértice de la cabeza cóncavo entre los ojos, densamente puntuado; Bolivia, Brasil, Colombia, Ecuador, Perú, Venezuela	*Cephaloleia ornata* Waterhouse (Fig. 202)
–	Vértice de la cabeza aplanado entre los ojos, finamente puntuado; Colombia, Venezuela	*Cephaloleia fasciata* Weise (Fig. 137)
122(120)	Elitros con mancha medial ovoide en la zona apical ½; del pronoto rojos lateralmente; Perú	*Cephaloleia uniguttata* Pic (Fig. 263)
–	Elitros con manchas humerales y escutelares negras basalmente y con bandas irregulares transversales desde la sutura hasta las márgenes laterales; pronoto negro; Ecuador	*Cephaloleia insidiosa* Pic (Fig. 165)
123(114)	Pronoto con línea negra longitudinal	124
–	Pronoto sin línea longitudinal negra	127
122(121)	Vértice de la cabeza con tubérculo pequeño; Colombia, Ecuador	*Cephaloleia daguana* Uhmann (Fig. 111)
–	Vértice de la cabeza sin tubérculo pequeño	122
123(122)	Vértice de la cabeza sin puntuaciones; márgenes laterales del pronoto caniculadas; Bolivia, Ecuador, Panamá, Perú	*Cephaloleia laeta* Waterhouse (Fig. 171)
–	Vértice de la cabeza irregularmente puntuada; márgenes laterales del pronoto con margenes evidentes	126
124(123)	Vértice de la cabeza con carina medial; pronoto sin impresión oblicua lateralmente alcanzando hasta la margen basal; especie más pequeña (<5.0 mm); Guayana Francesa	*Cephaloleia horvitzae* Staines, sp. n. (Fig. 161)
–	Vértice de la cabeza sin carina medial; pronoto con una impresión oblícua lateralmente alcanzando la margen basal; especie más grande (>6.0 mm); Brasil, Ecuador, Perú	*Cephaloleia antennata* Waterhouse (Fig. 73)
125(121)	Vértice de la cabeza con carina medial	126
–	Vértice de la cabeza sin carina medial	129
126(125)	Pronoto con impresiones	127
–	Pronoto sin impresiones; Ecuador, Perú	*Cephaloleia dilectans* Pic (Fig. 120)
127(125)	Pronoto con impresión oblicua en cada lado; Brasil	*Cephaloleia ornatula* Donckier (Fig. 204)
–	Pronoto sin impresión oblicua en cada flanco	128
128(127)	Márgenes laterales del pronoto rectas, paralelas; escutelo con apariencia coriacea y color café-amarillento; pronoto mucho más angosto que la base de los élitros; Brasil	*Cephaloleia strandi* Uhmann (Fig. 241)
–	Márgenes laterales del pronoto rectas, divergentes; escutelo puntuado; pronoto casi tan ancho como la base de los élitros; Argentina	*Cephaloleia maxima* Uhmann (Fig. 187)
129(125)	Vértice de la cabeza con sulco medial; Argentina, Brasil	*Cephaloleia linkei* Uhmann (Fig. 177)
–	Vértice de la cabeza sin sulco medial	130
130(129)	Margen lateral del pronoto sinuosa en la base, luego redondeada en el ápice; Argentina, Brasil	*Cephaloleia sagittifera* Uhmann (Fig. 228)
–	Margen lateral del pronoto rectas en la base	131
131(130)	Márgenes laterales del pronoto no divergentes, caniculadas; Perú	*Cephaloleia quinquemaculata* Weise (Fig. 221)
–	Márgenes laterales del pronoto divergentes, no caniculadas; Bolivia, Brasil, Colombia, Ecuador, Perú	*Cephaloleia kolbei* Weise (Fig. 170)

### Accounts of the known species of *Cephaloleia*

#### Cephaloleia abdita

Taxon classificationAnimaliaColeopteraChrysomelidae

Staines sp. n.

http://zoobank.org/F070557F-6C17-48D7-A6CF-57E22A07145F

http://species-id.net/wiki/Cephaloleia_abdita

Fig. 62

##### Description.

Elongate; subdepressed; yellowish; antennomeres 7-11 darker; elytra with black vitta of variable width from humerus along edges of lateral and apical margins, suture dark. Head: vertex finely, densely punctate, very faint medial carina present; frons not projecting; not depressed between eyes. Antenna: reaches to humerus; antennomeres uniform in thickness; 3 longest; 1–2, 4–5 subequal in length; 6–10 subequal in length, each shorter than 5; 11 2× length of 10, pointed at apex; 1–4 punctate with scattered setae; 5–11 setose. Pronotum: transverse; lateral margin straight then rounding to anterior angle, slightly margined; anterior angle rounded, not produced; posterior angle acute; anterior margin curved anteriorly; disc subconvex; surface coarsely, irregularly punctate, impunctate behind head; medial longitudinal sulcus present on disc; basal impression absent; pronotal length 1.4 mm; pronotal width 1.9 mm. Scutellum: pentagonal; impunctate. Elytron: lateral margin straight, smooth, slightly laminate, apex rounded, smooth; sutural angle without tooth; humerus rounded, not produced; slightly constricted behind humerus; moderately punctate-striate, row 10 removed from lateral margin; elytral length 4.4 mm; elytral width 2.0 mm. Venter: prosternum smooth medially, densely coarsely punctate laterally; mesosternum punctate; metasternum sparsely punctate medially, densely punctate laterally; abdominal sterna punctate, denser and larger laterally, each puncture with pale seta; suture between abdominal sterna 1 and 2 complete; last sternite with apical margin emarginate medially in male. Leg: slender; coxa, femur, and tibia punctate. Total length: 6.2 mm.

**Figures 62–70.**
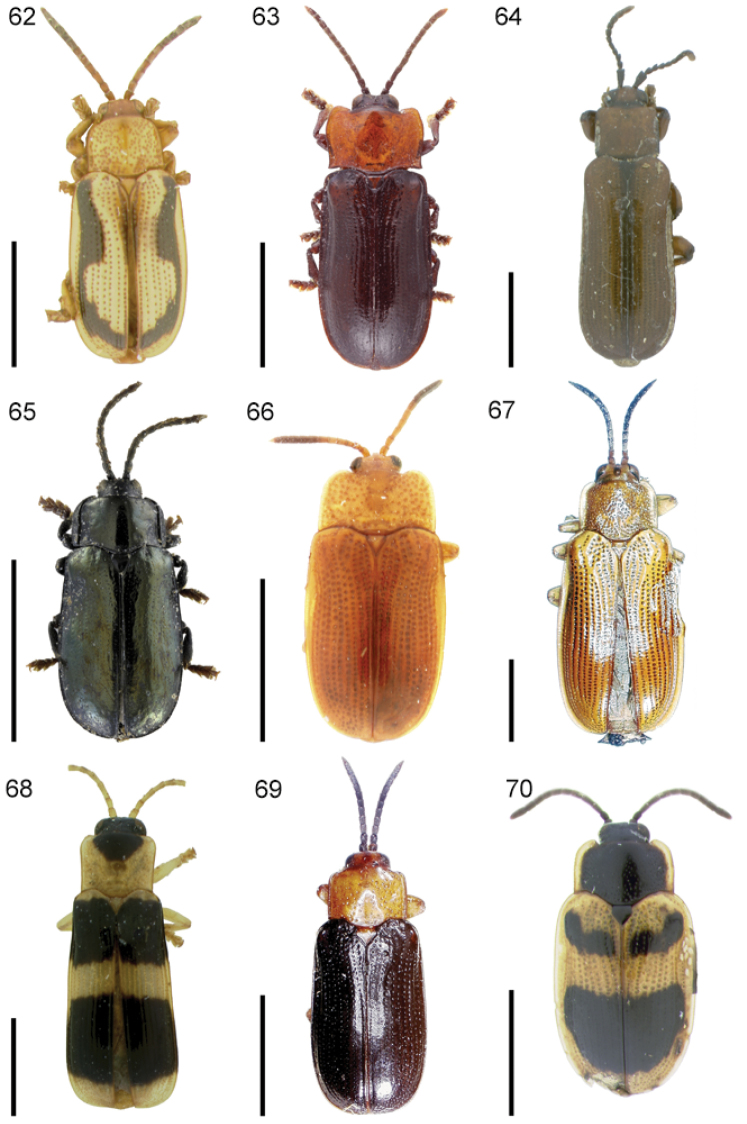
Habitus. **62** *Cephaloleia abdita* sp. n. **63** *Cephaloleia abdominalis* **64** *Cephaloleia adusta* **65** *Cephaloleia aeneipennis* **66** *Cephaloleia aequilata* **67** *Cephaloleia affinis* **68** *Cephaloleia alternans* **69** *Cephaloleia amazona* **70** *Cephaloleia amba* sp. n. Scale bars equal 3 mm.

##### Etymology.

Abdita (Latin) meaning forgotten since this species has been overlooked since being collected in 1899. The name is feminine.

##### Diagnosis.

This species is most similar to *Cephaloleia gemma* sp. n. and *Cephaloleia trilineata*. It can be distinguished from *Cephaloleia gemma* by the yellowish coloration (black in *Cephaloleia gemma*) and larger size (6.2 mm versus 4.0 mm). It can be distinguished from *Cephaloleia trilineata* by lacking a small tooth in the sutural angle of the elytra and that elytral puncture rows 3 and 4 are not confused basally.

##### Distribution.

Brazil (São Paulo).

##### Type series.

Holotype male: Brézil, et. de São Paulo, Cerqueira Cesar, E. Gounelle, i-99/ F. Monros collection 1959/ Holotype *Cephaloleia abdita* Staines, des. C. L. Staines 2012 [red label] (USNM).

#### Cephaloleia abdominalis

Taxon classificationAnimaliaColeopteraChrysomelidae

Pic, 1926c

http://species-id.net/wiki/Cephaloleia_abdominalis

Fig. 63

Cephalolia abdominalis Pic 1926c: 9. Uhmann 1953d: 47 (faunal list).
Cephaloleia abdominalis Pic. Uhmann 1957b: 14 (catalog), 1964a: 402 (catalog); Descarpentries and Villiers 1959a: 139 (types).

##### Description.

Elongate; subparallel; subdepressed; shining; black; pronotum, scutellum, and abdomen testaceous; elytra with reddish tinge at humeri. Head: vertex sparsely punctate, medial sulcus present; frons not projecting; depressed between eyes. Antenna: longer than head and pronotum combined; antennomere 1 incrassate; 2 ¾ length of 1; 3–4 elongate, 3 as long as 1 and 2 combined, longest; 4 ¾ length of 3; 5–10 transverse, decreasing in length; 11 longer than 10, acutely pointed at apex; 1–3 punctate with scattered setae; 4–11 setose. Pronotum: transverse; lateral margin sinuate then rounding to anterior angle, margined, very finely serrulate; anterior angle rounded, slightly produced; posterior angle acute; anterior margin emarginate behind head; disc subconvex; surface sparsely, irregularly punctate, punctures more dense laterally and basally; disc nearly impunctate; transverse basal impression present; pronotal length 1.3 mm; pronotal width 2.0 mm. Scutellum: pentagonal; impunctate. Elytron: lateral margin straight, moderately margined, finely serrulate below humerus; apex rounded; sutural angle without tooth; humerus rounded, slightly produced; slightly constricted behind humerus; subconvex; moderately punctate-striate, punctures obsolete apically; with medial longitudinal depression on disc; elytral width 4.2 mm; elytral width 2.2 mm. Venter: obscured by card mount. Leg: femur and tibia punctate, each puncture with pale seta. Total length: 7.0 mm.

##### Diagnosis.

This species falls into a group of bicolored species with pronotum yellowish or reddish which includes *Cephaloleia amazona*, *Cephaloleia princeps*, *Cephaloleia steinhauseni*, *Cephaloleia susanae* sp. n., and *Cephaloleia teutonica*. It can be distinguished from all of these species by the sinuate lateral margins of the pronotum, by the finely serrulate lateral margin of the elytra below the humerus, and the immaculate pronotum.

##### Distribution.

Peru.

##### Type material examined.

Pérou, Prov. Huallaga, Tocache, 500 m, G A Baer, 10-11-1900 [printed green label)/ abdominalis sp. n. [handwritten label]/ Type [handwritten label]/ coll. Pic [handwritten label]/ Museum Paris, Coll. M. Pic [blue printed label]/ Type [printed red label]/ *Cephaleia abdominalis* Pic [printed label]/ Holotype [printed red label]/ MNHN EC 2644 [printed label] (MNHN).

#### Cephaloleia adusta

Taxon classificationAnimaliaColeopteraChrysomelidae

Uhmann, 1930a

http://species-id.net/wiki/Cephaloleia_adusta

Fig. 64

Cephalolia adusta Uhmann 1930a: 218. Uhmann 1936b: 585 (key).
Cephaloleia adusta Uhmann. Blackwelder 1946: 718 (catalog); Uhmann 1950a: 274; Papp 1953: 13 (catalog); Uhmann 1957a: 15 (catalog); Gaedike and Döbler 1971: 342 (types); Wilcox 1983: 136 (catalog); Staines 1996: 14 (Central America species), 1997: 413 (Uhmann species list), 2004: 312 (host plants); Staines and Staines 1997: 2 (types); McKenna and Farrell 2005: 119 (phylogeny).

##### Description.

Elongate; subparallel; subdepressed; yellowish-brown, antennae black, except apical three antennomeres which are brownish; head near eyes black; pronotum with fine narrow black margins; scutellum and elytral suture black, elytral lateral margin dark reddish-brown. Head: vertex with scattered punctures, medial sulcus absent; frons triangular, projecting; not depressed between eyes. Antenna: reaches base of the pronotum; slender; antennomere 1 longest, expanding apically, clavate (especially in male); 2–4 combined as long as 1, subequal in length, laterally compressed, 3–4 projecting on inner margin; 5–10 elongate, subequal in length; 11 long oval; 1–4 punctate with scattered setae; 5–11 setose. Pronotum: transverse, slightly narrower apically than basally; lateral margin straight then rounding to anterior angle; anterior angle rounded, not projecting; posterior angle angulate; anterior margin weakly emarginate behind head; disc flattened; smooth; surface finely, irregularly punctate; basal impression absent; pronotal length 1.4–1.7 mm; pronotal width 1.9–2.1 mm. Scutellum: elongate pentagonal; impunctate. Elytron: lateral margin straight, smooth, rounding to apex; apex rounded; sutural angle angulate, without tooth; humerus rounded, not produced; slightly constricted behind humerus; finely punctate-striate, rows becoming obsolete after middle, apical punctures confused; elytral length 6.1–6.7 mm; elytral width 2.6–2.8 mm. Venter: inner margin of epipleuron ciliate; pro-, meso-, and metasterna impunctate; abdominal sterna punctate, each puncture with pale seta; suture between abdominal sterna 1 and 2 complete; last sternite with apical margin truncate, weakly emarginate in male, bisinuate in female. Leg: thickened, flattened; punctate; tibia with fringe of setae on inner margin of apex. Total length: 8.6–10.5 mm.

##### Diagnosis.

This species is most similar to *Cephaloleia kressi* sp. n. It can be distinguished by the following combination of characters: the elytra without a declivity from puncture row 7, the elytral punctation is obsolete after the middle, by the vertex of the head without a medial sulcus, by the angulate sutural angle of the elytra, and by the impunctate pro-, meso-, and metasterna.

##### Host plant.

Adults have been collected on *Heliconia* sp. (Heliconiaceae) (Staines 1996).

##### Distribution.

Costa Rica.

##### Type material examined.

Paralectotype female: Costa Rica, F. Nevermann [green label]/ Westabhang des Vulkans, Irazu, 1500–2000 m [reversed green label]/ Allotype [red label]/ *Cephalolia adusta* [female] sp. n. Uhmann 28/ Type No. 54625 USNM [orange label] (USNM).

##### Specimens examined.

**COSTA RICA:** Alajuela- Bijagua de Upala, Alberge de Heliconias, 1000–1100 m, 18 June 2000 (USNM); Río San Lorencito, 900 m, R. F. San Ramón, 5 km N de Colonia Palmareña, 13–18 June 1993 (INBIO); San Ramón, Angles, R.B. Alberto Brenes, 1000–1100 m (INBIO); E. B. San Ramón, R. B. San Ramón, 27 km N and 8 km W San Ramón, 8 July 2000 (USNM). Cartago- Las Palmas (USNM); Orosí, 1200 m, 28 August 1931 (DEI, USNM); Ref. Nac. Fauna Silv. Tapantí, 1050 m, August 1991 (INBIO); Quebrada Segunda, Ref. Nac. Fauna Silv. Tapantí, 1250 m, April 1992, August 1992, December 1992, May 1992, March 1993, September 1992, October 1992 (INBIO); Rancho Quemado, Pen. Osa, February 1991, May 1992, June 1992, April 1992 (INBIO); Río Grande de Orosí, 1500–1600 m (INBIO); Valle Orosí, Tapantí, 1500 m, 25 May 1941 (MUCR); Westabnung, Vulka Irazú, 1500–2000 m, 23 February 1925 (DEI, USNM). Guanacaste- Est. Pitilla, 700 m, 9 km S Sta. Cecilia, March 1991 (INBIO); Río San Lorenzo, 1050 m, Tierras Morenas, Z. P. Tenorio, March 1991, August 1992, November 1991, 23 March- 21 April 1992, July 1992, March 1990, January 1992, October 1991, December 1992, April 1991, April 1992, January 1993, June 1991, October 1992, February 1993 (INBIO). Heredia- El Angel Falls, 21 June 1969 (USNM). Puntarenas- Alajuela, Monteverde For. Res., 1600 m, 17–18 August 1976 (CASC); Monteverde (EGRC); Monteverde Reserve, 1500 m, 1 June 1979 (CMNC); Monteverde Cloud For. Res., 18–19 May 1985, 20 May 1985 (EMEC); A. C. A., Central Reserva Bosque Eterno de los Niños, El Camino, 1500–1600 m (INBIO); P. N. Piedras Blancas, Estation Esquinas, 0–100 m (INBIO); A. C. O., Golfito, Pque Nal Corcovado, Est. Sirena, 0–100 m (INBIO); Osa, Sierpe, Rancho Quemado, 200 a 300 m (INBIO); Est. La Casona, Las Torres, Z. P. Arenal Monteverde, 1500–1600 m (INBIO); Perez Zeledón, Santa Elena, 1200 m (INBIO). San José- San José (USNM). Total: 448.

#### Cephaloleia aeneipennis

Taxon classificationAnimaliaColeopteraChrysomelidae

Baly, 1858

http://species-id.net/wiki/Cephaloleia_aeneipennis

Fig. 65

Cephalolia aeneipennis Baly1858: 59. Gemminger and Harold 1876: 3601 (catalog); Donckier 1899: 547 (catalog); Weise 1911a: 7 (catalog), 1911b: 13 (catalog); Bryant 1942: 205 (faunal list); Staines and Staines 1999: 523 (Baly species list).
Cephaloleia aeneipennis Baly. Uhmann 1957b: 15 (catalog).

##### Description.

Elongate, elytra slightly expanding apically; depressed; black, often with golden, bluish or green sheen; shining; small. Head: vertex with faint medial sulcus, alutaceous; frons impunctate, not projecting; keel present between antennal bases; not depressed between eyes. Antenna: more than ½ body length; slender; antennomere 1 subglobose, shorter than 2; 2–3 elongate, subequal in length; 4–5 subequal in length, elongate, each shorter than 2; 6–10 subequal in length, elongate, each shorter than 5; 11 pointed at apex; 1 impunctate; 2 with scattered punctures; 3–11 setose. Pronotum: transverse; lateral margin straight for basal 4/5 then rounded to anterior angle, broadly margined; anterior angle rounded with small acute tooth; posterior angle acute; anterior margin weakly emarginate behind head; with sulcus parallel to lateral margin from near posterior angle to near anterior angle; disc convex; surface alutaceous with scattered punctures; basal impression absent; pronotal length 0.9 mm; pronotal width 1.4 mm. Scutellum: triangular; punctate. Elytron: lateral and apical margins smooth; lateral margin straight, margined; apex rounded; sutural angle with small tooth; humerus rounded, not produced; slightly constricted behind humerus; subconvex; shallowly punctate-striate, punctures near suture indistinct; elytral length 3.0 mm; elytral width 1.7 mm. Venter: pro- and mesosterna punctate; metasternum impunctate medially, punctate laterally; abdominal sterna irregularly punctate, each puncture with pale seta; suture between sterna 1 and 2 complete; last sternite with apical margin truncate, slightly sinuate on either side in female. Leg: sparsely punctate; femur and tibia with row of setae on inner margin; tibia with tuft of setae at apex. Total length: 4.0–4.5 mm.

##### Diagnosis.

This species is easily distinguished from all other known *Cephaloleia* by the distinct sulcus parallel to the lateral margins of the pronotum.

##### Distribution.

Colombia, Venezuela.

##### Type material examined.

Holotype: Venezuela [handwritten label]/ Baly Coll. [printed label]/ Cephalolia aeneipennis Baly, Venezuela [blue handwritten label] (BMNH).

##### Specimens examined.

**COLOMBIA:** no further data (NMW). Total: 1.

#### Cephaloleia aequilata

Taxon classificationAnimaliaColeopteraChrysomelidae

Uhmann, 1930a

http://species-id.net/wiki/Cephaloleia_aequilata

Fig. 66

Cephalolia aequilata Uhmann 1930a: 223. Uhmann 1942: 96 (noted).
Cephaloleia aequilata Uhmann. Blackwelder 1946: 718 (catalog); Uhmann 1950b: 336 (type); Papp 1953: 13 (catalog); Uhmann 1957a: 15 (catalog); Gaedike and Döbler 1971: 342 (types); Wilcox 1983: 136 (catalog); Staines 1996: 15 (Central America species), 1997: 413 (noted), 2011: 48 (faunal list); Staines and Staines 1997: 2 (types); McKenna and Farrell 2005: 119 (phylogeny), 2006: 10949 (phylogeny).

##### Description.

Small, almost rectangular in outline, depressed; reddish-brown; antennomeres 1–5 reddish, 6–10 black, 11 reddish. Head: vertex finely punctate; eyes dark; frons projecting; not depressed between eyes. Antenna: reaches to humerus; slender; antennomeres 1–2 elongate, subequal in length; 3 not compressed or widened, subequal in length to 1 or 2; 4–6 subequal in length, each shorter than 3; 7–10 transverse, subequal in length, each shorter than 6; 11 2× length of 10, oval; 1–6 punctate with scattered setae; 7–11 setose. Pronotum: twice as wide as long; lateral margin straight basally, rounding to anterior angle, margined; anterior angle rounded, slightly produced; posterior angle acute; anterior margin emarginate behind head; disc with surface sparsely, finely, irregularly punctate; irregularly, coarsely punctate laterally; basal impression absent; pronotal length 0.7–0.9 mm; pronotal width 1.4–1.7 mm. Scutellum: elongate pentagonal; impunctate. Elytron: lateral margin straight, smooth, margined; apex rounded; sutural angle without tooth; weakly expanded at humerus; slightly constricted behind humerus; moderately punctate-striate, rows 6–9 obscure on humerus; elytral length 3.4–4.3 mm; elytral width 2.0 mm. Venter: episternum punctate; epipleuron finely punctate, setose; pro-, meso, and metasterna punctate; abdominal sterna punctate, each puncture with pale seta; suture between sterna 1 and 2 complete; last sternite with apical margin semicircular, deeply emarginate medially in male, truncate in female. Leg: punctate; tibia with fringe of setae on inner margin of apex. Total length: 4.3–5.6 mm.

##### Diagnosis.

This species is one of the reddish-brown to yellow, nearly rectangular species, which includes *Cephaloleia cognata* and *Cephaloleia dilaticollis*. It can be easily distinguished from the other two species by the elytral puncture rows 6 to 9 being obsolete on the humerus.

##### Distribution.

Costa Rica, Guatemala.

##### Type material examined.

Syntype: Costa Rica, Hamburg Farm, Reventazon [green printed label]/ Ebene Limon, XII.1923, Nevermann [reversed green label]/ type [printed red label]/ Cephalilia aequaliata ♀ Uhmann 28 (DEI, 1).

##### Specimens examined.

**COSTA RICA:** Cartago- Turrialba (USNM). Guanacaste- Est. Cacao, 1000–1400 m, Lado suroeste de Volcán Cacao, June 1990 (INBIO); Est. Pitilla, 700 m, 8 km S Sta. Cecilia, 3–18 October 1991, 4–25 November 1991, December 1989, March 1990, January-April 1992 (INBIO); Río San Lorenzo, 1050 m, Tierras Morenas, Z. P. Tenorio, August 1992 (INBIO); Liberia, Mayorga, Estación Cacao, 2 km SW Cerro Cacao, 900–1000 m (INBIO); Río San Lorenzo, Tierras Morenas, 900–100 m (INBIO). Limón- Río Reventazón near Siquirres, 14 March 1991 (EGRC); Talamanca, Amubri, 0–100 m (INBIO). **GUATEMALA:** Zacapa- 3.5 km SE La Unión, 1500 m, 27 June 1993 (SEMC). Total: 29.

#### Cephaloleia affinis

Taxon classificationAnimaliaColeopteraChrysomelidae

Baly, 1858

http://species-id.net/wiki/Cephaloleia_affinis

Fig. 67

Cephalolia affinis Baly 1858: 44. Gemminger and Harold 1876: 3601 (catalog); Donckier 1899: 547 (catalog); Weise 1904b: 437 (noted), 1911a: 7 (catalog), 1911b: 11 (catalog); Uhmann 1935b: 47 (faunal list), 1936b: 113 (noted), 1942b: 96 (noted).
Cephaloleia affinis Baly Waterhouse 1881: 261 (distribution); Uhmann 1957b: 15 (catalog); Staines and Staines 1999: 523 (Baly species list).

##### Description.

Elongate; subdepressed; shining; reddish-yellow; eyes and antennomeres 3–11 dark. Head: vertex impunctate, with deep fovea; frons not projecting; slightly depressed between eyes. Antenna: reaches to humerus; slender; antennomere 1 compressed, thick, longer than 2, truncate at apex, with short acute tooth at apex; 2 short, transverse; 3 longer than 1; 4–5 elongate, subequal in length, each shorter than 3; 6–10 subequal in length, each shorter than 4; 11 2× length of 10, acutely pointed at apex; 1–2 punctate with scattered setae; 3–11 setose. Pronotum: transverse; lateral margin straight in female, diverging in male, then rounding to anterior angle, canaliculate; anterior angle rounded, slightly produced; posterior angle acute; anterior margin emarginate behind head; disc subconvex; surface densely punctate, punctures coarser basally; transverse basal impression present medially; pronotal length 2.1–2.4 mm; pronotal width 2.7–2.9 mm. Scutellum: pentagonal; impunctate. Elytron: lateral margin straight, smooth, slightly margined; apex obtusely rounded; sutural angle without tooth; humerus rounded, not produced; slightly constricted behind humerus; moderately punctate-striate, punctures somewhat confused basally; elytral length 6.9–7.2 mm; elytral width 3.6–4.0 mm. Venter: prosternum impunctate; meso- and metasterna impunctate medially, punctate laterally; abdominal sterna punctate, each puncture with pale seta; suture between sterna 1 and 2 complete; last sternite with apical margin sinuate in male, obtusely rounded in female. Leg: femur and tibia punctate; tibia expanding apically, with fringe of setae on inner margin of apex. Total length: 9.0–10.1 mm.

##### Diagnosis.

This species is one of the immaculate reddish-brown species. It can be easily be distinguished form all other species with this color pattern by the deep medial fovea on the vertex of the head.

##### Host plant.

According to label data adults have have been collected on *Ischnosiphon* sp. or *Monotagna* sp. (Marantaceae) and *Heliconia stricta* Huber (Heliconiaceae).

##### Distribution.

Bolivia, Brazil (Amazonas, Pará, São Paulo), Colombia, Ecuador, French Guiana, Guyana, Peru, Suriname, Venezuela.

##### Type material examined.

Syntype female: Brazil [handwritten label]/ Baly coll. [printed label]/ Cephalolia affinis Baly, Brazil [handwritten blue label] (BMNH, 1).

##### Specimens examined.

?- Upper Amazon (BMNH). **Bolivia:** La Paz- 9.4 km E. Chulumani, Apa, Apa Ecol. Reserve, 2100–2400 m, 18 January 2001 (SEMC). **Brazil:** Pará- (BMNH). São Paulo- August-September 1879 (USNM). **Colombia:** Valle- 18 km N Cali, 22 January 1982 (USNM). **Ecuador:** Los Ríos- Río Palenque, 47 km S Sto. Domingo, 220 m, 26 August 1997 (CDFA). Napo- Limoncocha, 300 m, 31 March 1974 (EGRC, USNM); Limoncocha Reserve, 10 August 1997 (CDFA); Sacha Lodge, 13–23 April 1994 (SEMC); Shushufindi, 215 m, 12 August 1997 (CDFA, USNM). Orellana- Estacion Cientifica Yasuni, 16 August 1997 (CDFA); 11 km W Plano, 500 m, 20 August 1997 (CDFA); Yasuni, 10–13 August 1998 (USNM). Sucua- Santa Cecilia NP, 25–31 March 1969 (USNM). **French guiana:** Roura, 8.4 km SSE, 200 m, 28 May 1997 (SEMC); Roura, 18.4 km SSE, 240 m, 29 May 1997 (SEMC); Saul, 7 km N Les Eaux Claires, 31 May 1997 (SEMC). **Guyana:** region 8, Iwokrama Forest, Kabocalli Field Station, 60 m, 21 May 2001, 5 June 2001 (SEMC). **Peru:** Loreto- Reserva Alpahuayo Mishana, 27 May 2005 (USNM). Pasco- Villa Rica Rd., 1150 m, 15 October 1999 (SEMC, USNM). Madre de Dios- CICRA Field Station, 272 m, 12 June 2011 (SEMC). Ucayali- Tingo Maria, February 1950 (USNM); Tingo Maria-Pucalpa Rd., Puenta Chino Rd. Km 205, 1300 m, 14 October 1999 (USNM). **Suriname:** Akintosoela, CELOS Camp, 39 km SE Suriname River bridge, road to Redi Doti, 29 June- 3 July 1999 (SEMC). **Venezuela:** Zuila- Kasmera, Río Yasa, Sierra de Perija, 350 m, 19 September 1961 (USNM). Total: 72.

#### Cephaloleia alternans

Taxon classificationAnimaliaColeopteraChrysomelidae

Waterhouse, 1881

http://species-id.net/wiki/Cephaloleia_alternans

Fig. 68

Cephaloleia alternans Waterhouse 1881: 261. Dohrn 1885: 145 (morphology); Uhmann 1957b: 15 (catalog); McKenna and Farrell 2005: 119 (phylogeny), 2006: 10949 (phylogeny).
Cephalolia alternans Waterhouse. Donckier 1899: 547 (catalog); Weise 1911a: 7 (catalog), 1911b: 11 (catalog); Uhmann 1932c: 261 (museum list).

##### Description.

Elongate; subdepressed; shining; yellow; head with vertex black; antennomeres 1–6 fuscous-yellow, 7–11 clear yellow; pronotum yellow with black trapezoidal macula on middle of anterior margin; scutellum yellow; elytra yellow with transverse black band near base and another on apical ⅓. Head: vertex finely, sparsely punctate, medial sulcus absent; eyes slightly convex; frons not projecting; not depressed between eyes. Antenna: longer than head and pronotum combined; slender; antennomere 1 clavate, thick; 2–4 elongate, 2 shorter than 1, 3 longer than 1; 4 subequal in length to 2; 5–6 subequal in length, each shorter than 4; 7–10 transverse, subequal in length, each shorter than 6; 11 longer than 10, rounded at apex; 1–4 punctate with scattered setae; 5–11 setose. Pronotum: transverse; lateral margin sinuate at base, then straight and divergent and rounding to anterior angle, slightly canaliculate; anterior angle obtuse, not produced; posterior angle acute; anterior margin emarginate behind head: disc flattened; surface with disc impunctate, punctate laterally; medial transverse impression present on basal margin; pronotal length 1.7–2.1 mm; pronotal width 2.1–2.4 mm. Scutellum: elongate triangular; impunctate. Elytron: lateral margin straight, smooth, slightly laminate; apex rounded; sutural angle without tooth; humerus rounded, not produced; slightly constricted behind humerus; weakly punctate-striate, punctures confused apically; elytral length 6.5–6.8 mm; elytral width 2.6–3.0 mm. Venter: pro- and mesosterna impunctate; metasternum impunctate medially, punctate laterally; abdominal sterna punctate, each puncture with pale seta; suture between sterna 1 and 2 complete. Leg: punctate; tibia with seta in each puncture, fringe of setae at apex. Total length: 8.8–9.2 mm.

##### Diagnosis.

This species is one of the yellowish species with black transverse bands on the elytra and pale elytral margins. It can be distinguished from all other species with this color pattern by the following combination of characters: the vertex of the head lacking a medial carina, sulcus or fovea, by the lateral margin of the pronotum being canaliculate, by antennomere 2 being elongate rather than subglobose, and by lacking a declivity on the elytra.

##### Host plant.

Accodring to label data adults have been collected on *Calathea lanata* Peterson (Marantaceae).

##### Distribution.

Ecuador, Peru.

##### Type material examined.

Holotype: Type H. T. [White disk with red border]/ Ecuador, Sarayacu [handwritten label]/ Buckley [handwritten label]/ Cephalolia aternans Waterh., C. Waterh. (Type) [handwritten label] (BMNH).

##### Specimens examined.

**Ecuador:** ?: 1880 (USNM). Morona Santiago- Macas (USNM). Orellana- 1 km S Onkone Gare Camp, Reserva Etnica Waorani, 216.3 m, 4 February 1996 (USNM). Pastaza- Kapawi, 300 m, 6 March–20 June 1996 (SEMC). **Peru:** Loreto- Madreselva Biol. Stn., 24 June 2005 (USNM). Total: 7.

#### Cephaloleia amazona

Taxon classificationAnimaliaColeopteraChrysomelidae

Baly, 1869

http://species-id.net/wiki/Cephaloleia_amazona

Fig. 69

Cephalolia amazona Baly 1869: 369. Gemminger and Harold 1876: 3601 (catalog); Donckier 1899: 547 (catalog); Weise 1911a: 7 (catalog), 1911b: 11 (catalog).
Cephaloleia amazona Baly. Uhmann 1942b: 96 (pygidium), 1957b: 15 (catalog); Staines and Staines 1999: 523 (Baly species list).

##### Description.

Elongate; subparallel; subdepressed; elytra black, abdomen yellowish, head, antennae, pronotum, and scutellum reddish-yellow. Head: vertex sparsely punctate, medial sulcus present; frons not projecting; not depressed between eyes. Antenna: less than ½ body length; antennomere 1 thickened, longer than 2; 2 longer than 3; 3–5 subequal in length; 6–10 transverse, shorter than preceding; 11 pointed at apex; 1–2 punctate with scattered setae; 3–11 setose. Pronotum: transverse; lateral margin straight, slightly diverging for basal ⅔ then rounding to anterior angle, canaliculate; anterior angle rectangular; posterior angle acute; anterior margin rounded anteriorly; disc subconvex, concave laterally; surface with sparse, rounded punctures; basal impression absent; pronotal length 1.4–1.6 mm; pronotal width 1.7–2.1 mm. Scutellum: pentagonal; impunctate. Elytron: lateral margin straight, smooth; apex rounded; sutural angle without tooth; humerus rounded, not produced; slightly constricted behind humerus; finely punctate-striate, punctures more impressed laterally; surface finely irregularly wrinkled; elytral length 4.4–4.7 mm; elytral width 2.3–2.7 mm. Venter: pro-, meso- and metasterna impunctate; abdominal sterna punctate, each puncture with pale seta; suture between sterna 1 and 2 obsolete medially; apical abdominal sternite subangulate-emarginate in male, bisinuate in female. Leg: punctate; femur with fringe of setae on inner margin; tibia with fringe of setae on inner margin of apex. Total length: 5.6–6.8 mm.

##### Diagnosis.

This species falls into a group of bicolored species with a reddish pronotum which includes *Cephaloleia abdominalis*, *Cephaloleia princeps*, *Cephaloleia steinhauseni*, *Cephaloleia susanae* sp. n., and *Cephaloleia teutonica*. It can be distinguished from these species by the vertex of the head lacking a medial fovea but having a medial sulcus and by the pronotum with straight lateral margins which are not serrulate.

##### Host plant.

According to label data adults have been collected on *Heliconia velutina* L. Anderson (Heliconiaceae).

##### Distribution.

Brazil (Minas Gerais, Santa Catharina), Peru.

##### Type material examined.

Syntype: Upper Amazons [handwritten label]/ Baly Coll. [printed label]/ Cephalolia amazona Baly, Upper Amazons [blue handwritten label] (BMNH, 1).

##### Specimens examined.

?- no label data (USNM). **Brazil:** Minas Gerais- Vila Monte Verde, 6 December 1974 (USNM). Santa Catharina- Theresopolis, 1887 (USNM). **Peru:** Loreto- Madreselva Biol. Stn., 27 May 2005 (USNM); Reserva Alpahuayo Mishana, 27 May 2005 (USNM); 1.5 km N Teniente Lopez, 210–240 m, 20 July 1993 (SEMC). Total: 8.

#### Cephaloleia amba

Taxon classificationAnimaliaColeopteraChrysomelidae

Staines sp. n.

http://zoobank.org/BB59F1A1-2C83-4E2E-B0EC-E956CE1350B9

http://species-id.net/wiki/Cephaloleia_amba

Fig. 70

##### Description.

Obovate; subconvex; head, scutellum, and pronotum (except lateral margin) black; elytra pale yellow with black irregular transverse band from puncture row 1 to 10 across humerus, apical ½ (except lateral and apical margins) black; venter brownish. Head: vertex finely punctate, each puncture with white seta, medial sulcus faint; frons not projecting; not depressed between eyes. Antenna: reaches beyond humerus; slender; antennomeres 1 and 2 transverse, short; 3–4 subequal in length, each 2× length of 2; 5–10 subequal in length, each ¾ length of 3; 11 2× length of 10, pointed at apex; 1–2 punctate with scattered setae, 3–11 setose. Pronotum: transverse; lateral margin straight then rounding to anterior angle, serrulate; anterior angle obtusely rounded, not produced; posterior angle acute; anterior margin emarginate behind head; disc subvoncex; surface irregularly punctate, punctures more dense basally and laterally; basal impression absent; pronotal length 1.1 mm; pronotal width 1.7–2.0 mm. Scutellum: pentagonal; alutaceous. Elytron: lateral margin straight for basal ⅔ then rounded to apex, smooth; apical margin rounded, smooth; sutural angle without tooth; humerus rounded, not produced; slightly constricted behind humerus; moderately punctate-striate, row 10 removed from lateral margin; elytral length 3.7–4.3 mm; elytral width 2.4–2.6 mm. Venter: prosternum slightly rugose medially, punctate laterally; meso- and metasterna slightly punctate medially, punctate laterally; abdominal sterna punctate, each puncture with pale seta; suture between abdominal sterna 1 and 2 obsolete medially; last sternite with apical margin broadly emarginate medially in male. Leg: femur and tibia punctate, each puncture with seta; tibia with fringe of setae on inner margin of apex. Total length: 4.9–5.6 mm.

##### Etymology.

From ambon (Greek) meaning edge for the pale flange of the pronotum and elytra. The name is a noun in apposition.

##### Diagnosis.

This species belongs to the group of species with a mostly dark pronotum which yellowish elytra with black transverse bands. It is most similar to *Cephaloleia degandei* and can be distinguished by the serrulate lateral margin of the pronotum and by antennomeres 1 and 2 being subequal in length.

##### Host plant.

Accodring to label data adults have been collected on *Costus* sp. (Costaceae).

##### Distribution.

Colombia, Ecuador, Peru.

##### Type material.

Holotype male: Colombia, Amazonas Pr., Mico (“Monkey”) Island, R. Amazonas, ca. 3°56'S, 70°8'W/ 3-VII-1978/ *Costus*/ Holotype *Cephaloleia amba* Staines, des. C. L. Staines 2012 [red label] (USNM). Paratypes (6) (each with Paratype *Cephaloleia amba* Staines, des. C. L. Staines 2012 [red label]): with same label data as holotype (USNM); Ecuador, Napo prov., 2 km S Puerto Misahauli, 01°02'46"S, 77°39'23"W, 450 m, second growth, 8.xii.2009, COST. Costus sp., L. Sekerka and L. Stajerova lgt. [green printed label] (LSC); Ecuador, Napo prov., Rio Puno, 13.xii.2009, 8 km SE of Misahuali, 400 m, 01°05'55"S, 77°38'30"W, *Costus* sp., tall, white flowers, L. Sekerka and L. Stajerova lgt [green printed label] (BMNH, LSC); Ecuador, Sucumbios pr., Lumbaqui, Cost. *Costus* sp., D. Windsor lgt., 5.ii.2007 (DWC); Peru, Madre de Dios, Rio Tambopata Reserve, 30 air km SW of Puerto Maldenado, 290 m, November 1–26 1982, Edward S. Ross (CASC).

#### Cephaloleia amblys

Taxon classificationAnimaliaColeopteraChrysomelidae

Staines, 1996

http://species-id.net/wiki/Cephaloleia_amblys

Image not available

Cephaloleia amblys Staines 1996: 15.

##### Description.

Elongate; subparallel; subconvex; brownish; head and pronotum darker; venter with pro-, meso-, and metasterna pale medially, dark laterally. Head: vertex punctate, medial sulcus absent; frons not projecting; not depressed between eyes. Antenna: reaches to humerus; slender; antennomere 1 elongate, longer than 2 or 3; 2–3 elongate, subequal in length, each shorter than 1; 4–10 transverse, subequal in length, each shorter than 3; 11 rounded at apex, subequal in length to 1; 1–4 punctate with scattered setae; 5–11 setose. Pronotum: transverse; lateral margin canaliculate, straight and divergent for basal ½ then rounding to anterior angle; anterior angle rounded, not produced; posterior angle acute; anterior margin curved behind head; disc subconvex; surface sparsely punctate, more dense laterally, apical margin impunctate; basal impression absent; pronotal length 0.7 mm; pronotal width 1.1 mm. Scutellum: pentagonal; impunctate. Elytron: lateral margin straight, smooth, margined; apex rounded; sutural angle without tooth; humerus rounded, not produced; slightly constricted behind humerus; weakly punctate-striate, humerus virtually impunctate; puncture rows converge and unite at apex; elytral length 2.4 mm; elytral width 1.3 mm. Venter: prosternum impunctate; meso- and metasterna punctate laterally; abdominal sterna punctate, each puncture with pale seta; suture between sterna 1 and 2 complete. Leg: robust; tibia with fringe of setae on inner margin of apex. Total length: 3.4 mm.

##### Diagnosis.

This species is most similar to *Cephaloleia facetus*. It can be distinguished by the pronotum being darker in color than the elytra and with antennomere 1 being longer than 2.

##### Distribution.

Panama.

##### Type material.

Holotype: Panama: Canal Zone, Madden Forest, mi 5.0, 9°07'N, 79°38'W/ 19-vii-1971, H. A. Hespenheide/ Cephaloleia tenella Baly CWT iii73/ Holotype Cephaloleia amblys Staines, Des. C. L. Staines 1994 [red label] (CHAH, not seen).

##### Comments.

Repeated requests for the loan of this specimen went unanswered. Image not included in the monograph.

#### Cephaloleia angustacollis

Taxon classificationAnimaliaColeopteraChrysomelidae

Staines sp. n.

http://zoobank.org/A1425B06-B0E0-4412-BB77-EA50BDD80DED

http://species-id.net/wiki/Cephaloleia_angustacollis

Fig. 71

##### Description.

Elongate; subdepressed; pale yellow; antennomeres 1-9 slightly darker; eyes black; pronotum with black demilune-shape behind head; elytra with wide black transverse band behind middle, dark macula laterally on humerus, and elongate dark macula covering puncture rows 9 and 10 from humerus to just before middle. Head: vertex finely punctate, oval basal fovea present medially, medial sulcus present; triangular projection present between antennal bases; frons not projecting; not depressed between eyes. Antenna: reaches to humerus; antennomeres 1–4 elongate; 2 ¾ length of 1; 3 longer than 1; 4 subequal in length to 2; 5–10 subequal in length and width, each widened apically, each shorter than 4; 11 2× length of 10, rounded at apex; 1–4 punctate with scattered setae; 5–11 setose. Pronotum: transverse; lateral margin straight then rounding to anterior angle, canaliculate; anterior angle rounded, not projecting; posterior angle acute; anterior margin straight; longitudinal sulcus present medially; disc flattened, virtually impunctate; basal impression absent; pronotal length 1.9–2.0 mm; pronotal width 1.9–2.0 mm. Scutellum: pentagonal; impunctate. Elytron: lateral margin straight, smooth, narrowly margined; apex rounded; small tooth present in sutural angle; humerus rounded, not produced; slightly constricted behind humerus; moderately punctate-striate, punctures confused apically; elytral length 6.4 mm; elytral width 2.4–2.7 mm. Venter: prosternum impunctate medially, punctate laterally; meso- and metasterna impunctate; abdominal sterna impunctate; suture between abdominal sterna 1 and 2 obsolete; sterna 2–4 each with shallowly curved transverse plica medially; sternite 5 with scattered setae apically, apical margin rounded in female, emarginate medially in male. Leg: slender; impunctate; tibia with fringe of setae on inner margin of apex. Total length: 9.1–10.3 mm.

**Figures 71–79.**
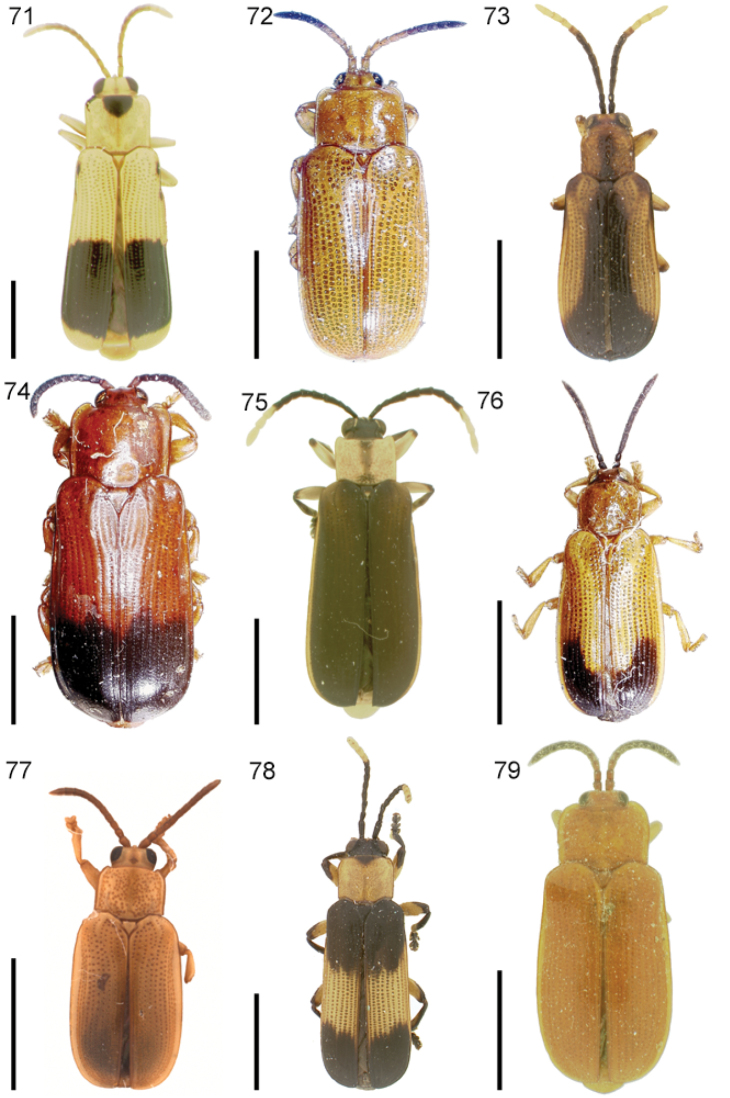
Habitus. **71** *Cephaloleia angustacollis* sp. n. **72** *Cephaloleia antennalis* **73** *Cephaloleia antennata* **74** *Cephaloleia apicalis* **75** *Cephaloleia apicata* **76** *Cephaloleia apicenotata* **77** *Cephaloleia apicicornis* **78** *Cephaloleia applicata* **79** *Cephaloleia approximata*. Scale bars equal 3 mm.

##### Etymology.

A combination of angustus (Latin) meaning narrow and collis (Latin) meaning neck for the narrow pronotum in this species. The name is feminine.

##### Diagnosis.

This is one of the mostly yellowish species with black pronotal markings, black transverse elytra bands and pale lateral margins of the elytra. It can be distinguished from all other species with this color pattern by the medial fovea on the vertex of the head.

##### Host plant.

Accodring to label data adults have been collected on *Calathea* sp. (Marantaceae).

##### Distribution.

Ecuador.

##### Type material.

Holotype male: Holotype Ecuador, Napo, Mishualli nr. Tena, 3-8 October 1999, Steven R. Keller/ *Cephaloleia angustacollis* Staines, des. C. L. Staines 2012 [red label] (BYUC). Paratypes (5) (each with Paratype *Cephaloleia angustacollis* Staines, des. C. L. Staines 2012 [red label]): Ecuador, Sucumbios, Shushufindi, 215 m, 00°0.96'S, 76°38.95'W, VIII-11-1997, Fred G. Andrews (AJGC, USNM); Ecuador, Orellana DF, EC Yasuni, Rio Tiputini, 00°40'16"S, 76°24'02"W, 26.ii-4.iii.2007, MAR. *Calathea* sp., D. Windsor lgt. [green printed label] (LSC); Ecuador, Napo, Yasuni, 215 m, 0°40.55S, 76°38.8W, 13–18 August 1997, D. M. Windsor (DWC).

#### Cephaloleia antennalis

Taxon classificationAnimaliaColeopteraChrysomelidae

Donckier, 1899

http://species-id.net/wiki/Cephaloleia_antennalis

Fig. 72

Cephaloleia antennata Baly 1885: 11 (homonym of *Cephaloleia antennata* Waterhouse, 1881). Staines and Staines 1999: 523 (Baly species list).
Cephalolia antennalis Donckier 1899: 547 (replacement name for *Cephaloleia antennata* Baly). Weise 1911a: 7 (catalog), 1911b: 10 (catalog).
Cephaloleia antennalis Donckier. Blackwelder 1946: 718 (catalog); Papp 1953: 13 (catalog); Uhmann 1957a: 15 (catalog); Wilcox 1983: 136 (catalog); Staines 1996: 16 (Central America species); McKenna and Farrell 2005: 119 (phylogeny), 2006: 10949 (phylogeny).

##### Description.

Elongate; subdepressed; shining; reddish-brown; antennomeres 1–4 yellow, 5–11 darker; eyes darker. Head: vertex sparsely punctate, medial sulcus present; frons not projecting; not depressed between eyes. Antenna: reaches to humerus; slender; antennomeres 1–4 laterally compressed, with inner anterior angle produced into acute tooth in male, elongate in female; 1 large, clavate; 2–4 subequal in length, each shorter than 1; 5–10 transverse, decreasing in length, each shorter than 4; 11 pointed at apex, subequal to 1; 1–2 punctate with scattered setae; 3–11 setose. Pronotum: transverse; lateral margin straight for basal ¾ then convergent, margined; anterior angle produced, obtuse; posterior angle angulate; anterior margin emarginate behind head; surface finely, sparsely punctate; basal impression absent; pronotal length 1.3–1.8 mm; pronotal width 2.0–2.6 mm. Scutellum: triangular; impunctate. Elytron: lateral margin straight, smooth, margined; apex rounded; sutural angle without tooth; humerus rounded, not produced; slightly constricted behind humerus; weakly punctate-striate; elytral length 5.1–6.4 mm; elytral width 2.7–3.3 mm. Venter: pro-, meso- and metasterna punctate; abdominal sterna punctate, each puncture with pale seta; suture between sterna 1 and 2 complete; last sternite with apical margin truncate in female, emarginate medially in male. Leg: femur and tibia punctate, each puncture with pale seta; femur robust; tibia expanded to apex, with fringe of setae on inner margin of apex. Total length: 6.9–8.4 mm.

##### Diagnosis.

This is one of the reddish-brown immaculate species. It can be distinguished from the other reddish-brown species by the suture between abdominal sterna 1 and 2 being complete and by antennomere 1 being as long as 2 to 4 combined.

##### Distribution.

Costa Rica, Guatemala, Panama.

##### Type material examined.

Holotype male: Type H. T. [white disk with red border]/ Panama, Volcan de Chiriquí, Champion/ B. C. A., Col. VI, 2. Cephaloleia antennata, Baly/ Cephaloleia/ Cephalolia antennata Baly, Nicaragua [blue handwritten label] (BMNH).

##### Specimens examined.

**COSTA RICA:** Alajuela- Res. For. San Ramón, 900 m, 12 March 1990 (INBIO). Cartago- Quebrada Segunda, Ref. Nac. Fauna Silv. Tapantí, 1250 m, April 1992 (INBIO); Turrialba (USNM). Guanacaste- Río San Lorenzo, 1050 m, Tierras Morenas, Z.P. Tenorio, 28 March–21 April 1992, July 1991, 10–20 February 1992, October 1992 (INBIO). Heredia- La Selva, 18 May 1993 (SEMC). Limón- Sector Cerro Cocorí, Fca. de E. Rojas, 150 m, 28 May–17 June 1992 (INBIO); Esta. Cuatro Esquinas, 0 m, P. N. Tortuguero, 27 March–29 April 1992 (INBIO); Hamburg Farm, Reventazón, Ebene Limón, 27 April 1934, 23 February 1934 (USNM). Puntarenas- Est. Biol. Las Alturas, 1500 m, Coto Brus, 23 March- 2 May 1992 (INBIO); Golfito, 10–200 m, 28 May 1993 (SEMC); Golfito, 3 July 1985 (EMEC); Rancho Quemado, 200 m, Peninsula de Osa, April 1992, 21 March–7 April 1992 (INBIO); Est. Sirena, P.N. Corcovado, September 1991, November 1989, February 1990, 21 March- 21 April 1992, 28 May–17 June 1992 (INBIO). **GUATEMALA:** Verapaz- Chacoj (USNM). Total: 46.

#### Cephaloleia antennata

Taxon classificationAnimaliaColeopteraChrysomelidae

Waterhouse, 1881

http://species-id.net/wiki/Cephaloleia_antennata

Fig. 73

Cephaloleia antennata Waterhouse 1881: 262. Uhmann 1957b: 15 (catalog).
Cephalolia antennata Waterhouse. Donckier 1899: 548 (catalog); Weise 1911a: 7 (catalog), 1911b: 11 (catalog).

##### Description.

Elongate; subparallel; convex; reddish-yellow; antennomeres 1–7, medial line of pronotum, scutellum, and elytral suture and humeri darker, head with blackish band on vertex, antennomeres 8–11 yellow; venter reddish-yellow medially, black laterally; legs yellow with femorotibial joint darkened. Head: eye large, prominent; vertex sparsely, irregularly punctate, medial sulcus absent; frons not projecting; depressed between eyes. Antenna: reaches to humerus; slender; antennomere 1 incrassate, robust; 2 transverse, less than ½ length of 1; 3 subequal in length to 1, cylindrical; 4–10 decreasing in length, each shorter than 3; 11 2× length of 10, broadly rounded at apex; 1–8 punctate; 9–11 setose. Pronotum: slightly longer than wide; lateral margin straight, rounding to anterior angle, narrowly margined; anterior angle obtusely rounded; posterior angle acute; anterior margin straight; disc subconvex; surface densely, strongly punctate; with oblique impression on each side reaching basal margin medially; pronotal length 1.4–1.7 mm; pronotal width 1.5–1.7 mm. Scutellum: pentagonal; impunctate. Elytron: lateral margin smooth, narrowly margined, slightly expanded apically; apex rounded, smooth; sutural angle without tooth; humerus slightly angulate, produced; slightly constricted behind humerus; strongly closely punctate-striate; elytral length 4.9–5.1 mm; elytral width 2.1–2.3 mm. Venter: pro-, meso-, and metasterna impunctate medially, punctate laterally; abdominal sterna punctate, each puncture with pale seta; suture between sterna 1 and 2 complete. Leg: punctate, each puncture with pale seta; tibia with fringe of setae on inner margin of apex. Total length: 6.5–7.1 mm.

##### Diagnosis.

This species is most similar to *Cephaloleia horvitzae* sp. n. It can be distinguished by the vertex of the head without a medial carina, by the pronotum having an oblique impression laterally, and by the larger size.

##### Distribution.

Brazil (Amazonas), Ecuador, Peru.

##### Type material examined.

Holotype: Type H. T. [white disk with red border]/ Ecuador, Sarayacu [handwritten label]/ Buckley [handwritten label]/ Cephaloleia antennata Waterh., C. H. Waterh. (Type) [handwritten label] (BMNH).

##### Specimens examined.

**Brazil:** Amazonas- St Paulo d’Olivenca, March 1883 (USNM). **Ecuador:** no further data (USNM). Napo- Limoncocha, 3 June 1977 (USNM); Shushufindi, 1200 m, 9 August 1998 (AJGC). **Peru:** Amazonas- May-July 1884 (USNM). Total: 5.

#### Cephaloleia apicalis

Taxon classificationAnimaliaColeopteraChrysomelidae

Baly, 1858

http://species-id.net/wiki/Cephaloleia_apicalis

Fig. 74

Cephalolia apicalis Baly 1858: 41. Gemminger and Harold 1876: 3601 (catalog); Donckier 1899: 548 (catalog); Weise 1910: 84 (noted), 1911a: 7 (catalog), 1911b: 11 (catalog); Uhmann 1936b: 111 (noted), 1936f: 482 (key).
Cephaloleia apicalis Baly. Uhmann 1957b: 15 (catalog); Staines and Staines 1999: 523 (Baly species list).

##### Description.

Elongate; subdepressed; shining; reddish-yellow, antennae (except basal antennomere), eyes, and apical ⅓ of elytra darker. Head: vertex sparsely punctate near eyes, medial sulcus absent; frons not projecting; not depressed between eyes. Antenna: as long as head and pronotum combined; robust; antennomeres 1–5 laterally subcompressed in male, 2–3 subequal in length, triangular; female with 1 incrassate, 2½ x length of 2; 3 ¾ length of 1; 4–5 subequal in length, each shorter than 3; 6–10 subequal in length, transverse; each shorter than 5; 11 2× length of 10, rounded at apex; 1–3 punctate with scattered setae; 4–11 setose. Pronotum: transverse; lateral margin straight then rounding to anterior angle, canaliculate; anterior angle obtuse, produced; posterior angle acute; anterior margin emarginate behind head; disc subconvex; surface finely, sparsely punctate; basal impression absent; pronotal length 1.9–2.2 mm; pronotal width 2.3–2.6 mm. Scutellum: pentagonal; impunctate. Elytron: lateral margin straight, narrowly margined, smooth; apical margin rounded, smooth; sutural angle emarginate; sutural angle without tooth; humerus rounded, not produced; constricted behind humerus; subconvex, somewhat flattened on disc; moderately punctate-striate; elytral length 6.0–6.2 mm; elytral width 2.8–3.1 mm. Venter: pro-, meso-, and metasterna impunctate; abdominal sterna punctate, each puncture with pale seta; suture between sterna 1 and 2 complete; pygidium obtuse; last sternite with apical margin sinuate in male, truncate in female. Leg: punctate; femur robust; tibia with fringe of setae on inner margin of apex. Total length: 7.8–9.0 mm.

##### Diagnosis.

This species belongs to a group of species with an immaculate pronotum and the apical portion of the elytra darkened. It is most similar to *Cephaloleia fulvipes*. It can be distinguished by the elytral punctures along the suture being distinct near the apex.

##### Distribution.

Colombia.

##### Type material examined.

Syntypes: Columbia [misspelling, handwritten label]/ Baly coll. [printed label]/ Cephalolia apicalis Baly, Columbia [misspelling, blue handwritten label] (BMNH, 2).

##### Specimens examined.

**Colombia:** no further data (USNM). ?: Nova Granada (USNM). Antioquia- Puerto Berrío, 8 August 1939 (USNM). Sartander- Puerto Barrie, 8 August 1938 (USNM). Total: 15.

#### Cephaloleia apicata

Taxon classificationAnimaliaColeopteraChrysomelidae

Uhmann, 1930a

http://species-id.net/wiki/Cephaloleia_apicata

Fig. 75

Cephalolia apicata Uhmann 1930a: 228. Uhmann 1942: 98 (noted).
Cephaloleia apicata Uhmann. Blackwelder 1946: 718 (catalog); Uhmann 1950b: 336 (type), 1957a: 15 (catalog), 1964a: 402 (catalog); Papp 1953: 14 (catalog); Gaedike and Döbler 1971: 342 (types); Wilcox 1983: 136 (catalog); Staines 1996: 16 (Central America species), 1997: 413 (Uhmann species list), 2004: 312 (host plants); Staines and Staines 1997: 3 (types); McKenna and Farrell 2005: 119 (phylogeny), 2006: 10949 (phylogeny).

##### Description.

Elongate; subdepressed; subparallel; hirsute; head and most of elytra black; antennomeres 1–8 black, 9–11 may be yellow, black or with the apex of 11 yellow; pronotum (some specimens with broad black medial longitudinal vitta) and lateral margin of elytra yellow; venter with pro-, meso-, and metasterna yellow medially, black laterally, abdominal sterna black medially, yellow laterally; leg with femur yellow, tibia and tarsi black. Head: almost as wide as apex of pronotum; vertex finely punctate, medial sulcus present; frons not projecting; depressed between eyes. Antenna: ½ body length; robust; antennomere 1 elongate; 2 ½ length of 1; 3 as long as 1–2 combined; 4–10 each shorter than 1, subequal in length, conical; 11 1½ length of 10, oval; 1–2 punctate with scattered setae; 3–11 densely setose. Pronotum: transverse; lateral margin almost straight, weakly convergent apically then rounding to anterior angle, margined; anterior angle pointed; posterior angle rectangular; anterior margin weakly emarginate behind head; surface sparsely punctate; wide V-shaped depression present basally; pronotal length 1.0–1.4 mm; pronotal width 1.6–1.9 mm. Scutellum: pentagonal; impunctate. Elytron: lateral margin straight, smooth, margined; apex rounded; sutural angle without tooth; humerus rounded, slightly produced; slightly constricted behind humerus; moderately punctate-striate; scutellar row very long; interspace 7 with additional puncture row; elytral length 5.9–7.3 mm; elytral width 2.3–2.8 mm. Venter: pro-, meso-, and metasterna impunctate medially, punctate laterally, each puncture with pale seta; abdominal sterna punctate, each puncture with pale seta; suture between sterna 1 and 2 complete; last sternite with apical margin straight in male, strongly curved in female. Leg: slender; punctate; femur and tibia with seta in each puncture; tibia with fringe of setae on inner margin and tuft of setae at apex. Total length: 7.4–10.0 mm.

##### Diagnosis.

This species is bicolored with the pronotum immaculate and lighter than the elytra. It is most similar to *Cephaloleia disjuncta* but can easily be distinguished by the additional row of punctures on the elytra and the coloration.

##### Host plant.

*Heliconia* sp. (Heliconiaceae) (Uhmann 1930a).

##### Distribution.

Costa Rica, Panama.

##### Type material examined.

Lectotype: Costa Rica, F. Nevermann, 20-VI-26 [green label]/ La Palma, 1050 m, Hondura [reversed green label]/ an Blättern v. Heliconica sp. [handwritten label]/ Holotype [red label]/ *Cephalolia apicata* sp. n. [male]/ Cotype No. 54632 USNM [orange label] (USNM).

##### Specimens examined.

**COSTA RICA:** Alajuela- E. B. San Ramón, R. B. San Ramón, 27 km N and 8 km W San Ramón, 8 July 2000 (SEMC, USNM); Río San Lorencito, 5 km N Colonia Palmareña, 900–1000 m (INBIO). Cartago- 1 km S. Cariblanco, 30 May 1992 (CDFA); Quebrada Segunda Ref. Nac. Fauna Silv. Tapantí, 1250 m, April 1992 (INBIO); Ref. Nac. Fauna Silv. Tapantí, 1250 m, August 1991 (INBIO); Turrialba, Santa Teresita, Monumento Nacional Guayabo, 1100–1200 m (INBIO). Heredia- Est. Sn Rafael Vara Blanca, P.N., Braulio Carillo, 1800–2000 m, April 1990, August 1991 (INBIO). Puntarenas- Alajuela-Monteverde For. Res., 1600 m, 17–18 August 1976 (CASC); Est. Leonel Hernandez, 1600 m, Res. Biol. Monteverde, January 1991 (INBIO); Est. G. Brenes, R. B. Monteverde, 1200–1300 m (INBIO); Est La Casona, Las Torres, 1500–1600 m (INBIO). San José- Est. Zurquí, 500 m, antes de Túnel, 1600 m, March 1991 (INBIO); 12 mi. N. San Isidro del General, 26 June 1969 (USNM). **PANAMA:** Bocas del Toro- 2.3 rd mi N from Continental Divide, 27 May 1993 (EGRC). Total: 24.

#### Cephaloleia apicenotata

Taxon classificationAnimaliaColeopteraChrysomelidae

Uhmann, 1938a

http://species-id.net/wiki/Cephaloleia_apicenotata

Fig. 76

Cephalolia apicenotata Uhmann 1938a: 411.
Cephaloleia apicenotata Uhmann. Uhmann 1950a: 274 (sculpture), 1957b: 15 (catalog); Gaedike and Döbler 1971: 342 (types); Staines 1997b: 413 (Uhmann species list).

##### Description.

Elongate; subdepressed; subparallel; shining; yellowish-brown; antennae, u-shaped marking on apical ⅓ of elytra, and last sternite with apical margin black. Head: vertex finely punctate, medial sulcus absent; frons not projecting; depressed between eyes. Antenna: reaches to humerus; slender; antennomeres 1 and 2 thick, cylindrical; 1 and 3 subequal in length; 2 ½ length of 1; 3–10 elongate; 4–10 decreasing in length, each shorter than 3; 11 slightly longer than 10, acutely pointed at apex; 1–2 punctate with scattered setae; 3–11 setose. Pronotum: transverse; lateral margin sinuate then rounding to anterior angle, distinctly margined; anterior angle rounded, not produced; posterior angle acute; anterior margin straight; disc subconvex; surface irregularly, sparsely punctate; basal impression absent; pronotal length 1.3–1.6 mm; pronotal width 1.6–1.9 mm. Scutellum: pentagonal; impunctate. Elytron: lateral margin straight, smooth, with broad margin; apex rounded; sutural angle without tooth; humerus rounded, not produced; slightly constricted behind humerus; finely punctate-striate; elytral length 4.7–4.9 mm; elytral width 2.1–2.4 mm. Venter: pro-, meso-, and metasterna impunctate medially, punctate laterally; abdominal sterna punctate, each puncture with pale seta; suture between sterna 1 and 2 complete; last sternite with apical margin subrounded, weakly emarginate in female, rounded in male. Leg: slender; punctate; femur and tibia with pale seta in each puncture; tibia with fringe of setae on inner margin of apex. Total length: 6.2–6.6 mm.

##### Diagnosis.

This is a bicolored species with the apical section of the elytra darker. It is most similar to *Cephaloleia bicolor* and *Cephaloleia bicoloriceps*. It can be easily distinguished by the sinuate lateral margin of the pronotum.

##### Distribution.

Brazil (Bahia), Ecuador.

##### Type material examined.

Lectotype: Brazil, Bahia [printed label]/ coll. Fry [printed label]/ Cephalolia apicenotata Uh., Det. E. Uhmann [handwritten label] (BMNH).

##### Specimens examined.

?- 3162 (USNM). **Brazil:** Bahia- no further data (DEI, USNM); St. Antonia de Barra, 1889 (USNM). **Ecuador:** Orellana- 1 km S Onkone Gare Camp, Reserva Etnica Waorani, 216.3 m, 21 January 2006 (USNM); Estación Cientifica Yasuni, 15–16 August 1997 (USNM). Total: 16.

#### Cephaloleia apicicornis

Taxon classificationAnimaliaColeopteraChrysomelidae

Baly, 1869

http://species-id.net/wiki/Cephaloleia_apicicornis

Fig. 77

Cephalolia apicicornis Baly 1869: 372. Gemminger and Harold 1876: 3601 (catalog); Donckier 1899: 548 (catalog); Weise 1911a: 7 (catalog), 1911b: 11 (catalog); Uhmann 1932a: 36 (museum list).
Cephaloleia apicicornis Baly. Uhmann 1957b: 15 (catalog); Staines and Staines 1999: 523 (Baly species list).

##### Description.

Elongate; subdepressed; subparallel; yellowish; antennomeres 1–8 brownish, 9–11 darker; eyes dark. Head: vertex finely, irregularly punctate; with deep medial sulcus; frons not projecting; depressed between eyes. Antenna: ½ body length; slender; cylindrical; antennomere 1 robust, 1½ x length of 2; 2–4 subequal in length; 5–10 subequal in length, each shorter than 4; 11 rounded at apex, 2× length of 10; 1–4 punctate with scattered setae; 5–11 setose. Pronotum: transverse; lateral margin straight then rounding to anterior angle, canaliculate; anterior angle rounded, slightly produced; posterior angle acute; anterior margin emarginate behind head; disc subconvex; surface with deep, round punctures, more dense laterally; basal impression absent; pronotal length 1.1–1.3 mm; pronotal width 1.5–1.7 mm. Scutellum: pentagonal; impunctate. Elytron: lateral margin straight, smooth, slightly laminate; apex rounded; sutural angle without tooth; humerus rounded, not produced; constricted behind humerus; disc flattened; moderately punctate-striate, punctures less impressed apically; interspaces convex; elytral length 3.8–4.2; elytral width 2.1–2.4 mm. Venter: pro-, meso-, and metasterna impunctate; abdominal sterna punctate, each puncture with pale seta; suture between sterna 1 and 2 obsolete medially; last sternite with apical margin concave-emarginate in male, bisinuate in female. Leg: slender; sparsely punctate; tibia with fringe of setae on inner margin of apex. Total length: 5.5–5.8 mm.

##### Diagnosis.

This is a unicolorous yellowish species. It is most similar to *Cephaloleia corallina*, *Cephaloleia halli*, *Cephaloleia ochra* sp. n., and *Cephaloleia proxima*. It can be distinguished from these species by the pronotum without a transverse basal impression, by the pale non-black antennae, and by the meso- and metasterna being impunctate.

##### Distribution.

Brazil (Bahia, Rio de Janeiro).

##### Type material examined.

Syntype: Rio Janeiro [handwritten label]/ Cephalolia apicicornis Baly, Rio Jan. [blue handwritten label] (BMNH, 1).

##### Specimens examined.

**Brazil:** Bahia- Belmente, 10 February 1914 (USNM). Rio de Janeiro- Nova Friburgo (BMNH, USNM). Total: 3.

#### Cephaloleia applicata

Taxon classificationAnimaliaColeopteraChrysomelidae

Pic, 1923

http://species-id.net/wiki/Cephaloleia_applicata

Fig. 78

Cephalolia applicata Pic 1923: 9. Uhmann 1938a: 407 (noted).
Cephaloleia applicata Pic. Uhmann 1957b: 15 (catalog), 1964a: 402 (catalog); Descarpentries and Villiers 1959a: 139 (types).

##### Description.

Elongate; subdepressed; subparallel; yellowish-brown; head black; antennomeres 1–7 black, 8–11 yellowish; pronotum with black triangular macula just behind head; elytra with basal and apical ⅓ black; tarsi black, tibiae black with pale apex, femora with black base and apex; venter with pro-, meso-, and metasterna yellowish-brown medially, dark laterally, abdomen diffuse black with yellow laterally. Head: vertex with V-shaped sulcus, sparsely and irregularly punctate; keel present between antennal bases; frons punctate, not projecting; not depressed between eyes. Antenna: reaches to humerus; slender; male with antennomere 1 thickened apically with a tuft of setae at apex, 2 with a rounded apical angle, 3 expanded triangular, apex obliquely truncate; female with antennomere 1 slender with tuft of setae at apex, 3 having a pointed inner apical angle; 4–10 cylindrical, decreasing in length; 11 2× length of 10, bluntly rounded at apex; 1–2 punctate; 3–11 setose. Pronotum: transverse; lateral margin straight for basal ¾ then rounding to anterior angle, canaliculate; anterior angle rounded, not produced; posterior angle acute; anterior margin straight; disc subconvex; surface sparsely, irregularly punctate laterally and basally; basal impression absent; pronotal length 1.4–1.7 mm; pronotal width 1.6–2.0 mm. Scutellum: pentagonal; punctate. Elytron: lateral margin straight, smooth, margined; apex rounded, apical margin slightly laminate; sutural angle without tooth; humerus rounded, slightly produced; slightly constricted behind humerus; shallowly punctate-striate; elytral length 5.8–6.2 mm; elytral width 2.3–2.6 mm. Venter: pro-, meso-, and metasterna impunctate medially, punctate laterally, each puncture with pale seta; abdominal sterna punctate, each puncture with pale seta; suture between sterna 1 and 2 complete; last sternite with apical margin emarginate medially in male, weakly rounded medially in female. Leg: slender; punctate; femur and tibia with seta in each puncture; tibia with fringe of setae on inner margin of apex. Total length: 7.8–8.2 mm.

##### Diagnosis.

This species is similar to *Cephaloleia nubila*. It can be distinguished by the pro-, meso-, and metasterna being punctate laterally and by antennomeres 4 to 10 decreasing in length.

##### Distribution.

Ecuador.

##### Type material.

Type: Ecuador, Cachabé, MNHN, not examined.

##### Specimens examined.

**Ecuador:** no further data (BMHN). Esmeraldas- 31.7 km NW Lita, 620 m, 23 August 1997 (USNM). Imbabura- Cachabé (MNHN). Total: 7.

#### Cephaloleia approximata

Taxon classificationAnimaliaColeopteraChrysomelidae

Baly, 1869

http://species-id.net/wiki/Cephaloleia_approximata

Fig. 79

Cephalolia approximata Baly 1869: 367. Gemminger and Harold 1876: 3601 (catalog); Donckier 1899: 548 (catalog); Weise 1911a: 7 (catalog), 1911b: 11 (catalog), 1921b: 174 (noted).
Cephaloleia approximata Baly. Uhmann 1957b: 15 (catalog); Staines and Staines 1999: 523 (Baly species list).

##### Description.

Elongate; subdepressed; shining; reddish-brown; eyes and antennomeres 6–11 darker; legs yellowish. Head: vertex punctate, medial carina present; frons impunctate, not projecting; depressed between eyes. Antenna: longer than head and pronotum combined; robust; antennomere 1 large, slightly incrassate; 2 elongate, shorter than 1, subequal in length to 3; 3–10 decreasing in length; 11 bluntly pointed at apex; 1–2 punctate; 3–11 setose. Pronotum: transverse; lateral margin straight and diverging for basal ¾ then rounding to anterior angle, slightly laminate; anterior angle with rounded tooth, projecting; posterior angle acute; anterior margin emarginate behind head; disc subconvex; surface sparsely, coarsely punctate, less punctate on disc; slight transverse basal impression present medially; pronotal length 1.4–1.7 mm; pronotal width 2.0–2.4 mm. Scutellum: triangular; impunctate. Elytron: lateral margin slightly expanding apically, smooth, margined; apex rounded, apical margin smooth; sutural angle without tooth; humerus rounded, not produced; slightly constricted behind humerus; shallowly punctate-striate, punctures confused apically; striae on apical half slightly sulcate; elytral length 5.4–5.9 mm; elytral width 2.9–3.0 mm. Venter: pro-, meso-, and metasterna impunctate medially, punctate laterally; abdominal sterna punctate, each puncture with pale seta; suture between abdominal sterna 1 and 2 complete; female with apical margin of last sternite bisinuate. Leg: slender; punctate; femur and tibia with seta in each puncture; tibia with fringe of setae at apex. Total length: 7.2–7.6 mm.

##### Diagnosis.

This species is most similar to *Cephaloleia bucki*. It can be distinguished by the medial carina on the vertex of the head and by the elytra being slightly costate apically.

##### Distribution.

Brazil, Peru.

##### Type material examined.

Holotype: Upper Amazons [handwritten label]/ Baly Coll. [printed label]/ Cephalolia approximata Baly, Upper Amazons [blue handwritten label] (BMNH).

#### Cephaloleia atriceps

Taxon classificationAnimaliaColeopteraChrysomelidae

Pic, 1926b

http://species-id.net/wiki/Cephaloleia_atriceps

Fig. 80

Cephaloleia atriceps Pic 1926b: 238. Uhmann 1930a: 227 (faunal list), 1930b: 137 (comparative note).
Cephalolia atriceps Pic. Blackwelder 1946: 718 (catalog); Papp 1953: 14 (catalog); Uhmann 1957a: 16 (catalog), 1964a: 402 (catalog); Descarpentries and Villiers 1959a: 139 (types); Wilcox 1983: 136 (catalog); Staines 1996: 17 (Central America species), 2011: 48 (faunal list).

##### Description.

Elongate; subdepressed; subparallel; black; pronotum, prosternum, and claws red. Head: vertex densely punctate, medial sulcus absent; keel present between antennal bases; frons not projecting; not depressed between eyes. Antenna: reaches beyond humerus; slender; antennomeres similar to each other; antennomere 1 transverse, slightly longer than 2; 2 transverse; 3 longer than 1; 4–10 transverse, decreasing in length, each shorter than 3; 11 2× length of 10, rounded at apex; 1–3 punctate with scattered setae; 4–11 setose. Pronotum: transverse; lateral margin sinuate then rounding to anterior angle, canaliculate; anterior angle rounded, slightly produced; posterior angle acute; apical margin straight; disc subconvex; disc with scattered, oval punctures, large medial area impunctate; basal impression present medially; pronotal length 0.9 mm; pronotal width 1.0 mm. Scutellum: triangular; impunctate. Elytron: lateral margin almost straight, margined, finely toothed; apex rounded; sutural angle with tooth; humerus rounded, not produced; slightly constricted behind humerus; puncture rows converging and uniting apically, except rows 1 and 10; rows 2–5 weakly punctate beyond middle; nearly impunctate at humerus; elytral length 2.7–3.1 mm; elytral width 1.4 mm. Venter: epipleuron wide, impunctate; prosternum punctate; meso- and metasterna impunctate medially, punctate laterally; abdominal sterna 1–3 punctate laterally; 4–5 setose; suture between sterna 1 and 2 complete; last sternite with apical margin broadly emarginate medially in male, truncate in female; pygidium keeled. Leg: slender; femur punctate each puncture with pale seta; tibia with row of setae on inner margin and fringe of setae at apex, punctate. Total length: 4.0–4.4 mm.

**Figures 80–88.**
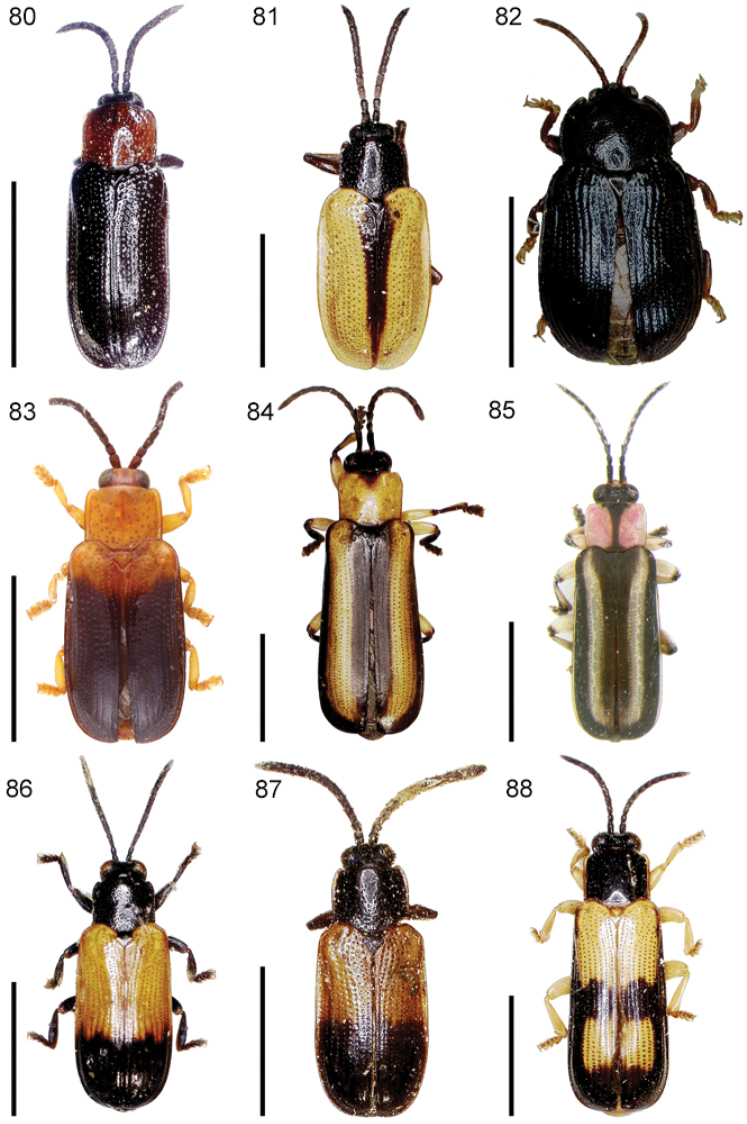
Habitus. **80** *Cephaloleia atriceps* **81** *Cephaloleia balyi* **82** *Cephaloleia barroi* **83** *Cephaloleia basalis* **84** *Cephaloleia bella* **85** *Cephaloleia belti* **86** *Cephaloleia bicolor* **87** *Cephaloleia bicoloripes* **88** *Cephaloleia bifasciata*. Scale bars equal 3 mm.

##### Diagnosis.

This species is most similar to *Cephaloleia ruficollis* and *Cephaloleia schmidti*. It can be distinguished by the unicolorous elytra and by the vertex of the head not being depressed between the eyes.

##### Distribution.

Costa Rica, Mexico.

##### Type material examined.

Holotype: Turrialba, Costa Rica [handwritten label]/ type [yellow label]/ TYPE [red label]/ Museum Paris, Coll. M. Pic [blue printed label]/ atriceps sp. n. [handwritten label] (MNHN).

##### Specimens examined.

**COSTA RICA:** no further data (MUCR). Alajuela- Fca. San Gabriel, 2 km SO de Dos Ríos, 600 m, 18 August 1991, 28 May 1991, May 1990 (INBIO); Peñas Blancas, August 1987, 7 July 1987 (USNM); 20 km S Upala, 4 September 1990, 19 February 1991, 13–18 March 1989, 22 July 1991, 11–21 September 1991, 1–9 May 1991 (BYUC). Cartago- Ref. Nac. Fauna Silv. Tapantí, 1150 m, January 1992 (INBIO); Turrialba (MNHN). Guanacaste- Est. Cacao, 1000–1400 m, Lado SO Vol. Cacao, P.N. Guan., 21–29 May 1992, July 1992 (INBIO); Est. Pitilla, 700 m, 9 km S Sta. Cecilia, P. N. Guanacaste, November 1989 (INBIO); Río San Lorenzo, 1050, Tierras Morenas, Z. P. Tenorio, July 1992 (INBIO); Finca YAFA, 200–300 m (INBIO); A. C. A. La Cruz, La Garita, Est Los Almendros, 200–300 m (INBIO); 3 km SE Río Naranjo, 5–9 July 1993, 13–18 March 1993, 8–15 June 1992, 17–19 July 1993, 1–15 June 1993 (BYUC). Heredia- 11 km SE La Virgen, 450–550 m, 12 April 2003 (USNM). Limón- Amubri (Talamanca), 26 July 1975 (BYUC); 8 km SW Guacimo, 17 May 1992 (AJGC); Hamburg Farm, Reventazón, Ebene Limón, 28 July 1932, 2 July 1929, 27 July 1930, 4 January 1925 (USNM), 1 June 1929, 27 July 1930 (DEI); Est. Jalova, 0 m, P. N. Tortuguero, July 1990 (INBIO); Salvadora Farm, Parismina, 30 May 1932 (USNM); A. C. Llanuras del Tortuguero, Pococí, Río Sardinas, Barra del Colorado (INBIO). Puntarenas- Monte Verde, 6 January 1974 (TAMU), 20–24 June 1986 (BYUC); Reserva de Monteverde, 27–29 May 1979 (CNC); Finca Las Cruces, 6 km. S. San Vito de Java, 4200 ft., 28 September- 2 October 1986 (FSCA); Buenos Aires, Sector Altamira, Biolley, 1700–1800 m (INBIO); Estació Pittier, 4.2 km SW Cerro Gemelo, 1600–1700 m (INBIO); Sendero a Cerro Pittier, 1 km N de la Estación, 1900–2000 m (INBIO). San José- Pan American Hwy, km 117, 19 km N San Isidro, 20–25 June 1997 (SEMC). **MEXICO:** Chiapas- Amatenango del Valle, 2134 m, 20 August 1972 (CASC); Palenque, 10 September 1974 (BYUC). Nayarit- 25 mi N. Tepic, 24 April 1961 (CNC). San Luis Potosí- Tamazunchale, 4 January 1941 (CASC), 20 July 1956 (AMNH). Total: 61.

#### Cephaloleia balyi

Taxon classificationAnimaliaColeopteraChrysomelidae

Duvivier, 1890

http://species-id.net/wiki/Cephaloleia_balyi

Fig. 81

Cephalolia balyi Duvivier 1890: xxxviii. Donckier 1899: 548 (catalog); Weise 1911a: 7 (catalog), 1911b: 12 (catalog).
Cephaloleia balyi Duvivier. Uhmann 1957b: 16 (catalog), 1959c: 621 (noted), 1964a: 402 (catalog).

##### Description.

Oval; subdepressed; shining; testaceous; legs yellow, darker at tibio-femoral joint; antennomeres 1–2 black, 3–11 brown; head, meso-, and metasterna black; pronotum brownish-black; scutellum testaceous; elytra yellowish with black vitta from scutellum extending down suture ⅔ length of elytra; venter testaceous, abdominal sternite 1 darker. Head: vertex strongly, densely punctate, without medial sulcus; projection present between antennal bases; frons not projecting; depressed between eyes. Antenna: slightly longer than head and pronotum combined; slender; antennomeres 1–2 robust, cylindrical; 3–4 cylindrical, subequal in length, each shorter than 2; 5–10 transverse, decreasing in length, each shorter than 4; 11 longer than 10, acutely pointed at apex; 1–2 strongly punctate with scattered setae; 3–11 setose. Pronotum: transverse; lateral margin straight then rounding to anterior angle, slightly canaliculate; anterior angle obtuse, slightly produced; posterior angle acute; anterior margin emarginate behind head; disc subconvex; with smooth longitudinal band medially; surface irregularly punctate, less punctate laterally; basal impression absent; pronotal length 1.2–1.4 mm; pronotal width 1.5–1.7 mm. Scutellum: pentagonal; impunctate. Elytron: lateral margin straight, smooth, slightly laminate; apex rounded; sutural angle without tooth; humerus rounded, not produced; slightly constricted behind humerus; shallowly punctate-striate; elytral length 3.7–4.1 mm; elytral width 2.1–2.5 mm. Venter: pro- and mesosterna densely punctate; metasternum impunctate medially, punctate laterally; abdominal sterna punctate, each puncture with pale seta; suture between sterna 1 and 2 complete; apical margin of last sternite emarginate medially in male, rounded in female. Leg: short; robust; punctate, each puncture with pale seta; tibia with fringe of setae on inner margin of apex. Total length: 5.3–5.6 mm.

##### Diagnosis.

This species is similar to *Cephaloleia deficiens*, *Cephaloleia discoidalis*, *Cephaloleia dorsalis*, *Cephaloleia linkei*, and *Cephaloleia suturalis*. It can be distinguished by the dark pronotum and by antennomeres 1 and 2 being robust.

##### Distribution.

Ecuador.

##### Type material.

Type: America Meridional, ISNB, not seen.

##### Specimens examined.

**Ecuador:** Bolivar- Guaranda-Caluma Hwy., Río Pita, 1289 m, 1 November 2008 (USNM). Pichincha- Chimba, 1891 (USNM). Total: 4.

#### Cephaloleia barroi

Taxon classificationAnimaliaColeopteraChrysomelidae

Uhmann, 1959a

http://species-id.net/wiki/Cephaloleia_barroi

Fig. 82

Cephaloleia barroi Uhmann 1959a: 617. Uhmann 1964a: 402 (catalog); Sanderson 1967: 137 (noted); Gaedike and Döbler 1971: 343 (types); Staines 1996: 17 (Central America species), 1997: 413 (Uhmann species list), 1999: 242 (mimicry), 2008: 1 (key), 2009a: 21 (noted); Piña et al. 2004: 106 (faunal list).

##### Description.

Oval; convex; bright metallic blue; antennae and legs yellow; venter black. Head: vertex densely punctate, medial sulcus absent; frons not projecting; not depressed between eyes. Antenna: reaches to hind margin of pronotum; slender; antennomeres similar in appearance; 1–2 subequal in length, transverse; 3 elongate, as long as 1–2 combined; 4–10 transverse, subequal in length, each shorter than 3; 11 pointed at apex, as long as 3; 1–4 punctate with scattered setae; 5–11 setose. Pronotum: transverse; lateral margin almost straight on basal ⅓, then rounding to anterior angle, strongly margined; anterior angle projecting, narrow; posterior angle angulate; anterior margin emarginate behind head; disc convex; surface finely and moderately punctate; weak impression present on each side; basal impression absent; pronotal length 1.0 mm; pronotal width 1.8 mm. Scutellum: triangular; impunctate. Elytron: lateral margin smooth, finely margined; apex rounded; sutural angle without tooth; humerus rounded, not produced; slightly constricted behind humerus; convex; with fine, dense punctures; scutellar row long; puncture rows converge and unite on apex; interspaces convex; elytral length 3.7 mm; elytral width 2.5 mm. Venter: pro-, meso-, and metasterna punctate; abdominal sterna finely punctate, each puncture with short setae; last sternite with apical margin rounded, weakly emarginate on each side in female; pygidium broadly rounded, finely punctate; last sternite with apical margin shallowly emarginate in male, weakly rounded in female. Leg: slender; protibia with longitudinal groove beneath; tarsi and apex of tibia bright brown; femur robust, punctate; tibia incised at apex, with fringe of setae on inner margin of apex. Total length: 4.8 mm.

##### Diagnosis.

This species is similar to *Cephaloleia sandersoni*. It can be distinguished by the densely punctate vertex of the head, by the evenly arcuate lateral margins of the pronotum, and by antennomere 1 being clavate and twice the length of 2.

##### Distribution.

Cuba.

##### Type material.

Holotype female: Cuba, Lomas de Trinidad [green label]/ Santa Clara, 12.VIII.1939 [green label]/ 7–6/ Holotypus [red label]/ Cephaloleia barroi Uh., Uhmann det 58 (DEI).

##### Specimens examined.

**CUBA:** Lomas de Trinidad, Santa Clara, 12 August 1939 (USNM). Total: 2.

#### Cephaloleia basalis

Taxon classificationAnimaliaColeopteraChrysomelidae

Pic, 1926e

http://species-id.net/wiki/Cephaloleia_basalis

Fig. 83

Cephalolia basalis Pic 1926e: 359.
Cephaloleia basalis Pic. Uhmann 1957b: 16 (catalog); Descarpentries and Villiers 1959a: 139 (types).

##### Description.

Oblong; shining; flattened; testaceous; elytra black with base reddish; antennae black with antennomeres 1–2 reddish; legs yellowish. Head: vertex sparsely punctate, medial sulcus present; eye slightly convex; frons not projecting; not depressed between eyes. Antenna: longer than head and pronotum combined; slender; antennomere 1 incrassate; 2–4 elongate; 2 ½ length 1; 3 longer than 2; 3–4 subequal in length; 5–10 transverse, decreasing in length, each shorter than 4; 11 missing; 1–2 punctate, with scattered setae; 3–10 setose. Pronotum: subquadrate; lateral margin straight then rounding to anterior angle, canaliculate; anterior angle rounded, slightly produced; posterior angle acute; anterior margin emarginate behind head; basal margin biangulate; disc flattened; surface strongly, sparsely punctate; basal impression absent; pronotal length 1.2 mm; pronotal width 1.4 mm. Scutellum: pentagonal; impunctate. Elytron: lateral margin straight, smooth, moderately margined; apex rounded; sutural angle without tooth; humerus rounded, not produced; slightly constricted behind humerus; subconvex; strongly punctate-striate; elytral length 3.6 mm; elytral width 2.0 mm. Venter: obscured by card. Leg: slender; punctate. Total length: 5.0 mm.

##### Diagnosis.

This species is most similar to *Cephaloleia waterhousei*. It can be distinguished by the elytral puncture rows being distinct to apex and by antennomere 2 being longer than 3.

##### Distribution.

Brazil (Espírito Santo).

##### Type material examined.

Holotype: Brazil [printed label]/basalis n. sp [handwritten label]/ Museum Paris Coll. M. Pic [blue printed label]/ Type [red printed label]/ Cephaloleia basalis Pic [printed label]/ Holotype [red printed label]/ MNHN EC 2645 [printed label] (MNHN).

#### Cephaloleia bella

Taxon classificationAnimaliaColeopteraChrysomelidae

Baly, 1885

http://species-id.net/wiki/Cephaloleia_bella

Fig. 84

Cephaloleia bella Baly 1885: 20. Blackwelder 1946: 718 (catalog); Papp 1953: 14 (catalog); Uhmann 1957a: 16 (catalog), 1959a: 621 (noted), 1964a: 402 (catalog); Gaedike and Döbler 1971: 343 (types); Wilcox 1983: 136 (catalog); Staines 1996: 18 (Central America species), 1999: 241 (mimicry), 2004: 312 (host plants), 2010: 27 (types), 2011: 48 (faunal list); Staines and Staines 1997: 4 (types), 1999: 523 (Baly species list); McKenna and Farrell 2005: 119 (phylogeny), 2006: 10949 (phylogeny); García–Robledo et al. 2013a: 3 (biology).
Cephalolia bella Baly. Donckier 1899: 548 (catalog); Weise 1905a: 132 (noted), 1911a: 7 (catalog), 1911b: 10 (catalog), 1921a: 263 (noted); Uhmann 1930a: 233 (key), 1936b: 483 (key).

##### Description.

Elongate; flattened; subparallel; head (except yellow frons), antennae, and scutellum black; pronotum yellow with black medial macula on anterior and basal margins; elytra black with yellow vitta which begins at interspace 4 and extends to interspace 7 from humerus and extends to near apical margin, lateral margin black; venter with prosternum yellow with black macula beyond coxae; mesosternum yellow medially, black laterally; metasternum black; abdominal sterna yellowish-orange with black vitta on lateral margin; leg with femur yellow with black ring at apex; tibia and tarsi black. Head: vertex punctate, with V-shaped sulcus between eyes; eyes slightly protruding, finely faceted; frons punctate, not projecting; depressed between eyes. Antenna: reaches to humerus; slender; antennomere 1 elongate, robust, compressed; 2 transverse, ⅓ length of 1; 3–4 elongate, projection on inner angle, 3 1½ x length of 2; 4 shorter than 3; 5–10 transverse, subequal in length, each shorter than 4; 11 2× length of 10, pointed at apex; 1–3 punctate with scattered setae; 4–11 setose. Pronotum: transverse; lateral margin straight then rounding to anterior angle, margined; anterior angle rounded, slightly produced; posterior angle acute; anterior margin straight; disc flattened; surface impunctate; basal impression absent; pronotal length 1.4–1.7 mm; pronotal width 1.7–1.9 mm. Scutellum: broadly triangular; alutaceous. Elytron: lateral margin straight, narrowly margined, smooth; apex rounded; sutural angle with minute tooth; humerus rounded, not produced; slightly constricted behind humerus; declivity beginning just behind humerus at puncture row 7 edged with faint carina; moderately punctate-striate; punctures in vitta larger than those on rest of elytron; elytral length 5.6–6.0 mm; elytral width 2.6–2.8 mm. Venter: pro-, meso-, and metasterna impunctate medially, punctate laterally, each puncture with pale seta; abdominal sterna punctate, each puncture with seta; suture between sterna 1 and 2 complete; last sternite with apical margin emarginate medially in male, truncate in female. Leg: sparsely punctate; femur robust; tibia with fringe of setae on inner margin of apex. Total length: 7.6–8.1 mm.

##### Diagnosis.

This species is similar to *Cephaloleia championi*, *Cephaloleia luctuosa*, and *Cephaloleia vicina*. It can be distinguished by the head being depressed between the eyes, by the impunctate pronotum, by antennomeres 3 and 4 being triangular, and by the larger punctures in the elytral vitta.

##### Comments.

Preliminary analysis of the CO1 gene indicates that cryptic species may be present under the current application of this species name. Further work is needed to resolve this question.

##### Host plant.

Adults have been collected on *Heliconia imbricata* (Kuntze) Baker (Heliconiaceae) (Staines 1996).

##### Distribution.

Colombia, Costa Rica, Mexico, Panama.

##### Type material examined.

Syntypes: Bugaba, 800–1500 ft., Champion/ Cephaloleia bella Baly (USNM, 4; AMNH, 4; ANSP, 2).

##### Specimens examined.

**COSTA RICA:** Cartago- Quebrada Segunda, Ref. Nac. Fauna Silv., Tapantí, 1250 m, April 1992, January 1992 (INBIO); Río Grande de Orosí, Puente Río Dos Amigos, 1550–1600 m (INBIO). Guanacaste- Est. Pitilla, 700 m, 9 km S Sta. Cecilia, P. N. Guanacaste, July 1991 (INBIO). Heredia- Finca La Selva nr. Puerto Viejo, 22 June 1969, 24 July 1969 (USNM), 20–23 June 2001 (USNM); Hamburg Farm, Reventazón, 16 January 1936 (USNM). Limón- Bataan, 16 June 1951 (USNM); Est. Hitoy-Cerere, 100 m, November 1991, 12 April 1992, 30 July 1992, June 1991, 27 June- 22 July (INBIO); Finca Hamburgo, 31 January 1931, 1 February 1932, 31 March 1931 (MUCR); Puerto Vargas, 7 December 1963 (MUCR); Valle La Estrella, R. B. Hitoy Cerere, 100–200 m (INBIO); Pococí Colorado, Sec Cerro Cocorí, 30 km N Cariari, 100–200 m (INBIO); Amubri, Sendero Soki, 0–100 m (INBIO). Puntarenas- Barranca site, 10 km N Puntarenas, 11 September 1969 (USNM); Corcovado, 13 December 1984 (INBIO); Est. Sirena, Corcovado NP, October 1989, November 1989, December 1989, January 1990, February 1990, March 1990, April 1990, October 1990, December 1990, June 1991, August 1991, September 1991, November 1991, January 1992, March 1992, 21 March- 21 April 1992, April 1992, June 1992, May 1992, November 1992, (INBIO), August 1993 (MUCR); Golfo Dulce, Río Salendato, 21 August 1936 (USNM); Puerto Cortes, 19 July 1972 (FSCA); Osa Peninsula, 0.8 mi SW Rincón, 25 July 1968 (CMNC); Osa Peninsula, 3.5 mi. S. Rincón, 1 March 1969 (USNM); Osa Peninsula, 5.0 mi SW Rincón, 31 August 1968 (INBIO, USNM); Peninsula de Osa, 31 July 1968 (MUCR); 2 mi. S. Palmar Sur, 19 August 1969 (USNM); 5.4 mi S Palmar Sur, 11 August 1969 (USNM); Rancho Quemado, Pen. Osa, February 1991, April 1991, July 1991, September 1992, November 1992 (INBIO); Río Piedras, sea level, 15 August 1969 (USNM); from Villa Neilly to Río Claro, 21 July 1972 (FSCA); Sierpe, 2.5 mi SW Rincón (INBIO). San José- Finca La Caja, La Uruea, 1200 m, 14 July 1931 (MUCR). **MEXICO:** Oaxaca- 16 mi. N of Jachitanm 6 July 1955 (SEMC). Veracruz- 8 km. ne. Sontecomapan, 20 July 1980 (TAMU). **PANAMA:** Chiriquí- Bugaba, 800–1500 ft. (DEI). Colón- Frijoles (USNM); Paraiso, 25 January 1911, 15 March 1911, 26 March 1911 (USNM); Porto Bello, 7 March 1911, 16 February 1911, 26 February 1911 (USNM). Panamá- Alajuelo, 2 March 1911, 5 April 1911, 15 April 1911, 18 April 1911 (USNM). Total: 536.

#### Cephaloleia belti

Taxon classificationAnimaliaColeopteraChrysomelidae

Baly, 1885

http://species-id.net/wiki/Cephaloleia_belti

Fig. 85

Cephaloleia belti Baly 1885: 22. Maulik 1932: 97 (host plant), 1937: 132 (host plants); Blackwelder 1946: 718 (catalog); Papp 1953: 14 (catalog); Uhmann 1957a: 16 (catalog); Wilcox 1983: 136 (catalog); Maes and Staines 1991: 36 (faunal list); Staines 1996: 19 (Central America species), 1996(1997): 14 (Nicaragua species), 1999: 241 (mimicry), 2004: 312 (host plants), 2011: 48 (faunal list); Maes 1999: 1016 (faunal list); Staines and Staines 1999: 523 (Baly species list); McKenna and Farrell 2005: 119 (phylogeny); Meskins et al. 2008: 163 (host plants), 2011: 483 (food web); Descampe et al. 2008: 227 (host plants); García–Robledo et al. 2010: 51 (larva, biology); 2013a: 3 (biology), 2013b: 193 (biology); García–Robledo and Horvitz 2011: 978 (biology), 2012: 40 (biology); Lawrence et al. 2011: 13 (nomenclature); Barrett and Heil 2012: 283 (noted); Schmitt and Frank 2013: 58 (biology); Sekerka et al. 2013: 304 (noted).
Cephalolia belti Baly. Donckier 1899: 548 (catalog); Weise 1911a: 7 (catalog), 1911b: 10 (catalog); Uhmann 1930a: 223 (faunal list), 1936a: 111 (noted), 1936b: 485 (key).
Cephaloleia consanguinea sensu Strong 1977a: 160 (misidentification). Strong 1977b: 573 (host plants), 1981: 185 (host plants), 1982a: 218 (host plants), 1982b: 1041 (host plants); Morrison and Strong 1981: 56 (host plants); Auerbach and Strong 1981: 64 (host plants); McCoy 1984: 10 (biology), 1985: 326 (biology); Grégoire 1988: 254 (noted); Jolivet 1997: 146 (noted); Jolivet and Verma 2002: 63 (noted); Staines 2004: 312 (identification).

##### Description.

Elongate; flattened; subparallel; head (except yellow frons), antennae, and scutellum black; pronotum yellowish with medial black vitta, vitta may be obsolete medially; elytra from suture to puncture row 2 black, then through puncture row 5 yellow, then black through puncture row 9, puncture row 10 to margin yellow, sutural vitta narrows and fades toward apex, fails to reach apex; venter with pro-, meso-, and metasterna yellow medially, black laterally, abdominal sternite 1 yellow medially, black laterally, sterna 2 and 3 black laterally, each has black vitta along apex, rest yellow, sterna 4 and 5 all black; leg femora yellow each dark apex, tibiae and tarsi dark. Head: vertex sparsely punctate, medial carina present; frons not projecting; eyes protuberant, finely faceted; not depressed between eyes. Antenna: reaches to humerus; slender; antennomere 1 elongate, as long as 2–4 combined, robust, tuft of setae at apex; 2–3 compressed laterally; 2 transverse; 3 triangular, longer than 2; 4–6 transverse, subequal in length to 3; 7–10 transverse, subequal in length, each shorter than 6; 11 2× length of 10, pointed at apex; 1–3 punctate with scattered setae; 4–11 setose. Pronotum: transverse; lateral margin straight then rounding to anterior angle, narrowly margined; anterior angle rounded; posterior angle acute; anterior margin straight; disc flattened; surface with scattered large punctures laterally, disc impunctate; basal impression absent; pronotal length 1.1–1.2 mm; pronotal width 1.3–1.5 mm. Scutellum: sharply triangular; micropunctate. Elytron: lateral margin straight, smooth, margined; apex rounded; sutural angle with small tooth; humerus rounded, not produced; slightly constricted behind humerus; weakly punctate-striate; elytral length 4.8–4.9 mm; elytral width 1.9–2.0 mm. Venter: pro-, meso-, and metasterna impunctate medially, punctate laterally, each puncture with pale seta; abdominal sterna punctate, each puncture with pale seta; suture between sterna 1 and 2 complete; last sternite with apical margin u-shaped in male, slightly acuminate in female. Leg: punctate; femur robust; tibia with fringe of setae on inner margin of apex. Total length: 6.4–6.56 mm; females are larger than males.

##### Diagnosis.

This species is similar to *Cephaloleia consanguinea*, *Cephaloleia erugatus*, *Cephaloleia semivittata*, *Cephaloleia triangularis*, *Cephaloleia trivittata*, and *Cephaloleia vittata*. It can be distinguished by the elytral puncture rows being distinct apically, by antennomere 1 being as long as 2 to 4 combined, and by the sutural angle with a small tooth.

##### Host plant.

*Calathea insignis* Hort. and Bull. (Marantaceae) (Uhmann 1930a); *Heliconia imbricata* (Kuntze) Baker (Maulik 1932); *Heliconia latispatha* Bentham (Strong 1977b), *Heliconia pogonantha* Cuford., *Heliconia mariae* Hook., *Heliconia tortuosa* Griggs (Strong 1982a) (Heliconiaceae); *Calathea latifolia* Klotzsch, *Cephaloleia lutea* Schult., *Heliconia catheta* R. R. Smith, *Heliconia irrasa* R. R. Smith (Meskins et al. 2008); *Heliconia vaginalis* Benth., *Heliconia wagneriana* Peterson (Descampe et al. 2008); *Heliconia mathiasiae* G. S. Daniels and F. G. Stiles, *Cephaloleia cleistantha* Standl., *Cephaloleia crotalifera* S. Watson, *Cephaloleia marantifolia* Standl., *Cephaloleia similis* H. Kenn., *Canna tuerckheimii* Kraenzl. (Cannaceae) (García–Robledo et al. 2013a); *Costus barbatus* Suess. (Costaceae), *Goeppertia lasiophylla* (H. Kenn.) Borchs. & S. Suárez, *Heliconia denielsiana* Kress, *Heliconia densiflora* B. Verl., *Heliconia longiflora* R. R. Sm., *Heliconia rostrata* Ruiz. & Pav., *Heliconia stricta* Huber (Heliconiaceae), *Musa paradisiaca* L. (Museceae) (Schmitt and Frank 2013); *Heliconia psittacorum* Sw., *Heliconia sarapiquensis* G. S. Daniels and F. G. Stiles, *Calathea* sp., *Musa velutina* H. Wendl. and Drude (Musaceae), *Ischnosiphon inflatus* L. Andersson, *Pleiostachya pruinosa* (W. Bull. ex. Regel) K. Schum. (Marantaceae).

##### Immatures.

Color when live (Figs 27–30) is yellowish-brown with outer margins translucent; center portion reddish with some yellowish areas especially near head. Color when dead is pale-brown centrally, margins paler becoming almost transparent at edge; venter paler than dorsum. Dorsum carries a longitudinal medial setose ridge extending from anterior to posterior margin. Pronotum with central area raised, micropunctate, with dark setae on either side of medial longitudinal ridge and on basal slope; lateral areas rugose; two diagonal carinae on central raised area extending to anterior margin. Mesonotum with central raised portion with shallow v-shaped carina; laterally with sharply curved carina which extends to lateral margin. Metanotum with central portion irregularly plicate; with transverse carina extending across entire width. Abdominal tergites 1–6 slightly narrowed medially, wider at sides; with transverse carina medially of each side; spiracle near basal margin on each side just off central elevation; spiracles appear as macula with dark margin. Abdominal tergites 7–10 with surface plicate; with three carinae along margin on each side. Venter with surface of expansions punctate, rugose-striate. Head surface rugose-punctate; labrum with surface alutaceous, without setae; clypeus with fringe of long setae at apex, with four setae on apical ½, surface alutaceous; mandibles tridentate; maxillary palps with 2 palpomeres and 12 short, robust setae at apex; maxilla robust, clavate, with fringe of long setae at apex; labium densely setose. Antenna with antennomere 1 short, robust; 2 wider than 1, transverse; 3 elongate, cylindrical, subequal in length to 1 and 2 combined, with fringe of short setae at apex. Pro- and mesosterna wider than long; slightly depressed medially; surface rugose-striate. Metasternum longer than others; depressed medially; with suture along apical margin. Abdominal sternites 1–7 wider than long; decreasing in width; with three sulci on apical ½; laterally with curved sulcus dividing the sternite into thirds; sternite 8 similar to preceding but without any sulci; sterna 9–10 fused, rounded at apex. Leg: femur wider and shorter than tibiotarsus; tibiotarsus subconical, with a strong claw and eight setae at apex. Total length: 6.7 mm; width 4.3 mm. (García–Robledo et al. 2010).

##### Biology.

From Strong 1977a, 1977b, 1981, 1982a, 1982b; Morrison and Strong 1981; Auerbach and Strong 1981, and García–Robledo et al. 2010. Eggs are about 2 mm long and are laid singly or in clusters of two or more on host plant petioles or rolled leaves and are covered with frass. Eggs hatch in 6.5 to 7.5 days. The larvae have two instars, the first lasting 10 to 14 days and the second, 32 to 46 days. The pupal stage lasts from 15 to 17 days. Adults live about 117 days.

##### Distribution.

Costa Rica, Guatemala, Honduras, Mexico, Nicaragua, Panama

##### Type material examined.

Lectotype: Type H. T. [white disk with red border]/ Chontales, Nicaragua. Janson [handwritten]/ B. C. A., Col. VI, 2. Cephaloleia belti, Baly [printed label]/ Cephaloleia belti Baly, Nicaragua [blue handwritten label]/ Lectotype Cephaloleia belti Baly Des. C. L. Staines 1993 [red label] (BMNH).

##### Specimens examined.

**COSTA RICA:** Alajuela- Bijagua, Alberge de Heliconicas, 1000–1100 m, 18 June 2000 (USNM); Caño Negro, 20 m, R.N.V.S., Caño Negro, 4–15 December 1992 (INBIO); Garita, 8 January 1995 (USNM); Río Frío, E. La Selva, Sarapiquí, June 1972, 16 July 1972 (FSCA); Río San Lorencito, 900 m, R. F. San Ramón, 5 km N de Colonia Palmareña, 13–18 June 1993 (INBIO); E. B. San Ramón, R. B. San Ramón, 27 km N and 8 km W. San Ramón, 810 m, 7 July 2000 (SEMC, USNM); Upala, 1 November 1989 (MUCR); 20 km S Upala, 16–25 September 1990, 1–5 October 1990, 13 December 1990- 9 January 1991, 10–21 May 1991, 1–11 June 1991, 21 June 1991, 1–15 July 1991, 21–31 August 1991, (BYUC); San Ramón, Angles, Reserva Biol. Alberto Brenes, 1000–1100 m (INBIO); San Ramón, Estación Eladios, 700–800 m (INBIO); Upala, Sector San Ramón de Dos Ríos, 1.5 km NW Hacienda Nueva Zelandia, 600–700 m (INBIO). Cartago- Aquiares nr. Santa Cruz, 9 km NE Turrialba, 1500 m, 16 May 1985 (EMEC); CATIE, 3 km SE Turrialba, 600 m, 16 May 1985 (EMEC); El Guarco, San Isidro, 4 km S del Cañon, 2200–2300 m (INBIO); La Palma, 30 April 1928 (USNM); Quebrada Segunda, 1200–1300 m (INBIO); Rancho Naturalista, 2 km NE Tula, 2500 ft., 15–16 June 1995 (BYUC); Río Grande de Orosí, desde La Catarata, 1500–1600 m (INBIO); 19.3 km NE San José, 17 May 1993 (SEMC); Turrialba, no further data (DEI), 13–17 March 1965 (USNM), 650 m., 4–13 August 1970 (USNM), 17 May 1979 (CMNC); 40 km NE Turrialba, 18 May 1979 (CMNC); Turrialba, Santa Teresita, Monumento Nacional Guayabo, 1100–1200 m (INBIO); IICA at Turrialba, 13 March 1965 (BYUC). Guanacaste- Est. Cacao, 1000–1400 m, Lado SO Vol. Cacao, P.N., 21–29 May 1992 (INBIO); Est. Pitilla, 700 m, 9 km S Sta. Cecilia, 1989, January 1989, 27 January- 4 February 1989, March 1989, November 1989, December 1989, March 1990, November 1990, March 1991, April 1991, May 1991, July 1991, September 1991, 4–14 November 1991, 2–19 March 1992, 31 March- 29 April 1992, May 1992, 24 August- 11 September 1992, 12 September 1992 (INBIO); Liberia, Mayorga, Estación Cacao, 2 km SW Cerro Cacao, 900–1000 m (INBIO); 3 km SE Río Naranjo, 16–29 February 1992, 15–20 October 1992, 12 June 1993 (BYUC); Río San Lorenzo, 1050 m, Tierras Morenas, R. F. Cord. Guanacaste, March 1990, April 1991, June 1991, October 1991, November 1991, January 1992, 23 March- 21 April 1992, July 1992, August 1992 (INBIO); Río Higuerón, 6 mi SE, 6 mi W Cañas, 8 February 1969 (USNM); Tilarán, 7 July 1972, 30 July 1972 (FSCA); 3 mi. W. Tilarán, 1969 (USNM). Heredia- El Angel falls, Vara Blanca area, 21 June 1969 (USNM); Los Arbolitos, 0–100 m (INBIO); Chilamate, 75 m, June-July 1989 (MUCR); Est. El Ceibo, Braulio Carillo, N.P., 400–500 m, March 1990 (INBIO); Est. Magasasay, P. N. Braulio Carillo, 200 m, January 1991 (INBIO); Est. Hitoy Cerere, 100 m, R. Cerere, Res. Biol. Hitoy Cerere, 7–26 January 1992, 19–29 April 1992, (INBIO); Est. Biol. La Selva, 150 m, 6 March 1965 (BYUC), 17 July 1973 (EMEC), 22 January 1989, April-May 1993 (MUCR), 23–30 June 2001 (USNM), 7 April 2003 (USNM), 14 June 2003 (USNM), 12 March 2005 (USNM), 13 March 2005 (USNM); 9 km E. Puerto Viejo, 14–15 July 1986 (SEMC, USNM); Finca La Selva nr. Puerto Viejo, 21–22 June 1969, 24 July 1969, 2–7 August 1969, 19 March 1973, 28 July 1989 (USNM); La Selva Biol. Sta., 2 km. S. Pt. Viejo, 3–5 June 1984 (EGRC); La Selva Biol. Sta., 3 km S Pto. Viejo, 20 July 1992; 12 km. S. Puerto Viejo, 500 ft., 23–26 September 1986 (FSCA); Rara Avis Biological Station, 5–22 November 2011 (USNM); Sendero Antigua, Est. Carillo, 8 January 1993 (INBIO); 11 km SE La Virgen, 450–550 m, 9 April 2003 (USNM); P. N. Tortuguero, Estación Sierpe, 0–100 m (INBIO); R. V. S. Barra del Colorado, Cerro Tortuguero, 0–100 m (INBIO); R. V. S. Barra del Colorado, Estación Sardinas, 0–100 m (INBIO); R. V. S. Barra del Colorado, A. C. Llanuras del Tortuguero, 0–100 m (INBIO); 11 km NE Vara Blanca, 1450–1550 m, 20 March 2005 (USNM). Limón- 4 km NE Bribri, November 1991 (USNM); Cerro Tortuguero, 0–120 m, P. N. Tortuguero, April 1989, October 1989, December 1989, January 1990, February 1990, 20 September- 7 October 1990, September 1991, June 1992, March 1993 (INBIO); Est. Cuatro Esquinas, 0 m, P. N. Tortuguero, April 1989, October 1989, November 1989, 20 September- 7 September 1990, October 1990, November 1990, December 1990, 23 April- 13 May 1991, June 1991, August 1991, November 1991, 27 March- 9 May 1992, December 1992, January 1993 (INBIO); Guacimo, 22 February 1988 (TAMU); 7 mi N Guacimo, 22 February- 3 March 1988 (BYUC); Guápiles, 30 October 1992 (MUCR); Hamburg Farm, Reventazón, 22 May 1931, 16 January 1936 (USNM), 1 January 1932, 28 January 1933, 27 January 1925 (DEI), 31 January 1931 (MUCR); Est. Hitoy Cerere, 100 m, R. Cerere, Res. Biol. Hitoy Cerere, 7–26 January 1992 (INBIO); Limón, 5 February 1989 (USNM); Puerto Vargas, 7 December 1968 (MUCR); Río Sardinas, 10 m, R.N.F.S. Barra del Colorado, September 1992, 10–14 October 1992, 11 December 1992 (INBIO); Salvadora Farm, Parismina Fluss, 19–31 December 1930 (DEI, USNM); Sector Cerro Cocorí, Fca. de E. Rojas, 150 m, May 1991, August 1991, October 1991, November 1991, January 1992, 31 January- 26 February 1992, 26 March- 24 April 1992, 28 May- 17 June 1992, 26 June- 16 July 1992, 12–31 August 1992, 10–30 September 1992, October 1992, 9–30 November 1992, January 1993, February 1993, March 1993, April 1993, May 1993 (INBIO). Puntarenas- Atam del Mogo, 50–100 m, 3 August 1993 (MUCR); Estación Biológica Las Alturas, 1400–1500 m (INBIO); Barranca nr. Puntarenas, 6 July 1972 (FSCA); Barranca site, 10 km N. Puntarenas, 17 June 1969, 11 September 1969 (USNM); 5 km S. Buenos Aires, 15 August 1969 (USNM); 25 km S. Buenos Aires, 10 August 1969 (USNM); Est. Carara, 200 m, Res. Biol. Carara, February 1990 (INBIO); Golfito, 30 October 1950 (CASC), 19 July 1972, 22 July 1981 (FSCA); Est. La Casona, 1520 m, Res. Biol. Monteverde, April 1992, September 1992 (INBIO); Est. Leonel Hernandes, 1600 m, Res. Biol. Monteverde, June 1991 (INBIO); P. N. Manuel Antonio, 80 m, Quepos, July 1991, September 1991, October 1991 August 1992, October 1992 (INBIO); Monteverde, Cordillera de Tilarán, 10 March 1991, 14 March 1991 (EGRC); Monteverde Cloud For. Res., 18–19 May 1985 (EMEC); Monteverde Reserve, 3 June 1992 (CDFA); Monteverde (EGRC), 12–24 May 1989 (SEMC); Est. Queb., Bonita, 50 m, Res. Biol. Carara, 17 March- 30 April, April 1992, May 1992, July 1992, August 1992, September 1992, 6–27 November 1992, January 1993, February 1993, March 1993, (INBIO); Rancho Quemado, 200 m, Peninsula de Osa, April 1989, October 1989, February 1991, July 1991, October 1991, November 1991, 21 March- 7 April 1992, April 1992, May 1992, 1–31 August 1992, December 1992 (INBIO); 20 km NE Puntarenas, 17 March 1965 (BYUC); Río San Lorenzo, 1050 m, Tierras Morenas, Z. P. Tenorio, 21 March- 7 April 1992 October 1992, December 1992, January 1993, February 1993 (INBIO); San Luis, 1040 m, R.B. Monteverde, 24 August- 15 September 1992, October 1992 (INBIO); San Vito Las Cruces, 20 November 1988 (INBIO); Est. Sirena, 0–100 m, P. N. Corcovado, October 1989, December 1989, January 1990, March 1990, April 1990, June 1990, September 1990, October 1990, March- June 1991, July 1991, August 1991, September 1991, November 1991, 21 March- 21 April 1992, April 1992 (INBIO); Sirena Corcovado, August 1993 (MUCR); Fca. Cafrosa, Est. Las Mellizas, P. N. Amistad, 1300 m, October 1989, November 1989, December 1989 (INBIO); Fca. Las Cruces, San Vito de Java, 27 June 1969, 11–18 August 1969 (USNM), 20 July 1972 (FSCA); F. Las Cruces, 6 mi. S. San Vito, 1200–1400 m, 21–25 August 76 (CASC); Puerto Cortes, 19 July 1972 (FSCA); 22 mi SW San Vito, 11 August 1969 (USNM); San Vito-Villa Neilly area, 13 August 1969 (USNM); Osa Peninsula, 3.5 mi. S. Rincón, 28 February- 12 March 1969 (CASC); Río Claro, sea level, 19 August 1969 (USNM); Rincón, 27 July 1972 (FSCA); 5 km S. Rincón, 20 March 1973 (SEMC); Osa Peninsula, 2.5 mi SW Rincón, 1–7 March 1967 (USNM); R. F. Golfito Dulce, 3 km SW Rincón, 10 m, October- December 1990, March- May 1991 (USNM); Río Piedras, sea level, 15 August 1969 (USNM); Sirena, Corcovado N. P., December 1989 (INBIO); 2 mi. S. Palmar Sur, 19 August 1969 (USNM); 3 mi. S. Palmar Sur, 11 August 1969 (USNM); 5 mi. S. Palmar Sur, 15 August 1969 (USNM); 1.5 mi. S. Palmar Sur, 11 August 1969 (USNM); 18 mi. S. Palmar Sur, 11 August 1969 (USNM); Jardin Botanico Las Cruces, San Vito, 1200–1300 m (INBIO); Corinto, Macacona, Esparza, 300–400 m (INBIO); A. C. L. A. P., Garabito, Reserva Biol Carara, Est Quebrada Bonita, 0–100 m (INBIO); A. C. P. C., Garabito, Tarcoles, Estación Quebrada Bonita, 100–200 m (INBIO); P. N. Piedras Blancas, Estación Esquinas, Peninsula de Osa, 0–100 m (INBIO); Río Bonito, 1.4 km O Cerro Gamba, 200–300 m (INBIO); Pque Nal Corcovado, Est Sirena, Osa Peninsula, 0–100 m (INBIO); Cerro La Torre, Fca La Purruja, Rila Matahambre, 100–200 m (INBIO); Golfito, Jiménez, Albergue Cerro de Oro, 100–200 m (INBIO); A. C. O. Golfito, Reserva Ftal Golfito Dulce, Est Agujas, 200–300 m (INBIO); Cerro Anguciana, Llano Bonito, Piedras Blancas, en Osa, 800–900 m (INBIO); Osa, Sierpe, 0.2 km NW Estación Esquinas, 0–100 m (INBIO); Guerra, Peninsula de Osa, 0–100 m (INBIO); Dos Brazos del Río Rincón, 0–100 m (INBIO); Est San Miguel, 3 km NW Cabo Blanco, 100–200 m (INBIO); Guacimal, Finca Buen Amigo Monteverde, 4 km S de la Reserva, 1000–1100 (INBIO). San José- San Isidro, 9 mi S, 31 December 1988 (BYUC); 12 mi. N San Isidro del General, 26 June 1969 (USNM); 7 mi. S. San Isidro del General, 26 June 1969 (USNM); San José (USNM); Pque Nal Braulio Carrillo, 1600–1700 m (INBIO); Perez Zeledón, Santa Elena Las Nubes, 1200–1300 m (INBIO); R. B. Carara, 2 km N Bijagual, 200–300 m (INBIO); Est. Bijagual, 600 m N Bijagualito, 400–500 m (INBIO); Estación Bijagual, 1.5 km N Bijagual, 400–500 m (INBIO). **GUATEMALA:** Santa Roca- Escuintla, Finca Caobamal, 17 km W Taxisco, 150 m, 23 June 1993 (CMNC). **Honduras:** Yoro- PN PicoPijo, 13 May 2002 (USNM). **MEXICO:** Veracruz- Peñuela, 11 July 1941 (FMNH). **NICARAGUA:** Granda- Res. Nac. Volcán Mombacho, 1150 m, 2-VI-2002 (SEMC, USNM). Jinotega- Peñas Blancas, 1300 m, 25 July 1997 (USNM). Malagualpa- 6 km N Malagualpa, 1350 m, 19 May 2002 (SEMC, USNM). Río San Juan- 16 km ESE El Castillo, Refugio Bartola, 23 April–10 May 1999 (USNM); San Juan del Norte, 26–29 September 2005 (BYUC). **PANAMA:** Chiriquí- Galera de Chorcha, 3 July 1976 (EGRC). Panamá- Ancón, 19–21 August 1970 (USNM). Total: 2143.

#### Cephaloleia bicolor

Taxon classificationAnimaliaColeopteraChrysomelidae

Uhmann, 1930c

http://species-id.net/wiki/Cephaloleia_bicolor

Fig. 86

Cephalolia bicolor Uhmann 1930c: 34. Uhmann 1936b: 116 (noted).
Cephaloleia bicolor Uhmann. Uhmann 1957b: 16 (catalog); Gaedike and Döbler 1971: 343 (types); Staines 1997b: 413 (Uhmann species list).

##### Description.

Elongate; subdepressed; shining; black; palps and basal half of elytra and epipleuron yellowish-brown; male with venter black; female with prosternum and base of abdomen reddish-yellow; antennae dark. Head: vertex finely, densely punctate, faint medial carina present; frons not projecting; slightly depressed between eyes. Antenna: reaches to humerus; slender; antennomeres oblong; 1 elongate; 2 shorter, oval; 3 longer than 2, elongate; 4–5 subequal in length, each shorter than 3; 6–10 each longer than 5; 11 longer than 10, pointed at apex; 1–2 punctate with scattered setae; 3–11 setose. Pronotum: transverse; lateral margin straight then rounding to anterior angle, slightly canaliculate; anterior angle rounded, produced; posterior angle acute; anterior margin straight; disc subconvex; surface punctate except medial longitudinal line on disc; basal impression absent; pronotal length 1.0–1.3 mm; pronotal width 1.3–1.5 mm. Scutellum: pentagonal; impunctate. Elytron: lateral margin straight, smooth, finely margined; apex rounded; sutural angle without tooth; humerus rounded, not produced; slightly constricted behind humerus; moderately punctate-striate, punctures becoming obsolete apically; elytral length 4.2–4.6 mm; elytral width 1.9–2.1 mm. Venter: pro-, meso-, and metasterna punctate, each puncture with pale seta; abdominal sterna punctate, each puncture with pale seta; suture between sterna 1 and 2 complete; last sternite with apical margin sinuate medially in male; truncate in female. Leg: slender; punctate, each puncture with pale seta; tibia with fringe of setae on inner margin of apex. Total length: 5.5–6.0 mm.

##### Diagnosis.

This is a bicolored species with the apical section of the elytra darker. It is most similar to *Cephaloleia apicenotata* and *Cephaloleia bicoloriceps*. It can be distinguished by the straight lateral margin of the pronotum, by antennomere 2 being oval, and by the elytral punctures becoming obsolete apically.

##### Distribution.

Bolivia, Brazil (Matto Grosso), Colombia, Ecuador, Peru, Venezuela.

##### Type material examined.

Holotype: Colombia [handwritten label]/ Holotypus [red printed label]/ Cephalolia bicolor Uh., Det. E. Uhamnn (DEI).

##### Specimens examined.

**Bolivia:** Cochabamba- February 1951, November 1953 (USNM). **Brazil:** no further data (AMNH). **Colombia:** no further data (NHMW). **Ecuador:** Sucumbios- 2 km E Lumbaqui, 7 August 1998 (AJGC); 9 km SE Lumbaqui, 650 m, 7–8 August 1998 (AJGC). **Peru:** Junin- San Martin, 1600 ft., 5 December 1946 (AMNH). **Venezuela:** Aragua- PN H. Pittier, Rancho Grande, Portochuela, 1120 m, 12 July 1998 (USNM); E-Merida, La Macuy, May 1984 (USNM); Rancho Grande, Maracay, January 1954 (USNM). Total: 11.

#### Cephaloleia bicoloriceps

Taxon classificationAnimaliaColeopteraChrysomelidae

Pic, 1926c

http://species-id.net/wiki/Cephaloleia_bicoloriceps

Fig. 87

Cephalolia bicoloriceps Pic 1926c: 13.
Cephaloleia bicoloriceps Pic. Uhmann 1957b: 16 (catalog); Descarpentries and Villiers 1959a: 139 (types).

##### Description.

Oblong-elongate; subdepressed; shining; head, antennae, pronotum (except lateral margins), scutellum, apical ½ of elytra, venter, and femora black; tibiae and tarsi paler; basal ½ elytra yellowish, some specimens have a black sutural vitta which narrows apically to the darker apical ½; elytra with lateral and apical (almost to sutural angle) margins pale. Head: vertex moderately punctate, medial sulcus absent; eye convex; frons not projecting; depressed between eyes. Antenna: reaches beyond humerus; slender; antennomere 1 elongate, not incrassate; 2 ½ length 1; 3–4 elongate, each subequal in length to 1; 5–10 transverse, decreasing in length, each shorter than 4; 11 longer than 10, pointed at apex; 1–6 punctate with scattered setae; 7–11 setose. Pronotum: quadrate; lateral margin straight for basal ½ then slightly rounding to anterior angle, canaliculate; anterior angle angulate, slightly produced; posterior angle acute; anterior margin straight; disc subconvex, with medial longitudinal impression, narrowing basally; surface sparsely, strongly punctate; pronotal length 1.2–1.4 mm; pronotal width 1.4–1.6 mm. Scutellum: pentagonal; punctate. Elytron: lateral margin straight, smooth, margined; apex rounded; sutural angle with small tooth; humerus rounded, slightly produced; constricted behind humerus; finely punctate-striate; elytral length 3.9–4.2 mm; elytral width 2.0–2.2 mm. Venter: pro-, meso-, and metasterna impunctate medially, punctate laterally; abdominal sterna punctate, each puncture with pale seta; suture between sterna 1 and 2 complete; last sternite with apical margin emarginate medially in male, rounded, entire in female. Leg: slender; punctate; tibia with fringe of setae on inner margin of apex. Total length: 5.4–5.7 mm.

##### Diagnosis.

This is a bicolored species with the apical section of the elytra darker. It is most similar to *Cephaloleia apicenotata* and *Cephaloleia bicolor*. It can be distinguished by the straight lateral margins of the pronotum, by antennomere 2 being elongate, and by the elytral punctures being distinct apically.

##### Distribution.

Bolivia, Brazil, Colombia, Ecuador.

##### Type material examined.

Holotype: P [unreadable], Bonvonn [handwrtten green label]/ fulvieps ex. coll. Donckier [handwritten label]/ Cephaloleia fulvipes [handwritten label]/ bicoloriceps sp. n. [handwritten label]/ Type [handwritten label]/ Museum Paris Coll. M. Pic [blue printed label]/ Type [red printed label]/ Holotype [red printed label]/ MNHN EC 2650 [printed label] (MNHN).

##### Specimens examined.

No label data (USNM). **BOLIVIA:** La Paz- Nor Yungas, Chica Parque, near Coroico, 30 November 2011 (BYUC). **Brazil:** no further data (MNHN, USNM); Juanfue (USNM). **Colombia:** no further data (USNM). **Ecuador:** Napo- Jatun Sacha, June 1986 (USNM); Misahualli, nr. Tena, 6–19 October 2001 (BYUC), 27 April–2 May 2003 (BYUC). Orellana- Yasuni Res. Stn., 4–9 May (BYUC); Estación Cientifica Yasuni, 215 m, 5–10 September 1999 (EGRC). Total: 11.

#### Cephaloleia bifasciata

Taxon classificationAnimaliaColeopteraChrysomelidae

Weise, 1905b

http://species-id.net/wiki/Cephaloleia_bifasciata

Fig. 88

Cephalolia bifasciata Weise 1905b: 56. Weise 1911a: 7 (catalog), 1911b: 11 (catalog); Uhmann 1936b: 114 (noted).
Cephaloleia bifasciata Weise. Uhmann 1957b: 16 (catalog); Descarpentries and Villiers 1959a: 139 (types); McKenna and Farrell 2006: 10949 (phylogeny).

##### Description.

Elongate; subparallel; subdepressed; yellowish; head, antennae, and pronotum (except paler lateral and basal margins) black; elytra yellowish with lateral and apical margins black and black transverse band beyond middle; scutellum, venter, and legs yellowish. Head: vertex sparsely, finely punctate, medial sulcus absent; frons not projecting; slightly depressed between eyes. Antenna: reaches to humerus; slender; antennomere 1 incrassate, 2× length of 2; 2 subglobose; 3–4 elongate, subequal in length, each slightly longer than 2; 5–10 subequal in length, each shorter than 3; 11 2× length 10, pointed at apex; 1–2 punctate with scattered setae; 3–11 setose. Pronotum: subquadrate; lateral margin straight then rounding to anterior angle, canaliculate; anterior angle slightly angulate, not produced; posterior angle acute; anterior margin straight; disc subconvex; surface finely punctate laterally, nearly impunctate on disc; transverse basal impression present medially; pronotal length 1.3–1.7 mm; pronotal width 1.5–1.9 mm. Scutellum: pentagonal; impunctate. Elytron: lateral margin straight, smooth, narrowly margined; apex rounded; sutural angle with small tooth; humerus rounded, not produced; slightly constricted behind humerus; moderately punctate-striate, rows confused basally, scutellar row long; declivity beginning just behind humerus at puncture row 7 edged with faint carina; elytral length 5.3–5.7 mm; elytral width 2.0–2.2 mm. Venter: pro-, meso-, and metasterna punctate, each puncture with pale seta; abdominal sterna punctate, each puncture with pale seta; suture between sterna 1 and 2 obsolete medially; last sternite with apical margin rounded in male, truncate in female. Leg: slender; punctate; tibia with seta in each puncture and fringe of setae at apex. Total length: 6.8–7.2 mm.

##### Diagnosis.

This species is similar to *Cephaloleia hnigrum* and *Cephaloleia recondita*. It can be distingusihed by the lateral margin of the elytra having black markings extending to puncture row 6.

##### Distribution.

Ecuador.

##### Type material examined.

Syntype: Ecuador, Donckier [green printed label]/ I. Weise det. [printed label]/ Type [printed salmon-colored label]/ Cephalolia bifasciata m [handwritten label] (ZMHB, 1).

##### Specimens examined.

**Ecuador:** no further data (MNHN). Esmeraldas- 31.7 km NW Lita, 620 m, 23 August 1997 (CDFA, USNM); Canton San Lorenzo, Chuchubi, 2 December 2008 (BYUC). Pinchincha- Chimba, July 1897 (USNM). Total: 9.

#### Cephaloleia brevis

Taxon classificationAnimaliaColeopteraChrysomelidae

Staines sp. n.

http://zoobank.org/DE1F1C85-942C-4926-B91D-B6FB55F9CED1

http://species-id.net/wiki/Cephaloleia_brevis

Fig. 89

##### Description.

Small; stubby; subdepressed; head black, antennomeres 1–2 reddish-brown, 3–10 black, 11 black basally, pale apically, pronotum black with pale lateral margins, scutellum elytra, and venter castaneous, legs yellowish-brown. Head: vertex moderately punctate; medial sulcus absent; keel present between antennal bases; frons punctate, not projecting; slightly depressed between eyes. Antenna: reaches to humerus; slender; antennomere 1 elongate, cylindrical; 2–3 elongate, cylindrical, subequal in length, each ¾ length of 1; 4–5 cylindrical, subequal in length, longest; 6–10 transverse; 11 2× length of 10, pointed at apex; 1–3 punctate with scattered setae; 4–11 setose. Pronotum: transverse; lateral margin straight slightly converging basally then rounding to anterior angle, margined; anterior angle rounded, not produced; posterior angle acute; anterior margin straight; disc subconvex; surface irregularly punctate medially, denser and larger punctures laterally and basally; basal impression absent; pronotal length 1.0 mm; pronotal width 1.6 mm. Scutellum: pentagonal; impunctate. Elytron: lateral margin straight, smooth, margined; apex rounded, finely serrate; sutural angle without tooth; humerus rounded, not produced; constricted behind humerus; moderately punctate-striate, row 10 removed from lateral margin along constriction, rows converge and unite apically; elytral length 3.0 mm; elytral width 1.8 mm. Venter: pro-, meso-, and metasterna impunctate medially, punctate laterally; abdominal sterna punctate, each puncture with pale seta; suture between sterna 1 and 2 complete; apical margin of last sternite emarginate medially in male. Leg: slender; punctate, each puncture with pale seta; tibia with fringe of setae on inner apical margin. Total length: 4.0 mm.

**Figures 89–97.**
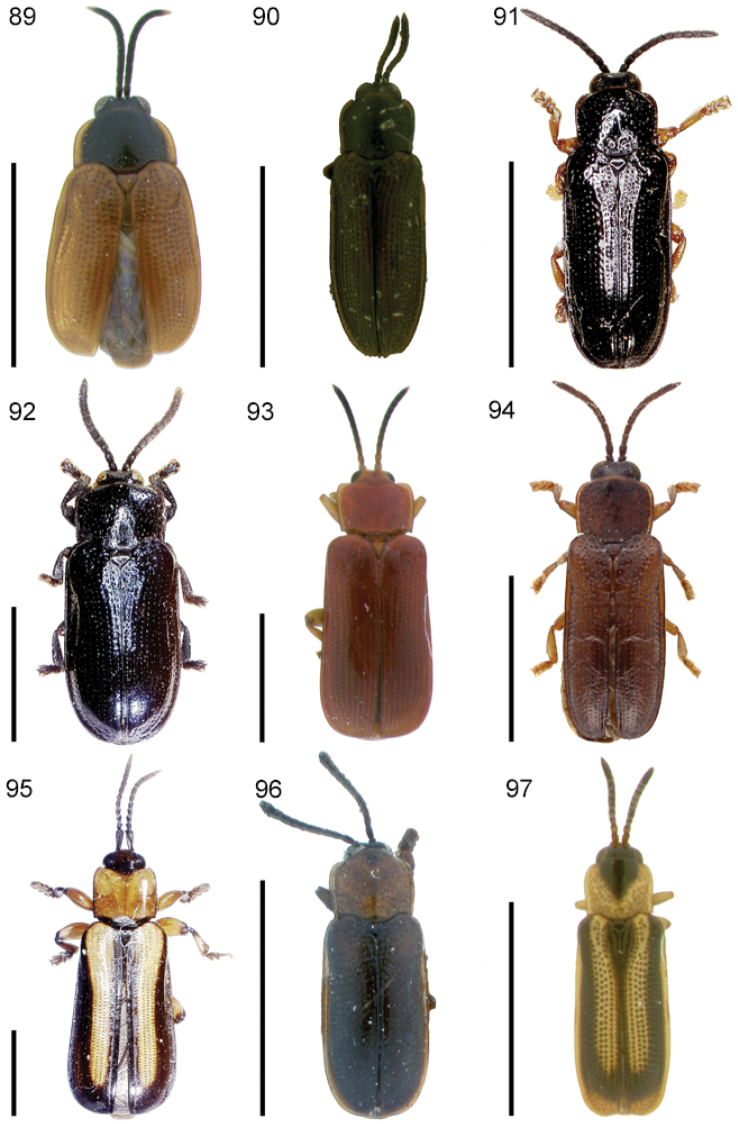
Habitus. **89** *Cephaloleia brevis* sp. n. **90** *Cephaloleia brunnea* **91** *Cephaloleia bucki* **92** *Cephaloleia caeruleata* **93** *Cephaloleia calathae* sp. n. **94** *Cephaloleia castanea* **95** *Cephaloleia championi* **96** *Cephaloleia chevrolatii* **97** *Cephaloleia chica* sp. n. Scale bars equal 3 mm.

##### Etymology.

Brevis (Latin) meaning short or narrow for the short, stubby appearance of this species.

##### Diagnosis.

This species is similar to *Cephaloleia pretiosa*. It can be easily be distinguished by the serrulate apical margin of the elytra.

##### Distribution.

French Guiana.

##### Type material.

Holotype male: French Guiana, Saul, 7 km N, 0.5 km ESE, Les Eaux Claires, Mt. Le Fumée, 3°39'46"N, 53°13'19"W, 300 m, 4–8 Jun 1997, J. Ashe, R. Brooks, FG1AB97 164, ex. flight intercept trap/ Holotype *Cephaloleia brevis* Staines, des. C. L. Staines 2012 (red label) (SEMC).

#### Cephaloleia brunnea

Taxon classificationAnimaliaColeopteraChrysomelidae

Staines, 1996

http://species-id.net/wiki/Cephaloleia_brunnea

Fig. 90

Cephaloleia brunnea Staines 1996: 21. Staines 2008: 2 (key), 2009a: 21 (noted).

##### Description.

Elongate; subconvex; small; brown; pronotum and head slightly darker than elytra; elytra with suture darker; venter dark brown; leg with tarsi and apex of tibia paler than rest. Head: vertex densely punctate, medial sulcus absent; frons not projecting; depressed between eyes. Antenna: reaches to humerus; slender; antennomere 1 elongate, subequal in length to 3; 2 transverse, shorter than 1 or 3; 4–10 transverse, subequal in length, each shorter than 3; 11 rounded at apex, subequal in length to 1 and 3; 1–2 punctate with scattered setae; 3–11 setose. Pronotum: transverse; lateral margin straight for basal ¾ then rounding to anterior angle, canaliculate; anterior angle obtuse, slightly produced; posterior angle acute; anterior margin emarginate behind head; disc slightly convex; surface deeply punctate; punctures more dense laterally; basal impression absent; pronotal length 0.7–0.9 mm; pronotal width 1.0–1.1 mm. Scutellum: pentagonal; punctate. Elytron: lateral margin straight, smooth, margined; apex rounded; sutural angle without tooth; humerus rounded, not produced; slightly constricted behind humerus; moderately punctate-striate, rows converge and unite apically; elytral length 3.0–3.1 mm; elytral width 1.3–1.4 mm. Venter: pro-, meso-, and metasterna impunctate medially, punctate laterally; abdominal sterna sparsely punctate, each puncture with pale seta; suture between sterna 1 and 2 obsolete medially; last sternite with apical margin broadly emarginate medially in male, truncate in female. Leg: slender; punctate, each puncture with pale seta; tibia with fringe of setae on inner margin of apex. Total length: 4.0–4.1 mm.

##### Diagnosis.

This species is similar to *Cephaloleia rubra*. It can be distinguished by the densely punctate pronotum and by antennomere 1 being subequal in length to 3.

##### Distribution.

Trinidad.

##### Type material examined.

Holotype: Morne Bleu, 2700', Trinidad, W. I., Aug. 6,1969, H. and A. Howden/ Holotype Cephaloleia brunnea Staines, Des. C. L. Staines 1994 [red label] (CMNC).

##### Specimens examined.

**TRINIDAD:** Curepe, St. George Par., 28–30 November 1977 (CNC); Morne Bleu, 2700', 15 August 1969 (CMNC); N. range, Arima-Blanchisseuse rd., mi. 10, 11 May 1985 (EGRC); St. Augustine, Mt. St. Benedict Abby, 9 July 1994 (EGRC). Total: 9.

#### Cephaloleia bucki

Taxon classificationAnimaliaColeopteraChrysomelidae

Uhmann, 1957b

http://species-id.net/wiki/Cephaloleia_bucki

Fig. 91

Cephaloleia bucki Uhmann 1957b: 36. Buck 1958: 146 (host plant); Uhmann 1964a: 403 (catalog); Gaedike and Döbler 1971: 345 (types); Staines 1997b: 413 (Uhmann species list); Staines and Staines 1997: 4 (types).

##### Description.

Elongate; subdepressed; shining; brownish-yellow; head black; pronotum black with brownish-yellow lateral margin; antennomeres 8–11 darker. Head: vertex finely punctate, medial sulcus absent; eye convex; frons not projecting; slightly depressed between eyes. Antenna: as long as head and pronotum combined; robust, cylindrical; antennomere 1 subequal to 3; 2 shorter than 1; 4–5 subequal in length, slightly longer than wide, each shorter than 3; 6–10 subequal in length, each shorter than 5; 11 2× length of 10, acutely pointed at apex; 1–2 punctate with scattered setae; 3–11 setose. Pronotum: transverse; lateral margin sinuate basally then straight and rounding to anterior angle, slightly canaliculate; anterior angle rounded, not produced; posterior angle acute; anterior margin curved anteriorly; disc subconvex, nearly impunctate; surface sparsely, irregularly punctate, punctures larger and denser basally; transverse basal impression present medially; pronotal length 1.0–1.1 mm; pronotal width 1.3–1.4 mm. Scutellum: pentagonal; impunctate. Elytron: lateral margin straight, smooth, margined; apex rounded; sutural angle without tooth; humerus rounded, not produced; slightly constricted behind humerus; subconvex; finely punctate-striate; elytral length 2.9–3.0 mm; elytral width 1.3–1.4 mm. Venter: pro-, meso-, and metasterna punctate; abdominal sterna punctate, each puncture with pale seta; suture between sterna 1 and 2 complete; male with apical margin of sternite 5 broadly rounded, female strongly truncate, convex medially. Leg: slender; punctate; femur and tibia with pale seta in each puncture; tibia with fringe of setae on inner margin of apex. Total length: 3.9–4.0 mm.

##### Diagnosis.

This species is most similar to *Cephaloleia approximata*. It can be distinguished by the vertex of the head without a medial carina or sulcus and by the elytra not being slightly costate apically.

##### Host plant.

*Carex* sp. (Cyperaceae) (Buck 1958).

##### Distribution.

Brazil (Rio Grande do Sul).

##### Type material examined.

Holotype: Brazil, Rio Grande do Sul, Villa Oliva, 4.XI.1952/28.I.1954, Buck [printed label]/ Holotypus [red printed label]/ Cephaloleia bucki Uh., Det. E. Uhmann (DEI).

##### Specimens examined.

**Brazil:** Río Grande do Sul- Dios Immaos, 25 May 1940 (USNM); Porto Alegre (USNM); Villa Oliva, 4 November 1952 (DEI). Total: 3.

#### Cephaloleia caeruleata

Taxon classificationAnimaliaColeopteraChrysomelidae

Baly, 1875a

http://species-id.net/wiki/Cephaloleia_caeruleata

Fig. 92

Cephalolia coeruleata Baly 1875a: 75. Gemminger and Harold 1876: 3601 (lapsus calami, catalog); Donckier 1899: 548 (catalog).
Cephaloleia caeruleata Baly. Weise 1911a: 7 (catalog), 1911b: 13 (catalog); Uhmann 1957b: 16 (catalog); Staines and Staines 1999: 523 (Baly species list).

##### Description.

Elongate; subdepressed; slightly shining; dorsum metallic-blue, antennae black. Head: vertex coarsely punctate; carina present between antennal bases; frons not projecting; slightly depressed between eyes. Antenna: ½ length of body; scarcely thickened apically; slender; antennomere 1 slightly thickened; 2 ¾ length 1; 3 nearly 1½ length of 2; 4–6 decreasing in length, each shorter than 2; 7–10 transverse, subequal in length, each shorter than 6; 11 2× length 10, acutely pointed at apex; 1–5 punctate, glabrous; 6–11 setose. Pronotum: transverse; lateral margin straight, slightly sinuate then rounded and convergent to anterior angle, canaliculate; anterior and posterior angles acute; anterior margin emarginate behind head; disc subconvex; surface coarsely punctate laterally, disc and anterior margin behind head nearly impunctate; basal impression absent; pronotal length 1.2 mm; pronotal width 2.0 mm. Scutellum: pentagonal; as wide as long; sharply pointed at apex; with two fovea apically; impunctate. Elytron: lateral margin straight, smooth, narrowly margined; apex rounded; sutural angle without tooth; humerus with ill-defined protuberance; slightly constricted behind humerus; disc subconvex; slightly flattened along suture; moderately punctate-striate, punctures larger laterally; elytral length 4.4 mm; elytral width 2.3 mm. Venter: prosternum impunctate medially, rugose laterally; meso- and metasterna punctate, each puncture with pale seta; abdominal sterna punctate, each puncture with pale seta; suture between sterna 1 and 2 complete; last sternite with apical margin bisinuate. Leg: slender; punctate; femur and tibia with pale seta in each puncture; femur robust; tibia with fringe of setae at apex. Total length: 6.1 mm.

##### Diagnosis.

This species is similar to *Cephaloleia dilatata* and *Cephaloleia diplothemium*. It can be distinguished by the cylindrical antennomere 1 which is shorter than 3, by the elytral punctures being larger laterally, and by the prosternum being rugose laterally.

##### Distribution.

Brazil (Río de Janiero, Santa Catharina), Ecuador.

##### Type material examined.

Holotype: Brazil, New Friburg [printed label]/ Baly Coll. [printed label]/ Cephalolia caeruleata Baly, Brazil [handwritten blue label] (BMNH).

##### Specimens examined.

**Brazil:** Río de Janiero- no further data; 26 February 1952 (USNM). Santa Catharina- Nova Teutonia, February 1977, March 1977 (EGRC). **Ecuador:** ?- San Gabriel, 750 m, 12 October 1970 (USNM). Total: 3.

#### Cephaloleia calathae

Taxon classificationAnimaliaColeopteraChrysomelidae

García-Robledo & Staines sp. n.

http://zoobank.org/574276D0-5663-499E-BC1B-564F86FE8043

http://species-id.net/wiki/Cephaloleia_calathae

Fig. 93

##### Description.

Elongate; subparallel; subdepressed; reddish-brown, venter and legs paler; antennae (except basal 2 antennomeres) and eyes black. Head: vertex finely punctate; faint medial sulcus present; small projection present between antennal bases; not depressed between eyes; frons punctate, each puncture with pale seta, not projecting. Antenna: reaches to humerus; antennomere 1 incrassate, 3× length of 2; 2–5 cylindrical, subequal in length; 6–10 transverse, subequal in length; 11 slightly longer than 10, pointed at apex; 1–2 punctate with scattered setae; 3–11 setose; male with antennomeres 1–3 with triangular projection in inner apical margin. Pronotum: subquadrate; lateral margin sinuate basally, then rounding to anterior angle, canaliculate; anterior angle bluntly pointed; posterior angle acute; anterior margin emarginate behind head; disc depressed; surface irregularly, sparsely punctate; basal impression absent; pronotal length 1.4–1.7 mm; pronotal width 1.9–2.2 mm. Scutellum: pentagonal; impunctate. Elytron: lateral margin straight, smooth, margined; apical margin rounded, narrowly margined; sutural angle without tooth; humerus rounded, slightly produced; slightly constricted behind humerus; shallowly punctate-striate, punctures larger on disc, rows confused apically; elytral length 4.9–5.3 mm; elytral width 2.3–2.5 mm. Venter: pro-, meso-, and metasterna impunctate medially, sparsely punctate laterally; abdominal sterna punctate, each puncture with pale seta; suture between sterna 1 and 2 obsolete medially; apical margin of last sternite weakly emarginate medially in male, sinuate in female. Leg: robust; femur punctate; tibia with fringe of setae on inner apical margin. Total length: 7.0–8.4 mm.

##### Etymology.

Named for the genus of the host plant. The name is feminine.

##### Diagnosis.

This species is similar to *Cephaloleia conforma* and *Cephaloleia erichsonii*. It can be distinguished by the elytra lacking a sulcus on the humeral callus, by antennomere 1 being incrassate and 3 times the length of 3, and by the elytral punctures on the disc being larger than those laterally.

##### Host plant.

Adults have been collected off *Calathea crotalifera* S. Watson, *Cephaloleia guzmanioides* LB Sm and Idrobo (Marantaceae).

##### Distribution.

Costa Rica.

##### Type material.

Holotype male: Costa Rica, Puntarenas, Coto Brus, Las Cruces Biol. Station, 1200 m, CG-MAY-11–63B, 10 March 2012, C. García-Robledo, ex. *Calathea crotalifera*/ Holotype *Cephaloleia calathae* García-Robledo & Staines, des. 2012 [red label] (USNM). Paratypes (6 males, 6 females) (each with Paratype *Cephaloleia calathae* García-Robledo & Staines, des. 2012 [red label]): same data as holotype except GC-MAY-11-53, GC-MAY-11-63, GC-MAY-11-63A, GC-MAY-11-63C, GC-MAY-11-63D, GC-MAY-11-63E, GC-MAY-11-63F, GC-MAY-11-672E; same data as holotype except 6 March 2012, GC-MAY-11-83, GC-MAY-11-83A, GC-MAY-11-83B, GC-MAY-11-83C (USNM, IEXA).

#### Cephaloleia castanea

Taxon classificationAnimaliaColeopteraChrysomelidae

Pic, 1929a

http://species-id.net/wiki/Cephaloleia_castanea

Fig. 94

Cephalolia castanea Pic 1929a: 139.
Cephaloleia castanea Pic. Uhmann 1957b: 16 (catalog); Descarpentries and Villiers 1959a: 139 (types).

##### Description.

Elongate; subdepressed; shining; reddish-yellow; antennae black; legs yellowish. Head: vertex moderately, irregularly punctate, faint medial sulcus present; frons not projecting; depressed between eyes. Antenna: as long as head and pronotum combined; slender; antennomere 1 short, transverse; 2–4 elongate; 2 thick, subequal to 1; 3 slightly longer than 2; 4 ¾ length 3; 5–10 subequal in length, each ¾ length of 4; 11 2× length 10, pointed at apex; 1–3 punctate with scattered setae; 4–11 setose. Pronotum: quadrate; lateral margin straight and slightly divergent for basal ¾ then rounding to anterior angle, slightly canaliculate; anterior angle broadly rounded, not produced; posterior angle acute; anterior margin curved anteriorly; disc subconvex; surface strongly, irregularly punctate; basal impression absent; pronotal length 1.1 mm; pronotal width 1.4 mm. Scutellum: pentagonal; alutaceous. Elytron: lateral margin straight, smooth, narrowly margined; apex rounded; sutural angle without tooth; humerus rounded, produced; slightly constricted behind humerus; moderately punctate-striate; elytral length 3.7 mm; elytral width 1.6 mm. Venter: obscured by card. Leg: slender; femur and tibia punctate; tibia with fringe of setae on inner margin of apex. Total length: 5.0 mm.

##### Diagnosis.

This species is similar to *Cephaloleia nigricornis* and *Cephaloleia opaca*. It can be distinguished by the punctate vertex of the head with a medial sulcus and by antennomere 3 being longer than 1.

##### Distribution.

Brazil (Río de Janiero).

##### Type material examined.

Holotype: Rio, Fry [handwritten label]/ Cephalolia [handwritten label]/ castanea sp. n. [handwritten label]/ Type [handwritten label]/ Museum Paris Coll. M. Pic [blue printed label]/ Type [red printed label]/ Holotype [red printed label]/ MNHN EC 2646 [printed label] (MNHN).

#### Cephaloleia championi

Taxon classificationAnimaliaColeopteraChrysomelidae

Baly, 1885

http://species-id.net/wiki/Cephaloleia_championi

Fig. 95

Cephaloleia championi Baly 1885: 9. Blackwelder 1946: 718 (catalog); Papp 1953: 14 (catalog); Uhmann 1957a: 16 (catalog); Wilcox 1983: 136 (catalog); Staines 1996: 21 (Central America species), 1999: 241 (mimicry), 2004: 312 (host plants), 2011: 48 (faunal list); Staines and Staines 1997: 5 (types), 1999: 523 (Baly species list); McKenna and Farrell 2005: 119 (phylogeny), 2006: 10949 (phylogeny); Schmitt and Frank 2013: 58 (biology).
Cephalolia championi Baly. Donckier 1899: 548 (catalog); Weise 1911a: 7 (catalog), 1911b: 10 (catalog); Uhmann 1936b: 483 (key).

##### Description.

Elongate; flattened; subparallel; reddish-yellow with head (except yellow frons), antennae (except antennomeres 10–11), all margins of pronotum, and sutural and lateral vittae of elytra black; elytra with lateral margin black; venter with prosternum black, mesosternum yellow medially, black laterally, metasternum black, abdominal sterna yellow with black margin; leg with femur yellow with base and apex dark. Head: vertex striate-punctate, faint medial carina present; frons slightly projecting; not depressed between eyes. Antenna: reaches to humerus; robust; antennomere 1 elongate, robust; 2–4 compressed, triangular, subequal in length, each shorter than 1; 5–10 transverse, subequal in length, each shorter than 4; 11 pointed at apex, 2× length of 10; 1–2 punctate with scattered setae; 3–11 setose. Pronotum: transverse; lateral margin converging to anterior angle; anterior angle acute; posterior angle rounded; anterior margin emarginate behind head; disc flattened; surface punctate; widest at base; basal impression absent; pronotal length 1.7–1.9 mm; pronotal width 2.0–2.6 mm. Scutellum: acutely triangular; impunctate. Elytron: lateral margin straight, margined, smooth; apex rounded; sutural angle without tooth; humerus rounded, not produced; slightly constricted behind humerus; declivity beginning just behind humerus at puncture row 7 edged with faint carina; punctures shallow; punctures larger and deeper on disc; elytral length 5.7–7.3 mm; elytral width 2.7–3.3 mm. Venter: pro-, meso-, and metasterna impunctate medially, punctate laterally; abdominal sterna punctate, each puncture with pale seta; suture between sterna 1 and 2 complete; last sternite with apical margin emarginate medially in male, rounded in female. Leg: slender; femur robust; tibia dentate at apex, with fringe of setae on inner margin of apex. Total length: 8.0–9.6 mm.

##### Diagnosis.

This species is similar to *Cephaloleia bella*, *Cephaloleia luctuosa*, and *Cephaloleia vicina*. It can be distinguished by the vertex of the head not being depressed between the eyes, by the suture between abdominal sterna 1 and 2 being complete, by the impunctate pronotum, and by antennomeres 3 and 4 being triangular.

##### Host plant.

Adults have been collected in *Heliconia* sp. (Heliconiaceae) leaf rolls (Staines 1996); *Calathea lutea* Schult. (Marantaceae), *Heliconia imbricata* Baker (Heliconiaceae) (Schmitt and Frank 2013).

##### Distribution.

Costa Rica, Panama.

##### Type material examined.

Syntypes: Bugaba, 800–1500 ft., Champion [printed label]/ Paratipo [red label]/ F. Monros Collection 1959 [printed label]/ Cephaloleia championi Baly, J. S. Baly det. [pink label] (USNM, 1; AMNH, 1).

##### Specimens examined.

**COSTA RICA:** Alajuela- A. C. A., San Ramón, Reserva Biol Alberto Brenes, 1000–1100 m (INBIO); R. San Lorencito, 900 m, R. F. San Ramón, 5 km N de Colonia Palmareña, 13–18 June 1993, December 1992 (INBIO); Res. For. San Ramón, 8 March 1990 (INBIO, MUCR). Cartago- Quebrada Segunda, P. N. Tapantí, 1250 m, March 1992, April 1992, May 1992, August 1992, September 1992, October 1992, December 1992, March 1993 (INBIO). Guanacaste- Río San Lorenzo, 1050 m, Tierras Morenas, Z. P. Tenorio, April 1992, October 1992 (INBIO). Heredia- Barva Volcán o, 6 November 2011 (USNM); 6 km ENE Vara Blanca, 2050 m, March 2002 (USNM); 9 km NE Vara Blanca, 1450–1550 m, 13 March 2005 (USNM); Rara Avis Biological Station, 6 November 2001 (USNM). Puntarenas- Est. Sirena, 0–100 m, P. N. Corcovado, January 1990, April 1990, January 1992 (INBIO), August 1993 (MUCR); Osa Peninsula, 2.5 mi. SW. Rincón, 6 August 1968 (CMNC); Osa Peninsula, 3.5 mi. S. Rincón, 1 March 1969 (USNM); Monteverde Reserve, 17 February 1990 (MUCR); 3 June 1992 (CDFA); 1.5 mi. S. Palmar Sur, 11 August 1969 (USNM); Peninsula de Osa, 9 July 1968 (MUCR); Puntarenas Res. For., Monteverde, 17 February 1990 (USNM); Rancho Quemado, Pen. Osa, February 1991, April 1991, September 1992 (INBIO); 3.5 mi. S. Rincón, Osa Peninsula, 28 February–12 March 1969 (CASC); San Luis, 1040 m, R. B. Monteverde, October 1992 (INBIO); Sirena, Corcovado Nat. Pk., 23 March 1981 (USNM); A. C. O. Golfito, F. Las Cruces, Fca Ilama, 1400–1500 m (INBIO); Estación Esquinas, P. N. Piedras Blancas, 0–100 m (INBIO); Est Río Bonito, Send. Río Bonito, 1.4 km O Cerro Gamba, 200–300 m (INBIO); A. C. O., Golfito, Pque Nal Corcovado, Estación Agujas, 600–700 m (INBIO); Osa, Sierpe, 0.2 km NW Estación Esquinas, 0–100 m (INBIO); Estero Guerra, Peninsula Osa, 0–100 m (INBIO); Guacimal, Finca Buen Amigo, Monteverde, 4 km S de la Reserva, 1000–1100 m (INBIO); A. C. A., Central Reserva Bosque Eterno de los Niños, 1500–1600 m (INBIO); Est La Casona, Las Torres, 1500–1600 m (INBIO). San José- C. Nara, NE Quepos, 16 July 1975 (BYUC); Estación Bijagual, 1.5 km N Bijagual, 400–500 m (INBIO). **PANAMA:** Bocas del Toro- 6 km N Punta Peña, 27 May 1993 (CDFA); Reserva La Fortuna, 26 May 1993 (EGRC). Chiriquí- Bugaba, 800–1500 ft. (AMNH, DEI); Fortuna, 17 May 1978, 19 May 1978 (EGRC); Reserva Fortuna, Continental Divide Trail, 25 May 1993, 26 May 1993 (CDFA); Reserva Fortuna, Fortuna Dam, 29 May 1993 (CDFA); Reserva La Fortuna, Hydrographic sta. trail, 26 May 1993 (EGRC). Total: 254.

#### Cephaloleia chevrolatii

Taxon classificationAnimaliaColeopteraChrysomelidae

Baly, 1858

http://species-id.net/wiki/Cephaloleia_chevrolatii

Fig. 96

Cephalolia chevrolati Baly 1858: 61. Gemminger and Harold 1876: 3601 (catalog); Weise 1911a: 7 (catalog), 1911b: 12 (catalog).
Cephaloleia chevrolatii Baly. Baly 1885: 18 (distribution); Blackwelder 1946: 718 (catalog); Papp 1953: 15 (catalog); Uhmann 1957a: 16 (catalog); Wilcox 1983: 136 (catalog); Staines 1996: 22 (Central America species); Staines and Staines 1999: 523 (Baly species list).

##### Description.

Elongate; small; subparallel; subdepressed; head, antennae, and scutellum black; pronotum red with anterior margin black; elytra dark with pale margins and indistinct reddish macula at humerus; venter with prosternum reddish laterally, meso- and metasterna black, abdominal sterna black; leg yellow with black markings. Head: vertex densely punctate, medial sulcus absent; frons not projecting; not depressed between eyes. Antenna: ½ body length; slender; antennomere 1 transverse; 2 transverse, longer than 1; 3 elongate, longer than 1 or 2; 4–10 transverse, each subequal in length to 2; 11 pointed at apex, subequal in length to 3; 1–3 punctate with scattered setae; 4–11 setose. Pronotum: quadrate, widest before middle; lateral margin straight then rounding to anterior angle, canaliculate; anterior angle rounded, obtuse, produced; posterior angle acute; anterior margin emarginate behind head; disc subconvex; surface deeply but sparsely punctate, disc impunctate; basal impression absent; pronotal length 0.7–0.9 mm; pronotal width 1.0 mm. Scutellum: pentagonal; impunctate. Elytron: lateral margin straight, smooth, margined; apex rounded; sutural angle without tooth; humerus rounded, not produced; slightly constricted behind humerus; subconvex, slightly flattened on disc; finely punctate-striate, lateral striae deeply impressed; elytral length 2.4–2.7 mm; elytral width 1.2–1.3 mm. Venter: pro-, meso-, and metasterna punctate; abdominal sterna punctate, each puncture with pale seta; suture between sterna 1 and 2 complete; last sternite with apical margin broadly emarginate and sinuate medially in male, rounded, entire in female. Leg: slender; punctate; femur robust; tibia with fringe of setae on inner margin of apex. Total length: 3.4–3.5 mm.

##### Diagnosis.

This species is similar to *Cephaloleia elegantula* and *Cephaloleia partita*. It can be distinguished by antennomere 1 being transverse.

##### Distribution.

Mexico.

##### Type material examined.

Holotype: Type H. T. [white disk with red border]/ Type [green disk]/ Cephaloleia chevrolatii Baly [folded]/ 67–56 [reversed] (BMNH).

##### Specimens examined.

**MEXICO:** no further data (DEI). Total: 1.

#### Cephaloleia chica

Taxon classificationAnimaliaColeopteraChrysomelidae

Staines sp. n.

http://zoobank.org/0A11252C-7FF8-4FD4-AEB8-3435FE7B95EC

http://species-id.net/wiki/Cephaloleia_chica

Fig. 97

##### Description.

Small; elongate; subdepressed; head, scutellum, and antennae (except basal 2 antennomeres paler) black; pronotum yellow with black triangular macula behind head; elytra black with lateral and apical margins yellow and yellow vitta from basal margin to apical ⅓ on puncture rows 2–5; venter and legs dark yellowish. Head: vertex punctate, medial sulcus absent; eye large; frons not projecting; not depressed between eyes. Antenna: reaches to humerus; slender; antennomere 1 incrassate, elongate; 2–10 subequal in length, each ¾ length of 1; 11 3× length of 10, pointed at apex; 1–2 punctate with scattered setae; 3–11 setose. Pronotum: subquadrate; lateral margin straight then rounding to anterior angle, canaliculate; anterior angle rounded, not produced; posterior angle acute; anterior margin straight; disc subconvex; surface coarsely, irregularly punctate; transverse basal impression present; pronotal length 0.7 mm; pronotal width 1.0 mm. Scutellum: pentagonal; impunctate. Elytron: lateral margin straight, smooth, margined; apex weakly rounded; sutural angle without tooth; humerus rounded, not produced; slightly constricted behind humerus; moderately punctate-striate; elytral length 2.9; elytral width 1.1 mm. Venter: pro-, meso-, and metasterna impunctate; abdominal sterna punctate, each puncture with white seta; suture between abdominal sterna 1 and 2 obsolete medially; last sternite with apical margin shallowly emarginate. Leg: slender; femur and tibia punctate, each puncture with seta; tibia with fringe of setae on inner margin of apex. Total length: 3.9 mm.

##### Etymology.

From chico (Spanish) meaning little or little one for the small size of this species. The name is feminine.

##### Diagnosis.

This species is similar to *Cephaloleia convexifrons*. It can be distinguished by the small body size and the triangular macula on the anterior margin of the pronotum behind the head.

##### Distribution.

Peru.

##### Type material.

Holotype: Peru, Dept. Loreto, Explorama Lodge, 80 km NE Iquitos on Amazon River, VI-24/VII-20 1990, Menke and Awertschenko/ collected in malaise trap/ Holotype *Cephaloleia chica* Staines, des. C. L. Staines 2012 [red label] (USNM).

#### Cephaloleia chimboana

Taxon classificationAnimaliaColeopteraChrysomelidae

Uhmann, 1938a

http://species-id.net/wiki/Cephaloleia_chimboana

Fig. 98

Cephalolia chimboana Uhmann 1938a: 408.
Cephaloleia chimboana Uhmann. Uhmann 1957b: 16 (catalog); Gaedike and Döbler 1971: 345 (types); Staines 1997b: 413 (Uhmann species list).

##### Description.

Elongate; subparallel; subdepressed; pale yellowish brown, the apical four or five antennomeres darkened, apex of antennomere 11 pale, elytral punctures dark. Head: vertex very finely punctate, impunctate between the eyes, medial sulcus absent; frons not projecting; not depressed between eyes. Antenna: as long as head and pronotum combined; slender; antennomere 1 elongate; 2 elongate, a little shorter than 1; 3 subequal in length to 1; 4–5 elongate, subequal in length, each nearly as long as 3; 6–10 slightly transverse, slightly different in shape from each other, subequal in length, each shorter than 5; 11 elongate, 2× length of 10, bluntly rounded at the apex; 1–4 impunctate; 5–11 with setae. Pronotum: transverse; lateral margin straight and divergent for basal ⅔ then rounded and convergent, canaliculate; anterior angle broadly rounded; posterior angle acute; anterior margin emarginate behind head; disc flattened; surface with scattered, very shallow punctures, disc nearly impunctate; basal impression absent; pronotal length 1.2–1.4 mm; pronotal width 1.7–1.9 mm. Scutellum: rounded-pentagonal; impunctate. Elytron: lateral margin straight, smooth, margined; apex rounded; sutural angle without tooth; humerus slightly prominent; slightly constricted behind humerus; finely punctate-striate, punctures mostly confluent, rows dark, clearly visible for entire length; elytral length 4.3–4.7 mm; elytral width 2.1–2.4 mm. Venter: pro-, meso-, and metasterna impunctate medially, punctate laterally; abdominal sterna punctate, each puncture with pale seta; suture between sterna 1 and 2 complete; last sternite with apical margin deeply and broadly emarginate in male; last sternite with apical margin rounded in female. Leg: slender; punctate; femur robust; femur and tibia with pale seta in each puncture; tibia with fringe of setae on inner margin of apex. Total length: 6.3–6.8 mm.

**Figures 98–106.**
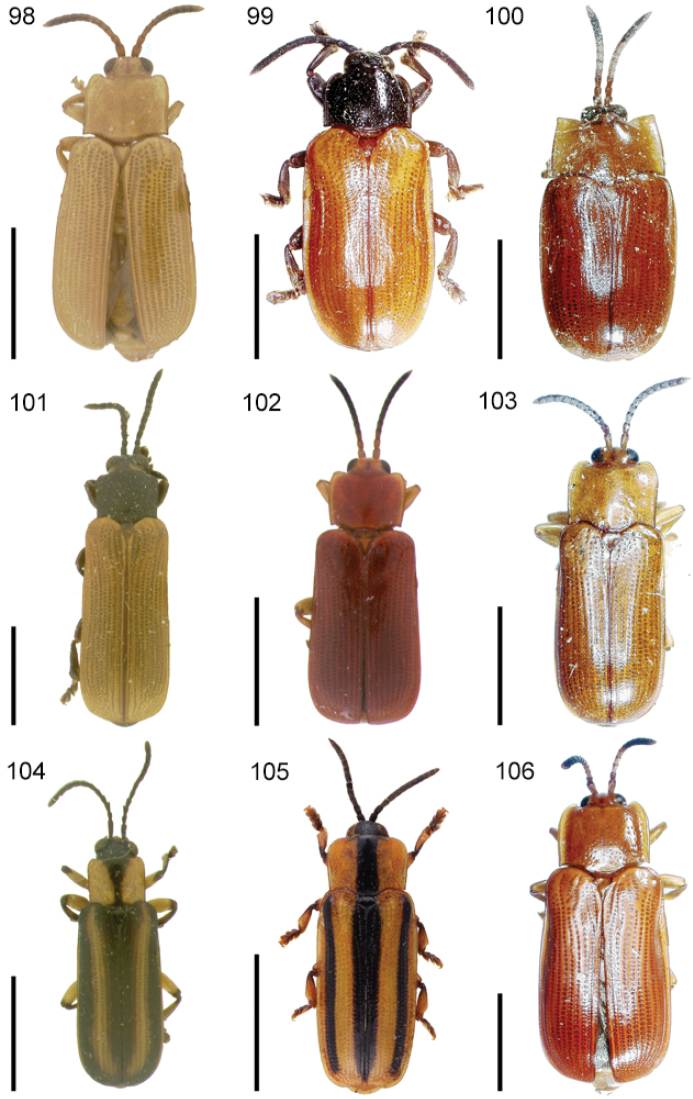
Habitus. **98** *Cephaloleia chimboana* **99** *Cephaloleia clarkella* **100** *Cephaloleia cognata* **101** *Cephaloleia collaris* **102** *Cephaloleia conforma* sp. n. **103** *Cephaloleia congener* **104** *Cephaloleia consanguinea* **105** *Cephaloleia convexifrons* **106** *Cephaloleia corallina*. Scale bars equal 3 mm.

##### Diagnosis.

This species is similar to *Cephaloleia lojaensis* and *Cephaloleia nigriceps*. It can be distinguished by the tranverse basal impression on the pronotum and by the confluent elytral punctures.

##### Distribution.

Ecuador, Peru.

##### Type material examined.

Holotype: Ecuador, Chimbo, Rosenberg [printed label]/ Holotypus [red printed label]/ Cephalolia chimboana Uh., Det. E. Uhmann (BMNH).

##### Specimens examined.

**Ecuador:** Imbabura- Cachabé, November 1896 (BMNH, USNM). Pichincha- Chimbo (DEI). **Peru:** Madre de Dios- Tambopata Wildlife Res., 30 km SW Pto. Maldanado, 26 November 1982 (USNM). Total: 6.

#### Cephaloleia clarkella

Taxon classificationAnimaliaColeopteraChrysomelidae

Baly, 1858

http://species-id.net/wiki/Cephaloleia_clarkella

Fig. 99

Cephalolia clarkella Baly 1858: 49. Gemminger and Harold 1876: 3601 (catalog); Donckier 1899: 548 (catalog); Weise 1911a: 7 (catalog), 1911b: 12 (catalog); Uhmann 1953d: 46 (faunal list).
Cephaloleia clarkella Baly. Uhmann 1957b: 16 (catalog), 1964b: 16 (faunal list); Balazuc 1988: 397 (pathogens); Staines and Staines 1999: 523 (Baly species list).

##### Description.

Elongate; subdepressed; shining; head, antennae, pronotum, and legs black, scutellum and elytra yellowish, venter yellowish. Head: vertex densely punctate, medial carina present; frons not projecting; slightly depressed between eyes. Antenna: ½ body length; slender; antennomere 1 incrassate, elongate; 2–3 elongate, subequal in length, shorter than 1; 4 ¾ length of 3, elongate; 5 shorter than 4; 6–10 transverse, subequal in length, each shorter than 5; 11 2× length of 10, acutely pointed at apex; 1–2 punctate with scattered setae; 3–11 setose. Pronotum: transverse; lateral margin sinuate at base then rounding to anterior angle, canaliculate; anterior angle rounded, produced; posterior angle acute; anterior margin emarginate behind head; disc moderately convex; surface sparsely punctate, punctures more dense laterally and basally; basal impression absent; pronotal length 1.3–1.7 mm; pronotal width 2.0–2.2 mm. Scutellum: pentagonal; sparsely punctate. Elytron: lateral margin straight, smooth, margined; apex rounded; sutural angle without tooth; humerus rounded, not produced; constricted behind humerus; slightly convex, slightly flattened along suture; punctate-striate, punctures more impressed basally and laterally; elytral length 5.3–5.8 mm; elytral width 2.9–3.2 mm. Venter: pro-, meso-, and metasterna impunctate medially, punctate laterally; abdominal sterna punctate, each puncture with pale seta; suture between sterna 1 and 2 complete; last sternite with apical margin sinuate medially in male, entire in female. Leg: slender; punctate; femur and tibia with pale seta in each puncture; tibia with fringe of setae on inner margin of apex. Total length: 7.1–7.5 mm.

##### Diagnosis.

This species is similar to *Cephaloleia luridipennis*. It can be distinguished by the pronotum lacking a transverse basal impression and by antennomere 1 being longer than 2.

##### Distribution.

Bolivia, Brazil, Ecuador, Peru.

##### Type material examined.

Syntype: Peru [handwritten label]/ Baly coll. [printed label]/ Cephalolia clarkella Baly, Peru [blue handwritten label] (BMNH, 1).

##### Specimens examined.

**Brazil:** Amazonas- Pontehoa (?) (USNM). **Ecuador:** Orellana- 1 km S Onkone Gare Camp, Reserva Etnica Waorani, 216.3 m, 9 February 1995, 1 July 1995 (USNM); Tiputini Biodiversity Station, nr. Yasuni National Park, 220–250 m, 6 February 1999 (USNM). **Peru:** no further data (USNM). Total: 5.

#### Cephaloleia cognata

Taxon classificationAnimaliaColeopteraChrysomelidae

Baly, 1869

http://species-id.net/wiki/Cephaloleia_cognata

Fig. 100

Cephalolia cognata Baly 1869: 372. Gemminger and Harold 1876: 3601 (catalog); Donckier 1899: 548 (catalog); Weise 1911a: 7 (catalog), 1911b: 11 (catalog).
Cephaloleia cognata Baly. Uhmann 1957b: 17 (catalog), 1964a: 403 (catalog), 1965: 113 (museum list); Staines and Staines 1999: 523 (Baly species list).

##### Description.

Elongate; subdepressed; pale yellow, antennae (except antennomeres 1–2 which are reddish) black; eyes dark. Head: vertex sparsely punctate, with medial sulcus; frons not projecting; not depressed between eyes. Antenna: ½ body length; slender; antennomere 1 elongate, subequal in length to 2 and 3 combined; 2 transverse, ⅓ length of 1; 3 transverse, ½ length of 2; 4–10 transverse, subequal in length, each longer than 2; 11 2× length of 10, rounded at apex; 1–4 punctate with scattered setae; 5–11 setose. Pronotum: subquadrate; lateral margin straight then rounding to anterior angle, slightly canaliculate; anterior angle angulate, produced; posterior angle acute; anterior margin emarginate behind head; disc subconvex, flattened laterally; surface sparsely punctate; basal impression absent; pronotal length 1.3–1.5 mm; pronotal width 2.3–2.7 mm. Scutellum: pentagonal; impunctate. Elytron: lateral margin straight, smooth, slightly laminate; apex rounded; sutural angle without tooth; humerus rounded, not produced; constricted behind humerus; disc flattened; punctate-striate, punctures smaller and less impressed apically; interspace behind humerus costate for ½ length; elytral length 4.2–4.4 mm; elytral width 2.7–3.1 mm. Venter: pro-, meso-, and metasterna impunctate medially, punctate laterally; abdominal sterna punctate, each puncture with seta; suture between sterna 1 and 2 complete; last sternite with apical margin bisinuate in female, emarginate medially in male. Leg: slender; punctate; femur robust; tibia with fringe of setae on inner margin of apex. Total length: 5.6–6.8 mm.

##### Diagnosis.

This species is similar to *Cephaloleia aequilata* and *Cephaloleia dilaticollis*. It can be distinguished by the elytra with a costate interspace behind the humerus.

##### Host plant.

Accodring to data adults have been collected from *Calathea lutea* Schult. (Marantaceae).

##### Distribution.

Brazil (Bahia), Peru, Venezuela.

##### Type material.

Type: Brazil, Bahia, BMNH, not seen.

##### Specimens examined.

No label data (USNM). **Brazil:** Bahia- no further data (BMNH). **Peru:** Madre de Dios- CICRA Field Station, 272 m, 13 June 2011 (SEMC). **Venezuela:** Merida- 6 km E. Las Cruces, 13 July 1986 (BYUC). Total: 6.

#### Cephaloleia collaris

Taxon classificationAnimaliaColeopteraChrysomelidae

Weise, 1910

http://species-id.net/wiki/Cephaloleia_collaris

Fig. 101

Cephaloleia collaris Weise 1910: 90. Weise 1911a: 7 (catalog), 1911b: 12 (catalog).
Cephaloleia collaris Weise. Uhmann 1957b: 17 (catalog).

##### Description.

Subparallel; slender; subconvex; shining; black; elytra and scutellum yellowish-brown; venter and legs yellowish-brown. Head: vertex irregularly punctate, medial sulcus present; frons not projecting; not depressed between eyes. Antenna: reaches to humerus; slender; antennomere 1 cylindrical, thick, longest, 2× length of 2; 2 transverse; 3 cylindrical, elongate, shorter than 1; 4–5 cylindrical, elongate, shorter than 3; 6–10 subequal in length, transverse, subequal in length to 2; 11 2× length of 10, pointed at apex; 1–2 punctate with scattered setae; 3–11 setose. Pronotum: transverse; lateral margin straight then rounding to anterior angle, narrowly margined; anterior angle weakly rounded, slightly produced; posterior angle acute; anterior margin emarginate behind head; disc subconvex; surface densely punctate basally and laterally; transverse basal impression present medially; pronotal length 1.3–1.5 mm; pronotal width 1.8–2.0 mm. Scutellum: elongate pentagonal; impunctate. Elytron: lateral margin straight, smooth, narrowly margined; apex rounded; sutural angle without tooth; humerus rounded, not produced; slightly constricted behind humerus; moderately punctate-striate, scutellar row long, punctures confused basally and apically; elytral length 6.0–6.5 mm; elytral width 2.3–2.5 mm. Venter: pro-, meso-, and metasterna impunctate medially, sparsely punctate laterally; abdominal sterna punctate, each puncture with white seta; suture between sterna 1 and 2 obsolete medially. Leg: slender; sparsely punctate; femur and tibia with pale seta in each puncture; tibia with tuft of setae at apex. Total length: 7.9–8.3 mm.

##### Diagnosis.

This species is similar to *Cephaloleia flavipennis* and *Cephaloleia neglecta*. It can be distinguished by the basal impression on the pronotum and by the margined lateral margin of the pronotum.

##### Distribution.

Colombia, Venezuela.

##### Type material examined.

Syntypes: Colombia, in der Terra templads Historaques, 6000 ft., Thieme [Green printed label]/ J. Weise det. [printed label]/ Type [printed salomon-colored label]/ Cephalolia collaris m [handwritten label] (ZMHB, 2).

##### Specimens examined.

**Venezuela:** Aragua- PN H. Pittier, Rancho Grande, Portochioula, 1120 m, 12 July 1998 (USNM); Nov Grande, 10 February 1877 (USNM). Total: 1.

#### Cephaloleia conforma

Taxon classificationAnimaliaColeopteraChrysomelidae

García-Robledo & Staines sp. n.

http://zoobank.org/E9ECFE96-71A5-4F69-8262-210E5931DCFF

http://species-id.net/wiki/Cephaloleia_conforma

Fig. 102

##### Description.

Elongate; subparallel; subdepressed; reddish-brown; antennae (except basal 2 antennomeres) and eyes black; venter and legs paler. Head: vertex finely, irregularly punctate; medial sulcus present; not depressed between eyes; frons punctate, not projecting. Antenna: reaches to humerus; antennomere 1 subincrassate, 2× length of 2; 2–4 cylindrical, 3 slightly longer than 2, 4 subequal in length to 2; 5–10 transverse, subequal in length; 11 2× length of 10, rounded at apex; 1–2 punctate with scattered setae; 3–11 setose. Pronotum: transverse; lateral margin straight, divergent for basal ¾ then rounding to anterior angle, canaliculate; anterior angle rounded; posterior angle acute; anterior margin emarginate behind head; disc depressed; surface sparsely, irregularly punctate; basal impression absent; pronotal length 1.3–1.4 mm; pronotal width 1.7–1.8 mm. Scutellum: pentagonal; impunctate. Elytron: lateral margin straight, smooth, margined; apical margin smooth, narrowly margined; sutural angle without tooth; humerus rounded, slightly produced; slightly constricted behind humerus; moderately punctate-striate, punctures obovate, scutellar row long, rows confused at apex; elytral length 4.0–4.2 mm; elytral width 2.3–2.4 mm. Venter: pro-, meso-, and metasterna impunctate medially, punctate laterally; prosternum with pale seta in each puncture; abdominal sterna finely punctate, each puncture with pale seta; sutural between sterna 1 and 2 obsolete medially; apical margin of last sternite emarginate medially in male, sinuate in female. Leg: robust; punctate; tibia with fringe of setae on inner apical margin. Total length: 6.2–6.6 mm.

##### Etymology.

From conformis (Latin) meaning like or resembling, since the species resembles *Cephaloleia congener* and *Cephaloleia sallei*. The name is a noun in apposition.

##### Diagnosis.

This species is similar to *Cephaloleia calathae* and *Cephaloleia erichsonii*. It can be distinguished by the elytra lacking a sulcus on the humeral callus, by antennomere 1 being clavate and twice the length of 3, and by the elytral punctures not being larger on the disc.

##### Host plant.

Adults have been collected off *Calathea* sp., *Cephaloleia guzamanioides* LB Sm and Idrobo (Marantaceae).

##### Distribution.

Costa Rica.

##### Type material.

Holotype male: Costa Rica, Puntarenas, Coto Brus, Las Cruces Biol. Station, 1200 m, GC-MAY-11-72A, 10 March 2012, C. García-Robledo, ex. *Calathea guazmanioides*/ Holotype *Cephaloleia conforma* García-Robledo & Staines, des. 2012 [red label] (USNM). Paratypes (6 males, 3 females) (each with Paratype *Cephaloleia conforma* García-Robledo & Staines, des. 2012 [red label]): same label data as holotype except GC-MAY-11-72, GC-MAY-11-72B, GC-MAY-11-72C, GC-MAY-11-72D, GC-MAY-11-43, GC-MAY-11-43A, GC-MAY-11-43B; same label data as holotype except GC-MAY-11-26, ex *Zingiber spectabile*; same label data as holotype except no GG number, ex. *Calathea* sp. (USNM, IEXA).

#### Cephaloleia congener

Taxon classificationAnimaliaColeopteraChrysomelidae

Baly, 1885

http://species-id.net/wiki/Cephaloleia_congener

Fig. 103

Cephaloleia congener Baly 1885: 12. Blackwelder 1946: 718 (catalog); Papp 1953: 15 (catalog); Uhmann 1957a: 17 (catalog); Wilcox 1983: 136 (catalog); Staines 1996: 22 (Central America species), 2004: 312 (host plants), 2011: 48 (faunal list); Maes 1999: 1016 (faunal list); Staines and Staines 1999: 523 (Baly species list); McKenna and Farrell 2005: 119 (phylogeny), 2006: 10949 (phylogeny); García–Robledo et al. 2013a: 3 (biology), 2013b: 193 (biology).
Cephalolia congener Baly. Donckier 1899: 548 (catalog); Weise 1911a: 7 (catalog), 1911b: 11 (catalog).

##### Description.

Oblong-ovate; shining; subconvex; light reddish-brown, antennae (except basal antennomere), and eyes darker. Head: small; vertex and front finely punctate, medial sulcus absent; frons not projecting; not depressed between eyes. Antenna: less than ½ body length; slender; antennomere 1 elongate, subequal in length to 3; 2 transverse in female, triangular in male, ½ length of 1; 3 triangular in male, elongate in female; 4–10 elongate, decreasing in length, each shorter than 3; 11 2× length of 10, pointed at apex; 1–2 punctate with scattered setae; 3–11 setose. Pronotum: transverse; lateral margin straight, diverging from base to middle, then rounding to anterior angle, margined; anterior angle obtuse, slightly produced; posterior angle acute; anterior margin emarginate behind head; disc subconvex; surface finely punctate; basal impression absent; pronotal length 1.3–1.4 mm; pronotal width 2.0–2.4 mm. Scutellum: scarcely longer than wide; pentagonal; impunctate. Elytron: lateral margin sinuate before middle, smooth; apex rounded; sutural angle without tooth; humerus rounded; slightly constricted behind humerus; convex; finely punctate-striate; declivity beginning just behind humerus at puncture row 7 edged with faint carina; elytral length 5.0–5.4 mm; elytral width 2.7–3.1 mm. Venter: pro-, meso-, and metasterna impunctate medially, punctate laterally; abdominal sterna punctate, each puncture with pale seta; suture between sterna 1 and 2 complete; last sternite with apical margin emarginate medially in male, truncate in female. Leg: slender; femur robust; tibia expanded to apex, with fringe of setae on inner margin of apex. Total length: 6.6–7.4 mm.

##### Diagnosis.

This species is similar to *Cephaloleia immaculata*. It can be distinguished by the finely punctate pronotum, by the suture between abdominal sterna 1 and 2 being complete, and by antennomere 1 being subequal in length to 3.

##### Host plant.

Adults have been collected from *Heliconia latispatha* Bentham and *Heliconia tortuosa* Griggs (Heliconiaceae) (Staines 1996); *Heliconia imbricata* Baker, *Heliconia irrasa* R. R. Sm., *Heliconia mathiasiae* G. S. Daniels and F. G. Stiles, *Heliconia psittacorum* Sw., *Heliconia pogonantha* Cufod., *Heliconia sarapiquensis* G. S. Daniels and F. G. Stiles, *Heliconia wagneriana* Peterson, *Calathea crotalifera* S. Watson, *Cephaloleia inocephala* Juntze, *Ischnosiphon inflatus* L. Andersson (Marantaceae), *Musa velutina* H. Wendl. and Drude (Musaceae) (García–Robledo et al. 2013a).

##### Distribution.

Costa Rica, Guatemala, Nicaragua, Panama.

##### Type material examined.

Holotype: Type H. T. [white disk with red border]/ Panama, Bugaba, Champion [printed label]/ B. C. A., Col., VI, 2. Cephaloleia congener Baly [printed label]/ Cephaloleia congener Baly, Panama [blue handwritten label] (BMNH).

##### Specimens examined.

**COSTA RICA:** Alajuela- Est. Biol. Alberto Brenes, 20 June–06 July 1999 (BYUC); Caño Negro, 20 m, R.N.V.S. Caño Negro, 4–17 December 1992 (INBIO); Río San Lorencito, 900 m, Res. For. San Ramón, 5 km N Col. Palmarena, March 1990, 13–18 June 1993 (INBIO); N slope Volcán de Rincón, 2 km W Dos Ríos, 22 May 1985 (EMEC); Upala, Dos Ríos, 31 March 1988 (MUCR); 20 km S Upala, 16–25 September 1990, 1–25 September 1991 (BYUC); A. C. G., Upala, Aguas Claras, Pque Nal Rincón de la Vieja, 600–700 m (INBIO). Cartago- Aquiares nr. Santa Cruz, 9 km NW Turrialba, 1500 m, 16 May 1985 (EMEC); CATIE, 3 km SE Turrialba, 600 m, 15 May 1985, 29 May 1985 (EMEC); Quebrada Segunda, P. N. Tapantí, 1250 m, April 1992, August 1992 (INBIO); Ref. Nac. Fauna Silv. Tapantí, 1250 m, August 1991 (INBIO); Turrialba (USNM), April (DEI), 4–13 August 1970, 30 May 1973 (USNM), 26–29 June 1986, 20 August 1989 (BYUC); nr. Tuis, 16–22 July 1993 (BYUC). Guanacaste- Estac. Pitilla, 700 m, 9 km S Santa Cecilia, 1988, January 1989, 27 January-4 February 1989, 21 March–21 April 1989, September 1989, December 1989, February 1990, March 1990, July 1991, August 1991, 18 April–9 May 1993 (INBIO); Hda. Sta. Maria, 2 February 1993 (INBIO); Río San Lorenzo, 1050 m, R. F. Cord, March 1990, April 1991, November 1991, 10–20 February 1992, April 1992, July 1992 (INBIO); 3 km SE Río Naranjo, 14–20 August 1993 (BYUC). Heredia- Est. El Ceibo, Braulio Carillo N.P., 400–600 m, November 1989, March 1990, April 1990 (INBIO); Chilamate, Thomas Ray Property, 7 January 1990 (UMMZ); Finca Naranjo Valencia, 2 km sur Pueblo Nuevo, Sarapiquí, 90 m, 9–30 September 1992 (INBIO); 1 km S. Pt. Viejo, 4–5 June 1984 (EGRC); Est San Rafael Vara Blanca, P.N. Braulio Carillo, 1800–2000 m, April 1990 (INBIO); Est. Biol. La Selva, 21 January 1989 (MUCR), 14–15 August 1991 (BYUC), 15 April 2003, 2 March 2005 (USNM); Fca. La Selva nr. Puerto Villa, 5 August 1969 (USNM), 19 July 1985 (UMMZ), 7 September 1988 (UMMZ), 30 March 1990 (INBIO); El Plastico Station, 4 July 2011 (USNM); 11 km SE La Virgen, 450–500 m, 12 April 2003 (USNM); Rara Avis Biological Station, 5 July 2011, 8 November 2011, 13 November 2011 (USNM). Limón- Amubri, 70 m, Talamanca, 1–22 July 1992 (INBIO); Cerro Tortuguero, P. N. Tortuguero, April 1989, August 1992, December 1992 (INBIO); CATIE, Turrialba, 600 m, S. Espavals, 10 September 1998 (BYUC); Cuatro Esquinas, P. N. Tortuguero, April 1989, October 1989, November 1989, December 1989, 20 September–7 October 1990, September 1990, 27 March–29 April 1992, November 1992 (INBIO); 7 mi N Guacimo, 22 February–3 March 1988 (BYUC); 7 km W. Guápiles at Río Toro Amarillo, 22 August 1984 (UMMZ); Hamburg Farm, Reventazón, Ebene Limón, 24 January 1931, 1 February 1932, 15 January 1936 (USNM), 1 February 1932 (DEI); Est. Hitoy Cerere, 100 m, R. Cerere Res. Biol. Hitoy Cerere, 4–20 December 1991, 19–29 April 1992, 30 June–20 July 1992, 15-27 February 1993 (INBIO); Manzanillo, 0-100 m, RFNS Gandoca y Manzanillo, 22 October–11 November 1992 (INBIO); Río Sardinas, 10 m R.N.F.S. Barra del Colorado, September 1992 (INBIO); Salvadora Farm, Parismina, 5 September 1930 (USNM); Sector Cerro Cocorí, Fca. de E. Rojas, 150 m, 31 January–21 February 1992, 26 March–24 April 1992, April 1992, October 1992, 9–30 November 1992, January 1993, February 1993, March 1993, April 1993 (INBIO); Valle La Estrella (INBIO); Pococí, Colorado, Sector Cerro Cocorí, 100–200 m (INBIO). Puntarenas- Alajuela Peñas Blancas, 800 m, 19 May 1989 (SEMC); Est. Biol. Las Alturas, 2 km NE Alturas, 1520 m, 10 July 1999 (CMNC); Barranca site, 10 km N. Puntar., 17 June 1969 (USNM); Estación Boscosa, Peninsula de Osa, 15 September 1991 (INBIO); Est Biol. Las Alturas, 1500 m, Coto Brus., October 1991, 23 March–2 May 1992, August 1992, 3–4 September 1992, November 1992 (INBIO), 23–26 May 1992 (AJGC); Fca. Cafrosa, Est Las Mellizas, P.N. Amistad, November 1989, May 1990 (INBIO); Fca. Las Cruces, San Vito de Java, 27 June 1969 (USNM); Monteverde Cloud For. Res., 27–31 May 1984 (EGRC); Monteverde Cloud For. Res., 1500 m, 20 May 1985 (EMEC); Est. Queb. Bonita, 50 m, Res. Biol. Carara, 17 March–30 April, April 1992 (INBIO), 11 August 1991 (BYUC); Peninsula de Osa, 22 July 1960 (MUCR); Rancho Quemado, Pen Osa, April 1991, July 1991, October 1991, 21 March–7 April 1992, April 1992, May 1992, October 1992, December 1992 (INBIO); 3.5 mi. S. Rincón, Osa Peninsula, 28 February–12 March 1969 (CASC); San Vito-Villa Nielly area, 13 August 1969 (USNM); San Vito, Est. Biol. Las Alturas, May 1992 (BYUC); Est Sirena, Corcovado NP, December 1989, October 1989, November 1989, January 1990, February 1990, March 1990, April 1990, May 1990, June 1990, September 1990, October 1990, June 1991, September 1991, January 1992, April 1992 (INBIO); Wilson Botanical Garden (Las Cruces Biol. Stn.) nr. San Vito, 1200 m, 26 May 1993 (SEMC). San José- San Isidro, 9 mi S, 31 December 1988 (BYUC); Est. Boscoas, 0–100 m (INBIO); km 117 Pan American Hwy, 19 km N San Isidro, 20–25 June 1997 (SEMC). **GUATEMALA:** no further data (USNM). **NICARAGUA:** no further data (USNM). **PANAMA:** no further data (USNM). Chiriquí- 10 mi W. Boquete, 14 March 1960 (BYUC); 11.2 km E Chiriquí, 30 May 1993 (AJGC); Fortuna, 17 May 1978 (USNM); Reserva Fortuna, Fortuna Dam, 29 May 1993 (CDFA); 27.7 km NE Volcán Hartmann’s Finca, 1450 m, 18 June 1996 (USNM); Las Laguna, El. 1360 m., 4 km W. Hato del Volcán, 24 May 1973 (EGRC). Coclé- Cerro Gaital, 4000', 1 May 1993 (AJGC). Colón- Reserva Sobrina, Pipeline Road, 23 May 1993, 6 May 1993 (AJGC); Porto Bello, 18 February 1911, 19 February 1911, 27 February 1911, 6 March 1911 (USNM). Darien- Estación Ambiental Cana, 525–750 m, 3–10 June 1996 (USNM). Panamá- Cerro Campana, 3000 ft., 1 August 1979 (CMNC); Old Gamboa road, 4 June 1993 (AJGC). Total: 449.

#### Cephaloleia consanguinea

Taxon classificationAnimaliaColeopteraChrysomelidae

Baly, 1885

http://species-id.net/wiki/Cephaloleia_consanguinea

Fig. 104

Cephaloleia consanguinea Baly 1885: 23. Blackwelder 1946: 718 (catalog); Papp 1953: 15 (catalog); Uhmann 1957a: 17 (catalog); Wilcox 1983: 136 (catalog); Seifert 1982: 11; Staines 1996: 23 (Central America species), 1999: 241 (mimicry), 2010: 29 (types), 2011: 48 (faunal list); Staines and Staines 1997: 6 (types), 1999: 523 (Baly species list); Flowers and Hanson 2003: 50 (distribution); Jolivet 1989: 301 (noted), 2003: 313 (noted); McKenna and Farrell 2005: 119 (phylogeny); Naczi and Staines 2011: 2 (faunal list).
Cephalolia consanguinea Baly. Donckier 1899: 548 (catalog); Weise 1911a: 7 (catalog), 1911b: 11 (catalog); Uhmann 1930a: 233 (faunal list), 1936b: 484 (key), 1942: 93 (noted).

##### Description.

Elongate; flattened; head (except yellow frons), antennae, and scutellum black; pronotum yellow with medial black macula narrowing toward base, macula extremely variable in shape; elytra black with medial yellow vitta on each elytron, vitta begins at humerus and goes to apical 1/5, from puncture row 6 to puncture row 7 or 8 (variable in width), narrower at base, widens slightly, does not follow puncture rows entire length; venter yellow medially, black laterally; leg tibia black; femur yellow basally, black apically. Head: vertex strongly, densely punctate, Y-shaped medial sulcus present; frons with some large punctures, not projecting; not depressed between eyes. Antenna: reaches to humerus; slender; antennomere 1 elongate, robust, longest, flattened laterally, fringe of setae at apex; 2 elongate, ¼ length of 1; 3 triangular, 1½ length of 2; 4–6 elongate, each shorter than 3; 7–10 transverse, each shorter than 6; 11 2× length of 10, bluntly pointed; 1–4 punctate with scattered setae; 5–11 setose. Pronotum: transverse; lateral margin straight then rounding to anterior angle, margined; anterior angle acute, slightly produced; posterior angle acute; anterior margin emarginate behind head; disc flattened; surface with scattered large punctures laterally and basally; basal impression absent; pronotal length 1.1–1.3 mm; pronotal width 1.5–1.6 mm. Scutellum: triangular; impunctate. Elytron: lateral margin straight, smooth, margined; apex rounded; sutural angle without tooth; humerus rounded, slightly produced; slightly constricted behind humerus; moderately punctate-striate; elytral length 4.1–5.1 mm; elytral width 1.9–2.1 mm. Venter: pro-, meso-, and metasterna impunctate; abdominal sterna punctate, each puncture with pale seta; suture between sterna 1 and 2 complete; last sternite with apical margin truncate medially in male; weakly rounded medially in female. Leg: slender; impunctate; tibia with fringe of setae on inner margin of apex. Total length: 5.7–7.0 mm.

##### Diagnosis.

This species is similar to *Cephaloleia belti*, *Cephaloleia erugatus*, *Cephaloleia semivittata*, *Cephaloleia triangularis*, *Cephaloleia trivittata*, *Cephaloleia variabilis*, and *Cephaloleia vittata*. It can be distinguished by the elytral punctures being distinct apically and by antennomere 1 being as long as 2 and 3 combined.

##### Host plant.

*Heliconia imbricata* (Strong 1977a), *Heliconia latispatha* Bentham (Strong 1977b), *Heliconia pogonantha* Cuford., *Heliconia mariae* Hook., *Heliconia tortuosa* Griggs (Strong 1982a); *Heliconia bourgaeana* Peterson, *Heliconia collinsiana* Griggs, *Heliconia wagneriana* Peterson (Naczi and Staines 2011); *Heliconia longa* H. J. P. Winkl., *Heliconia nutans* Woodson, *Heliconia stilesii* W. J. Kress, *Heliconia wilsonii* G. S. Daniels and F. G. Stiles (Heliconiaceae), *Calathea crotalifera* S. Watson, *Cephaloleia guzmanioides* L. B. Sm. and Idrobo (Marantaceae), *Musa ornata* Roxb., *Musa velutina* H. Wendl. and Drude (Musaceae).

##### Distribution.

Belize, Costa Rica, Guatemala, Panama.

##### Type material examined.

Lectotype: Cubilguitz, Vera Paz. Champion [printed label]/ B. C. A., Col., VI, 2. Cephaloleia consanguinea Baly label]/ Lectotype Cephaloleia consanguinea Baly Des. C. L. Staines 1993 [red label] (BMNH).

##### Specimens examined.

**Belize:** Toledo- ca 9 mi NNE Medina Bank, N side Bladen Branch, 6 January 2006, 4 January 2007 (USNM); Belize Foundation for Research and Environmental Education property, 4 January 2007 (USNM). **COSTA RICA:** Cartago- Ref. Fauna Silv. Tapantí, 1650 m, Repressa Río Gde. de Orosí, August 1991 (INBIO); Quebrada Segunda, Ref. Nac. Fauna Tapantí, 1250 m, April 1992, August 1992 (INBIO); El Guarco, San Isidro, 4 km S Cañón, 2200–2300 m (INBIO); Chirripo, Turrialba, 1100–1200 m (INBIO). Guanacaste- R. Sn. Lorenzo, 1050 m, Tierras Morenas, R. F. Cord., October 1991, January 1992, April 1992 (INBIO). Heredia- El Angel falls, Vara Blanca area, 21 June 1969 (USNM); Fca. La Selva nr. Puerto Viejo, Sarapiquí Dist., 21 June 1969, 22 June 1969, 24 July 1969 (USNM); Piedras Negras (USNM). Limón- Amubri, 70 m, Talamanca, 16–31 August 1992, 12–30 September 1992, 12–29 November 1992, 5–26 January 1993 (INBIO); 4 km NE Bribri, December 1989, March 1990 (MUCR); 5 mi S Cahuita, 23–26 December 1988 (BYUC); Est. Hitoy Cerere, 100 m, R. Cerere, Res. Biol. Hitoy Cerere, September 1990, May 1991, July 1991, 4–20 December 1991, 5–19 March 1992, 19–29 April 1992, 27 June–22 July 1992, November 1992, 15–27 February 1993 (INBIO); Limón, 5 February 1989 (MUCR); Manzanillo, 0–100 m RNFS Gandoca y Manzanillo, 7–19 August 1992, 9 September–13 October 1992, 22 October–11 November 1992, 4–12 December 1992, 6–27 January 1993 (INBIO); Valle de la Estrella Valle de Posas, nr. Pandora, 17 February 1984, 17–20 February 1984 (CMNC); A. C. Llanuras del Tortuguero, Pococí, 0–100 m (INBIO). Puntarenas- Barranca site, 10 km N. Puntarenas, 17 June 1969, September 11 1969 (USNM); Coto Brus, Las Alturas, 6 March 2012 (USNM); Coto Brus, Las Cruces Biological Station, 5 March 2012, 6 March 2012, 8 March 2012, 10 March 2012 (USNM); 10.9 mi. E. Esparta, 17 June 1969 (USNM); Monteverde Reserve, 3 June 1992 (CDFA); Osa Peninsula, 0–5 m., December 1983 (CMNC); Río Piedras, sea level, 15 August 1969 (USNM); Est. Sirena, P. N. Corcovado, 0–100 m, October 1989 (INBIO); Estación Biológica Las Alturas, 1400–1500 m (INBIO); Est. La Casona, Las Torres, 1500–1600 m (INBIO). **GUATEMALA:** Alta Verapaz- Cahabón; Chiacam, Sabo (AMNH, USNM). San Antonio- Irebal sierra Espíritu Sto. Amates, 10 August 1990 (EGRC). **PANAMA:** Canal Zone- 22 August 1970 (USNM). Colon- vic. Fort Sherman, 15–16 February 1999 (USNM). Panamá- Alajuelo, 18 April 1911 (USNM); Las Cumbres, 8 January 1959 (FMNH); Ft. Kobbe, 13 October 1969 (CMNC). Total: 383.

#### Cephaloleia convexifrons

Taxon classificationAnimaliaColeopteraChrysomelidae

Pic, 1923

http://species-id.net/wiki/Cephaloleia_convexifrons

Fig. 105

Cephalolia convexifrons Pic 1923: 9.
Cephaloleia convexifrons Pic. Uhmann 1957b: 17 (catalog), 1964a: 403 (catalog); Descarpentries and Villiers 1959a: 139 (types).

##### Description.

Elongate; subconvex; antennae, head, and scutellum black; pronotum yellowish with broad medial longitudinal vitta from base to apex; elytra yellowish with broad black sutural vitta which narrows to apex and narrow black vitta from humerus to near apex; legs yellowish with darker joints and tarsi. Head: small; vertex densely punctate, medial sulcus absent; frons not projecting; not depressed between eyes. Antenna: reaches to humerus; slender; cylindrical; antennomere 1 short; 2 2× length of 1; 3 subequal in length to 2; 4–10 subequal in length, each shorter than 3; 11 2× length of 10, longest, acutely pointed at apex; 1–2 punctate; 3–11 setose. Pronotum: slightly transverse; lateral margin straight, slightly divergent for basal 4/5 then rounding to anterior angle, narrowly margined; anterior angle rounded, not produced; posterior angle acute; anterior margin emarginate behind head; disc subconvex; surface irregularly punctate; transverse basal impression present medially; pronotal length 0.8–1.0 mm; pronotal width 1.7–1.9 mm. Scutellum: broadly pentagonal; impunctate. Elytron: lateral margin straight, slightly narrowing apically, smooth, margined; apex rounded; minute tooth present in sutural angle; humerus rounded, slightly produced; shallowly punctate-striate; pygidium exposed, punctate; elytral length 4.3–4.7 mm; elytral width 2.0–2.1 mm. Venter: pro-, meso-, and metasterna punctate; abdominal sterna punctate, each puncture with pale seta; suture between sterna 1 and 2 obsolete medially; last sternite with apical margin broadly emarginate medially in male, sinuate in female. Leg: slender; punctate; femur and tibia with pale seta in each puncture; tibia with fringe of setae on inner margin of apex. Total length: 5.9–6.1 mm.

##### Diagnosis.

This species is similar to *Cephaloleia chica* sp. n. It can be distinguished by the larger size and by the medial longitudinal vitta on the pronotum.

##### Distribution.

Bolivia, Peru.

##### Type material examined.

Holotype: Bolivie, Cochabamba, Germain [green printed label]/ *Cephalolia convexifrons* m [handwritten label]/ *convexifrons* Pic (1923) [handwritten label]/ type [white printed label with red border]/ Museum Paris Coll. \M. Pic [blue printed label]/ Holotype [red printed label]/ MNHN EC 2652 [printed label] (MNHN).

##### Specimens examined.

No label data (USNM). **Bolivia:** Cochambamba- November 1953 (USNM). **Peru:** Huanuco- Tingo Maria region, 15–24 June 1937 (SEMC); Quiapicanchia- Cuzco, Quincemil, 11 June 1976 (USNM). Madre de Dios- Tambopata Wildlife Res., 30 km SW Pto Maldonado, 290 m, 15–30 November 1982 (USNM). Total: 5.

#### Cephaloleia corallina

Taxon classificationAnimaliaColeopteraChrysomelidae

Erichson, 1847

http://species-id.net/wiki/Cephaloleia_corallina

Fig. 106

Cephaloleia corallina Erichson 1847: 151. Baly 1858: 42 (redescription); Waterhouse 1881: 261 (distribution); Uhmann 1957b: 17 (catalog), 1959b: 8 (scutellum), 1964a: 403 (catalog); Staines and Staines 1999: 523 (noted).
Cephalolia corallina Erichson. Guérin–Méneville 1855: 601 (faunal list); Gemminger and Harold 1876: 3601 (catalog); Donckier 1899: 548 (catalog); Weise 1911a: 7 (catalog), 1911b: 10 (catalog); Uhmann 1936b: 109 (noted), 1936f: 481 (key), 1953d: 47 (faunal list); Soukup 1942: 317 (museum list).

##### Description.

Elongate; subdepressed; bright shining red; antennomeres 1-6 red, 7-11 black. Head: vertex smooth, faint medial sulcus present; sparsely, irregularly punctate around eye; frons not projecting; not depressed between eyes. Antenna: reaches to humerus; robust; antennomere 1 compressed, subclavate, obliquely truncate at apex, longer than 2; 2–3 in male triangularly expanded, elongate in female; 3 longer than 2; 4–10 transverse, each shorter than 2, decreasing in length; 11 2× length of 10, bluntly rounded at apex; 1–6 punctate with scattered setae; 7–11 setose. Pronotum: subquadrate; lateral margin straight then rounding to anterior angle, canaliculate; anterior angle obtuse, produced; posterior angle acute; anterior margin emarginate behind head, slightly curved; disc subconvex; surface distinctly but sparsely punctate; transverse medial basal impression present; pronotal length 2.0–2.2 mm; pronotal width 2.9–3.2 mm. Scutellum: pentagonal; impunctate. Elytron: lateral margin straight, smooth, narrowly margined; apex obtusely rounded; sutural angle without tooth; humerus rounded, not produced; constricted behind humerus; moderately convex, scarcely flattened at suture; short longitudinal sulcus present at base near humerus; moderately punctate-striate, punctures large, ovate; pygidium obtusely rounded; elytral length 6.7–7.0 mm; elytral width 3.6–4.0 mm. Venter: prosternum impunctate; meso- and metasterna impunctate medially, punctate laterally; abdominal sterna punctate, each puncture with pale seta; suture between sterna 1 and 2 obsolete medially; last sternite with apical margin rounded, sinuate at apex in male, rounded, entire in female. Leg: slender; sparsely punctate; femur robust; tibia with fringe of setae on inner margin of apex. Total length: 9.0–9.6 mm.

##### Diagnosis.

This species is similar to *Cephaloleia apicicornis*, *Cephaloleia halli*, *Cephaloleia ochra* sp. n., and *Cephaloleia proxima*. It can be distinguished by having a transverse basal impression on the pronotum.

##### Host plant.

Accodring to data adults have been collected feeding on *Calathea inocephala* (Kuntze) H. Kennedy, *Cephaloleia lutea* Schult. (Marantaceae), and *Heliconia stricta* Huber (Heliconiaceae).

##### Distribution.

Bolivia, Brazil (Amazonas, Minas Gerais, Pará, Rondonia), Colombia, Ecuador, French Guiana, Peru.

##### Type material.

Type: Peru, female, ZMHB, not seen.

##### Specimens examined.

**BOLIVIA:** Cochabamba- no further data (USNM); Chyan Villa Gnal(?) Rombain, January 1952, November 1953 (USNM). Santa Cruz- Buena Vista, 10 March 1951 (USNM). **Brazil:** no further data (AMNH). Amazonas- Manaus, October 1946 (AMNH). Bahia- São Paulo d’Olivenca (USNM). Minas Gerais- Santa Cruz de Sarra, 17 July 1985 (USNM), 21 July 1985 (USNM), 3 June 1985 (USNM); Santa Rosa, Nova Grenda, August 1878 (USNM). Pará- Val de Cans, Belem, 20–21 November 1968 (AMNH). Rondonia- 62 km SW Ariquames, Fzda Rancho Grande, 15–22 May 1991 (BYUC), 2 November 1989 (USNM), 15–22 March 1991 (USNM), 7 November 1989 (USNM), 6–15 December 1990 (EGRC, USNM), 12–22 November 1991 (CDFA), 6 October 1993 (BYUC). **COLOMBIA:** Antioquia- Río Berrío, 8 August 1938 (USNM). **ECUADOR:** Esmeraldas- 31.7 km NW Lita, 620 m, 23 August 1997 (CDFA). Los Ríos- Río Palenque, 47 km S Sto. Domingo, 220 m, 26 August 1997 (CDFA, USNM). Napo- Oriente, June 1986 (USNM). Orellana- Estación Cientifica Yasuni, 215 m, 5–10 September 1999 (EGRC). Pichincha- Chimba, 1000 ft., August 1897 (USNM). Sucumbios- Limoncocha Reserve, 215 m, 10 August 1997 (CDFA); Sushufindi, 215 m, 12 August 1997, 11 August 1997 (CDFA, USNM). **French Guiana:** Saul, 7 km N. Les Eaux Claires, 31 May 1997 (SEMC); Matcury, 42.5 km SSW on Hwy N2, 26 May 1997 (SEMC). **PERU:** Arequipa- Chancha (USNM). Ayaucho- La Mar, Santa Rosa, 640 m, 19–25 September 1976 (USNM). Huanuco- Chinchao Carpish, 25oo m, 8 September 1947, 15 September 1947 (AMNH); Leonpampa region, December 1937 (SEMC); Munson Valley, Tingo Maria, 2 November 1954 (CASC); Tingo Maria, 2200 ft., 28 December 1946, 21 October 1946 (AMNH), 670 m, 1–10 May 1937 (SEMC). Loreto- Madreselva Biol. Stn., 24 June 2005 (USNM). Madre de Dios- CICRA Field Station, 272 m, 9 June 2011, 11 June 2011, 12 June 2011, 14 June 2011 (SEMC); Rio Tambopata Reserve, 30 km (air) SW Puerto Maldonaro, 29–30 April 1988 (CASC). Total: 128.

#### Cephaloleia coroicoana

Taxon classificationAnimaliaColeopteraChrysomelidae

Uhmann, 1930c

http://species-id.net/wiki/Cephaloleia_coroicoana

Fig. 107

Cephalolia coroicoana Uhmann 1930c: 37.
Cephaloleia coroicoana Uhmann. Uhmann 1957b: 17 (catalog); Staines 1997b: 413 (Uhmann species list).

##### Description.

Elongate-ovate; subdepressed; shining; black; pronotum and elytra with lateral margins dull black; legs and epipleuron dark pitchy-black. Head: vertex distinctly, finely punctate, with narrow medial carina; frons not projecting; depressed between eyes. Antenna: as long as head and pronotum combined; slender; antennomeres 1–10 cylindrical, elongate; 1 not longer than 2; 2 and 3 subequal in length; 4–10 shorter than 3, elongate; 11 2× length of 10, acutely pointed at apex; 1–2 punctate with scattered setae; 3–11 setose. Pronotum: transverse; lateral margin straight then rounding to anterior angle, narrowly margined; anterior angle broadly rounded, projecting; posterior angle acute; anterior margin emarginate behind head; disc subconvex; surface sparsely punctate, larger punctures present medially; basal impression absent; pronotal length 1.0–1.2 mm; pronotal width 1.0–1.3 mm. Scutellum: pentagonal; impunctate. Elytron: lateral margin straight, smooth, broadly margined; apex rounded; sutural angle without tooth; humerus rounded, not produced; slightly constricted behind humerus; moderately punctate-striate, punctures may be confused; elytral length 3.4–3.6 mm; elytral width 1.8–2.0 mm. Venter: pro-, meso-, and metasterna punctate; abdominal sterna punctate, each puncture with pale seta; suture between sterna 1 and 2 complete. Leg: slender; punctate; femur and tibia with pale seta in each puncture; femur with fringe of setae on inner margin; tibia with fringe of setae on inner margin of apex. Total length: 4.9–5.0 mm.

**Figures 107–115.**
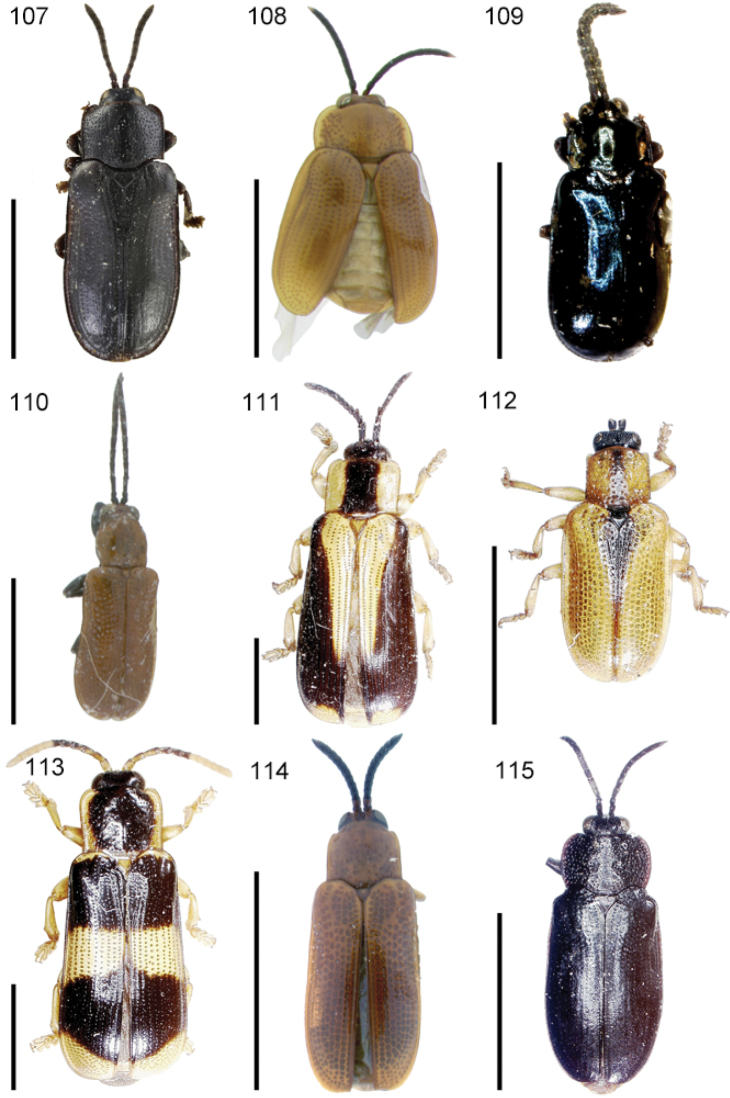
Habitus. **107** *Cephaloleia coroicoana* **108** *Cephaloleia crenulata* sp. n. **109** *Cephaloleia cyanea* **110** *Cephaloleia cylindrica* **111** *Cephaloleia daguana* **112** *Cephaloleia deficiens* **113** *Cephaloleia degandei* **114** *Cephaloleia delectabilis* **115** *Cephaloleia deplanata*. Scale bars equal 3 mm.

##### Diagnosis.

This species is similar to *Cephaloleia deplanata*, *Cephaloleia fiebrigi*, *Cephaloleia marantae*, and *Cephaloleia rufipes*. It can be distinguished by the pronotum without a basal impression.

##### Distribution.

Bolivia (Bahia), Brazil, Venezuela.

##### Type material examined.

Holotype: Bolivia, Coroico [handwritten label]/ Holotypus [printed red label]/ Cephalolia coroicana Uh., Det. E. Uhmann (NHMW).

##### Specimens examined.

No locality label (USNM). **BRAZIL:** Bahia- San Antonio de Bara, 1890 (USNM). **Venezuela:** Aragua- Rancho Grande Biological Station, 1300 m, 12–14 May 1998 (SEMC). Total: 3.

#### Cephaloleia crenulata

Taxon classificationAnimaliaColeopteraChrysomelidae

Staines sp. n.

http://zoobank.org/7129312B-E74E-468F-89A6-20334EFA1B56

http://species-id.net/wiki/Cephaloleia_crenulata

Fig. 108

##### Description.

Small; stubby; subdepressed; yellowish-brown, antennomere 1 yellowish-brown, 2–10 darker, 11 dark basally, paler apically. Head: vertex strongly punctate, medial sulcus absent; frons punctate, not produced; keel absent between antennal bases; depressed between eyes. Antenna: reaches to humerus; slender; antennomeres 1–2 cylindrical, elongate, subequal in length; 3 cylindrical, elongate, 2× length of 2; 4–5 cylindrical, elongate, decreasing in length; 6–10 transverse, subequal in length, each ¾ length of 5; 11 2× length of 10, pointed at apex; 1–2 punctate with scattered setae; 3–11 setose. Pronotum: transverse; lateral margin arcuate from base to anterior angle, finely crenulate, margined; anterior angle obtuse, slightly projecting; posterior angle acute; anterior margin emarginate behind head; disc subconvex; surface with disc irregularly sparsely punctate, densely coarsely punctate laterally; basal impression absent; pronotal length 0.9 mm; pronotal width 1.6 mm. Scutellum: pentagonal; impunctate. Elytron: lateral margin straight, nearly smooth, finely margined; apex rounded, smooth; sutural angle without tooth; humerus rounded, not produced; constricted behind humerus; moderately irregularly punctate-striate, row 10 removed from lateral margin; elytral length 2.9 mm; elytral width 1.7 mm. Venter: pro-, meso-, and metasterna impunctate medially, punctate laterally; abdominal sterna punctate, each puncture with pale seta; suture between sterna 1 and 2 complete; apical margin of last sternite concave in female. Leg: slender; punctate, each puncture with pale seta; tibia with fringe of setae on inner apical margin. Total length: 3.9 mm.

##### Etymology.

From crenulatum (Latin) meaning minutely emarginate for the finely emarginate lateral margin of the pronotum. The name is feminine.

##### Diagnosis.

This species is similar to the pale form of *Cephaloleia steinhauseni*. It can be distinguished by the crenulate lateral margin of the pronotum, by the punctate vertex of the head, and by antennomere 1 and 2 being subequal in length.

##### Distribution.

Ecuador.

##### Type material.

Holotype female: Ecuador: Napo, Yasuni Biological Research Station, 220 m, 0°40'12"S, 76°23'24"W, 18–28 May 1996, P. Hibbs, ECU2H96 005B, ex. malaise trap/ Holotype *Cephaloleia crenulata* Staines, des. C. L. Staines 2012 (red label) (SEMC).

#### Cephaloleia cyanea

Taxon classificationAnimaliaColeopteraChrysomelidae

Staines, 1996

http://species-id.net/wiki/Cephaloleia_cyanea

Fig. 109

Cephaloleia cyanea Staines 1996: 25. McKenna and Farrell 2006: 10949 (phylogeny).

##### Description.

Oval; subconvex; head, pronotum, and scutellum black; elytra dark metallic blue; legs reddish. Head: vertex shallowly, sparsely punctate, without sulcus; frons not projecting; not depressed between eyes. Antenna: reaches to humerus; slender; antennomere 1 subglobose; 2 cylindrical, elongate, longer than 1; 3 elongate, longer than 1–2 combined; 4–10 elongate, decreasing in length, each shorter than 3; 11 pointed at apex, subequal in length to 4; 1–11 setose. Pronotum: transverse; lateral margin straight for basal ½ then rounding to anterior angle, canaliculate; anterior angle produced; posterior angle acute; anterior margin emarginate behind head; disc subconvex; with slight depression in posterior angle; surface impunctate; basal impression absent; pronotal length 0.6–0.9 mm; pronotal width 1.0–1.3 mm. Scutellum: pentagonal, impunctate. Elytron: lateral margin straight, dentate, especially on apical ½, margined; apex rounded; sutural angle without tooth; humerus rounded, slightly produced; slightly constricted behind humerus; punctures shallow; rows converge and unite near apex; punctures are larger and more impressed on elytral disc, rest almost obsolete; elytral length 2.6–3.3 mm; elytral width 1.4–1.7 mm. Venter: pro-, meso-, and metasterna smooth medially, punctate laterally; abdominal sterna punctate, each puncture with pale seta; suture between sterna 1 and 2 obsolete medially, punctate laterally. Leg: slender; punctate; femur and tibia with row of setae on inner margin; tibia with fringe of setae on inner margin of apex. Total length: 3.4–4.1 mm.

##### Diagnosis.

This is a distinctive species which can be distinguished by the metallic blue color, by the smooth lateral margins of the pronotum, and by the serrulate lateral margins of the elytra.

##### Host plant.

Adults have been collected on *Chusquea* sp. (Poaceae) (Staines 1996).

##### Distribution.

Colombia, Costa Rica, Venezuela.

##### Type material examined.

Holotype: Venezuela: Aragua, Rancho Grande, 8 May 1973, 1100 m., Ginter Ekis/ Holotype Cephaloleia cyanea Staines, Des. C. L. Staines 1994 [red label] (USNM).

##### Specimens examined.

**COLOMBIA:** Boyaca- 11 km NW Arcabuco, 13 July 1982 (USNM). **COSTA RICA:** Puntarenas- Monteverde Reserve, 1500 m, 19 August 1987 (CMNC), 1400m 27 February 1980 (CMNC). **VENEZUELA:** Distrito Federal- Caracas, 1921 (USNM). Total: 10.

#### Cephaloleia cylindrica

Taxon classificationAnimaliaColeopteraChrysomelidae

Staines, 1996

http://species-id.net/wiki/Cephaloleia_cylindrica

Fig. 110

Cephaloleia cylindrica Staines 1996: 25.

##### Description.

Elongate; narrow; convex reddish-brown; antennae, eyes, and legs black. Head: vertex smooth, medial sulcus absent; frons not projecting; not depressed between eyes. Antenna: reaches past humerus; slender; antennomeres 1–2 subglobular, short; 3–4 elongate, subequal in length, each 1½x length of 1 and 2 combined; longest; 5–10 elongate, each shorter than 4; 11 subequal to 10, pointed at apex; 1–4 punctate with scattered setae; 5–11 setose. Pronotum: transverse; lateral margin bisinuate, margined; anterior angle pointed, slightly produced; posterior angle acute; anterior margin emarginate behind head; disc subconvex; surface impunctate; basal impression absent; pronotal length 1.1 mm; pronotal width 1.1–1.3 mm. Scutellum: triangular; impunctate. Elytron: lateral margin straight, smooth, margined; apex rounded; sutural angle without tooth; humerus rounded, not produced; slightly constricted behind humerus; shallowly punctate-striate, punctures large; elytral length 3.3–3.6 mm; elytral width 1.7 mm. Venter: pro- and metasterna impunctate; mesosternum impunctate medially, punctate laterally; abdominal sterna punctate, each puncture with pale seta; suture between sterna 1 and 2 complete. Leg: slender; femur punctate at base; tibia with fringe of setae on inner margin of apex. Total length: 4.7–5.3 mm.

##### Diagnosis.

This species is similar to *Cephaloleia puncticollis* and *Cephaloleia sallei*. It can be distinguished by antennomeres 1 and 2 being subglobose.

##### Distribution.

Costa Rica, Panama.

##### Type material examined.

Holotype: X X Plantation, Rep Panama, Feb. 11, 1930 [blue-green label]/ Blackwelder Collection [blue-green label]/ Holotype *Cephaloleia cylindrica* Staines, Des. C. L. Staines 1994 [red label] (USNM).

##### Specimens examined.

**COSTA RICA:** Puntarenas- Est. Sirena, 0–100 m, May 1992 (INBIO). **PANAMA:** 11 February 1930 (USNM). Total: 2.

#### Cephaloleia daguana

Taxon classificationAnimaliaColeopteraChrysomelidae

Uhmann, 1930e

http://species-id.net/wiki/Cephaloleia_daguana

Fig. 111

Cephalolia daguana Uhmann 1930e: 149. Uhmann 1936b: 110 (noted), 1936f: 482 (key), 1938a: 407 (distribution).
Cephaloleia daguana Uhmann. Uhmann 1950a: 274 (sculpture), 1957b: 17 (catalog), 1964a: 403 (catalog); Gaedike and Döbler 1971: 346 (types); Staines 1997b: 413 (Uhmann species list).
Cephalolia palmarum Pic 1923: 9 (type: Ecuador, MNHN, NHRS, not seen).
Cephaloleia palmarum Pic. Uhmann 1957b: 23 (catalog), 1961b: 23 (synonymy), 1964a: 404 (catalog); Descarpentries and Villiers 1959a: 139 (types).
Cephaloleia daguana palmarum Pic. Uhmann 1964a: 404 (transfer).

##### Description.

Slightly elongate; subdepressed; shining; yellowish-brown; head, antennae, and broad medial pronotal vitta black; elytra with black vitta from base along suture extending to beyond middle, reaches to puncture row 7 just below humeri and black apical macula; venter bright yellowish-brown. Head: vertex smooth, with small tubercle and faint medial carina; frons not projecting; not depressed between eyes. Antenna: as long as head and pronotum combined; slender; male with antennomere 1 elongate, 2× as long as wide, with triangular projection; 2 ½ length 1, weakly triangular; 3 and 4 as long as 2, strongly triangular; 11 elongate-oval; female with 1 elongate, 2 conical; 3–4 concial, shorter than 2; 5–10 subequal in length, each shorter than 4; 11 2× length of 10, rounded at apex; 1–3 punctate with scattered setae; 4–11 setose. Pronotum: transverse; lateral margin straight then rounding to anterior angle, narrowly margined; anterior angle broadly rounded, produced; posterior angle acute; anterior margin emarginate behind head; basal margin narrowly margined; disc subconvex, surface distinctly punctate; basal impression absent; pronotal length 2.0–2.2 mm; pronotal width 2.4–2.6 mm. Scutellum: elongate pentagonal; impunctate. Elytron: lateral margin straight, smooth, narrowly margined; apex nearly truncate; sutural angle with small tooth; humerus rounded, not produced; slightly constricted behind humerus; strongly punctate-striate, punctures confused apically; elytral length 7.2–7.4 mm; elytral width 3.2–3.4 mm. Venter: pro-, meso-, and metasterna impunctate; abdominal sterna punctate, each puncture with pale seta; suture between sterna 1 and 2 complete; last sternite with apical margin weakly rounded in male, bisinuate in female. Leg: robust; thick; punctate; femur and tibia with pale seta in each puncture; tibia with fringe of setae on inner margin of apex. Total length: 9.9–10.2 mm.

##### Diagnosis.

This species is similar to *Cephaloleia laeta*. It can be distinguished by the small tubercle on the vertex of the head.

##### Distribution.

Colombia, Ecuador.

##### Type material examined.

Paralectotype: Colombia, Río Dagua, Felsche [printed label]/ Paratypus [red printed label]/ Cephalolia daguana Uh., E. Uhmann det. (DEI).

##### Specimens examined.

**Colombia:** no further data (DEI). **ECUADOR:** Imbabura- Cachabé, November 1896 (USNM); Cachabé to Paramba, February 1897 (USNM). Loja- no further data (USNM). Pichincha- Estación Orongo, Palmitopomba, 23 July 2001 (USNM). Total: 30.

#### Cephaloleia deficiens

Taxon classificationAnimaliaColeopteraChrysomelidae

Uhmann, 1930a

http://species-id.net/wiki/Cephaloleia_deficiens

Fig. 112

Cephaloleia deficiens Uhmann 1930a: 226. Blackwelder 1946: 719 (catalog); Papp 1953: 15 (catalog); Uhmann 1957a: 17 (catalog); Gaedike and Döbler 1971: 346 (types); Wilcox 1983: 136 (catalog); Staines 1996: 26 (Central America species), 1997: 413 (Uhmann species list), 2011: 49 (faunal list); Staines and Staines 1997: 7 (types); McKenna and Farrell 2005: 119 (phylogeny), 2006: 10949 (phylogeny).

##### Description.

Small, ovate-elongate; subconvex; yellowish-brown; antennae, head, and mouthparts black; black vitta present on pronotum and elytra; elytra with black orbicular macula present on suture around scutellum, narrowing and disappearing before middle; venter with pro-, meso-, and metasterna yellow medially, dark laterally, abdomen reddish-brown; leg yellowish, femur dark at apex. Head: vertex densely punctate, golden seta in each puncture, medial sulcus present; frons not projecting; not depressed between eyes. Antenna: ½ body length; slender; antennomere 1 elongate, slightly clavate; 2 elongate, shorter than 1 or 3; 3 elongate, longer than 1, slightly shorter than 11; 4–10 elongate, subequal in length, each shorter than 3; 11 2× length of 10, acutely pointed at apex; 1–5 punctate with scattered setae, 6–11 setose. Pronotum: narrow, transverse, widest at base; lateral margin straight, margined; anterior angle rounded, with small tooth; posterior angle angulate; anterior margin emarginate behind head; disc subconvex; surface coarsely, irregularly punctate; basal impression absent; pronotal length 0.9–1.2 mm; pronotal width 1.0–1.4 mm. Scutellum: impunctate; pentagonal. Elytron: lateral margin straight, smooth, margined; apex rounded; sutural angle without tooth; humerus rounded, not produced; slightly constricted behind humerus; shallowly punctate-striate, punctures larger on disc; humerus nearly impunctate; elytral length 3.1–3.5 mm; elytral width 1.7–1.9 mm. Venter: pro-, meso-, and metasterna impunctate medially, punctate laterally; abdominal sterna punctate, each puncture with pale seta; suture between sterna 1 and 2 obsolete medially. Leg: slender; punctate; femur and tibia with short seta in each puncture; tibia with fringe of setae on inner apex, apex dentate. Total length: 4.3–4.7 mm.

##### Diagnosis.

This species is similar to *Cephaloleia balyi*, *Cephaloleia discoidalis*, *Cephaloleia dorsalis*, *Cephaloleia linkei*, and *Cephaloleia suturalis*. It can be distinguished by the yellowish pronotum with a dark longitudinal vitta and by antennomere 1 being clavate and shorter than 3.

##### Host plant.

According to label data, adults have been collected on *Costus bracteatus* Gleason, *Cephaloleia malortieanus* H. Wendl. (Marantaceae).

##### Distribution.

Costa Rica.

##### Type material examined.

Syntype- Costa Rica, F. Nevermann, 16.VIII.25 [green label]/ Hamburg Farm, Reventazon, Ebene Limon [reversed green label]/ Holotype [red label]/ *Cephalolia deficiens* sp. n./ Cotype No. 54635 USNM [orange label] (USNM, 1).

##### Specimens examined.

**COSTA RICA:** Alajuela- Upala, Sector San Ramón, 1.5 km NW Hacienda Nueva Zelandia (INBIO). Cartago- Quebrada Segunda, P. N. Tapantí, 1200–1300 m (INBIO); Turrialba (USNM). Guanacaste- Estación Pitilla, 9 km S Santa Cecilia, 600–700 m (INBIO); La Cruz, 9 km S Santa Cecilia, 600–700 m (INBIO). Heredia- La Selva nr. Pto. Viejo, 50 m, 19 February 1980 (CMNC); Rara Avis Biological Station, 5 July 2011, 6 July 2011 (USNM). Limón- Hamburg Farm, Reventazón, Ebene Limón, 16 August 1925 (DEI); Sardinas, Barra del Colorado, 4 km NW Cerro Cocorí, 0–100 m (INBIO); A. C. Llanuras del Tortuguero, 0–100 m (INBIO); Pococí, Colorado, Sector Cerro Cocorí, 30 km N Cariari, 100–200 m (INBIO); Sito Tibieblas, 2 km NE Tigra, 1400–1500 m (INBIO). Total: 13.

#### Cephaloleia degandei

Taxon classificationAnimaliaColeopteraChrysomelidae

Baly, 1858

http://species-id.net/wiki/Cephaloleia_degandei

Fig. 113

Cephalolia degandei Baly 1858: 57. Gemminger and Harold 1876: 3601 (catalog); Donckier 1899: 548 (catalog); Weise 1910: 89 (noted), 1911a: 7 (catalog), 1911b: 11 (catalog); Uhmann 1936b: 115 (noted), 1942b: 98 (noted), 1953d: 47 (faunal list).
Cephaloleia degandei Baly. Waterhouse 1881: 261 (distribution); Uhmann 1957b: 17 (catalog); Balazuc 1988: 397 (pathogens); Staines and Staines 1999: 523 (Baly species list).

##### Description.

Narrowly elongate; subparallel; subdepressed; dorsum black, venter pale yellow; vertex of head black, frons yellowish; antennomeres 1–3 yellowish, 4–6 black, 7–11 whitish; lateral margins of pronotum, scutellum, and base of elytra with broad yellowish band, middle and apex of elytra with broad yellowish band; legs pale yellow. Head: vertex finely punctate, medial sulcus present; frons concave, not projecting; not depressed between eyes. Antenna: reaches to humerus; slender; antennomere 1 incrassate, 2× length 2; 2 slightly elongate; 3 slightly longer than 2; 4–6 elongate, subequal in length, each ¾ length of 3; 7–10 subequal in length, each shorter than 6; 11 2× length 10, rounded at apex; 1–6 punctate with scattered setae; 7–11 setose. Pronotum: quadrate; lateral margin slightly sinuate then rounding to anterior angle, canaliculate; anterior angle rounded; posterior angle acute; anterior margin emarginate behind head; disc subconvex; surface deeply, irregularly punctate, punctures more dense laterally, punctures moderate to large; basal impression absent; pronotal length 1.7–1.9 mm; pronotal width 2.2–2.4 mm. Scutellum: elongate pentagonal; impunctate. Elytron: lateral margin straight, smooth, narrowly margined; apex slightly rounded, sutural angle emarginate, without tooth; humerus rounded, not produced; slightly constricted behind humerus; subconvex, disc flattened; shallowly punctate-striate, punctures in irregular rows; elytral length 6.4–6.8 mm; elytral width 3.0–3.2 mm. Venter: pro-, meso-, and metasterna impunctate; abdominal sterna punctate, each puncture with pale seta; suture between sterna 1 and 2 complete; last sternite with apical margin concave-emarginate in male, broadly emarginate in female. Leg: slender; impunctate; femur robust; tibia with fringe of setae on inner margin of apex. Total length: 8.8–9.2 mm.

##### Diagnosis.

This species is similar to *Cephaloleia amba* sp. n. It can be distinguished by the smooth lateral margins of the pronotum and by antennomere 1 being twice the length of 2.

##### Distribution.

Ecuador, Peru.

##### Type material examined.

Syntype: Peru [handwritten label]/ Baly coll. [printed label]/ Cephalolia degandei Baly, Peru [blue handwritten label] (BMNH, 1).

##### Specimens examined.

No label data (USNM). **Ecuador:** 1880 (USNM). Napo- Puyo, 960 m, 1–8 October 1970 (USNM); Limonocha, 300 m, 31 March 1974 (EGRC); Río Napo, Sacha Lodge, 3–13 June 1994 (BYUC); Shushufindi, 215 m, 11 August 1997 (CDFA, USNM). **Peru:** Loreto- Yurimaguas, June-August 1885 (USNM). Total: 22.

#### Cephaloleia delectabilis

Taxon classificationAnimaliaColeopteraChrysomelidae

Staines, 1996

http://species-id.net/wiki/Cephaloleia_delectabilis

Fig. 114

Cephaloleia delectabilis Staines 1996: 26.

##### Description.

Elongate; subparallel; moderately convex; reddish-brown; antennal antennomeres 3–11 black; eyes dark. Head: vertex finely punctate, with two curved sulci, separated by wide carina; frons not projecting; not depressed between eyes. Antenna: as long as head and pronotum combined; robust; antennomeres 1–2 elongate, subequal in length; 3–10 transverse, subequal in length, each shorter than 2; 11 rounded at apex, subequal in length to 1 or 2; 1–5 punctate with scattered setae; 6–11 setose. Pronotum: transverse; lateral margin straight, slightly convergent from base to apex, margined; anterior angle rounded, not produced; posterior angle acute; anterior margin curved posteriorly; disc subconvex; surface sparsely, irregularly punctate; basal impression absent; pronotal length 0.9 mm; pronotal width 1.0 mm. Scutellum: pentagonal; impunctate. Elytron: lateral margin straight, smooth, margined; apex rounded; small tooth present in sutural angle; humerus rounded, not produced; slightly constricted behind humerus; moderately punctate-striate, punctures shallow, large; puncture rows converge and unite at apex; elytral length 2.9 mm; elytral width 1.4 mm. Venter: pro- and mesosterna impunctate medially, rugose laterally; metasternum impunctate medially, punctate laterally; abdominal sterna sparsely punctate, each puncture with pale seta; suture between sterna 1 and 2 obsolete medially. Leg: slender; femur and tibia punctate; tibia with fringe of setae on inner margin of apex. Total length: 3.7 mm.

##### Diagnosis.

This species is similar to *Cephaloleia nigricornis*. It can be distinguished by the elytral puncture rows being distinct apically and by antennomere 1 being subequal in length to 2.

##### Distribution.

Mexico.

##### Type material examined.

Holotype: Mexico, Chiapas, Pq. Laguna Belgica, 16kmNW Ocozocoautla, 14.VI.1989, H. Howden/ flight intercept trap/ Holotype Cephaloleia delectabilis Staines, Des. C. L. Staines 1994 [red label] (CMNC).

#### Cephaloleia deplanata

Taxon classificationAnimaliaColeopteraChrysomelidae

Uhmann, 1927

http://species-id.net/wiki/Cephaloleia_deplanata

Fig. 115

Cephalolia deplanata Uhmann 1927: 51. Uhmann 1930d: 150 (noted).
Cephaloleia deplanata Uhmann. Uhmann 1948a: 220 (noted), 1957b: 17 (catalog); Gaedike and Döbler 1971: 347 (types); Staines 1997b: 413 (Uhmann species list).

##### Description.

Elongate; subdepressed; shining; pitchy-black except lateral margin of pronotum and elytra; elytra with metallic sheen; antennae and venter brownish. Head: vertex punctate, medial carina present; frons not projecting; depressed between eyes. Antenna: as long as head and pronotum combined; slender; antennomere 1 elongate; 2 shorter than 1; 3 subequal in length to 1; 4–10 elongate, nearly as long as 3; 11 2× length of 10, pointed at apex; 1–2 punctate with scattered setae; 3–11 setose. Pronotum: transverse; lateral margin arcuate, narrowly margined; anterior angle rounded, produced; posterior angle acute; anterior margin emarginate behind head; disc subdepressed; surface with large rounded punctures present laterally, disc nearly impunctate; transverse basal impression present medially; pronotal length 0.9–1.2 mm; pronotal width 1.3–1.7 mm. Scutellum: pentagonal; impunctate. Elytron: lateral margin weakly rounded, smooth, narrowly margined; apex obliquely rounded; sutural angle without tooth; humerus rounded, not produced; constricted behind humerus; weakly punctate-striate, punctures weaker apically; pygidium visible, triangular in female, angulate in male; elytral length 3.1–3.5 mm; elytral width 1.6–1.9 mm. Venter: pro-, meso-, and metasterna sparsely punctate; abdominal sterna sparsely punctate, each puncture with pale seta; suture between sterna 1 and 2 obsolete medially; last sternite with apical margin strongly sinuate in male, emarginate medially in female. Leg: slender; punctate; femur and tibia with pale seta in each puncture; tibia with fringe of setae on inner margin of apex. Total length: 4.0–5.0 mm.

##### Diagnosis.

This species is similar to *Cephaloleia coroicoana*, *Cephaloleia fiebrigi*, *Cephaloleia marantae*, and *Cephaloleia rufipes*. It can be distinguished by the basal impression on the pronotum, by the elytral punctures being distinct basally and apically, and by the puncture rows not converging and uniting apically.

##### Host plant.

According to label data, adults have been collected feeding on *Elaeis guineensis* Jacq. (Arecaceae).

##### Distribution.

Brazil (Bahia, Rondonia), Suriname, Venezuela.

##### Type material examined.

Holotype male: Suriname, ♂, Haag/ Holotypus [red label]/ Cephalolia deplanata Uh., Det. E. Uhmann (DEI).

##### Specimens examined.

**Brazil:** Bahia- no further data (USNM); Opalma, 27 June 1967 (USNM); Sta. Amaro, 16 December 1996 (USNM). Rondonia- 62 km SW Ariquames, Fzda Rancho Grande, 15 November 1994 (BYUC). **Venezuela:** Aragua- Rancho Grande Biological Station, 1100 m, 4 June 1996 (SEMC). Total: 30.

#### Cephaloleia depressa

Taxon classificationAnimaliaColeopteraChrysomelidae

Baly, 1858

http://species-id.net/wiki/Cephaloleia_depressa

Fig. 116

Cephalolia depressa Baly 1858: 60. Gemminger and Harold 1876: 3601 (catalog); Donckier 1899: 548 (catalog); Weise 1911a: 7 (catalog), 1911b: 11 (catalog).
Cephaloleia depressa Baly. Maulik 1924: 246 (noted); Uhmann 1948a: 220 (noted), 1957b: 17 (catalog); Mariau 1999: 233 (noted), 2001: 132 (noted); Staines and Staines 1999: 523 (Baly species list).

##### Description.

Small; elongate; flattened; black, lateral margins of pronotum paler; venter black; tarsi piceous. Head: vertex punctate, with medial carina; frons not projecting; depressed between eyes. Antenna: ½ body length; slender; antennomere 1 slightly incrassate, elongate, longer than 2, subequal in length to 3; 2 elongate, cylindrical; 3 elongate, slender; 4–10 elongate, slightly decreasing in length, each shorter than 3; 11 2× length of 10, pointed at apex; 1–2 punctate; 3–11 setose. Pronotum: quadrate; lateral margin arcuate, canaliculate, serrulate; anterior angle pointed; posterior angle acute; anterior margin emarginate behind head; disc slightly convex; surface densely coarsely punctate; basal impression absent; pronotal length 0.6–0.9 mm; pronotal width 1.1–1.5 mm. Scutellum: broadly triangular; alutaceous. Elytron: lateral and apical margins smooth; lateral margin straight, margined; apex rounded; sutural angle without tooth; humerus rounded, not produced; slightly constricted behind humerus; punctures moderately impressed; pygidium coarsely punctate, obtusely rounded at apex; elytral length 2.4–2.8 mm; elytral width 1.1–1.5 mm. Venter: pro-, meso-, and metasterna sparsely punctate; abdominal sterna sparsely punctate, each puncture with pale seta; suture between sterna 1 and 2 obsolete medially; last sternite with apical margin deeply sinuate in male, emarginate medially in female. Leg: slender; punctate; femur and tibia with pale seta in each puncture; tibia with fringe of setae on inner margin of apex. Total length: 3.5–3.9 mm.

**Figures 116–124.**
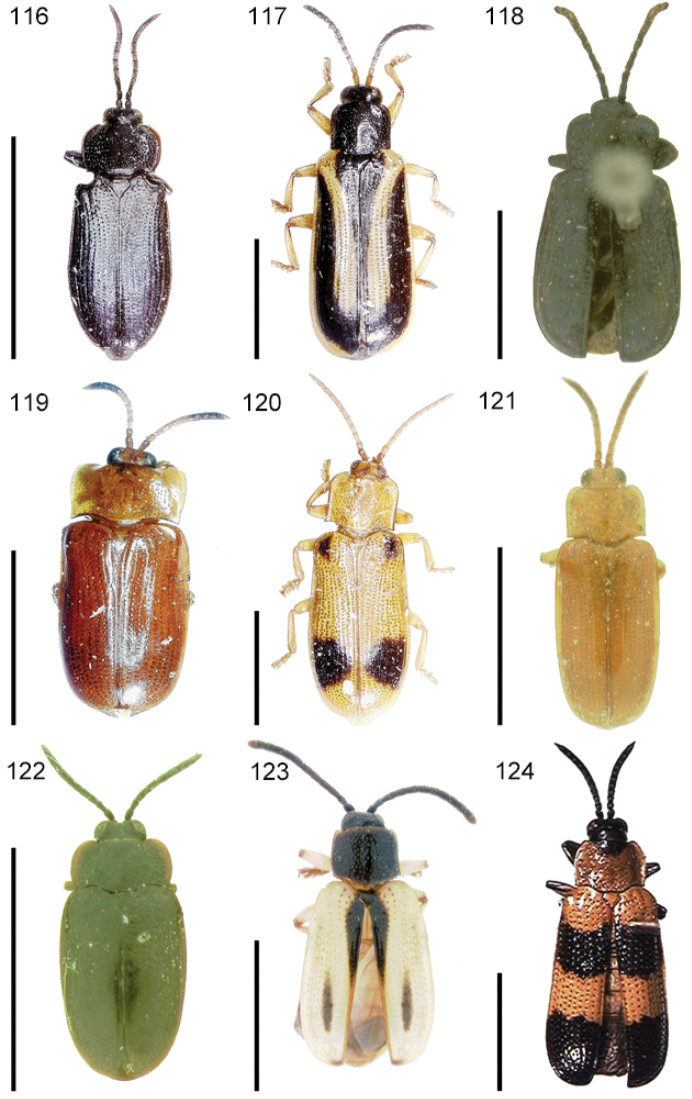
Habitus. **116** *Cephaloleia depressa* **117** *Cephaloleia deyrollei* **118** *Cephaloleia dilatata* **119** *Cephaloleia dilaticollis* **120** *Cephaloleia dilectans* **121** *Cephaloleia dimidiaticornis* **122** *Cephaloleia diplothemium* **123** *Cephaloleia discoidalis* **124** *Cephaloleia disjuncta*. Scale bars equal 3 mm.

##### Diagnosis.

This species is similar to *Cephaloleia donckieri*, *Cephaloleia elaeidis*, *Cephaloleia tarsata*, and *Cephaloleia zikani*. It can be distinguished by the vertex of the head without a medial fovea, by the serrulate lateral margins of the pronotum, by the smooth apical margins of the elytra, and by antennomere 2 being obconic.

##### Host plant.

*Elaeis guineensis* Jacq. (Arecaceae) (Mariau 2001).

##### Distribution.

Brazil (Bahia, Matto Grosso, Pará), Ecuador.

##### Type material examined.

Holotype male: Santarem, Upper Amazons [handwritten label]/ Bates [handwritten label]/ Cephalolia depressa Baly, Santarem [blue handwritten label] (BMNH).

##### Specimens examined.

No label data (USNM). **Brazil:** ?- Taperina (USNM). Bahia- Opalma, 17 June 1967 (USNM). Matto Grosso- 1886 (USNM). **Ecuador:** Orellana- 1 km S Onkone Gare Camp, Reserva Etnica Waorani, 216.3 m, 23 June 1996, 2 October 1996, 4 October 1995 (USNM). Total: 10.

#### Cephaloleia deyrollei

Taxon classificationAnimaliaColeopteraChrysomelidae

Baly, 1858

http://species-id.net/wiki/Cephaloleia_deyrollei

Fig. 117

Cephalolia deyrollei Baly 1858: 53. Gemminger and Harold 1876: 3601 (catalog); Donckier 1899: 548 (catalog); Weise 1910: 89 (noted), 1911a: 7 (catalog), 1911b: 12 (catalog).
Cephaloleia deyrollei Baly. Lüderwaldt 1910: 61 (host plant); Uhmann 1957b: 17 (catalog), 1964b: 4 (faunal list); Buck 1958: 146 (museum list); Staines and Staines 1999: 523 (Baly species list); Staines 2004a: 312 (host plant).

##### Description.

Elongate; moderately convex; black, eyes dark, antennomeres all black or 1–4 yellowish, 5–11 dark, elytra with yellowish vitta from humeri to just beyond middle, lateral margin yellowish; venter black, abdominal sterna yellowish laterally; legs yellowish. Head: vertex punctate, medial sulcus absent; small tubercle present on front; frons not projecting; depressed between eyes. Antenna: ½ body length; slender; antennomere 1 elongate, 2× length of 2; 2 transverse, shortest; 3 cylindrical, subequal in length to 1; 4–5 subequal in length, each shorter than 3; 6–10 transverse, subequal in length, each shorter than 5; 11 2× length 10, rounded at apex; 1–2 punctate with scattered setae; 3–11 setose. Pronotum: subquadrate; lateral margin straight then rounding to anterior angle, canaliculate; anterior angle rounded, slightly produced; posterior angle acute; anterior margin straight; disc subconvex; surface deeply, sparsely punctate; basal impression absent; pronotal length 1.2–1.4 mm; pronotal width 1.3–1.5 mm. Scutellum: broadly pentagonal; impunctate. Elytron: lateral margin straight, smooth, slightly margined; apex rounded; sutural angle without tooth; humerus rounded, not produced; slightly constricted behind humerus; distinctly punctate-striate; interspaces slightly raised; elytral length 4.2–5.3 mm; elytral width 1.8–2.2 mm. Venter: pro-, meso-, and metasterna punctate; abdominal sterna punctate, each puncture with pale seta; suture between sterna 1 and 2 complete; last sternite with apical margin broadly, weakly emarginate medially in male, rounded, entire in female. Leg: slender; impunctate; tibia with fringe of setae on inner margin of apex Total length: 5.8–7.0 mm.

##### Diagnosis.

This species is similar to *Cephaloleia flavovittata*. It can be distinguished by antennomere 1 being elongate.

##### Host plant.

*Marantha* sp. (Marantaceae) (Lüderwaldt 1910).

##### Distribution.

Bolivia, Brazil (Bahia, Corcovado, Paraná, Río de Janiero, Santa Catharina, São Paulo), Ecuador, French Guiana.

##### Type material examined.

Holotype male: St. Catharina [handwritten label]/ Baly coll. [printed label]/ Cephalolia deyrollei Baly, St. Catharina [blue handwritten label] (BMNH).

##### Specimens examined.

**BOLIVIA:** Cochabamba- Para Chapare, Villa Tuneri, 450 m, November 1952 (USNM). **Brazil:** Bahia- no further data (USNM). Río de Janiero- Corcovado, Río Guanabara, 19 September 1961 (USNM); Nuri, 1000 m, 26 February 1952 (USNM). Santa Catharina- Río Vermeine, June 1946 (USNM). Goiás- Therezopolis, October 1944 (USNM). São Paulo- Casa Grande, September 1938 (USNM). **ECUADOR:** Bolivar- Balzapamba, March-April 1894 (USNM). **French Guiana:** Region 8, Iwokrama Forest, Kabocalli Field Station, 4 June 2001, 5 June 2001 (SEMC). Total: 21.

#### Cephaloleia dilatata

Taxon classificationAnimaliaColeopteraChrysomelidae

Uhmann, 1948a

http://species-id.net/wiki/Cephaloleia_dilatata

Fig. 118

Cephaloleia dilatata Uhmann 1948a: 220. Uhmann 1957b: 17 (catalog), 1961b: 6 (noted), 1964a: 403 (catalog); Gaedike and Döbler 1971: 347 (types); Staines 1997b: 413 (Uhmann species list).

##### Description.

Elongate-oval; flattened; somewhat shining; head, antenna, pronotum, and legs black; elytra metallic blue; venter with prosternum brown. Head: vertex sparsely punctate, with medial sulcus; frons with small tubercle, not projecting; slightly depressed between eyes. Antenna: as long as head and pronotum combined; slender; antennomeres 1–3 elongate; 1 longest; 2–3 subequal in length; 4–10 transverse, subequal in length, each shorter than 3; 11 2× length of 10, rounded at apex; 1–3 punctate with scattered setae; 4–11 setose. Pronotum: transverse; lateral margin straight and divergent for basal ¾ then rounding to anterior angle, canaliculate; anterior angle angulate, not produced; posterior angle acute; anterior margin emarginate behind head; disc transversely convex, virtually impunctate; surface moderately punctate laterally; basal impression absent; pronotal length 1.0–1.2 mm; pronotal width 1.6–1.8 mm. Scutellum: pentagonal; impunctate. Elytron: lateral margin straight, smooth, margined; apex rounded, smooth; sutural angle without tooth; humerus rounded, not produced; slightly constricted behind humerus; subconvex; shallowly punctate-striate; elytral length 4.0–4.2 mm; elytral width 2.2–2.4 mm. Venter: prosternum punctate medially, alutaceous laterally; meso- and metasterna punctate; abdominal sterna punctate, each puncture with pale seta; suture between sterna 1 and 2 complete; last sternite with apical margin bisinuate. Leg: robust; punctate; femur and tibia with pale seta in each puncture; tibia with fringe of setae on inner margin of apex. Total length: 5.2–5.6 mm.

##### Diagnosis.

This species is similar to *Cephaloleia caeruleata* and *Cephaloleia diplothemium*. It can be distinguished by the cylindrical antennomere 1 which is longer than 3, by the elytral punctures being the same size laterally, and by the prosternum being alutaceous laterally.

##### Distribution.

Brazil (Minas Gerais).

##### Type material examined.

Holotype: Brazil, Virginia, 1500 m, S. Minas Gerais, Faz. Campos, V.1919, Zikan [green printed label]/ Holotyp [red printed label]/ Cephaloleia dilata Uh., Det. E. Uhmann (DEI).

#### Cephaloleia dilaticollis

Taxon classificationAnimaliaColeopteraChrysomelidae

Baly, 1858

http://species-id.net/wiki/Cephaloleia_dilaticollis

Fig. 119

Cephalolia dilaticollis Baly 1858: 46. Gemminger and Harold 1876: 3601 (catalog); Donckier 1899: 548 (catalog); Weise 1911a: 7 (catalog), 1911b: 11 (catalog).
Cephaloleia dilaticollis Baly. Baly 1885: 13 (distribution); Uhmann 1936a: 112 (comparative note); Blackwelder 1946: 719 (catalog); Papp 1953: 15 (catalog); Uhmann 1957a: 17 (catalog); Wilcox 1983: 136 (catalog); Staines 1996: 27 (Central America species), 1996(1997): 14 (Nicaragua species), 2004: 312 (host plants), 2011: 49 (faunal list); Maes 1999: 1016 (faunal list); Staines and Staines 1999: 523 (Baly species list); McKenna and Farrell 2005: 119 (phylogeny), 2006: 10949 (phylogeny); Meskins et al. 2008: 163 (host plants), 2011: 483 (food web); Descampe et al. 2008: 227 (host plants); García–Robledo et al. 2010: 51 (larva, biology), 2013a: 3 (biology), 2013b: 193 (biology); García–Robledo and Horvitz 2011: 978 (biology), 2012: 40 (biology).
Cephalolia laticollis Baly 1869a: 368 (type: Upper Amazons; Peru, BMNH, not seen). Gemminger and Harold 1876: 3601 (catalog); Donckier 1899: 549 (catalog); Weise 1910: 87 (synonymy), 1911a: 7 (catalog), 1911b: 11 (catalog).
Cephaloleia laticollis Baly. Waterhouse 1881: 262 (distribution); Staines and Staines 1999: 524 (Baly species list).
Cephalolia dilaticollis laticollis Baly. Uhmann 1930a: 224 (faunal list).
Cephalolia abscisa Uhmann 1936a: 112 (Lectotype: Costa Rica, Hamburg Farm, F. Nevermann [green printed lable]/ Ebene Limon, 15.II.1924/24.V.1931 [reversed green label]/ Holotyp [red printed label]/ Cephalolia abscisa Uh., DEI). Uhmann 1942: 94 (noted), 1953: 47 (faunal list).
Cephaloleia abscisa Uhmann. Blackwelder 1946: 718 (catalog); Papp 1953: 13 (catalog); Uhmann 1957a: 14 (catalog); Gaedike and Döbler 1971: 341 (types): Staines 1996: 27 (synonymy), 1997: 413 (Uhmann species list).

##### Description.

Subquadrate; flattened; reddish brown, eyes and apical four antennomeres darker. Head: vertex with faint medial carina, sparsely punctate; frons not projecting; not depressed between eyes. Antenna: reaches to humerus; robust; antennomere 1 obovate, subequal to 3; 2 transverse; 3 elongate; 4–10 transverse, subequal in length; 11 2× length of 10, pointed at apex; 1–2 punctate with scattered setae; 3–11 setose. Pronotum: twice as wide as long; lateral margin dilated, slightly rounded; anterior angle angulate; posterior angle angulate; anterior margin emarginate behind head; disc flat; surface sparsely punctate; basal impression absent; pronotal length 0.7–1.0 mm; pronotal width 1.9 mm. Scutellum: triangular; impunctate. Elytron: lateral margin straight, smooth, margined; apex obtusely rounded; sutural angle without tooth; humerus rounded, not produced; slightly constricted behind humerus; slightly convex, flattened along suture; finely punctate-striate; puncture rows converge and unite at apex; last segment of pygidium u-shaped in male, truncate in female; elytral length 3.0–3.6 mm; elytral width 2.1 mm. Venter: prosternum rugose medially, impunctate laterally; meso- and metasterna impunctate medially, punctate laterally; abdominal sterna sparsely punctate, each puncture with pale seta; suture between sterna 1 and 2 obsolete medially; last sternite with apical margin u-shaped in male, truncate in female. Leg: slender; punctate; tibia darker at base, fringe of setae on inner apical margin. Total length: 4.3–4.6 mm; females are larger than males.

##### Diagnosis.

This species is similar to *Cephaloleia aequilata* and *Cephaloleia cognata*. It can be distinguished from *Cephaloleia aequilata* by elytral puncture rows 6 to 9 distinct on the humerus and from *Cephaloleia cognata* by lacking a costate interspace behind the humerus on the elytra.

##### Comments.

Preliminary analysis of the CO1 gene indicates that cryptic species may be present under the current application of this species name. Further work is needed to resolve this question.

##### Host plant.

Adults have been collected on Musaceae and *Calathea insignis* Hort. and Bull. (Staines 1996); *Cephaloleia lutea* Schult. (Marantaceae), *Renealmia* sp. (Zingiberaceae) (McKenna and Farrell 2005), *Renealmia alpinia* (Rottb.) Maas (García–Robledo et al. 2010); *Cephaloleia inocephala* (Kuntze) H. Kennedy, *Ischnosiphon pruinosus* Peterson (Meskins et al. 2008); *Cephaloleia insignis* Hort. and Bull. (Descampe et al. 2008) (Marantaceae); *Cephaloleia crotalifera* S. Watson, *Cephaloleia lasiostachya* Donn. Sm., *Cephaloleia marantifolia* Standl., *Cephaloleia similis* H. Kenn., *Heliconia imbricata* Baker, *Heliconia latispatha* Benth., *Heliconia pogonantha* Cufod., (Heliconiaceae) *Renealmia cernua* J. F. Macbr. (Zingiberaceae), (García–Robledo et al. 2013a); *Heliconia latispatha* Benth., *Heliconia rostrata* Ruiz & Pav. (Heliconiceae), *Musa paradisiaca* L (Musaceae) (Schmitt and Frank 2013); *Cephaloleia guzmanioides* L. B. Sm. and Idrobo, *Alpinia purpurata* K. Schum., *Hedychium coronarium* J. Koenig, *Musa velutina* H. Wendl. and Drude (Musaceae).

##### Immatures.

Color when live (Figs 31–34) pale yellowish-brown, margins translucent; which dark markings as follows: posterior margin and small medial macula on pronotum; mesonotum with medial longitudinal vitta and basal margin; metanotum and abdominal tergite 1 with medial longitudinal vitta and anterior and posterior margins; abdominal tergites 2–4 with medial longitudinal vitta; tergites 6–7 similar to metanotum; tergites 8–10 with medial longitudinal vitta. Venter pale yellowish. Color when dead pale yellowish with dark markings. With medial longitudinal ridge from anterior to posterior margin. Pronotum with central raised area, surface micropustulate; with two diagonal carinae from central raised area to anterior margin; anterior and lateral areas punctate. Mesonotum with anterior margin carinate from side to side; laterally with sharply curved carina which extends to anterior margin; punctate laterally. Metanotum with diagonal carina which extends to lateral margin; punctate laterally. Abdominal tergites 1–6 wider than long, decreasing in width; punctate laterally. Tergites 7–9 with two diagonal carinae on each side which extend to lateral margins. Spiracles just off central elevation, with margins darkened. Venter: surface of expansions rugose-punctate. Head surface punctate; labrum with surface alutaceous, without setae; clypeus with fringe of long setae at apex, with four setae on apical ½, surface alutaceous; mandibles tridentate; maxillary palps with 2 palpomeres and 12 short, robust setae at apex; maxilla robust, clavate, with fringe of long setae at apex; labium densely setose. Antenna with antennomere 1 longer than 2, subelongate; 2 wider than 1, transverse; 3 obconical, slightly longer than 2, with short setae at apex. Prosternum nearly as wide as long. Meso- and metasterna wider than long. Abdominal sternites 1–7 wider than long; decreasing in width; with three sulci on apical ½; laterally with curved sulcus dividing the sternite into thirds; sternite 8 similar to preceding but without any sulci; sterna 9–10 fused, rounded at apex. Leg robust; femur rugose-striate, with scattered setae; tibiotarsus obconical, with scattered long setae and strong claw at apex. Total length: 5.7–6.0 mm; width 3.6 mm. (García–Robledo et al. 2010).

##### Biology.

From García–Robledo et al. (2010): Eggs are about 2 mm long and are laid singly or in clusters or two or more on host plant petioles or rolled leaves and are covered with frass. Eggs hatch in 5 to 9 days. The larvae have two instars, the first lasting 8 to 14 days and the second 24 to 40 days. The pupal stage lasts 13 to 19 days. Adults live about 169 days.

##### Distribution.

Bolivia, Brazil, Colombia, Costa Rica, Ecuador, Mexico, Nicaragua, Panama, Peru, Venezuela.

##### Type material examined.

Syntype: Bogata [handwritten label]/ Cephalolia dilaticollis Baly, Bogata [blue handwritten label] (BMNH, 1).

##### Specimens examined.

**Bolivia:** Cochabamba- 67.5 km NE Cochabamba, Est Biol Valle del Sajita, 9–13 February 1999 (SEMC). **COSTA RICA:** Alajuela- Estación Elasios, 700–800 m (INBIO); Peñas Blancas, 850 m, 17 May 1989, 19 May 1989 (SEMC). Cartago- 19.3 km NE San José, 1010 m, 17 May 1993 (SEMC); Turrialba, 4–13 August 1970 (USNM), 28 February 1980 (CMNC). Guanacaste- Finca Loaiciga, 500 m, 6 km S Sta Cecilia, P. N. Guanacaste, 23 September–14 October 1992 (INBIO); Est. Pitilla, 700 m, 9 km S Sta. Cecilia, P.N. Guanacaste, February 1990, 12 May 1991, 22 October–8 November 1992 (INBIO); Río San Lorenzo, 1050 m, Tierras Morenas, Z. P. Tenorio, April 1991, 28 March–21 April 1992, August 1992 (INBIO). Heredia- Chilamate, 18–23 August 1988 (BYUC); Fca. La Selva nr. Puerto Viejo, 27 June 1969, 2 August 1969 (USNM), 31 March 1990 (MUCR), 03 July 2001, 7 April 2003, 12 March 2005 (USNM); Colateola area, La Selva, 5 August 1969 (USNM); Sarapiquí, Finca La Selva, 0–100 m (INBIO); La Selva Res. Stn., 16 July 1973 (EMEC), 1 September 1998 (BYUC); Los Arbolitos, La Virgen, 0–100 m (INBIO); Rara Avis Biological Station, 10 November 2011 (USNM). Limón- Cerro Tortuguero, 0–120 m, P. N. Tortuguero, February 1993 (INBIO); 7 mi N Guacimo, 22 February–3 March 1988 (BYUC); Guápiles, 17 February 1924 (USNM); Hamburg Farm, Reventazón, Ebene Limón, 3 March 1928, 22 May 1931, 24 May 1931 (USNM), 24 May 1931 (DEI), February 1925, February 1932 (MUCR); Est. Hitoy-Cerere, 100 m, R. Cerere, Res. Biol. Hitoy Cerere, January 1991, June 1991, 12 April 1992, 30 June–20 July 1992 (INBIO); Sector Cerro Cocorí, Fca. de E. Rojas, 150 m, November 1991, March 1992, 12–31 August 1992 (INBIO); Waldeck, 17 February 1924 (USNM); Valle La Estrella, 100–200 m (INBIO); Pococí, Colorado, Estación Cuatro Esquinas (INBIO); A. C. Llanuras del Tortuguero, 0–100 m (INBIO); Pococí, Sector Cerro Cocorí, 30 km N Cariari, 100–200 m (INBIO); Amubri, Sendero Soki, 0–100 m (INBIO); Talamanca, Amubri, 0–100 m (INBIO); Gandoca Manzanillo, 0–100 m (INBIO). Puntarenas- Las Alturas, 1400 m, 22 May 1992 (CDFA); Coto Brus, Las Cruces Biological Station, 5 March 2012, 10 March 2012 (USNM); Corcovado National Park, Sierna Stn., Corcovado Trail, 150 m, 29 June 2000 (SEMC); 1.5 mi. S. Palmar Sur, 11 August 1969 (USNM); Rancho Quemado, Peninsula de Osa, 200 m, February 1991, April 1991, October 1991, 21 March–7 April 1992 (INBIO); 5 km S. Rincón, 20 March 1973 (SEMC); 3.5 mi. S. Rincón, Osa Peninsula, 28 February-12 March 1969 (CASC); Est. Sirena, Corcovado N.P., 0–100 m, January 1990, March 1990, April 1990, September 1991, November 1991, December 1991, January 1992, 12–31 August 1992 (INBIO); Sirena Station, Corcovado National Park, Corcovado Trail, 29 June 2000 (SEMC); Sirena Station, Corcovado National Park, lower Ollas Trail, 24–28 June 2000 (SEMC); Estación Altamira, 1 km S Cerro Biolley, 1400–1500 m (INBIO); F. Las Cruces, Laguna Gamboa, 1400–1500 m (INBIO); Estación Esquinas, Peninsula Osa, 0–100 m (INBIO); Est Río Bonito, 1.4 km O Cerro Gamba, 200–300 m (INBIO); Fila Madre, 3 km SW Cerro Rincón, 500–600 m (INBio); Sierpe, 0.2 km NW Estación Esquinas, 0–100 m (INBIO); Guacimal, Finca Buen Amigo, 1000–1100 m (INBIO); A. C. A., Bosque Eterno de los Niños, 1500–1600 m (INBIO); Est La Casona, Las Torres, 1500–1600 m (INBIO). San José- Finca La Caja, 14 June 1931 (MUCR); Teleferico, P.N. Braulio Carrillo, 13 July 1999 (USNM). **Ecuador:** Esmeraldas- Canton San Lorenzo Chuchubi, 2 December 2008 (BYUC); San Mateo, 6 September 1958 (USNM); 31.7 km NW Lita, 620 m, 24 August 1997 (USNM). Imbabura- Cachabé, no date, November 1896 (USNM). Los Ríos- Río Palenque, 47 km S Sto. Domingo, 220 m, 26 August 1997 (USNM). Napo- Limoncoha, 8 June 1977 (USNM); San Rafael Falls, 20 km SW El Reventador, 7 August 1997 (USNM); San Rafael Falls, 1100 m, 5–6 August 1998 (USNM); Sacha Lodge, 14–24 March 1994, 14–24 May 1994, 16–29 August 1994, 23 March 1999 (SEMC); Shushufindi, 215 m, 12 August 1997 (USNM); Orellana- Tiputini Biodiversity Station, nr. Yasuni National Park, 220–250 m, 22 October 1998 (USNM); Yasuni, 10–13 August 1998 (USNM); Estación Cientifica Yasuni, 215 m, 5–10 September 1999 (EGRC). Pastaza- Puyo, 960 m, 1–8 October 1970 (USNM). **MEXICO:** no further data (USNM); Perez Zeledón, Santa Elena, Las Nubes, 1200–1300 m (INBIO); Finca El Gringo, Estación Las Nubes de Santa Elena, 1200–1300 m (INBIO); Finca El Gringo, Est. Las Nubes de Santa Elena, 1500–1600 m (INBIO). **Nicaragua:** Río San Juan- 60 km SE San Carlos, Refugio Bartoia, 27 May 2002 (SEMC). **PANAMA:** Bocas del Toro- 6 km N Punta Peña, 27 May 1993 (CDFA). Chiriquí- Bugaba (AMNH, USNM); + 10 mi. N. Concepción, 3 June 1977 (CMNC); Reserva Fortuna, Continental Divide Trail, 25 May 1993 (CDFA), 26 May 1993 (AJGC); Reserva La Fortuna, 26 May 1993 (EGRC); Reserva La Fortuna, Hydrographic sta. trail, 26 May 1993. Coclé- Cerro Gaital, 4000', 1 June 1993 (AJGC). Colón- Pipeline Rd. km 2, 12–17 June 1993, 21 June 1993 (SEMC); Porto Bello, 16 February 1911, 17 February 1911, 25 February 1911, 26 February 1911, 27 February 1911, 3 March 1911, 6 March 1911, 12 March 1911, 14 March 1911 (USNM). Panamá- Barro Colorado Island, 16 July 1994, 22 July 1994, 1 August 1994, 11 August 1994, 30 June–5 July 2000, 23–27 July 2000, 7 July 2000, 31 July–4 August 2000 (SEMC); Cerro Campana, 3000', 29 July 1970 (CMNC), 11–15 May 1980 (USNM), 12 March 1972 (EGRC). **PERU:** no further data (USNM). Huanuco- Tingo Maria, 19 July 1968 (BYUC). Loreto- Reserva Alpahuayo Mishana, 27 May 2005 (USNM). Madre de Dios- CICRA Field Station, 272 m, 10 June 2011, 12 June 2011, 13 June 2011, 14 June 2011 (SEMC). San Martin- Río Seco, 27 km W Rioja, 23 September 1938 (SEMC). Total: 330.

#### Cephaloleia dilectans

Taxon classificationAnimaliaColeopteraChrysomelidae

Pic, 1923

http://species-id.net/wiki/Cephaloleia_dilectans

Fig. 120

Cephalolia dilectans Pic 1923: 9. Uhmann 1938a: 410 (noted).
Cephaloleia dilectans Pic. Uhmann 1957b: 18 (catalog); Descarpentries and Villiers 1959a: 139 (types).

##### Description.

Elongate; subdepressed; yellow; elytra with black humeral macula and black transverse band beyond middle which ends on apical ¼. Head: vertex smooth, depressed, with scattered punctures in depressed area, small carina present between base of antennae; frons smooth, not projecting; not depressed between eyes. Antenna: reaches to humerus; slender; antennomere 1 elongate, thicker than others; 2 cylindrical, subequal in length to 1; 3 cylindrical, slightly longer than 2; 4–10 cylindrical, elongate, decreasing in length; 4 shorter than 3; 11 2× length of 10, bluntly pointed at apex; 1–2 punctate; 3–11 setose. Pronotum: transverse; lateral margin straight for basal ½ then rounding to anterior angle, canaliculate; anterior angle with broad, blunt tooth; posterior angle acute; anterior margin emarginate behind head; disc flattened; surface sparsely, coarsely punctate; basal impression absent; pronotal length 1.4–1.5 mm; pronotal width 1.6–1.9 mm. Scutellum: triangular, impunctate. Elytron: lateral margin straight, smooth, slightly laminate; apex rounded, smooth; sutural angle with small tooth; humerus rounded, not produced; slightly constricted behind humerus; moderately punctate-striate, puncture rows confused apically; elytral length 5.0–5.2 mm; elytral width 2.2–2.6 mm. Venter: pro-, meso-, and metasterna impunctate medially, coarsely punctate laterally; abdominal sterna punctate, each puncture with pale seta; suture between sterna 1 and 2 complete; last sternite with apical margin rounded entire in male, truncate in female. Leg: slender; punctate; femur robust; tibia with fringe of setae on inner margin of apex. Total length: 6.8–7.2 mm.

##### Diagnosis.

This species is similar to *Cephaloleia ornatula* and *Cephaloleia strandi*. It can be distinguished by the pronotum without a basal impression.

##### Distribution.

Ecuador, Peru.

##### Type material.

Type: Ecuador, MNHN, not seen.

##### Specimens examined.

**Ecuador:** no further data (MNHN). Imbabura- Cachabé, November 1896 (USNM). Los Ríos- Río Palenque, 47 km S Sto. Domingo, 220 m, 26 August 1997 (CDFA). Pichincha- Chimbo, 1000 ft., August 1897 (USNM). **PERU:** Cuzco- Quince Mil, 27 January 1979 (USNM). Total: 7.

#### Cephaloleia dimidiaticornis

Taxon classificationAnimaliaColeopteraChrysomelidae

Baly, 1869

http://species-id.net/wiki/Cephaloleia_dimidiaticornis

Fig. 121

Cephalolia dimidiaticornis Baly 1869: 370. Gemminger and Harold 1876: 3601 (catalog); Donckier 1899: 548 (catalog); Weise 1911a: 7 (catalog), 1911b: 11 (catalog); Uhmann 1953d: 47 (faunal list).
Cephaloleia dimidiaticornis Baly. Uhmann 1957b: 18 (catalog); Staines and Staines 1999: 523 (Baly species list).

##### Description.

Small; elongate; subparallel; subconvex; reddish-brown; eyes and antennomeres 7–11 darker. Head: vertex sparsely punctate, medial sulcus absent; small carina present between bases of antennae; frons impunctate, not projecting; slightly depressed between eyes. Antenna: ½ body length; slender; antennomere 1 short, transverse; 2–10 cylindrical; 2 elongate, 2× length of 1; 3–4 elongate, subequal in length, each longer than 2; 5–6 elongate, subequal, each shorter than 3; 7–10 transverse, subequal, each shorter than 6; 11 2× length of 10, acutely pointed at apex; 1–6 punctate; 7–11 setose. Pronotum: transverse; lateral margin straight for basal ¾ then rounding to anterior angle, canaliculate; anterior angle rounded, not produced; posterior angle acute; anterior margin emarginate behind head; disc subconvex; surface sparsely, coarsely punctate, punctures more dense laterally; basal impression absent; pronotal length 0.9–1.1 mm; pronotal width 1.2–1.6 mm. Scutellum: triangular, impunctate. Elytron: lateral margin straight, smooth, margined; apex rounded; sutural angle without tooth; humerus rounded, not produced; slightly constricted behind humerus; subconvex with disc flattened; moderately punctate-striate, punctures less impressed apically; elytral length 3.2–3.6 mm; elytral width 1.5–1.7 mm. Venter: pro-, meso-, and metasterna punctate, each puncture with pale seta; abdominal sterna punctate, each puncture with pale seta; suture between sterna 1 and 2 complete; last sternite with apical margin sinuate medially in male; in female emarginate medially. Leg: slender; punctate, each puncture with pale seta; femur robust; tibia with fringe of setae on inner margin of apex. Total length: 4.0–4.9 mm.

##### Diagnosis.

This species is similar to *Cephaloleia latipennis* and *Cephaloleia polita*. It can be distinguished by the elytral puncture rows being regular to the apex and by antennomere 1 being transverse.

##### Host plant.

According to label data, adults have been collected on an unidentified palm (Arecaceae).

##### Distribution.

Brazil (São Paulo), Peru.

##### Type material examined.

Syntype: Peru [handwritten label]/ Cephalolia dimidiaticornis Baly, Peru [blue handwritten label] (BMNH, 1).

##### Specimens examined.

**BRAZIL:** São Paulo- no further data (USNM). **Peru:** no further data (BMNH). Loreto- Tambo Pirana on Río Cochiquinas, 1 July 1978 (USNM). Total: 3.

#### Cephaloleia diplothemium

Taxon classificationAnimaliaColeopteraChrysomelidae

Uhmann, 1951a

http://species-id.net/wiki/Cephaloleia_diplothemium

Fig. 122

Cephaloleia diplothemium Uhmann 1951a: 70. Uhmann 1957b: 18 (catalog); Gaedike and Döbler 1971: 347 (types); Staines 1997b: 413 (Uhmann species list).

##### Description.

Elongate-ovate; flat; head and pronotum black with weak metallic sheen; elytra clearly metallic-green; mouthparts and antennae brownish; legs and sides of abdomen pitch-brown. Head: vertex finely punctate between the eyes, with faint medial carina and fine longitudinal keel between antennae; eyes strongly convex; frons impunctate, not projecting; slightly depressed between eyes. Antenna: slightly longer than head and pronotum combined; slender; antennomere 1 elongate, only slightly thicker than the others, obliquely truncate apically; 2 less than ½ length of 1, subglobose; 3 as long as 1, weakly conical; 4–10 each shorter than 3, cylindrical, elongate, 11 slightly flattened, 2× length of 10, pointed at apex; 1–2 coarsely, densely punctate; 3–11 finely pubescent. Pronotum: 1½ times as wide as long; lateral margin diverging from the base then broadly rounding to anterior angle, slightly canaliculate; anterior angle rounded, slightly produced; posterior angle acute; anterior margin emarginate behind head; disc subconvex; with punctures very fine medially, denser and coarser laterally; basal impression absent; pronotal length 0.6–0.8 mm; pronotal width 1.0–1.3 mm. Scutellum: pentagonal, micropunctate. Elytron: lateral margin straight, smooth, finely margined; apex rounded; sutural angle truncate, without tooth; humerus rounded, not produced; slightly constricted behind humerus; scutellar row long; puncture rows 1–6 are very fine; interspaces flat, 9 slightly raised behind the humerus; pygidium densely punctate, with very weak impression; elytral length 2.2–2.6 mm; elytral width 1.0–1.4 mm. Venter: epipleuron, pro-, and metasterna finely punctate; mesosternum impunctate; abdominal sterna finely punctate, each puncture with pale seta; suture between sterna 1 and 2 totally obsolete; male with apical margin of last sternite emarginate medially, female rounded, entire. Legs: slender; punctate; femur robust; tibia with fringe of setae on inner margin of apex. Total length: 3.0–3.5 mm.

##### Diagnosis.

This species is similar to *Cephaloleia caeruleata* and *Cephaloleia dilatata*. It can be distinguished by the subglobose antennomere 1.

##### Host plant.

*Diplothemium caudescens* Martius (Arecaceae) (Uhmann 1951a).

##### Distribution.

Brazil (Bahia, Goiás, Matto Grosso, Minas Gerais).

##### Type material examined.

Holotype: Brazil, Bahia, Caxandó, Bondar [printed label]/ nr. 4665 [handwritten label]/ Holotyp [red printed label]/ Cephaloleia diplothemium Uh., Det. E. Uhmann (BMNH).

##### Specimens examined.

**Brazil:** no further data (USNM). ?- Taperina (USNM). Bahia- Caxandó (DEI); São Paulo d’Olivenca, March 1883, May 1883 (USNM). Goiás- Jatahy, 1895–1896 (USNM). Minas Gerais- Caraca, December 1885 (USNM). Total: 18.

#### Cephaloleia discoidalis

Taxon classificationAnimaliaColeopteraChrysomelidae

Baly, 1885

http://species-id.net/wiki/Cephaloleia_discoidalis

Fig. 123

Cephaloleia discoidalis Baly 1885: 15. Blackwelder 1946: 719 (catalog); Papp 1953: 16 (catalog); Uhmann 1957a: 18 (catalog); Wilcox 1983: 136 (catalog); Staines 1996: 28 (Central America species); Staines and Staines 1999: 523 (Baly species list); McKenna and Farrell 2006: 10949 (phylogeny).
Cephalolia discoidalis Baly. Donckier 1899: 548 (catalog); Weise 1911a: 7 (catalog), 1911b: 12 (catalog).

##### Description.

Oblong-oval; subconvex; head, antennae, pronotum, and scutellum dark brown; elytra yellowish with dark brown sutural vitta starting at basal margin, dilated just past scutellum, then gradually narrows to just beyond midline, then only suture darkened, and a brown pointed, ovoid, elongate macula on apical ⅓; venter with prosternum brown, meso- and metasterna brown medially and black laterally, abdominal sterna dark medially and pale laterally; legs yellow. Head: vertex densely punctate, each puncture with short pale seta, medial sulcus absent; frons not projecting; keel between antennal bases; not depressed between eyes. Antenna: reaches to humerus; slender; antennomere 1 longer than 2, thickened; 2–3 transverse, subequal in length; 4–10 transverse, decreasing in length; 11 2× length of 10, rounded at apex; 1–4 punctate with scattered setae; 5–11 setose. Pronotum: transverse; lateral margin straight and convergent from base to apex, canaliculate, especially near apex; anterior angle obtuse, produced; posterior angle acute; anterior margin emarginate behind head; disc convex, surface densely and shallowly punctate; basal impression absent; pronotal length 0.9–1.2 mm; pronotal width 1.3–1.7 mm. Scutellum: pentagonal; micropunctate. Elytron: lateral margin straight, smooth, slightly laminate; apex obtusely rounded; sutural angle without tooth; humerus rounded, not produced; slightly constricted behind humerus; punctures large, shallow; humerus nearly impunctate; elytral length 4.0–4.2 mm; elytral width 2.1–2.3 mm. Venter: prosternum impunctate; meso- and metasterna impunctate medially, punctate laterally; abdominal sterna sparsely punctate, each puncture with pale seta; suture between sterna 1 and 2 obsolete medially. Leg: slender; glabrous, impunctate; tibia with fringe of setae on inner margin of apex. Total length: 5.1–5.4 mm.

##### Diagnosis.

This species is similar to *Cephaloleia balyi*, *Cephaloleia deficiens*, *Cephaloleia dorsalis*, *Cephaloleia linkei*, and *Cephaloleia suturalis*. It can be distinguished by the black pronotum and by antennomere 1 being longer than 3.

##### Distribution.

Guatemala, Mexico Honduras, Panama.

##### Type material examined.

Holotype: Type H. T. [white disk with red border]/ Orizaba Mexico, Salle Coll. [printed label]/ 1344 [handwritten pale blue label]/ B.C.A., Col. VI, 2. Cephaloleia discoidalis/ Cephaloleia discoidalis Baly, Mexico [handwritten blue label] (BMNH).

##### Specimens examined.

**GUATEMALA:** no further data (USNM). **HONDURAS:** Santa Bárbara- 13 km SE El Mochito, 22 July 1977, 31 July 1977 (EGRC). **PANAMA:** Chiriquí- BdT Cont. div. on Gualaca Chir. Gr. Hwy, 14 June 1985 (EGRC). Total: 4.

#### Cephaloleia disjuncta

Taxon classificationAnimaliaColeopteraChrysomelidae

Staines, 1998

http://species-id.net/wiki/Cephaloleia_disjuncta

Fig. 124

Cephaloleia disjuncta Staines 1998: 672. Staines 2011: 49 (faunal list).

##### Description.

Elongate; subparallel; subdepressed; yellowish-brown with head and antennae black; pronotum yellowish-brown with black medial macula on anterior margin behind head; scutellum yellowish-brown; elytra yellowish-brown with transverse black band before midline and apical ⅓ black; venter with prosternum yellowish, meso- and metasterna yellowish at middle and black laterally, abdominal sterna 1 and 2 yellowish, sterna 3–5 black with golden setae; legs yellow with femur black at apex, tarsi black. Head: vertex sparsely punctate, medial carina present; frons not projecting; slightly depressed between eyes. Antenna: reaches to humerus; slender; antennomere 1 incrassate, longest; 2 transverse, slightly rounded; 3–10 transverse, subequal in length, each longer than 2; 11 2× length of 10, rounded at apex; 1–2 punctate with scattered setae; 3–11 setose. Pronotum: lateral margin sinuate, canaliculate anteriorly; anterior angle with rounded tooth; posterior angle acute; anterior margin emarginate behind head; disc convex, depressed laterally; surface with scattered large punctures, mostly basally; pronotal length 1.3 mm; pronotal width 1.9 mm. Scutellum: pentagonal. Elytron: lateral margin straight, smooth; apex rounded; sutural angle without tooth; humerus rounded, not produced; slightly constricted behind humerus; puncture rows moderately impressed; elytral length 5.1 mm; elytral width 2.3 mm. Venter: prosternum impunctate; meso- and metasterna impunctate medially, punctate laterally; suture between sterna 1 and 2 almost obsolete. Leg: slender; femur robust; tibia spatulate at apex, with fringe of setae on inner margin of apex. Total length: 6.9 mm.

##### Diagnosis.

This species is similar to *Cephaloleia apicata*. It can be distinguished by the lack of an additional elytral puncture row and by the paler elytra with black transverse bands.

##### Host plant.

Collected from *Vitex copperi* Stanley (Verbenaceae) (Staines 1998).

##### Distribution.

Costa Rica.

##### Type material examined.

Holotype: COSTA RICA, Heredia, Est. Biol. La Selva, 50–150m, 10°26'N, 84°01W, Jan 1994, INBIO-OET/ Vitex cooperi FOT-16–30, 5 Enero 1994// bar code 068725/ HOLOTYPE Cephaloleia disjuncta Staines 1997 [red label] (INBIO).

#### Cephaloleia distincta

Taxon classificationAnimaliaColeopteraChrysomelidae

Baly, 1885

http://species-id.net/wiki/Cephaloleia_distincta

Fig. 125

Cephaloleia distincta Baly 1885: 10. Blackwelder 1946: 719 (catalog); Papp 1953: 16 (catalog); Uhmann 1957a: 18 (catalog), 1964a: 403 (catalog); Gaedike and Döbler 1971: 348 (types); Wilcox 1983: 137 (catalog); Staines 1996: 28 (Central America species), 2004: 312 (host plants), 2010: 30 (types), 2011: 49 (faunal list); Staines and Staines 1997: 7 (types), 1999: 523 (Baly species list); Samuelson 1996: 290 (morphology); McKenna and Farrell 2005: 119 (phylogeny), 2006: 10949 (phylogeny); García–Robledo et al. 2013a: 3 (biology); Schmitt and Frank 2013: 58 (biology).
Cephalolia distincta Baly. Donckier 1899: 548 (catalog); Weise 1910b: 83 (noted), 1911a: 7 (catalog), 1911b: 10 (catalog), 1921a: 263 (noted); Uhmann 1935a: 103 (redescription), 1936b: 482 (key).
Cephalolia nigripes Pic 1926a: 9 (type: Costa Rica, Turrialba, MNHN, not seen).
Cephaloleia nigripes Pic. Uhmann 1935a: 104 (comparative note); Blackwelder 1946: 719 (catalog); Papp 1953: 18 (catalog); Uhmann 1957a: 18 (catalog), 1964a: 403 (catalog); Descarpentries and Villiers 1959a: 139 (types).
Cephalolia distincta nigripes Pic. Uhmann 1936b: 482 (key).

##### Description.

Large; elongate, subparallel; subconvex; reddish-brown, with antennae, eyes, and parts of legs darker; elytra with narrow black sutural vitta present on some specimens; venter with abdominal sterna dark, paler laterally; legs variable in color- all dark or tibia and tarsi dark, femur pale. Head: vertex finely, sparsely punctate, more punctate toward eyes, medial sulcus present; frons not projecting; carina between antennal bases on front; depressed between eyes. Antenna: reaches to humerus; robust; female with antennomere 1 thick, compressed at base, 2–11 elongate; male with 1–4 compressed, dilated, 1 subclavate, 2–4 subtriangular, 2 short, wider than long, 3–4 less dilated, each longer than 2, 5–10 transverse, subequal in length, each shorter than 4, 11 2× length of 10, pointed at apex; 1–4 punctate; 5–11 setose. Pronotum: slightly wider than long; lateral margin convergent to anterior angle, margined; anterior angle rounded, slightly produced; posterior angle angulate; anterior margin emarginate behind head; disc subconvex; disc sparsely punctate; punctation more dense laterally; basal impression absent; pronotal length 1.4–1.9 mm; pronotal width 2.0–2.7. Scutellum: elongate, pentagonal, apex acute, impunctate. Elytron: lateral margin straight, smooth; apex rounded; sutural angle without tooth; humerus rounded, not produced; slightly constricted behind humerus; subconvex; strongly punctate-striate, puncture rows converge and unite at apex; humerus almost impunctate; elytral length 5.9–7.3 mm; elytral width 2.8–3.6 mm. Venter: prosternum impunctate medially, rugose laterally; meso- and metasterna impunctate medially, punctate laterally; abdominal sterna punctate, each puncture with pale seta; suture between sterna 1 and 2 complete; last sternite with apical margin emarginate medially in male, rounded in female. Leg: robust; sparely punctate; femur and tibia with pale seta in each puncture; tibia with fringe of setae on inner margin of apex. Total length: 7.6–9.6 mm.

**Figures 125–133.**
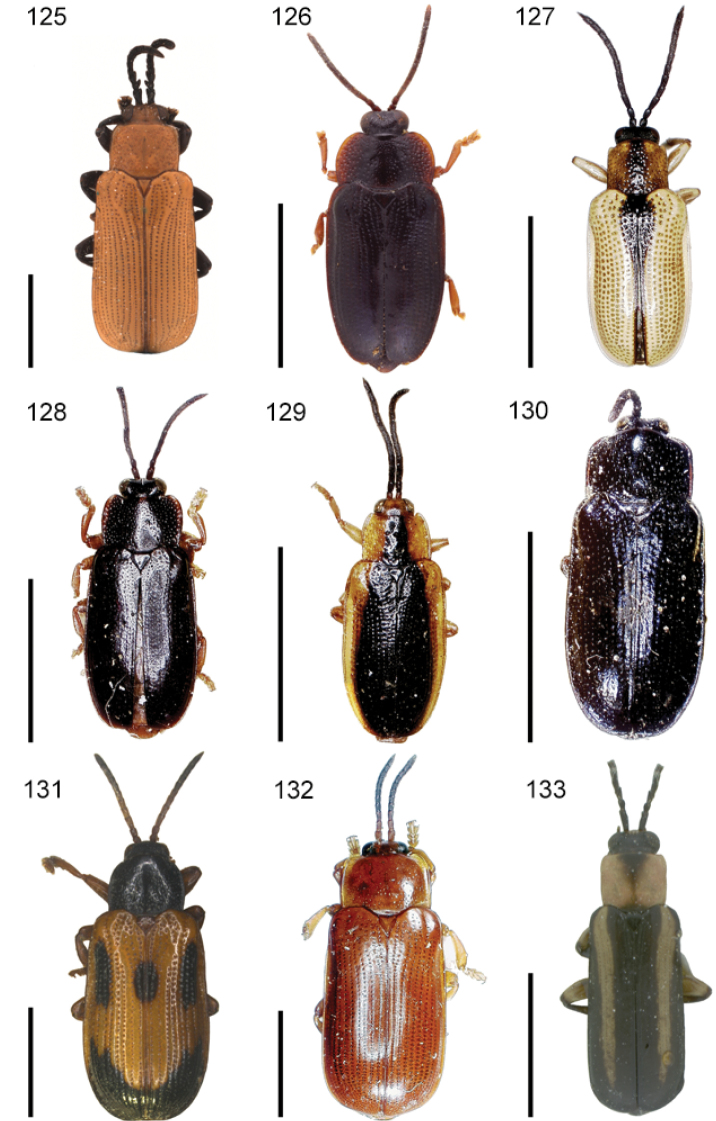
Habitus. **125** Cephaloleia *distincta* **126** *Cephaloleia donckieri* **127** *Cephaloleia dorsalis* **128** *Cephaloleia elaeidis* **129** *Cephaloleia elegantula* **130** *Cephaloleia emarginata* **131** *Cephaloleia emdeni* **132** *Cephaloleia erichsonii* **133** *Cephaloleia erugatus*. Scale bars equal 3 mm.

##### Diagnosis.

This species is similar to *Cephaloleia castanea* and *Cephaloleia nigricornis*. It can be distinguished by antennomere 3 being shorter than 1 and by the elytral puncture rows converging and uiting apically.

##### Host plant.

Adults have been collected on *Calathea* sp. (Marantaceae). Adults and larvae have been collected on *Heliconia imbricata* (Kuntze) Baker (Staines 1996); *Heliconia mariae* Hook. (Heliconiaceae) (García–Robledo et al. 2013a); *Alpinia purpurata* K. Schum. (Zingiberaceae), *Heliconia latispatha* Benth., *Heliconia rostrata* Ruiz & Pav., *Heliconia stricta* Huber, *Heliconia wagneriana* Peterson (Heliconiaceae), *Musa paradisiaca* L. (Musaeae) (Schmitt and Frank 2013); *Cephaloleia crotalifera* S. Watson, *Pleiostachya leiostachya* (Donn. Sm.) Hammel (Marantaceae).

##### Distribution.

Colombia, Costa Rica, Panama.

##### Type material examined.

Syntypes: V. de Chiriqui, 25–4000 ft., Champion [printed label]/ Paratipo [handwrittenred label]/ F. Monros Collection 1959 [printed label]/ Cephaloleia distincta Baly, J. S. Baly det. [handwritten pink label] (USNM, 3; AMNH, 1). Bugaba, Panama, Champion (AMNH, 1); Bugaba, Panama Champion/ Collection Biolog. Centr. Americana/ SYNTYPE *Cephaloleia distincta* Baly, C. L. Staines 2002 [red label]/ Paratype Cephaloleia distincta [blue label] (ANSP, 1); Bugaba, 800–1,500 ft., Champion/ Collection Biolog. Centr. Americana/ SYNTYPE *Cephaloleia distincta* Baly, C. L. Staines 2002 [red label]/ Paratype Cephaloleia distincta [blue label] (ANSP, 1).

##### Specimens examined.

**COLOMBIA:** Valle de Cauca- 1898 (USNM). **COSTA RICA:** Alajuela- Atenas (USNM); Río San Lorencito, 900 m, Res. For. San Ramón, 5 km N Col. Palmerena, March 1990 (INBIO), 8 March 1990 (MUCR); Turrialba, CATIE, 28–29 June 1986 (BYUC). Cartago- Aquiares nr. Santa Cruz, 9 km NW Turrialba, 1500 m, 16 May 1985 (EMEC); Quebrada Segunda Ref. Nac. Fauna Silv. Tapantí, 1250 m, April 1992 (INBIO); Turrialba, CATIE, 20 May 1979, 19 May 1979 (CMNC), 4–13 August 1970 (USNM), 26–29 June 1986 (BYUC); Turrialba (MNHN), 26 May 1951, 31 May 1951, 19 June 1951, 20 June 1951 (USNM), 21 August 1966 (FSCA); Turrialba, Santa Teresita, Monumento Nacional Guayabo, 1100–1200 m (INBIO); Turrialba, Tayutic, Grano de Oro, Chirripo, 1100–1200 m (INBIO); Ref. Nac. Fauna Silv. Tapantí, 1250 m, August 1991 (INBIO); Tucurrique (USNM); Valle Orosí, Tapantí, 25 May 1991 (MUCR). Guanacaste- Río San Lorenzo, 1050 m, Tierras Morenas, Z. P. Tenorio, April 1991, 23 March–21 April 1992 (INBIO). Heredia- Transecto. Braulio Carillo N. P., October 1989 (INBIO); Rara Avis Biological Station, 17 November 2011 (USNM). Limón- Hamburg Farm, Reventazón, Ebene Limón, August 1924 (USNM), 1 February 1933 (MUCR); Est. Hitoy Cerere, 100 m, R. Cerere, Res. Biol. Hitoy Cerere, January 1991, 4–20 December 1991, 19–29 April 1992 (INBIO); Pococí, Colorado, Sector Cerro Cocorí, 30 km N Cariari, 100–200 m (INBIO); Amburi, Sendero Soki, 0–100 m (INBIO); Talamanca, Amburi, 0–100 m (INBIO). Puntarenas- Alajuela, Monteverde For. Res., 1600 m, 17–18 August 1976 (CASC); Coto Brus, Las Cruces Biological Station, 10 March 2012 (USNM); Est. La Casona, Res. Biol. Monteverde, 1520 m, September 1990 (INBIO); Fca. Las Cruces, San Vito de Java, 27 June 1969 (USNM); F. Las Cruces, 6 km. S. San Vito, 1200–1400 m., 21–25 August 1976 (CASC); 22 mi SW San Vito, 11 August 1969 (USNM); Est. Sirena, 0–100 m, P. N. Corcovado, July 1991 (INBIO); A. C. O., Golfito, F. Las Cruces, Fca Ilama, Jiménez, Alpízar, 1400–1500 m (INBIO); Coto Brus, Pittier, Palmira, 1100–1200 m (INBIO); Finca Las Alturas, 1300–1400 m (INBIO); F. La Cruces, Laguna Gamboa, 1400–1500 m (INBIO); Estació Equinas, Peninsula de Osa, 0–100 m (INBIO); Sendero, Playa San Josecito, 100–200 m (INBIO); Est. Boscosa, 0–100 m (INBIO); Guacimal, Finca Buen Amigo, Monteverde, 4 km S Reserva, 1000–1100 m (INBIO); A. C. A., Central, Reserva Bosque Eterno de los Niños, 1500–1600 m (INBIO); Rincón de Osa, 23–26 June 2001 (SEMC). San José- La Caja, 8 km We San José, 1934 (DEI); Farm La Caja (DEI). **PANAMA:** ?- Petrelios, 25 May- June 1935 (CASC). Chiriquí- Fortuna, 19 May 1978 (EGRC, USNM), 20 May 1978 (EGRC); Dst. Ren., Oest. Clara, 13–22 May 1977, 5000' (EGRC); Reserva Fortuna, Continental Divide Trail, 25 May 1993, 26 May 1993, 29 May 1993 (AJGC, CDFA, EGRC); Reserva La Fortuna, Finca la Suiza, 25 May 1993 (EGRC); Santa Clara, 23–25 May 1980 (EGRC, USNM); 2 km N Sta. Clara, 24–25 May 1977 (CMNC), 20 May 1977 (CMNC). Colón- Ft. Davis, 21 January 1957 (USNM). Panamá- Cerro Campana, 850 m, 1 August 1970 (CMNC), 11–15 May 1980 (EGRC, USNM), 24 June 1973 (USNM), 6 April 1971, 13 July 1971 (EGRC), 17 May 1993 (CDFA), 2 June 1993 (AJGC). Total: 223.

#### Cephaloleia donckieri

Taxon classificationAnimaliaColeopteraChrysomelidae

Pic, 1926c

http://species-id.net/wiki/Cephaloleia_donckieri

Fig. 126

Cephalolia donckieri Pic 1926c: 10.
Cephaloleia donckieri Pic. Uhmann 1957b: 18 (catalog); Descarpentries and Villiers 1959a: 139 (types).

##### Description.

Oblong-subovate; flattened; somewhat shining; head and pronotum black, elytra bluish; antennae at base, pronotum laterally, elytra at humeri, legs, and body red. Head: vertex densely, strongly punctate, medial carina present; frons not projecting; depressed between eyes. Antenna: reaches to humerus; slender; antennomeres 1–2 thicker than others; 1 incrassate, elongate; 2 cylindrical; ½ length 1; 3 cylindrical; longest; 4 cylindrical; elongate, subequal in length to 1; 5–10 cylindrical, decreasing in length; 11 2× length of 10, broadly rounded at apex; 1–4 punctate with scattered setae; 5–11 setose. Pronotum: transverse; lateral margin arcuate, finely serrate, margined; anterior angle rounded, slightly produced; posterior angle acute; anterior margin emarginate behind head; basal margin biangulate; disc subconvex, callused medially; surface coarsely, densely punctate laterally, finely, irregularly punctate medially; basal impression absent; pronotal length 1.0–1.2 mm; pronotal width 1.7–2.1 mm. Scutellum: broadly pentagonal; impunctate. Elytron: lateral margin straight, smooth, margined; apex rounded; sutural angle without tooth; humerus rounded, not produced; slightly constricted behind humerus; moderately punctate-striate; costate at apex; pygidium visible; elytral length 3.2–3.6 mm; elytral width 2.0–2.2 mm. Venter: pro-, meso-, and metasterna impunctate medially, punctate laterally, each puncture with pale seta; abdominal sterna punctate, each puncture with pale seta; suture between sterna 1 and 2 complete; last sternite with apical margin emarginate medially in female. Leg: slender; punctate, each puncture with pale seta; tibia with fringe of setae on inner margin of apex. Total length: 4.8–5.3 mm.

##### Diagnosis.

This species is similar to *Cephaloleia depressa*, *Cephaloleia elaeidis*, *Cephaloleia tarsata*, and *Cephaloleia zikani*. It can be distinguished by the lack of a medial fovea on the vertex of the head, by the serrulate lateral margins of the pronotum, by the smooth apical margins of the elytra, and by the incrassate antennomere 1.

##### Distribution.

French Guiana, Suriname.

##### Type material examined.

Syntypes: Cayenne [handwritten]/ depressa con coll. Donckier [handwritten label]/ type [handwritten label]/ Type [red printed label]/ Museum Paris Coll. M. Pic [blue printed label]/ Cephaloleia donckieri Pic [printed label]/ Syntype [red printed label]/ MNHN EC 2606 [printed label] (MNHN, 1); Type [[handwritten label]/ Cephaloleia donckieri Pic [printed label]/ Syntype [red printed label]/ MNHN EC 2607 [printed label] (MNHN, 1).

##### Specimens examined.

**French Guiana:** Gampoi, 1900 (USNM); Rivere Lunier, 1899 (USNM); Saul, 7 km N 1 km NW Les Eaux Clairs along Rue de Belizon trail, 280 m, 4–8 January 1997 (SEMC, USNM). **SURINAME:** Brokopondo- Brownsberg Nature Preserve, Wili Creek Trail, 80 m, 23–25 June 1999 (SEMC). Commewijne- Akinioseola, CELOS Camp, 39 km SE Suriname River Bridge, road to Redi Doti, 40 m, 29 June-3 July 1999 (SEMC, USNM). Marowijne- Perica, 70 km E Paramaribo on East-West Road, 5 m, 31 May–5 June 1999 (SEMC, USNM). Total: 24.

#### Cephaloleia dorsalis

Taxon classificationAnimaliaColeopteraChrysomelidae

Baly, 1885

http://species-id.net/wiki/Cephaloleia_dorsalis

Fig. 127

Cephaloleia dorsalis Baly 1885: 15. Blackwelder 1946: 719 (catalog); Papp 1953: 16 (catalog); Uhmann 1957a: 18 (catalog); Wilcox 1983: 137 (catalog); Staines 1996: 29 (Central America species), 2004: 312 (host plants), 2011: 49 (faunal list); Staines and Staines 1997: 8 (types), 1999: 523 (Baly species list) Hsaio and Windsor 1999: 43 (phylogeny); Flowers and Hanson 2003: 51 (distribution); McKenna and Farrell 2005: 119 (phylogeny), 2006: 10949 (phylogeny); Meskins 2008: 163 (host plants), 2011: 483 (food web); García–Robledo and Horvitz 2009: 116 (host plants), 2011: 978 (biology), 2012: 40 (biology); García–Robledo et al. 2010: 51 (larva, biology), 2013a: 3 (biology), 2013b: 193 (biology).
Cephalolia dorsalis Baly. Donckier 1899: 549 (catalog); Weise 1911a: 8 (catalog), 1911b: 12 (catalog), 1921a: 263 (noted).

##### Description.

Elongate; subconvex; subparallel; head, antennae, and pronotum black; elytra yellow with black sutural vitta at base extending to puncture row 3 then narrowing to only suture and darkened after middle; venter with pro-, meso-, and metasterna red medially, dark laterally; legs yellow. Head: vertex densely punctate, faint medial carina present; keel present between antennae; frons not projecting; not depressed between eyes. Antenna: more than ½ body length; slender; antennomere 1 thickened, subequal in length to 2; 2 elongate; 3 elongate, as long as 1 and 2 combined; 4–10 elongate, decreasing in length; 11 2× length of 10, rounded at apex; 1–2 punctate with scattered setae; 3–11 setose. Pronotum: slightly wider than long; lateral margin straight then rounding to anterior angle, strongly margined; anterior angle slightly produced, rounded; posterior angle angulate; anterior margin weakly emarginate behind head; disc subconvex; disc strongly punctate; basal impression absent; pronotal length 0.7–1.0 mm; pronotal width 1.1–1.6 mm. Scutellum: pentagonal; impunctate. Elytron: lateral margin straight, smooth, margined; apex rounded; sutural angle without tooth; humerus rounded, not produced; slightly constricted behind humerus; moderately convex; shallowly punctate-striate; puncture rows complete; elytral length 3.3–4.0 mm; elytral width 1.9–2.3 mm. Venter: pro-, meso-, and metasterna impunctate medially, punctate laterally; abdominal sterna punctate, each puncture with pale seta; suture between sterna 1 and 2 obsolete medially; last sternite with apical margin u-shaped in male, ovoid in female. Leg: slender; femur punctate on top; tibia with fringe of setae on inner margin of apex. Total length: 4.4–5.4 mm; females are larger than males.

##### Diagnosis.

This species is similar to *Cephaloleia balyi*, *Cephaloleia deficiens*, *Cephaloleia linkei*, and *Cephaloleia suturalis*. It can be distinguished by the yellowish pronotum with black longitudinal vitta and by antennomere 3 being larger than 1 and 2 combined.

##### Comments.

Preliminary analysis of the CO1 gene indicates that cryptic species may be present under the current application of this species name. Further work is needed to resolve this question.

##### Host plant.

Adults have been collected on *Costus* sp. (Costaceae) (Staines 1996); *Renealmia* sp. (Zingiberaceae) (McKenna and Farrell 2005); *Cephaloleia pulverulentus* C. Presl. (Meskins et al. 2008); *Cephaloleia bracteatus* Gleason, *Cephaloleia laevis* Ruiz and Pav., *Cephaloleia malortieanus* H. Wendl. (García–Robledo and Horvitz 2009); *Cheilocostus speciosus* (J. Koenig) C. D. Specht (Costaceae).

##### Immatures.

Color when live (Figs 35–38) pale yellowish, margins translucent; venter pale yellowish. Color when dead dirty-brown with paler margins. Pronotum surface of central elevation micropustulate; with two diagonal carinae laterally extending to lateral margin, carinae wide at base, narrowing apically to sharp point; surface laterally punctate. Meso- and metanota with base elevated, sloping back to apex, narrowing laterally into sharp point; punctate laterally. Abdominal tergites 1–6 wider than long, decreasing in width; base elevated, sloping back to apex, ending in sharp point; punctate laterally. Tergites 7–9 with two diagonal carinae on each side which extend to lateral margin. Spiracles just off central elevation, with margins darkened. Venter with surface of expansions punctate, rugose-striate. Head surface rugose-punctate; labrum with surface alutaceous, without setae; clypeus with fringe of long setae at apex, with four setae on apical ½, surface alutaceous; mandibles tridentate; maxillary palps with 2 palpomeres and short, robust setae at apex; maxilla robust, clavate, with fringe of long setae at apex; labium densely setose. Antenna with antennomere 1 short, robust; 2 narrower than 1, transverse; 3 elongate, cylindrical, narrower than 2, with fringe of short setae at apex. Pro- and mesosterna wider than long; slightly depressed medially; surface rugose-striate. Metasternum longer than others; depressed medially; with suture along apical margin. Abdominal sternites 1–8 wider than long; decreasing in width; laterally with curved sulcus dividing the sternite into thirds; sterna 9–10 fused, rounded at apex. Leg: femur short, robust; tibiotarsus subconical, with a strong claw and eight setae at apex. Total length:6.4–6.7 mm; width 4.3–4.4 mm. (García–Robledo et al. 2010).

##### Biology.

Eggs are about 2.5 mm long and are laid singly or in clusters of two or more on host plant petioles, the surface of bracts, or the inner surface of inflorescence bracts and are covered with frass. Eggs hatch in 10 to 14 days. The larvae have two instars the first lasting 10 to 18 days and the second 36 to 48 days. The pupal stage lasts 14 to 20 days. Adults live about 157 days (García–Robledo et al. 2010).

##### Distribution.

Costa Rica, Guatemala, Panama.

##### Type material examined.

Syntype: Bugaba, 800–1500 ft., Champion [printed label]/ Paratipo [handwritten red label]/ F. Monros Collection 1959 [printed label]/ Cephaloleia dorsalis Baly, J. S. Baly det. [handwritten pink label] (USNM, 1).

##### Specimens examined.

**COSTA RICA:** Alajuela- 20 km S Upala, 11–20 July 1991 (BYUC); Upala, Sector San Ramón de Dos Ríos, 1.5 km NW Hacienda Nueva Zelandia, 600–700 m (INBIO). Cartago- Quebrada Segunda, Ref. Nac. Fauna Silv. Tapantí, 1250 m, April 1992 (INBIO). Guanacaste- Est Cacao, 1000–1400 m, Lado SO Vol. Cacao, P.N. Guan., 21–29 May 1992 (INBIO); Est. Pitilla, 700 m, 9 km S Sta. Cecilia, P.N. Guanacaste, February 1990, September 1991, (INBIO); Río San Lorenzo, 1050 m, Tierras Morenas, Z. P. Tenorio, 23 March- 21 April 1992 (INBIO); Volcán Cacao, 1100 m, Est. Mengo, pasture, 11 September 1989 (INBIO); 3 km SE Río Naranjo, 19 May 1993 (BYUC). Heredia- Est. El Ceibo, Braulio Carillo, N.P., 400–600 m, November 1989 (INBIO); La Selva Biol. Sta., 2 km. S. Pt. Viejo, 3–5 June 1984 (EGRC); Est. Magasasay, 200 m, P. N. Braulio Carillo, May 1991 (INBIO); Rara Avis Biological Station, 9 November 2011, 13 November 2011, 17 November 2011 (USNM). Limón- Amubri, 70 m, Talamanca, 5–26 January 1993 (INBIO); Sector Cerro Cocorí, Fca. de E. Rojas, 150 m, April 1991, May 1991, August 1991, October 1991, November 1991, December 1991, January 1992, 31 January- 21 February 1992, March 1992, 26 March- 24 April 1992, 28 May- 17 June 1992, 26 June- 16 July 1992, October 1992, 9–30 November 1992, December 1992, January 1993, February 1993, April 1993, May 1993 (INBIO); Río Sardinas, 10 m, R. N. F. S., Barra del Colorado, 25 August 1992, 10 October 1992 (INBIO); Valle de la Estrella Pandora, 17–20 February 1984 (CMNC); A. C. Llanuras del Tortuguero, Pococí, 0–100 m (INBIO); Pococí, Sector Cerro Cocorí, 30 km N Cariari, 100–200 m (INBio). Puntarenas- Fca Cafrosa, Est Las Mellizas, P.N. Amistad, 1300 m, April 1991 (INBIO); Finca Las Cruces, 6 km. S. San Vito de Java, 4200 ft., 28 September- 2 October 1986 (FSCA); Est Sirena, 0–100 m, P.N. Corcovado, December 1989, June 1991, July 1991, September 1991, October 1991, June 1992 (INBIO); Estación Altamira, 1 km S Cerro Biolley, 1400–1500 m (INBIO); Osa, Sierpe, Rancho Quemando, 200–300 m (INBIO). San José- Perez Zeledón, Santa Elena Las Nubes, 1200–1300 m (INBIO). **GUATEMALA:** Zacapa- 3.5 km S.E. La Unión, 1500 m, 4 June 1991 (CMNC), 23–25 June 1993, 25–27 June 1993, 27 June 1993 (SEMC). **PANAMA:** Bocas del Toro- 6 km N Punta Peña, 28 May 1993 (CDFA). Chiriquí- Dst. Recacimiento, Santa Clara, 4000–4200', 4 July 1976 (EGRC); Reserva Fortuna, Continental Divide Trail, 26 May 1993, 29 May 1993 (CDFA, EGRC). Coclé- Cerro Gaital, 4000', 1 June 1993 (AJGC, CDFA). Colón- Skunk Hollow nr. Ft. Sherman, 28 May 1980 (EGRC). Panamá- Cerro Campana, 29 July 1970 (CMNC), 30 May 1970, 11 May 1974, 11–15 May 1980 (EGRC), 17 May 1993 (CDFA). Total: 144.

#### Cephaloleia elaeidis

Taxon classificationAnimaliaColeopteraChrysomelidae

Maulik, 1924

http://species-id.net/wiki/Cephaloleia_elaeidis

Fig. 128

Cephaloleia elaeidis Maulik 1924: 245. Maulik 1937: 132 (host plants); Lepesme 1947: 529 (host plants); Uhmann 1948a: 220 (noted), 1957b: 18 (catalog), 1964a: 403 (catalog); Risbec 1950: 380 (host plants); Bondar 1954: 18 (host plants); Speyer 1954: 365 (host plant); Lima 1955: 202 (faunal list); Menezes 1957a: 86 (pest status); Sandino 1972: 77 (noted); Mariau 1999: 233 (noted), 2001: 132 (noted).
Cephalolia elaeidis Maulik. Lima 1927: 189 (faunal list), 1936: 325 (faunal list); Bondar 1931: 134 (biology), 1940c: 847 (biology); Guérin 1953: 97 (faunal list).
Cephalolia elacidis Maulik. Silva et al. 1985: 36 (misspelling, faunal list).

##### Description.

Elongate; subparallel; subconvex; shining black, elytral and pronotal margins, legs, and abdominal sterna pitchy-brown; antennomeres 1–2 pitchy-brown, 3–11 darker. Head: vertex punctate, with longitudinal carina; eyes strongly convex; frons not projecting; depressed between eyes. Antenna: reaches to humerus; slender; antennomere 1 incrassate, elongate; 2 elongate, shorter than 1; 3 longest; 4 shorter than 2; 3–6 cylindrical; 7–10 transverse, subequal in length, each shorter than 4; 11 subequal in length to 10, pointed at apex; 1–2 punctate with scattered setae; 3–11 setose. Pronotum: quadrate; lateral margin straight then rounding to anterior angle, serrulate, margined; anterior angle broadly rounded, slightly produced; posterior angle acute; anterior margin weakly emarginate behind head; disc subconvex; surface with coarse punctures laterally and fine punctures throughout, longitudinal medial line almost impunctate; basal impression absent; pronotal length 1.0–1.3 mm; pronotal width 1.3–1.4 mm. Scutellum: cuneiform; acutely pointed at apex; alutaceous. Elytron: lateral margin straight, smooth, margined; apex rounded, serrate; sutural angle without tooth; humerus rounded, not produced; slightly constricted behind humerus; moderately punctate-striate, punctures becoming confused apically; elytral length 3.2–3.7 mm; elytral width 1.5–1.8 mm. Venter: pro- and mesosterna punctate; metasternum impunctate medially, punctate laterally; abdominal sterna punctate, each puncture with pale seta; suture between sterna 1 and 2 complete; last sternite with apical margin truncate in female, deeply emarginate medially in male. Leg: slender, short; punctate, each puncture with pale seta; tibia with fringe of setae on inner margin of apex; tarsi short; tarsal claw slightly projecting from tarsomere 3. Total length: 4.5–5.0 mm.

##### Diagnosis.

This species is similar to *Cephaloleia depressa*, *Cephaloleia donckieri*, *Cephaloleia tarsata*, and *Cephaloleia zikani*. It can be distinguished by the lack of a medial fovea on the vertex of the head, by the serrulate lateral margins of the pronotum, by the serrulate apical margins of the elytra, by antennomere 1 being incrassate, and by the larger size.

##### Host plant.

*Elaeis guineensis* Jacq., *Geonoma* sp. (Arecaceae) (Maulik 1924).

##### Distribution.

Brazil (Bahia, São Paulo), Ecuador.

##### Type material examined.

Holotype: Brazil, Bahia, Bondar [printed label]/ Type [red printed label]/ Cephaloleia elaeidis Mlk., Maulik det. [handwritten label] (BMNH).

##### Specimens examined.

**Brazil:** ?- Retiro (?), 8 March 1912 (USNM). São Paulo- Cantareira, September 1939 (USNM). **Ecuador:** Napo- above Papallacta Paramo, 4000 m, 14 February 1983 (USNM);Sacha Lodge, 270 m, 12–22 February 1994, 13–23 June 1994, 25 July- 3 August 1994 (SEMC); San Rafael Falls, 1100 m, 5–6 August 1998 (USNM). Pichincha- E Sto. Domingo, 6–12 May 1990, 8–14 May 1988 (BYUC, USNM); Tinalandia, July 1983 (BYUC), 850 m, 2 February 1983 (USNM); Tinalandia, 15 km SE Santo Domingo de los Colorados, 30 June 1982 (USNM); Tinalandia, nr. St. Domingo, July 1983 (USNM); 12 km E Sto. Domingo, ca. 2500 ft., 11–17 May 1986 (TAMU). Orellana- Estación Cientifica Yasuni, 215 m, 6–10 September 1999 (EGRC). Total: 47.

#### Cephaloleia elegantula

Taxon classificationAnimaliaColeopteraChrysomelidae

Baly, 1885

http://species-id.net/wiki/Cephaloleia_elegantula

Fig. 129

Cephaloleia elegantula Baly 1885: 17. Blackwelder 1946: 719 (catalog); Papp 1953: 16 (catalog); Uhmann 1957a: 18 (catalog); Wilcox 1983: 137 (catalog); Staines 1996: 30 (Central America species), 2011: 49 (faunal list); Staines and Staines 1999: 524 (Baly species list).
Cephalolia elegantula Baly. Donckier 1899: 549 (catalog); Weise 1911a: 8 (catalog), 1911b: 12 (catalog).

##### Description.

Small; elongate; subparallel; subconvex; antennae, except antennomere 1, black; head and legs yellow; pronotum reddish-yellow with black central macula; elytra yellow with black medial vitta expanding broadly to apex, recurving slightly in graceful arch almost following contour of elytral margin at apical 1/5; venter yellow except meso- and metasterna dark laterally. Head: finely punctate near eyes, medial sulcus present; frons not projecting; not depressed between eyes. Antenna: ⅔ body length; slender; antennomeres 1–7 cylindrical, elongate; 1 shorter than 2; 2 robust; 3 longest; 4–7 decreasing in length; 8–10 transverse, subequal in length, each shorter than 7; 11 2× length of 10, pointed at apex; 1–5 punctate with scattered setae; 6–11 setose. Pronotum: transverse; lateral margin straight from base to beyond middle then obliquely rounding to anterior angle, margined; anterior angle moderately obtuse, produced; posterior angle acute; anterior margin emarginate behind head; disc subconvex; surface punctate, more so laterally; basal impression absent; pronotal length 0.7–0.8 mm; pronotal width 1.0–1.2 mm. Scutellum: pentagonal; impunctate. Elytron: lateral margin straight, smooth, margined; apex rounded; sutural angle without tooth; humerus rounded, not produced; slightly constricted behind humerus; puncture rows moderately impressed; row 10 removed from margin for entire length; elytral length 2.7–3.2 mm; elytral width 1.5–1.7 mm. Venter: pro-, meso-, and metasterna impunctate medially, punctate laterally; abdominal sterna punctate, each puncture with pale seta; suture between sterna 1 and 2 complete; last sternite with apical margin emarginate medially in male, rounded in female. Leg: slender; punctate, each puncture with pale seta; femur robust; tibia with fringe of setae on inner margin of apex. Total length: 3.7–4.2 mm.

##### Diagnosis.

This species is similar to *Cephaloleia chevrolatii* and *Cephaloleia partita*. It can be distinguished by antennomere 1 being elongate and by the elytral humerus being impunctate.

##### Host plant.

According to label data, adults have been collected in palm frond (Areaceae).

##### Distribution.

Brazil(?), Costa Rica, Panama.

##### Type material examined.

Lectotype male: SYNTYPE [white disk with blue border]/ V. de Chiriqui, 25–400 ft., Champion [printed label]/ B.C.A. Col. VI, 2. Cephaloleia elegantula Baly [printed label]/ Cephaloleia elegantula Baly, Panama [handwritten label]/ Lectotype Cephaloleia elegantula Baly, des. C. L. Staines 1994 [red label] (BMNH).

##### Specimens examined.

(?)**BRAZIL:** ?- Chapada (USNM). **Costa Rica:** San José- km 117 Pan American Hwy, 19 km N San Isidro, 20–25 June 1997 (SEMC). Puntarenas- 27 km S. Puerto Jimenez, Rio Piro, 75 m, November 1990 (BYUC). **PANAMA:** no further data (USNM). Bocas del Toro- Reserva La Fortuna, 28 May 1993 (EGRC). Chiriquí- Bugaba (BMNH). Herrera- Cerro Alto Higo, el. 900 m, 23 May 1992 (EGRC). Total: 11.

#### Cephaloleia emarginata

Taxon classificationAnimaliaColeopteraChrysomelidae

Baly, 1875a

http://species-id.net/wiki/Cephaloleia_emarginata

Fig. 130

Cephaloleia emarginata Baly 1875a: 74. Uhmann 1957b: 18 (catalog); Staines and Staines 1999: 524 (Baly species list).
Cephalolia emarginata Baly. Gemminger and Harold 1876: 3601 (catalog); Donckier 1899: 549 (catalog); Weise 1911a: 8 (catalog), 1911b: 12 (catalog).

##### Description.

Elongate; subconvex; shining; metallic blue, antennae black. Head: vertex punctate, carina present between antennal bases; frons not projecting; depressed between eyes. Antenna: as long as head and pronotum combined; robust; antennomeres 1–3 transverse, subequal in length; 4–10 transverse, subequal in length, each shorter than 3; 11 2× length of 10, rounded at apex; 1–2 punctate with scattered setae; 3–11 setose. Pronotum: transverse; lateral margin very slightly converging from base to apex, canaliculate; anterior angle rounded, not produced; posterior angle acute; anterior margin emarginate behind head, with small tubercle in notch; disc subconvex; surface with large, variolose punctures, punctures sparse on disc, more dense laterally; basal impression absent; pronotal length 1.0–1.3 mm; pronotal width 1.5–1.9 mm. Scutellum: broadly subpentagonal, acutely pointed at apex; alutaceous. Elytron: lateral margin straight, smooth, margined; apex rounded, finely serrate; sutural angle without tooth; humerus rounded, not produced; slightly constricted behind humerus; regularly but not coarsely punctate-striate, punctures nearly obsolete apically; elytral length 3.5–3.9 mm; elytral width 1.7–2.1 mm. Venter: pro-, meso-, and metasterna punctate; abdominal sterna punctate, each puncture with pale seta; suture between suture 1 and 2 complete; last sternite with apical margin broadly truncate-emarginate medially. Leg: slender; punctate; tibia with fringe of setae on inner margin of apex. Total length: 4.5–5.6 mm.

##### Diagnosis.

This species is similar to *Cephaloleia nitida* and can be distinguished by the serrulate apical margin of the elytra.

##### Distribution.

Brazil (Pará), Ecuador.

##### Type material examined.

Syntype: Para, Santarem [printed label]/ Cephaloleia emarginata Baly, Para [blue handwritten label] (BMNH, 1).

##### Specimens examined.

**Brazil:** Pará- no further data (USNM). **Ecuador:** Napo- Río Napo, Sacha Lodge, 4–14 May 1992 (BYUC). Total: 2.

#### Cephaloleia emdeni

Taxon classificationAnimaliaColeopteraChrysomelidae

Uhmann, 1930d

http://species-id.net/wiki/Cephaloleia_emdeni

Fig. 131

Cephalolia emdeni Uhmann 1930d: 151.
Cephaloleia emdeni Uhmann. Uhmann 1957b: 18 (catalog); Staines 1997b: 413 (Uhmann species list).

##### Description.

Elongate; subdepressed; shining; head, pronotum, and antennomeres 7–11 black; antennomeres 1–6, legs, and abdomen reddish-yellow; elytra yellowish-brown with elongate black macula just below humerus, a black macula on suture on apical ¼, and black apices; legs reddish-brown. Head: vertex distinctly punctate, medial sulcus absent; frons not projecting; not depressed between eyes. Antenna: as long as head and pronotum combined; slender; antennomeres 1 elongate, oval; 2 oval, shorter than 1; 3 elongate, longest; 4–10 elongate, decreasing in length; 11 2× length of 10, acutely pointed at apex; 1–5 punctate with scattered setae; 6–11 setose. Pronotum: transverse, widest medially; lateral margin arcuate, narrowly margined; anterior angle rounded, little produced; posterior angle acute; anterior margin curved anteriorly; disc subconvex; surface weakly punctate, except medial line which is impunctate, more densely punctate basally and laterally; basal impression absent; pronotal length 1.7 mm; pronotal width 2.0 mm. Scutellum: pentagonal; impunctate. Elytron: lateral margin straight, smooth, narrowly margined; apex strongly rounded, smooth; sutural angle without tooth; humerus rounded, not produced; slightly constricted behind humerus; disc subconvex; strongly punctate-striate, punctures confused basally; elytral length 5.5 mm; elytral width 2.7 mm. Venter: obscured by card; strongly punctate laterally, impunctate medially; last sternite finely punctate. Leg: slender; punctate; tibia with fringe of setae on inner margin of apex. Total length: 7.5 mm.

##### Diagnosis.

This species is similar to *Cephaloleia parvula* and can be distinguished by the vertex of the head not being depressed between the eyes.

##### Distribution.

Brazil (Río de Janeiro).

##### Type material examined.

Holotype: Petropolis, Dr. Ohaus [green printed label]/ 1 1907 [green printed label]/ *Cephalolia emdeni* sp. n. [handwritten label]/ Type [red printed label] (SMTD).

#### Cephaloleia erichsonii

Taxon classificationAnimaliaColeopteraChrysomelidae

Baly, 1858

http://species-id.net/wiki/Cephaloleia_erichsonii

Fig. 132

Cephalolia erichsoni Baly 1858: 43. Gemminger and Harold 1876: 3601 (catalog); Donckier 1899: 549 (catalog); Weise 1910b: 83 (noted), 1911a: 8 (catalog), 1911b: 10 (catalog); Uhmann 1936b: 482 (key).
Cephaloleia erichsonii Baly. Baly 1885: 11 (distribution); Blackwelder 1946: 719 (catalog); Papp 1953: 16 (catalog); Uhmann 1957a: 18 (catalog), 1964b: 16 (faunal list); Strong 1977a: 163 (host plants); Wilcox 1983: 137 (catalog); Staines 1996: 31 (Central America species), 2004: 312 (host plants), 2011: 49 (faunal list); Staines and Staines 1999: 524 (Baly species list); McKenna and Farrell 2005: 119 (phylogeny), 2006: 10949 (phylogeny); Meskins et al. 2008: 163 (host plants), 2011: 483 (food web); Descampe et al. 2008: 227 (host plants); García–Robledo and Horvitz 2009: 116 (host plants); García–Robledo et al. 2010: 64 (noted), 2013a: 3 (biology).

##### Description.

Elongate; subparallel; subconvex; reddish-brown; eyes dark; antennomeres 1 or 1–2 yellow, 3–11 darker. Head: vertex impunctate, medial sulcus absent; frons not projecting; not depressed between eyes. Antenna: reaches to humerus; robust; male with antennomeres 1–4 or 1–5 compressed; 1 clavate, almost twice length of 2, subtriangular; 2–3 nearly equal, subtriangular; in female 3 longer than 2; 4–10 transverse, subequal in length, each shorter than 3; 11 2× length of 10, pointed at apex; 1–6 punctate with scattered setae; 7–11 setose. Pronotum: subquadrate; lateral margin slightly rounded, somewhat dilated, broadly canaliculate; anterior angle obtuse, slightly produced; posterior angle angulate; anterior margin emarginate behind head; disc subconvex; surface very finely and sparsely punctate; disc surface impunctate; basal impression absent; pronotal length 1.4–1.7 mm; pronotal width 1.9–2.6 mm. Scutellum: broadly triangular, impunctate. Elytron: lateral margin straight, smooth, margined; apex obtusely rounded; sutural angle without tooth; humerus rounded, not produced; slightly constricted behind humerus; slightly convex; disc flattened; indistinct longitudinal sulcus at humeral callus; puncture rows moderately impressed, rows faint and confused at apex; elytral length 5.0–6.0 mm; elytral width 2.4–3.1 mm. Venter: prosternum impunctate; meso- and metasterna impunctate medially, punctate laterally; abdominal sterna punctate, each puncture with pale seta; suture between sterna 1 and 2 obsolete medially; last sternite with apical margin truncate, slightly sinuate medially in male, broadly but slightly sinuate in female. Leg: short; robust; femur sparsely punctate; tibia spatulate, setose at apex. Total length: 6.7–8.0 mm.

##### Diagnosis.

This species is similar to *Cephaloleia calathae* sp. n. and *Cephaloleia conforma* sp. n. It can be distinguished by the sulcus on the humerus callus.

##### Host plant.

Adults have been collected on *Calathea gymnocarpa* H. Kennedy (Staines 1996); *Cephaloleia inocephala* (Kuntze) H. Kenn. and Nicolson, *Cephaloleia leucostachys* Hook. (Strong 1977a) (Marantaceae); *Heliconia* sp. (Heliconiaceae) (McKenna and Farrell 2005); *Cephaloleia insignis* Hort. and Bull., *Cephaloleia latifolia* Klotzsch, *Cephaloleia lutea* Schult. (Marantaceae), *Heliconia catheta* R. R. Smith, *Heliconia latispatha* Nemth., *Heliconia mariae* Hook., *Heliconia vaginalis* Benth. (Meskins et al. 2008); *Heliconia wagneriana* Peterson (Descampe et al. 2008) (Heliconiaceae); *Cephaloleia cleistantha* Standl., *Cephaloleia crotalifera* S. Watson, *Cephaloleia lutea* Schult., *Cephaloleia marantifolia* Standl. (Marantaceae) (García–Robledo and Horvitz 2009); *Heliconia stricta* Huber.

##### Immatures.

Color when alive yellow with darker margins (Figs 51–52); color when dead yellowish-brown, somewhat darker medially, venter paler than dorsum. Body elongate-ovate, flat. Dorsum with glabrous longitudinal ridge extending from anterior to posterior margin. Pronotum with central area micropustulate, rugose-punctate along anterior and lateral margins. Mesonotum with central area micropustulate, lateral margin punctate. Metanotum with raised central area micropustulate, lateral areas rugose-punctate, without carina or sulcus. Abdominal tergites 1–6 narrowed medially, without carinae near lateral margin, rugose-punctate along lateral margin; spiracles appear as darker brown macula without darker margin; orifice as in Fig. 13. Abdominal tergites 7–10 without carina, punctate along lateral and posterior margins. Venter with surface expansions rugose-striate, micropustulate, without fringe of setae along margins. Head (Fig. 11) with surface rugose-punctate; labrum with surface alutaceous, with row of short setae along anterior margin; clypeus with dense fringe of setae at apex, with four setae on apical ½, surface punctate; mandibles tridentate; maxillary palps with two palpomeres and 12 short robust setae at apex; maxilla robust, clavate, with fringe of long setae at apex; labrum densely setose. Antenna with antennomere 1 short, robust; 2 robust, nearly subglobular; 3 elongate, cylindrical, with a few setae at apex. Pro-, meso-, and metasterna wider than long, slightly depressed medially, surface rugose-punctate. Abdominal sternites wider than long, decreasing in length and width; with one sulcus on apical ½; laterally with curved sulcus on each side; 9–10 fused, rounded at apex. Leg (Fig. 12) with femur short, robust; tibotarsus subconical, with strong claw and 7 setae at apex. Total length 9.7–10.6 mm; total width 4.7–5.1 mm.

##### Distribution.

Brazil, Colombia, Costa Rica, Panama, Peru.

##### Type material examined.

Holotype: Columbia [handwritten label]/ Baly Coll. [printed label]/ Cephalolia erichsonii Baly, Columbia [blue handwritten label] (BMNH).

##### Specimens examined.

No label data (USNM). **COLOMBIA:** Antioquia- Puerto Berrío, 5 August 1938 (USNM). **COSTA RICA:** Alajuela- San Carlos (USNM). Heredia- Transecto Braulio Carillo, N.P., 1500–2050 m, October 1989 (INBIO); F. La Selva, 3 km S Pto. Viejo, 21 July 1992 (USNM); La Selva Biol. Sta., 2 km S. Pt. Viejo, 3–5 June 1984 (EGRC), 07 July 2001 (USNM); Sarapiquí, La Virgen, P. N. Braulio Carrillo, Estación Magsaysay, 100–200 m (INBIO). Limón- Finca Castilla, 30 April 1940 (MUCR); Guápiles, 30 October 1942 (MUCR); Hamburg Farm, Reventazón, Ebene Limón, 1 February 1932 (MUCR), 16 January 1936 (USNM); Est Hitoy Cerere, 100 m, R. Cerere Res. Biol. Hitoy Cerere, 30 June- 20 July 1992 (INBIO); Valle Estrella, 100–200 m (INBIO); Est Miramar, R. B. Hitoy Cerere, 300–400 m (INBIO); Pococí, Estación Cuatro Esquinas (INBIO); Talamanca, Amubri, 0–100 m (INBIO); R. V. S. Gandoca Manzanillo, 0–100 m (INBIO). Puntarenas- Barranca site, 1 km N Puntarenas, 11 September 1969 (USNM); Las Alturas, 1400 m, 22–26 May 1992 (CDFA, USNM); Est Sirena, 100 m, P. N. Corcovado, March 1990, June 1991, July 1991 (INBIO); Estación Altamira, 1 km S Cerro Biolley, 1400–1500 m (INBIO); El Carmen, 400–500 m (INBio); P. N. Carara, Garabito, Tarcoles, Estación Quebrada Bonita; Estación Esquinas, Peninsula de Osa, 0–100 m (INBIO); Finca Isaac Murillo, 0–100 m (INBIO); Sirena, Corcovado Nat Pk, 0–100 m (INBIO); Alrededor del Río Corcovado, 0–100 m (INBIO); Reserva Ftal Golfo Dulce, Est Agujas, 200–300 m (INBIO); Pque Nal Corcovado, Est Agujas, Cerro Rincón, 600–700 m (INBIO); Sierpe, Ranco Quemado, 200–300 m (INBIO). San José- San José (USNM). **PANAMA:** Bocas del Toro- 6 km N Punta Peña, 3 June 1994 (CDFA). Canal Zone- no further data (USNM). Colón- Achiote Road, 10 km SW Gatun, 12 June 1976, 12 June 1977 (EGRC); Fort Sherman, 8 May 1999 (USNM, EMEC); Gamboa, 22 July 1975 (EGRC); 5 mi NW Gamboa, 27-??-74 (EGRC); Gatun (USNM); Pipeline Road, 12 May 1978 (USNM), 23 June 1984 (EGRC); Porto Bello, 2 March 1911, 3 March 1911, 13 March 1911 (USNM); Skunk Hollow nr. Ft. Sherman, 28 May 1980 (EGRC); Skunk Hollow, nr. Fort Sherman, 28 May 1980 (EMEC). Panamá- Barro Colo. Isl., April–May 1939, 15–27 May 1972 (USNM); Bueno Vista Pt., nr. B.C.I., May 1972 (USNM); 6 km N Capira (Cerro Campana), 8 April 1981 (SEMC); Trinidad Río, 4 June 1912 (USNM). **PERU:** Loreto- Yahuarango, 11 February 1910 (USNM). Madre de Dios- CICRA Field Station, 272 m, 9 June 2011, 11 June 2011, 12 June 2011, 13 June 2011 (SEMC); Río Tamnopata Res., 30 km SW Puerto Maldonado, 290 m, 1–26 November 1982 (CASC), 22 October 1983 (USNM). Ucayali- Tingo Maria (USNM). Total: 222.

#### Cephaloleia erugatus

Taxon classificationAnimaliaColeopteraChrysomelidae

Staines, 1996

http://species-id.net/wiki/Cephaloleia_erugatus

Fig. 133

Cephaloleia erugatus Staines 1996: 31.

##### Description.

Elongate; subparallel; flattened; head (except yellow frons) and scutellum black; pronotum yellowish with medial black macula on anterior and basal margins connected by thin black vitta; elytra with yellowish vitta from base to apical ¼ including puncture rows 6–8, lateral margin black; pro-, meso-, and metasterna yellow medially, black at sides; abdominal sterna 1–4 black with yellow margins; 5 totally black; femur yellow with dark apex, tibia darker. Head: vertex impunctate, medial sulcus absent; frons not projecting; not depressed between eyes. Antenna: slender; antennomere 1 incrassate, elongate, 4× length of 2; 2 transverse, short; 3 2× length of 2, with projection on inner apical angle; 4–6 elongate, subequal in length, each shorter than 3 (rest missing). Pronotum: transverse; lateral margin straight then slightly rounding to anterior angle, margined; anterior angle slightly rounded, not projecting; posterior angle angular; anterior margin straight; disc slightly convex; surface with scattered punctures laterally; disc impunctate; basal impression absent; pronotal length 1.1 mm; pronotal width 1.4 mm. Scutellum: broadly triangular; impunctate. Elytron: lateral margin straight, smooth, margined; apex rounded; sutural angle without tooth; humerus rounded, not produced; slightly constricted behind humerus; puncture rows little impressed, obsolete on apex; elytral length 4.6 mm; elytral width 2.0 mm. Venter: pro-, meso-, and metasterna impunctate; abdominal sterna punctate, each puncture with pale seta; suture between sterna 1 and 2 complete; male with last sternite with apical margin truncate. Leg: slender; impunctate; tibia with fringe of setae on inner margin of apex. Total length: 6.1 mm.

##### Diagnosis.

This species is similar to *Cephaloleia belti*, *Cephaloleia consanguinea*, *Cephaloleia semivittata*, *Cephaloleia triangularis*, *Cephaloleia variabilis*, and *Cephaloleia vicina*. It can be distinguished by the elytral puncture rows being obsolete apically, by antennomere 1 being 3 times longer than 3, by the pronotum being punctate laterally, and by the pro-, meso-, and metasterna being impunctate.

##### Distribution.

Panama.

##### Type material examined.

Holotype male: Panamá, Isthmus Matachin, O. Thieme, S [green label]/ Coll. Thieme/ 94423/ Am Mango-Walde Herz blätterrollen [green label]/ luctuosa Panama ! [green label]/ sp. 3 male nicht luctuosa Chap. E. Uhmann Det. 34/ Zool. Mus. Berlin/ Holotype Cephaloleia erugatus Staines, Det. C. L. Staines 1993 [red label] (ZMHB).

#### Cephaloleia eumorpha

Taxon classificationAnimaliaColeopteraChrysomelidae

Staines, 1996

http://species-id.net/wiki/Cephaloleia_eumorpha

Fig. 134

Cephaloleia eumorpha Staines 1996: 32. Staines 2004: 312 (host plants).

##### Description.

Elongate; subparallel; subconvex; metallic green; elytra with creamy white vitta from suture to puncture row 3 narrowing on apical 1/5 to suture, and laterally behind humerus; venter with pro-, meso-, and metasterna dark laterally, abdominal sterna 1–4 dark with pale sides, 5 pale; leg with base and apex of femur, apex of tibia and tarsi darker. Head: vertex densely punctate, medial carina present; frons not projecting; slightly depressed between eyes. Antenna: reaches to humerus; slender; antennomere 1 elongate, subequal in length to 3; 2 transverse, ½ length of 1; 3–4 elongate, 4 shorter than 3; 5–10 transverse, subequal in length, each shorter than 4; 11 slightly longer than 10, pointed at apex; 1–2 punctate with scattered setae; 3–11 setose. Pronotum: transverse; lateral margin straight then rounding to anterior angle, margined; anterior angle rounded, not produced; posterior angle acute; anterior margin straight; disc slightly convex; surface irregularly punctate; behind head and medial area of pronotum impunctate; medial longitudinal carina present; basal impression absent; pronotal length 1.1–1.3 mm; pronotal width 1.3–1.4 mm. Scutellum: elongate triangular; alutaceous. Elytron: lateral margin straight, smooth, margined; apex rounded; sutural angle without tooth; humerus rounded, not produced; slightly constricted behind humerus; puncture rows moderately impressed; rows converge and unite at apex; elytral length 4.1–4.7 mm; elytral width 1.9–2.1 mm. Venter: pro-, meso-, and metasterna impunctate medially, punctate laterally; abdominal sterna punctate, each puncture with pale seta; suture between sterna 1 and 2 complete. Leg: slender; impunctate; tibia with fringe of setae on inner margin of apex. Total length: 5.6–6.4 mm.

**Figures 134–142.**
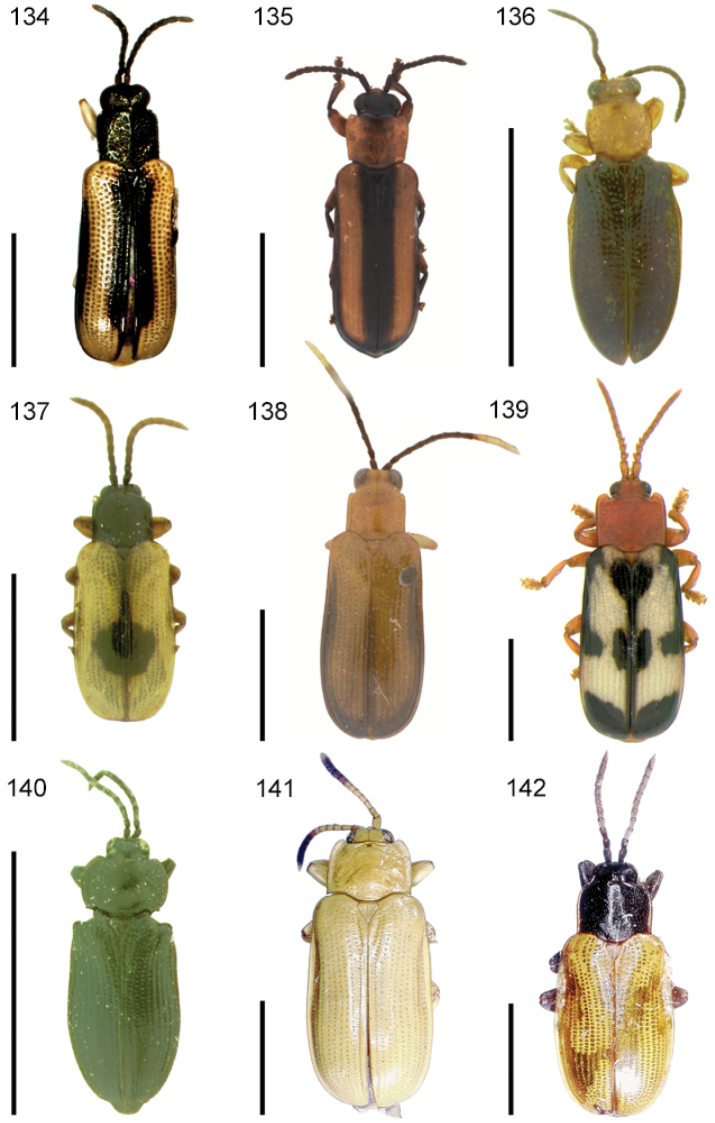
Habitus. **134** *Cephaloleia eumorpha* **135** *Cephaloleia eximia* **136** *Cephaloleia facetus* **137** *Cephaloleia fasciata* **138** *Cephaloleia felix* **139** *Cephaloleia fenestrata* **140** *Cephaloleia fiebrigi* **141** *Cephaloleia flava* **142** *Cephaloleia flavipennis*. Scale bars equal 3 mm.

##### Diagnosis.

This species is similar to *Cephaloleia interrupta* sp. n. and *Cephaloleia postuma*. It can be distinguished by antennomere 1 being longer than 2 and subequal in length to 3 and the metallic green color.

##### Host plant.

*Heliconia* sp. (Heliconiaceae) (Staines 1996).

##### Distribution.

Panama.

##### Type material examined.

Holotype: Panama, Chiriqui Prov., Reserva Fortuna, Continental Divide Trail, V-26–1993, F. Andrews and A. Gilbert/ collected in Heliconia sp. whorls/ Holotype Cephaloleia eumorpha Staines, Des. C. L. Staines 1994 [red label] (CASC).

##### Specimens examined.

**PANAMA:** Chiriquí- Reserva Fortuna, Continental Divide Trail, 25 May 1993, 26 May 1993 (AJGC, CDFA, USNM, EGRC), 1 June 1994 (CDFA, USNM). Panamá: Cerro Azul, Los Altos, 24 May 1994 (AJGC); Nusagandi area, Cordillera Igar, 19 May 1993 (EGRC). Total: 19.

#### Cephaloleia eximia

Taxon classificationAnimaliaColeopteraChrysomelidae

Baly, 1858

http://species-id.net/wiki/Cephaloleia_eximia

Fig. 135

Cephaloleia eximia Dejean 1836: 390 Nomen Nudum.
Cephalolia eximia Baly 1858: 53. Gemminger and Harold 1876: 3601 (catalog); Donckier 1899: 549 (catalog); Weise 1911a: 8 (catalog), 1911b: 12 (catalog).
Cephaloleia eximia Baly. Maulik 1916: 569 (museum list); Uhmann 1957b: 19 (catalog); Balazuc 1988: 397 (pathogens); Remillet 1988: 148 (host plant); Staines and Staines 1999: 524 (Baly species list).

##### Description.

Elongate; subparallel; subdepressed; shining; black; pronotum yellowish with large apical macula and smaller basal macula; elytra with narrow marginal line just below middle and longitudinal vitta on disc yellow; venter black; legs yellowish, tarsi and tibio-femoral joint black. Head: vertex finely punctate, medial carina present; frons not projecting; slightly depressed between eyes. Antenna: reaches beyond humerus; robust; antennomere 1 incrassate, longer than 2; 2 cylindrical, shorter than 1; 3 cylindrical, elongate, as long as 1 and 2 combined; 4–5 transverse, subequal in length, each shorter than 3; 6–10 transverse, subequal in length, each shorter than 5; 11 2× length of 10, rounded at apex; 1–7 punctate with scattered setae; 8–11 setose. Pronotum: subquadrate; lateral margin straight, narrowing then rounding to anterior angle, canaliculate; anterior angle obtuse, slightly produced; posterior angle acute; anterior margin weakly emarginate behind head; disc subconvex; surface irregularly punctate; basal impression absent; pronotal width 0.9–1.1 mm; pronotal width 0.9–1.1 mm. Scutellum: pentagonal; impunctate. Elytron: lateral margin straight, smooth, narrowly margined; apex rounded, smooth; sutural angle without tooth; humerus rounded, not produced; slightly constricted behind humerus; moderately punctate-striate; interspaces slightly elevated laterally; elytral length 3.0–3.5 mm; elytral width 1.1–1.4 mm. Venter: pro-, meso-, and metasterna impunctate medially, punctate laterally; abdominal sterna punctate, each puncture with pale seta; suture between sterna 1 and 2 complete; last sternite with apical margin broadly emarginate medially in male, truncate in female. Leg: slender; punctate; femur with row of setae on inner margin; tibia with fringe of setae on inner margin of apex. Total length: 4.6–6.0 mm.

##### Diagnosis.

This species is similar to *Cephaloleia pulchella* and *Cephaloleia saundersii*. It can be distinguished by the suture between abdominal sterna 1 and 2 being complete.

##### Host plant.

Adults have been collected feeding on *Heliconia psittacorum* Sw. (Heliconiaceae) (Remillet 1988).

##### Distribution.

French Guiana, Amazonas.

##### Type material examined.

Holotype: Cayenne [handwritten label]/ Baly coll. [printed label]/ Cephalolia eximia Baly, Cayenne [blue handwritten label] (BMNH).

##### Specimens examined.

?: Amazonas, 1880 (USNM). **FRENCH GUIANA:** vic. Saül, 8–14 830 ft., August 2012 (AJGC); vic. Saül airport, 748 ft., 8–14 August 2012 (AJGC); Rte. 6 along Montages de Kaw Trail at 1 km N Camp Patawa, 7 June 1996 (AMNH); Roura, 8.4 km SSE, 200 m, 28 May 1997, 31 May 1997 (SEMC); Saül, 7 km N Les Eaux Claires, 31 May 1997 (USNM); 3.5 mi. N Saül, Les Eaux Claires, 14 June 1996 (AMNH); Hwy D6 to Kaw, 34 km SE of Roura, 5–6 June 1986 (EGRC); 23 km SE Roura, 1000 ft., 15–20 August 2012 (AJGC). Total: 57.

#### Cephaloleia facetus

Taxon classificationAnimaliaColeopteraChrysomelidae

Staines, 1996

http://species-id.net/wiki/Cephaloleia_facetus

Fig. 136

Cephaloleia facetus Staines 1996: 33.

##### Description.

Small; elongate; flattened; head, pronotum, venter, and legs reddish-brown; elytra, antennae, and eyes dark. Head: vertex sparsely punctate, with faint medial carina; frons not projecting; not depressed between eyes. Antenna: reaches to humerus; slender; antennomeres 1–2 short, cylindrical, subequal in length; 3 elongate, cylindrical, as long as 1 and 2 combined; 4–10 transverse, subequal in length, each longer than 2; 11 2× length of 10, rounded at apex; 1–2 punctate with scattered setae; 3–11 setose. Pronotum: subquadrate; lateral margin curved, margined; anterior angle rounded, not produced; posterior angle acute; anterior margin straight; basal margin broadly, shallowly emarginate laterally; disc flattened, impunctate with faint longitudinal medial sulcus; surface punctate laterally; basal impression absent; pronotal length 0.7 mm; pronotal width 0.7–0.9 mm. Scutellum: pentagonal; impunctate. Elytron: lateral margin sinuate, smooth, margined; apex rounded; sutural angle without tooth; humerus angulate, slightly produced; slightly constricted behind humerus; basal margin produced, which fits into pronotal notch; shallowly punctate-striate, rows converge and unite at apex; elytral length 2.4–2.7 mm; elytral width 1.1–1.4 mm. Venter: pro-, meso-, and metasterna impunctate medially, punctate laterally; abdominal sterna sparsely punctate, each puncture with seta; suture between sterna 1 and 2 complete. Leg: slender; sparsely punctate; tibia with fringe of setae on inner margin of apex. Total length: 3.3–3.7 mm.

##### Diagnosis.

This species is similar to *Cephaloleia amblys*. It can be distinguished by the pronotum being paler than the elytra and by antennomeres 1 and 2 being subequal in length.

##### Host plant.

Adults have been collected on unidentified palm (Areaceae) (Staines 1996).

##### Distribution.

Panama.

##### Type material examined.

Holotype: in folded palm leaf/ Porto Bello, Pan, Mar 11, 11, E A Schwarz/ spec. degreasing.., washed in chloroform and remounted Oct. 1912/ greasing again! washed 2 hrs. in chloroform and remrd. July 10, 1916/ Holotype Cephaloleia facetus Staines, Des. C. L. Staines 1994 [red label] (USNM).

##### Specimens examined.

**PANAMA:** Colón- Porto Bello, 11 March 1911 (USNM). Total: 2.

#### Cephaloleia fasciata

Taxon classificationAnimaliaColeopteraChrysomelidae

Weise, 1904b

http://species-id.net/wiki/Cephaloleia_fasciata

Fig. 137

Cephalolia fasciata Weise 1904b: 438. Weise 1910: 91 (noted), 1911a: 8 (catalog), 1911b: 12 (catalog); Uhmann 1936b: 116 (noted); Bryant 1942: 205 (faunal list).
Cephaloleia fasciata Weise. Uhmann 1957b: 19 (catalog).

##### Description.

Subelongate; subconvex; shining; head, pronotum, and venter black; basal antennomeres reddish; palps, legs, and abdomen reddish-yellow; elytra yellowish with suture, humeral macula, and transverse band black. Head: vertex finely punctate, medial sulcus absent; small triangular projection present between antennal bases; frons not projecting; not depressed between eyes. Antenna: reaches beyond humerus; slender; antennomere 1 cylindrical, elongate; 2 transverse, ½ length of 1; 3 longer than 2, with triangular projection in male; 4 cylindrical, elongate, slightly longer than 3; 5–10 cylindrical, subequal in length, each shorter than 4; 11 subequal in length to 1, pointed at apex; 1–2 sparsely punctate; 3–11 setose. Pronotum: subquadrate; lateral margin straight then rounding to anterior angle, canaliculate; anterior angle rounded; posterior angle acute; anterior margin weakly emarginate behind head; disc subconvex; surface densely punctate, medial longitudinal line impunctate; transverse basal impression present; pronotal length 1.0–1.2 mm; pronotal width 1.1–1.3 mm. Scutellum: pentagonal; impunctate. Elytron: lateral margin straight, smooth, narrowly margined; apex rounded; sutural angle with small tooth; humerus rounded, not produced; slightly constricted behind humerus; strongly punctate-striate, rows converge and unite apically; elytral length 2.9–3.1 mm; elytral width 1.6–1.8 mm. Venter: pro-, meso-, and metasterna punctate; abdominal sterna punctate, each puncture with white seta; suture between sterna 1 and 2 complete; last sternite with apical margin emarginate medially in male, sinuate in female. Leg: slender; punctate, each puncture with pale seta; tibia with fringe of setae on inner margin of apex. Total length: 4.2–4.5 mm.

##### Diagnosis.

This species is similar to *Cephaloleia ornata*. It can be distinguished by the punctate vertex of the head which is not depressed between the eyes. Distribution. Colombia, Venezuela.

##### Distribution.

Colombia, Venezuela.

##### Type material.

Type: Puerto Cabello, Sievers (ZMUH, not seen).

##### Specimens examined.

**VENEZUELA:** ?- Puerto Cabello (ZMHB). Aragua- PN Pittier, Rancho Grande, Portochuelo, 1120 m, 22 June 1984, 21 July 1990, 12 July 1998 (USNM). Total: 5.

#### Cephaloleia felix

Taxon classificationAnimaliaColeopteraChrysomelidae

Waterhouse, 1881

http://species-id.net/wiki/Cephaloleia_felix

Fig. 138

Cephaloleia felix Waterhouse 1881: 263. Uhmann 1957b: 19 (catalog).
Cephalolia felix Waterhouse. Donckier 1899: 549 (catalog); Weise 1911a: 8 (catalog), 1911b: 12 (catalog).

##### Description.

Elongate; subdepressed; yellowish-red; antennae black with antennomeres 9–11 yellow; elytra with suture, lateral margin, and apical margin black; eyes dark. Head: vertex with small depression, medial sulcus present, impunctate; frons not projecting; not depressed between eyes. Antenna: reaches to humerus; slender; antennomere cylindrical, elongate, 1 longer than 2; 2 cylindrical, shorter than 1; 3 cylindrical, elongate, 2× length of 2; 4–10 cylindrical, elongate, decreasing in length; 11 2× length of 10, rounded at apex; 1–8 punctate with setae; 9–11 setose. Pronotum: transverse; lateral margin straight, slightly converging then rounding to anterior angle, narrowly canaliculate; anterior angle obtuse; posterior angle acute; anterior margin emarginate behind head; disc subconvex; surface strongly, moderately punctate, disc with impunctate longitudinal medial line; slight impression on present each side of basal margin; pronotal length 1.2–1.4 mm; pronotal width 1.2–1.4 mm. Scutellum: broadly triangular; impunctate. Elytron: lateral margin slightly expanding apically, smooth, slightly laminate; apex rounded, smooth; sutural angle without tooth; humerus slightly angulate, produced; slightly constricted behind humerus; moderately punctate-striate; elytral length 4.4–4.8 mm; elytral width 1.9–2.1 mm. Venter: pro-, meso-, and metasterna impunctate medially, punctate laterally; abdominal sterna punctate, each puncture with pale seta; suture between sterna 1 and 2 complete; last sternite with apical margin broadly emarginate medially in male, rounded, entire in female. Leg: slender; punctate, each puncture with reddish seta; tibia with fringe of setae on inner margin of apex. Total length: 6.0–6.4 mm.

##### Diagnosis.

This species is similar to *Cephaloleia whitei*. It can be distinguished by the straight lateral margin of the pronotum and the smaller size.

##### Distribution.

Ecuador, Peru.

##### Type material examined.

Holotype: Type H. T. [White disk with red border]/ Ecuador, Sarayacu [handwritten label]/ Buckley [handwritten label]/ Cephaloleia felix C. H. Waterh., (Type) [handwritten label] (BMNH).

##### Specimens examined.

**Ecuador:** Orellana- Estación Cientifica Yasuni, 215 m, 5–10 September 1999 (EGRC). **Peru:** Loreto- 1.5 km N Teniente Lopez, 210–240 m, 20 July 1993 (SEMC). Total: 3.

#### Cephaloleia fenestrata

Taxon classificationAnimaliaColeopteraChrysomelidae

Weise, 1910

http://species-id.net/wiki/Cephaloleia_fenestrata

Fig. 139

Cephalolia fenestrata Weise 1910: 86. Weise 1911a: 8 (catalog), 1911b: 10 (catalog).
Cephaloleia fenestrata Weise. Blackwelder 1946: 719 (catalog); Papp 1953: 16 (catalog); Uhmann 1957a: 19 (catalog); Wilcox 1983: 137 (catalog); Staines 1996: 33 (Central America species), 2004: 312 (host plants), 2011: 49 (faunal list); Johnson 2004a: 353 (biology), 2004b: 2037 (biology), 2005: 3088 (biology); Johnson and Horvitz 2005: 1185 (biology); McKenna and Farrell 2005: 119 (phylogeny), 2006: 10949 (phylogeny); Chaboo 2007: 44 (noted); García–Robledo and Horvitz 2009: 116 (host plants); García–Robledo et al. 2010: 64 (noted), 2013a: 3 (biology).
Cephalolia quadrimaculata Uhmann 1930a: 220 (Holotype: Costa Rica, F. Nevermann, 20-V-24 [green label]/ Hamburg Farm, Reventazon, Ebene Limon [reversed green label]/ Holotype [red label]/ *Cephalolia quadrimaculata* sp. n. [female]/ Cotype No. 54639 USNM [orange label], USNM).
Cephaloleia quadrimaculata Uhmann. Blackwelder 1946: 719 (catalog); Papp 1953: 21 (catalog); Uhmann 1957a: 24 (catalog); Gaedike and Döbler 1971: 358 (types); Wilcox 1983: 137 (catalog); Staines 1996: 33 (synonymy), 1997: 414 (Uhmann species list); Staines and Staines 1997: 18 (types).
Cephaloleia sp. Strong 1977a: 163. Staines 2004: 312 (identification).

##### Description.

Elongate; subparallel; subdepressed; head, pronotum, scutellum, legs, and antennomeres 1–2 red; antennomeres 3–11 red or black; elytra with black sutural vitta from puncture rows 1 to 4 and lateral vitta which narrows from base to apex and six reddish maculae. Head: vertex very finely punctate, medial sulcus present; frons with white setae, not projecting; not depressed between eyes. Antenna: reaches to humerus; slender; antennomere 1 elongate, as long as 2–4 combined; 2–4 transverse, short, 3–4 with projection on inner margin; 5–10 elongate, subequal in length, each shorter than 4; 11 2× length of 10, pointed at apex; 1–2 punctate with scattered setae; 3–11 setose. Pronotum: transverse; lateral margin straight then rounding to anterior angle, margined; anterior angle rounded, produced; posterior angle oblique; anterior margin emarginate behind head; disc flattened; surface sparsely punctate laterally, impunctate medially; deep sulcus along lateral margin; basal impression absent; pronotal length 1.3–1.4 mm; pronotal width 1.9–2.0 mm. Scutellum: triangular; pointed at apex; alutaceous. Elytron: lateral margin straight, smooth, broadly margined; apex rounded; sutural angle without tooth; humerus rounded, not produced; slightly constricted behind humerus; disc flat; moderately punctate-striate; declivity beginning just behind humerus along puncture row 7 with weak carina; rows confused near apex; elytral length 4.6–5.6 mm; elytral width 2.0–2.6 mm. Venter: pro-, meso-, and metasterna impunctate; abdominal sterna punctate, each puncture with pale seta; suture between sterna 1 and 2 obsolete medially; last sternite with apical margin weakly emarginate medially in male, truncate in female. Leg: large; impunctate; femur, especially profemur, robust; tibia with fringe of setae on inner margin of apex. Total length: 6.4–8.0mm.

##### Diagnosis.

This species is similar to *Cephaloleia histrionica* and *Cephaloleia reventazonica*. It can be distinguished by the suture between abdominal sterna 1 and 2 being obsolete medially.

##### Host plant.

Adults have been collected on *Ischnosiphon* sp. (Staines 1996); *Ischnosiphon cerotus* Leos. (Strong 1977a), *Pleiostachya pruinosa* (W. Bull. ex. Regel) K. Schum. (Johnson 2004a) (Marantaceae).

##### Immatures.

Eggs are yellow, oval, about 2 mm in length. Pupa is yellowish with two maroon series of dots in lateral rows on the dorsum (Johnson 2004a).

##### Biology.

Adults feed primarily in young rolled-leaves while larvae feed in the concavity of leaf petioles. Eggs are laid singly, in pairs, or in clusters of up to eight in leaf petioles, are covered with a frass-like material, and hatch in 7–13 days. Larval development requires 94 days and there are two larval instars. Pupae are formed in leaf petioles and the pupal period is 30 days. Adults live an average of six weeks and disperse immediately after eclosion; males live longer than females. One generation is completed in six months (Johnson 2004a).

##### Distribution.

Costa Rica, Panama.

##### Type material examined.

Holotype: 28091/ Costarica [green label]/ Wagner [green label]/ fenestrata n. [green label]/ J. Weise det./ 14/ Type [red label]/ Zool. Mus. Berlin/ Cephalolia fenestrata m (ZMHB).

##### Specimens examined.

**COSTA RICA:** Cartago- ITCA at Turrialba, 13 March 1965 (BYUC); CATIE, 3 km SW Turrialba, 600 m, 29–30 May 1985 (EMEC). Guanacaste- Río Higuerón, 6 mi W Cañas, 8 February 1969, 19 June 1969 (USNM). Heredia- Est. Biol. La Selva, 50 m, 31 March 1990 (INBIO). Limón- Hamburg Farm, Reventazón, 2 May 1924 (USNM), 31 March 1990 (MUCR); Hamburg Farm, Reventazón, Ebene Limón, 2 May 1924 (DEI). Puntarenas- Barranca site, 10 km N Puntarenas, 4 July 1969, 11 September 1969 (USNM); Corcovado Estacida S. Pedrillo, 20 March 1992 (INBIO); Golfito, 3 July 1976 (EMEC); Rancho Quemado, 200 m, Peninsula de Osa, September 1992 (INBIO); Aguirre, Quepos, P. N. Manuel Antonio, 0–100 m (INBIO); Estación Esquinas, Peninsula de Osa, 0–100 m (INBIO). **PANAMA:** Chiriquí- + 10 mi. N. Concepción, 31 June 1977 (CMNC). Total: 57.

#### Cephaloleia fiebrigi

Taxon classificationAnimaliaColeopteraChrysomelidae

Uhmann, 1936b

http://species-id.net/wiki/Cephaloleia_fiebrigi

Fig. 140

Cephalolia fiebrigi Uhmann 1936b: 115. Uhmann 1942b: 94 (noted).
Cephaloleia fiebrigi Uhmann. Uhmann 1950a: 274 (sculpture), 1957b: 19 (catalog); Gaedike and Döbler 1971: 348 (types); Staines 1997b: 413 (Uhmann species list).

##### Description.

Small; subdepressed; shining; black; lateral margin of pronotum and elytra and tarsi brownish. Head: vertex finely, densely punctate, medial sulcus absent; frons not projecting; not depressed between eyes. Antenna: as long as head and pronotum combined; slender; antennomeres 1 and 2 subequal in length; 1 cylindrical; 2 subglobular; 3 2× length of 2, cylindrical, elongate, more slender; 4 cylindrical, elongate, shorter than 3; 5–10 decreasing in length, becoming transverse; 11 2× length of 10, acutely pointed at apex; 1–5 punctate with scattered setae; 6–11 setose. Pronotum: transverse; lateral margin rounded, smooth, margined; anterior angle rounded, produced; posterior angle acute; anterior margin curved posteriorly behind head; disc subconvex; disc with fine, irregular punctures, punctures larger, denser laterally; transverse basal impression present medially; pronotal length 0.4–0.5 mm; pronotal width 1.1–1.3 mm. Scutellum: pentagonal; impunctate. Elytron: lateral margin straight, smooth, margined; apex obliquely rounded; sutural angle with small tooth; humerus rounded, not produced; slightly constricted behind humerus; finely punctate-striate, punctures obsolete at apex; elytral length 2.3–2.8 mm; elytral width 1.2–1.4 mm. Venter: prosternum rugose medially, punctate laterally; meso- and metasterna impunctate medially, punctate laterally; abdominal sterna punctate, each puncture with white seta; suture between sterna 1 and 2 complete; last sternite with apical margin emarginate in male. Leg: slender; punctate; femur and tibia with pale seta in each puncture; tibia with fringe of setae on inner margin of apex. Total length: 3.3–3.7 mm.

##### Diagnosis.

This species is similar to *Cephaloleia coroicoana*, *Cephaloleia deplanata*, *Cephaloleia marantae*, and *Cephaloleia rufipes*. It can be distinguished by the basal impression on the pronotum and by the elytral punctures being distinct basally but obsolete apically.

##### Distribution.

Argentina, Brazil (Matto Grosso), Paraguay.

##### Type material examined.

Lectotype: Paraguay, San Bernardino, 4.III, Fiebrig [printed label]/ Holotypus [red printed label]/ Cephalolia fiebrigi Uh., Det. E. Uhmann (ZMHB).

##### Specimens examined.

**Argentina:** Misiones- Hwy. 12, 28 km SW Mona Cario, J-21–1989 (BYUC). **BRAZIL:** Matto Grosso- 1886 (USNM). Total: 6.

#### Cephaloleia flava

Taxon classificationAnimaliaColeopteraChrysomelidae

Uhmann, 1930b

http://species-id.net/wiki/Cephaloleia_flava

Fig. 141

Cephalolia flava Uhmann 1930b: 136. Uhmann 1936a: 110 (noted), 1936b: 482 (key).
Cephaloleia flava Uhmann. Uhmann 1942: 97 (pygidium), 1957a: 19 (catalog); Blackwelder 1946: 719 (catalog); Papp 1953: 16 (catalog); Gaedike and Döbler 1971: 348 (types); Wilcox 1983: 137 (catalog); Staines 1996: 34 (Central America species), 1997: 413 (Uhmann species list), 2011: 49 (faunal list); Staines and Staines 1997: 8 (types); McKenna and Farrell 2005: 119 (phylogeny), 2006: 10949 (phylogeny).

##### Description.

Large; elongate; subconvex; reddish brown with eyes and antennomeres 6–11 black. Head: vertex impunctate, slight medial sulcus present; frons not projecting; slightly depressed between eyes. Antenna: reaches to humerus; robust; antennomere 1 incrassate, as long as 2–3 combined; 2 and 3 elongate, with projection on inner margins in male, cylindrical in female; 5–10 transverse, subequal in length, each shorter than 4; 11 rounded at apex, subequal in length to 1; 1–6 punctate with scattered setae; 7–11 setose. Pronotum: transverse; lateral margin straight then rounding to anterior angle, canaliculate; anterior angle rounded, produced; posterior angle acute; anterior margin emarginate behind head; disc subconvex; surface with scattered large, shallow punctures, disc virtually impunctate; basal impression absent; pronotal length 1.3 mm; pronotal width 2.3 mm. Scutellum: pentagonal; impunctate. Elytron: lateral margin straight, smooth, margined; apex rounded; sutural angle without tooth; humerus rounded, not produced; slightly constricted behind humerus; convex; punctures shallow, rows confused at apex; humeral callus virtually impunctate; declivity beginning just behind humerus along puncture row 7 with weak carina; elytral length 5.5 mm; elytral width 3.3 mm. Venter: pro-, meso-, and metasterna impunctate; abdominal sterna punctate, each puncture with pale seta; suture between sterna 1 and 2 complete; female with last sternite with apical margin bisinuate, weakly emarginate medially in male. Leg: robust; punctate; tibia curved, incised, with fringe of setae on inner margin of apex. Total length: 7.2 mm.

##### Diagnosis.

This species is similar to *Cephaloleia fulvolimbata* and *Cephaloleia gratiosa*. It can be distinguished by the impunctate disc of the pronotum, by the larger size, and by the elytral punctures being confused apically.

##### Distribution.

Costa Rica, Panama.

##### Type material examined.

Holotype male: flava Uh, male, E. Uhmann Det 30/ Holotype [red label]/ Turrialba Costa Rica, Heyne, Berlin-Wilm., V 900 m/ coll. DEI Eberswalde (DEI).

##### Specimens examined.

**COSTA RICA:** Alajuela- Est. Biol. Alberto Brenes, 29 June- 06 July 1999 (BYUC); 22 km N. San Ramon, 3000 feet, 15–25 June 2010 (AJGC). Cartago- 800 m (USNM). **PANAMA:** Panamá- Barro Colorado, winter 1924 (USNM). Total: 5.

#### Cephaloleia flavipennis

Taxon classificationAnimaliaColeopteraChrysomelidae

Baly, 1869

http://species-id.net/wiki/Cephaloleia_flavipennis

Fig. 142

Cephalolia flavipennis Baly 1869: 373 Gemminger and Harold 1876: 3601 (catalog); Donckier 1899: 549 (catalog); Weise 1911a: 8 (catalog), 1911b: 12 (catalog); Uhmann 1936b: 115 (noted), 1953d: 47 (faunal list).
Cephaloleia flavipennis Baly. Waterhouse 1881: 261 (distribution); Uhmann1957b: 19 (catalog); Staines and Staines 1999: 524 (Baly species list); McKenna and Farrell 2006: 10949 (phylogeny).

##### Description.

Elongate; large; subparallel; subconvex; head, pronotum (except castanaceous lateral margins), antennae, and legs black; scutellum and elytra yellowish. Head: vertex finely, densely punctate, medial sulcus present; frons not projecting; slightly depressed between eyes. Antenna: reaches to humerus; slender; antennomeres 1–5 elongate; 1 thickened, ⅓ longer than 2; 2–5 cylindrical; 3 longer than 2; 4–5 subequal in length, each shorter than 3; 6–10 subequal in length, transverse, each shorter than 5; 11 2× length of 10, pointed at apex; 1–2 punctate with scattered setae; 3–11 setose. Pronotum: transverse; lateral margin straight then rounding to anterior angle, canaliculate; anterior angle rounded, slightly produced; posterior angle acute; anterior margin emarginate behind head; disc subconvex; surface sparsely, strongly punctate, more dense laterally, longitudinal impunctate line on disc; basal impression absent; pronotal length 1.3–1.7 mm; pronotal width 2.0–2.4 mm. Scutellum: pentagonal; impunctate. Elytron: lateral margin straight, smooth, narrowly margined; apex rounded; sutural angle without tooth; humerus rounded, not produced; slightly constricted behind humerus; subconvex; moderately punctate-striate, punctures rather oval; interspaces flat; elytral length 5.2–5.6 mm; elytral width 2.9–3.2 mm. Venter: pro-, meso-, and metasterna impunctate medially, punctate laterally; abdominal sterna punctate, each puncture with pale seta; suture between sterna 1 and 2 obsolete medially; last sternite with apical margin truncate in female, emarginate medially in male. Leg: robust; punctate, each puncture with pale seta; tibia with fringe of setae on inner margin of apex. Total length: 7.0–7.5 mm.

##### Diagnosis.

This species is similar to *Cephaloleia collaris* and *Cephaloleia neglecta*. It can be distinguished by the lack of a transverse basal impression on the pronotum.

##### Host plant.

According to label data, adults have been collected feeding on *Heliconia standleyi* J. F. Mador. (Heliconiaceae) and *Elaeis guineensis* Jacq. (Arecaceae).

##### Distribution.

Brazil (Amazonas), Colombia, Ecuador, Peru.

##### Type material examined.

Holotype female: Ecuador [handwritten label]/ Buckley [handwritten label]/ Cephalolia flavipennis Baly, Ecuador [blue handwritten label] (BMNH).

##### Specimens examined.

**BRAZIL:** Amazonas- Manaus (USNM). **COLOMBIA:** ?- Florencia, 22 March 1972 (USNM). **Ecuador:** Napo- Aguarico, 16 August 1975 (USNM); Coca (USNM); Limonocha, 31 March 1974 (ERGC), 3 June 1977 (USNM); Limoncoha Reserve, 10 August 1997 (CDFA, USNM); Sacha Lodge, 23 March 1999 (SEMC, USNM), 21–22 March 1999 (SEMC); San Rafael Falls, 215 m, 11 August 1997 (USNM); Shushufindi, 215 m, 11–12 August 1997 (CDFA); 11 km W Plano, 500 m, 20 August 1997 (CDFA); Pununo, 20 August 1997 (CDFA); San Rafael Falls, 20 km SW El Reventador, 1100 m, 7–12 August 1997 (CDFA, USNM); 3 km S Río Molin, 19 August 1997 (CDFA); 12 km SE Tena, 24 May 1977 (USNM).; Orellana- Estacion Cientifica Yasuni, 215 m, 14 August 1997, 17 August 1997 (USNM); Yasuni, 300 m, 10–13 August 1998 (USNM); Yasuni area, 36 km S Cientifica Yasuni, 15 August 1997 (CDFA); Estacion Cientifica Yasuni, 14–17 August 1997 (CDFA); Yasuni Lodge, Río Napo, 270 m, 21 March 1999 (USNM); Yuturi Lodge, Río Napo, 270 m, 21 March 1999 (SEMC). **PERU:** Huanuca- Cueva de las Pavas Canyon, 2600 feet, 8 km S Tingo Maria, 24 April 1987 (EGRC). Loreto- Madreselva Biol. Stn., 24 June 2005 (USNM). Total: 154.

#### Cephaloleia flavovittata

Taxon classificationAnimaliaColeopteraChrysomelidae

Baly, 1858

http://species-id.net/wiki/Cephaloleia_flavovittata

Fig. 143

Cephalolia flavovittata Baly 1858: 52. Gemminger and Harold 1876: 3601 (catalog); Donckier 1899: 549 (catalog); Weise 1911a: 8 (catalog), 1911b: 11 (catalog); Bondar 1938: 17 (host plant).
Cephaloleia flavovittata Baly. Lima 1955: 202 (faunal list); Uhmann 1957b: 19 (catalog); Staines and Staines 1999: 524 (Baly species list).

##### Description.

Elongate; subparallel; flattened; black; pronotum with lateral margin yellow; elytra with lateral margin yellow and yellow vitta from humerus to middle; legs yellow. Head: vertex punctate; carina present near base of antennae; frons not projecting; slightly depressed between eyes. Antenna: nearly ½ length of body; slender; antennomeres 1 and 2 transverse, subequal in length; 3 elongate, as long as 1 and 2 combined; 4–10 transverse, decreasing in length; 11 2× length of 10, pointed at apex; 1 and 2 with scattered setae; 3–11 covered with yellow setae. Pronotum: transverse; lateral margin straight and slightly divergent for basal ¾ then rounding to anterior angle, canaliculate; anterior angle rounded, slightly produced; posterior angle acute; anterior margin emarginate behind head; disc subconvex with scattered punctures; surface impunctate medially; basal impression absent; pronotal length 1.0 mm; pronotal width 1.4 mm. Scutellum: pentagonal; alutaceous. Elytron: lateral margin straight, smooth, margined; apex rounded, smooth; sutural angle emarginate, without tooth; humerus rounded, not produced; slightly constricted behind humerus; shallowly punctate-striate, rows converge and unite apically; elytral length 3.9 mm; elytral width 2.0 mm. Venter: pro-, meso-, and metasterna impunctate medially, punctate laterally; abdominal sterna punctate, each puncture with pale seta; suture between sterna 1 and 2 complete; last sternite with apical margin broadly truncate-emarginate in female. Leg: slender; impunctate; tibia with fringe of setae on inner margin of apex. Total length: 5.0 mm.

**Figures 143–151.**
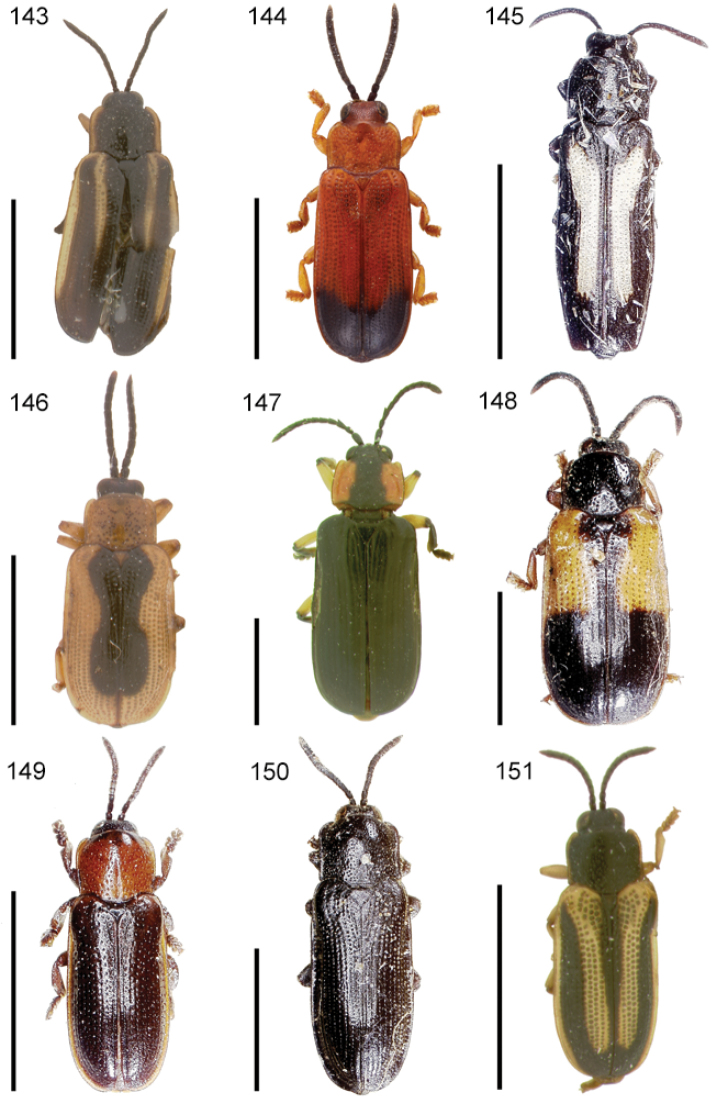
Habitus. **143** *Cephaloleia flavovittata* **144** *Cephaloleia forestieri* **145** *Cephaloleia formosus* **146** *Cephaloleia fryella* **147** *Cephaloleia fulvicollis* **148** *Cephaloleia fulvipes* **149** *Cephaloleia fulvolimbata* **150** *Cephaloleia funesta* **151** *Cephaloleia gemma* sp. n. Scale bars equal 3 mm.

##### Diagnosis.

This species is similar to *Cephaloleia deyrollei*. It can be distinguished by antennomere 1 being transverse.

##### Host plant.

*Pharus latifolius* L. (Poaceae) (Bondar 1938).

##### Distribution.

Brazil.

##### Type material examined.

Holotype: Brazil [handwritten label] Fry coll. [printed label]/ Cephalolia flavovitta Baly, Brazil [blue handwritten label] (BMNH).

#### Cephaloleia forestieri

Taxon classificationAnimaliaColeopteraChrysomelidae

Pic, 1926c

http://species-id.net/wiki/Cephaloleia_forestieri

Fig. 144

Cephalolia forestieri Pic 1926c: 13.
Cephaloleia forestieri Pic. Uhmann 1957b: 19 (catalog); Descarpentries and Villiers 1959a: 139 (types).

##### Description.

Elongate; subparallel; subdepressed; shining; reddish; eyes and antennae black; elytral apex with broad, oblique black marking. Head: vertex moderately punctate, medial sulcus present; frons not projecting; not depressed between eyes. Antenna: reaches to humerus; slender; antennomeres 1–2 elongate, thick; 2 ½ length of 1; 3 subequal in length to 1; 4–5 elongate, decreasing in length; 6–10 transverse, decreasing in length; 11 2× length 10, pointed at apex; 1–2 punctate with scattered setae; 3–11 setose. Pronotum: transverse; lateral margin straight for basal ¾ then rounding to anterior angle, canaliculate; anterior angle rounded, slightly produced; posterior angle acute; anterior margin straight; disc subconvex; surface sparsely, strongly punctate, medial longitudinal line impunctate; transverse basal impression present medially; pronotal length 0.8–1.1 mm; pronotal width 1.2–1.5 mm. Scutellum: pentagonal; impunctate. Elytron: lateral margin straight, smooth, narrowly margined; apex rounded; sutural angle without tooth; humerus rounded, not produced; slightly constricted behind humerus; finely punctate-striate, rows converge and unite apically; elytral length 3.2–3.6 mm; elytral width 1.4–1.8 mm. Venter: pro-, meso-, and metasterna impunctate medially, sparsely punctate laterally; abdominal sterna punctate, each puncture with pale seta; suture between sterna 1 and 2 complete. Leg: slender; impunctate; tibia with fringe of setae on inner margin of apex. Total length: 4.2–5.0 mm.

##### Diagnosis.

This species is similar to *Cephaloleia histrio*. It can be distinguished by antennomere 1 being subequal in length to 3.

##### Distribution.

Colombia, French Guiana.

##### Type material examined.

Holotype: Guyane Française, La Forestiére [green printed label]/ Juillet [printed label]/ Cephalolia sp. n. [handwritten label]/ var. partita Weise [handwritten label]/ forestieri sp. n. [handwritten label]/ Type [handwritten label]/ Cephaloleia forestieri Pic [printed label]/ Holotype [red printed label]/ MNHN EC 2608 [printed label] (MNHN).

##### Specimens examined.

**COLOMBIA:** Cauca- 1877 (USNM). Total: 1.

#### Cephaloleia formosus

Taxon classificationAnimaliaColeopteraChrysomelidae

Staines, 1996

http://species-id.net/wiki/Cephaloleia_formosus

Fig. 145

Cephaloleia formosus Staines 1996: 34.

##### Description.

Narrow, elongate, subparallel, flattened; black with yellow elytral vitta; leg with tarsi yellow, rest black. Head: vertex densely, deeply punctate, medial sulcus absent; frons not projecting; not depressed between eyes. Antenna: as long as head and pronotum combined; slender; antennomere 1 elongate, subequal in length to 3; 2 elongate, shorter than 1 or 3; 4–10 transverse, subequal in length; 11 2× length of 10, rounded at apex; 1–2 punctate with scattered setae; 3–11 setose. Pronotum: transverse; lateral margin evenly arcuate from base to anterior angle, serrulate, slightly canaliculate; anterior angle rounded, not produced; posterior angle acute; anterior margin sinuate behind head; disc subconvex; surface with large, shallow punctures laterally, very few punctures on disc; basal impression absent; pronotal length 0.9–1.0 mm; pronotal width 1.1–1.3 mm. Scutellum: pentagonal, alutaceous. Elytron: lateral margin straight, smooth, margined; apex truncate; sutural angle without tooth; humerus rounded, not produced; slightly constricted behind humerus; puncture rows shallow, almost obsolete laterally and apically; elytral length 3.4–3.8 mm; elytral width 1.3–1.6 mm. Venter: pro-, meso-, and metasterna punctate; abdominal sterna punctate, each puncture with pale seta; suture between sterna 1 and 2 obsolete medially; last sternite with apical margin emarginate medially in male, truncate in female. Leg: slender; femur and tibia punctate; tibia with fringe of setae on inner margin of apex. Total length: 4.4–5.3 mm.

##### Diagnosis.

This species is similar to *Cephaloleia gracilis* and *Cephaloleia vagelineata*. It can be distinguished by the serrulate lateral margins of the pronotum, by the rounded anterior angles of the pronotum, and by the suture between abdominal sterna 1 and 2 being obsolete medially.

##### Host plant.

Adults have been collected on *Elaeis guineensis* Jacq. (Areaceae) (Staines 1996).

##### Distribution.

Belize, Colombia, Panama.

##### Type material examined.

Holotype: in folded palm leaf/ Porto Bello, Pan, Mar 11, 11, E A Schwarz/ spec. degreasing.., washed in chloroform and remounted Oct. 1912/ greasing again! washed 2 hrs. in chloroform and remtd. July 10, 1916/ Holotype Cephaloleia formosus Staines, Des. C. L. Staines 1994 [red label] (USNM).

##### Specimens examined.

**BELIZE:** Belize- Mile 45 Northern Road, 13 August 1977 (EGRC). **COLOMBIA:** Antioquia- Turbo, 9 August 1971 (USNM). Total: 7.

#### Cephaloleia fryella

Taxon classificationAnimaliaColeopteraChrysomelidae

Baly, 1858

http://species-id.net/wiki/Cephaloleia_fryella

Fig. 146

Cephalolia fryella Baly 1858: 62. Gemminger and Harold 1876: 3601 (catalog); Donckier 1899: 549 (catalog); Weise 1911a: 8 (catalog), 1911b: 11 (catalog).
Cephaloleia fryella Baly. Uhmann 1957b: 19 (catalog); Staines and Staines 1999: 524 (Baly species list).

##### Description.

Oblong-elongate; subparallel; moderately convex; dull yellow, head, antennae, and scutellum black; elytra yellowish with black hourglass-shaped macula along suture from base to apical ¼; venter black, abdominal sterna yellowish laterally; legs yellow, tarsi darker. Head: vertex punctate, medial sulcus present; frons not projecting; not depressed between eyes. Antenna: reaches to humerus; slender; antennomere 1 incrassate, elongate, 2× length 2; 2–10 transverse, subequal in length; 11 2× length 10, broadly rounded at apex; 1–3 punctate with scattered setae; 4–11 setose. Pronotum: transverse; lateral margin straight then rounding to anterior angle, narrowly margined; anterior angle rounded, slightly produced; posterior angle acute; anterior margin emarginate behind head; disc subconvex; surface sparsely, coarsely punctate; basal impression absent; pronotal length 1.0 mm; pronotal width 1.3 mm. Scutellum: elongate pentagonal; impunctate. Elytron: lateral margin straight, smooth, narrowly margined; apex rounded; sutural angle without tooth; humerus rounded, not produced; slightly constricted behind humerus; slightly flattened on disc; moderately punctate-striate, rows converge and unite apically; interspaces flattened; elytral length 3.4 mm; elytral width 1.9 mm. Venter: pro-, meso-, and metasterna impunctate; abdominal sterna punctate, each puncture with pale seta; suture between sterna 1 and 2 complete; last sternite with apical margin truncate in female. Leg: slender; impunctate; tibia with fringe of setae on inner margin of apex. Total length: 4.5 mm.

##### Diagnosis.

This species is similar to *Cephaloleia lydiae*. It can be distinguished by the vertex of the head having a medial sulcus and by the straight lateral margins of the pronotum.

##### Distribution.

Brazil.

##### Type material examined.

Holotype: Brazil [handwritten label]/ Fry coll. [printed label]/ Cephalolia fryella Baly, Brazil [blue handwritten label] (BMNH).

#### Cephaloleia fulvicollis

Taxon classificationAnimaliaColeopteraChrysomelidae

Weise, 1910

http://species-id.net/wiki/Cephaloleia_fulvicollis

Fig. 147

Cephalolia fulvicollis Weise1910: 84. Weise 1911a: 8 (catalog), 1911b: 10 (catalog); Uhmann 1936a: 110 (noted), 1936b: 483 (key).
Cephaloleia fulvicollis Weise. Blackwelder 1946: 719 (catalog); Papp 1953: 17 (catalog); Uhmann 1957a: 19 (catalog); Wilcox 1983: 137 (catalog); Staines 1996: 35 (Central America species), 2004: 312 (host plants); McKenna and Farrell 2005: 119 (phylogeny).

##### Description.

Elongate; subparallel; flattened; blackish-blue with reddish yellow markings; head with frons yellowish; venter black except prosternum and legs yellow. Head: vertex punctate, with inverted V-shaped sulcus between eyes; frons punctate, not projecting; not depressed between eyes. Antenna: reaches to humerus; robust; antennomere 1 very large, as long as 2–3 combined, laterally flattened and punctate, in male, a backward projecting tooth at base and smaller tooth near apex, female with antennomere 1 not as robust and smaller basal tooth; 2 smaller, narrower than 1, inner angle produced at apex; 3 longer than but similar shape to 2; 4 shortest; 5–10 transverse, subequal in length; 11 2× length of 10, pointed at apex; 1–2 punctate with scattered setae; 3–11 setose. Pronotum: transverse; lateral margin straight and divergent for basal ⅔ then rounding to anterior angle, margined; anterior angle rounded, produced; posterior angle angulate; anterior margin emarginate behind head; disc flattened; surface almost impunctate; basal impression absent; pronotal length 1.1–1.4 mm; pronotal width 1.9–2.0 mm. Scutellum: triangular; pointed at apex; impunctate. Elytron: lateral margin straight, smooth, narrowly margined; apex rounded; sutural angle without tooth; humerus rounded, slightly produced; slightly constricted behind humerus; declivity beginning just behind humerus along puncture row 7 with weak carina; shallowly punctate-striate; elytral length 4.9–5.7 mm; elytral width 2.3–2.8 mm. Venter: prosternum impunctate; meso- and metasterna punctate; abdominal sterna punctate each puncture with pale seta; suture between sterna 1 and 2 complete; last sternite with apical margin emarginate medially in male, rounded in female. Leg: robust; punctate; profemur enlarged; tibia with fringe of setae on inner margin of apex. Total length: 6.0–7.8 mm.

##### Diagnosis.

This is a very distinctive species with the pronotum paler than the elytra and the immaculate elytra.

##### Host plant.

Adults have been collected on *Heliconia stilesii* J. W. Kress (Heliconiaceae).

##### Distribution.

Costa Rica, Mexico.

##### Type material examined.

Lectotype male: Mexico J. Flohr G. [green label]/ Type [red label]/ J. Weise det./ 94415/ fulvicollis Ws/ Zool. Mus. Berlin/ Lectotype Cephaloleia fulvicollis Weise, des. C. L. Staines 1993 [red label] (ZMHB).

##### Specimens examined.

**COSTA RICA:** Puntarenas- Estación Boscosa, Peninsula de Osa, 15 September 1991 (INBIO); Fca. Las Cruces, San Vito de Java, 11–14 August 1969 (USNM); Río Claro, 19 August 1969 (USNM); Rancho Quemado, 200 m, Peninsula de Osa, 21 March- 7 April 1992, September 1992 (INBIO); San Vito de Java, 20 July 1972 (FSCA); San Vito- Villa Neilly area, 13 August 1969 (USNM); Est. Sirena, P. N. Corcovado, 0–100 m, October 1989 (INBIO). **MEXICO:** no further data (DEI). Hidalgo- 4 mi S of Chapulhuacan, 25 May 1979 (EGRC); 4 rd. mi. SW Chapulhuacan, 4 January 1981 (EGRC); 4.1 rd. mi. SW Chapulhuacan, 4 January 1981 (EGRC); 3 mi W Hidalgo, 24 May 1979 (USNM); 3 mi W. Hild. and S. L. P. border on 85, 25 June 1979 (EGRC). Total: 162.

#### Cephaloleia fulvipes

Taxon classificationAnimaliaColeopteraChrysomelidae

Baly, 1858

http://species-id.net/wiki/Cephaloleia_fulvipes

Fig. 148

Cephalolia fulvipes Baly 1858: 49. Gemminger and Harold 1876: 3601 (catalog); Donckier 1899: 549 (catalog); Weise 1911a: 8 (catalog), 1911b: 12 (catalog).
Cephaloleia fulvipes Baly. Uhmann 1957b: 19 (catalog), 1964b: 20 (faunal list); Staines and Staines 1999: 524 (Baly species list); McKenna and Farrell 2006: 10949 (phylogeny).

##### Description.

Subelongate; subparallel; subdepressed; head, antennae, pronotum (except paler lateral margins), and scutellum shining black; elytra yellow with apical ½ black, suture darkened at base; venter black, legs dark yellowish. Head: vertex finely, sparsely punctate, medial sulcus absent; frons with longitudinal sulcus, not projecting; not depressed between eyes. Antenna: ½ body length; robust; antennomere 1 subclavate, incrassate, longer than 2; 2 cylindrical, ¾ length of 1; 3–4 cylindrical, elongate, subequal in length, longer than 2; 5–10 transverse, subequal in length, each shorter than 4; 11 2× 10, rounded at apex; 1–4 punctate with scattered setae; 5–11 setose. Pronotum: subquadrate; lateral margin straight then rounding to anterior angle, narrowly canaliculate; anterior angle obtuse, not produced; posterior angle acute; anterior margin emarginate behind head; disc subconvex; surface strongly irregularly punctate, less so on disc; transverse impression present just behind middle, obsolete medially; pronotal length 1.3–1.5 mm; pronotal width 1.6–1.8 mm. Scutellum: broadly pentagonal; impunctate. Elytron: lateral margin straight, smooth, narrowly margined; apex rounded; sutural angle without tooth; humerus rounded, not produced; slightly constricted behind humerus; flattened at suture; puncture rows near suture nearly obsolete apically, otherwise moderately punctate-striate; elytral length 4.4–4.8 mm; elytral width 2.2–2.6 mm. Venter: pro-, meso-, and metasterna punctate; abdominal sterna punctate, each puncture with pale seta; suture between sterna 1 and 2 obsolete medially; pygidium truncate-emarginate; last sternite with apical margin broadly emarginate and sinuate medially in male, truncate in female. Leg: slender; punctate; femur and tibia with pale seta in each puncture; tibia with fringe of setae on inner margin of apex. Total length: 6.0–6.4 mm.

##### Diagnosis.

This species is similar to *Cephaloleia apicalis*. It can be distinguished by the elytral punctures along the suture being obsolete near the apex.

##### Distribution.

Brazil (São Paulo), Ecuador.

##### Type material examined.

Holotype: Brazil [handwritten label]/ Fry coll. [printed label]/ Cephalolia fulvipes Baly, Brazil [blue handwritten label] (BMNH).

##### Specimens examined.

**Brazil:** Bahia- no further data (USNM). São Paulo- Cantareira, 29 October 1939 (USNM), 24 December 1939, November 1959 (USNM). **ECUADOR:** Imbabura- Cachabé, January 1897 (USNM). Total: 25.

#### Cephaloleia fulvolimbata

Taxon classificationAnimaliaColeopteraChrysomelidae

Baly, 1885

http://species-id.net/wiki/Cephaloleia_fulvolimbata

Fig. 149

Cephaloleia fulvolimbata Baly 1885: 24. Champion 1894: 234 (distribution); Blackwelder 1946: 719 (catalog); Papp 1953: 17 (catalog); Uhmann 1957a:19 (catalog); Wilcox 1983: 137 (catalog); Staines 1996: 35 (Central America species), 2011: 49 (faunal list); Staines and Staines 1997: 9 (types), 1999: 524 (Baly species list); McKenna and Farrell 2005: 119 (phylogeny), 2006: 10949 (phylogeny); Naczi and Staines 2011: 2 (faunal list).
Cephalolia fulvolimbata Baly. Weise 1911a: 8 (catalog), 1911b: 12 (catalog).

##### Description.

Small; narrow; subparallel; subdepressed; head, antennae (except antennomere 1 and apex of 11 reddish-black), scutellum, and elytra (except margins) dark; pronotum reddish with narrow black vitta laterally; venter with prosternum reddish, rest black; legs red except apical ½ of femur which is darker. Head: vertex densely punctate, medial sulcus absent; frons not projecting; depressed between eyes. Antenna: ½ body length; slender; antennomeres 1–3 elongate; 1 and 3 subequal in length; 2 slightly shorter than 1 or 3; 4–10 transverse, subequal in length; 11 longer than preceding, pointed at apex; 1–4 punctate with scattered setae; 5–11 setose. Pronotum: nearly twice as wide as long; lateral margin straight and converging from base to anterior angle, margined; anterior angle broadly rounded, not produced; posterior angle acute; anterior margin emarginate behind head; disc subconvex; surface densely punctate; basal impression absent; pronotal length 0.9 mm; pronotal width 1.1–1.3 mm. Scutellum: scarcely longer than wide; pentagonal; impunctate. Elytron: lateral margin straight, smooth, margined; apex rounded; sutural angle without tooth; humerus rounded, not produced; slightly constricted behind humerus; declivity beginning just behind humerus at puncture row 7 with faint carina; subconvex; strongly punctate-striate; puncture rows converge and unite at apex; elytral length 3.0 mm; elytral width 1.4 mm. Venter: prosternum impunctate; meso- and metasterna impunctate medially, punctate laterally; abdominal sterna punctate, each puncture with pale seta; suture between sterna 1 and 2 complete; last sternite with apical margin deeply emarginate medially in male, truncate in female. Leg: slender; punctate; tibia with rows of setae on outer margin and fringe of setae at apex. Total length: 4.0 mm.

##### Diagnosis.

This species is similar to *Cephaloleia flava* and *Cephaloleia gratiosa*. It can be distinguished by the uniformly punctate pronotum and the smaller size.

##### Distribution.

Belize, Guatemala, Honduras, México.

##### Type material examined.

Syntypes: Senahu, Vera Paz, Champion/ Paratipo [red label]/ F. Monros Collection 1959/ Cephaloleia fulvolimbata Baly, J. S. Baly det. [pink label]; Tepa, Tabasco, Feb. H.H.S./ Cephaloleia fulvolimbata (USNM, 2).

##### Specimens examined.

**BELIZE:** Toledo District- ca. 8 mi NNE Medina Bank, ca. 1 mi S Bladen Branch, 13 April 2008 (USNM). **GUATEMALA:** Alta Verapaz- 24 July 1957 (BYUC); Cacao Trece Aguas, 4 October (USNM), 5 April, 4 April, 25 March, 12 April (USNM). Baja Verapaz- 3.3 km. W. Chilascó, 1800 m, 25 May 1991 (CMNC). **HONDURAS:** ?- Progesso, 5 March 1923 (UMMZ). **MEXICO:** no further data (DEI). Chiapas- Montebello, 21 July 1969 (CNC). Tabasco- Teapa (USNM); 4 mi. N. Teapa, 14- June 1965 (TAMU). Veracruz- 3 mi. n. Huatusco, 17 July 1980 (TAMU); 4 mi SE Jalapa, 29 June 1955 (INHS); 7 mi. S. E. Orizaba, 19–20 June 1983 (FSCA); 2 mi. se. Tebanca, Lago Catemaco, 3 June 1964 (TAMU); Veracruz, 23 March 1968 (USNM). Total: 107.

#### Cephaloleia funesta

Taxon classificationAnimaliaColeopteraChrysomelidae

Baly, 1858

http://species-id.net/wiki/Cephaloleia_funesta

Fig. 150

Cephalolia funesta Baly 1858: 59. Gemminger and Harold 1876: 3601 (catalog); Donckier 1899: 549 (catalog); Weise 1905a: 132 (noted), 1906: 221 (museum list), 1911a: 8 (catalog), 1911b: 13 (catalog); Bruch 1915: 375 (faunal list), 1928: 202 (faunal list); Spaeth 1937: 144 (noted); Uhmann 1942b: 96 (noted); Bosq 1943: 40; Monrós and Viana 1947: 164 (Argentina species).
Cephaloleia funesta Baly. Uhmann 1936b: 116 (museum list), 1938b: 365 (comparative note), 1957b: 19 (catalog), 1957c: 364 (noted), 1964a: 403 (catalog); Gaedike and Döbler 1971: 349 (types); Staines and Staines 1999: 524 (Baly species list); Roig–Juñent 2004: 116 (faunal list); Staines 2004a: 312 (host plants).
Cephaloleia handschini Uhmann 1948b: 12 (Holotype male: Brazil, Rio de Janeiro, VIII.1946, coll. Wygodzinsky [printed label]/ Holotyp [red printed label]/ Cephaloleia handschini Uh., Det. E. Uhmann, DEI). Uhmann 1957b: 20 (catalog), 1957e: 364 (synonymy); Gaedike and Döbler 1971: 350 (types); Staines 1997b: 413 (Uhmann species list).

##### Description.

Elongate; subparallel; subdepressed; shining black, basal palpomeres lighter. Head: vertex sparsely punctate, medial sulcus present; interantennal keel present; frons not projecting; depressed between eyes. Antenna: as long as head and pronotum combined; robust; antennomere 1 subclavate, longer than 2; 2 cylindrical; 3 cylindrical, elongate, longest, nearly 2× length of 2; 4–10 transverse, decreasing in length; 11 2× length of 10, broadly rounded at apex; 1–4 or 5 punctate with scattered setae; 5 or 6–11 setose. Pronotum: subquadrate; lateral margin straight then rounding to anterior angle, slightly canaliculate; anterior angle obtuse, slightly produced; posterior angle acute; anterior margin emarginate behind head; disc subconvex; surface densely punctate, less so on disc; basal impression absent; pronotal length 1.4–1.6 mm; pronotal width 1.6–1.8 mm. Scutellum: broadly pentagonal; impunctate. Elytron: lateral margin straight, smooth, margined; apex rounded; sutural angle weakly emarginate, with minute tooth; humerus rounded, not produced; slightly constricted behind humerus; subconvex, slightly flattened on disc; punctate-striate, punctures moderately impressed basally, larger and coarser laterally, finer apically; declivity beginning just behind humerus at puncture row 7 edged with faint carina; elytral length 4.5–4.8 mm; elytral width 2.0–2.4 mm. Venter: pro-, meso-, and metasterna punctate; abdominal sterna punctate, each puncture with pale seta; suture between sterna 1 and 2 complete; last sternite with apical margin broadly truncate-emarginate in female. Leg: slender, punctate; femur and tibia with seta in each puncture; tibia with fringe of setae on inner margin of apex. Total length: 5.7–6.1 mm.

##### Diagnosis.

This species is similar to *Cephaloleia impressa* and *Cephaloleia obsoleta*. It can be distinguished by the vertex of the head without a medial sulcus, by the elytra with a declivity beginning at puncture row 7, and by antennomere 1 being longer than 3.

##### Host plant.

*Canna* sp. (Cannaceae), *Maranta divaricata* Rosc. (Marantaceae), *Pharus glaber* Kunth. (Poaceae), *Cordyline* sp. (Agavaceae) (Monrós and Viana 1947).

##### Distribution.

Argentina, Bolivia, Brazil (Rio de Janeiro, Santa Catharina), Paraguay.

##### Type material examined.

Holotype female: Brazil [handwritten label]/ Fry Coll. [printed label]/ Cephalolia funesta Baly, Brazil [blue handwritten label] (BMNH).

##### Specimens examined.

**Argentina:** Chaco- Resistenica, October-December 1936 (USNM). Corrrientes- 42 km E. Ituzanigo, 23 January 1989 (BYUC). Misiones- no further data (USNM); 1931 (USNM); Dos de Mayo, November 1989 (USNM); Eldorado, 6 November 1992 (USNM); Puerto Igazú, 16 November 1989 (USNM); 19 km S. Wanda, 22 January 1989 (BYUC). Tucuman- Quchada de Jules, 10 August 1952 (USNM). **Brazil:** no further data (DEI). Rio de Janeiro- no further data (DEI); Muri, 4 February 1952 (USNM). Santa Catharina- Nova Teutonia, September 1967, October 1968 (USNM), September 1976, October 1976, November 1976, December 1976, January 1977, February 1977 (EGRC). Total: 39.

#### Cephaloleia gemma

Taxon classificationAnimaliaColeopteraChrysomelidae

Staines sp. n.

http://zoobank.org/016C4CBB-8625-4974-AA7A-6462A1E6F2E3

http://species-id.net/wiki/Cephaloleia_gemma

Fig. 151

##### Description.

Elongate; small; subparallel; subconvex; black, lateral margin of pronotum paler; elytra with broad pale yellow vitta from puncture rows 2–6 from base extending to humerus to near apex, lateral margin pale; venter dark brown, abdominal sterna paler laterally; legs yellow. Head: eyes large; vertex coarsely, irregularly punctate, medial sulcus absent; frons not projecting; not depressed between eyes. Antenna: as long as head and pronotum combined; robust; antennomere 1 incrassate, longer than 2; 2 subglobose; 3 cylindrical, subequal in length to 1; 4–10 transverse, subequal in length, each about ¾ length of 3; 11 2× length of 10, pointed at apex; 1–2 punctate with scattered setae; 3 punctate basally, setose apically; 4–11 setose. Pronotum: transverse; lateral margin straight then rounding to anterior angle, canaliculate; anterior angle obtuse, produced; posterior angle acute; anterior margin emarginate behind head; disc subconvex; surface irregularly punctate except medial longitudinal area; basal impression absent; pronotal length 0.7 mm; pronotal width 1.0 mm. Scutellum: pentagonal; impunctate. Elytron: lateral margin straight then rounding to apex, smooth; apex rounded; sutural angle without tooth; humerus rounded, not produced; slightly constricted behind humerus; moderately punctate-striate, punctures mostly large; elytral length 3.1 mm; elytral width 1.4 mm. Venter: pro- and mesosterna punctate; metasternum impunctate medially, punctate laterally; abdominal sterna punctate, each puncture with pale seta; suture between abdominal sterna 1 and 2 obsolete medially; apical margin of last sternite with apical margin emarginate medially in male. Leg: slender; femur and tibia punctate; tibia with fringe of setae on inner margin of apex. Total length: 4.0 mm.

##### Etymology.

From gemma (Latin) bud, eye, jewel for the large eyes and beautiful appearance of this species. The name is feminine.

##### Diagnosis.

This species is similar to *Cephaloleia abdita* sp. n. and *Cephaloleia trilineata*. It can be distinguished by the smaller size and the overall black dorsal coloration.

##### Host plant.

Adults have been collected off *Calathea* sp. (Marantaceae).

##### Distribution.

Bolivia, Brazil (Matto Grosso, Rondonia).

##### Type material.

Holotype male: Brazil, Rondonia, 62 km SW Ariquames, Fazfa. Rancho Grande, nr. Cacaulandia, Nov. 3, 1989, R. W. Flowers/ Holotype *Cephaloleia gemma* Staines, des. C. L. Staines 2012 [red label] (USNM). Paratypes (14): Brazil, Mato Gr., 10°25'S, 59°28'W, 17–22.III.77, 300 m, Col: D. Engleman/ Edward G. Riley Collection/ Paratype *Cephaloleia gemma* Staines, des. C. L. Staines 2012 [red label] (EGRC); Bolivia, Santa Cruz dpt., Nuflo de Chavez, 28 xi-5 xii 2011, Concepeción- FCBC, Alta Vista, 16°08.15"S, 61°56.1"W, 425 m, MAR. Calathea sp. (green+violet leaf), L. Sekerka and D. Windsor lgt. (LSC, DWC).

#### Cephaloleia gilvipes

Taxon classificationAnimaliaColeopteraChrysomelidae

Uhmann, 1930a

http://species-id.net/wiki/Cephaloleia_gilvipes

Fig. 152

Cephalolia gilvipes Uhmann 1930a: 230.
Cephaloleia gilvipes Uhmann. Blackwelder 1946: 719 (catalog); Papp 1953: 17 (catalog); Uhmann 1957a: 19 (catalog); Wilcox 1983: 137 (catalog); Staines 1996: 36 (Central America species), 1997: 413 (Uhmann species list); Staines and Staines 1997: 9 (types); Flowers and Hanson 2003: 51 (distribution); McKenna and Farrell 2005: 119 (phylogeny).

##### Description.

Elongate-oval; convex; small; shining; metallic blue; legs, antennae, and mouthparts yellow. Head: vertex impunctate, medial sulcus absent; frons not projecting; depressed between eyes. Antenna: reaches to humerus; slender; antennomeres 1–2 transverse, 2 longer than 1; 3 elongate, cylindrical, as long as 1 and 2 combined; 4–10 elongate, subequal in length, each shorter than 3; 11 2× length of 10, rounded at apex; 1–3 punctate with scattered setae; 4–11 setose. Pronotum: transverse but much narrower than base of elytra; lateral margin sinuate, finely margined; anterior angle with short blunt tooth not produced; posterior angle acute; anterior margin straight; disc convex; surface sparsely irregularly punctate; transverse prebasal depression behind disc and longitudinal depression laterally; pronotal length 0.8 mm; pronotal width 1.2 mm. Scutellum: pentagonal; micropunctate. Elytron: lateral margin weakly expanding apically, smooth, fringed with setae; apex rounded, with very small teeth; sutural angle without tooth; humerus rounded, not produced; slightly constricted behind humerus; punctate-striate, puncture rows obsolete on apex and humerus; elytral length 3.3 mm; elytral width 2.0 mm. Venter: pro-, meso-, and metasterna impunctate medially, alutaceous laterally; abdominal sterna punctate, each puncture with pale seta; suture between sterna 1 and 2 complete. Leg: slender; punctate, each puncture with pale seta; tibia with fringe of setae on inner margin of apex. Total length: 4.5 mm.

**Figures 152–160.**
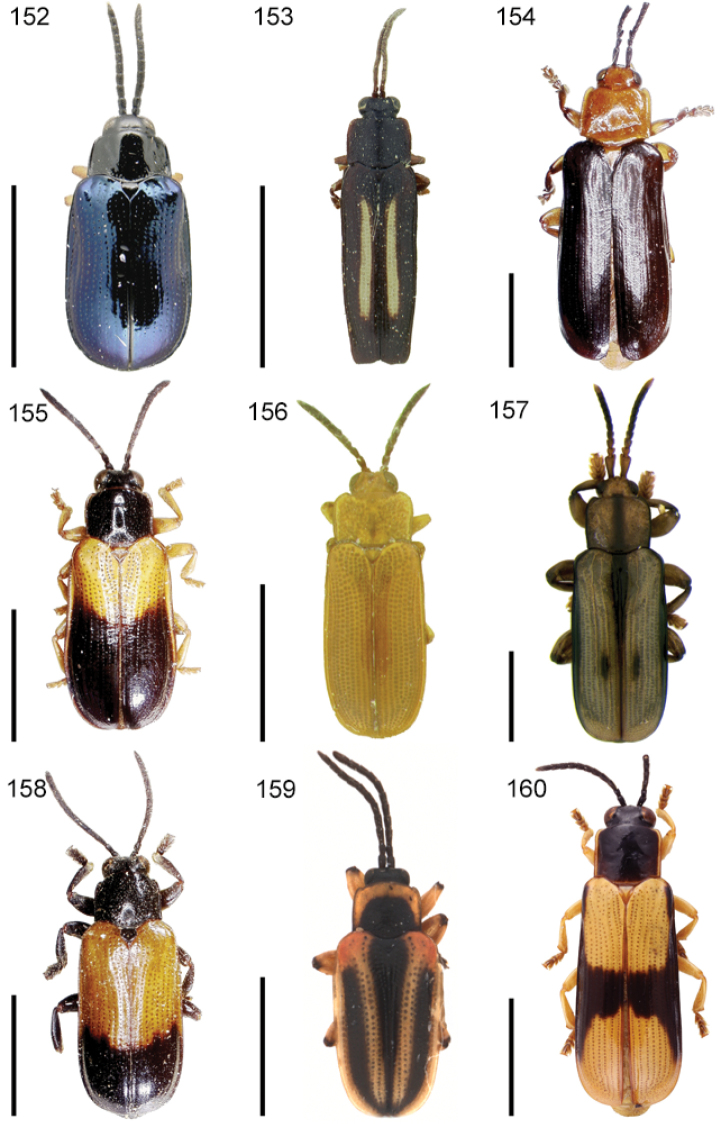
Habitus. **152** *Cephaloleia gilvipes* **153** *Cephaloleia gracilis* **154** *Cephaloleia gratiosa* **155** *Cephaloleia grayei* **156** *Cephaloleia halli* **157** *Cephaloleia heliconiae* **158** *Cephaloleia histrio* **159** *Cephaloleia histrionica* **160** *Cephaloleia hnigrum*. Scale bars equal 3 mm.

##### Diagnosis.

This species is similar to *Cephaloleia punctatissima*. It can be distinguished by the metallic blue coloration and by the elytral puncture rows being obsolete apically.

##### Distribution.

Costa Rica.

##### Type material examined.

Holotype: Costa Rica, F. Nevermann, 4-I-24 [green label]/ Sta. Maria de Dota, auf Blüten [reversed green label]/ Type No. 54603 USNM [orange label]/ Holotype [red label]/ *Cephalolia gilvipes* sp. n. (USNM).

##### Specimens examined.

**COSTA RICA:** Cartago- El Guarco, San Isidro, 4 km S Cañon, 2200–2300 m (INBIO); La Chonta, 27 km SE Cartago, 2250 m, 5 February 1965 (BYUC). Limón- Valle del Silencio, 2400–2500 m (INBIO); Valle del Silencio, Vallecito, Los Sphagnum, 2400–2500 m (INBIOo); Valle del Silencio, Send. Jardín Natural, 2400–2500 m (INBIO). Puntarenas- Sta. Elena Cloud Forest Res., 1700 m, 9 September 1998 (BYUC). Total: 6.

#### Cephaloleia gracilis

Taxon classificationAnimaliaColeopteraChrysomelidae

Baly, 1878

http://species-id.net/wiki/Cephaloleia_gracilis

Fig. 153

Cephaloleia gracilis Baly1878: 41. Donckier 1899: 549 (catalog); Weise 1911a: 8 (catalog), 1911b: 13 (catalog).
Cephaloleia gracilis Baly. Uhmann 1957b: 20 (catalog); Staines and Staines 1999: 524 (Baly species list).

##### Description.

Small; slender; elongate; subdepressed; dark chestnut brown; elytra with ivory white vitta from humeri to apical ¼ between puncture rows 2–5; basal 5 antennomeres reddish-black, rest black; legs reddish. Head: vertex deeply punctate, with medial carina; frons not projecting; slightly depressed between eyes. Antenna: reaches beyond humerus; slender; antennomere 1 subincrassate, 2× length of 2; 2 subglobose; 3 cylindrical, elongate, subequal in length to 2; 4 cylindrical, elongate, 1½ length of 3; 5–10 elongate, decreasing in length; 11 longer than 10, rounded at apex; 1–3 punctate; 4–11 setose. Pronotum: quadrate; lateral margin straight then rounding to anterior angle, margined; anterior angle rounded; posterior angle acute; anterior margin straight; basal margin biangulate; disc moderately convex; surface moderately densely punctate; basal impression absent; pronotal length 0.9 mm; pronotal width 1.0 mm. Scutellum: pentagonal; impunctate. Elytron: lateral margin straight, smooth, converging toward apex; apex truncate; sutural angle with small tooth; humerus slightly angulate; moderately punctate-striate; elytral length 3.3 mm; elytral width 1.2 mm. [Venter and legs obscured by card]. Total length: 4.5 mm.

##### Diagnosis.

This species is similar to *Cephaloleia formosus* and *Cephaloleia vagelineata*. It can be distinguished by the smooth lateral margins of the pronotum.

##### Distribution.

Amazonas.

##### Type material examined.

Holotype: Amazons [handwritten label]/ Cephaloleia gracilis Baly, Amazons [blue handwritten label] (BMNH).

#### Cephaloleia gratiosa

Taxon classificationAnimaliaColeopteraChrysomelidae

Baly, 1858

http://species-id.net/wiki/Cephaloleia_gratiosa

Fig. 154

Cephalolia gratiosa Baly 1858: 40. Gemminger and Harold 1876: 3601 (catalog); Donckier 1899: 549 (catalog); Weise 1905a: 130 (noted), 1911a: 8 (catalog), 1911b: 10 (catalog); Uhmann 1932b: 261 (museum list), 1936b: 483 (key), 1942: 94 (noted).
Cephaloleia gratiosa Baly. Baly 1858: 165 (noted), 1885: 8 (distribution); Blackwelder 1946: 719 (catalog); Papp 1953: 17 (catalog); Uhmann 1957a: 20 (catalog); Wilcox 1983: 137 (catalog); Staines 1996: 36 (Central America species); Staines and Staines 1999: 524 (Baly species list); McKenna and Farrell 2005: 119 (phylogeny), 2006: 10949 (phylogeny); Frank and Morón 2012: 8 (host plants).
Cephaloleia gratiosa nigripennis Weise 1905a: 130 (type: México, ZMHB?, not seen). Uhmann 1936b: 483 (key).
Cephaloleia unicolor Weise 1905a: 130 (type: México, ZMHB?, not seen).
Cephaloleia gratiosa unicolor Weise. Uhmann 1936b: 483 (key).
Cephalolia beckeri Weise 1905a: 131 (type: México, Amatan, ZMHB?, not seen). Weise 1911a: 7 (catalog), 1911b: 10 (catalog).
Cephaloleia beckeri Weise. Blackwelder 1946: 718 (catalog); Papp 1953: 14 (catalog); Uhmann 1957a: 16 (catalog); Wilcox 1983: 136 (catalog); Staines 1996: 36 (synonymy).

##### Description.

Large; subparallel; subdepressed, flattened; variable in color- reddish-brown with black eyes and black central maculae on apical ½ of elytra; or black head, antennae, and elytra, and reddish brown pronotum; or totally reddish brown; antennae black, sometimes with antennomeres 1–3 yellow; venter with prosternum yellow, meso- and metasterna yellow medially and black laterally, abdominal sterna black with yellow side margin; legs with femur yellow with black apex, tibiae black at base and apex. Head: vertex sparsely punctate, medial carina present; frons not projecting; depressed between eyes. Antenna: reaches beyond humerus; slender; antennomeres 1–5 compressed; 1 clavate, incrassate, elongate, very compressed; 2 and 4 elongate, subequal in length, dilated interiorly at apex, 3 longer than 2 or 4; 4 subtriangular; 5–10 transverse, subequal in length, each shorter than 4; 11 rounded at apex, elongate, ¾ length of 1; 1–3 punctate with scattered setae; 4–11 setose. Pronotum: transverse; lateral margin straight for basal ¾ then rounding to anterior angle, margined; anterior angle rounded, slightly produced; posterior angle angulate; anterior margin emarginate behind head; disc subconvex; surface finely, sparsely punctate; disc almost impunctate; basal impression absent; pronotal length 1.6–2.1 mm; pronotal width 1.9–2.7 mm. Scutellum: triangular; shining; impunctate. Elytron: lateral margin straight, smooth, margined; apex rounded; sutural angle without tooth; humerus rounded, not produced; slightly constricted behind humerus; declivity beginning just behind humerus along puncture row 7 edged with faint carina; subconvex, disc flattened; moderately punctate-striate; puncture rows converge and unite at apex; elytral length 6.7–8.0 mm; elytral width 3.4–3.7 mm. Venter: prosternum impunctate; meso- and metasterna impunctate medially, punctate laterally; abdominal sterna punctate, each puncture with pale seta; suture between sterna 1 and 2 complete; last sternite with apical margin slightly sinuate in male, rounded in female. Leg: robust; tibia with setae on inner apical ⅓ and fringe of setae at apex; protibial basal marking extends along outer edge almost to apex. Total length: 9.1–10.3 mm.

##### Diagnosis.

This species is similar to *Cephaloleia flava* and *Cephaloleia fulvolimbata*. It can be distinguished by the impunctate pronotal disc, by the larger size, and by the elytral puncture rows being regular apically.

##### Host plant.

Adults have been collected off flowers of *Heliconia bourgaeana* Peterson (Heliconiaceae) (Frank and Morón 2012).

##### Distribution.

Costa Rica, Mexico, Panama.

##### Type material.

Type: México, Sallé, Baly coll., Saunders coll. (BMNH, not seen).

##### Specimens examined.

**COSTA RICA:** Alajuela- Río San Lorencito, 900 m, Res. For. San Ramón, 5 km N Col. Palmarena, March 1990, 13–18 June 1993 (INBIO); Upala Fluss, 17–21 August 1988 (MUCR). Guanacaste- Río San Lorenzo, 1050 m, Tierras Morenas, Z. P. Tenorio, April 1991, June 1991, 10–20 February 1992, 28 March- 21 April 1992, April 1992, July 1992, October 1992, December 1992, February 1993, April 1993 (INBIO). Heredia- Est. Magasasay, 200 m, P. N. Braulio Carillo, May 1992 (INBIO). Limón- Amubri, 700 m, Talamanca, 1–22 July 1992, 16–31 August 1992 (INBIO); Est. Cuatro Esquinas, 0 m, P.N. Tortuguero, 27 March- 29 April 1992, January 1993 (INBIO); Est. Hitoy Cerere, 100 m, R. Cerere, Res. Biol. Hitoy Cerere, September 1992, November 1992, 15–27 February 1993, April 1993 (INBIO); Manzanillo, 0–100 m, RNFS Gandoca y Manzanillo, 7–19 August 1992, 9 September- 13 October 1992, 24 September- 13 October 1992, 22 October- 11 November 1992, 4–12 December 1992, 15–13 December 1992, 6–27 January 1993 (INBIO); Est. Miramar, 500 m, Res. Biol. Hitory Cerere, September 1992 (INBIO). Puntarenas- Est. Queb. Bonita, 50 m, Res. Biol. Carara, May 1992, June 1992 (INBIO). **MEXICO:** no further data (DEI, ZMHB). Chiapas- (DEI). **PANAMA:** Bocas del Toro- 2.3 rd m. N from Continental Divide, 20 May 1993 (EGRC). Coclé- rd N. Cerro Gaital, 15 May 1980 (EGRC). Panamá- Nusagandi area, I. K. U. S. A. Igar, 20 May 1993 (EGRC). Total: 287.

#### Cephaloleia grayei

Taxon classificationAnimaliaColeopteraChrysomelidae

Baly, 1858

http://species-id.net/wiki/Cephaloleia_grayei

Fig. 155

Cephalolia grayei Baly 1858: 51. Gemminger and Harold 1876: 3601 (catalog); Donckier 1899: 549 (catalog); Weise 1911a: 8 (catalog), 1911b: 12 (catalog), 1921a: 174 (faunal list); Uhmann 1936b: 116 (noted), 1953d: 47 (faunal list).
Cephaloleia grayei Baly. Waterhouse 1881: 262 (faunal list); Uhmann 1957b: 20 (catalog); Staines and Staines 1999: 524 (Baly species list).

##### Description.

Elongate; subparallel; subdepressed; shining; head, pronotum, scutellum, and apical ½ of elytra black; elytra with basal portion yellowish; venter yellowish. Head: vertex punctate, medial carina present; frons not projecting; depressed between eyes. Antenna: ½ body length; slender; antennomere 1 clavate, slightly incrassate, elongate, longer than 2; 2 thicker than 3, ¾ length of 3; 3–5 subequal in length, each longer than 2; 6–10 transverse, subequal in length, each shorter than 5; 11 longer than 10, pointed at apex; 1–2 punctate with scattered setae; 3–11 setose. Pronotum: subquadrate; lateral margin sinuate basally then rounding to anterior angle, slightly canaliculate; anterior angle obtuse, not produced; posterior angle acute; anterior margin nearly straight; disc subconvex; surface sparsely punctate, punctures more dense basally; transverse basal impression present medially; pronotal length 1.3–1.5 mm; pronotal width 1.5–1.7 mm. Scutellum: broadly pentagonal; impunctate. Elytron: lateral margin straight, smooth, narrowly margined; apex rounded, smooth; sutural angle emarginate, without tooth; humerus rounded, not produced; slightly constricted behind humerus; moderately punctate-striate, rows converge and unite apically; elytral length 4.5–4.8 mm; elytral width 2.1–2.4 mm. Venter: prosternum punctate; meso- and metasterna impunctate medially, punctate laterally; abdominal sterna punctate, each puncture with pale seta; suture between sterna 1 and 2 complete; last sternite with apical margin emarginate medially in male, sinuate laterally in female. Leg: slender; punctate; tibia with fringe of setae on inner margin of apex. Total length: 6.0–6.5 mm.

##### Diagnosis.

This species is similar to *Cephaloleia apicalis* and *Cephaloleia fulvipes*. It can be distinguished by the pronotum having a transverse basal impression.

##### Distribution.

Bolivia, Brazil (Bahia, Minas Gerais, Rondonia), Ecuador, Peru.

##### Type material examined.

Holotype: Peru [handwritten label]/ Baly coll. [printed label]/ Cephalolia grayei Baly, Peru [blue handwritten label] (BMNH).

##### Specimens examined.

**BOLIVIA:** Santa Cruz- Buena Vista, March 1951 (USNM). **Brazil:** ?- Capada (USNM); Juanfue (USNM). Bahia- São Paulo d’Olivenca (USNM). Minas Gerais- nr. Timoleo, 15 January 1989 (BYUC). Rondonia- 62 km SW Ariquames, Fzda Rancho Grande, 15 November 1994 (BYUC). **ecuador:** Napo- Sacha Lodge, 270 m, 4–14 March 1994, 23 March 1994, 13–25 July 1994 (SEMC). Total: 11.

#### Cephaloleia halli

Taxon classificationAnimaliaColeopteraChrysomelidae

Uhmann, 1951a

http://species-id.net/wiki/Cephaloleia_halli

Fig. 156

Cephaloleia halli Uhmann 1951a: 69. Uhmann 1957b: 20 (catalog); Gaedike and Döbler 1971: 350 (types); Staines 1997b: 413 (Uhmann species list).

##### Description.

Elongate; subparallel; subconvex; shining; reddish-brown; eyes and apical antennomeres darker. Head: vertex impunctate, medial sulcus becomes carina between antennal bases; frons impunctate, not projecting; depressed between eyes. Antenna: as long as head and pronotum combined; slender; antennomere 1 slightly incrassate, longer than 2; 2–3 cylindrical, elongate, subequal in length to 3; 4–10 transverse, subequal in length; 11 longer than 10, rounded at apex; 1–3 punctate with scattered setae; 4–11 setose. Pronotum: quadrate; lateral margin straight for basal 4/5 then angulate to anterior angle, canaliculate; anterior angle angulate, produced; posterior angle acute; anterior margin emarginate behind head; disc subconvex, nearly impunctate; surface sparsely punctate; basal impression absent; pronotal length 1.1–1.3 mm; pronotal width 1.2–1.4 mm. Scutellum: pentagonal, impunctate. Elytron: lateral and apical margins smooth; lateral margin straight, margined; apex rounded; sutural angle with minute tooth; humerus rounded, not produced; slightly constricted behind humerus; moderately punctate-striate, rows confused apically; elytral length 3.5–3.9 mm; elytral width 1.4–1.8 mm. Venter: pro-, meso-, and metasterna impunctate medially, punctate laterally; abdominal sterna punctate, each puncture with pale seta; suture between sterna 1 and 2 obsolete medially; last sternite with apical margin truncate in female, emarginate in male. Leg: slender; sparsely punctate; tibia with fringe of setae on inner margin of apex. Total length: 5.0–5.4 mm.

##### Diagnosis.

This species is similar to *Cephaloleia apicicornis*, *Cephaloleia corallina*, *Cephaloleia ochra* sp. n., and *Cephaloleia proxima*. It can be distinguished by the pronotum lacking a transverse basal impression, by the paler basal antennomeres, by the meso- and metaserna being punctate laterally, by the vertex of the head being depressed between the eyes, by antennomere 2 being longer than 1, and by the anterior angle of the pronotum being angulate.

##### Distribution.

Brazil (Bahia, Parana, Rondonia), Ecuador.

##### Type material examined.

Holotype female: Ecuador, Cachabé, low c., xi.1896, Rosenberg [printed label]/ 70 248 [handwritten label]/ Holotypus [red printed label]/ Cephaloleia halli Uh., Det. E. Uhmann (BMNH).

##### Specimens examined.

**BRAZIL:** Bahia- São Paulo d’Olivenca, May 1883 (USNM). Parana- Parque N Fdz. Do Iguacu, 9 October 1968 (BYUC). Rondonia- 62 km SW Ariquames, Fzda. Rancho Grande, 6 October 1993 (BYUC). **Ecuador:** Imbabura- Cachabé, November 1896 (DEI, USNM). Total: 8.

#### Cephaloleia heliconiae

Taxon classificationAnimaliaColeopteraChrysomelidae

Uhmann, 1930a

http://species-id.net/wiki/Cephaloleia_heliconiae

Fig. 157

Cephalolia heliconiae Uhmann 1930a: 217. Uhmann 1936b: 483 (key).
Cephaloleia heliconiae Uhmann. Blackwelder 1946: 719 (catalog); Uhmann 1950b: 336 (type), 1957a: 20 (catalog); Papp 1953: 17 (catalog); Gaedike and Döbler 1971: 350 (types); Wilcox 1983: 137 (catalog); Staines 1996: 37 (Central America species), 1997: 413 (Uhmann species list), 2004: 312 (host plants), 2011: 49 (faunal list); Staines and Staines 1997: 10 (types); McKenna and Farrell 2005: 119 (phylogeny), 2006: 10949 (phylogeny); García–Robledo et al. 2013a: 3 (biology).

##### Description.

Elongate; subparallel; large, subdepressed, yellowish-brown; eyes dark; antennomeres 1, 2, 11 yellowish, 3–10 dark; scutellum black; elytra with black sutural and lateral vittae and two black maculae after middle; pronotum sometimes with black longitudinal medial vitta. Head: vertex impunctate, with fine medial carina; keel present between antennae; frons projecting; not depressed between eyes. Antenna: reaches to humerus; slender; antennomere 1 incrassate, clavate, elongate, as long as 2–4 combined; 2–4 transverse, compressed with interior projection, 2–3 subequal in length, 4 shorter; 5–10 transverse, subequal in length, each shorter than 4; 11 2× length of 10, rounded at apex; 1–3 punctate with scattered setae; 4–11 setose. Pronotum: transverse but much narrower than base of elytra; lateral margin straight for basal ¾ then rounding to anterior angle, margined; anterior angle rounded, slightly produced; posterior angle acute; anterior margin emarginate behind head; disc flattened; surface with scattered, fine punctures; basal impression absent; pronotal length 1.7–1.9 mm; pronotal width 2.0 mm. Scutellum: elongate, acutely triangular; impunctate. Elytron: lateral margin straight, smooth, margined; apex rounded; suture angle without tooth; humerus rounded, not produced; slightly constricted behind humerus; declivity beginning just behind humerus along puncture row 7 with weak carina; finely punctate-striate; puncture rows converge and unite at apex, but are slightly confused; elytral length 6.0–6.4 mm; elytral width 2.6–3.0 mm. Venter: pro-, meso-, and metasterna impunctate; abdominal sterna punctate, each puncture with pale seta; suture between sterna 1 and 2 obsolete medially; last sternite with apical margin weakly rounded in male, bisinuate in female. Leg: robust; femur and tibia punctate; tibia dentate at apex, with setae on inner apical ⅓. Total length: 8.1–9.0 mm.

##### Diagnosis.

This species is similar to *Cephaloleia adusta* and *Cephaloleia championi*. It can be distinguished by the elytral declivity along puncture row 7, by the elytral punctures being distinct after the middle, and by the suture between abdominal sterna 1 and 2 being obsolete medially.

##### Host plant.

Adults have been collected in young rolled leaves of *Heliconia* sp. (Heliconiaceae), *Calathea insignis* Hort. and Bull. (Staines 1996); *Cephaloleia crotalifera* S. Watson, *Cephaloleia lutea* G. Mey. (Marantaceae) (García–Robledo et al. 2013a).

##### Distribution.

Costa Rica, Panama.

##### Type material examined.

Lectotype: Costa Rica, F. Nevermann, 15-XI-23 [green label]/ Hamburg Farm, Reventazon, Ebene Limon [reversed green label]/ in jungen jusuinen-gerollin Blätten, 1. Heliconia sp or Calathea insignis (USNM).

##### Specimens examined.

**COSTA RICA:** Alajuela- Est. Eladios, 820 m, Ref. Peñas Blancas, Res. Biol. Monteverde, July 1991 (INBIO); Río San Lorencito, 900 m, R. F. San Ramón, 5 km N de Colonia Palmareña, March 1990, 13–18 June 1993 (INBIO). Guanacaste- La Gloria, alt. 900 m, June 1931 (USNM); Río San Lorenzo, 1050 m, Tierras Morenas, Z. P. Tenorio, April 1991, 10–20 February 1992, 23 March- 21 April 1992, April 1992, July 1992, August 1992, October 1992, December 1992, February 1993, March 1993, April 1993 (INBIO). Heredia- Est. Biol. La Selva, 06 July 2001 (USNM). Limón- Sector Cerro Cocorí, Fca. de E. Rojas, 150 m, February 1993, March 1993, April 1993, May 1993 (INBIO); Hamburg Farm, Reventazón, Ebene Limón, 15 November 1923, 1 February 1932 (DEI); Río Sardinas, 10 m, R.N.F.S., Barra del Colorado, September 1992 (INBIO); Salvadora Farm, Parismina Fluss, 19–31 December 1930 (DEI); Waldeck, 22 August 1928 (USNM); Valle La Estrella, R. B. Hitoy Cerere, 100–200 m (INBIO). Puntarenas- Coto Brus, Las Cruces Biological Station, 10 March 2012 (USNM); Monteverde, Cordillera de Tilarán, 10 March 1991 (EGRC); San Luis, 1040 m, R. B. Monteverde, October 1992 (INBIO); Santa Clara- Colombiana Farm, April 1924 (USNM); Las Mercedes, 200–300 m, 15 November 1924 (DEI), July 1926, 8 May 1928 (USNM); Guacimal, Finca Buen Amigo Monteverde, 4 km S la Reserva, 1000–1100 m (INBIO); Reserva Bosque Eterno de los Niños, 1500–1600 m (INBIO). San José- Finca La Caja, 14 June 1931 (MUCR); Carillo, 5 January 1933 (USNM). **PANAMA:** Bocas del Toro- 6 km N Punta Peña, 27 May 1993 (AJGC); Reserva La Fortuna, 28 May 1993 (EGRC). Chiriquí- Repr. la Fortuna, 3200', 17–21 September 1976 (EGRC); Reserva Fortuna, Continental Divide Trail, 29 May 1993 (CDFA); Reserva Fortuna, Fortuna Dam, 29 May 1993 (CDFA). Coclé- Cerro Gaital, 2300 ft., 28 May 1994 (USNM). Total: 182.

#### Cephaloleia histrio

Taxon classificationAnimaliaColeopteraChrysomelidae

Guérin-Méneville, 1844

http://species-id.net/wiki/Cephaloleia_histrio

Fig. 158

Cephaloleia histrio Guérin–Méneville 1844: 282. Baly 1858: 51 (redescription); Uhmann 1957b: 20 (catalog), 1966d: 269 (noted); Angel 1989: 81 (museum list); McKenna and Farrell 2005: 119 (phylogeny), 2006: 10949 (phylogeny).
Cephalolia histrio Guérin-Méneville. Gemminger and Harold 1876: 3601 (catalog); Donckier 1899: 549 (catalog); Weise 1904b: 439 (noted), 1911a: 8 (catalog), 1911b: 12 (catalog); Uhmann 1936b: 116 (noted).

##### Description.

Elongate; subparallel; subdepressed; shining black; basal ⅔of elytra yellowish, apical ⅓black; scutellum and legs black; venter yellowish with dark maculae. Head: vertex strongly, densely punctate, medial sulcus present; frons not projecting; slightly depressed between eyes. Antenna: longer than head and pronotum combined; slender; antennomere 1 clavate, elongate, thick; 2–4 cylindrical, elongate, 2 ½ length 3, 4 longer than 2; 5–10 transverse, decreasing in length; 11 longer than 10, broadly rounded at apex; 1–2 punctate with scattered setae; 3–11 setose. Pronotum: transverse; lateral margin straight then rounding to anterior angle, canaliculate; anterior angle rounded, not produced; posterior angle acute; anterior margin straight; disc subconvex; surface coarsely punctate; transverse basal impression present; pronotal length 1.2–1.4 mm; pronotal length 1.4–1.6 mm. Scutellum: pentagonal; punctate. Elytron: lateral margin straight, smooth, narrowly margined; apex rounded; sutural angle without tooth; humerus rounded, not produced; slightly constricted behind humerus; slightly flattened on disc; moderately punctate-striate, punctures obsolete apically; pygidium pentagonal; elytral length 4.3–4.8 mm; elytral width 2.0–2.2 mm. Venter: pro-, meso-, and metasterna punctate; abdominal sterna punctate, each puncture with pale seta; suture between sterna 1 and 2 complete; last sternite with apical margin truncate at apex in female, weakly emarginate then sinuate medially in male. Leg: slender; punctate, each puncture with pale seta; tibia with fringe of setae on inner margin of apex. Total length: 5.6–6.8 mm.

##### Diagnosis.

This species is similar to *Cephaloleia forestieri*. It can be distinguished by antennomere 1 being longer than 3.

##### Host plant.

According to label data, adults have been collected feeding on *Heliconia* sp. (Heliconiaceae).

##### Distribution.

Bolivia, Colombia, Ecuador, Peru, Venezuela.

##### Type material.

Type: Colombia, Santa Fe de Bogata (depository unknown, not seen).

##### Specimens examined.

?- no label data (USNM); Tacopalmsleon (?) plantation (USNM). **BOLIVIA:** Cochabamba- Chapare, Villa Tunari, December 1985 (USNM); Cochabamba, 67.5 km NE Est. Biol. Valle de Sajita, Univ. de San Simon, 300 mm, 7–9 February 1999 (SEMC, USNM). **COLOMBIA:** no further data (USNM). Antioquia- Puerto Berrío, 9 August 1938, 11 August 1938 (USNM). **Ecuador:** Napo- Pununo, 20 August 1997 (CDFA). **PERU:** Madre de Dios- Tambopata Wildlife Res., 30 km SW Pto. Maldanado, 290 m, 26 November 1982, 25 December 1982 (USNM). Pasco- Oxapampa-Puzuzo Rd., 1300 m, 20 October 1999 (SEMC, USNM), 16 October 1999 (SEMC). **VENEZUELA:** no further data (USNM). Aragua- Rancho Grande, 18 October 1975 (USNM). Federal District- Caracas, May-June 1877 (USNM). Miranda- Guarenas, 420 m, 27 August 1964 (USNM). Total: 37.

#### Cephaloleia histrionica

Taxon classificationAnimaliaColeopteraChrysomelidae

Baly, 1885

http://species-id.net/wiki/Cephaloleia_histrionica

Fig. 159

Cephaloleia histrionica Baly 1885: 15. Blackwelder 1946: 719 (catalog); Papp 1953: 17 (catalog); Uhmann 1957a: 20 (catalog); Wilcox 1983: 137 (catalog); Staines 1996: 38 (Central America species), 2011: 49 (faunal list); Staines and Staines 1997: 10 (types), 1999: 524 (Baly species list); Flowers and Hanson 2003: 51 (distribution); McKenna and Farrell 2005: 119 (phylogeny), 2006: 10949 (phylogeny); García–Robledo et al. 2013b: 190 (larva); Schmitt and Frank 2013: 58 (biology).
Cephalolia histrionica Baly. Donckier 1899: 549 (catalog); Weise 1911a: 8 (catalog), 1911b: 12 (catalog).

##### Description.

Elongate; subparallel; subconvex; head, antennae, and scutellum black; pronotum yellow with large black basal trapezoidal macula from basal margin covering posterior ⅔ of disc; elytra yellowish with reddish humerus and black sutural and lateral vittae, sutural vitta widest at base, narrowed posteriorly, lateral vittae begin behind humerus then converge and narrow toward suture; venter with pro-, meso-, and metasterna reddish-brown medially, darker laterally; leg yellowish with femur and tibia with dark area at apices. Head: vertex densely punctate, medial sulcus absent; frons with adpressed yellowish setae, not projecting; not depressed between eyes. Antenna: reaches to humerus; slender; antennomeres 1 and 3 subequal in length; 2 shorter than 1 or 3; 1 elongate, clavate; 2 transverse; 3–10 elongate, cylindrical, decreasing in length; 11 2× length of 10, rounded at apex; 1–3 punctate with scattered setae; 3–11 setose. Pronotum: transverse; lateral margin straight then slightly converging to anterior angle, margined; anterior angle subacute, slightly produced; posterior angle acute; anterior margin emarginate behind head; disc flattened, irregularly punctate, rest nearly impunctate; basal impression absent; pronotal length 1.0–1.1 mm; pronotal width 1.4–1.6 mm. Scutellum: broadly triangular; impunctate. Elytron: lateral margin straight, smooth, margined; apex obtusely rounded; sutural angle without tooth; humerus angulate, slightly produced; slightly constricted behind humerus; declivity beginning just behind humerus along puncture row 7 with weak carina; moderately punctate-striate, punctures smaller on disc; puncture row 10 removed from lateral margin; rows converge and unite apically; elytral length 4.0–4.4 mm; elytral width 2.3–2.4 mm. Venter: pro-, meso-, and metasterna impunctate medially, punctate laterally; abdominal sterna punctate, each puncture with pale seta; suture between sterna 1 and 2 complete; last sternite with apical margin u-shaped in male, slightly acuminate in female. Leg: slender; coxae punctate; tibia with fringe of setae on inner margin of apex. Total length: 5.4–5.9 mm.

##### Diagnosis.

This species is similar to *Cephaloleia fenestrata* and *Cephaloleia reventazonica*. It can be distinguished by the suture between abdominal sterna 1 and 2 being complete, by the impunctate disc of the pronotum, by the elytral punctures being larger on the disc, and by puncture row 10 being removed from the lateral margin.

##### Comments.

Preliminary analysis of the CO1 gene indicates that cryptic species may be present under the current application of this species name. further work is needed to resolve this question.

##### Host plant.

*Pitcairnia arcuata* (André) André (Bromeliaceae) (García–Robledo et al. 2013b); *Calathea lutea* Schult. (Marantaceae), *Heliconia imbricata* Baker, *Heliconia latispatha* Benth. (Heliconiaceae) (Schmitt and Frank 2013); *Costus* sp (Costaceae).

##### Immatures.

Color when live white becoming translucent laterally and apically. Color when dead yellowish-brown. Pronotum without raised central area; micropustulate; with pale setae along lateral and apical margins. Mesonotum without raised central area or carina or sulcus; micropustulate; laterally with numerous shallow sulci on expansion. Metanotum with central portion micropustulate; without carina or sulcus. Abdominal tergites 1–6 slightly narrowed in middle; with carina laterally; spiracle near basal margin; each spiracle appears as spot with darker margin and surrounded by short lanceolate setae. Abdominal tergites 7–10 without surface plicae or carinae. Venter: surface if expansions sulcate near body, smooth laterally. Head with surface sparsely punctate; labrum smooth, without setae; clypeus with fringe of setae at apex; mandibles tridentate; maxillary palps with two palpomeres, with setae at apex; maxilla robust, clavate, with fringe of long setae at apex; labium densely setose. Antenna with antennomere 1 short, ½ length of 2; 2 cylindrical, longer than 1 and 3 combined; 3 shortest, with ring of setae at apex. Prosternum shorter than others, wider than long, slightly depressed in middle; surface rugose-striate. Meso- and metasterna wider than long, slightly depressed in middle, surface rugose-striate. Abdominal sternites 1–8 wider than long, decreasing in width; with transverse sulcus just beyond middle and second transverse sulcus near apex; sterna 9–10 fused, rounded at apex. Leg with femur wider and longer than tibiotarsus; tibiotarsus subconical, with robust claw and six setae at apex. Total length 4.6 mm; width 3.8 mm (n=1).

##### Distribution.

Costa Rica, Guatemala, Panama.

##### Type material examined.

Syntype: Bugaba, Panama, Champion [printed label]/ Paratipo [handwritten red label]/ F. Monros Collection 1959 [printed label]/ Cephaloleia histrionica Baly, J. S. Baly det. [handwritten pink label] (USNM, 1).

##### Specimens examined.

**COSTA RICA:** Alajuela- 8 km N Vara Blanca, Volcán Poas, 1500 m, 11 May 1985 (EMEC). Heredia- Chilamate, 24–30 July 1993 (BYUC); Rara Avis Biological Station, 25 November 2011 (USNM). Puntarenas- Coto Brus, Las Cruces Biological Station, 3 March 2012 (USNM); Golfito, July 1981 (FSCA); Monteverde Cloud For., 27–31 May 1984 (EGRC); Rancho Quemado, Peninsula de Osa, 200 m, October 1991 (INBIO); Quepos, 80 m, P. N. Manuel Antonio, April 1991 (INBIO); Osa Peninsula, 5.0 mi SW Rincón, 31 July 1968 (USNM); Pen. Osa., 31 July 1968 (MUCR); Finca Las Cruces, 6 km. S. San Vito de Java, 4200 ft., 28 September-2 October 1986 (FSCA). **GUATEMALA:** Suchitepequez, Los Tarrales Private Nature Res, 27 July 2008 (BYUC). **PANAMA:** Colón- Achiote Road 10 km SW Gatun, 12 June 1976 (EGRC). Panamá- Cerro Campana, 800 m, 2 September 1972, 20 June 1985, 17 May 1993 (EGRC), 2 June 1993 (AJGC, CDFA). Total: 27.

#### Cephaloleia hnigrum

Taxon classificationAnimaliaColeopteraChrysomelidae

Pic, 1923

http://species-id.net/wiki/Cephaloleia_hnigrum

Fig. 160

Cephalolia hnigrum Pic 1923: 8. Uhmann 1942b: 116 (noted).
Cephaloleia hnigrum Pic. Uhmann 1957b: 20 (catalog), 1961b: 23 (noted), 1964a: 403 (catalog); Descarpentries and Villiers 1959a: 139 (types).

##### Description.

Elongate; subparallel; large; shining; subdepressed; yellowish-brown; antennae and head black; pronotum black with pale lateral margins; elytra with lateral black vitta and medial black macula; venter and legs yellowish-brown. Head: vertex finely punctate, medial sulcus absent; keel present between antennal bases; frons not projecting; depressed between eyes. Antenna: longer than head and pronotum combined; slender; antennomere 1 incrassate, elongate; 2 elongate, ½ length of 1, shortest; 3–4 elongate, cylindrical, 4 longer than 3; 5–10 cylindrical, each as long as 3, elongate; 11 longer than 4, rounded at apex; 1–2 punctate with scattered setae; 3–11 setose. Pronotum: quadrate; lateral margin straight then rounding to anterior angle, canaliculate; anterior angle rounded, not produced; posterior angle acute; anterior margin straight; disc subconvex; surface sparsely irregularly punctate; transverse basal impression present medially; pronotal length 1.6–1.8 mm; pronotal width 1.6–1.8 mm. Scutellum: elongate pentagonal; impunctate. Elytron: lateral margin straight, smooth, margined; apex rounded; sutural angle without tooth; humerus rounded, not produced; slightly constricted behind humerus; declivity beginning just behind humerus along puncture row 7 with weak carina; moderately punctate-striate, rows confused apically; pygidium densely setose; elytral length 5.8–6.2 mm; elytral width 2.3–2.5 mm. Venter: pro-, meso-, and metasterna impunctate; abdominal sterna punctate, each puncture with pale seta; suture between sterna 1 and 2 complete; last sternite with apical margin emarginate in male, truncate in female. Leg: slender; impunctate; tibia with fringe of setae on inner margin of apex. Total length: 7.8–8.2 mm.

##### Diagnosis.

This species is similar to *Cephaloleia bifasciata* and *Cephaloleia recondita*. It can be distinguished by the lateral margins of the elytra having black markings which do not extend to puncture row 6 and by the vertex of the head not having a medial sulcus.

##### Distribution.

Ecuador.

##### Type material examined.

Syntypes: Cachabe, Equateur [handwritten label]/ 269 [printed label]/ Cephalolia hnigrum m, Type [handwritten label]/ Hnigrum Pic (1923) [handwritten label]/ Museum Paris Coll. M. Pic [blue printed label]/ Cephaloleia hnigrum Pic [printed label]/ Syntype [red label]/ MNHN EC 2460 [printed label]; Cachabe, Equateur [handwritten label]/ Type [printed label with red border]/ Museum Paris Coll. M. Pic [blue printed label]/ Cephaloleia hnigrum Pic [printed label]/ Syntype [red label]/ MNHN EC 2461 [printed label]; Cachabe, Equateur [handwritten label]/ Museum Paris Coll. M. Pic [blue printed label]/ Cephaloleia hnigrum Pic [printed label]/ Syntype [red label]/ MNHN EC 2462 [printed label] (MNHN, 3).

##### Specimens examined.

**Ecuador:** Imbabura- Cachabé (USNM). Total: 3.

#### Cephaloleia horvitzae

Taxon classificationAnimaliaColeopteraChrysomelidae

Staines sp. n.

http://zoobank.org/29B63AC1-BD95-46C2-8390-9865F6761100

http://species-id.net/wiki/Cephaloleia_horvitzae

Fig. 161

##### Description.

Small; elongate; subparallel; subdepressed; head and antennae yellowish-brown; pronotum pale yellow with broad black longitudinal vitta from anterior margin to basal margin; scutellum dark; elytra pale yellow with black cordate macula at base along suture, with small black macula at humerus, with black W-shaped vitta on apical ½, and black macula on apex near sutural angle; venter brownish-yellow; legs pale yellow. Head: vertex coarsely, densely punctate, medial carina present; frons punctate, not projecting; keel present between antennal bases; depressed between eyes. Antenna: reaches to humerus; robust; antennomere 1 subclavate, elongate; 2 elongate, ¾ length of 1; 3–4 elongate, subequal in length, each ¾ length of 2; 5–10 transverse, subequal in length, each ¾ length of 4; 11 2× length of 10, broadly rounded at apex; 1–2 punctate with scattered setae; 3–11 setose. Pronotum: transverse; lateral margin sinuate basally then rounding to anterior angle, narrowly margined; anterior angle rounded, slightly produced; posterior angle acute; anterior margin emarginate behind head; disc subconvex; surface sparsely punctate; basal impression absent; pronotal length 1.0 mm; pronotal width 1.2–1.4 mm. Scutellum: pentagonal; impunctate. Elytron: lateral margin straight, smooth, narrowly margined; apex rounded; sutural angle without tooth; humerus rounded, not produced; slightly constricted behind humerus; declivity beginning just behind humerus along puncture row 7 with weak carina; moderately punctate-striate, rows converge and unite apically; elytral length 3.0–3.1 mm; elytral width 1.5–1.8 mm. Venter: pro-, meso-, and metasterna punctate; abdominal sterna punctate, each puncture with pale seta; suture between sterna 1 and 2 complete; last sternite with apical margin emarginate medially in male, truncate in female. Leg: slender; punctate, each puncture with pale seta; tibia with fringe of setae on inner margin of apex. Total length: 4.2–4.7 mm.

**Figures 161–169.**
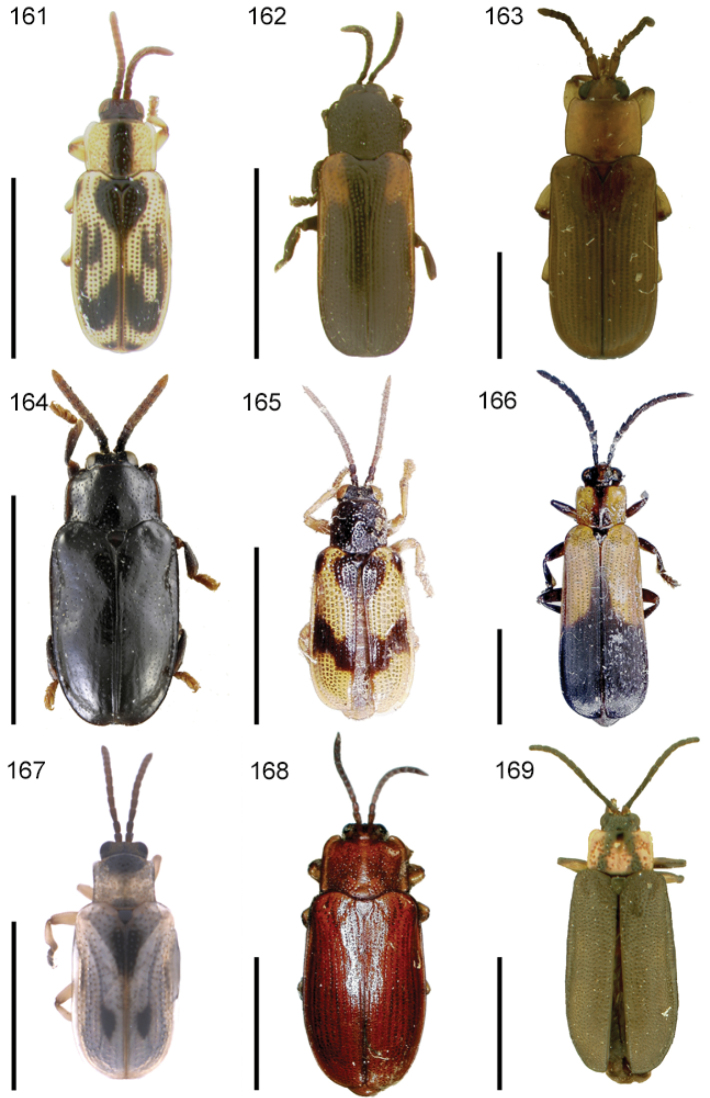
Habitus. **161** *Cephaloleia horvitzae* sp. n. **162** *Cephaloleia humeralis* **163** *Cephaloleia immaculata* **164** *Cephaloleia impressa* **165** *Cephaloleia insidiosa* **166** *Cephaloleia instabilis* **167** *Cephaloleia interrupta* sp. n. **168** *Cephaloleia interstilialis* **169** *Cephaloleia irregularis*. Scale bars equal 3 mm.

##### Etymology.

Named for Carol C. Horvitz in recognition of her many contributions to the understanding of the ecology and evolution of Zingiberales and their interactions with pollinators, seed dispersers, and insect herbivores and her collegiality over the years. Also in recognition of her mentorship to new generations of resesearchers. The name is feminine.

##### Diagnosis.

This species is similar to *Cephaloleia antennata*. It can be distinguished by the vertex of the head having a medial carina, by the pronotum lacking an oblique impression, and by the smaller size.

##### Distribution.

French Guiana.

##### Type material.

Holotype male: Fr. Guiana, Hwy N2 to Regina at Bolanger Creek, 1-VI-1986, E. G. Riley and D. A. Rider/ Holotype *Cephaloleia horvitzae* Staines, des. C. L. Staines 2012 (red label) (TAMU). Paratypes (3) (each with Paratype *Cephaloleia horvitzae* Staines, des. C. L. Staines 2012 [red label]): French Guiana, Roura, 27.4 km SSE, 280 m, 4°44'20"N, 52°13'25"W, 10 Jun 1997, J. Ashe, R. Brooks, FGIAB97177, ex flight intercept trap (SEMC); Guyana (Regina), Montagen de Kaw, PK37, 28.V.98, J. A. Cerda/ piege malaise (LSC); Guyana French, NE, S of Regina, 30 XII 2006, Snizek (LSC).

#### Cephaloleia humeralis

Taxon classificationAnimaliaColeopteraChrysomelidae

Weise, 1910

http://species-id.net/wiki/Cephaloleia_humeralis

Fig. 162

Cephalolia humeralis Weise 1910: 94. Weise 1911a: 8 (catalog), 1911b: 13 (catalog); Uhmann 1936b: 116 (noted).
Cephaloleia humeralis Weise. Uhmann 1957b: 20 (catalog).

##### Description.

Small; elongate; subparallel; slender; subconvex; black; elytra with elongate reddish humeral macula. Head: vertex smooth, faint medial sulcus present; frons not projecting; slightly depressed between eyes. Antenna: reaches to humerus; slender; antennomere 1 subglobose, short; 2 slightly elongate, obconic, longer than 1; 3 cylindrical, much longer than 2, longest; 4–10 transverse, subequal in length, each shorter than 1; 11 longer than 10, pointed at apex; 1–4 punctate with scattered setae; 5–11 setose. Pronotum: quadrate; lateral margin straight for basal ⅔ then rounding to anterior angle, narrowly margined; anterior angle rounded, not produced; posterior angle acute; anterior margin straight; disc subconvex; surface distinctly punctate with longitudinal medial impunctate line; transverse basal impression present medially; pronotal length 1.0–1.2 mm; pronotal width 1.0–1.2 mm. Scutellum: pentagonal; impunctate. Elytron: lateral margin straight, smooth, narrowly margined; apex rounded; sutural angle without tooth; humerus rounded, not produced; slightly constricted behind humerus; moderately punctate-striate; puncture rows converge and unite apically; elytral length 3.0–3.2 mm; elytral width 1.2–1.4 mm. Venter: pro-, meso-, and metasterna punctate; abdominal sterna punctate, each puncture with pale seta; suture between abdominal sterna 1 and 2 obsolete medially; last sternite with apical margin broad and slightly truncate. Leg: slender; punctate, each puncture with pale seta; tibia with two rows of setae on inner margin and fringe of setae at apex. Total length: 4.0–4.4 mm.

##### Diagnosis.

This species is similar to *Cephaloleia tucumana*. It can be distinguished by antennomere 2 being obconic and by the pronotum having a medial basal impression.

##### Distribution.

Brazil (Matto Grosso, Pará), Peru.

##### Type material examined.

Syntype: Brasilia, Cuyaba, Matto Grosso, Staudinger [green printed label]/ J. Weise det. [printed label] Type [printed salmon-colored label]/ Cephalolia humeralis m [handwritten label] (ZMHB, 1).

##### Specimens examined.

**Brazil:** ?- Chapada (USNM). Pará- no further data (USNM). **Peru:** Lima- 2000–3000 ft. (USNM). Made de Dios- Cocha Salvador Reserved Zone, Manu National Park, 310 m, 20–21 October 2000 (SEMC). Total: 5.

#### Cephaloleia immaculata

Taxon classificationAnimaliaColeopteraChrysomelidae

Staines, 1996

http://species-id.net/wiki/Cephaloleia_immaculata

Fig. 163

Cephaloleia immaculata Staines 1996: 38. McKenna and Farrell 2005: 119 (phylogeny), 2006: 10949 (phylogeny).

##### Description.

Large; elongate; subparallel; subdepressed; reddish-brown, eyes darker; venter with meso- and metasterna paler medially, darker laterally. Head: vertex sparsely punctate, medial sulcus absent; frons not projecting; not depressed between eyes. Antenna: reaches to humerus; robust; antennomere 1 incrassate, as long as 2–4 combined; 2–3 transverse, each with projection on inner apex; 4–10 transverse, decreasing in length; 11 2× length of 10, pointed at apex; 1–4 punctate with scattered setae; 5–11 setose. Pronotum: transverse; lateral margin straight then rounding to anterior angle, margined; anterior angle rounded, slightly produced; posterior angle acute; anterior margin weakly emarginate behind head; disc flattened; surface sparsely punctate; basal impression absent; pronotal length 1.3–1.6 mm; pronotal width 1.9–2.0 mm. Scutellum: pentagonal; alutaceous. Elytron: lateral margin straight, smooth, margined; apex rounded; sutural angle without tooth; humerus rounded, slightly produced; slightly constricted behind humerus; shallowly punctate-striate, humerus virtually impunctate; rows converge and unite near apex; declivity beginning just behind humerus along puncture row 7 with weak carina; elytral length 5.0–5.7 mm; elytral width 2.3–2.7 mm. Venter: pro-, meso-, and metasterna impunctate medially, punctate laterally; abdominal sterna punctate, each puncture with pale seta; suture between sterna 1 and 2 obsolete medially. Leg: robust; impunctate; tibia with fringe of setae on inner margin of apex. Total length: 6.6–7.8 mm.

##### Diagnosis.

This species is similar to *Cephaloleia congener*. It can be distinguished by the sparsely punctate pronotum, by the suture between abdominal sterna 1 and 2 being obsolete medially, and by antennomere 1 being subequal in length to 2 to 4 combined.

##### Distribution.

Costa Rica.

##### Type material examined.

Holotype: Estacion Boscosa, Peninsula de Osa, Pro. Punt. Costa Rica, R. W. Flowers, R. Aguilar. 15 Set 1991 L-N-294500, 517000/ Costa Rica CR10000, 516949/ Holotype Cephaloleia immaculata Staines, Des. C. L. Staines 1994 [red label] (INBIO).

##### Specimens examined.

**COSTA RICA:** Puntarenas- Rancho Quemado, 200 m, Peninsula de Osa, August 1992, September 1992, October 1992 (INBIO); Est. Sirena, P. N. Corcovado, 0–100 m, October 1989 (INBIO, USNM). Total: 14.

#### Cephaloleia impressa

Taxon classificationAnimaliaColeopteraChrysomelidae

Uhmann, 1930c

http://species-id.net/wiki/Cephaloleia_impressa

Fig. 164

Cephalolia impressa Uhmann 1930c: 36.
Cephaloleia impressa Uhmann. Uhmann 1957b: 20 (catalog); Staines 1997b: 413 (Uhmann species list).

##### Description.

Oblong-ovate; subconvex; shining; black; palps, tarsi, antennae (except basal 4 antennomeres) reddish-brown. Head: vertex finely, sparsely punctate, with sharp medial carina; keel present between antennal bases; frons not projecting; not depressed between eyes. Antenna: as long as head and pronotum combined; robust; slightly compressed laterally; antennomeres 1–4 subquadrate, subequal in length; 5–10 transverse, slightly decreasing in length; 11 longer than 10, pointed at apex; 1–2 punctate with scattered setae; 3–11 setose. Pronotum: transverse; lateral margin straight then rounding on apical ⅓ to anterior angle, slightly canaliculate; anterior angle rounded, produced; posterior angle acute; anterior margin weakly emarginate behind head; disc subconvex; surface finely, irregularly punctate; basal impression absent; pronotal length 0.8 mm; pronotal width 1.1 mm. Scutellum: pentagonal; impunctate. Elytron: lateral margin narrowing apically, smooth, finely margined; apex weakly rounded, faintly crenulate; sutural angle without tooth; humerus rounded, not produced; constricted behind humerus; subconvex; finely punctate-striate; declivity beginning just behind humerus along puncture row 7 with weak carina; elytral length 2.5 mm; elytral width 1.5 mm. Venter: obscured by card. Leg: slender; punctate, each puncture with pale seta; tibia with fringe of setae on inner margin of apex. Total length: 3.5 mm.

##### Diagnosis.

This species is similar to *Cephaloleia funesta* and *Cephaloleia obsoleta*. It can be distinguished by the vertex of the head having a medial carina.

##### Distribution.

Brazil (São Paulo).

##### Type material examined.

Holotype: Brazil, São Paulo, Mráz [printed label]/ Holotypus [red printed label]/ Cephalolia impressa Uh., Det. E. Uhmann (NHMW).

#### Cephaloleia insidiosa

Taxon classificationAnimaliaColeopteraChrysomelidae

Pic, 1923

http://species-id.net/wiki/Cephaloleia_insidiosa

Fig. 165

Cephalolia insidiosa Pic 1923: 9. Uhmann 1938a: 411 (noted).
Cephaloleia insidiosa Pic. Uhmann 1957b: 20 (catalog); Descarpentries and Villiers 1959a: 139 (types).

##### Description.

Suboblong; small; subconvex; head and pronotum black; legs and apex of abdomen red; elytra testaceous with black macula at base near humerus, a second macula near basal margin approaching suture, and a medial black shallow V-shaped transverse band from suture to near lateral margin; antennae yellowish-brown. Head: vertex densely punctate, medial sulcus absent; frons not projecting; depressed between eyes. Antenna: reaches beyond humerus; slender; antennomeres 1–2 robust, elongate, cylindrical, 1 longest; 3–5 elongate, cylindrical, decreasing in length; 6–10 transverse, decreasing in length; 11 2× length of 10, acutely pointed at apex; 1–2 punctate with scattered setae; 3–11 setose. Pronotum: quadrate; lateral margin straight for basal 4/5 then rounding to anterior angle, margined; anterior angle rounded, not produced; posterior angle acute; anterior margin straight; disc subconvex; surface coarsely, irregularly punctate; transverse basal impression present medially; pronotal length 0.8–1.0 mm; pronotal width 0.9–1.1 mm. Scutellum: pentagonal; impunctate. Elytron: lateral margin straight, smooth, margined; apex rounded; sutural angle without tooth; humerus rounded, not produced; slightly constricted behind humerus; coarsely punctate-striate; elytral length 2.7–3.1 mm; elytral width 1.3–1.5 mm. Venter: pro-, meso-, and metasterna impunctate medially, punctate laterally; abdominal sterna punctate, each puncture with pale seta; suture between sterna 1 and 2 complete; last sternite with apical margin subtruncate in male, rounded in female. Leg: slender; punctate; femur and tibia with row of setae on inner margin; tibia with fringe of setae on inner margin of apex. Total length: 3.9–4.3 mm.

##### Diagnosis.

This species is similar to *Cephaloleia uniguttata*. It can be distinguished by the totally black pronotum and by the elytra with a black humeral and scutellar macula basally and an irregular transverse band from the suture to the lateral margin.

##### Distribution.

Ecuador.

##### Type material.

Type: Ecuador (MNHN, not seen).

##### Specimens examined.

**Ecuador:** no further data (MNHN). Esmeraldas- Canton San Lorenzo Chuchubi, 2 December 2008 (BYUC); 31.7 km NW Lita, 620 m, 23 August 1997 (CDFA). Imbabura- Cachabé, December 1896 (USNM); Cachabé to Paramba, February 1897 (USNM); Paramba, 5500 ft., May 1887 (USNM). Pichincha- Chimba, 3000 ft., August 1897 (USNM). Total: 14.

#### Cephaloleia instabilis

Taxon classificationAnimaliaColeopteraChrysomelidae

Baly, 1885

http://species-id.net/wiki/Cephaloleia_instabilis

Fig. 166

Cephaloleia instabilis Baly 1885: 18. Blackwelder 1946: 719 (catalog); Papp 1953: 18 (catalog); Uhmann 1957a: 20 (catalog); Wilcox 1983: 137 (catalog); Strong 1977b: 573 (host plants), 1981: 184 (host plants), 1982b: 1045 (host plants); Staines 1996: 39 (Central America species), 1999: 242 (mimicry), 2004: 312 (host plants); Staines and Staines 1997: 10 (types), 1999: 524 (Baly species list); McKenna and Farrell 2005: 119 (phylogeny), 2006: 10949 (phylogeny); Meskins 2008: 163 (host plants), 2011: 483 (food web); Descampe et al. 2008: 227 (host plants); Schmitt and Frank 2013: 58 (biology).
Cephalolia instabilis Baly. Donckier 1899: 549 (catalog); Weise 1905a: 131 (noted), 1911a: 8 (catalog), 1911b: 10 (catalog); Uhmann 1936b: 483 (key), 1942: 93 (noted).
Cephaloleia intermedia Baly 1885: 19 (type: Panama, Bugaba, BMNH, not seen). Staines and Staines 1999: 524 (Baly species list).
Cephalolia intermedia Baly. Donckier 1899: 549 (catalog); Weise 1905a: 131 (synonymy).
Cephaloleia instabilis gilvipennis Weise 1905a: 131 (type: Mexico, ZMHB, not seen). Weise 1911a: 8 (catalog), 1911b: 10 (catalog); Blackwelder 1946: 719 (catalog); Uhmann 1957a: 21 (catalog); Wilcox 1983: 137 (catalog).
Cephalolia instabilis gilvipennis Weise. Uhmann 1936b: 483 (key).
Cephaloleia instabilis obscura Weise 1905a: 131 (type: México, NHRS). Weise 1911a: 8 (catalog); Blackwelder 1946: 719 (catalog); Uhmann 1957a: 21 (catalog); Wilcox 1983: 137 (catalog).
Cephaloleia insignis Meskins et al. 2008: 166 (misspelling, host plants).

##### Description.

Large; elongate; subparallel; subconvex; head, antennae, and scutellum black; pronotum reddish-brown with variable black markings; elytra varies from totally reddish-brown, to variable black markings, to totally black; venter variable in color; leg with protibia black, profemur reddish-yellow basally, black apically. Head: vertex and front densely punctate, medial sulcus present; frons not projecting; not depressed between eyes. Antenna: nearly ½ body length; robust; antennomeres 1–4 compressed; 1 clavate, longest of all; 2–4 triangular, subequal in length in male, 3 longer in female; 4–10 transverse, decreasing in length; 11 2× length of 10, pointed at apex; 1–3 punctate with scattered setae; 4–11 setose. Pronotum: slightly wider than long; lateral margin straight then rounding to anterior angle, slightly canaliculate; anterior angle rounded, slightly produced; posterior angle acute; anterior margin emarginate behind head; disc flattened; surface sparsely punctate; disc nearly impunctate; depressed near side; pronotal length 1.3–1.5 mm; pronotal width 1.58–2.16 mm. Scutellum: longer than wide, acutely triangular; alutaceous. Elytron: lateral margin straight, smooth, margined; apex rounded; sutural angle with tooth; humerus rounded, not produced; slightly constricted behind humerus; finely punctate-striate; declivity beginning just behind humerus along puncture row 7 with weak carina; elytral length 5.3–6.4 mm; elytral width 2.2–2.7 mm. Venter: pro-, meso-, and metasterna impunctate; abdominal sterna punctate, each puncture with pale seta; suture between sterna 1 and 2 complete; last sternite with apical margin entire, rounded in female, sinuate medially in male. Leg: slender; profemur more robust than others; metatibia punctate, others impunctate, all with fringe of setae at apex. Total length: 6.88–8.32 mm.

##### Diagnosis.

This species is similar to *Cephaloleia stenosoma*. It can be distinguished by antennomeres being triangular, by the pronotum being punctate laterally, and by the elytra having a declivity from puncture row 7.

##### Host plant.

*Heliconia latispatha* Benth., *Heliconia difficilis* [sic], *Heliconia imbricata* (Kuntze) Baker, *Heliconia wagneriana* Petersen (Heliconiaceae) (Strong 1977b); *Calathea latifolia* Klotzsch (Marantaceae), *Heliconia catheta* R. R. Smith, *Heliconia mariae* Hook. (Heliconiaceae) (Meskins et al. 2008); *Heliconia rostrata* Ruiz & Pav. (Schmitt and Frank 2013); *Musa velutina* H. Wendl. and Drude (Musaceae), *Calathea crotalifera* S. Watson, *Heliconia wilsonii* G. S. Daniels and F. G. Stiles.

##### Distribution.

Costa Rica, Guatemala, Mexico, Panama.

##### Type material examined.

Lectotype: male/ H.T. [white disk with red border]/ Cubilguitz, Vera Paz. Champion/ Cephaloleia instabilis/ Sp. figured/ B. C. A, Col. VI, 2. Cephaloleia instabilis Baly/ Cephaloleia instabilis Baly C. America/ Lectotype Cephaloleia instabilis Baly des. C. L. Staines 1993 [red label] (BMNH).

##### Specimens examined.

**COSTA RICA:** Cartago- Turrialba, 26 May 1951 (USNM). Puntarenas- 5 km S. Buenos Aires, 15 August 1969 (USNM); Est. Queb. Bonita, 50 m, Res. Biol. Carara, 30 April, 1–29 July 1992, August 1992, 2–23 September 1992, January 1993, 4–26 January 1993, February 1993, 8 February 1993, November 1992, 6–27 November 1992 (INBIO); Estación Boscosa, Peninsula de Osa, 15 September 1991 (INBIO); Coto Brus, Las Cruces Biological Station, 5 March 2012, 6 March 2012, 10 March 2012 (USNM); Fca. Las Cruces, San Vito de Java, 27 June 1969, 11–14 August 1969 (USNM); F. Las Cruces, 6 km. S. San Vito, 1200–1400 m, 21–25 August 1976 (CASC); 1.5 mi S Palmar Sur, 11 August 1969 (USNM); Puerto Cortes, 19 July 1972 (FSCA); Rancho Quemado, 200 m, Peninsula de Osa, November 1991, April 1992, October 1992 (INBIO); 3.5 mi. S. Rincón, 28 February- 12 March 1969 (CASC); 5 km S. Rincón, 20 March 1973 (SEMC); Río Claro, sea level, 19 August 1969 (USNM); 22 mi SW San Vito, 11 August 1969 (USNM); San Vito-Villa Neilly area, 13 August 1969 (USNM); Wilson Botanical Garden (Las Cruces Biol. Stn.) nr. San Vito, 1200 m, 26 May 1993 (SEMC); 22 m. SW San Vito, 11 August 1969 (USNM); San Vito-Villa Neilly area, 13 August 1969 (USNM); F. Las Cruces, Laguna Gamboa, 1400–1500 m (INBIO); Estación Esquinas, Peninsula de Osa, 0–100 m (INBIO); Pque Nal Corcovado, Est Sirena, Playa Sirena, 0–100 m (INBIO); Est Boscoas, 0–100 m (INBIO). **GUATEMALA:** Irebal- Sierra, Espíritu Sto. Amates, San Antonio, 10 August 1990 (EGRC). Verapaz- Cahabón (BMNH, USNM); Chiacam (BMNH); Lanquín (BMNH). **PANAMA:** Chilbre- Chilibrillo Caves, 3 January 1945 (CASC). Colón- Paraiso, 26 January 1911, 5 February 1911, 12 February 1911 16 March 1911, 17 March 1911, 20 March 1911, 26 March 1911, 2 April 1911, 5 April 1911 (USNM). Panamá- Alajuela, 5 April 1911, 18 April 1911 (USNM); Arraiján (USNM); Cerro Campana, 17 May 1993 (CDFA); Corazal, 12 March 1911 (USNM); Las Cascadas, 30 March 1911 (USNM); Old Gamboa Road, 4 June 1993 (CDFA); Pedro Miguel, 17 April 1911 (USNM). Total: 218.

#### Cephaloleia interrupta

Taxon classificationAnimaliaColeopteraChrysomelidae

García-Robledo & Staines sp. n.

http://zoobank.org/6D6120A1-3746-4CCC-917D-E2ADB411434D

http://species-id.net/wiki/Cephaloleia_interrupta

Fig. 167

##### Description.

Small; elongate; subparallel; subconvex; head and scutellum black; antennae pitchy-brown; pronotum and legs pale yellowish; elytral pale yellowish with short black vitta along suture just behind scutellum which ends before middle, subequal in length to length of scutellar row, and elongate oval black macula behind middle on puncture rows 2–5; venter with pro-, meso- and metasterna black, abdominal sterna reddish-yellow. Head: vertex densely punctate, medial sulcus present; slightly depressed between eyes; frons punctate. Antenna: reaches to humerus; elongate; filiform; antennomeres 1–2 subequal in length, elongate; 3 elongate, cylindrical, 1½ length of 2; 4–5 subequal in length, expanding to apex, each ½ length of 3; 6–10 transverse, subequal in length, each ¾ length of 5; 11 2× length of 10, pointed at apex; 1–2 punctate, each puncture with pale seta; 3–11 hirsute. Pronotum: transverse; subconvex; lateral margin straight for basal ¾ then rounding to anterior angle, margined; anterior angle with obtuse tooth; posterior angle acute; anterior margin weakly emarginate behind head; surface irregularly punctate; basal impression absent; pronotal length 1.0 mm; pronotal width 1.1–1.2 mm. Scutellum: pentagonal; micropunctate. Elytron: lateral margin straight, margined; exterior apical angle rounded, margined; apical margin rounded, margined; sutural angle without tooth; humerus rounded, not produced; slightly constricted behind humerus; declivity absent; moderately punctate-striate; puncture rows converge and untie apically; rows 4–7 irregularly interrupted basally; elytral length 3.2 mm; elytral width 1.8–1.9 mm. Venter: pro-, meso-, and metasterna punctate, glabrous; suture between abdominal sterna 1 and 2 complete; abdominal sterna punctate laterally, glabrous, last sternite with pale white setae, apical margin rounded in male, emarginate in female. Leg: tibia and femur punctate, each puncture with pale seta; tibia with fringe of setae on inner margin near apex. Total length: 4.3–4.5 mm.

##### Etymology.

From interruptum (Latin) for the interrupted elytral puncture rows at base. The name is feminine.

##### Diagnosis.

This species is similar to *Cephaloleia eumorpha* and *Cephaloleia postuma*. It can be distinguished by antennomere 1 being subequal in length to 2 and by lacking a V-shaped depression basally on the pronotum.

##### Host plant.

Adults have been collected off *Costus* sp. (Costaceae). The name is feminine.

##### Distribution.

Costa Rica.

##### Type material.

Holotype male: Costa Rica, Heredia, Braulio Carrillo National Park, 1500 m, 14 February 2013, 1500 m, C. García-Robedo, CG MAY 4–13–2, feeding on *Costus* aff. *scaber*/ ♂/Holotype *Cephaloleia interrupta* García-Robledo & Staines, des. C. L. Staines 2013 [red label] (USNM); Paratype male: Costa Rica, Heredia, Braulio Carrillo National Park, 1500 m, 14 February 2013, 1500 m, C. García-Robledo, CG MAY 4–13–1, feeding on *Costus* aff. *scaber*/ ♂/Paratype *Cephaloleia interrupta* García-Robledo & Staines, des. C. L. Staines 2013 [red label] (USNM); Paratype female: Costa Rica, Heredia, Braulio Carrillo National Park, 1500 m, 14 February 2013, 1500 m, C. García-Robledo, CG MAY 4–13–3, feeding on *Costus* aff. *scaber*/ ♀/ Paratype Cephaloleia interrupta García-Robledo & Staines, des. C. L. Staines 2013 [red label] (USNM).

#### Cephaloleia interstitialis

Taxon classificationAnimaliaColeopteraChrysomelidae

Weise, 1904b

http://species-id.net/wiki/Cephaloleia_interstitialis

Fig. 168

Cephalolia interstitialis Weise 1904b: 437. Weise 1911a: 8 (catalog), 1911b: 11 (catalog); Uhmann 1936b: 112 (noted), 1953d: 47 (faunal list).
Cephaloleia interstitialis Weise. Uhmann1957b: 21 (catalog); Gaedike and Döbler 1971: 351 (types).

##### Description.

Elongate; subparallel; subdepressed; shining; reddish-yellow; apical three antennomeres and eyes darkened. Head: vertex finely, distinctly punctate, medial carina present; frons not projecting; depressed between eyes. Antenna: reaches to humerus; slender; antennomere 1 incrassate, elongate, obliquely truncate at apex; 2 ½ length of 1, cylindrical; 3 longer than 2; 4–10 transverse, subequal in length, each shorter than 3; 11 2× length of 10, pointed at apex; 1–3 punctate with scattered setae; 4–11 setose. Pronotum: transverse; lateral margin straight then rounding to anterior angle, canaliculate; anterior angle rounded, produced; posterior angle acute; anterior margin emarginate behind head; disc subconvex; surface distinctly, finely punctate with longitudinal area medially impunctate; slight transverse basal impression present medially; pronotal length 1.2–1.4 mm; pronotal width 1.8–2.0 mm. Scutellum: pentagonal; impunctate. Elytron: lateral margin straight, smooth, narrowly margined; apex rounded; sutural angle without tooth; humerus rounded, not produced; slightly constricted behind humerus; weakly punctate-striate, punctures large, confused apically; elytral length 3.3–4.2 mm; elytral width 2.0–2.2 mm. Venter: pro- and mesosterna impunctate medially, punctate laterally; metasternum punctate; abdominal sterna punctate, each puncture with pale seta; suture between sterna 1 and 2 complete. Leg: robust; punctate; tibia with fringe of setae on inner margin of apex. Total length: 5.0–6.0 mm.

##### Diagnosis.

This species is similar to *Cephaloleia striata*, *Cephaloleia subdepressa*, *Cephaloleia truncatipennis*, and *Cephaloleia unctula*. It can be distinguished by the transverse basal impression on the pronotum and by antennomere 1 being longer than 2.

##### Distribution.

Brazil (Amazonas, Pará, Rondonia), Peru.

##### Type material examined.

Syntype male: Brasilia, Amazonas, Staudinger [green printed label]/ I. Weise det. [printed label]/ Type [printed salmon-colored label]/ Cephalolia interstitialis m. [handwritten label] (DEI, 1).

##### Specimens examined.

No label data (USNM). **Brazil:** Pará- Belem, 10–22 November 1963 (AMNH); Val de Cans, 20–21 November 1963 (AMNH). Rondonia- 62 km SW Ariquames, Fzda Rancho Grande, 18 November 1994 (BYUC). Total: 4.

#### Cephaloleia irregularis

Taxon classificationAnimaliaColeopteraChrysomelidae

Uhmann, 1930a

http://species-id.net/wiki/Cephaloleia_irregularis

Fig. 169

Cephalolia irregularis Uhmann 1930a: 231.
Cephaloleia irregularis Uhmann. Blackwelder 1946: 719 (catalog); Papp 1953: 18 (catalog); Uhmann 1957a: 21 (catalog); Wilcox 1983: 137 (catalog); Staines 1996: 40 (Central America species), 1997: 413 (Uhmann species list), 1999: 241 (mimicry); Staines and Staines 1997: 11 (types); McKenna and Farrell 2005: 119 (phylogeny), 2006: 10949 (phylogeny); Sekerka et al. 2013: 305 (comparative note).

##### Description.

Cantharid-like in appearance; oblong; subparallel; subdepressed; dull; head (except at antennal insertions which are brown), antennae (except basal antennomere), and elytra black, pronotum red with inverted black V-shaped vitta from base to apex; venter with pro-, meso-, and metasterna reddish laterally, black medially; abdominal sterna black medially, yellow laterally; legs with base of femur yellowish, rest with upper surface dark, lower surface yellowish. Head: vertex densely punctate, medial sulcus absent; frons not projecting; not depressed between eyes. Antenna: ½ body length; slender; antennomere 1 transverse, ¾ length of 2; 2 elongate; 3 elongate, longer than 1 and 2 combined; 4–10 elongate, subequal in length, each ¾ length of 3; 11 longer than 10, rounded at apex; 1–2 punctate with scattered setae; 3–11 setose. Pronotum: transverse but much narrower than base of elytra; lateral margin straight then rounding to anterior margin, margined; anterior angle rounded, slightly produced; posterior angle acute; anterior margin sinuate; disc subconvex; surface punctate, more so laterally; basal impression absent; pronotal length 0.8 mm; pronotal width 1.5 mm. Scutellum: broadly triangular; micropunctate. Elytron: lateral margin straight, smooth, margined; apex rounded; sutural angle without tooth; humerus rounded, not produced; slightly constricted behind humerus; disc flattened behind humerus laterally, irregularly punctate; declivity beginning just behind humerus at puncture row 7 not edged with faint carina; elytral length 4.7 mm; elytral width 2.0 mm. Venter: pro-, meso-, and metasterna impunctate medially, punctate laterally; abdominal sterna punctate each puncture with seta; suture between sterna 1 and 2 complete. Leg: slender; sparsely punctate; tibia with fringe of setae on inner margin of apex. Total length: 6.0 mm

##### Diagnosis.

This species is similar to *Cephaloleia orchideivora*. It can be distinguished by the irregular elytral punctation, by not having an additional row of elytral punctures, and by the anterior margins of the pronotum being straight.

##### Distribution.

Costa Rica.

##### Type material examined.

Holotype: Costa Rica, F. Nevermann, II-26 [green label]/ Coronado, 1400–1500 m., T. Assmann leg. [reversed green label]/ Holotype [red label]/ Type No. 54602 USNM [orange label]/ *Cephalolia irregularis* sp. n. (USNM).

#### Cephaloleia kolbei

Taxon classificationAnimaliaColeopteraChrysomelidae

Weise, 1910

http://species-id.net/wiki/Cephaloleia_kolbei

Fig. 170

Cephaloleia kolbei Weise 1910: 85. Weise 1911a: 8 (catalog), 1911b: 10 (catalog); Uhmann 1957b: 21 (catalog).
Cephalolia kolbei Weise. Uhmann 1936b: 110 (noted), 1936f: 482 (key).

##### Description.

Slightly elongate; subparallel; large; subconvex; shining; reddish; elytra with dark macula near scutellum, a second at humerus and third near apex. Head: vertex sparsely, irregularly punctate, medial sulcus absent; frons not projecting; not depressed between eyes. Antenna: reaches to humerus; slender; antennomere 1 incrassate, elongate; 2 ½ length of 1; 3 longer than 2 but shorter than 1; 4–10 transverse; 4 shorter than 3; 5 as long as wide, shorter than 4; 6 longer than 5; 7–10 subequal in length, each shorter than 6; 11 2× length of 10, elongate-oval; 2–4 triangularly compressed in male; 1–5 punctate with scattered setae; 6–11 setose. Pronotum: transverse; lateral margin straight, slightly divergent then rounding to anterior angle, margined; anterior angle angulate, slightly produced; posterior angle acute; anterior margin emarginate behind head; disc flattened; surface sparsely, irregularly punctate; basal impression absent; pronotal length 1.9–2.1 mm; pronotal width 2.4–2.6 mm. Scutellum: pentagonal; sparsely punctate. Elytron: lateral margin straight, smooth, narrowly margined; apex rounded; sutural angle without tooth; humerus rounded, not produced; slightly constricted behind humerus; moderately punctate-striate, punctures confused apically; elytral length 7.5–7.8 mm; elytral width 3.1–3.5 mm. Venter: pro-, meso-, and metasterna impunctate; abdominal sterna punctate, each puncture with pale seta; suture between sterna 1 and 2 complete; last sternite with apical margin weakly rounded, almost truncate in male, bisinuate in female. Leg: slender; impunctate; tibia with fringe of setae on inner margin of apex. Total length: 9.7–10.2 mm.

**Figures 170–178.**
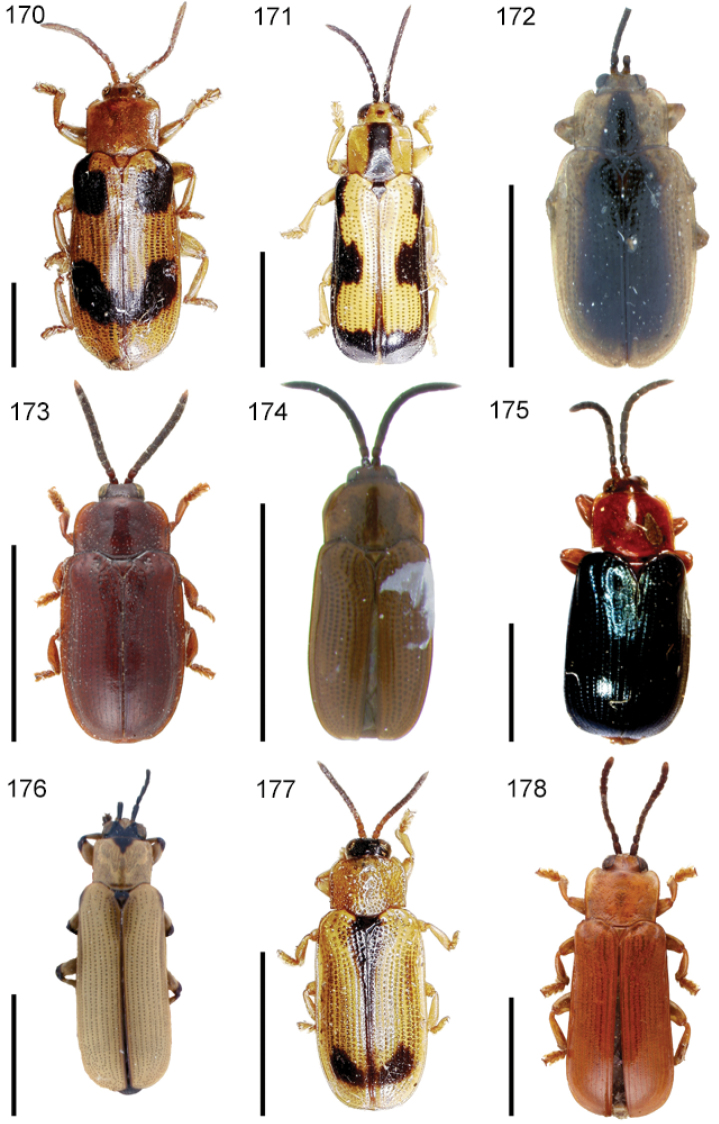
Habitus. **170** *Cephaloleia kolbei* **171** *Cephaloleia laeta* **172** *Cephaloleia lateralis* **173** *Cephaloleia latipennis* **174** *Cephaloleia lenticula* sp. n. **175** *Cephaloleia lepida* **176** *Cephaloleia leucoxantha* **177** *Cephaloleia linkei* **178** *Cephaloleia lojaensis*. Scale bars equal 3 mm.

##### Diagnosis.

This species is similar to *Cephaloleia quinquemaculata* and *Cephaloleia sagittifera*. It can be distinguished by the lateral margin of the pronotum being straight and divergent, not canaliculate.

##### Distribution.

Bolivia, Brazil (Bahia), Colombia, Ecuador, Peru.

##### Type material.

Type: Colombia, Cauca bei Cali (ZMHB, not seen).

##### Specimens examined.

No label data (USNM). **BOLIVIA:** Cochabamba- Chapare, January 1952, November 1953, December 1985 (USNM). **BRAZIL:** Bahia- São Paulo d’Olivenca (USNM). **Colombia:** Valle de Cauca- Cauca to Cali (ZMHB). **Ecuador:** Napo- Huaticocha, 19 August 1997 (USNM); Limoncocha, 7 June 1977 (USNM). **PERU:** Madre de Dios- CICRA Field Station, 272 m, 12 June 2011 (SEMC). Pasco- Villa Pica-Puerto Bermudas Rd., 1350 m, 17 October 1999 (SEMC, USNM). Ucayali- Tingo Maria-Pucalipa Rd., Puente Chipo km 205, 1300 m, 14 October 1999 (SEMC, USNM). Total: 79.

#### Cephaloleia kressi

Taxon classificationAnimaliaColeopteraChrysomelidae

García-Robledo sp. n.

http://zoobank.org/456C3CAB-1AFA-4395-B7D6-C0778A7E01EE

http://species-id.net/wiki/Cephaloleia_kressi

Fig. 272

##### Description.

Elongate; parallel-sided; subdepressed; head black, mouthparts yellowish; antenna black except for apex of antennomere 11 (some specimens with antennomeres 1–2 paler); pronotum yellow; scutellum black; elytra black with wide longitudinal yellow vitta from humerus to near apex; venter with pro-, meso-, and metasterna fuscous, abdominal sterna yellowish medially darker laterally; legs yellow, joints and tarsi darker. Head: vertex striate-punctate, with wide medial sulcus, not depressed between eyes; slight swelling present between antennal bases; clypeus punctate. Antenna: reaches to humerus; antennomere 1 elongate, as long as 2 to 4 combined, incrassate, expanding apically; 2–3 subequal in length, triangularly produced; 4 triangularly produced, shorter than 3; 5–10 cylindrical, increasing in length; 11 longer than 10, pointed at apex; 1–2 punctate; 3–11 punctate, each puncture with dark seta. Pronotum: transverse, widest at base; lateral margin smooth, margined; anterior angle rounded; anterior margin straight; posterior angle subacute; posterior margin bisinuate; disc flattened; surface sparsely, irregularly punctate; pronotal length 1.1–1.3 mm; pronotal width 1.4–1.6 mm. Scutellum: elongate, triangular, punctate. Elytron: lateral margin straight, smooth, margined; rounding to sutural angle; humerus rounded, not produced; slightly constricted behind humerus; surface faintly punctate-striate, declivity present beginning at puncture row 7, without carina; elytral length 5.3–5.7 mm; elytral width 2.0–2.3 mm. Venter: pro-, meso-, and metasterna impunctate medially, punctate laterally; abdominal sterna punctate, each puncture with pale seta; suture between abdominal sterna 1 and 2 complete; apical margin of last sternite truncate weakly notched in male, weakly rounded in female. Leg: elongate; robust; finely punctate; tibia with fringe of setae on inner apical margin. Total length: 7.0–7.6 mm.

##### Etymology.

Named for W. John Kress in recognition of his many contributions to the understanding of the ecology and evolution of plant animal interactions, in particular between plants from the order Zingiberales, their pollinators and *Cephaloleia* insect herbivores. Also in recognition of his mentorship of new generations of researchers. The name is a noun in apposition.

##### Diagnosis.

*Cephaloleia kressi* sp. n. is most similar to *Cephaloleia adusta* Uhmann. It can be distinguished by the following combination of characters: vertex of head striate-punctate; vertex of head with wide medial sulcus; frons not projecting; elytra with sutural angle rounded; and elytral puncture rows being visible after middle.

##### Host plant.

*Heliconia lankesteri* Standl. (Heliconiaceae).

##### Distribution.

Costa Rica.

##### Type material.

Holotype male: Costa Rica: Heredia Prov., Braulio Carrillo National Park, Refugia 2000 m, 10 July 2013, Carlos García-Robledo, *Heliconia lankesteri*/ Holotype *Cephaloleia kressi* García-Robledo, des. C. García-Robledo 2014 (red label), USNM. Paratypes (each with Paratype *Cephaloleia kressi* García-Robledo, des. C. García-Robledo 2014 [red label]). (37 males, 7 females): same label data as holotype, USNM, BMNH, TAMU, IEXA.

#### Cephaloleia laeta

Taxon classificationAnimaliaColeopteraChrysomelidae

Waterhouse, 1881

http://species-id.net/wiki/Cephaloleia_laeta

Fig. 171

Cephaloleia laeta Waterhouse 1881: 262. Blackwelder 1946: 719 (catalog); Uhmann 1951: 72 (noted), 1957a: 21 (catalog); Papp 1953: 18 (catalog); Staines 1996: 40 (Central America species).
Cephalolia laeta Waterhouse. Donckier 1899: 549 (catalog); Weise 1911a: 8 (catalog), 1911b: 11 (catalog); Uhmann 1931: 219 (museum list), 1936a: 114 (noted).

##### Description.

Elongate; subparallel; subconvex; shining, yellow; eyes, antennae, and scutellum black; pronotum with black medial vitta; elytra with black lateral and apical maculae. Head: vertex impunctate, with small medial carina; frons not projecting; slightly depressed between eyes. Antenna: reaches to humerus; slender; antennomeres 1–10 cylindrical; 1 elongate, clavate; 2 ½ length of 1, transverse, shortest; 3–4 elongate, 3 ¾ length of 1, 4 shorter than 3; 5–10 transverse, slightly decreasing in length; 11 rounded at apex, 2× length of 10; 1–2 punctate with scattered setae; 3–11 setose. Pronotum: transverse; lateral margin straight for basal ¾ then rounding to anterior angle, slightly canaliculate; anterior angle rounded, not produced; posterior angle acute; anterior margin weakly emarginate behind head; disc subconvex; surface virtually impunctate, a few punctures present laterally; basal impression absent; pronotal length 1.3–1.5 mm; pronotal width 1.6–1.8 mm. Scutellum: pentagonal; impunctate. Elytron: lateral margin straight, smooth, margined; apex rounded; sutural angle without tooth; humerus rounded, not produced; slightly constricted behind humerus; humerus virtually impunctate; declivity beginning just behind humerus at puncture row 7 not edged with faint carina; moderately punctate-striate; rows converge and unite apically; elytral length 4.6–5.2 mm; elytral width 2.1–2.3 mm. Venter: pro-, meso-, and metasterna impunctate; abdominal sterna punctate, each puncture with pale seta; suture between sterna 1 and 2 complete; last sternite with apical margin rounded in male, truncate in female. Leg: slender; sparsely punctate; tibia with fringe at apex. Total length: 6.3–7.0 mm.

##### Diagnosis.

This species is similar to *Cephaloleia daguana*. It can be distinguished by the vertex of the head not having a small tubercle.

##### Distribution.

Bolivia, Ecuador, Panama (?), Peru.

##### Type material examined.

Holotype: Type H. T. [white disk with red border]/ Balizar m 80–14 [handwritten label]/ Cephaloleia laeta C. Waterh. (Type) [handwritten label] (BMNH).

##### Specimens examined.

**BOLIVIA:** Cochabamba- Chapare, December 1985 (USNM). **ECUADOR:** ?- San Gabriel, 750 m, 12 October 1970 (USNM). Bolivar- Balzapamba, March-April 1884 (USNM). Los Ríos- Quavedo, Est. Exp. Tropical Pichingue, Sta. Rita, 2 February 2008 (USNM), 30 March 2004 (USNM); Río Palenque, 47 km S. Sto. Domingo, 220 m, 26 August 1997 (USNM); Vic. Quebrada, March-April 1955 (USNM). Pichincha- above Chimba, August 1897 (USNM); Estación Orongo, Palmitopomba, 23 July 2001 (USNM); 10.6 km N Mindo, Mindo Road, 28 March 1999 (SEMC). **PANAMA(?):** no further data (DEI). Total: 20.

#### Cephaloleia lateralis

Taxon classificationAnimaliaColeopteraChrysomelidae

Baly, 1885

http://species-id.net/wiki/Cephaloleia_lateralis

Fig. 172

Cephaloleia lateralis Baly 1885: 17. Blackwelder 1946: 719 (catalog); Papp 1953: 18 (catalog); Uhmann 1957a: 21 (catalog); Wilcox 1983: 137 (catalog); Staines 1996: 41 (Central America species); Staines and Staines 1999: 524 (Baly species list).
Cephalolia lateralis Baly. Donckier 1899: 549 (catalog); Weise 1911a: 8 (catalog), 1911b: 12 (catalog).

##### Description.

Small; elongate; subconvex; yellowish; eyes pale, outlined in black; antennomeres 2–11 darker; pronotum with medial black wedge-shaped macula from base to apex, widest at base, narrows anteriorly; scutellum dark brown; elytra dark brownish medially; venter with prosternum reddish-yellow medially, dark laterally; mesosternum reddish-brown medially, dark laterally Head: vertex punctate, medial sulcus absent; frons not projecting; not depressed between eyes. Antenna: more than ½ body length; slender; elongate; antennomere 1 transverse, short, ¾ length of 2; 2 transverse, longer than 1; 3 elongate, as long as 1–2 combined; 4–5 subequal in length, ¾ length 3; rest missing from holotype; 1–2 punctate with scattered setae; 3–5 setose. Pronotum: transverse; lateral margin straight for basal ⅔ then rounding to anterior angle, margined; anterior angle produced, rounded; posterior angle acute; anterior margin emarginate behind head; disc subconvex; surface finely punctate, more dense laterally; basal impression absent; pronotal length 1.5 mm; pronotal width 0.8 mm. Scutellum: pentagonal; micropunctate. Elytron: lateral margin smooth, dilated apically, slightly margined; apex rounded, subangulate, emarginate in sutural angle, with small tooth; humerus rounded, not produced; slightly constricted behind humerus; convex, slightly flattened at suture; moderately punctate-striate, converge and unite on apex; elytral length 3.7 mm; elytral width 2.0 mm. Venter: abdominal sterna punctate, each puncture with seta; suture between sterna 1 and 2 obsolete for entire length; rest obscured by card. Leg: slender; impunctate; tibia with fringe of setae on inner apical margin. Total length: 4.8 mm.

##### Diagnosis.

This species is similar to *Cephaloleia discoidalis*. It can be distinguished by the emarginate sutural angle of the elytra.

##### Distribution.

Guatemala.

##### Type material examined.

Holotype: Type H. T. [white disk with red border]/ Pancina, Vera Paz, Champion [printed label]/ B.C.A. Col. VI, 2. Cephaloleia lateralis Baly [printed label]/ Cephaloleia lateralis Baly, Guatemala [blue handwritten label] (BMNH).

#### Cephaloleia latipennis

Taxon classificationAnimaliaColeopteraChrysomelidae

Pic, 1928

http://species-id.net/wiki/Cephaloleia_latipennis

Fig. 173

Cephalolia latipennis Pic 1928: 4.
Cephaloleia latipennis Pic. Uhmann 1957b: 21 (catalog), 1964a: 403 (catalog); Descarpentries and Villiers 1959a: 139 (types).

##### Description.

Oblong; small; subconvex; shining; dark reddish-brown; eyes and antennae black (except basal 2 antennomeres). Head: vertex finely, irregularly punctate, medial sulcus absent; frons not projecting; slightly depressed between eyes. Antenna: reaches to humerus; robust; antennomere 1 cylindrical, slightly longer than 2; 2–10 transverse, subequal in length; 11 2× length of 10, pointed at apex; 1–2 punctate with scattered setae; 3–11 setose. Pronotum: transverse; lateral margin straight for basal 4/5 then rounding to anterior angle, canaliculate; anterior angle rounded, slightly produced; posterior angle acute; apical margin emarginate behind head; disc subconvex; surface sparsely punctate; basal impression absent; pronotal length 0.9–1.0 mm; pronotal width 1.4–1.6 mm. Scutellum: pentagonal; impunctate. Elytron: lateral margin straight, smooth, distinctly margined; apex rounded; sutural angle without tooth; humerus rounded, slightly produced; slightly constricted behind humerus; moderately punctate-striate, rows converge and unite apically; elytral length 2.9–3.1 mm; elytral width 1.6–1.8 mm. Venter: pro-, meso-, and metasterna impunctate medially, punctate laterally; abdominal sterna sparsely punctate, each puncture with pale seta; suture between sterna 1 and 2 complete. Leg: slender; sparsely punctate, each puncture with pale seta; tibia with fringe of setae on inner margin of apex. Total length: 3.9–4.1 mm.

##### Diagnosis.

This species is similar to *Cephaloleia dimidiaticornis* and *Cephaloleia polita*. It can be distinguished by the elytral puncture rows converging and uniting apically.

##### Distribution.

Bolivia, Ecuador, Peru.

##### Type material examined.

Holotype: Cochabamba, Bolivie, Germain [green printed label]/ *Cephalolia* sp. n. [handwritten label]/ *latipennis* sp. n. [handwritten label]/ Type [handwritten label]/ Museum Paris Coll. M. Pic [blue printed label]/ Type [red printed label]/ Holotype [red printed label]/ *Cephaloleia latipennis* Pic [printed label]/ MNHN EC 2643 [printed label] (MNHN).

##### Specimens examined.

**Bolivia:** Beni- Ruranabaque, 10 July 1958 (USNM). Buena Vista- no further data (USNM). **Ecuador:** Napo- Sacha Lodge, 270 m, 3–13 April 1994, 24 May- 3 June 1994 (SEMC). Orellana- 1 km S Onkone Gare Camp, Reserva Etnica Waorani, 216.3 m, 4 October 1994, 9 October 1994, 11–12 February 1995, 23 June 1996 (USNM); Tiputini Biodiversity Station, nr. Yasuni National Park, 220–250 m, 9 February 1999 (USNM). **Peru:** Loreto- 1.5 km N Teniente Lopez, 210–240 m, 3 July 1993, 18 July 1993 (SEMC). Total: 14.

#### Cephaloleia lenticula

Taxon classificationAnimaliaColeopteraChrysomelidae

Staines sp. n.

http://zoobank.org/DDD1E645-5E84-4C6B-B84B-24BB639B41D8

http://species-id.net/wiki/Cephaloleia_lenticula

Fig. 174

##### Description.

Small; elongate; subdepressed; castaneous, antennomeres 4–10 darker, 11 dark basally and pale apically. Head: vertex irregularly punctate, medial sulcus absent; keel present between antennal bases; frons punctate, not projecting; not depressed between eyes. Antenna: reaches to humerus; slender; antennomere 1 subglobose, shorter than 2; 2 expanding apically; 3–4 cylindrical, elongate, subequal in length, each nearly as long as 1 and 2 combined; 5–10 elongate, subequal in length, each ¾ length of 4; 11 longer than 10, pointed at apex; 1–3 punctate with scattered setae; 4–11 setose. Pronotum: transverse; lateral margin straight basally then rounding to anterior angle, with lenticular swelling anteriorly, margined; anterior angle rounded, slightly produced; posterior angle acute; anterior margin weakly emarginate behind head; disc subconvex; disc micropunctate, scattered larger punctures present laterally; basal impression absent; pronotal length 1.4 mm; pronotal width 1.0 mm. Scutellum: pentagonal; punctate. Elytron: lateral margin straight, smooth, margined; apex obliquely rounded, smooth; sutural angle without tooth; humerus rounded, not produced; slightly constricted behind humerus; shallowly punctate-striate, rows converge and unite apically; elytral length 2.9 mm; elytral width 1.6 mm. Venter: pro-, meso-, and metasterna punctate; abdominal sterna punctate, each puncture with pale seta; suture between sterna 1 and 2 complete. Leg: slender; coxae coarsely punctate; trochanter, femur, and tibia punctate, each puncture with pale seta; tibia with fringe of setae on inner apical margin. Total length: 3.9 mm.

##### Etymology.

Lenticula (Latin) meaning lens for the lens-shaped swelling on the lateral margin of the pronotum. The name is feminine.

##### Diagnosis.

This is a very distinctive species which can be distinguished by the brown color and the lens-like swelling on the lateral margin of the pronotum.

##### Distribution.

Ecuador, French Guiana, Peru, Suriname.

##### Type material.

Holotype: Ecuador Sucumbios, Sacha Lodge, 0.5°S, 76.5°W, 270 m, 4–14-III-1994, Hibbs, ex. malaise/ Holotype *Cephaloleia lenticula* Staines, des. C. L. Staines 2012 (red label) (SMEC). Paratypes (4): Ecuador Sucumbios, Sacha Lodge, 0.5°S, 76.5°W, 270 m, 24-III-3-VI-1994 March 1994, Hibbs, ex. malaise/ Paratype *Cephaloleia lenticula* Staines, des. C. L. Staines 2012 (red label) (SMEC); Peru, Dept. Loreto, 1.5 km N Teniente, 2°35.66'S, 76°06.92'W, 22 July 1993, 210–240 m, Richard Leschen, #165 ex. flt. Intept. Trap Qd. 17/ Paratype *Cephaloleia lenticula* Staines, des. C. L. Staines 2012 (red label) (SMEC); French Guiana, Saul, 7 km N, 0.5 km ESE, Les Eaux Claires, Mt. Le Fumée, 3°39'46"N, 53°13'19"W, 300 m, 4–8 Jun 1997, J. Ashe, R. Brooks, FG1AB97 164, ex. flight intercept trap/ Paratype *Cephaloleia lenticula* Staines, des. C. L. Staines 2012 (red label) (SMEC); Suriname, Commewijne, Akintosoela, CELOS Camp, 39 km SE Suriname River bridge, road to Redi Doti, 40 m, 5°16'17"N, 54°55'15"W, 29 Jun- 3 Jul 1999, Z. H. Falin, SUR1F99 152, ex. flight intercept trap/ Paratype *Cephaloleia lenticula* Staines, des. C. L. Staines 2012 (red label) (SMEC).

#### Cephaloleia lepida

Taxon classificationAnimaliaColeopteraChrysomelidae

Staines, 1996

http://species-id.net/wiki/Cephaloleia_lepida

Fig. 175

Cephaloleia lepida Staines 1996: 42. Staines 2004: 312 (host plants); McKenna and Farrell 2005: 119 (phylogeny), 2006: 10949 (phylogeny).

##### Description.

Elongate; subparallel; subconvex; head, antennae, pronotum, scutellum, and venter reddish-brown; elytra metallic blue. Head: vertex punctate, medial carina present; frons not projecting; not depressed between eyes. Antenna: reaches to humerus; slender; antennomere 1 robust, elongate; 2 transverse; 3 elongate, subequal in length to 1; 4–10 transverse, decreasing in length; 11 rounded at apex, subequal in length to 3; 1–2 punctate with scattered setae; 3–11 setose. Pronotum: transverse; lateral margin straight for basal ¾ then rounding to anterior angle, canaliculate; anterior angle rounded, slightly produced; posterior angle acute; anterior margin emarginate behind head; disc flattened; surface sparsely, irregularly punctate; basal impression absent; pronotal length 1.3–1.4 mm; pronotal width 1.7–2.0 mm. Scutellum: pentagonal, impunctate. Elytron: lateral margin straight, smooth, margined; apex rounded; sutural angle without tooth; humerus rounded, slightly produced; slightly constricted behind humerus; declivity beginning just behind humerus at puncture row 7 edged with faint carina; subconvex; moderately punctate-striate, rows converge and unite apically; row 10 removed from margin; elytral length 4.1–5.1 mm; elytral width 2.3–2.7 mm. Venter: pro-, meso-, and metasterna impunctate medially, punctate laterally; abdominal sterna punctate, each puncture with pale seta; suture between sterna 1 and 2 complete; last sternite with apical margin sinuate medially in male. Leg: femora robust, sparsely punctate; tibia with fringe of setae on inner margin of apex. Total length: 5.7–6.9 mm.

##### Diagnosis.

This species is similar to *Cephaloleia gratiosa*. It can be distinguished by the sparsely punctate pronotum and by the elongate antennomere 1.

##### Host plant.

Adults have been collected in rolled leaves of gingers (Zingiberaceae) (Staines 1996).

##### Distribution.

Panama.

##### Type material examined.

Holotype: Panama, Chiriqui Repr. la Fortuna, 17–21, IX.76: 3200', Col: D. Engleman/ Holotype Cephaloleia lepida Staines, Des. C. L. Staines 1994 [red label] (USNM).

##### Specimens examined.

**PANAMA:** Chiriquí- Repr. La Fortuna, 17–21 September 1976, 3200' (USNM, EGRC); Reserva La Fortuna, Hydrographic sta. trail, 28 May 1993 (EGRC, USNM). Total: 19.

#### Cephaloleia leucoxantha

Taxon classificationAnimaliaColeopteraChrysomelidae

Baly, 1885

http://species-id.net/wiki/Cephaloleia_leucoxantha

Fig. 176

Cephaloleia leucoxantha Baly 1885: 20. Blackwelder 1946: 719 (catalog); Papp 1953: 18 (catalog); Uhmann 1957a: 21 (catalog); Wilcox 1983: 137 (catalog); Staines 1996: 42 (Central America species); Staines and Staines 1999: 524 (Baly species list); McKenna and Farrell 2005: 119 (phylogeny).
Cephalolia leucoxantha Baly. Donckier 1899: 550 (catalog); Weise 1911a: 8 (catalog), 1911b: 10 (catalog).

##### Description.

Elongate; subparallel; subdepressed; head (except yellow front), antennae, and scutellum black; pronotum yellowish with black triangular medial macula on anterior margin; elytra yellowish, suture darker and black macula at humerus; venter prosternum yellowish, meso- and metasterna yellow medially, black laterally, abdominal sterna 2–5 black medially, yellow laterally; leg femur yellowish; tibia and tarsi darker. Head: vertex impunctate, Y-shaped medial sulcus present; frons not projecting; not depressed between eyes. Antenna: reaches to humerus; slender; antennomere 1 elongate, clavate; 2 elongate; 3 compressed, triangular; 4–10 transverse, each shorter than 3; 11 pointed at apex; 1–3 punctate with scattered setae; 4–11 setose. Pronotum: subquadrate; lateral margin straight then rounding to anterior angle, margined; anterior angle rounded, slightly produced; posterior angle acute; anterior margin straight; disc flattened; surface punctate laterally; basal impression absent; pronotal length 1.3 mm; pronotal width 1.5 mm. Scutellum: triangular; impunctate. Elytron: lateral margin straight, smooth, margined; apex rounded; sutural angle without tooth; humerus rounded, slightly produced; slightly constricted behind humerus; flattened at suture; moderately punctate-striate, punctation obsolete at humerus, punctures confused apically; weak declivity beginning just behind humerus at puncture row 7 not edged with faint carina; elytral length 5.0 mm; elytral width 2.0 mm. Venter: pro-, meso-, and metasterna impunctate medially, punctate laterally; abdominal sterna punctate, each puncture with pale seta; suture between sterna 1 and 2 complete. Leg: slender; femur punctate; tibia with fringe of setae on inner margin of apex. Total length: 6.7 mm.

##### Diagnosis.

This species is similar to *Cephaloleia instabilis*. It can be distinguished by by only antennomere 3 being triangular.

##### Distribution.

Panama.

##### Type material examined.

Holotype: V. de Chiriqui 25–4000 ft. Champion [printed label]/ B. C. A, Col. VI, 2. Cephaloleia leucoxantha Baly [printed label]/ Cephaloleia leucoxantha Baly Panama [blue handwritten label] (BMNH).

##### Specimens examined.

**PANAMA:** Chiriquí- 11.2 mi S Chiriqui, 2 June 1994 (CDFA). Total: 1.

#### Cephaloleia linkei

Taxon classificationAnimaliaColeopteraChrysomelidae

Uhmann, 1939

http://species-id.net/wiki/Cephaloleia_linkei

Fig. 177

Cephalolia linkei Uhmann 1939: 153.
Cephaloleia linkei Uhmann. Uhmann 1942b: 101 (pygidium), 1950a: 274 (sculpture), 1957b: 21 (catalog), 1964b: 4 (faunal list); Gaedike and Döbler 1971: 352 (types); Staines 1997b: 413 (Uhmann species list); Staines and Staines 1997: 12 (types).

##### Description.

Elongate; subparallel; subconvex; shining; yellowish-brown, with variable black markings on elytra; venter with pro-, meso-, and metasterna yellowish medially, black laterally; abdominal sterna dark brownish. Head: vertex finely punctate, faint medial sulcus present; frons punctate, not projecting; slightly depressed between eyes. Antenna: reaches to humerus; slender; antennomere 1 incrassate, longer than 2; 2 transverse, ½ length of 1; 3–4 elongate, subequal in length; 5–10 transverse, subequal in length, each shorter than 4; 11 2× length 10, pointed at apex; 1–4 punctate with scattered setae; 5–11 setose. Pronotum: transverse; lateral margin sinuate at base then straight and slightly diverging to apical 1/5 then rounding to anterior angle, margined; anterior angle rounded, slightly produced; posterior angle acute; anterior margin emarginate behind head; disc subconvex; surface irregularly punctate; basal impression absent; pronotal length 1.0–1.2 mm; pronotal width 1.3–1.5 mm. Scutellum: pentagonal; impunctate. Elytron: lateral margin straight, smooth, narrowly margined; apex rounded; sutural angle without tooth; humerus rounded, slightly produced; slightly constricted behind humerus; subconvex; strongly punctate-striate, puncture rows converge and unite apically; declivity beginning just behind humerus at puncture row 7 not edged with faint carina; elytral length 3.4–3.8 mm; elytral width 1.7–1.9 mm. Venter: pro-, meso-, and metasterna impunctate medially, punctate laterally; abdominal sterna punctate, each puncture with pale seta; suture between sterna 1 and 2 complete; last sternite with apical margin truncate laterally, rounded medially in male; truncate laterally, bisinuate medially in female. Leg: slender; punctate, each puncture with pale seta; tibia with fringe of setae on inner margin of apex. Total length: 4.9–5.4 mm.

##### Diagnosis.

This species is similar to *Cephaloleia balyi*, *Cephaloleia deficiens*, *Cephaloleia discoidalis*, *Cephaloleia dorsalis*, and *Cephaloleia suturalis*. It can be distinguished by the yellow pronotum and by antennomere 1 being incrassate and longer than 3.

##### Distribution.

Argentina, Brazil (Santa Catharina, São Paulo).

##### Type material examined.

Lectotype male: Brazil, S. Catharina, Nova Teutonia, Plaumann [printed label]/ Holotyp [red printed label]/ Cephalolia linkei Uh., Det. E. Uhmann (DEI).

##### Specimens examined.

**ARGENTINA:** Misiones- Guarani Soberbio, October 1947 (USNM); Sta. Maria, Ocotober 1947 (USNM). **Brazil:** Pernambuco: Serra de Communaty, Pernambuco, 3 December 1893 (USNM). Santa Catharina- Nova Teutonia, 4 December 1935, 26 October 1936, 3 November 1936 (DEI), 5 January 1937, 24 April 1938, 7 October 1956, June 1968, November 1976 (USNM), no date (AMNH), November 1976, December 1976 (EGRC). Total: 65.

#### Cephaloleia lojaensis

Taxon classificationAnimaliaColeopteraChrysomelidae

Pic, 1931

http://species-id.net/wiki/Cephaloleia_lojaensis

Fig. 178

Cephalolia lojaensis Pic 1931: 34.
Cephaloleia lojaensis Pic. Uhmann 1957b: 21 (catalog); Descarpentries and Villiers 1959a: 139 (types).

##### Description.

Elongate; subparallel; subdepressed; shining; yellowish-red; eyes and antennae black. Head: vertex sparsely, irregularly punctate, medial sulcus absent; frons not projecting; slightly depressed between eyes. Antenna: reaches beyond humerus; robust; antennomere 1 incrassate, longest; 2, 4–10 transverse, subequal in length, each ½ length of 1; 3 cylindrical, elongate, longer than 2, ¾ length 1; 11 2× 10, rounded at apex; 1–2 punctate with scattered setae; 3–11 setose. Pronotum: quadrate; lateral margin straight for basal 4/5 then rounding to anterior angle, canaliculate; anterior angle rounded, prominent; posterior angle acute; anterior margin emarginate behind head; disc flattened; surface irregularly punctate, punctures widely separated; transverse basal impression present medially; pronotal length 1.4–1.5 mm; pronotal width 1.9–2.0 mm. Scutellum: elongate triangular; impunctate. Elytron: lateral margin straight, smooth, moderately margined; apex rounded; sutural angle without tooth; humerus rounded, slightly projecting; slightly constricted behind humerus; shallowly punctate-striate, punctures confused apically; elytral length 5.0–5.1 mm; elytral width 2.2–2.3 mm. Venter: pro-, meso-, and metasterna impunctate; abdominal sterna punctate, each puncture with pale seta; suture between sterna 1 and 2 complete; last sternite with apical margin truncate medially in male. Leg: robust, sparsely punctate; tibia with fringe of setae on inner margin of apex. Total length: 6.9–7.0 mm.

##### Diagnosis.

This species is similar to *Cephaloleia chimboana* and *Cephaloleia nigriceps*. It can be distinguished by the lack of a transverse basal impression on the pronotum and by the distinct elytral punctures which continue to the apex.

##### Distribution.

Ecuador.

##### Type material examined.

Holotype: Equateur, Loja [handwritten label]/ *Cephalolia lojaensis* sp. n. [handwritten label]/ Type [red printed label]/ Museum Paris Coll. M. Pic [blue printed label]/ Type [red printed label]/ Holotype [red printed label]/ MNHN EC 2639 [printed label] (MNHN).

##### Specimens examined.

**Ecuador:** Napo- Lago Agrio (18 km E), 30 August 1975 (USNM). Total: 1.

#### Cephaloleia luctuosa

Taxon classificationAnimaliaColeopteraChrysomelidae

Guérin-Méneville, 1844

http://species-id.net/wiki/Cephaloleia_luctuosa

Fig. 179

Cephaloleia luctuosa Guérin–Méneville 1844: 282. Baly 1885: 55; Blackwelder 1946: 719 (catalog); Papp 1953: 18 (catalog); Uhmann 1957a: 21 (catalog), 1966: 269 (noted); Staines 1996: 42 (Central America species), 1999: 241 (mimicry); McKenna and Farrell 2005: 119 (phylogeny), 2006: 10949 (phylogeny).
Cephalolia luctuosa Guérin-Méneville. Baly 1858: 55 (redescription); Gemminger and Harold 1876: 3601 (catalog); Donckier 1899: 550 (catalog); Weise 1910: 86 (noted), 1911a: 8 (catalog), 1911b: 10 (catalog), 1913: 101 (noted); Uhmann 1932b: 261 (museum list), 1936a: 111 (noted), 1936b: 484 (key); Bryant 1942: 205 (faunal list).

##### Description.

Elongate; subparallel; subdepressed; head (except reddish-yellow frons) and scutellum black; antennae entirely black or antennomeres 8–11 yellow; pronotum reddish-yellow with black quadrangular macula medially of anterior margin extending towards base; elytra black with yellow vitta from puncture rows 4–9, not reaching apex, lateral margin usually dark, sometimes pale; venter pro-, meso-, and metasterna yellow medially, black laterally, abdominal sternite 1 yellow medially, black laterally; 2–4 black except pale marginal vitta; 5 entirely black; leg femur yellow, tibia darker. Head: vertex punctate, not rugose; Y-shaped medial sulcus present; frons punctate, not projecting; not depressed between eyes. Antenna: reaches to humerus; slender; antennomere 1 elongate, with tuft of setae at apex; 2 ⅓ length of 1, with sharp inner angle; 3 2× length of 2, triangular; 4–10 elongate, decreasing in length; 11 2× length of 10, pointed at apex; 2 punctate with scattered setae; 1, 3–11 setose. Pronotum: subquadrate; lateral margin straight then rounding to anterior angle, margined; anterior angle angulate, slightly produced; posterior angle acute; anterior margin weakly emarginate behind head; disc subconvex; surface punctate basally; basal impression absent; pronotal length 1.0–1.3 mm; pronotal width 1.4–1.6 mm. Scutellum: broadly triangular; impunctate. Elytron: lateral margin straight, smooth, narrowly margined; apex rounded; sutural angle without tooth; humerus rounded, slightly produced; slightly constricted behind humerus; shallowly punctate-striate; declivity beginning just behind humerus at puncture row 7 not edged with faint carina; elytral length 4.6–5.3 mm; elytral width 1.9–2.1 mm. Venter: pro-, meso-, and metasterna impunctate medially, punctate laterally; abdominal sterna punctate, each puncture with pale seta; suture between sterna 1 and 2 complete; last sternite with apical margin sinuate medially in male, truncate in female. Leg: slender, sparsely punctate; tibia with tuft of setae at apex. Total length: 6.0–7.0 mm.

**Figures 179–187.**
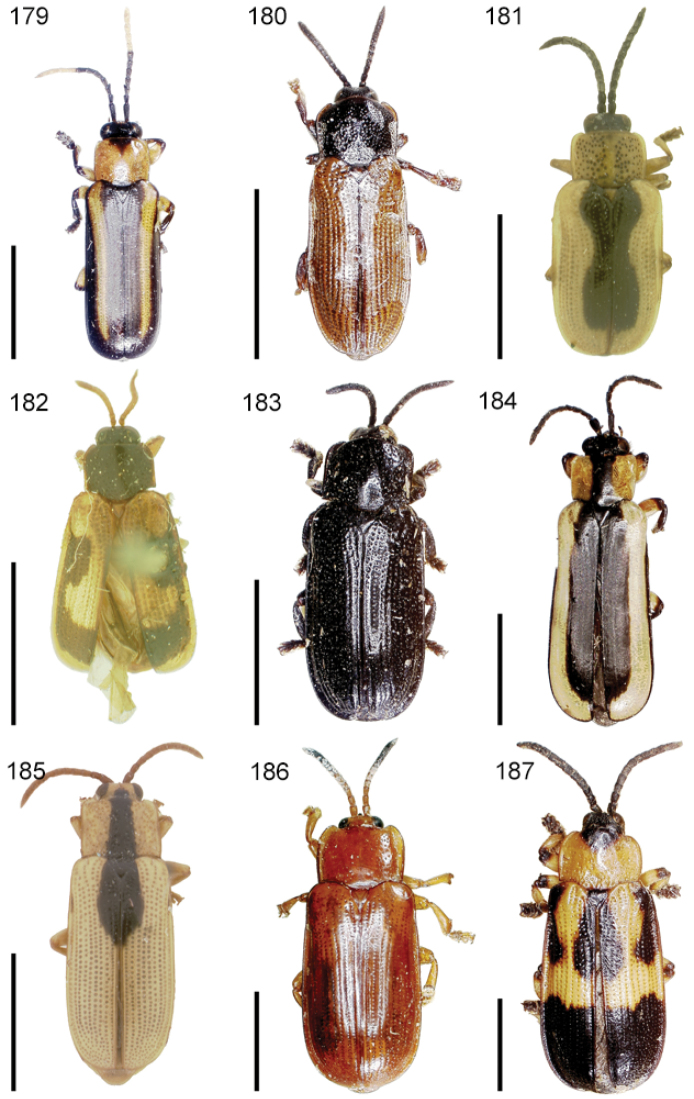
Habitus. **179** *Cephaloleia luctuosa* **180** *Cephaloleia luridipennis* **181** *Cephaloleia lydiae* **182** *Cephaloleia maculipennis* **183** *Cephaloleia marantae* **184** *Cephaloleia marginella* **185** *Cephaloleia marshalli* **186** *Cephaloleia mauliki* **187** *Cephaloleia maxima*. Scale bars equal 3 mm.

##### Diagnosis.

This species is similar to *Cephaloleia bella*, *Cephaloleia championi*, and *Cephaloleia vicina*. It can be distinguished by the vertex of the head not being depressed between the eyes, by the suture between abdominal sterna 1 and 2 being complete, and by antennomeres 2 and 3 being triangular.

##### Distribution.

Colombia, Panama.

##### Type material.

Type: Colombia, Santa Fe de Bogata (depository unknown, not seen).

##### Specimens examined.

**Colombia:** Meta- road to Bogota- Villavicencio, 1500 m, 29 June 1965 (AMNH). **PANAMA:** Bocas de Toro- Almirante, S 4 38 (USNM), 22 March 1959 (FMNH); 6 km N Punta Peña, 27 May 1993, 28 May 1993 (CDFA, AJGC); 2.3 mi. N Continental Divide, Reserva Fortuna, 26 May 1993 (AJGC), 28 May 1993 (CDFA). Canal Zone- 22 August 1970 (USNM). Chiriquí- 11.2 km E Chiriquí, 30 May 1993 (CDFA, AJGC); Repr. La Fortuna, 17–21 September 1976, 3200' (EGRC); Reserva Fortuna, Continental Divide Trail, 25 May 1993, 26 May 1993, 29 May 1993 (CDFA, EGRC). Coclé- Cerro Gaital, 10–12 June 1985 (EGRC). Colón- Achiote Road, 10 km SW Gatun, 12 June 1976 (USNM, EGRC); El Valle, alt 2400–2600 ft, 21 February 1959, 22 February 1959, 23 February 1959 (FMNH); vic. Fort Sherman, 15–16 February 1999 (USNM); Gamboa, 22 June 1976 (EGRC); 5 mi NW Gamboa, 27 April 1974 (EGRC); Pipeline road, 23 May 1993 (AJGC, CDFA); Pipeline rd. nr. Gamboa, 1 July 1976 (EGRC); Paraiso, 08 January 1911, 17 January 1911, 26 January 1911, 5 February 1911, 5 March 1911, 26 March 1911 (USNM); Madden Rd., 27 February 1959 (FSCA); Porto Bello, 23 February, 19 February 1911, 26 February, 27 February 1911, 2 March 1911 (USNM). Panamá- Alajuela, 5 April 1911, 18 April 1911 (USNM); Arraiján (USNM); Cerro Campana, 850 m, 17 February 1959 (FMNH), 11–15 May 1985 (EGRC), 17 May 1993 (CDFA); nr. Chepo, 3 April 1971 (EGRC); Corazal, 12 January 1911 (USNM); Cristobal, 9 February 1959 (FMNH); Fort Kobbe, 6 June 1976, 8 June 1976, 15 June 1976, 20 June 1976 (EGRC); Madden Forest, Mi 2.5, 2 August 1970 (CMNC); Ft. Howard, 24 October 1973 (FSCA); Madden Forest, 9 January 1971, 6 March 1971, 27 March 1971, 27 May 1971, 2 November 1973, 8 August 1977 (EGRC); 9 km N El Llano, 18 May 1993 (CDFA); Fort Kobbe, 22 May 1993 (AJGC, CDFA); Old Gamboa Road, 4 June 1993 (AJGC, CDFA); Las Cumbres, 8 January 1959 (FMNH); La Pita Signal Station rd., 8 June 1976, 16 June 1980 (EGRC); Panama Road leading to La Pita signal station, 2 May 1971 (EGRC); Reserva Sobrina, Powerline Road, 29 October 1972 (FSCA); Summit, September 1946 (USNM). San Blas- Salud, 30 December 1972 (EGRC). Total: 396.

#### Cephaloleia luridipennis

Taxon classificationAnimaliaColeopteraChrysomelidae

(Weise, 1905b)

http://species-id.net/wiki/Cephaloleia_luridipennis

Fig. 180

Stenispa luridipennis Weise 1905b: 52. Weise 1911a: 10 (catalog), 1911b: 14 (catalog).
Cephalolia luridipennis (Weise). Uhmann 1936b: 117 (transfer).
Cephaloleia luridipennis (Weise). Uhmann 1957b: 21 (catalog).

##### Description.

Elongate; subparallel; subdepressed; head dark with metallic sheen, mouthparts reddish-brown; pronotum black with reddish lateral margins; scutellum black with reddish middle; elytra yellowish-brown with suture and apex darker; venter black; legs black with base of femur and apex of tibia reddish. Head: vertex distinctly, finely punctate, medial sulcus absent; frons not projecting; depressed between eyes. Antenna: reaches to humerus; slender; antennomeres 1–10 cylindrical; 1 subequal in length to 2, cylindrical; 2 ¾ length of 3; 3 cylindrical, longer than 1, longest; 4–10 subequal in length, cylindrical, each shorter than 2; 11 2× length of 10, pointed at apex; 1–3 punctate with scattered setae; 4–11 setose. Pronotum: transverse; lateral margin straight then rounding to anterior angle, margined; anterior angle rounded, not produced; posterior angle acute; anterior margin emarginate behind head; disc convex; surface finely punctate with medial longitudinal impunctate line; transverse basal impression present medially; pronotal length 1.1–1.2 mm; pronotal width 1.4–1.5 mm. Scutellum: pentagonal; impunctate. Elytron: lateral margin straight, smooth, rounded on apical ⅓; apex weakly rounded; sutural angle without tooth; humerus rounded, not produced; slightly constricted behind humerus; finely punctate-striate, punctures larger in rows 6–9, rows 5–6 obsolete before apex, rows converge and unite apically; elytral length 3.4–3.5 mm; elytral width 1.5–1.6 mm. Venter: pro-, meso-, and metasterna punctate; abdominal sterna punctate, each puncture with pale seta; suture between sterna 1 and 2 complete; last sternite with apical margin emarginate medially in male, rounded, entire in female. Leg: robust; femur rugose; tibia deeply incised apically with fringe of setae apically. Total length: 4.7–5.0 mm.

##### Diagnosis.

This species is similar to *Cephaloleia clarkella*. It can be distinguished by the pronotum with a transverse basal impression and by antennomere 1 being subequal in length to 2.

##### Host plant.

According to label data, adults have been collected on Cyperaceae.

##### Distribution.

Brazil (Bahia, Rondonia), Paraguay, Peru, Venezuela.

##### Type material.

Type: Brazil, Ceara (ZMHB, not seen).

##### Specimens examined.

**Brazil:** ?- Ceara (ZMHB). Bahia- no further data (USNM). Rondonia- 62 km SW Ariquames Fazenda Rancho Grande, 12–22 November 1991 (CDFA). **Paraguay:** Cordillera- Inst. Agro. Nac. Caacupe, 17–20 January 1983 (EGRC). **PERU:** Oxapampa- Puerto Bermudes, Río Richia, 13–19 July 1920 (USNM). **Venezuela:** Aragua- Rancho Grande Biological Station, 1250 m, 14 May- 2 June 1998 (SEMC). Total: 6.

#### Cephaloleia lydiae

Taxon classificationAnimaliaColeopteraChrysomelidae

Uhmann, 1954

http://species-id.net/wiki/Cephaloleia_lydiae

Fig. 181

Cephaloleia lydiae Uhmann 1954: 497. Uhmann 1957b: 21 (catalog); Gaedike and Döbler 1971: 353 (types); Staines 1997b: 413 (Uhmann species list).

##### Description.

Ovate; subconvex; shining; yellowish-brown; head and antennae black; elytra with variable black markings; venter black. Head: vertex finely, densely punctate, medial carina present; frons not projecting; slightly depressed between eyes. Antenna: as long as head and pronotum combined; robust, filiform; antennomere 1 longest; 2–10 subequal in length, each ½ length of 1; 11 3× length of 10, acutely pointed at apex; 1–2 punctate with scattered setae; 3–11 setose. Pronotum: transverse; lateral margin sinuate then rounding to anterior angle, margined; anterior angle rounded, produced; posterior angle acute; anterior margin emarginate behind head; disc subconvex; surface irregularly punctate, medial longitudinal line impunctate; basal impression absent; pronotal length 1.3 mm; pronotal width 1.7 mm. Scutellum: pentagonal; impunctate. Elytron: lateral margin straight, smooth, weakly margined; apex weakly rounded; sutural angle without tooth; humerus rounded, not produced; slightly constricted behind humerus; moderately punctate-striate, punctures converge and unite apically; elytral length 3.9 mm; elytral width 2.2 mm. Venter: pro-, meso-, and metasterna rugose medially, punctate laterally; abdominal sterna punctate, each puncture with white seta; suture between sterna 1 and 2 complete; last sternite with apical margin rounded. Leg: slender, impunctate; tibia with fringe of setae on inner margin of apex. Total length: 5.5 mm.

##### Diagnosis.

This species is similar to *Cephaloleia fryella* and can be distinguished by the vertex of the head having a medial carina and by the sinuate lateral margins of the pronotum.

##### Distribution.

Brazil (Bahia).

##### Type material examined.

Holotype: Brazil, Bahia, Bondar [printed label]/ 2365 [handwritten label]/ Holotypus [red printed label]/ Cephaloleia lydiae Uh., Det. E. Uhmann (BMNH).

#### Cephaloleia maculipennis

Taxon classificationAnimaliaColeopteraChrysomelidae

Baly, 1858

http://species-id.net/wiki/Cephaloleia_maculipennis

Fig. 182

Cephalolia maculipennis Baly 1858: 58. Gemminger and Harold 1876: 3602 (catalog); Donckier 1899: 550 (catalog); Weise 1911a: 8 (catalog), 1911b: 12 (catalog).
Cephaloleia maculipennis Baly. Uhmann 1957b: 21 (catalog); Staines and Staines 1999: 524 (Baly species list).

##### Description.

Elongate; subparallel; subdepressed; head black, antennae yellow with apical two antennomeres darker; pronotum black with lateral margin yellow; elytra yellow with black curved transverse band near apex, a black submarginal vitta starting at base ending in a black transverse band near the middle; venter yellowish. Head: vertex impunctate, medial sulcus absent; frons not projecting; not depressed between eyes. Antenna: reaches to humerus; slender; antennomere 1 incrassate; 2 robust, ¾ length of 1; 3 elongate, longer than 2; 4–5 elongate, subequal in length, each shorter than 3; 6–10 transverse; 11 2× length of 10, rounded at apex; 1–9 punctate with scattered setae; 10–11 setose. Pronotum: transverse; lateral margin straight then rounding to anterior angle, narrowly margined; anterior angle slightly excavated; posterior angle acute; anterior margin weakly emarginate behind head; disc subconvex; surface coarsely, irregularly punctate; basal impression absent; pronotal length 0.9 mm; pronotal width 1.3 mm. Scutellum: pentagonal; impunctate. Elytron: lateral margin straight, smooth, narrowly margined; apex rounded; sutural angle without tooth; humerus rounded, slightly produced; slightly constricted behind humerus; moderately convex, flattened at suture; distinctly punctate-striate, punctures large; elytral length 3.2 mm; elytral width 1.6 mm. Venter: pro-, meso-, and metasterna impunctate; abdominal sterna sparsely punctate, each puncture with pale seta; suture between sterna 1 and 2 obsolete medially; last sternite with apical margin broadly emarginate, sinuate medially in male. Leg: slender, impunctate; tibia with fringe of setae on inner margin of apex. Total length: 4.5 mm.

##### Diagnosis.

This species is similar to *Cephaloleia tetraspilota*. It can be distinguished by the yellowish elytra and the impunctate vertex of the head.

##### Distribution.

Brazil.

##### Type material examined.

Holotype male: Brazil [handwritten label]/ Fry Coll. [printed label]/ Cephlalolia macuulipennis Baly, Brazil [blue handwritten label] (BMNH).

#### Cephaloleia marantae

Taxon classificationAnimaliaColeopteraChrysomelidae

Uhmann, 1957c

http://species-id.net/wiki/Cephaloleia_marantae

Fig. 183

Cephalolia funesta Weise 1906: 221 (homonym of *Cephaloleia funesta*Baly 1858). Weise 1911a: 8 (catalog), 1911b: 13 (catalog); Uhmann 1936b: 116 (noted), 1938b: 365 (faunal list), 1942b: 96 (pygidium), 1964a: 403 (catalog); Monrós and Viana 1947: 164 (Argentina species); Staines 1997b: 413 (Uhmann species list).
Cephaloleia marantae Uhmann 1957c: 365 (new name) Gaedike and Döbler 1971: 353 (types).

##### Description.

Narrow, elongate; subparallel; subconvex; black; shining. Head: vertex punctate, medial sulcus present; frons not projecting; depressed between eyes. Antenna: as long as head and pronotum combined; robust; antennomeres 1–2 robust, short, subequal in length; 3 elongate, longest; 4–10 transverse, subequal in length; 11 2× length of 10, pointed at apex; 1–2 punctate with scattered setae; 3–11 setose. Pronotum: transverse; lateral margin divergent from base then rounding to anterior angle, canaliculate; anterior angle rounded, slightly produced; posterior angle acute; anterior margin emarginate behind head; disc subconvex; surface coarsely, densely punctate, longitudinal band impunctate medially; transverse basal impression present medially; pronotal length 1.1–1.5 mm; pronotal width 1.4–1.8 mm. Scutellum: pentagonal; impunctate. Elytron: lateral margin straight, smooth, narrowly margined; apex rounded; sutural angle with small tooth; humerus rounded, not produced; slightly constricted behind humerus; finely punctate-striate, rows converge and unite apically; elytral length 3.9–4.4 mm; elytral width 1.8–2.2 mm. Venter: prosternum punctate; meso- and metasterna impunctate medially, punctate laterally; abdominal sterna punctate, each puncture with pale seta; suture between sterna 1 and 2 complete; last sternite with apical margin emarginate medially in male, truncate in female. Leg: slender, punctate, each puncture with pale seta; tibia with fringe of setae on inner margin of apex. Total length: 5.0–5.9 mm.

##### Diagnosis.

This species is similar to *Cephaloleia coroicoana*, *Cephaloleia deplanata*, *Cephaloleia fiebrigi*, and *Cephaloleia rufipes*. It can be distinguished by the basal impression on the pronotum, by the elytral puncutres being distinct basally and apically, and by the elytral puncture rows converging and uniting apically.

##### Distribution.

Argentina, Bolivia, Brazil (Amazonas, Paraná, Rio Grande do Sul, São Paulo, Santa Catharina), Paraguay.

##### Type material examined.

Syntype: Argentina, Gab. Misiones, 190.., aus Coll. Buck [green handwritten label]/ I. Weise det. [printed label]/ Type [printed salmon-colored label]/ Cephalolia funesta m [handwritten label] (DEI, 1).

##### Specimens examined.

**Argentina:** Chaco- Resistencia, October-November 1936 (DEI). Misiones- Loreto (USNM); Igazú, January 1944, July 1945 (USNM); Puerto Rico, August 1945 (USNM); San Ignacia, July 1945, October 1952 (USNM); Sta. Maria (USNM). **Bolivia:** Coroico (DEI). **Brazil:** Amazonas- Porto Algere, 17 October 1951 (USNM). Paraná- October 1942 (USNM); Punta Grosso, August 1942 (USNM). Río Grande do Sul- Parecy Novo, August 1932 (USNM). Santa Catharina- Nova Teutonica (DEI). São Paulo- São Jose dos Campos, 23–30 October 1997 (BYUC). **PARAGUAY:** ?- San Salvador (USNM). Carguazu- Paso Yobai, 28 November 1951 (USNM). Central- San Lorenzo, 8 September 1954 (USNM). Cordillera- Inst. Agro. Nac. Caacupe, 17–20 January 1983 (EGRC). Total: 83.

#### Cephaloleia marginella

Taxon classificationAnimaliaColeopteraChrysomelidae

Uhmann, 1930a

http://species-id.net/wiki/Cephaloleia_marginella

Fig. 184

Cephalolia marginella Uhmann 1930a: 222. Uhmann 1936b: 485 (key).
Cephaloleia marginella Uhmann. Blackwelder 1946: 719 (catalog); Uhmann 1950b: 336 (type), 1957a: 21 (catalog); Papp 1953: 19 (catalog); Gaedike and Döbler 1971: 353 (types); Wilcox 1983: 137 (catalog); Staines 1996: 43 (Central America species), 1997: 413 (Uhmann species list), 1999: 242 (mimicry); Staines and Staines 1997: 13 (types); McKenna and Farrell 2005: 119 (phylogeny), 2006: 10949 (phylogeny).

##### Description.

Elongate; subparallel; large; subdepressed; head black except yellow frons; pronotum yellow except black longitudinal medial vitta from base to apex, narrowest at base; elytra yellow with two black vittae; venter with abdominal sterna 1 and 2 yellow medially; legs with tibiae and tarsi dark. Head: vertex finely, sparsely punctate, medial sulcus absent; frons not projecting; not depressed between eyes. Antenna: reaches to humerus; slender; male with antennomere 3 triangular, ½ length of 1, 4 weakly triangular; female with 3 triangular, ½ length of 1, 4 cylindrical, elongate; 1 elongate; 2 transverse, ¼ length of 1; 5–10 transverse, decreasing in length; 11 pointed at apex, 2× length of 10; 1–3 punctate with scattered setae; 4–11 setose. Pronotum: transverse; lateral margin straight then slightly narrowing to anterior angle, margined; anterior angle rounded; posterior angle angulate; anterior margin weakly emarginate behind head; disc flattened; surface impunctate; basal impression absent; pronotal length 1.0–1.3 mm; pronotal width 1.6–1.9 mm. Scutellum: elongate triangular; alutaceous. Elytron: lateral margin straight, smooth, margined; apex rounded; sutural angle without tooth; humerus rounded, not produced; slightly constricted behind humerus; shallowly punctate-striate, punctures obsolete at apex; row 1 weakly striate; declivity beginning just behind humerus at puncture row 7 not edged with faint carina; elytral length 5.0–5.7 mm; elytral width 2.1–2.4 mm. Venter: pro-, meso-, and metasterna extremely finely punctate, laterally each puncture with pale seta; abdominal sterna punctate, each puncture with pale seta; suture between sterna 1 and 2 complete; last sternite with apical margin emarginate in male, bisinuate in female. Leg: slender; impunctate; tibia with fringe of setae on inner margin of apex. Total length: 6.4–8.0 mm.

##### Diagnosis.

This species is similar to *Cephaloleia apicata*. It can be distinguished by the lack of a declivity on the elytra beginning at puncture row 7 and by the yellow vitta on the lateral margins of the elytra.

##### Host plant.

*Heliconia* sp. (Heliconiaceae) (Uhmann 1930a); adults have been collected on *Caesalpinia eriostachys* Benth. (Caesalpiniaceae) (Staines 1996).

##### Distribution.

Costa Rica, Panama.

##### Type material examined.

Lectotype: Costa Rica, F. Nevermann, 20-VI-26 [green label]/ La Palma, 1050 m, Hondura [reversed green label]/ Cephalolia marginella Uhm/ Cotype 54630 USNM [orange label] (USNM).

##### Specimens examined.

**COSTA RICA:** no further data (USNM). Alajuela- San Carlos, La Fortuna, P. N. Arenal, 500–600 m (INBIO); Estación Eladios, 700–800 m (INBIO); Río San Lorencito, 5 km N Colonia Palmareña, 900–1000 m (INBIO); E. B. San Ramón, R. B. San Ramón, 27 km N and 8 km S San Ramón, 8 July 2000 (SEMC, USNM); Upala, Sec San Ramón, 1.5 km NW Hacienda Nueva Zelandia, 600–700 m (INBIO). Cartago- Quebrada Segunda Ref. Nac. Fauna Tapantí, 1250 m, April 1992, May 1992, August 1992, December 1992 (INBIO); Ref. Nac. Fauna Silv. Tapantí, 1250 m, August 1991 (INBIO); Turrialba, 4–13 August 1970 (USNM); Río Grande de Orosí, La Catarata, 1500–1600 m (INBIO); Turrialba, Tayutic, 1100–1200 m (INBIO); Grano de Oro, Chirripo, 1100–1200 m (INBIO). Guanacaste- Comelco Property, 29 December 1972 (TAMU); Est. Pitilla, 700 m, 9 km S Sta Cecilia, P. N. Guanacaste, March 1991, November 1991, 24 August- 11 September 1992 (INBIO); Río San Lorenzo, 1050 m, Tierras Morenas, Z. P. Tenorio, April 1991, October 1991, January 1992, 23 March- 21 April 1992, April 1992, July 1992, September 1992, October 1992, December 1992, January 1993 (INBIO). Heredia- El Angel falls, Vara Blanca area, 21 June 1969 (USNM); La Palma, 20 June 1926 (USNM). Puntarenas- Est. La Casona, 1520 m, Res. Biol. Monteverde, September 1991 (INBIO); Monteverde Reserve, 3 June 1992 (CDFA), 18 August 1987 (CMNC), 17 February 1990 (USNM); Res. For. Monteverde, 17 February 1990 (INBIO, MUCR); Monteverde Cloud For. Res. 1450 m, 18–19 May 1985, 1300 m, 20 May 1985 (EMEC); San Luis, 1040 m, R. B. Monteverde, August 1992 (INBIO); Pque Nal Corcovado, Est Sirena, 0–100 m (INBIO); Guacimal, Finca Buen Amigo Monteverde, 4 km S Reserva, 1000–1100 m (INBIO); Reserva Bio Bosque Eterno, 1500–1600 m (INBIO). San José- San José, 24 June (USNM); Perez Zeledón, Santa Elena Las Nubes, 1200–1300 m (INBIO). **PANAMA:** Chiriquí- Repr. la Fortuna, 17–21 September 1976: 3200' (EGRC); Reserva Fortuna, Continental Divide Trail, 25 May 1993, 26 May 1993, 29 May 1993 (CDFA), 18 May 1993 (EGRC). Total: 193.

#### Cephaloleia marshalli

Taxon classificationAnimaliaColeopteraChrysomelidae

Uhmann, 1938a

http://species-id.net/wiki/Cephaloleia_marshalli

Fig. 185

Cephalolia marshalli Uhmann 1938a: 409.
Cephaloleia marshalli Uhmann. Uhmann 1957b: 21 (catalog); Gaedike and Döbler 1971: 353 (types); Staines 1997b: 413 (noted).

##### Description.

Elongate; subparallel; subdepressed; shining; yellow; antennae yellowish brown; vertex of head with oval black macula, pronotum with black broad longitudinal band, which begins at the apex and widens posteriorly, scutellum black, and basal ⅓ of elytra with black oval macula, elytra slightly darkened beneath humerus; pro-, meso-, and metasternum yellowish medially, dark laterally; sternites diffuse black. Head: vertex extremely finely punctate, with medial sulcus and callus between the antennal bases; frons not projecting; not depressed between eyes. Antenna: reaches to base of elytra; slender; antennomeres with whitish setae; 1 longest, incrassate; 2–6 cylindrical, elongate, decreasing in length; 7–10 transverse, subequal in length, each shorter than 6; 11 2× length of 10, rounded at apex; 1–3 punctate with scattered setae; 3–11 setose. Pronotum: transverse, nearly as wide as base of elytra; lateral margin straight then rounding to anterior angle, margined; anterior angle rounded, produced; posterior angle acute; anterior margin emarginate behind head; disc subconvex; surface with scattered strong and irregular punctures; basal impression absent; pronotal length 1.3–1.5 mm; pronotal width 1.7–1.9 mm. Scutellum: pentagonal, impunctate. Elytron: lateral margin straight, smooth, narrowly margined; apex rounded; sutural angle without tooth; humerus rounded, not produced; slightly constricted behind humerus; shallowly punctate-striate, rows converge and unite apically; interspaces not raised; each puncture darkened; elytral length 4.7–4.9 mm; elytral width 2.0–2.2 mm. Venter: prosternum impunctate; meso- and metasterna impunctate medially, punctate laterally; abdominal sterna punctate, each puncture with pale seta; suture between sterna 1 and 2 complete; last sternite with apical margin emarginate medially in male, rounded in female. Leg: slender; sparsely punctate, each puncture with pale seta; tibia with fringe of setae on inner margin of apex. Total length: 6.4–6.7 mm.

##### Diagnosis.

This species is similar to *Cephaloleia fryella* and *Cephaloleia lydiae*. It can be distinguished by the longitudinal black vitta on the pronotum which extends from the base to the apex.

##### Distribution.

Ecuador.

##### Type material examined.

Holotype: Ecuador, Cachabé, low c., XI.1896, Rosenberg [printed label]/ Holotypus [red printed label]/ Cephalolia marshalli Uh., Det. E. Uhmann (BMNH).

##### Specimens examined.

**Ecuador:** Imbabura- Cachabé (BMNH), January 1897, November 1896 (USNM). Total: 4.

#### Cephaloleia mauliki

Taxon classificationAnimaliaColeopteraChrysomelidae

Uhmann, 1930a

http://species-id.net/wiki/Cephaloleia_mauliki

Fig. 186

Cephalolia mauliki Uhmann 1930a: 215. Uhmann 1936b: 481 (key), 1942: 94 (noted).
Cephaloleia mauliki Uhmann. Maulik 1932: 295 (larva), 1933: 937 (noted), 1937: 132 (host plants); Uhmann 1936b: 481 (key), 1950a: 274 (sculpture), 1957a: 22 (catalog); Blackwelder 1946: 719 (catalog); Papp 1953: 19 (catalog); Lima 1955: 207 (faunal list); Gaedike and Döbler 1971: 353 (types); Wilcox 1983: 137 (catalog); Staines 1996: 44 (Central America species), 1997: 413 (Uhmann species list), 2004: 312 (host plants), 2011: 50 (faunal list); Staines and Staines 1997: 13 (types); Flowers and Hanson 2003: 51 (distribution); Sekerka et al. 2013: 304 (noted).

##### Description.

Elongate; subparallel; subdepressed; dark reddish brown; antennomeres 1–5 and eyes black. Head: vertex with fine, scattered punctures, medial carina present; frons not projecting; slightly depressed between eyes. Antenna: as long as the head and pronotum combined; robust; antennomere 1 as long as 2 and 3 combined; male with antennomeres 2 and 3 compressed, projecting inward; female with antennomeres 2 and 3 elongate; 4–10 transverse, decreasing in length; 11 elongate, pointed at apex, subequal in length to 1; 1–3 punctate with scattered setae; 4–11 setose. Pronotum: transverse; almost as wide as base of elytra; lateral margin straight for basal ¾ then rounding to anterior angle, canaliculate; anterior angle rounded, produced; posterior angle angulate; anterior margin emarginate behind head; disc subconvex; surface irregularly punctate; basal impression absent; pronotal length 1.6–1.9 mm; pronotal width 2.3–2.4 mm. Scutellum: pentagonal; apex acute; impunctate. Elytron: lateral margin straight, smooth, narrowly margined; apex rounded; sutural angle without tooth; humerus rounded, not produced; slightly constricted behind humerus; shallowly punctate-striate, punctures large, rows converge and unite irregularly at apex; elytral length 5.9–6.0 mm; elytral width 2.1–3.2 mm. Venter: prosternum impunctate; meso- and metasterna impunctate medially, punctate laterally; abdominal sterna punctate, each puncture with pale seta; suture between sterna 1 and 2 obsolete medially; last sternite with apical margin emarginate medially in male; truncate, lightly bisinuate in female. Leg: slender; profemur canaliculate on underside; femur punctate; tibia spoon-shaped at apex, with fringe of setae. Total length: 8.0–8.4mm.

##### Diagnosis.

This species is similar to *Cephaloleia placida*, *Cephaloleia simplex*, and *Cephaloleia sulciceps*. It can be distinguished by the suture between abdominal sterna 1 and 2 being obsolete medially and by antennomere 1 being subequal in length to 2 and 3 combined.

##### Host plant.

*Heliconia* sp. (Heliconiaceae) and *Calathea insignis* Petersen (Marantaceae) (Uhmann 1930; Maulik 1932, 1937); *Renealmia alpinia* (Rottb.) Maas, *Zingiber spectabile* Griff. (Zingiberaceae).

##### Immatures.

Color when dead creamy white with darker dorsal area along middle; surface shagreened, more so laterally. Total length: 8.5–9.5 mm; width 4.5–5.5 mm. Dorsum with anterior and lateral margins rugose; scattered setae especially along anterior margin; slightly raised medially. Venter with head somewhat elongate, epicranial halves not separated from each other; with six ocelli, four in line and two below; labrum large, covering mandibles; with setae; clypeus wider than labrum; mandible tridentate; maxillary palps short, stout; labial palps with one palpomere. Antenna long, antennomere 3 longest, with two unequal processes at apex; 2 shorter, wider, with four setae plus shorter setae at apex. Leg: with robust claw. (Maulik 1932)

##### Distribution.

Costa Rica, Panama.

##### Type material examined.

Paralectotypes: Costa Rica, F. Nevermann, 24.IV.26 [green label]/ Hamburg Farm, Reventazon, Ebene Limon [reversed green label]/ Allotype [red label]/ Cephalolia mauliki [female]/ Det. Uhmann/ Cotype No. 54629 USNM [orange label]. Three labeled paratypes- one with same data, 2 same except date-20.II.28, 17.II.24 (USNM, 4).

##### Specimens examined.

**COSTA RICA:** Heredia- Fca. La Selva, nr. Puerto Viejo, 22 June 1969, 7 August 1969 (USNM), 25–30 June 2001 (USNM), 16 June 2001 (SEMC). Limón- Amubri, 70 m, Talamanca, 16–31 August 1992 (INBIO); Cerro Tortuguero 0–120 m, P.N. Tortuguero, February 1992, January 1993 (INBIO); Est. Cuatro Esquinas, 0 m, P.N. Tortuguero, January 1992, November 1989, October 1989, 27 April- 9 May 1992, June 1992, December 1992, November 1992, December 1990 (INBIO); 7 mi N Guacimo, 22 February- 3 March 1988 (BYUC); Guápiles, 17 February 1924 (USNM); Hamburg Farm, Reventazón, Ebene Limón, 3 March 1928, 15 February 1924, 15 November 1923 (DEI); Est. Hitoy Cerere, 100 m, R. Cerere, Res. Biol. Hitoy Cerere, 30 June- 20 July 1992, June 1991, 4–20 December 1991 (INBIO); Manzanillo, 0–100 m, RNFS Gandoca y Manzanillo, 22 October- 11 November 1992, 7–19 August 1992, 7–14 August 1992, 4–12 December 1992, 9 September- 13 October 1992 (INBIO); Est. Miramar, 500 m, Res. Biol. Hitoy Cerere, September 1992 (INBIO); Salvadora Farm, Parismina Fluss, 24 February 1931, 26 October 1930 (DEI); Waldeck, 24 February 1928 (USNM); Valle La Setrella, 100–200 m (INBIO). Puntarenas- Barranca site, 10 km N. Puntarenas, 4 July 1969 (USNM); Fil de Cal, 6 km N Ciudad Neily, 610 m, 2 January 1990 (UMMZ); Coto Brus, Las Cruces Biological Station, 1200 m, 11 March 2012 (USNM); Golfto, 1 January 1990 (UMMZ); Est. Queb. Bonita, 50 m, Res. Biol. Carara, 17 March- 30 April, June 1992, 1–29 July 1992 (INBIO); Est. Sirena, P.N. Corcovado, 0–100 m, September 1991, January 1992, April 1990, November 1990, July 1991, June 1992, 21 March- 21 April 1992 (INBIO). **PANAMA:** Panamá- Barro Colorado Is., 16 January 1953, 22 May 1978 (USNM), 17 January 1952 (DEI), 14–18 June 2001 (SEMC). Total: 99.

#### Cephaloleia maxima

Taxon classificationAnimaliaColeopteraChrysomelidae

Uhmann, 1942b

http://species-id.net/wiki/Cephaloleia_maxima

Fig. 187

Cephalolia maxima Uhmann 1942b: 99.
Cephaloleia maxima Uhmann. Uhmann 1957b: 22 (catalog); Gaedike and Döbler 1971: 354 (types); Staines 1997b: 414 (Uhmann species list).
Uhmannispa maculata Monrós and Viana 1947: 172 (Holotype: Argentina, Chaco, Pto. Tizol, MACN, not seen). Uhmann 1957b: 22 (synonymy); Staines 1995b: 863 (Monrós species list); Staines and Staines 1997: 13 (types); Roig–Juñent 2004: 119 (faunal list); Bachmann and Cabrera 2010: 67 (types).

##### Description.

Elongate; slightly expanding apically; large; subconvex, shining, head, antennae, and scutellum black; pronotum orangy-yellow, with a triangular black macula as wide as the head and reaches more or less ⅓ its length; elytra orangy-yellow, with three black maculae- one humeral, which extends beyond basal ⅓, from puncture row 6 to the lateral margin; another postscutellar, irregular, extends beyond basal ½ from the suture to the interspace 3; the last, from anterior margin on the apical ⅓, irregular; venter orangish-yellow, with a black macula on the penultimate sternite, which does not reach the lateral margin; legs yellow, femora with a black apical ring, trochanters, tarsi, and base and apex of tibiae reddish-chestnut color. Head: vertex densely irregularly punctate, medial carina present; eye slightly convex, elongate, finely faceted; frons projecting; not depressed between eyes. Antenna: reaches to humerus; slender; antennomeres 1–2 cylindrical, elongate; 1 longer than 3; 2 slightly shorter than 3; 3 ¾ length of 1; 3–4 obconic, subequal in length; 5–10 transverse, subequal in length, shorter than 4; 11 pointed at apex, as long as 9–10 combined; 1–3 punctate with scattered setae; 4–11 setose. Pronotum: transverse; lateral margin slightly divergent from base to middle then rounding to anterior angle, narrowly margined; anterior angle rounded, not produced; posterior angle acute; anterior margin weakly emarginate behind head; disc transversely subconvex, depressed on each side; surface with coarse, fine, irregular punctures, more dense laterally and basally; basal impression present; pronotal length 1.9–2.1 mm; pronotal width 2.7–3.0 mm. Scutellum: pentagonal; punctate. Elytron: lateral margin somewhat divergent for basal ⅔, smooth, slightly laminate from humeral angle; apex rounded; sutural angle without tooth; humerus rounded, not produced; slightly constricted behind humerus; moderately punctate-striate, row 1 becomes furrow on posterior half, rows confused on humerus and apex; declivity beginning just behind humerus at puncture row 7 not edged with faint carina; elytral length 6.5–7.0 mm; elytral width 3.5–3.8 mm. Venter: pro-, meso-, and metasterna punctate; abdominal sterna punctate, each puncture with short yellow seta, punctures denser on apical sternites; suture between sterna 1 and 2 completely obsolete; last sternite with apical margin emarginate laterally, sinuate medially in male, female sinuate laterally and rounded medially. Leg: short; robust, punctate, each puncture with fine seta; tibia flattened, triangular, with fringe of setae on inner apical margin. Total length: 8.5–9.2 mm.

##### Diagnosis.

This species is similar to *Cephaloleia dilectans*, *Cephaloleia ornatula*, and *Cephaloleia strandi*. It can be distinguished by the pronotum with a basal impression and which is as wide as the base of the elytra.

##### Host plant.

*Ananas macrodentes* E. Morren (Bromeliaceae) (Monrós and Viana 1947).

##### Distribution.

Argentina.

##### Type material.

Holotype male: Argentina, Chaco, entre Villa Jalón y ‘la Popilar’, Picada Venturini, 11.IV.1936, Denier (not seen).

##### Specimens examined.

**Argentina:** Chaco- entre Villa Halon y “la Popular”, Picada Venturini, February-April 1936 (DEI); Pto. Tizol (MACN), 11 April 1936 (USNM). Total: 5.

#### Cephaloleia metallescens

Taxon classificationAnimaliaColeopteraChrysomelidae

Baly, 1885

http://species-id.net/wiki/Cephaloleia_metallescens

Fig. 188

Cephaloleia metallescens Baly 1885: 25. Weise 1911a: 8 (catalog), 1911b: 13 (catalog); Blackwelder 1946: 719 (catalog); Papp 1953: 19 (catalog); Uhmann 1957a: 22 (catalog); Wilcox 1983: 137 (catalog); Staines 1996: 45 (Central America species), 1999: 242 (mimicry), 2011: 50 (faunal list); Staines and Staines 1999: 524 (Baly species list); McKenna and Farrell 2005: 119 (phylogeny), 2006: 10949 (phylogeny).
Cephalolia metallescens Baly. Donckier 1899: 550 (catalog); Uhmann 1942: 94 (noted).
Cephaloleia metalescens Baly. Meskins et al. 2008: 163 (misspelling, host plants).

##### Description.

Broadly oblong-ovate; small; subdepressed; metallic blue; pronotum with lateral margin paler; venter and legs yellowish-red; antenna with antennomeres 1 and apical ½ of 11 reddish. Head: vertex punctate, medial carina present; frons not projecting; depressed between eyes. Antenna: ½ body length; slender; antennomeres 1–3 elongate; 1 and 3 subequal in length; 2 slightly shorter; 4–10 transverse, subequal in length; 11 2× length of 10, pointed at apex; 1–3 punctate with scattered setae; 4–11 setose. Pronotum: nearly twice as wide as long; lateral margin straight then rounding to anterior angle, canaliculate; anterior angle rounded, not produced; posterior angle acute; anterior margin emarginate behind head; disc transversely subconvex, depressed on each side; surface densely punctate; basal impression absent; pronotal length 0.6–0.7 mm; pronotal width 0.7–1.1 mm. Scutellum: pentagonal, impunctate. Elytron: lateral margin slightly curved, smooth, margined; apex rounded; sutural angle without tooth; humerus slightly produced, callus extends on base to scutellum; slightly constricted behind humerus; disc subconvex; moderately punctate-striate, interspaces sulcate; puncture rows obsolete at apex; elytral length 2.1–2.3 mm; elytral width 1.4–1.6 mm. Venter: pro- and mesosterna punctate; metasternum impunctate medially, punctate laterally; abdominal sterna punctate, each puncture with pale seta; suture between sterna 1 and 2 complete; last sternite with apical margin emarginate medially in male, rounded, entire in female. Leg: slender; punctate, each puncture with pale seta; tibia with fringe of setae on inner margin of apex. Total length: 3.1–3.3 mm.

**Figures 188–196.**
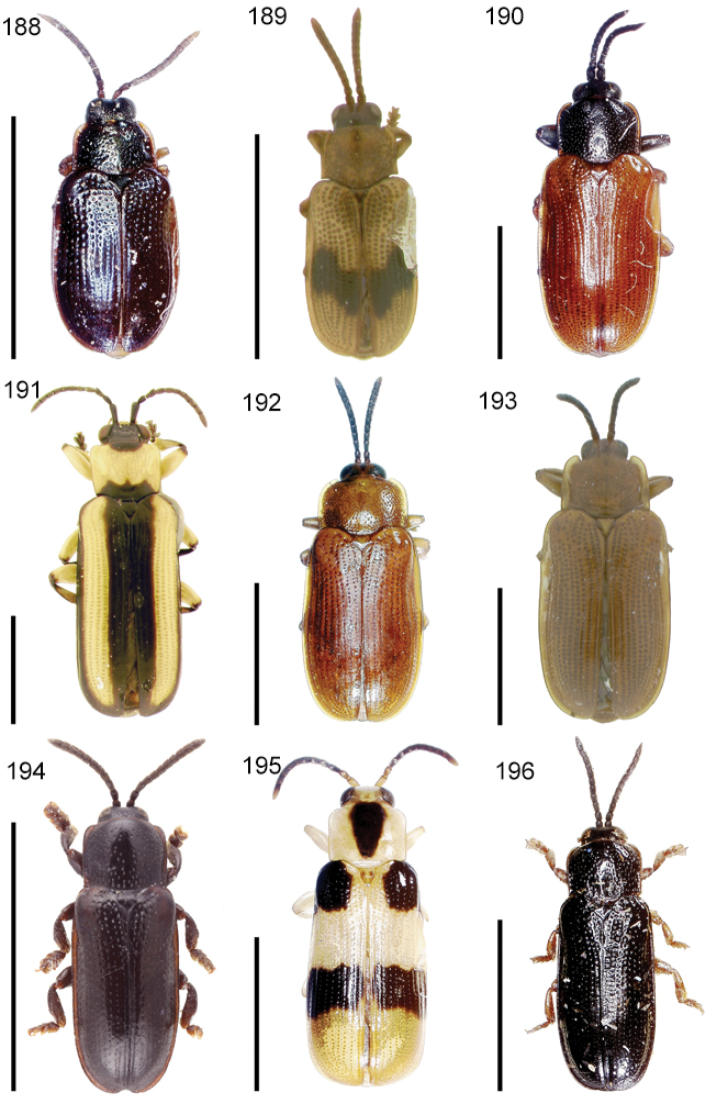
Habitus. **188** *Cephaloleia metallescens* **189** *Cephaloleia nana* sp. n. **190** *Cephaloleia neglecta* **191** *Cephaloleia nevermanni* **192** *Cephaloleia nigriceps* **193** *Cephaloleia nigricornis* **194** *Cephaloleia nigrithorax* **195** *Cephaloleia nigropicta* **196** *Cephaloleia nitida*. Scale bars equal 3 mm.

##### Diagnosis.

This is a very distinctive species with the flattened elongate body, the smooth rounded elytral apical margin, the smooth lateral margin of the pronotum, the lack of additional puncture rows on the elytra, the smooth lateral margins of the elytra, the lack of a declivity beginning at puncture row 7 on the elytra, and the unicolorous metallic blue dorsum.

##### Host plant.

*Bactris major* Jacq., *Chamaedorea wendlandiana* Hemsl. (Arecaceae) (Meskins et al. 2008).

##### Distribution.

Costa Rica, Guatemala, Nicaragua, Panama.

##### Type material examined.

Holotype: Type H. T. [white disk with red border]/ Guatemala, Vera Paz, San Juan, Champion/ B. C. A., Col. VI, 2. Cephaloleia metallescens, Baly/ Cephaloleia/ Cephalolia metallescens Baly, Guatemala [blue handwritten label] (BMNH).

##### Specimens examined.

**COSTA RICA:** Alajuela- Brasilia, 3 April 1988 (MUCR). Cartago- Turrialba (DEI, USNM); Turrialba, Tayutic, Grano de Oro, 1100–1200 m (INBIO). Guanacaste- Estación Pitila, 9 km S Santa Cecilia, 600–700 m (INBIO); La Cruz, 9 km S Santa Cecilia, 600–700 m (INBIO). Limón- Est. Cuatro Equinas, 0 m, P.N. Tortuguero November 1991 (INBIO); Pococí, 30 km N Cariari, 100–200 m (INBIO); Pque Nal Corcovado, Est Sirena, 0–100 m (INBIO). **NICARAGUA:** Río San Juan- Refugio Bartola, 16 km ESE El Castillo, 26 April 1993 (USNM). **PANAMA:** Chiriquí- Reserva Fortuna, Continental Divide Trail, 25 May 1993 (CDFA). Colón- 5 mi NW Gamboa, 29 September 1969 (CMNC); Paraiso, 26 March 1911, 18 April 1911, 6 April 1911, 4 May 1911, 20 May 1911, 17 April 1911, 28 March 1911, 21 April 1911, 14 April 1911 (USNM). Colón- Parq. Nac. Soberania, Pipeline road, 23 May 1993 (EGRC). Panamá- Barro Colorado Is., 7 January 1929 (USNM); Cerro Campana, 800 m, 3 November 1969 (EGRC); Cerro Jefe, 700 m, 19 June, 76 (EGRC); Coco Solito Hosp., 11 December 1971 (EGRC); Llano Carti Rd. at km 9, 18 May 1993 (EGRC); Madden Forest, 14 May 1978 (USNM); Nusagandi area, I. K. U. S. A. Igar, 20 May 1993 (EGRC). Total: 69.

#### Cephaloleia nana

Taxon classificationAnimaliaColeopteraChrysomelidae

Staines sp. n.

http://zoobank.org/55384CD4-B7AF-433E-9882-4B0B986A66A6

http://species-id.net/wiki/Cephaloleia_nana

Fig. 189

##### Description.

Small; elongate; subparallel; subconvex; head brownish-black; antennae, pronotum, and scutellum brown; elytra yellowish-brown with irregular black diagonal band from suture to puncture row 10; venter and legs yellowish-brown. Head: vertex punctate, medial sulcus absent; frons not projecting; not depressed between eyes. Antenna: reaches to humerus; slender; antennomere 1 incrassate, elongate; 2 subglobose, ¾ length of 1; 3 elongate, slightly longer than 2; 4–10 transverse, subequal in length; 11 2× length of 10, rounded at apex; 1–2 punctate with scattered setae; 3–11 setose. Pronotum: transverse; lateral margin straight then rounding to anterior angle, canaliculate; anterior angle rounded, slightly produced; posterior angle acute; anterior margin weakly emarginate behind head; disc subconvex; surface irregularly punctate, punctures more dense laterally; basal impression absent; pronotal length 0.7 mm; pronotal width 0.9 mm. Scutellum: pentagonal; impunctate. Elytron: lateral margin straight, smooth, margined from below humerus narrowing toward sutural angle; apex rounded, smooth; suture margined; sutural angle without tooth; humerus rounded, not produced; slightly constricted behind humerus; declivity beginning just behind humerus at puncture row 7 not edged with faint carina; moderately punctate-striate, rows converge and unite apically; elytral length 2.6 mm; elytral width 1.4 mm. Venter: pro- and metasterna impunctate medially, punctate laterally; mesosternum punctate; abdominal sterna punctate, each puncture with pale seta; suture between abdominal sterna 1 and 2 obsolete; apex of sternite 5 emarginate medially in male. Leg: slender; femur and tibia punctate, each puncture with pale seta; tibia with fringe of setae on inner margin of apex. Total length: 3.4 mm.

##### Etymology.

From nana (Greek and Latin) meaning little for the small size of this species. The name is feminine.

##### Diagnosis.

This species is one of the yellowish species with black transverse bands with pale lateral margins of the elytra. It can be distinguished from all other species in this color group by the vertex of the head lacking a medial fovea or sulcus, by the lateral margins of the pronotum being canaliculate, by antennomere 2 being subglobose rather than elongate, and by the declivity on the elytra beginning at puncture row 7.

##### Distribution.

Ecuador.

##### Type material.

Holotype male: Ecuador, Sucumbios, 9 km SE Lumbaqui, 650 m, 00°01.76'N, 077°17.06'W, VIII-7/8–1998, A. J. Gilbert/ Holotype *Cephaloleia nana* Staines, des. C. L. Staines 2012 [red label] (CASC); Paratypes (7) (each with Paratype *Cephaloleia nana* Staines, des. C. L. Staines 2012 [red label]): with same label data as holotype (AJGC, USNM); Ecuador, Orellana prov., EC Yasuni, Rio Tiputini, 00°40'16"S, 76°24'02"W, 200 m, D. W. Windsor lgt., 10.i.2005 [green printed label] (LSC, DWC); Ecuador, Sucumb., Shushufindi, 0°11.06/76°39.0W, 11, 12 August 1997, D. M. Windsor (BMNH, LSC, DWC).

#### Cephaloleia neglecta

Taxon classificationAnimaliaColeopteraChrysomelidae

Weise, 1910

http://species-id.net/wiki/Cephaloleia_neglecta

Fig. 190

Cephalolia neglecta Weise 1910: 91. Weise 1911a: 8 (catalog), 1911b: 12 (catalog); Uhmann 1936b: 114 (noted); Bryant 1942: 205 (faunal list).
Cephaloleia neglecta Weise. Uhmann 1957b: 22 (catalog); Seifert and Seifert 1979b: 51 (biology); Seifert 1982: 8 (biology); Jolivet and Verma 2002: 63 (noted); Jolivet 2003: 313 (noted); Staines 2004a: 312 (host plants); Chaboo 2007: 45 (noted); García–Robledo et al. 2010: 64 (noted).

##### Description.

Elongate; subparallel; subdepressed; yellowish; antennae, head, and pronotum (except reddish lateral margin) darker. Head: vertex finely, irregularly punctate, medial sulcus present; frons projecting; slightly depressed between eyes. Antenna: as long as head and pronotum combined; robust; antennomere 1 incrassate, elongate, 2 x length of 2; 2 transverse; 3–4 cylindrical, elongate; 3 ¾ length of 1; 4 shorter than 3; 5 transverse, shorter than 4; 5 transverse, shorter than 4; 6–10 transverse, subequal in length, each shorter than 5; 11 2× length of 10, pointed at apex; 1–4 punctate with scattered setae; 5–11 setose. Pronotum: transverse; lateral margin straight for basal ¾ then rounding to anterior angle, canaliculate; anterior angle rounded, not produced; posterior angle acute; anterior margin weakly emarginate behind head; disc subconvex; surface irregularly punctate except impunctate longitudinal medial line; transverse basal impression present medially; pronotal length 1.2–1.6 mm; pronotal width 1.6–2.0 mm. Scutellum: pentagonal; impunctate. Elytron: lateral margin straight, smooth, narrowly margined; apex rounded; sutural angle without tooth; humerus rounded, not produced; constricted behind humerus; flattened along suture; finely punctate-striate, punctures confused apically; elytral length 4.1–4.6 mm; elytral width 2.3–2.6 mm. Venter: pro-, meso-, and metasterna impunctate medially, punctate laterally; abdominal sterna punctate, each puncture with pale seta; suture between sterna 1 and 2 complete; last sternite with apical margin laterally emarginate and rounded medially in male, broadly emarginate laterally and weakly curved medially in female. Leg: slender; punctate; tibia with fringe of setae on inner margin of apex. Total length: 5.7–6.5 mm.

##### Diagnosis.

This species is similar to *Cephaloleia collaris* and *Cephaloleia flavipennis*. It can be distinguished by the transverse basal impression on the pronotum and the canalicultae lateral margins of the pronotum.

##### Host plant.

*Heliconia aurea* G. Rodriquez, *Heliconia bihai* L. (Seifert and Seifert 1979b). According to the label data, adults have been collected on *Heliconia stricta* Huber (Heliconiaceae).

##### Distribution.

Bolivia, Brazil (Rondonia), Colombia, Ecuador, Panama, Peru, Venezuela.

##### Type material.

Type: Venezuela, Merida (ZMHB, not seen).

##### Specimens examined.

**Bolivia:** Santa Cruz- Río Mamore, 2 km N mouth Río Chapare, 31 July 1965 (AMNH). **BRAZIL:** no further data (AMNH). Rondonia- Río Pardo, SW Ariquames, 5 November 1989 (USNM); 62 km SW Ariquames Fzda. Rancho Grande, 6–15 December 1990, 3 November 1989 (USNM), 12–22 November 1991 (CDFA). **COLOMBIA:** Amazonas- Leticia, 23 February- 2 March 1974 (USNM). **ECUADOR:** Napo- Jatun Sacha Biol. Stn., 23 km W Puerto Napo, 469 m, 19 September 1993 (SEMC); Puyo, 960 m, 1–8 October 1970 (USNM); 4.2 km S Cosanga, on Baeza-Tena Road, then 1.5 km W on pipeline Access road, 5–7 November 1999 (SEMC). Pichincha- 3.5 km N Pedro Vicente Maldonado, 29 March 1999 (SEMC). **PANAMÁ:** Coclé- El Valle, 14 June 1967 (USNM). **PERU:** Peru-Brazil frontier, August 27 (AMNH). Huanuco- Tingo Maria, 2200 ft., 21 May 1947 (AMNH). Loreto- Reserva Alpahuayo Mishana, 27 May 2005 (USNM); Aguaitia, 295 m, September 1946 (AMNH); Iquitos, 400 m, 27 May 1992 (SEMC). Madre de Dios- Cocha Cashi Biol. Stn., Mami Nat Pk, 350 m, 17 October 2000 (SEMC, USNM); CICRA Field Station, 272 m, 9 June 2011, 12 June 2011, 13 June 2001 (SEMC); Río Tambopata Res., 30 km SW Pto. Maldanodo, 22 October 1983, 8 November 1983, 9 March 1984, 10 September 1984 (USNM). Pasco- Oxapampa-Puzuzo Rd., 1300 m, 19 October 1999 (SEMC, USNM); San Juan, NE Villa Rica-Puerto Bermudez Rd., 16 October 1999 (SEMC). **Venezuela:** Aragua- Rancho Grande, Maracay, January 1954, 14 February 1976, 23 January 1975, 13 May 1998 (USNM), 13 May 1998, 14 May- 2 June 1998 (SEMC); Rancho Grande, Portachado Pass, 150 m, 27 February 1995, 8 March 1995 (SEMC). Merida- no further data (ZMHB). Total: 137.

#### Cephaloleia nevermanni

Taxon classificationAnimaliaColeopteraChrysomelidae

Uhmann, 1930a

http://species-id.net/wiki/Cephaloleia_nevermanni

Fig. 191

Cephalolia nevermanni Uhmann 1930a: 221. Uhmann 1936b: 485 (key).
Cephaloleia nevermanni Uhmann. Blackwelder 1946: 718 (catalog); Uhmann 1950b: 336 (type); Papp 1953: 19 (catalog); Uhmann 1957a: 22 (catalog); Descarpentries and Villiers 1959a: 139 (types); Gaedike and Döbler 1971: 355 (types); Wilcox 1983: 136 (catalog); Staines 1996: 45 (Central America species), 1997: 414 (Uhmann species list), 2004: 312 (host plants), 2011: 50 (faunal list); Staines and Staines 1997: 15 (types); McKenna and Farrell 2006: 10949 (phylogeny).

##### Description.

Elongate; large; subdepressed; yellow; head, antennae, and scutellum black, pronotum with black anterior and posterior maculae, elytra yellow with black sutural vitta to puncture row 2 and black lateral vitta to puncture row 8, sutural and lateral vittae unite near apex; venter with pro-, meso-, metasterna, and abdominal sterna yellow medially, black laterally; leg with femur yellow except for base and apex, tibia and tarsi darker. Head: vertex sparsely, finely punctate, with 3 carinae near base of antennae; frons punctate, not projecting; slightly depressed between eyes. Antenna: reaches to humerus; slender; antennomere 1 incrassate, with fringe of setae on lower apex, largest, longer than 2 and 3 combined; 2 transverse; 3 transverse and triangular in male, elongate in female; 4–10 elongate, subequal in length; 11 rounded at apex, ¾ length of 1; 1–3 punctate with scattered setae; 4–11 setose. Pronotum: transverse; lateral margin straight and divergent for basal ¾ then rounding to anterior angle, margined; anterior angle rounded, not produced; posterior angle angulate; anterior margin weakly emarginate behind head; disc flattened; surface sparsely, irregularly punctate, disc impunctate; basal impression absent; pronotal length 1.4–1.6 mm; pronotal width 1.7–1.9 mm. Scutellum: pentagonal; alutaceous. Elytron: lateral margin straight, smooth, margined; apex rounded; sutural angle without tooth; humerus rounded, not produced; slightly constricted behind humerus; declivity beginning just behind humerus at puncture row 7 not edged with faint carina; disc flattened; shallowly punctate-striate, punctures obsolete apically; elytral length 5.9 mm; elytral width 2.7 mm. Venter: pro-, meso-, and metasterna impunctate medially, rugose laterally; abdominal sterna punctate, each puncture with pale seta; suture between sterna 1 and 2 complete; last sternite with apical margin rounded in female, bisinuate in male. Leg: slender; apex of tibia with spoon-shaped depression with fringe of setae; mesolegs densely pubescent. Total length: 7.0–8.5 mm.

##### Diagnosis.

This species is similar to *Cephaloleia quadrilineata* and *Cephaloleia suaveola*. It can be distinguished by the elytra not expanding apically and by antennomere 1 being incrassate with 2 transverse.

##### Host plant.

*Calathea insignis* Hort. and Bull. (Uhmann 1930a); adults have been collected feeding on *Heliconia imbricata* (Kuntze) Baker (Heliconiaceae), *Calathea macrosepala* K. Schumann (Marantaceae) (Staines 1996).

##### Distribution.

Costa Rica, Panama.

##### Type material examined.

Lectotype: Costa Rica, F. Nevermann, 25-VI-22 [green label]/ Hamburg Farm, Reventazon, Ebene Limon [reversed green label]/ Cotype No. 54631 USNM [orange label]/ Cephalolia nevermanni Uh. Det. E. Uhmann- designated by Uhmann 1950 (USNM).

##### Specimens examined.

**COSTA RICA:** Alajuela- Río Frío, 16 July 1972 (FCSA). Cartago- Aquiares nr. Santa Cruz, 9 km NW Turrialba, 1500 m, 16 May 1985; Turrialba, 9 March 1967 (EGRC); ITICA at Turrialba, 13 March 1965 (BYUC). Heredia- Est. Biol. La Selva, 50 m, 30 March 1990 (INBIO), 31 March 1990 (USNM, MUCR); Finca La Selva, 21–30 July 1969, 23–30 May 2001, 11 March 2003 (USNM), 21 March 2003 (USNM); Finca La Selva, nr Puerto Viejo, 24 July 1969 (USNM). Limón- Sector Cerro Cocorí, Fca. de E. Rojas, 150 m, 28 May- 28 June 1992, December 1992, May 1993 (INBIO); Est. Hitoy Cerere, 100 m, R. Cerere, Res. Biol. Hitoy Cerere, 28–12 April 1992, 30 June- 20 July 1992, July 1992, November 1992, 15–27 February 1993 (INBIO); Hamburg Farm, Reventazón, Ebene Limón, 24 January 1924, 15 February 1924, 1 February 1932, 20 July 1931, 22 December 1933 (USNM), 15 November 1923, 15 February 1924, December 1929, 24 January 1932 (DEI); Limón, June 1972 (FSCA); Reventazón, 25 June 1922, 15 February 1924 (USNM); Valle La Estrella, 100–200 m (INBIO). Puntarenas- 5 km S. Rincón, 20 March 1973 (SEMC). San José- between Cerro de la Muerte and San Isidro, 18 July 1972 (FSCA); Pan American Hwy., km 80 S, 9.5 km SSW on San Gerado Rd., Catalata Trail, 23 July 2000 (SEMC, USNM). **PANAMA:** Bocas del Toro- 6 km N Punta Peña, 27 May 1993 (CDFA). Chiriquí- Reserva Fortuna, Continental Divide Trail, 26 May 1993 (CDFA). Coclé- El Valle (trail to Las Minas), 19 February 1959, 21 February 1959, 23 February 1959 (FMNH). Panamá- Cerro Campana, 17 February 1959 (FMNH), 11–15 May 1980 (EGRC), 17 May 1993 (CDFA); Fort Kobbe, 22 May 1993 (CDFA); Madden Forest, 9 January 1971, 27 March 1971, 25 June 1976 (EGRC). San Blas- Salud, 30 December 1972 (EGRC). Total: 155.

#### Cephaloleia nigriceps

Taxon classificationAnimaliaColeopteraChrysomelidae

Baly, 1869

http://species-id.net/wiki/Cephaloleia_nigriceps

Fig. 192

Cephalolia nigriceps Baly 1869: 370. Gemminger and Harold 1876: 3602 (catalog); Donckier 1899: 550 Weise 1911a: 8 (catalog), 1911b: 11 (catalog); Uhmann 1953d: 47 (faunal list).
Cephaloleia nigriceps Baly. Uhmann 1957b: 22 (catalog); Staines and Staines 1999: 524 (Baly species list).

##### Description.

Elongate; subparallel; subdepressed; reddish-yellow, head and antennae darker. Head: vertex finely punctate, medial sulcus absent; frons not projecting; slightly depressed between eyes. Antenna: ½ body length; slender; antennomeres 1–4 elongate; 1 subclavate; 2 shorter than 1, thicker than 3, subequal in length to 4; 3 longest, 2× longer than 2; 5–10 transverse, subequal in length; 11 2× length of 10, broadly rounded at apex; 1–2 punctate with scattered setae; 3–11 setose. Pronotum: transverse; lateral margin straight and diverging to beyond middle then rounding to anterior angle, slightly canaliculate; anterior angle obtuse, slightly projecting; posterior angle acute; anterior margin emarginate behind head; disc subconvex; surface with disc sparsely punctate, larger punctures laterally; transverse medial basal impression present; pronotal length 1.1–1.5 mm; pronotal width 1.9–2.1 mm. Scutellum: broadly pentagonal; impunctate. Elytron: lateral margin straight, smooth, narrowly margined; apex rounded; sutural angle without tooth; humerus rounded, not produced; constricted behind humerus; finely punctate-striate, punctures nearly obsolete at apex; with ill-defined longitudinal sulcus behind humerus; elytral length 4.0–4.4 mm; elytral width 2.1–2.3 mm. Venter: pro-, meso-, and metasterna punctate; abdominal sterna punctate, each puncture with pale seta; suture between sterna 1 and 2 complete; last sternite with apical margin quadrate-emarginate and sinuate medially in male, truncate in female. Leg: slender; punctate, each puncture with pale seta; tibia with fringe of setae on inner margin of apex. Total length: 5.3–5.8 mm.

##### Diagnosis.

This species is similar to *Cephaloleia chimboana* and *Cephaloleia lojaensis*. It can be distinguished by the pronotum without a transverse basal impression and by the distinct elytral punctures which becomes obsolete apically.

##### Distribution.

Brazil (Amazonas, Bahia, Rondonia), Peru.

##### Type material.

Holotype male: Peru [handwritten label]/ Cephalolia nigriceps Baly, Peru [blue printed label] (BMNH).

##### Specimens examined.

**Brazil:** Amazonas- Ega (USNM); approx 80 km N Manaus, 20 February 2000 (USNM). Bahia- Belmonte, 10 February 1914 (USNM). Rondonia- 62 km SW Ariquames, Fzda Rancho Grande, 12–21 November 1991 (CDFA), 15 November 1994 (BYUC), 6–15 December 1990 (USNM). Total: 21.

#### Cephaloleia nigricornis

Taxon classificationAnimaliaColeopteraChrysomelidae

(Fabricius, 1792)

http://species-id.net/wiki/Cephaloleia_nigricornis

Fig. 193

Hispa nigricornis Fabricius 1792: 73. Olivier 1792: 99 (noted), 1808: 773 (noted); Fabricius 1801: 64 (noted); Gyllenhal 1817: 5 (noted); Schönherr 1817: 7 (catalog); Zimsen 1964a: 127 (catalog).
Cephaloleia nigricornis (Fabricius). Chevrolat 1836: 390 (transfer); Orbigny 1845: 60 (noted); Baly 1858: 47 (redescription); Maulik 1916: 568 (museum list); Blackwelder 1946: 719 (catalog); Papp 1953: 19 (catalog); Uhmann 1957a: 22 (catalog), 1968: 248 (faunal list); Staines 1991(1992): 247 (nomenclature), 1996: 46 (Central America species), 2011: 50 (faunal list); McKenna and Farrell 2005: 119 (phylogeny).
Cephalolia nigricornis (Fabricius). Gemminger and Harold 1876: 3602 (catalog); Donckier 1899: 550 (catalog); Weise 1911a: 8 (catalog), 1911b: 11 (catalog); Uhmann 1936a: 113 (noted), 1942: 94 (noted), 1953: 47 (faunal list); Guérin 1953: 97 (faunal list).

##### Description.

Elongate; subparallel; subdepressed; reddish-brown, eyes and antennomeres 4–11 darker. Head: vertex impunctate, medial sulcus present; frons not projecting; not depressed between eyes. Antenna: reaches to humerus; slender; antennomere 1 elongate, thickened; 2 transverse; 3 elongate, ¾ length of 1; 4–10 transverse, subequal in length; 11 2× length of 10, pointed at apex; 1–3 punctate with scattered setae; 4–11 setose. Pronotum: transverse, lateral margin straight for basal ¾ then rounding to anterior angle, margined; anterior angle rounded, produced; posterior angle angulate; anterior margin emarginate behind head; disc subconvex; surface sparsely and irregularly punctate; basal impression absent; pronotal length 1.1–1.3 mm; pronotal width 1.7–1.9 mm. Scutellum: pentagonal, impunctate. Elytron: lateral margin straight, smooth, margined; apex rounded; sutural angle without tooth; humerus rounded, not projecting; slightly constricted behind humerus; shallowly punctate-striate, humerus almost impunctate; puncture rows confused at apex; elytral length 4.1–4.9 mm; elytral width 2.1–2.4 mm. Venter: prosternum impunctate medially, rugose laterally; meso- and metasterna entirely impunctate; abdominal sterna punctate, each puncture with pale seta; suture between sterna 1 and 2 obsolete medially; last sternite with apical margin weakly rounded in male; strongly rounded in female. Leg: slender; punctate; femur with pale seta in each puncture; tibia dentate at apex, fringe of setae at apex. Total length: 5.6–6.4 mm.

##### Diagnosis.

This species is similar to *Cephaloleia castanea*, *Cephaloleia delectabilis*, and *Cephaloleia opaca*. It can be distinguished by the impunctate vertex of the head which has a medial sulcus, by antennomere 3 being longer than 1, and by the elytral punctures being confused apically.

##### Host plant.

According to label data, adults have been collected on *Heliconia* sp. (Heliconiaceae).

##### Distribution.

Bolivia, Brazil, Colombia, Costa Rica, Ecuador, French Guiana, Guatemala, Honduras, Mexico, Panama, Peru, Venezuela.

##### Type material.

Type: Cap. Bon. (depository not known, not seen).

##### Specimens examined.

**Bolivia:** Cochabamba- 117 km E Yungas, Villa Tunari Rd., 10–12 February 1999 (SEMC). **BRAZIL:** Amazonas- Teffe (Ega), 1879 (USNM). **COLOMBIA:** Amazonas- nr. Letica, 29 August-5 September 1970 (USNM). **COSTA RICA:** Alajuela- N slope Volcán de Rincón, 2 km W Dos Ríos, 550 m, 22 May 1985 (EMEC); R. San Lorencito, 900 m, R. F. San Ramón, 5 km N de Colonia Palmañera, 13–18 June 1993 (INBIO); Upala, Sector San Ramó de Dos Ríos, 1. 5 km NW Hacienda Nueva Zelandia, 600–700 m (INBIO). Cartago- Quebrada Segunda, Ref. Nac. Fauna Silv. Tapantí, 1250 m, March 1992, April 1992, May 1992 (INBIO); Turrialba, 9 March 1967 (EGRC); Santa Teresita, Monumento Nacional Guayabo, 1100–1200 m (INBIO); Turrialba, Tayutic, Grano de Oro, Chirripo, 1100–1200 m (INBIO). Guanacaste- Est. Pitilla, 700 m, 9 km S Sta. Cecilia, P. N. Guanacaste, 24 August- 11 September 1992 (INBIO); Río San Lorenzo, 1050 m, Tierras Morenas, Z. P. Tenorio, April 1991, 10–20 February 1992, 23 March- 21 April 1992, December 1992, February 1993 (INBIO). Heredia- Est. El Ceibo, Braulio Carillo N. P., 400–600 m, March 1990 (INBIO); La Selva Biol. Sta, 2 km S. Pt. Viejo, 3–5 June 1984 (EGRC); Finca Naranjo Valenciana, 2 km sur Pueblo Nuevo, Sarapiquí, 90 m, 9–30 September 1992 (INBIO). Limón- Amburi, 70 m, Talamanca, 5–26 January 1993 (INBIO); Bananito, 20 April 1925 (USNM); Sector Cerro Cocorí, Fca. de E. Rojas, 150 m, August 1991, 31 January–21 February 1992, 26 March- 24 April 1992, April 1992, 28 May–17 June 1992, 26 June- 16 July 1992, 12–31 August 1992, October 1992, 9–30 November 1992, December 1992, January 1993, February 1993, March 1993, April 1993 (INBIO); Est. Cuatro Esquinas, 0 m, P. N. Tortuguero, June 1992, December 1990 (INBIO); Estrella Valley, 11 March 1964 (USNM); Hamburg Farm, Reventazón, Ebene Limón, 7 November 1924 (USNM); Manzanillo, 0–100 m, R.N.F.S. Gandoca y Manzanillo, 9 September- 13 October 1992, 22 October- 11 November 1992 (INBIO); Río Sardinas, 10 m, R.N.F.S. Barra del Colorado, September 1992 (INBIO); R. B. Hitoy Cerere, Sendero Toma de Agua, 0–100 m (INBIO); Valle La Estrella, 100–200 m (INBIO). Puntarenas- Est. La Casona, 1520 m, Res. Biol. Monteverde, September 1992 (INBIO); Est. Biol. Las Alturas, 1500m, Coto Brus, May 1992, November 1992 (INBIO); Palo Seco, 10 m, Pazifik, 31 December 1923 (USNM); Est. Queb. Bonita, 50 m, Res. Biol. Carara, 2–22 September 1992 (INBIO); Rancho Quemado, 200 m, Peninsula de Osa, July 1991 (INBIO); Sirena, Corcovado N. P., 0–100 m, November 1989, August 1991, 9–27 July 1992 (INBIO); Buenos Aires, Sector Altamira Biolley, 1700–1800 m (INBIO); Corredores, Ciudad Neily, 0–100 m (INBIO); F. Las Cruces, Ent. de los Alturas, Fca Fabio Sandoval, 1100–1200 m (INBIO); Golfito, F. Las Cruces, Fca Ilama, 1400–1500 m (INBIO); F. Las Cruces, Laguna Gamboa, 1400–1500 m (INBIO); Est. Boscosa, 0–100 m (INBIO); Bosque Eterno de los Niños, Sector Monteverde, Sendero El Camino, 1500–1600 m (INBIO). San José- Perez Zeledón, Santa Elena, Las Nubes, 1200–1300 m (INBIO); Finca El Gringo, Estación Las Nubes de Santa Elena, 1200–1300 m (INBIO). **ECUADOR:** Esmeraldas- 31.7 km NW Lita, 620 m, 23 August 1997 (USNM). Pinchica- Quito, 45 km NNW Macquipuanuna Station, 3–18 April 1996 (SEMC). **FRENCH GUIANA:** Hwy. N2 to Regina, 67 km S of Cayenne, 6 June 1986 (EGRC); Roura, 18.4 km SSE, 240 m, 24 May 1997, 29 May 1997 (SEMC, USNM), 23 May 1997, 25 May 1997, 30 May 1997 (SEMC); Saul, 7 km N Les Eaux Claires, 30 May 1997, 31 May 1997 (SEMC); Saul, 7 km N, 0.5 km ESE Les Eaux Claires, Mt. La Fumee, 4–8 June 1997 (SEMC). **GUATEMALA:** Zacapa- 3.5 km S.E. La Unión, 1500, 4 June 1991 (EGRC). **Honduras:** Altanica- Lanceville Bot. Gardens, Tela, 10 m, 23 June 1994 (SEMC). Cortez- Yola Lake, Deer Island, 670 m, 19 June 1994 (SEMC). Santa Barbara- La Fe, Finca La Roca, 5.3 km S Peña Blanca, 740 m, 19–21 June 1994 (SEMC). **Mexico:** Tabasco- 3 mi W Cardenas, 16 June 1966 (SEMC). **PANAMA:** Bocas del Toro- 6 km N Punta Peña, 27 May 1993 (CDFA). Chiriquí- Fortuna, 20 May 1978, 17 May 1978 (EGRC); La Fortuna, “Cont. Divide Trail”, 1150 m, 9 June 1995 (SEMC); Las Lagunas, 1360 m, 4 km W. Hato del Volcán, 22–23 May 1977 (CMNC); Reserva Fortuna, Continental Divide Trail, 25 May 1993 (CDFA); Hartmann's finca, St. Clara, 15–18 June 1985 (EGRC); Las Lagunas, 4 km W. Hato del Volcán, 1360 m, 24 May 1973 (EGRC); Santa Clara, 23–25 May 1980 (EGRC). Colón- Parque Nac. Soberania, Pipeline Rd., km 6.1, 40 m, 14 May 1995, 21 June 1995, km 2.0, 23 June 1995 (SEMC). Panamá- Old Gamboa Road, 4 June 1993 (CDFA); Old Plantation Road, 6.9 km S Gamboa, 80 m, 22 June 1995 (SEMC); Reserva Sobrina, Pipeline road, 23 May 1993, 16 May 1993 (CDFA). **Peru:** Huanuco- Cueva de las Pavas Canyon, 2600 feet. 8 km S. Tingo Maria, 28 April 1987 (ERGC). **VENEZUELA:** Aragua- Rancho Grande, 10 June 1983 (BYUC), 21 July 1990 (USNM), 8 May 1978 (EGRC). Total: 388.

#### Cephaloleia nigrithorax

Taxon classificationAnimaliaColeopteraChrysomelidae

Pic, 1930

http://species-id.net/wiki/Cephaloleia_nigrithorax

Fig. 194

Cephalolia nigrithorax Pic 1930: 3.
Cephaloleia nigrithorax Pic. Uhmann 1957b: 22 (catalog), 1964a: 404 (catalog); Descarpentries and Villiers 1959a: 139 (types).

##### Description.

Small; elongate; subparallel; subdepressed; shining; black with slightly metallic-blue sheen, elytra lightly margined in red; profemora reddish. Head: vertex sparsely, deeply punctate, medial sulcus absent; frons not projecting; depressed between eyes. Antenna: reaches to humerus; slender; antennomeres 1–3 elongate; 1 and 3 subequal in length; 2 slightly shorter than 1; 4–10 transverse, subequal in length; 11 2× length of 10, pointed at apex; 1–2 punctate with scattered setae; 3–11 setose. Pronotum: subquadrate; lateral margin straight for basal 4/5 then rounding to anterior angle, canaliculate; anterior angle rounded, not produced; posterior angle acute; anterior margin straight; disc subconvex; surface strongly, sparsely punctate, nearly impunctate medially and anteriorly; basal impression absent; pronotal length 0.6–0.8 mm; pronotal width 0.7–0.9 mm. Scutellum: pentagonal; finely punctate. Elytron: lateral margin straight, smooth, narrowly margined; apex rounded; sutural angle with minute tooth; humerus rounded, not produced; slightly constricted behind humerus; moderately punctate-striate; suture subsulcate; elytral length 2.1–2.3 mm; elytral width 0.9–1.0 mm. Venter: pro-, meso-, and metasterna punctate; abdominal sterna punctate, each puncture with pale seta; suture between sterna 1 and 2 complete; last sternite with apical margin emarginate medially in male, rounded, entire in female. Leg: short, robust; tibia punctate, each puncture with pale seta; with fringe of setae on inner margin of apex. Total length: 2.9–3.1 mm.

##### Diagnosis.

This species is similar to *Cephaloleia impressa*. It can be distinguished by the vertex of the head lacking a medial sulcus or carina.

##### Distribution.

Colombia, Ecuador, Venezuela.

##### Type material examined.

Holotype: El Naranjo, Venezuela [handwritten label]/ *nigrithorax* sp. n. [handwritten label]/ Type [handwritten label]/ Museum Paris Coll. M. Pic [blue printed label]/ *Cephaloleia nigrithorax* Pic [printed label]/ Holotype [red printed label]/ MNHN EC 2605 [printed label] (MNHN).

##### Specimens examined.

**COLOMBIA:** Meta- Restrepo, 2 October 1965 (USNM). **ECUADOR:** Napo- Limoncocha, 9 June 1997 (USNM). Pichincha- Tinalandia, 800 m, February 1983 (USNM); Sucumbios, Sacha Lodge, 3–13 July 1994 (SEMC). **Venezuela:** Aragua- Rancho Grande, 1100–1500 m, 6 May 1978 (EGRC). Total: 7.

#### Cephaloleia nigropicta

Taxon classificationAnimaliaColeopteraChrysomelidae

Baly, 1885

http://species-id.net/wiki/Cephaloleia_nigropicta

Fig. 195

Cephaloleia nigropicta Baly 1885: 10. Blackwelder 1946: 719 (catalog); Papp 1953: 19 (catalog); Uhmann 1957a: 22 (catalog); Wilcox 1983: 137 (catalog); Strong 1977b: 580 (host plants); Staines 1996: 47 (Central America species), 2004: 312 (host plants); Staines and Staines 1999: 524 (Baly species list); McKenna and Farrell 2005: 119 (phylogeny).
Cephalolia nigropicta Baly. Donckier 1899: 550 (catalog); Weise 1911a: 8 (catalog), 1911b: 12 (catalog).
Cephalileia nigripicta Baly. Strong 1982b: 1045 (lapsus calami, host plants).

##### Description.

Elongate; subparallel; subconvex; head yellow, eyes darker; antennae black, except antennomere 1 or 1 and 2 which are yellow; pronotum yellow with black triangular macula from anterior margin to near base; scutellum black; elytra yellow with black humeral maculae and black band just after midline from suture to lateral margin; legs yellow, apex of tibia and tarsi darker. Head: vertex impunctate, medial sulcus present; frons not projecting; slightly depressed between eyes. Antenna: less than ½ body length; slender; antennomere 1 elongate; 2 transverse; 3–4 elongate, shorter than 1; 5–10 transverse, subequal in length; 11 pointed at apex, subequal in length to 1; 1–2 punctate with scattered setae; 3–11 setose. Pronotum: subquadrate; lateral margin straight then rounding to anterior angle, margined; anterior angle rounded, not produced; posterior angle angulate; anterior margin straight; disc subconvex, impunctate; with slight prebasal depression laterally; pronotal length 1.0–1.2 mm; pronotal width 1.3–1.5 mm. Scutellum: pentagonal; micropunctate. Elytron: lateral margin slightly expanding apically, smooth, margined; apex rounded; sutural angle without tooth; humerus rounded, not produced; slightly constricted behind humerus; declivity beginning just behind humerus at puncture row 7 not edged with faint carina; slightly flattened along suture; moderately punctate-striate, interspaces finely sulcate; puncture rows converge and unite apically; humerus virtually impunctate; elytral length 4.3–4.7 mm; elytral width 1.8–2.0 mm. Venter: pro-, meso-, and metasterna impunctate; abdominal sterna punctate, each puncture with pale setae; suture between sterna 1 and 2 obsolete medially; last sternite with apical margin emarginate medially in male, rounded, entire in female. Leg: slender; tibia with fringe of setae on inner margin of apex. Total length: 5.8–6.3 mm.

##### Diagnosis.

This species is similar to *Cephaloleia reventazonica*. It can be distinguished by antennomere 3 being shorter than 1, by the humerus being nearly impunctate, and by the elytral puncture rows being regular to the apex.

##### Host plant.

Adults have been collected on *Heliconia* sp. (Strong 1977b); *Heliconia tortuosa* Griggs (Heliconiaceae), *Calathea crotalifera* S. Watson, *Pleiostachya leiostachya* (Donn. Sm.) Hammel (Marantaceae).

##### Distribution.

Costa Rica, Panama.

##### Type material examined.

Lectotype: Syntype [white disk with blue border]/ V. de Chiriqui, 25–4000 ft. Champion/ B.C.A. Col. VI, 2. Cephaloleia nigropicta Baly/ nigropicta/ Lectotype Cephaloleia nigropicta Baly des. C. L. Staines 1994 [red label] (BMNH).

##### Specimens examined.

**COSTA RICA:** Alajuela- Río San Lorencito, 900 m, R. F. San Ramón, 5 km N de Colonia Palmareña, 13–18 June 1993, March 1990 (INBIO); 20 km S Upala, 11–21 June 1991, 10–19 March 1991, 20 January- 12 February 1991, 24 June- 22 July 91 (BYUC). Guanacaste- 3 km SE R. Naranjo, 1–10 October 1992, 25–29 June 1992, 11–21 April 1992, 1–15 May 1992, 1–5 June 199-, 11–18 February 1993, 11–20 November 1992, 16–31 October 1992, 14–21 June 1993 (BYUC); Río San Lorenzo, 1050 m, Tierras Morenas, Z. P. Tenorio, February 1993, April 1992, 23 March- 21 April 1992, 10–20 February 1992 (INBIO). Heredia- Estación Biologica La Selva, 02 July 2001, 04 July 2001, 7 April 2003, 28 July 1989 (USNM). Puntarenas- Coto Brus, Las Cruces Biological Station, 10 March 2012 (USNM). **PANAMA:** no further data (USNM). Bocas del Toro- 6 km N Punta Peña, 27 May 1993 (CDFA). Chiriquí- Reserva Fortuna, Continental Divide Trail, 26 May 1993 (CDFA). Total: 47.

#### Cephaloleia nitida

Taxon classificationAnimaliaColeopteraChrysomelidae

Uhmann, 1930c

http://species-id.net/wiki/Cephaloleia_nitida

Fig. 196

Cephalolia nitida Uhmann 1930c: 36.
Cephaloleia nitida Uhmann. Uhmann 1942b: 97 (sculpture), 1948b: 13 (noted), 1957b: 22 (catalog), 1964b: 5 (faunal list); Gaedike and Döbler 1971: 355 (types); Staines 1997b: 414 (Uhmann species list).

##### Description.

Elongate; subparallel; subdepressed; shining; dark metallic blue-black; palps and legs reddish-yellow. Head: vertex finely punctate, medial sulcus absent; keel present between antennal bases; frons not projecting; not depressed between eyes Antenna: reaches beyond humerus; slender; antennomeres elongate; 1–2 subequal in length; 3 as long as 1 and 2 combined; 4–5 subequal in length, each ¾ length of 3; 6–10 subequal in length, each shorter than 5; 11 2× length of 10, broadly rounded at apex; 1–2 punctate with scattered setae; 3–11 setose. Pronotum: transverse; lateral margin straight, diverging for basal 4/5 then rounding to anterior angle, finely margined; anterior angle rounded, not produced; posterior angle acute; anterior margin straight; disc subconvex; surface strongly, sparsely punctate, medial line impunctate; transverse basal impression present medially; pronotal length 1.0–1.2 mm; pronotal width 1.1–1.3 mm. Scutellum: pentagonal; impunctate. Elytron: lateral margin straight, smooth, narrowly margined; apex rounded; sutural angle without tooth; slightly constricted behind humerus; humerus rounded, not produced; strongly punctate-striate; elytral length 3.2–3.4 mm; elytral width 1.3–1.5 mm. Venter: pro- and metasterna impunctate medially, punctate laterally; mesosternum punctate; abdominal sterna punctate, each puncture with pale seta; suture between sterna 1 and 2 complete; last sternite with apical margin weakly emarginate medially in male, rounded, entire in female. Leg: slender; punctate, each puncture with pale seta; tibia with fringe of setae on inner margin of apex. Total length: 4.3–4.7 mm.

##### Diagnosis.

This species is similar to *Cephaloleia emarginata*. It can be distinguished by the smooth apical margins of the elytra.

##### Distribution.

Argentina, Brazil (Santa Catharina, São Paulo).

##### Type material.

Holotype: Brazil, São Paulo, Mráz (DEI, not seen).

##### Specimens examined.

**ARGENTINA:** Misiones- Sta. Maria, October 1942 (USNM); Guarani Soberbio, October 1947 (USNM). **Brazil:** Santa Catharina- Nova Teutonia, 25 January 1938, 14 November 1938, October 1968, October 1952 (USNM), 24 December 1938 (AMNH). São Paulo- no further data (DEI, MNHN). Total: 9.

#### Cephaloleia nubila

Taxon classificationAnimaliaColeopteraChrysomelidae

Weise, 1905b

http://species-id.net/wiki/Cephaloleia_nubila

Fig. 197

Cephalolia nubila Weise 1905b: 55. Weise 1911a: 8 (catalog), 1911b: 10 (catalog); Uhmann 1936b: 110 (noted), 1936f: 484 (key), 1938a: 407 (noted).
Cephaloleia nubila Weise. Uhmann 1957b: 22 (catalog); Descarpentries and Villiers 1959a: 139 (types).

##### Description.

Elongate; subparallel; subdepressed; black with submedial pronotal macula, and elytra with wide submedial transverse vitta yellowish; antennae dark, last three antennomeres yellowish; head black, reddish between antennae; scutellum black; pro-, meso-, and metasterna yellowish medially, black laterally; abdominal sterna black with yellowish lateral margin; legs with femur yellow basally, dark apically, tibiae and tarsi dark. Head: vertex finely punctate, faint medial sulcus present; frons not projecting; not depressed between eyes. Antenna: reaches to humerus; slender; antennomere 1 incrassate, elongate; 2 subglobose, ⅓ length of 1; 3 weakly triangular in male, cylindrical in female, 2× length of 2; 4–10 elongate, cylindrical, subequal in length, each 2× length of 2; 11 2½x length of 10, longest; 1–2 punctate with scattered setae; 3–11 setose. Pronotum: subquadrate; lateral margin straight then rounding to anterior angle, canaliculate; anterior angle rounded, not produced; posterior angle acute; anterior margin curved anteriorly; disc subconvex; surface finely punctate; transverse basal impression present medially; pronotal length 1.2–1.3 mm; pronotal width 1.4–1.5 mm. Scutellum: pentagonal; punctate. Elytron: lateral margin straight, smooth, narrowly margined; apex rounded; sutural angle without tooth; humerus rounded, not produced; slightly constricted behind humerus; weakly punctate-striate, punctures obsolete apically; elytral length 5.1–5.3 mm; elytral width 2.0–2.1 mm. Venter: pro-, meso-, and metasterna impunctate; abdominal sterna punctate, each with white seta; suture between sterna 1 and 2 complete; last sternite with apical margin rounded. Leg: slender; punctate; tibia with fringe of setae on inner margin of apex. Total length: 6.5–7.0 mm.

**Figures 197–205.**
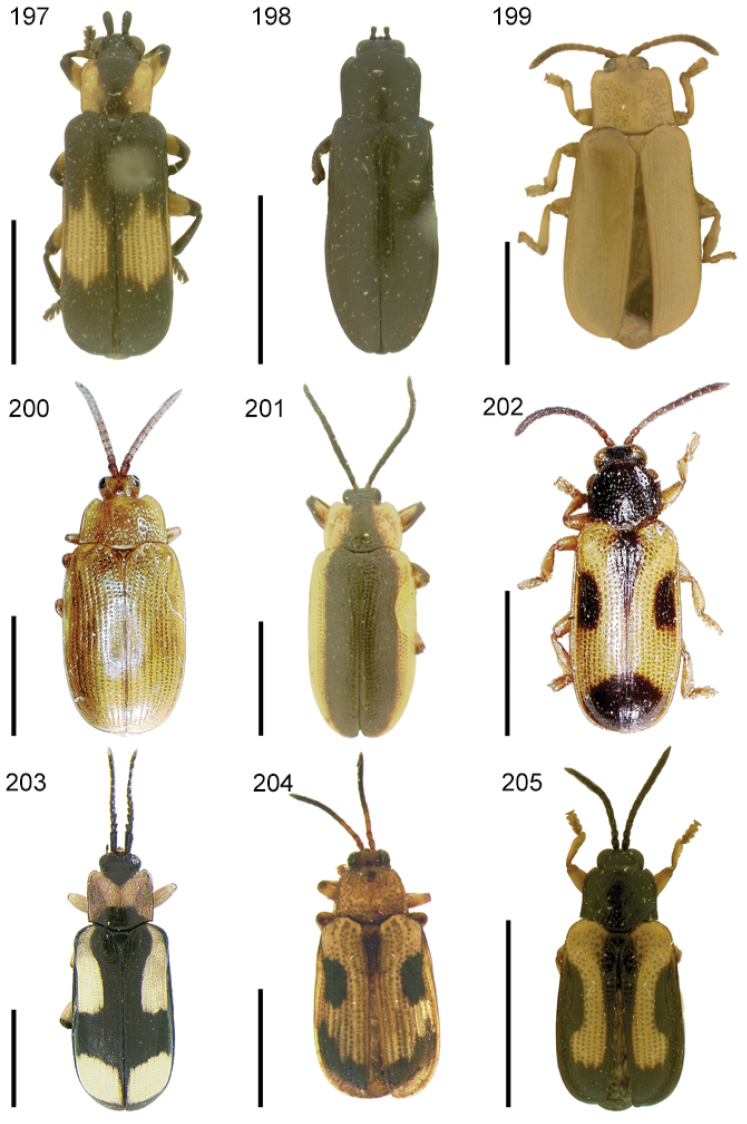
Habitus. **197** *Cephaloleia nubila* **198** *Cephaloleia obsoleta* **199** *Cephaloleia ochra* sp. n. **200** *Cephaloleia opaca* **201** *Cephaloleia orchideviora* **202** *Cephaloleia ornata* **203** *Cephaloleia ornatrix* **204** *Cephaloleia ornatula* **205** *Cephaloleia parenthesis*. Scale bars equal 3 mm.

##### Diagnosis.

This species is similar to *Cephaloleia applicata*. It can be distinguished by the pro-, meso-, and metasterna being impunctate laterally and by antennomeres 4 to 10 decreasing in length.

##### Distribution.

Brazil, Ecuador.

##### Type material examined.

Syntype: Ecuador, Chimbo, 1000’, 7.1897, Rosenberg [printed label]/ I. Weise det. [printed label]/ Type [printed salmon-colored label]/ Cephalolia nubila m. [handwritten label] (ZMHB, 1).

##### Specimens examined.

**Brazil:** no further data (AMNH). **Ecuador:** no further data (BMNH). Pichincha- Chimbo (ZMHB); Pedro Vicente Maldonado, 3.5 km N, 530 m, 23 March 1999 (SEMC). Total: 5.

#### Cephaloleia obsoleta

Taxon classificationAnimaliaColeopteraChrysomelidae

Weise, 1910

http://species-id.net/wiki/Cephaloleia_obsoleta

Fig. 198

Cephalolia obsoleta Weise 1910: 93. Weise 1911a: 8 (catalog), 1911b: 13 (catalog); Uhmann 1938b: 365 (comparative note).
Cephaloleia obsoleta Weise. Uhmann 1957b: 22 (catalog).

##### Description.

Elongate; subparallel; subconvex; shining black, head with bluish or greenish metallic sheen. Head: vertex smooth, faint medial sulcus present; frons not projecting; depressed between eyes. Antenna: reaches to humerus; slender; antennomeres 1–2 transverse, subequal in length; 3 as long as 1–2 combined, elongate; 4 elongate, ¾ length of 3; 5–10 transverse, subequal in length, each ¾ length of 4; 11 2× length of 10, pointed at apex; 1–4 punctate with scattered setae; 5–11 setose. Pronotum: quadrate; lateral margin straight, slightly divergent then rounding to anterior angle, slightly canaliculate; anterior angle rounded, not produced; posterior angle acute; anterior margin weakly emarginate behind head; disc subconvex; surface finely, sparsely punctate; basal impression absent; pronotal length 1.2–1.4 mm; pronotal width 1.2–1.4 mm. Scutellum: pentagonal; impunctate. Elytron: lateral margin straight, smooth, narrowly margined; apex rounded, finely serrulate; sutural angle with small tooth; humerus rounded, not produced; slightly constricted behind humerus; finely punctate-striate, punctures larger and deeper apically, rows converge and unite apically; elytral length 4.0–4.4 mm; elytral width 1.5–1.7 mm. Venter: pro-, meso-, and metasterna punctate; abdominal sterna sparsely, irregularly punctate, each puncture with white seta; suture between sterna 1 and 2 complete. Leg: slender; punctate, each puncture with pale seta; tibia with fringe of setae on inner margin of apex. Total length: 5.3–5.8 mm.

##### Diagnosis.

This species is similar to *Cephaloleia funesta* and *Cephaloleia impressa*. It can be distinguished by the vertex of the head having a medial sulcus, by the elytra being regularly convex, and by antennomere 1 being shoter than 3.

##### Distribution.

Brazil.

##### Type material examined.

Syntype: Brasilia [green handwritten label]/ J. Weise det. [printed label]/ Type [printed salmon-colored label]/ Cephalolia obsoleta m [handwritten label] (ZMHB, 1).

##### Specimens examined.

**Brazil:** São Paulo- Cipo, 1966 (EGRC). Total: 2.

#### Cephaloleia ochra

Taxon classificationAnimaliaColeopteraChrysomelidae

Staines sp. n.

http://zoobank.org/EA2DA715-B22B-4B4F-B913-90ABD67D98DA

http://species-id.net/wiki/Cephaloleia_ochra

Fig. 199

##### Description.

Elongate; subparallel; flattened; ochre-yellow; eyes and apical antennomeres darker. Head: vertex finely punctate, medial sulcus present; small triangular projection present between antennal bases; frons not projecting; not depressed between eyes. Antenna: reaches to humerus; slender; antennomeres 1–5 elongate, cylindrical; 1 incrassate; 2 ¾ length of 1; 3 slightly longer than 2; 2 and 4 subequal in length; 5 shorter than 4; 6–10 transverse, subequal in length, each shorter than 5; 11 2× length of 10, bluntly pointed at apex; 1–3 punctate with scattered setae; 4–11 setose. Pronotum: transverse; lateral margin nearly straight then rounding to anterior angle, canaliculate; anterior angle broadly rounded, slightly produced; posterior angle acute; anterior margin emarginate behind head; disc subconvex; surface irregularly punctate, anterior margin behind head, lateral margin, and medial longitudinal area from base to apex impunctate; faint medial longitudinal sulcus present; basal impression absent; pronotal length 1.4 mm; pronotal width 1.9 mm. Scutellum: pentagonal; impunctate. Elytron: lateral margin straight, smooth, margined; apex rounded; sutural angle without tooth; humerus rounded, not produced; slightly constricted behind humerus; finely punctate-striate, rows converge and unite apically; elytral length 5.0 mm; elytral width 2.7 mm. Venter: prosternum impunctate; mesosternum sparsely punctate medially, more densely punctate laterally; metasternum impunctate medially, punctate laterally; abdominal sterna finely punctate, each puncture with pale seta; suture between sterna 1 and 2 complete; last sternite with apical margin rounded in female. Leg: slender; coxa, femur and tibia punctate; tibia with fringe of setae on inner margin of apex. Total length: 7.0 mm.

##### Etymology.

From ochrus (Latin) for the ochre yellow body color of this species. The name is feminine.

##### Diagnosis.

This species is similar to *Cephaloleia apicicornis*, *Cephaloleia corallina*, *Cephaloleia halli*, and *Cephaloleia proxima*. It can be distinguished by the pronotum lacking a transverse basal impression, by the basal antennomeres being pale, by the meso- and metasterna being impunctate, by the vertex of the head not depressed between the eyes, by antennomeres 2 and 4 being subequal in length, and by the anterior angle of the pronotum being rounded.

##### Distribution.

Ecuador.

##### Type material.

Holotype female: Ecuador, Imbabura, 3500’, II.97, dry season (Rosenberg)/ F. Monros collection 1959/ Holotype *Cephaloleia ochra* Staines, des. C. L. Staines 2012 [red label] (USNM).

#### Cephaloleia opaca

Taxon classificationAnimaliaColeopteraChrysomelidae

Baly, 1858

http://species-id.net/wiki/Cephaloleia_opaca

Fig. 200

Cephalolia opaca Baly 1858: 62. Gemminger and Harold 1876: 3602 (catalog); Donckier 1899: 550 (catalog); Weise 1906: 222 (museum list), 1910: 88 (noted), 1911a: 8 (catalog), 1911b: 11 (catalog); Uhmann 1935b: 47 (faunal list), 1942b: 96 (noted); Guérin 1953: 97 (faunal list); Lima 1955: 207 (faunal list).
Cephaloleia opaca Baly. Baly 1858: 167 (noted); Lima 1955: 207 (faunal list); Uhmann 1957b: 22 (catalog), 1968c: 125 (faunal list); Staines and Staines 1999: 524 (Baly species list); Staines 2004a: 312 (host plants).
Himatidium fernandoi Bondar 1940b: 38 (type: Brazil, Bahia, Jequié; Nazareth; BMNH). Bondar 1940c: 850 (description); Monrós 1945: 413 (synonymy); Monrós and Viana 1947: 413 (synonymy); Bachmann and Cabrera 2010: 64 (types).

##### Description.

Subelongate; subdepressed; pale yellow, eyes, antennae and pleurae darker; elytra with suture behind middle with indistinct dark macula; venter pale yellowish. Head: vertex finely not densely punctate, with longitudinal medial carina; frons not projecting; depressed between eyes. Antenna: as long as head and pronotum combined; robust, compact; antennomeres short; 1 obovate, not incrassate, 2× longer than 2; 2 subglobose, shortest; 3 cylindrical, slightly longer than 2; 4–10 transverse, subequal in length, each shorter than 3; 11 2× length of 10, pointed at apex; 1–2 punctate with scattered setae; 3–11 setose. Pronotum: transverse, twice as wide as long at base; lateral margin nearly straight basally then rounding and narrowing to anterior angle, margined; anterior angle obtuse, produced; posterior angle acute; anterior margin emarginate behind head; disc subconvex; surface coarsely, deeply punctate, more dense laterally; basal impression absent; pronotal length 1.4–1.6 mm; pronotal width 2.0–2.2 mm. Scutellum: triangular; impunctate. Elytron: lateral margin straight, smooth, margined; apex rounded; sutural angle without tooth; humerus rounded, not produced; slightly constricted behind humerus; slightly flattened along suture; subconvex laterally; moderately punctate-striate, rows converge and unite apically; interspaces sulcate laterally; elytral length 4.8–5.2 mm; elytral width 2.7–2.9 mm. Venter: pro-, meso-, metasterna, and abdominal sterna impunctate; suture between sterna 1 and 2 entirely obsolete; last sternite with apical margin bisinuate in female, emarginate medially in male. Leg: slender; punctate; tibia with fringe of setae on inner margin of apex. Total length: 6.5–6.8 mm.

##### Diagnosis.

This species is similar to *Cephaloleia castanea* and *Cephaloleia nigricornis*. It can be distinguished by the vertex of the head having a medial carina.

##### Host plant.

*Calathea ovata* Lindl., *Cephaloleia virginalis* Linden (Marantaceae) (Bondar 1940b, 1940c).

##### Distribution.

Brazil (Bahia, Rio de Janeiro, Santa Catharina), Peru, Venezuela.

##### Type material examined.

Holotype male: Brazil [handwritten label]/ Baly coll. [printed label]/ Cephalolia opaca Baly, Brazil [blue handwritten label] (BMNH).

##### Specimens examined.

**Brazil:** no further data (USNM). Bahia- no further data (USNM); Jequié (BMNH); Nazareth (BMNH). Rio de Janeiro- 1883, 1945 (USNM). Santa Catharina- Corupa, October 1944 (AMNH), January 1947 (USNM). **Peru:** Loreto- nr. Laguna Reserva Pacaya-Samiria, 31 March- 5 April 1998 (BYUC). **Venezuela:** Aragua- Portachelo Pass, 12 July 1987 (BYUC). Total: 14.

#### Cephaloleia orchideivora

Taxon classificationAnimaliaColeopteraChrysomelidae

Sekerka, Windsor & Staines, 2013

http://species-id.net/wiki/Cephaloleia_orchideivora

Fig. 201

Cephaloleia orchideivora Sekerka et al. 2013: 305.

##### Description.

Elongate-oval; subconvex; head metallic olive-green with bluish-violet reflection; antennae (except basal antennomere rust-colored) black; pronotum yellow (in dry specimens) to pink (in live specimens) with medial black triangular marking from base to apex; scutellum olive-green; elytra brownish or yellow with broad olive-green marking from base to apex from suture to puncture row 7; venter diffuse brownish-black medially, yellowish laterally; legs black on upper surface, yellowish on lower surface. Head: vertex densely, coarsely punctate, punctures tend to for short striae, medial sulcus faint; frons impunctate, not projecting; depressed between eyes. Antenna: reaches beyond humerus; slender; antennomeres 1–2 elongate, cylindrical, subequal in length, each ½ length of 3; 3 elongate, cylindrical; 4–10 cylindrical, decreasing in length; 11 2× length of 10, rounded at apex; 1–2 punctate with scattered setae; 3–11 setose. Pronotum: transverse; lateral margin straight then rounding to anterior angle (some specimens regularly arcuate), slightly explanate, margined; anterior angle broadly rounded, slightly produced; posterior angle acute; anterior margin emarginate behind head; disc subconvex; surface irregularly foveolate-punctate, punctures finer on disc; transverse basal impression present medially; pronotal length 1.0–1.4 mm; pronotal width 1.5–2.0 mm. Scutellum: pentagonal; impunctate. Elytron: lateral margin straight, slightly serrate, margined; apical margin rounded, serrate; sutural angle emarginate, without tooth; humerus broadly rounded, not produced; slightly constricted behind humerus; moderately punctate-striate, punctures slightly foveolate, additional row of punctures present between puncture rows 7 and 8 after middle, row 10 removed from lateral margin, rows converge and unite apically, rows 7 and 8 confused; elytral length 4.7–5.2 mm; elytral width 2.4–3.1 mm. Venter: prosternum rugose medially, punctate laterally; meso- and metasterna punctate; abdominal sterna punctate, each puncture with pale seta; suture between sterna 1 and 2 complete; last sternite weakly emarginate in male, deeply emarginate in female. Leg: robust; coxa, femur, and tibia punctate; tibia with fringe of setae on inner margin of apex. Total length: 5.5–6.9 mm.

##### Diagnosis.

This species is similar to *Cephaloleia irregularis*. It can be distinguished by the regular elytral punctation, and by the additional row of elytral punctures.

##### Host plant.

*Elleanthus* cf. *robustus* (Rchb. f.) Rchb. f., *Elleanthus* sp., *Epidendrum werklei* Schltr., *Oerstedella exasperata* (Rchb. f.) Hágsater, and *Oerstedella wallisii* (Rchb. f.) Hágsater (Orchideaceae) (Sekerka et al. 2013).

##### Immatures.

Color when live is white with outer margins translucent, with one or two elongate darker areas on pronotum and last abdominal segment, two pinkish longitudinal stripes from pronotum to abdominal segment 6 (Figs 53–54). Dorsum shallowly rugose, with medial non-setose ridge from anterior to posterior margin. Pronotum raised medially at base, surface shallowly rugose and with sparse microsetae; with medial diagonal carina anteriorly; lateral margins rugose, terminating in membranous fringe. Meso- and metanota elevated medially; shallowly rugose with sparse microsetae. Abdominal tergites 1–8 without carinae, shallowly rugose with sparse microsetae. Spiracles annular with crenulate peritreme. Tergites 9–10 without spiracle, rounded apically.Venter with surface expansions smooth to shallowly tuberculate. Head with surface smooth with five large and one small stemmata on each side; clypeus with surface smooth, basally with four long, stout setae and apically with margins densely fringed with unequal short robust setae; maxially palps with 2 palpomeres, basal palpomere with two setae, apical palpomere with one lateral seta and terminating with 11 short setae; maxilla robust, clavate, with two stout setae at base of palpomere and fringes with unequal setae apically; labrium densely setose; labial palp with one palpomere with eight setae apically; mandibles shallowly quadridentate. Antenna with 3 antennomeres; antennomere 3 with one long, stout concial seta and eight short setae. Sternal and abdominal segments shorter than wide; surface smooth, concave. Leg: femur wider and shorter than tibiotarsus; tibiotarsus subconical, with stout claw and ten setae at apex. Total length 7.9 mm; width 4.4 mm. (Sekerka et al. 2013).

##### Distribution.

Panama.

##### Type material.

Holotype male: PANAMÁ: Chiriquí: holotype, ♂, glued: ‘PANAMA: Chiriqui/ LaFortuna; 1200m/ 8°45'N, 82°15'W/ 12 - V [handwritten] -199**7**/ D.M. Windsor [white, printed and cardboard label] (USNM).

##### Specimens examined.

Panamá: Panamá- Cerro Campana, 800 m, 29 April 1970 (USNM). Total: 1.

#### Cephaloleia ornata

Taxon classificationAnimaliaColeopteraChrysomelidae

Waterhouse, 1881

http://species-id.net/wiki/Cephaloleia_ornata

Fig. 202

Cephaloleia ornata Waterhouse 1881: 261. Uhmann 1957b: 23 (catalog).
Cephalolia ornata Waterhouse. Donckier 1899: 550 (catalog); Weise 1911a: 9 (catalog), 1911b: 13 (catalog).

##### Description.

Elongate; subparallel; subconvex; shining; elytra yellow with dark vitta at base along suture and apical 1/5 black, small elongate black macula on side near middle; venter yellowish medially, black laterally; legs yellowish. Head: vertex densely punctate, medial sulcus absent; frons not projecting; depressed between eyes. Antenna: longer than head and pronotum combined; slender; antennomere 1 longest, slightly incrassate; 2 ½ length 1, elongate; 3–5 cylindrical, elongate, decreasing in length, 3 subequal in length to 1; 6–10 transverse, subequal in length, each shorter than 5; 11 2× length 10, acutely pointed at apex; 1–3 punctate with scattered setae; 4–11 setose. Pronotum: transverse; lateral margin straight then rounding to anterior angle, canaliculate; anterior angle obtuse, produced; posterior angle acute; anterior margin emarginate behind head; disc subconvex; surface moderately, deeply punctate laterally, nearly impunctate on anterior margin; transverse basal impression present medially; pronotal length 1.1–1.3 mm; pronotal width 1.3–1.5 mm. Scutellum: pentagonal; impunctate. Elytron: lateral margin straight, smooth, distinctly margined; apex rounded; sutural angle without tooth; humerus rounded, not produced; slightly constricted behind humerus; distinctly punctate-striate, rows converge and unite apically; elytral length 3.9–4.4 mm; elytral width 1.7–1.9 mm. Venter: pro-, meso-, and metasterna punctate; abdominal sterna punctate, each puncture with pale seta; suture between sterna 1 and 2 obsolete medially; last sternite with apical margin emarginate medially in male, truncate in female. Leg: slender; punctate, each puncture with pale seta; tibia with fringe of setae on inner margin of apex. Total length: 5.4–5.8 mm.

##### Diagnosis.

This species is similar to *Cephaloleia fasciata*. It can be distinguished by the densely punctate vertex of the head which is depressed between the eyes.

##### Host plant.

Accodring to data, adults have been collected feeding on *Calathea lanata* Peterson (Marantaceae) and *Heliconia* sp. (Heliconiaceae).

##### Distribution.

Bolivia, Brazil (São Paulo), Colombia, Ecuador, Peru, Venezuela.

##### Type material examined.

Holotype: Type H. T. [white disk with red border]/ Ecuador, Sarayacu [handwritten label]/ Buckley [handwritten label]/ Cephaloleia ornata C. H. Waterh., (Type) [handwritten label] (BMNH).

##### Specimens examined.

**BOLIVIA:** Beni- Buena Vista, 9 July 1957 (USNM). **BRAZIL:** ?- Taperina (USNM). São Paulo- Boraceia, 14 November 1970 (USNM). **COLOMBIA:** ?- Merida (USNM). **Ecuador:** Napo- Sacha Lodge, 270 m, 22 March 1999 (SEMC); Shushfindi, 9 August 1998 (AJGC). **PERU:** Chambireyacu- Yurimaguas, June-August 1885 (USNM). Loreto- Reserva Alpahuayo Mishana, 27 May 2005 (USNM). Madre de Dios- Tambopata Wildlife Res, 30 km SW Pto Maldanado, 6 December 1982 (USNM). **Venezuela:** Aragua- Cuyagua, 15 May 1985 (BYUC); Maracay, 5 December 1968 (USNM). Total: 16.

#### Cephaloleia ornatrix

Taxon classificationAnimaliaColeopteraChrysomelidae

Donckier, 1899

http://species-id.net/wiki/Cephaloleia_ornatrix

Fig. 203

Cephaloleia ornata Baly 1885: 9 (homonym of *Cephaloleia ornata*Waterhouse 1881). Staines and Staines 1999: 524 (Baly species list).
Cephalolia ornatrix Donckier 1899: 550 (replacement name for *Cephaloleia ornata*Baly 1885). Weise 1911a: 8 (catalog), 1911b: 11 (catalog); Uhmann 1930a: 219 (redescription), 1936b: 484 (key).
Cephaloleia ornatrix Donckier. Blackwelder 1946: 719 (catalog); Papp 1953: 20 (catalog); Uhmann 1957a: 23 (catalog); Wilcox 1983: 137 (catalog); Strong 1977a: 163 (host plants); Maes and Staines 1991: 36 (faunal list); Staines 1996: 48 (Central America species), 1996(1997): 15 (Nicaragua species), 2004: 312 (host plants), 2011: 50 (faunal list); Maes 1999: 1016 (faunal list); McKenna and Farrell 2005: 119 (phylogeny), 2006: 10949 (phylogeny); García–Robledo et al. 2013a: 3 (biology), 2013b: 193 (biology).

##### Description.

Elongate; subparallel; subdepressed; shining; head, antennae, and scutellum black; pronotum and elytra reddish-brown with variable black markings; venter with prosternum and abdomen yellow, meso- and metasterna dark; leg with femur yellow with black apex, tibiae and tarsi darker. Head: vertex sparsely punctate-striate, medial carina present; frons not projecting; slightly depressed between eyes. Antenna: reaches to humerus; slender; antennomere 1 incrassate; male with antennomere 1 clavate, long, compressed, obliquely truncate apically; 2 ½ length of 1, finely punctate, setose, inner apical angle projecting; 3 1½ times length of 2, triangular; 4 subequal in length to 3, triangular; 5 subequal in length to 4, elongate; 6–10 subequal in length, expanding slightly to apex; 11 oval; female has only antennomere 1 and 3 expanded; 2 longer, elongate, ½ length of 1; 1–3 punctate with scattered setae; 4–11 setose. Pronotum: slightly wider than long; lateral margin straight, then rounding to anterior angle, margined; anterior angle rounded, produced; posterior angle slightly produced, acute; anterior margin emarginate behind head; disc sparsely punctate; surface sparsely, irregularly punctate; basal impression absent; pronotal length 1.4–1.6 mm; pronotal width 1.7–2.0 mm. Scutellum: triangular, slightly longer than wide; alutaceous. Elytron: lateral margin straight, smooth, margined; apex obtusely truncate; sutural angle without tooth; humerus rounded, not produced; slightly constricted behind humerus; flattened along suture; finely punctate-striate, punctures little impressed, nearly obsolete apically; elytral length 5.4–6.1 mm; elytral width 2.6–3.0 mm. Venter: pro-, meso-, and metasterna impunctate medially, punctate laterally, each puncture with pale seta; abdominal sterna punctate, each puncture with pale seta; suture between sterna 1 and 2 complete; last sternite with apical margin emarginate medially in male, rounded in female. Leg; slender; sparsely punctate; tibia dentate at apex, with fringe of setae on inner margin of apex. Total length: 7.4–8.3 mm.

##### Diagnosis.

This species is similar to *Cephaloleia presignis* and *Cephaloleia separata*. It can be distinguished by the elytral puncture rows being nearly obsolete apically and by antennomere 1 being incrassate subequal in length to 2 to 4 combined.

##### Host plant.

Adults have been collected on *Heliconia* sp. (Strong 1977a); *Heliconia mariae* Hook., *Heliconia pogonantha* Cufod. (Heliconiaceae) (García–Robledo et al. 2013a).

##### Distribution.

Costa Rica, Nicaragua, Panama.

##### Type material examined.

Holotype: Type H. T. [white disk with red border]/ Nicaragua, Chontales, Belt/ B. C. A., Col. VI, 2. Cephaloleia ornata, Baly/ Cephaloleia/ Cephalolia ornata Baly, Nicaragua [blue handwritten label] (BMNH).

##### Specimens examined.

**COSTA RICA:** Alajuela- Río Frío, 16 July 1972 (FSCA). Guanacaste- Est. Pitilla, 700 m, 9 km S Sta. Cecilia, P.N. Guanacaste, May 1991, July 1991, October 1991, 4–25 November 1991 (INBIO). Heredia- Finca La Selva nr. Puerto Viejo, 22 June 1969, 31 July 1969, 4 August 1969, 12 March 2005 (USNM); La Selva Biol. Sta., 2 km S. Pt. Viejo, 3–5 June 1984 (EGRC); 1 km S. Pt. Viejo, 4–5 June 1984 (EGCR); 9 km. E. Puerto Viejo, 14–15 July 1966 (BYUC, SEMC, USNM); 5 mi E Puerto Viejo, 14–15 July 1966 (BYUC); Rara Avis Biological Station, 6 November 2011 (USNM). Limón- Sector Cerro Cocorí, Fac. de E. Rojas, 150 m, June 1991, November 1991, 31 January- 21 February 1992, 26 March- 24 April 1992, April 1992, May 1992, 28 May- 17 June 1992, 26 June- 16 July 1992, 12–31 August 1992, October 1992, January 1993, February 1993, March 1993, May 1993 (INBIO); Cerro Tortuguero, 0–120 m, P. N. Tortuguero, June 1992, December 1992, January 1993 (INBIO); Est. Cuatro Esquinas, 0 m, P. N. Tortuguero, December 1989, 27 March- 29 April 1992, June 1992, August 1992, November 1992, 20 November 1992, December 1992, January 1993 (INBIO); 7 mi N Guacimo, 22 February- 3 March 1988 (BYUC); Hamburg Farm, Reventazón, Ebene Limón, 15 November 1923, 24 January 1924, 24 April 1926, 15 January 1932, 14 March 1937 (USNM), 15 February 1924 (DEI); Est. Hitoy Cerere, 100 m, R. Cerere, Res. Biol. Hitoy Cerere, 12 April 1992, 27 June- 22 July 1992, 30 June- 20 July 1992 (INBIO); Río Sardinas, 10 m, R. N. F. S. Barra del Colorado, 25 August 1992, September 1992, 20 November 1992, 11 December 1992 (INBIO); Salvadora, Parismina, 5 September 1930 (USNM); Valle La Estrella, 100–200 m (INBIO). **PANAMA:** Colón- Achiote Road, 10 km SW Gatun, 12 June 1976 (EGRC); Porto Bello, 16 February 1911, 27 February 1911, 2 March 1911, 3 March 1911, 4 March 1911, 11 March 1911, 13 March 1911 (USNM); Santa Rita Ridge, 13 June 1976 (EGRC). Panamá- Cerro Campana, 850 m, 8 April 1972, 11–15 May 1980 (EGRC). Total: 376.

#### Cephaloleia ornatula

Taxon classificationAnimaliaColeopteraChrysomelidae

Donckier, 1899

http://species-id.net/wiki/Cephaloleia_ornatula

Fig. 204

Cephaloleia ornata Duvivier 1890: xxxvii (homonym of *Cephaloleia ornata* Waterhouse, 1881).
Cephalolia ornatula Donckier 1899: 550 (replacement name for *Cephaloleia ornata* Duvivier). Weise 1911a: 9 (catalog), 1911b: 12 (catalog).
Cephaloleia ornatula Donckier. Uhmann 1957b: 23 (catalog).

##### Description.

Elongate; subparallel, rounded apically; subconvex; shining; testaceous; head black with red vertex; antennomeres 1–4 red, 5–11 black; pronotum with basal margin slightly darker; scutellum reddish; macula on each elytron behind humerus and apex black; venter testaceous, darker apically. Head: vertex densely punctate; with strong carina between antennal bases; frons not projecting; palps slender; not depressed between eyes. Antenna: as long as head and pronotum combined; slender; antennomeres 1–2 subglobular, rounded, subequal in length, each shorter than 3; 3–4 cylindrical, elongate; 3 longest; 4 shorter than 3, longer than 2; 5–10 cylindrical, subequal in length to 2; 11 acutely pointed at apex; 1–5 punctate with scattered setae; 6–11 setose. Pronotum: transverse, wider at base than apex; lateral margin sinuate then rounding to anterior angle, narrowly margined; anterior angle obtuse, not produced; posterior angle acute; anterior margin curved anteriorly; disc flattened; surface with large, scattered punctures, disc almost impunctate; with weak basal impression laterally; pronotal length 1.2–1.5 mm; pronotal width 1.8–2.0 mm. Scutellum: subpentagonal; impunctate. Elytron: lateral margin straight, smooth, narrowly margined; apex rounded; sutural angle without tooth; humerus rounded, not produced; slightly constricted behind humerus; deeply punctate-striate, punctures confused basally, rows converge and unite apically; elytral length 4.2–4.5 mm; elytral width 1.9–2.1 mm. Venter: epipleuron wide, almost flat, black at base; pro-, meso-, and metasterna punctate; abdominal sterna punctate, each puncture with a white seta; suture between sterna 1 and 2 obsolete medially; last sternite with apical margin emarginate medially in male. Leg: short; robust; impunctate; tibia with fringe of setae on inner margin of apex. Total length: 6.2–6.5 mm.

##### Diagnosis.

This species is similar to *Cephaloleia dilectans*, *Cephaloleia maxima*, and *Cephaloleia strandi*. It can be distinguished by by the two oblique impressions on the pronotum.

##### Distribution.

Brazil (São Paulo).

##### Type material examined.

Syntypes: coll. I.R.Sc.N.B. [printed label]/ America Meridional [green handwritten label]/ Type [red printed label]/ Cephaloleia ornata Duv. [handwritten label] (ISNB, 2).

##### Specimens examined.

**Brazil:** Rio de Janeiro- beach area, 19 January 1969 (EGRC). Total: 2.

#### Cephaloleia parenthesis

Taxon classificationAnimaliaColeopteraChrysomelidae

Weise, 1904

http://species-id.net/wiki/Cephaloleia_parenthesis

Fig. 205

Cephalolia parenthesis Weise 1904: 437. Weise 1911a: 9 (catalog), 1911b: 12 (catalog); Uhmann 1936a: 116 (noted); Bryant 1942: 205 (faunal list).
Cephaloleia parenthesis Weise. Blackwelder 1946: 719 (catalog); Papp 1953: 20 (catalog); Uhmann 1957a: 23 (catalog); Wilcox 1983: 137 (catalog); Staines 1996: 48 (Central America species).
Cephalolia parenthesis reducta Pic 1926a: 10 (type: Amérique méridionale, MNHN, not seen).
Cephaloleia parenthesis reducta Pic. Uhmann 1957a: 23 (catalog); Descarpentries and Villiers 1959a: 139 (types).

##### Description.

Small; elongate; subparallel; subdepressed; dark with reddish-yellow markings; elytra with yellow parenthesis-shaped vitta; venter prosternum reddish-brown; meso- and metasterna reddish-brown medially, darker laterally; abdominal sterna 1–4 yellowish medially, darker laterally; sternite 5 totally dark; legs yellowish. Head: vertex densely punctate, medial sulcus absent; frons lightly punctate, slightly projecting; slightly depressed between eyes. Antenna: reaches to humerus; slender; antennomere 1 slightly elongate; 2 transverse; 3 elongate, cylindrical, subequal in length to 1; 4–5 elongate, cylindrical, subequal in length, each shorter than 3; 6–7 elongate, subequal in length, each shorter than 5; 8–10 transverse, subequal in length; 11 2× length of 10, pointed at apex; 1–4 punctate with scattered setae; 5–11 setose. Pronotum: transverse; lateral margin nearly straight for basal ⅔, then rounding to anterior angle, margined; anterior angle produced into blunt tooth; posterior angle acute; anterior margin weakly emarginate behind head; disc subconvex; surface sparsely punctate, medial area behind apical margin punctate; depression just behind midline laterally; pronotal length 0.9 mm; pronotal width 1.1 mm. Scutellum: pentagonal, apex acute; micropunctate. Elytron: lateral margin expanding apically, smooth; apex rounded; sutural angle without tooth; humerus rounded, not produced; slightly constricted behind humerus; moderately punctate-striate, humerus nearly impunctate, rows converge and unite apically; elytral length 3.0 mm; elytral width 1.6 mm. Venter: pro-, meso-, and metasterna impunctate medially, punctate laterally; abdominal sterna punctate, each puncture with pale seta; suture between sterna 1 and 2 complete; last sternite with apical margin broadly emarginate medially in male, subtruncate in female. Leg: slender; punctate, each puncture with pale seta; tibia with fringe of setae on inner margin of apex. Total length: 4.3 mm.

##### Diagnosis.

This species is similar to *Cephaloleia scitulus*. It can be distinguished by the suture between abdominal sterna 1 and 2 being complete and by the sutural angle of the elytra without a small tooth.

##### Host plant.

According to label data, adults have been collected on *Calathea* sp. (Marantaceae).

##### Distribution.

Mexico, Peru, Venezuela.

##### Type material examined.

Holotype male: Puerto Cabello, Mex. Hanil [green label]/ Puerto Cabello, Sievers leg., eog. Ges. ded. 6.X.9 [reversed white label]/ 105996/ Paratypus [red label]/ male/ Zool. Mus. Berlin/ parenthesis m (ZMHB).

##### Specimens examined.

**MEXICO:** no further data (NMHN). **Peru:** intercepted at Miami, Florida, 15 July 1977 (USNM). Loreto- Iquitos, 90 m, 7 May 1992 (SEMC); 1.5 km N Teniente Lopez, 210–240 m, 22 July 1993 (SEMC). Pasco- Villa Rica-Puerto Bermudas Rd., 910 m, 17 October 1999 (SEMC). **Venezuela:** Tachira- Pueblo Nuevo, 29 June 1983 (BYUC). Total: 5.

#### Cephaloleia partita

Taxon classificationAnimaliaColeopteraChrysomelidae

Weise, 1910

http://species-id.net/wiki/Cephaloleia_partita

Fig. 206

Cephalolia partita Weise 1910: 88. Weise 1911a: 9 (catalog), 1911b: 11 (catalog); Uhmann 1931: 219 (museum list), 1936a: 114 (noted).
Cephaloleia partita Weise. Blackwelder 1946: 719 (catalog); Papp 1953: 20 (catalog); Uhmann 1957a: 23 (catalog); Staines 1996: 49 (Central America species), 2004: 312 (host plants); McKenna and Farrell 2005: 119 (phylogeny), 2006: 10949 (phylogeny); Meskins et al. 2008: 163 (host plants), 2011: 483 (food web).

##### Description.

Elongate; subparallel; subdepressed; shining; yellowish with eyes, antennae, and apical ½ of elytra black. Head: vertex impunctate, medial sulcus absent; frons not projecting; not depressed between eyes. Antenna: reaches to humerus; slender; antennomere 1 clavate, longer than 3; 2 transverse, ½ length of 1; 3–5 elongate, subequal in length; 6–10 transverse, subequal in length, each shorter than 5; 11 2× length of 10, rounded at apex; 1–3 punctate, with scattered setae; 4–11 setose. Pronotum: transverse; lateral margin straight for basal ¾ then rounding to anterior angle, margined; anterior angle rounded, not produced; posterior angle acute; anterior margin straight; disc flattened; surface with scattered punctures along basal and lateral margins; basal impression absent; pronotal length 1.0–1.1 mm; pronotal width 1.4 mm. Scutellum: triangular; impunctate. Elytron: lateral margin straight, smooth, margined; apex rounded; sutural angle with minute tooth; humerus rounded, not produced; slightly constricted behind humerus; moderately punctate-striate, humerus almost impunctate, rows converge and unite apically; elytral length 4.3–4.4 mm; elytral width 1.9 mm. Venter: pro-, meso-, and metasterna impunctate; abdominal sterna punctate, each puncture with pale seta; suture between sterna 1 and 2 complete; last sternite with apical margin deeply rounded in male, broadly truncate in female. Leg: slender; impunctate; tibia with fringe of setae on inner margin of apex. Total length: 5.9–6.0 mm.

**Figures 206–214.**
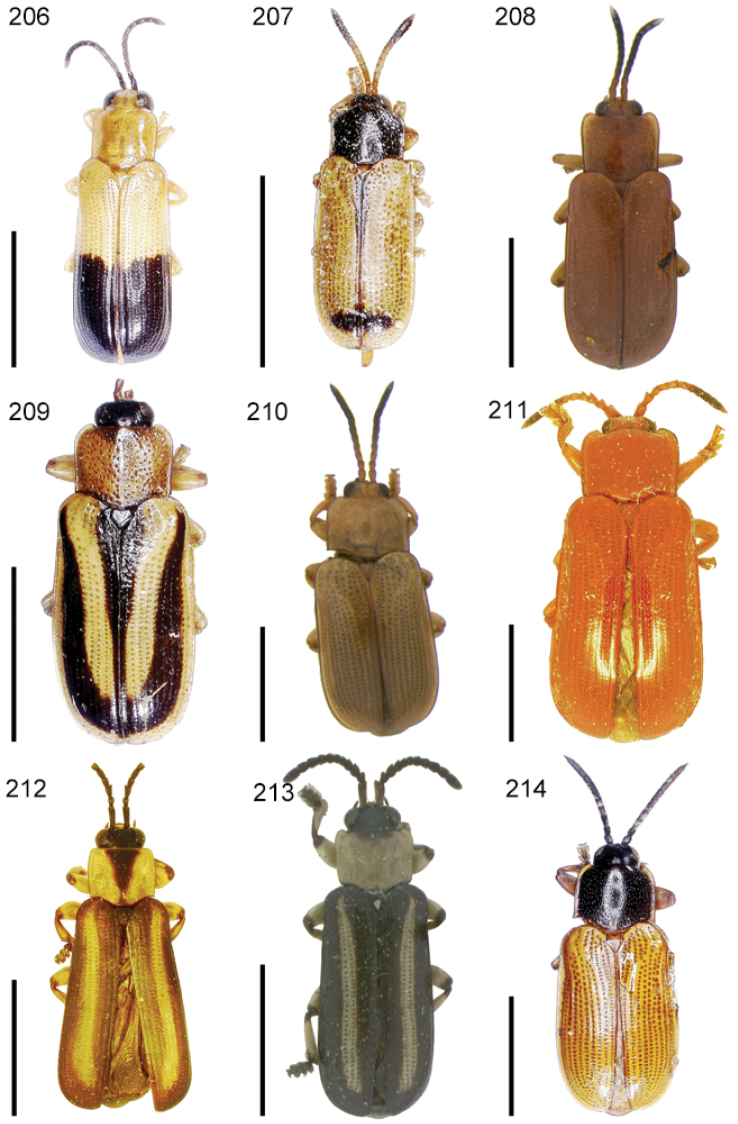
Habitus. **206** *Cephaloleia partita* **207** *Cephaloleia parvula* **208** *Cephaloleia perplexa* **209** *Cephaloleia picta* **210** *Cephaloleia placida* **211** *Cephaloleia polita* **212** *Cephaloleia postuma* **213** *Cephaloleia presignis* **214** *Cephaloleia pretiosa*. Scale bars equal 3 mm.

##### Diagnosis.

This species is similar to *Cephaloleia chevrolatii* and *Cephaloleia elegantula*. It can be distinguished by the clavate antennomere 1 and the punctate elytral humerus.

##### Host plant.

Adults have been collected on *Heliconia* sp. (Staines 1996); *Heliconia lathispatha* Benth. (McKenna and Farrell 2005) (Heliconiaceae); *Calathea latifolia* Klotzsch (Marantaceae), *Heliconia catheta* R. R. Smith (Heliconiaceae) (Meskins et al. 2008); *Calathea lutea* G. Mey.

##### Distribution.

Bolivia, Colombia, Panama, Peru, Venezuela.

##### Type material examined.

Syntype: Colombia, Rio Magdalena, Thieme [Green printed label]/ J. Weise det. [printed label]/ Type [printed salmon-colored label]/ Cephalolia partita m. [handwritten label] (ZMHB).

##### Specimens examined.

**Bolivia:** La Paz- Tumupasa, November-December (USNM). **Colombia:** Sartander- Puerto Berrío, 8 August 1938 (USNM). **PANAMA:** Bocas del Toro- 6 km N Punta Peña, 27 May 1993 (CDFA). Colón- Achiote Road, 10 km SW Gatun, 12 June 1976 (EGRC); 1 mi S Gamboa, 9 November 1969 (EGRC); Reserva Sobrina, Pipeline road, 23 May 1993 (AJGC, CDFA), 17 May 1993 (AGJC); Porto Bello, 18 February 1911, 20 February 1911, 26 February 1911, 27 February 1911, 28 February 1911, 2 March 1911, 3 March 1911, 4 March 1911, 11 March 1911, 12 March 1911, 13 March 1911, 16 March 1911 (USNM). Panamá- Ancón, 19–21 August 1970 (USNM); Arraijan, 25 December 1972 (EGRC); 40 km SE Cañita, 26 May 1994 (CDFA); Cerro Campana, 11–15 May 1980 (EGRC, USNM), 20 June 1985 (EGRC), 17 May 1993 (CDFA); Ft. Clayton, 21 June 1972 (EGRC); Madden Dam, 19 December 1971 (EGRC); Madden Forest, 24 December 1969 (EGRC); Madden Rd., 27 February 1959 (FSCA); farm near Summit, 17 May 1950 (USNM). **Peru:** Loreto- 1.5 km N Teniente Lopez, 210–240 m, 20 July 1993 (SEMC). Madre de Dios- Tambopata, 15 km NE Puerto Maldanado, Reserva Cuzco, Amazónico, 22 June 1989 (SEMC). Total: 84.

#### Cephaloleia parvula

Taxon classificationAnimaliaColeopteraChrysomelidae

Weise, 1910

http://species-id.net/wiki/Cephaloleia_parvula

Fig. 207

Cephalolia parvula Weise 1910: 93. Weise 1911a: 9 (catalog), 1911b: 12 (catalog); Uhmann 1936b: 116 (noted).
Cephaloleia parvula Weise. Uhmann 1957b: 23 (catalog).

##### Description.

Elongate; subparallel; subdepressed; head black; antennomeres 1–7 reddish, 8–11 darker; palps and legs yellowish; pronotum black with paler anterior and lateral margins; elytra reddish-yellow with black longitudinal vitta on basal ½ along suture and black dilated macula apically; venter with pro- and mesosterna black, metasternum reddish-yellow medially, black laterally, abdominal sterna reddish-yellow; legs reddish-yellow. Head: vertex distinctly, finely punctate, medial sulcus absent; frons not projecting; slightly depressed between eyes. Antenna: reaches to humerus; slender; antennomeres 1–5 elongate, cylindrical; 1 slightly longer and thicker than 2; 3 longer than 1; 4–5 shorter than 3, subequal in length; 6–10 transverse, subequal in length, each shorter than 5; 11 2× length of 10, pointed at apex; 1–3 punctate with scattered setae; 4–11 setose. Pronotum: transverse; lateral margin straight then weakly rounding to anterior angle, distinctly margined; anterior angle rounded, not produced; posterior angle acute; anterior margin weakly emarginate behind head; disc subconvex; surface finely punctate, with medial dilated line impunctate; basal impression absent; pronotal length 0.8–1.0 mm; pronotal width 1.0–1.2 mm. Scutellum: pentagonal; impunctate. Elytron: lateral margin straight, smooth, narrowly margined; apex rounded; sutural angle without tooth; humerus rounded, not produced; slightly constricted behind humerus; shallowly punctate-striate, punctures large, rows converge and unite apically; elytral length 2.9–3.2 mm; elytral width 1.3–1.5 mm. Venter: pro-, meso-, and metasterna impunctate medially, punctate laterally; abdominal sterna punctate, each puncture with pale seta; suture between sterna 1 and 2 complete; last sternite with apical margin truncate, rounded medially in male, emarginate medially in female. Leg: slender; punctate, each puncture with pale seta; tibia with fringe of setae on inner margin of apex. Total length: 3.9–4.1 mm.

##### Diagnosis.

This species is similar to *Cephaloleia emdeni*. It can be distinguished by the vertex of the head beign depressed between the eyes.

##### Host plant.

According to the label data, adults have been collected on *Carex* sp. (Cyperaceae).

##### Distribution.

Bolivia, Brazil (Goyas, Río Grande do Sul).

##### Type material examined.

Syntype: Brasilia, Goyaz, Jatahy, Clavareau [green printed label]/ J. Weise det. [printed label]/ Type [printed salmon-colored label]/ Cephalolia parvula m. [handwritten label] (ZMHB,1).

##### Specimens examined.

**Bolivia:** La Paz- Mapiri Dist., September 1925 (USNM). Santa Cruz- Andres Ibanez, Potreillos de Grunda, 400 m, 14–18 January 2007 (USNM). **Brazil:** Río Grande do Sul- Vila Oliva, 4 February 1952 (USNM). Total: 3.

#### Cephaloleia perplexa

Taxon classificationAnimaliaColeopteraChrysomelidae

Baly, 1885

http://species-id.net/wiki/Cephaloleia_perplexa

Fig. 208

Cephaloleia perplexa Baly 1885: 13. Blackwelder 1946: 719 (catalog); Papp 1953: 20 (catalog); Uhmann 1957a: 23 (catalog); Wilcox 1983: 137 (catalog); Staines 1996: 49 (Central America species); Staines and Staines 1999: 524 (Baly species list); Naczi and Staines 2011: 3 (faunal list); García–Robledo et al. 2013a: 3 (biology), 2013b: 193 (biology).
Cephalolia perplexa Baly. Donckier 1899: 550 (catalog); Weise 1911a: 9 (catalog), 1911b: 11 (catalog).

##### Description.

Elongate; subparallel; subdepressed; shining; reddish-brown, eyes and antennae darker. Head: vertex sparsely punctate, faint medial carina present; frons not projecting; not depressed between eyes. Antenna: more than ½ body length; slender; antennomere 1 elongate; 2 elongate, shorter than 1, subequal in length to 3; female with 3 triangular, male with 3 elongate; 4–10 transverse, subequal in length; 11 rounded at apex, subequal in length to 1; 1–2 punctate with scattered setae; 3–11 setose. Pronotum: transverse; lateral margin straight then rounding to anterior angle in male, canaliculate, female with margin convergent then rounding to anterior angle, canaliculate; anterior angle obtuse, slightly produced; posterior angle acute; anterior margin emarginate behind head; disc flattened; surface punctate, center of disc impunctate; basal impression absent; pronotal length 1.1–1.3 mm; pronotal width 1.4–1.9 mm. Scutellum: elongate, triangular; impunctate. Elytron: lateral margin straight, smooth, margined; apex rounded; sutural angle without tooth; humerus rounded, not produced; slightly constricted behind humerus; moderately punctate-striate, rows converge and unite apically, at base punctures sparse and slightly confused; elytral length 3.7–4.7 mm; elytral width 2.0–2.6 mm. Venter: pro-, meso-, and metasterna punctate; abdominal sterna punctate, each puncture with pale seta; suture between sterna 1 and 2 obsolete medially; last sternite with apical margin truncate medially in male, rounded in female. Leg: slender; punctate, each puncture with pale seta; tibia with fringe of setae on inner margin of apex. Total length: 5.1–6.3 mm.

##### Diagnosis.

This immaculate reddish-brown species is recognized by not being depressed between the eyes, by the suture between abdominal sterna 1 and 2 being obsolete medially, and by the elytral punctures being confused basally.

##### Host plant.

*Heliconia bourgaeana* Griggs, *Heliconia lathispatha* Peterson (Heliconiaceae) (Naczi and Staines 2011); *Cephaloleia inocephala* Kuntze, *Cephaloleia lutea* Schult., *Cephaloleia similis* H. Kenn.; *Calathea* sp., *Cephaloleia foliosa* Rowlee ex. Woodson and Schery, *Cephaloleia guzmanioides* L. B. Sm. and Idrodo, *Cephaloleia leucostachys* Hook., *Cephaloleia marantifolia* Standl., *Pleiostachya leiostachya* (Donn. Sm.) Hammel (Marantaceae).

##### Distribution.

Belize, Costa Rica, Guatemala, Mexico.

##### Type material examined.

Holotype female: Type H.T. [white disk with red border]/ Senahu, Vera Paz. Champion/ B.C.A., Col. VI, 2. Cephaloleia perplexa Baly/ Cephaloleia perplexa Baly, Guatemala (BMNH).

##### Specimens examined.

**BELIZE:** Stann Creek- 12 mi SW Stann Creek Town, 17 August 1977 (EGRC). Toledo- ca. 9 mi NNE Medina Bank, N side Bladen Branch, 6 January 2006, 4 January 2007 (USNM); Belize Foundation for Research and Environmental Education property, 4 January 2007 (USNM). **COSTA RICA:** Alajuela- Sector Colonia, R. B. San Ramón, 600–700 m (INBIO); Río San Lorencito, 5 km N Colonia Palmareña, 900–1000 m (INBIO); Upala, Sector San Ramón de Dos Ríos, 1.5 km NW Hacienda Nueva Zelandia, 600–700 m (INBIO). Cartago- Estación Quebrada Segundo, 1200–1300 m (INBio); Quebrada Segunda, 1200–1300 m (INBIO); Río Grande de Orosí, 1500–1600 m (INBIO); Turrialba, Santa Teresita, Monumento Nacional Guayabo, 1100–1200 m (INBIO); Grano de Oro, Chirripo, Turrialba, 1100–1200 m (INBIO). Guanacaste- Estación Mengo, SW side Volcán Cacao, 1000–1100 m (INBIO); Estación Pitilla, 9 km S Santa Cecilia, 600–700 m (INBIO); Río San Lorenzo, Tierras Morenas, 900–1000 m (INBIO). Heredia- Finca Naranjo Valenciana, 2 km sur Pueblo Nuevo, Sarapiquí, 90 m, 24 July- 22 August 1992, 9–30 September 1992 (INBIO); Sarapiquí, La Virgen, P. N. Braulio Carrillo, Estació Magsasay, 100–200 m (INBIO); Rara Avis Biological Station, 6 July 2011, 7 November 2011, 8 November 2011, 13 November 2011, 15 November 2011 (USNM). Limón- R. B. Hitoy Cerere, Sendero Toma de Agua, 0–100 m (INBIO); Valle La Estrella, 100–200 m (INBIO); Pococí, Colorado, Estació Cuatro Esquinas (INBIO); Cerro Tortuguero, R. V. S. Barra del Colorado, 0–100 m (INBIO); Sardinas, Barra del Colorado, 4 km NW Cerro Cocorí, 0–100 m (INBIO); Río Sardinas, Pococí, Barra del Colorado, 0–100 m (INBIO); Pococí, Sector Cerro Cocorí, 30 km N Cariari, 100–200 m (INBIO); Pococí, Sector Cedrales de la Rita, 3 km N Puente Río suerte, Ruta Puerto Lindo, 0–100 m (INBIO); R.V.S. Gandoca Manzanillo, 0–100 m (INBIO). Puntarenas- 1 km SW Cerro Biolley, Sector Altamira, 1300–1400 m (INBIO); Estación Altamira, 1 km S Cerro Biolley, 1400–1500 m (INBIO); Altamire, Biolley, Sector Buenos Aires, 1700–1800 m (INBIO); Golfito, F. Las Cruces, Fca Ilama, 1400–1500 m (INBIO); Coto Brus, Estación Pittier, Sendero La Escuadra, 1600–1700 m (INBIO); Estación Pittier, 4.2 km SW Cerro Gemelo, 1600–1700 m (INBIO); Estación Pittier, Sendero Altamira, 1700–1800 m (INBIO); Finca Cafrosa, El Embalse, 1300–1400 m (INBIO); Finca Las Alturas, 1300–1400 m (INBIO); Garabito, Reserva Biol Carara, Est Quebrada Bonita, 0–100 m (INBIO); Garabito, Tarcoles, Estación Quebrada Bonita, 100–200 m (INBIO); Estación Esquinas, Peninsula de Osa, 0–100 m (INBIO); Golfito, Pque Nal Corcovado, Est Sirena, 0–100 m (INBIO); Estación Sirena, 0–100 m (INBIO); Cerro La Torre, Fca La Purruja, Fila Matahambre, 100–200 m (INBIO); Cerro Anguciana, Llano Bonito, Piedras Blancas, Pen. Osa, 800–900 m (INBIO); Cerro Oscuro, Llano Bonito de Piedras Blancas, 900–1000 m (INBIO); Osa, Sierpe, 0.2 km NW Estación Esquinas, 0–100 m (INBIO); Osa, Sierpe, Rancho Quemado, 200–300 m (INBIO); Guacimal, Finca Buen Amigo, Monteverde, 1000–1100 m (INBIO); Bosque Eterno de los Niños, Sector Monteverde, 1500–1600 m (INBIO); Monteverde, Estación La Casona, 1500–1600 m (INBIO). San José- Finca El Gringo, Estación Las Nubes de Santa Elena, 1200–1300 m (INBIO). Total: 62.

#### Cephaloleia picta

Taxon classificationAnimaliaColeopteraChrysomelidae

Baly, 1858

http://species-id.net/wiki/Cephaloleia_picta

Fig. 209

Cephalolia picta Baly 1858: 54. Gemminger and Harold 1876: 3602 (catalog); Donckier 1899: 550 (catalog); Weise 1911a: 9 (catalog), 1911b: 12 (catalog); Uhmann 1932a: 36 (museum list); Guérin 1953: 97 (faunal list).
Cephaloleia picta Baly. Uhmann 1951b: 333 (noted), 1957b: 23 (catalog); Balazuc 1988: 397 (pathogens); Staines and Staines 1999: 524 (Baly species list).
Cephaloleia picta interrupta Uhmann 1951b: 333 (type: Brazil, Río de Janiero, depository unknown). Uhmann 1957b: 23 (catalog), 1960f: 303 (noted), 1964a: 404 (catalog); Staines 1997b: 414 (Uhmann species list).

##### Description.

Narrowly elongate; subdepressed; shining; head, antennae (except antennomeres 1–4 yellowish), and scutellum black; pronotum yellow; elytra black, with yellow lateral margin and broad yellow subsutural vitta which reaches to beyond middle and connects to lateral margin; venter black with lateral margin of abdomen piceous; coxae and legs yellow, darkened at tibio-tarsal joint. Head: vertex with small fovea, faint medial carina present; front and vertex finely punctate; frons not projecting; slightly depressed between eyes Antenna: reaches to humerus; robust; antennomere 1 clavate, slightly incrassate, longer than 2; 2 transverse, ½ length of 1; 3 elongate, cylindrical, ¾ length of 1; 4–10 transverse, subequal in length; 11 2× length of 10, subequal in length to 2, broadly rounded at apex; 1–2 punctate with scattered setae; 3–11 setose. Pronotum: transverse; lateral margin slightly sinuate, narrowed basally then rounding to anterior angle, canaliculate; anterior angle obtuse, produced; posterior angle acute; anterior margin emarginate behind head; disc subconvex; surface deeply, coarsely punctate, more dense laterally; basal impression absent; pronotal length 1.2–1.4 mm; pronotal width 1.5–1.7 mm. Scutellum: pentagonal; impunctate. Elytron: lateral margin slightly expanding apically, smooth, slightly margined; apex obtusely rounded; weakly emarginate at suture, sutural angle without tooth; humerus rounded, not produced; slightly constricted behind humerus; flattened; moderately punctate-striate, rows converge and unite apically; lateral interspaces sulcate; pygidium obtuse; elytral length 4.0–4.3 mm; elytral width 1.9–2.1 mm. Venter: pro-, meso-, and metasterna impunctate medially, punctate laterally; abdominal sterna punctate, each puncture with pale seta; suture between sterna 1 and 2 complete; last sternite with apical margin broadly emarginate, sinuate medially in male, sinuate in female. Leg: slender; punctate; tibia with fringe of setae on inner margin of apex. Total length: 5.1–5.8 mm.

##### Diagnosis.

This species is similar to *Cephaloleia vittipennis*. It can be distinguished by the medial fovea on the vertex of the head and by the basal impression on the pronotum.

##### Distribution.

Argentina, Brazil (Bahia, Santa Catharina), Paraguay.

##### Type material examined.

Syntype: Brazil [handwritten label]/ Cephalolia picta Baly, Brazil [blue handwritten label] (BMNH, 1).

##### Specimens examined.

**ARGENTINA:** Chaco- October-December 1935 (USNM). Misones- Dos de Mayo, November 1989 (USNM). **Brazil:** Bahia- no further data (USNM). Sanata Catharina- Corupa, November 1944 (AMNH). **Paraguay:** Cordillera- Inst. Agro. Nac. Caacupe, 17–20 January 1983 (EGRC). Total: 11.

#### Cephaloleia placida

Taxon classificationAnimaliaColeopteraChrysomelidae

Baly, 1885

http://species-id.net/wiki/Cephaloleia_placida

Fig. 210

Cephaloleia placida Baly 1885: 11. Calvert and Calvert 1909: 394 (noted); Blackwelder 1946: 712 (catalog); Papp 1953: 20 (catalog); Uhmann 1957a: 23 (catalog); Wilcox 1983: 137 (catalog); Staines 1996: 50 (Central America species), 2004: 312 (host plants), 2011: 50 (faunal list); Staines and Staines 1997: 17 (types), 1999: 524 (Baly species list); McKenna and Farrell 2005: 119 (phylogeny); García–Robledo and Horvitz 2009: 116 (host plants), 2011: 978 (biology), 2012: 40 (biology); García–Robledo et al. 2010: 51, 2013a: 3 (biology); (larva, biology); Barrett and Heil 2012: 283 (noted).
Cephalolia placida Baly. Donckier 1899: 550 (catalog); Weise 1910: 87 (noted), 1911a: 9 (catalog), 1911b: 11 (catalog); Calvert and Calvert 1917: 394 (noted).
Cephaloleia placida variicornis Weise 1910: 88 (type: Panamá, ZMHB, not seen). Weise 1911a: 9 (catalog), 1911b: 11 (catalog); Uhmann 1957a: 23 (catalog), 1968: 248 (faunal list).

##### Description.

Elongate; slightly expanding apically; subdepressed; shining; reddish-brown, eyes and antennae (except antennomeres 1–2) darker. Head: vertex punctate, medial sulcus present; wide keel present between antennal bases; frons not projecting; depressed between eyes. Antenna: ½ body length; slender; antennomere 1 clavate, compressed, longer than 2; 2 transverse, robust, subequal in length to 3; 4–10 transverse; subequal in length, each shorter than 3; 11 2× length of 10, rounded at apex; 1–4 punctate with scattered setae; 5–11 setose. Pronotum: subquadrate; lateral margin straight then rounding to anterior angle, margined; anterior angle obtuse, produced; posterior angle acute; anterior margin curved posteriorly; disc flattened; surface finely, sparsely punctate; basal impression absent; pronotal length 1.3–1.4 mm; pronotal width 1.9–2.0 mm. Scutellum: elongate, acutely triangular; impunctate. Elytron: lateral margin straight, smooth, margined; apex rounded; sutural angle without tooth; humerus rounded, slightly produced; slightly constricted behind humerus; flattened along suture; shallowly punctate-striate, punctures large, rows converge and unite at apex; last segment of pygidium u-shaped in male, slightly acuminate in female; elytral length 4.4–5.4 mm; elytral width 2.3–2.6 mm. Venter with pro-, meso-, and metasterna impunctate medially, punctate laterally; abdominal sterna punctate, each puncture with pale seta; suture between sterna 1 and 2 complete; last sternite with apical margin sinuate medially in male, rounded, entire in female. Leg: robust; sparsely punctate; tibia with fringe of setae on inner margin of apex. Total length: 6.1–7.1 mm; male larger than female.

##### Diagnosis.

This species is similar to *Cephaloleia mauliki*, *Cephaloleia simplex*, and *Cephaloleia sulciceps*. It can be distinguished by the suture between abdominal sterna 1 and 2 being complete, by the larger size, and by the pronotal disc being impunctate.

##### Host plant.

Adults have been collected on *Heliconia* sp. (Heliconiaceae) (Calvert and Calvert 1909); *Renealmia* sp. (Staines 1996); *Renealmia alpinia* (Rottb.) Maas, *Renealmia cernua* (Sw. ex. Roem. and Schult.) J. F. Macbr. (García–Robledo and Horvitz 2009); *Alpinia purpurata* K. Schum., *Hedychium coronarium* J. Koenig (Zingiberaceae).

##### Immatures.

Color when live (Figs 43–46) brownish-yellow with body proper reddish, margins translucent; venter paler. Color when dead dirty-brown with paler margins. Dorsum with longitudinal medial setose ridge extending from anterior to posterior margins. Pronotum without diagonal carinae on central raised area; central area slightly raised, micropunctate; lateral areas micropunctate. Mesonotum without carinae, micropunctate. Metanotum with transverse carina in middle of each side. Abdominal tergites 1–6 slightly narrowed medially; with transverse carina in middle of each side just off central elevation; spiracles appear as darker brownish macula without darker margin. Abdominal tergites 7–10 with two carinae along margin on each side; surface micropunctate. Venter: surface of expansions rugose-punctate. Head surface punctate; clypeus slightly rugose, with fringe of setae at apex; mandibles tridentate; maxillary palps with 2 palpomeres and short, robust setae at apex; maxilla robust, clavate, with fringe of long setae at apex; labium densely setose. Antenna with antennomere 1 short, robust; 2 elongate, cylindrical, longer than 3; 3 elongate, with fringe of short setae at apex. Pro- and mesosterna wider than long; slightly depressed medially; surface rugose-striate. Metasternum longer than others; depressed medially; with suture along apical margin. Abdominal sternites 1–8 wider than long; decreasing in width; laterally with curved sulcus dividing the sternite into thirds; sterna 9–10 fused, rounded at apex. Leg: femur short, robust; tibiotarsus subconical, with a strong claw and eight setae at apex. Total length: 7.3 mm; width 4.4 mm. (García–Robledo et al. 2010).

##### Biology.

Eggs are about 2.5 mm long and are laid singly or in clusters or two or more in the concavity of leaf petioles or the inner surface of inflorescence and are covered with frass. Eggs hatch in 9 to 13 days. The larvae have two instars the first lasting 15 to 34 days and the second 43 to 75 days. The pupal stage lasts from 15 to 19 days. Adults live about 102 days (García–Robledo et al. 2010).

##### Distribution.

Colombia, Costa Rica, Panama.

##### Type material examined.

Syntypes: V. de Chiriqui, 25–4000 ft., Champion/ F. Monros Collection 1959/ Cephaloleia placida Baly, J. S. Baly det. [pink label] (USNM, 2).

##### Specimens examined.

**COSTA RICA:** Alajuela- N slope Volcán de Rincón, 2 km W Dos Ríos, 550 m, 22 May 1985 (EMEC); Upala, Aguas Claras, Pque Nal Rincón de la Vieja, Volcán Santa Maria, 600–700 m (INBIO); Upala, Sector San Ramó Dos Ríos, 1.5 km NW Hacienda Nueva Zelandia, 600–700 m (INBIO). Cartago- Turrialba, 650 m, 24 February 1980 (CMNC). Guanacaste- Estac. Pitilla, 700 m, 9 km S Sta Cecilia, December 1989 (INBIO); Río San Lorenzo, 1050 m, Tierras Morenas, R. F. Cord. Guanacaste, November 1991 (INBIO); Hda. Sta Moria, 2 February 1993 (INBIO); 3 km SE Río Narnajo, 21–30 June 1992, 1–10 September 1992, 21–30 September 1992 (BYUC). Heredia- Est. El Ceibo, Braulio Carillo, N.P., 400–600 m, April 1990 (INBIO); La Selva, nr. Pto. Viejo, 50 m, 19 February 1980 (CMNC); Sendero Antigua, Est. Carillo, 8 January 1993 (INBIO). Limón- Sector Cerro Cocorí, Fca. de E. Rojas, 150 m, November 1991, January 1992 (INBIO); Est. Exp. Diamantes, Guápiles, 6 February 1992 (MUCR); Est. Hitoy Cerere, 100 m, R. Cerere, Res. Biol. Hitoy Cerere, 28–12 April 1992 (INBIO); Valle la Estrella Pandora, 17 February 1984 (CMNC); Valle La Estella, 100–200 m (INBIO); Pococí, P.N. Colorado, Estación Cuatro Esquinas (INBIO); Llanuras del Tortuguero, Río Sardinas, Barra del Colorado, 0–100 m (INBIO). Puntarenas- Est. Biol. Las Alturas, 1500 m, Coto Brus., October 1991 (INBIO); Monteverde Cloud For. Res., 27–31 May 1984 (EGRC); Rancho Quemado, Peninsula de Osa, 200 m, October 1991 (INBIO); Río Claro, 15 August 1969 (USNM); Est. Sirena, Corcovado N.P., 0–100 m, January 1990 (INBIO); AGUIRRE, Quepos, P.N. Manuel Antonio, 0–100 m (INBIO); Buenos Aires, Sector Altamira, Biolley, 1700–1800 m (INBIO); Estación Esquinas, Peninsula de Osa, 0–100 m (INBIO); Sirena, Corcovado Nat Pk, Osa Peninsula, 0–100 m (INBIO). San José- Pque Nal Braulio Carrillo, 1600–1700 m (INBIO). **PANAMA:** Chiriquí- 2 km N Sta. Clara, 24–25 May 1977 (CMNC); V. de Chiriquí, 25–4000 ft. (AMNH); Hartmann's finca, St. Clara, 15–18 May 1985 (EGRC). Panamá- Barro Colorado Is., 16 January 1953 (USNM). Vera Paz- Chacoj (AMNH). Veraguas- Cerro Azul, 15 January 1953 (USNM). Total: 75.

#### Cephaloleia polita

Taxon classificationAnimaliaColeopteraChrysomelidae

Weise, 1910

http://species-id.net/wiki/Cephaloleia_polita

Fig. 211

Cephalolia polita Weise 1910: 83. Weise 1911a: 9 (catalog), 1911b: 10 (catalog); Uhmann 1936b: 111 (noted), 1936f: 482 (key).
Cephaloleia polita Weise. Uhmann 1957b: 23 (catalog); Gaedike and Döbler 1971: 357 (types).

##### Description.

Elongate; subparallel; subdepressed; shining; reddish, antennomeres 1–3 yellowish-brown, 4–11 darker. Head: vertex sparsely, irregularly punctate, medial sulcus absent; frons not projecting; not depressed between eyes. Antenna: as long as head and pronotum combined; slender; antennomere 1 incrassate, longest; 2 ½ length of 1; 3–5 cylindrical, subequal in length, each shorter than 2; 2–4 laterally compressed and triangular in male; 6–10 transverse, subequal in length, each shorter than 5; 11 2× length of 10, pointed at apex; 1–4 punctate with scattered setae; 5–11 setose. Pronotum: quadrate; lateral margin straight then rounding to anterior angle, canaliculate; anterior angle rounded, slightly produced; posterior angle acute; anterior margin emarginate behind head; disc subconvex, impunctate; sparsely punctate laterally; basal impression absent; pronotal length 1.6–1.8 mm; pronotal width 2.2–2.4 mm. Scutellum: pentagonal; impunctate. Elytron: lateral margin straight, smooth, narrowly margined; apex rounded; sutural angle without tooth; humerus rounded, not produced; slightly constricted behind humerus; declivity beginning just behind humerus at puncture row 7 not edged with faint carina; weakly punctate-striate in male, strongly punctate-striate in female; humerus sparsely punctate; scutellar row long; elytral length 5.8–6.2 mm; elytral width 2.9–3.1 mm. Venter: last sternite with apical margin curved posteriorly in male; truncate in female. Leg: slender; punctate, each puncture with pale seta; tibia with fringe of setae on inner margin of apex. Total length: 8.3–8.8 mm.

##### Diagnosis.

This species is similar to *Cephaloleia dimidiaticornis* and *Cephaloleia latipennis*. It can be distinguished by the elytral puncture rows being regular to the apex and by antennomere 1 being incrassate.

##### Distribution.

Colombia.

##### Type material examined.

Syntype: Columbien, Cauca bei Cali, Bürger [green printed label]/ J. Weise det. [printed label]/ Type [printed salmon-colored label]/ Cephalolia polita m. [handwritten label] (DEI, 1).

##### Specimens examined.

**Colombia:** Valle del Cauca- Cauca to Cali (BMNH, NHRS, ZMHB). Total: 9.

#### Cephaloleia postuma

Taxon classificationAnimaliaColeopteraChrysomelidae

Weise, 1905a

http://species-id.net/wiki/Cephaloleia_postuma

Fig. 212

Cephalolia postuma Weise 1905a: 131. Weise 1911a: 9 (catalog), 1911b: 10 (catalog).
Cephaloleia postuma Weise. Blackwelder 1946: 719 (catalog); Papp 1953: 20 (catalog); Uhmann 1957a: 23 (catalog); Staines 1996: 50 (Central America species).

##### Description.

Elongate; subparallel; subdepressed; head (except yellow frons), antennae, and scutellum black; pronotum yellowish with longitudinal black vitta; elytra black with narrow yellow vitta between puncture rows 4 and 5, lateral margin entirely black; venter with pro-, meso-, and metasternum yellow medially, black laterally, abdominal sterna 1–4 yellow medially, dark laterally, sternite 5 entirely dark; leg with coxae and trochanter blackish, femur yellow, darkened at base and apex, tibia and tarsi dark brown. Head: vertex densely punctate, medial sulcus absent; inverted V-shaped sulcus between eyes; frons not projecting; slightly depressed between eyes. Antenna: reaches to humerus; slender; antennomere 1 robust, elongate, as long as 2–4 combined; 2 transverse; 3 triangular, longer than 2; 4–6 elongate; 7–10 transverse; 11 pointed at apex; 1–3 punctate with scattered setae; 4–11 setose. Pronotum: transverse; lateral margin straight, divergent for basal ⅔ then rounding to anterior angle, narrowly margined; anterior angle rounded, slightly produced; posterior angle acute, slightly produced; anterior margin straight; disc subconvex; surface with few scattered punctures laterally; basal impression absent; pronotal length 1.0 mm; pronotal width 1.4 mm. Scutellum: triangular; pointed at apex; impunctate. Elytron: lateral margin straight, smooth, narrowly margined; apex rounded; sutural angle without tooth; humerus rounded, not produced; slightly constricted behind humerus; shallowly punctate-striate, rows converge and unite apically; elytral length 4.4 mm; elytral width 1.9 mm. Venter: pro- and mesosterna impunctate; metasternum impunctate medially, punctate laterally; abdominal sterna punctate, each puncture with pale seta; suture between sterna 1 and 2 complete. Leg slender; impunctate; tibia with fringe of setae on inner margin of apex. Total length: 5.5–6.0 mm.

##### Diagnosis.

This species is similar to *Cephaloleia eumorpha* and *Cephaloleia interrupta*. It can be distinguished by antennomere 1 being subequal in length to 2 to 4 combined and by the black color.

##### Distribution.

Mexico.

##### Type material examined.

Lectotype: Tiacotaipam/ Zool. Mus. Berlin/ Cephalolia postuma m I. Weise determ. 1904/ Lectotype Cephaloleia postuma Weise, des C. L. Staines 1993 [red label] (ZMHB).

##### Specimens examined.

**MEXICO:** Tiacotaipam (ZMHB). Total: 2.

#### Cephaloleia presignis

Taxon classificationAnimaliaColeopteraChrysomelidae

Staines, 1996

http://species-id.net/wiki/Cephaloleia_presignis

Fig. 213

Cephaloleia presignis Staines 1996: 51. Arreguin–Espinosa et al. 2000: 239 (biochemistry).

##### Description.

Elongate; subparallel; subdepressed; head (except yellow frons), antennae (except yellow basal antennomere), and scutellum black; pronotum yellowish with dark medial longitudinal macula on anterior margin; elytra black with yellow vitta; venter with pro-, meso-, and metasterna yellow medially, black laterally; abdominal sterna yellow; leg with femur yellow, darker at apex; tibia dark at base and apex, rest yellow; tarsi dark. Head: vertex sparsely punctate, medial sulcus absent; frons not projecting; slightly depressed between eyes Antenna: reaches to humerus; robust; antennomere 1 elongate, compressed laterally; 2–4 transverse, triangular; 5–10 transverse, subequal in length, each shorter than 4; 11 slightly longer than 10, rounded at apex; 1–2 punctate with scattered setae; 3–11 setose. Pronotum: lateral margin straight then rounding to anterior angle, margined; anterior angle oblique, slightly produced; posterior angle rounded, slightly produced; anterior margin emarginate behind head; disc subconvex; surface impunctate; basal impression absent; pronotal length 1.1 mm; pronotal width 1.6 mm. Scutellum: triangular; micropunctate. Elytron: lateral margin straight, smooth, margined; apex rounded; sutural angle without tooth; humerus rounded, not produced; slightly constricted behind humerus; shallowly punctate-striate, punctures obsolete apically; elytral length 4.9 mm; elytral width 2.1 mm. Venter: pro-, meso-, and metasterna impunctate medially, punctate laterally; abdominal sterna punctate, each puncture with pale seta; suture between sterna 1 and 2 complete; last sternite with apical margin bisinuate in male. Leg: slender; impunctate; tibia expanded to apex, with fringe of setae on inner margin of apex. Total length: 6.4 mm.

##### Diagnosis.

This species is similar to *Cephaloleia ornatrix* and *Cephaloleia separata*. It can be distinguished by the elytral puncture rows being nearly obsolete apically and by antennomere 1 being shorter than 2 to 4 combined.

##### Distribution.

Mexico.

##### Type material examined.

Holotype male: Jalapa Mexico/ Zool. Mus. Berlin/ Cephaloleia n. sp male, E. Uhmann Det 34/ Holotype Cephaloleia presignis Staines, Des. C. L. Staines 1993 [red label] (ZMHB).

#### Cephaloleia pretiosa

Taxon classificationAnimaliaColeopteraChrysomelidae

Baly, 1858

http://species-id.net/wiki/Cephaloleia_pretiosa

Fig. 214

Cephaloleia pretiosa Baly 1858: 50. Baly 1885: 14 (distribution); Blackwelder 1946: 719 (catalog); Papp 1953: 20 (catalog); Uhmann 1953: 47 (faunal list), 1957a: 24 (catalog); Wilcox 1983: 137 (catalog); Angel 1989: 81 (museum list); Staines 1996: 51 (Central America species); Staines and Staines 1999: 524 (Baly species list); McKenna and Farrell 2005: 119 (phylogeny), 2006: 10949 (phylogeny); Meskins et al. 2008: 163 (host plants), 2011: 483 (food web); Descampe et al. 2008: 227 (host plants).
Cephalolia pretiosa Baly. Gemminger and Harold 1876: 3602 (catalog); Donckier 1899: 550 (catalog); Weise 1910: 91 (noted), 1911a: 9 (catalog), 1911b: 12 (catalog); Uhmann 1936a: 114 (noted), 1938a: 410 (noted), 1942: 96 (noted), 1953: 47 (faunal list).

##### Description.

Elongate; subparallel; subconvex; shining; head and pronotum black; pronotal margin, scutellum, legs, and elytra reddish-brown; venter with prosternum reddish-brown medially, darker laterally, meso-, metasterna and abdominal sterna totally reddish-brown; leg with first pair black, rest reddish-brown or all reddish-brown. Head: vertex punctate, medial sulcus absent; frons not projecting; slightly depressed between eyes. Antenna: reaches to humerus; slender; antennomere 1 incrassate, longer than 2, subequal to 3; 2 transverse; 3 elongate; 4–10 transverse, decreasing in length; 11 pointed at apex, longer than 10; 1–2 punctate with scattered setae; 3–11 setose. Pronotum: subquadrate; lateral margin nearly straight then rounding to anterior angle, narrowly canaliculate; anterior angle rounded, slightly produced; posterior angle acute; anterior margin emarginate behind head; disc subconvex; surface punctate, less so on disc; basal impression absent; pronotal length 1.1–1.3 mm; pronotal width 1.7 mm. Scutellum: pentagonal, impunctate. Elytron: lateral margin straight, smooth, margined; apex rounded; sutural angle without tooth; humerus rounded, slightly produced, slightly carinate at base; slightly constricted behind humerus; declivity beginning just behind humerus at puncture row 7 not edged with faint carina; shallowly punctate-striate, rows converge and unite apically; elytral length 4.1 mm; elytral width 2.1 mm. Venter: pro-, meso-, and metasterna impunctate medially, punctate laterally; abdominal sterna punctate, each puncture with pale seta; suture between sterna 1 and 2 obsolete medially; last sternite with apical margin broadly emarginate medially in male, rounded in female. Leg: slender; punctate, each puncture with pale seta; tibia with fringe of setae on inner margin of apex. Total len gth: 5.0–6.8 mm.

##### Diagnosis.

This species is similar to *Cephaloleia brevis* sp. n. It can be distinguished by the smooth apical margin of the elytra.

##### Host plant.

*Heliconia* sp. (Heliconicaeae) (McKenna and Farrell 2005); *Calathea pulverulentus* C. Presl. (Marantaceae), *Heliconia catheta* R. R. Smith, *Heliconia latispatha* Benth., *Heliconia mariae* Hook. (Meskins et al. 2008); *Heliconia wagneriana* Peterson (Descampe et al. 2008) (Heliconiaceae).

##### Distribution.

Bolivia, Brazil, Colombia, Costa Rica, Panama, Ecuador, Peru, Venezuela.

##### Type material.

Type: Colombia (BMNH, not seen).

##### Specimens examined.

**Bolivia:** Beni- Vaea Diez, vicinity of Riberalta, 230 m, 17–23 July 1982 (USNM). **BRAZIL:** Rondonia- 62 km SW Ariquames, Fzda Rancho Grande, 12–22 November 1991 (CDFA), 15 November 1994 (BYUC). **Colombia:** intercepted in California (USA), 26 March 1991 (CDFA). **COSTA RICA:** Alajuela- Finca La Selva, Sarapiquí, 1972 (FSCA). **Ecuador:** intercepted in California (USA), 11 October 1986 (CDFA). Napo- Lago Agrio (3 km N), 27 December 1975 (USNM). Orellana- Estación Cientifica Yasuni, 215 m, 5–10 September 1999 (EGRC). Pichincha- Estación Orongo, 18 July 2001, 23 July 2001 (USNM); Reserva Maquipucuna, 9 November 1999 (USNM). **PANAMA:** Bocas del Toro- 6 km N Punta Peña, 27 May 1993 (AJGC, CDFA). Canal Zone- Jct. K-6 and K-9, 22 May 1980 (EGCR). Chiriquí- Reserva Fortuna, Fortuna Dam, 29 May 1993 (CDFA); Reserva La Fortuna, Hydrographic sta. trail, 28 May 1993 (AJGC, EGRC). Coclé- Cerro Gaital, 28 May 1994, 1 June 1993 (CDFA); rd N Cerro Gaital, 15 May 1980 (EGRC); 7.2 km NE El Copé, 730 m, 20 May 1996 (SEMC); 7.0 km N El Valle, 810 m, 19 May 1996 (SEMC). Colón- Gamboa, June 1944 (CASC), 18 June 1976 (EGRC); Gamboa, Pipeline Rd., July 1967 (USNM); Pipeline Rd., 21 June 1993 (SEMC); Gatun, 22 August 1970 (USNM); Las Cruces, 4 February 1911 (USNM); Parque Nac. Soberania, Pipeline Rd., km 2.0, 40 m, 23 June 1995 (SEMC); Porto Bello, 2 March 1911, 14 March 1911 (USNM); Paraiso, 27 January 1911 (USNM). Darién- Cana Biological Station, 500–550 m, 6 June 1996 (SEMC). Panamá- Alajuela, 5 April 1911 (USNM); Barro Colorado, 18 July 1923 (USNM); 40 km SE Cañita, 26 May 1994 (CDFA); Coco Solito Hospital, 22 May 1978 (USNM); Cerro Campana, 6 July 1974 (FSCA), 11–15 May 1980 (EGRC), 17 May 1993, 27 May 1993, 2 June 1993 (CDFA), 790 m, 1 June 1996 (SEMC); Cristobal, 19 July 1934 (CASC); Diablo Heights, 20 February 1971 (EGRC); Ft. Clayton, September 1944 (CASC); Fort Kobbe, 15 June 1976 (USNM), 1 May 1971, 2 May 1971, 6 June 1976, 8 June 1976, 15 June 1976, 20 June 1976 (EGRC), 22 May 1993, 7 June 1993 (AJGC, CDFA); Ft. Sherman, 19 January 1980 (EGRC); Las Cascadas (USNM); Madden Forest, 28 September 1969, 2 August 1970 (CMNC), 14 July 1976 (USNM), 6 March 1971, 2 November 1973, 25 June 1976 (EGRC); Madden Rd., 27 February 1959 (FSCA); 1 km N El Llano on El Llano-Carti Road, 6 June 1994 (CDFA); 6–8 km N El Llano on El Llano-Carti Road, 6 June 1994 (CDFA); Llano-Carti rd nr Jct. main hwy, 18 May 1993 (EGRC); Old Plantation Rd., 6.9 km S Gamboa, 80 m, 3 June 1995 (SEMC); Panama Road leading to La Pita signal station, 27 February 1971, 6 February 1971, 8 June 1976, 18 June 1976 (EGRC); Pipeline Road, 21 June 1993 (SEMC); 9 km N El Llano, 18 May 1993 (AJGC); Old Gamboa Road, 4 June 1993, 25 June 1994 (CDFA); Panama City (USNM); Pedro Miguel, 17 April 1911 (USNM); Powerline Road, 29 October 1972 (FSCA); Summit, September 1946 (USNM); Reserva Sobrina, Pipeline road, 23 May 1993 (CDFA). Veraguas- Sante Fe, 6.1 km N Cerro Tute, 13 June 1996 (SEMC). **Peru:** Huanuco- Huanuco, 1912 m, 21–26 May 1937 (SEMC). Jauja- Junin Dept., Sani Beni, 840 m, 11–18 October 1935 (SEMC). Loreto- Tambo Pirana on Río Cochiquinas, 1 July 1978 (USNM). **VENEZUELA:** Aragua- Rancho Grande, 10 May 1973, 23 June 1984, 12 July 1998 (USNM); Portachuelo Pass, 1120 m, 12 July 1987 (BYUC). Zulia- Kasmere, Río Yara, Sierra de Per, 250 m, 21 September 1961 (USNM). Total: 437.

#### Cephaloleia princeps

Taxon classificationAnimaliaColeopteraChrysomelidae

Baly, 1858

http://species-id.net/wiki/Cephaloleia_princeps

Fig. 215

Cephalolia princeps Baly 1858: 45. Gemminger and Harold 1876: 3602 (catalog); Donckier 1899: 550 (catalog); Weise 1910: 84 (noted), 1911a: 9 (catalog), 1911b: 10 (catalog); Uhmann 1932c: 261 (noted), 1936b: 110 (noted), 1936f: 481 (key), 1953d: 47 (faunal list).
Cephaloleia princeps Baly. Waterhouse 1881: 261 (distribution); Uhmann 1942b: 110 (pygidium), 1957b: 24 (catalog); Staines and Staines 1999: 524 (Baly species list); McKenna and Farrell 2006: 10949 (phylogeny).

##### Description.

Large; elongate; subparallel; subdepressed; shining; head, pronotum, scutellum, venter, and legs reddish; eyes and antennae black, elytra greenish-black. Head: vertex finely punctate, with small fovea; frons not projecting; slightly depressed between eyes. Antenna: as long as head and pronotum combined; slender; antennomere 1 obovate, incrassate, longer than 2; 2 transverse, triangular in male; 3 elongate, triangular in male, subequal to 1; 4–5 elongate, subequal in length, each shorter than 3; 4 triangular in male; 6–10 transverse, subequal in length; 11 2× length 10, broadly rounded at apex; 1–5 punctate, glabrous; 6–11 setose. Pronotum: subquadrate; lateral margin dilated, straight for basal ¾, then rounding to anterior angle, canaliculate; anterior angle acute, slightly produced; posterior angle acute; anterior margin curved anteriorly; disc subconvex; surface deeply punctate, more dense laterally, sparse on disc; basal impression absent; pronotal length 2.3–2.7 mm; pronotal width 3.2–3.6 mm. Scutellum: pentagonal; impunctate. Elytron: lateral margin straight, smooth, narrowly margined; apex rounded, apical margin thickened; sutural angle without tooth; humerus rounded, not produced; slightly constricted behind humerus; faint carina present behind humerus along puncture row 7; disc flattened along suture; finely punctate-striate, rows converge and unite apically; pygidium finely punctate, rounded at apex; elytral length 8.4–9.0 mm; elytral width 4.0–4.4 mm. Venter: pro-, meso-, and metasterna impunctate; abdominal sterna punctate, each puncture with pale seta; suture between sterna 1 and 2 complete; last sternite with apical margin emarginate medially in male, sinuate in female. Leg: slender; punctate; tibia with fringe of setae on inner margin of apex. Total length: 11.0–11.7 mm.

**Figures 215–223.**
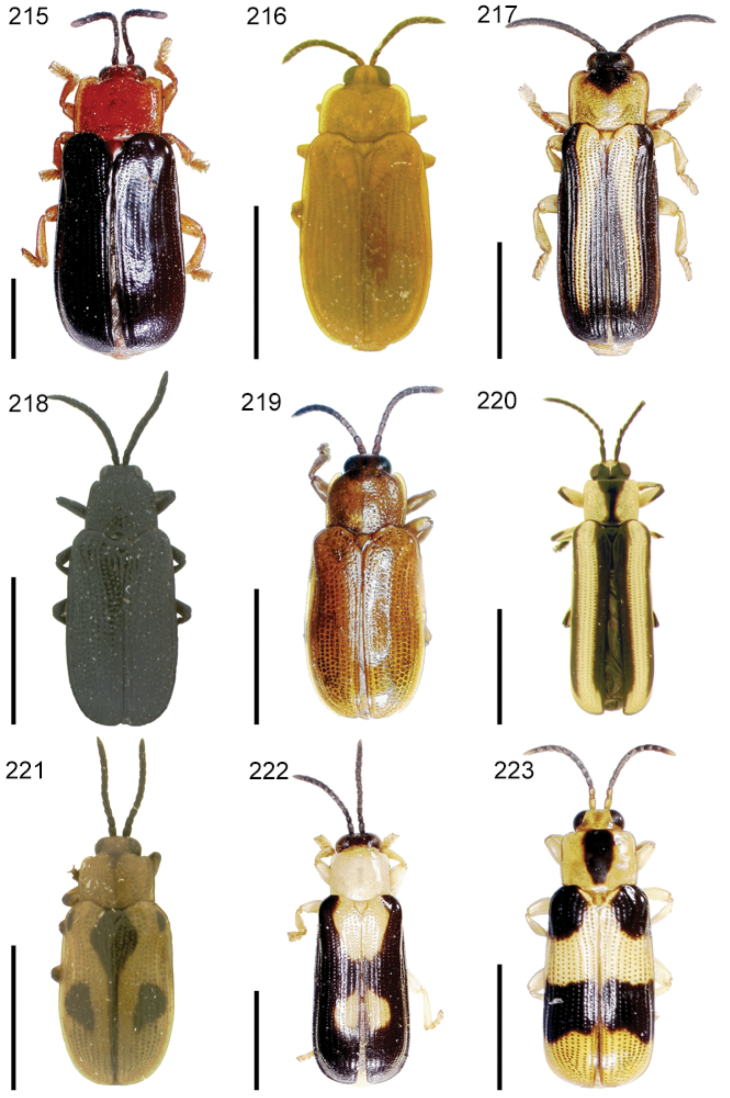
Habitus. **215** *Cephaloleia princeps* **216** *Cephaloleia proxima* **217** *Cephaloleia pulchella* **218** *Cephaloleia punctatissima* **219** *Cephaloleia puncticollis* **220** *Cephaloleia quadrilineata* **221** *Cephaloleia quinquemaculata* **222** *Cephaloleia recondita* **223** *Cephaloleia reventazonica*. Scale bars equal 3 mm.

##### Diagnosis.

This species is similar to *Cephaloleia abdominalis*, *Cephaloleia amazona*, *Cephaloleia steinhauseni*, *Cephaloleia susanae* sp. n., and *Cephaloleia teutonica*. It can be distinguished by the vertex of the head having a medial fovea and by the faint carina behind the humerus along puncture row 7 of the elytra.

##### Distribution.

Colombia, Ecuador, Peru.

##### Type material examined.

Holotype male: Peru, Fraser [handwritten label]/ Saunders Coll. [printed label]/ Cephalolia princeps Baly, Peru [blue handwritten label] (BMNH).

##### Specimens examined.

No label data (USNM). **Colombia:** Meta- Villavicencio, May 1946 (USNM). **Ecuador:** Morona Santiago- Macas (USNM). Imbabura- Cachabe to Paramba, February 1897 (USNM). Napo- Limonchcha, 7 June 1977 (USNM); Pununo, 20 August 1997 (CDFA, USNM);Limonchocha Reserve, 215 m, 10 August 1997 (CDFA); Sacha Lodge, 270 m, 22 March 1999 (SEMC, USNM), 3–13 April 1994, 23 March 1999 (SEMC); Shushufindi, 100 m, 11 August 1997 (CDFA); Sta. Cecilia, 340 m, 8 June- 1 August 1968 (SEMC). Orellana- 1 km S Onkone Gare Camp, Reserva Etnica Waorani, 216.3 m, 23 January 2006 (USNM); Yasuni area, 36 km S Pompeya, 15 August 1997 (USNM); Estación Cientifica Yasuni, 215 m, 5–10 November 1999 (EGRC). Pichincha- above Chimba, 3000’, August 1897 (USNM); Limonocha, 300 m, 31 March 1975 (EGRC); 10.6 km W Mindo, Mindo Road, 1375 m, 28 March 1999 (SEMC). **Peru:** no further data (ISNB). Total: 44.

#### Cephaloleia proxima

Taxon classificationAnimaliaColeopteraChrysomelidae

Baly, 1858

http://species-id.net/wiki/Cephaloleia_proxima

Fig. 216

Cephalolia proxima Baly 1858: 47. Gemminger and Harold 1876: 3602 (catalog); Donckier 1899: 550 (catalog); Weise 1911a: 9 (catalog), 1911b: 11 (catalog); Uhmann 1936b: 113 (noted), 1942b: 96 (noted).
Cephaloleia proxima Baly. Maulik 1916: 568 (museum list); Uhmann 1957b: 24 (catalog), 1964b: 16 (faunal list), 1965: 113 (museum list); Staines and Staines 1999: 524 (Baly species list).

##### Description.

Elongate; subparallel; subconvex; reddish-yellow; eyes and antennae (except antennomere 1) black. Head: vertex punctate, medial sulcus present; frons not projecting; not depressed between eyes. Antenna: as long as head and pronotum combined; slender; antennomere 1 slightly incrassate, elongate; 2 transverse, ½ length of 1; 3 elongate, slightly expanding apically, 2× length of 2; 4–10 elongate, subequal in length, each slightly longer than 2; 11 2× length of 10, pointed at apex; 1–3 punctate with scattered setae; 4–11 setose. Pronotum: transverse; lateral margin straight for basal 4/5 then rounding to anterior angle, canaliculate; anterior angle obtuse, produced; posterior angle acute; anterior margin emarginate behind head; disc subconvex, impunctate; surface finely punctate laterally; basal impression absent; pronotal length 1.2–1.4 mm; pronotal width 1.8–2.0 mm. Scutellum: pentagonal; impunctate. Elytron: lateral margin straight, smooth, canaliculate; apex rounded, serrate; sutural angle without tooth; humerus rounded, not produced; slightly constricted behind humerus; slightly flattened along suture; shallowly punctate-striate, rows converge and unite apically; elytral length 4.6–5.0 mm; elytral width 2.2–2.6 mm. Venter: pro-, meso-, and metasterna impunctate medially, punctate laterally; abdominal sterna punctate, each puncture with pale seta; suture between sterna 1 and 2 complete; last sternite with apical margin broadly emarginate, sinuate medially in male; broadly truncate-emarginate in female. Leg: slender; punctate; tibia with fringe of setae at apex. Total length: 5.6–6.8 mm.

##### Diagnosis.

This species is similar to *Cephaloleia apicicornis*, *Cephaloleia corallina*, *Cephaloleia halli*, and *Cephaloleia ochra* sp. n. It can be distinguished by the pronotum lacking a transverse basal impression and by antennomeres 2 to 11 being black.

##### Host plant.

According to the label data, adults have been collected feeding on *Heliconia* sp. (Heliconiaceae).

##### Distribution.

Brazil (Amazonas, Pará), French Guiana, Peru.

##### Type material examined.

Syntypes male, female: Amazons [handwritten label]/ Baly Coll. [printed label]/ Cephalolia proxima Baly, Amazons [blue handwritten label]; Cayenne [handwrttien label]/ Saunders Coll. [printed label] (BMNH, 2).

##### Specimens examined.

**Brazil:** Pará- Obidos (USNM). **French Guiana:** Cayenne (BMNH); Rte. D6, along Montage de Kaw Trail at 1 km N Camp Patawa, 7 April 1996 (AMNH); 23 km SE Roura, 1000 ft., 15–20 August 2012 (AJGC); Roura, 8.4 km SSE, 200 m, 28 May 1997 (USNM); vic. Saül, 830 ft., 8–14 August 2012 (AJGC); vic. Saül airport, 748 ft., 8–14 August (AJGC); 7 km WSW Tonate, 86 ft., 7–12 August 2012 (AJGC). **Peru:** ?- Middle Río Ucayali, 23 September 1923 (AMNH). Total: 35.

#### Cephaloleia pulchella

Taxon classificationAnimaliaColeopteraChrysomelidae

Baly, 1858

http://species-id.net/wiki/Cephaloleia_pulchella

Fig. 217

Cephalolia pulchella Baly 1858: 56. Gemminger and Harold 1876: 3602 (catalog); Donckier 1899: 550 (catalog); Weise 1911a: 9 (catalog), 1911b: 12 (catalog); Uhmann 1953d: 48 (faunal list).
Cephaloleia pulchella Baly. Waterhouse 1881: 261 (distribution); Uhmann 1957b: 24 (catalog); Staines and Staines 1999: 524 (Baly species list); McKenna and Farrell 2005: 119 (phylogeny), 2006: 10949 (phylogeny).

##### Description.

Narrowly elongate; subparallel; subdepressed; head and antennae (except for basal 2 antennomeres brownish) black, pronotum yellow (except for indistinct trilobed dark macula on anterior margin); elytra black with bright yellow longitudinal vitta medially on each elytron from base, narrowing behind, abbreviated near apex; scutellum and venter yellow. Head: vertex finely punctate, medial carina present, extending to between antennal bases; frons not projecting; depressed between eyes. Antenna: less that ½ body length; robust; antennomeres elongate; 1 subincrassate; 2 cylindrical, ½ length of 1, shortest; 3 subequal in length to 1; 4–10 decreasing in length, each shorter than 3; 11 2× length of 10, broadly rounded at apex; 1–2 punctate with scattered setae; 3–11 setose. Pronotum: quadrate; lateral margin straight then rounding to anterior angle, canaliculate; anterior angle rounded, slightly produced; posterior angle acute; anterior margin emarginate behind head; disc subconvex; surface deeply punctate, less dense on disc; basal impression absent; pronotal length 1.6–1.8 mm; pronotal width 2.0–2.2 mm. Scutellum: elongate; pentagonal; impunctate. Elytron: lateral margin straight, smooth, narrowly margined; apex subtruncate; sutural angle without tooth; humerus rounded, not produced; slightly constricted behind humerus; flattened along suture; declivity beginning just behind humerus at puncture row 7 not edged with faint carina; moderately punctate-striate, punctures more impressed laterally, confused at apex; interspaces sulcate laterally, 3 costate for entire length; elytral length 5.8–6.2 mm; elytral width 2.2–2.6 mm. Venter: pro-, meso-, and metasterna punctate; abdominal sterna punctate, each puncture with pale seta; suture between sterna 1 and 2 entirely obsolete; last sternite with apical margin broadly emarginate, slightly produced medially in male, truncate in female. Leg: slender; punctate; tibia with fringe of setae on inner margin of apex. Total length: 7.7–8.1 mm.

##### Diagnosis.

This species is similar to *Cephaloleia eximia* and *Cephaloleia saundersii*. It can be distinguished by the suture between abdominal sterna 1 and 2 being obsolete medially and by the pro-, meso-, and metasterna being punctate.

##### Distribution.

Peru.

##### Type material examined.

Syntype: Peru [handwritten label]/ Cephalolia pulchella Baly, Peru [blue handwritten label] (BMNH, 1).

##### Specimens examined.

?- 1920 (USNM). **Peru:**?- Chambireyacu, pres Yurimaguas, June-August 1885 (USNM); Huallaga (USNM). Total: 3.

#### Cephaloleia punctatissima

Taxon classificationAnimaliaColeopteraChrysomelidae

Weise, 1910

http://species-id.net/wiki/Cephaloleia_punctatissima

Fig. 218

Cephaloleia punctatissima Weise 1910: 94. Weise 1911a: 9 (catalog), 1911b: 13 (catalog).
Cephaloleia punctatissima Weise. Blackwelder 1946: 719 (catalog); Papp 1953: 21 (catalog); Uhmann 1957a: 24 (catalog); Wilcox 1983: 137 (catalog); Staines 1996: 52 (Central America species).

##### Description.

Elongate; subparallel; subconvex; shining; black. Head: vertex densely punctate laterally, medial sulcus absent; frons not projecting, densely punctate; slightly depressed between eyes. Antenna: reaches to humerus; slender; antennomeres 1, 3–5 cylindrical; 1 longer than 2, subequal to 3; 2 transverse; 3 elongate; 4–5 elongate, subequal in length, each shorter than 3; 6–10 transverse, subequal in length, each longer than 2; 11 longer than 10, pointed at apex; 1–2 punctate with scattered setae; 3–11 setose. Pronotum: subquadrate; lateral margin straight then rounding to anterior angle, canaliculate; anterior angle rounded, produced; posterior angle acute; anterior margin emarginate behind head; disc subconvex; surface strongly and densely punctate; basal impression absent; pronotal length 0.9 mm; pronotal width 1.6 mm. Scutellum: triangular; micropunctate. Elytron: lateral margin straight, smooth, narrowly margined; apex rounded, finely serrate; sutural angle without tooth; humerus rounded, not produced; slightly constricted behind humerus; moderately punctate-striate, rows converge and unite apically; elytral length 4.3 mm; elytral width 2.1 mm. Venter: metasternum and abdominal sterna punctate laterally. Leg: slender; punctate; tibia with fringe of setae on inner margin of apex. Total length: 5.4 mm.

##### Diagnosis.

This species is similar to *Cephaloleia gilvipes*. It can be distinguished by the black coloration and by the elytral puncture rows converging and uniting apically.

##### Biology.

Specimens have been collected sweeping vegetation.

##### Distribution.

Mexico.

##### Type material examined.

Holotype: Guadalajara 11 [printed label]/ Mexico, J. Flohr G. [green printed label]/ J. Weise det. [printed label]/ 247 [handwritten label]/ Type [red printed label]/ 95057 [handwritten label]/ Zool. Mus. Berlin [printed label]/ Cephalolia punctatissima m. [handwritten label] (ZMHB).

##### Specimens examined.

**MEXICO:** Jalisco- MX 80, 26 km S. Autlan, 18 July 2006 (BYUC). Total: 1.

#### Cephaloleia puncticollis

Taxon classificationAnimaliaColeopteraChrysomelidae

Baly, 1885

http://species-id.net/wiki/Cephaloleia_puncticollis

Fig. 219

Cephaloleia puncticollis Baly 1885: 12. Blackwelder 1946: 719 (catalog); Papp 1953: 21 (catalog); Uhmann 1957a: 24 (catalog); Wilcox 1983: 137 (catalog); Seifert and Seifert 1976a: 464 (biology), 1976b: 235 (biology), 1979a: 466 (biology); Strong 1977a: 160 (biology), 1977b: 580 (biology), 1983: 710 (biology); Seifert 1982: 8 (biology), 1984: 58 (biology); Maes and Staines 1991: 36 (faunal list); Staines 1996: 52 (Central America species), 1996(1997): 15 (Nicaragua species), 2004: 312 (host plants), 2010: 36 (types), 2011: 50 (faunal list); Staines and Staines 1997: 18 (types), 1999: 524 (Baly species list); Maes 1999: 1017 (faunal list); Jolivet and Verma 2002: 63 (noted); Jolivet 2003: 312 (noted); García–Robledo et al. 2010: 64 (noted).
Cephalolia puncticollis Baly. Donckier 1899: 551 (catalog); Weise 1911a: 9 (catalog), 1911b: 11 (catalog); Uhmann 1930a: 224 (faunal list), 1936a: 113 (noted).

##### Description.

Elongate; slightly dilated apically; subdepressed; reddish-brown, eyes and antennae darker. Head: vertex punctate, medial sulcus absent; frons not projecting; not depressed between eyes. Antenna: more than ½ body length; slender; antennomere 1 slightly thickened and compressed, longer than 2, subequal in length to 3; 2 transverse, shortest; 3 cylindrical, elongate; 4–10 transverse, subequal in length; 11 2× length of 10, pointed at apex; 1–3 punctate with scattered setae; 4–11 setose. Pronotum: ⅓ wider than long; lateral margin straight from base to near apex then rounding to anterior angle, margined; anterior angle obtuse, slightly produced; posterior angle acute, produced; anterior margin emarginate behind head; disc subdepressed; surface densely punctate; basal impression absent; pronotal length 1.0–1.1 mm; pronotal width 1.4–1.9 mm. Scutellum: broadly triangular; impunctate. Elytron: lateral margin straight, slightly dilated beyond middle, smooth, margined; apex rounded, margined; sutural angle without tooth; humerus rounded, slightly produced; slightly constricted behind humerus; flattened along suture; shallowly punctate-striate, rows converge and unite apically; elytral length 3.7–4.3 mm; elytral width 2.0–2.4 mm. Venter: pro-, meso-, and metasterna impunctate medially, punctate laterally; abdominal sterna punctate, each puncture with pale seta; suture between sterna 1 and 2 complete; last sternite with apical margin broadly emarginate medially in male, entire, rounded in female. Leg: slender; punctate, each puncture with pale seta; tibia with fringe of setae on inner margin of apex. Total length: 5.0–5.9 mm.

##### Diagnosis.

This species is similar to *Cephaloleia cylindrica* and *Cephaloleia sallei*. It can be distinguished by antennomeres 1 and 2 not being subglobose, by the punctate disc of the pronotum, and by the pro-, meso-, and metasterna being punctate laterally.

##### Host plant.

*Calathea insignis* Hort. and Bull. (Marantaceae) (Uhmann 1930a); *Heliconia imbricata* Benth. (Seifert and Seifert 1976a). Adults have been collected on *Heliconia latispatha* (Kuntze) Baker (Heliconiaceae), and *Musa* sp. (Musaceae) (Staines 1996).

##### Immatures.

Color when alive yellowish-white (Figs 47–50); when dead yellowish-brown, somewhat darker medially; venter paler than dorsum. Body ovate; flat. Dorsum with longitudinal ridge extending from anterior to posterior margin, with fringe of setae along margins. Pronotum with central area raised, micropustulate, lateral area rugose. Mesonotum with central area micropustulate, lateral area punctate. Metanotum with central area micropustulate, lateral areas punctate; with transverse sulcus near base; with transverse carina on each side. Abdominal tergites 1–6 narrowed medially, with transverse carina near lateral margin; spiracles appear as darker brownish macula without darker margin. Abdominal tergites 7–10 with transverse carina on each side. Venter: with surface of expansions rugose-striate, punctate. Head (Fig. 15) surface rugose-punctate, labrum with surface alutaceous, without setae; clypeus with fringe of setae at apex, with four setae on apical ½, surface striate; mandibles tridentate; maxillary palps with two palpomeres and 12 short, robust setae at apex; maxilla robust, clavate, with fringe of long setae at apex; labrum densely setose. Antenna (Fig. 17) with antennomere 1 robust, short; 2 robust, nearly subglobular, wider than 1, ½ length of 3; 3 elongate, cylindrical, with 10 setae at apex. Pro-, meso-, and metasterna wider than long; slightly depressed medially; surface rugose-striate; mesonotum longer than others. Abdominal sternites 1–8 wider than long, decreasing in length and width; with two sulci on apical ½; laterally with curved sulcus; sterna 9–10 fused, rounded at apex. Leg (Fig. 16) femur short, robust; tibiotarsus subconical, with a strong claw and 11 setae at apex. Total length 8.4–8.7 mm; total width 5.6–5.8 mm.

##### Biology.

Guthrie (2005) discussed the biology of this species.

##### Distribution.

Costa Rica, Nicaragua, Panama.

##### Type material examined.

Syntypes: V. de Chiriqui, 25–4000 ft., Champion/ F. Monros Collection 1959/ Cephaloleia puncticollis Baly, J. S. Baly det. [pink label] (USNM,1; AMNH, 3; ANSP, 2. Also- Bugaba, Panama, Champion USNM, 2: AMNH, 1; ANSP, 2).

##### Specimens examined.

**COSTA RICA:** Alajuela- San Ramón EB, 27 km N and 6 km W San Ramón, 7 July 2000 (SEMC). Cartago- La Palma, 1050 m, La Hondura, 20 June 1926 (USNM); Peralta, 400 m, 26 January 1933 (USNM); Turrialba (USNM), 29 May 1951, 15 July 1965 (USNM), 8–11 June 1980 (EGRC). Guanacaste- Cacao Biological Station, 11 July 2000 (SEMC); Est. Pitilla, 700 m, 9 km S Sta. Cecilia, 4–25 November 1991 (INBIO); Est. Queb. Bonita, 50 m, Res. Biol. Carara, 17 March- 30 April (INBIO); Est. Sirena, 0–100 m, P.N. Corcovado, September 1991 (INBIO); Tilarán, 7 July 1972, 30 July1972 (FSCA). Heredia- Finca La Selva nr Puerto Viejo, 24 July 1969, 4 August 1969 (USNM); La Selva Biol. Sta., 2 km S. Pt. Viejo, 3–5 June 1984 (EGRC), 10 June 2001, 03 July 2001 (USNM), 7 March 1965 (BYUC). Limón- Hamburg Farm, Reventazón, Ebene Limón, 27 January 1925, 1 January 1932 (USNM); Est. Hitoy Cerere, 100 m, R. Cerere, Res. Biol. Hitoy Cerere, June 1991 (INBIO); Pandora, 30 May 1962 (MUCR); Salvadora, Parismina Fluss, 5 October 1930, 19–31 December 1930 (USNM); Valle La Estrella, 100–200 m (INBIO); R.V.S. Gandoca Manzanillo, 0–100 m (INBIO). Puntarenas- Barranca near Puntarenas, 6 July 1972 (FSCA); 25 mi. S. Buenos Aires, 10 August 1969 (USNM); El Roble, 25 July 1929 (USNM); 10.9 E. Esparta, 17 June 1969 (USNM); Gulfo Dulce, Río Sandali, 21 August 1936 (USNM); Monte Verde, 26 March 1987 (USNM); 3 mi. S. Palmar Sur, 11 August 1969 (USNM); 5.4 mi. S. Palmar Sur, 11 August 1969 (USNM); 18 mi. S. Palmar Sur, 11 August 1969 (USNM); Puerto Cortes, 19 July 1972 (FSCA); 2.3 km N Río Catarata Bridge on Route 2, 250 m, 31 December 1989 (UMMZ); Río Piedras, 15 August 1969 (USNM); San Vito de Java, 20 July 1972 (FSCA); Sirena Corcovado, August 1993 (MUCR); Garabito, Reserva Biol Carara, Est Quebrada Bonita, 0–100 m (INBIO); A.C.O. Golfito, Pque Nal Corcovado, Est Sirena, 0–100 m (INBIO); Sirena Station, Corcovado National Park, lower Ollas Trail, 24–28 June 2000 (SEMC). San José- 12 mi. N. San Isidro del General, 26 June 1969 (USNM). **NICARAGUA:** Granada- Res. Nat. Volcán Mombacho, 1150 m, 3 June 1922 (USNM). Jinotega- SE Jinotega, 5100', 15 July 1974 (FSCA); 16 km N Matagalpa, Matagalpa-Jinotega Road, 22 May 2002 (SEMC). Malagalpa- 6 km N Malagalpa, Selva Negra Hotel, 1350 m, 20 May 2002 (SEMC, USNM). Río San Juan- 60 km SE San Carlos, Refugio Bartola, 30 May 2002 (SEMC). **PANAMA:** no further data (CASC); 11 April 1929, 17 November 1930, 12 January 1931, 26 January 1931, 16 February 1933, 13 April 1933, 22 November 1934 (CASC). ?- La Joya, December 1944 (CASC). Chiriquí- Galera de Chorcha, 3 July 1976 (EGRC); Hartmann's finca, St. Clara, 15–18 June 1985 (EGRC); Santa Clara, 23–25 May 1980 (EGRC); 2 km N Sta. Clara, 24–25 May 1977 (CMNC); Soledad to Fortuna, 16 May 1978 (EGRC). Panamá- Curundu, 13 March 1970 (EGRC); Ft. Sherman, 2 August 1974 (FSCA). Total: 423.

#### Cephaloleia quadrilineata

Taxon classificationAnimaliaColeopteraChrysomelidae

Baly, 1885

http://species-id.net/wiki/Cephaloleia_puncticollis

Fig. 220

Cephaloleia quadrilineata Baly 1885: 21. Calvert and Calvert 1917: 394 (noted); Blackwelder 1946: 719 (catalog); Papp 1953: 21 (catalog); Uhmann 1957a: 24 (catalog); Wilcox 1983: 137 (catalog); Staines 1996: 53 (Central America species), 1999: 241 (mimicry), 2004: 312 (host plants), 2010: 36 (types), 2011: 50 (faunal list); Staines and Staines 1997: 18 (types), 1999: 524 (Baly species list); McKenna and Farrell 2005: 119 (phylogeny), 2006: 10949 (phylogeny).
Cephalolia quadrilineata Baly. Donckier 1899: 551 (catalog); Weise 1911a: 9 (catalog), 1911b: 11 (catalog); Calvert and Calvert 1917: 394 (noted); Uhmann 1930a: 223 (faunal list), 1936a: 111 (noted), 1936b: 485 (key).

##### Description.

Elongate; subparallel; subdepressed; Head, antennae, and scutellum black; pronotum yellow, may have black medial vitta; elytra black with variable yellow markings- usually with vitta from suture to interspace 4 and lateral vitta from puncture row 6–9, puncture row 10 and lateral margin yellow; venter with prosternum yellow with black macula laterally, meso- and metasterna yellow medially, black laterally, abdominal sterna dark medially, yellow laterally; leg with femora yellow, tibiae and tarsi black. Head: vertex punctate near eyes, medial sulcus present, eyes protruding, finely faceted; frons finely, densely punctate, not projecting; slightly depressed between eyes. Antenna: reaches to humerus; slender; antennomere 1 elongate, robust, fringe of setae at apex; 2 subglobose, twice as long as 3; 3 triangular, shortest; 4 cylindrical, subequal in length to 2; 5–10 elongate, decreasing in length; 11 pointed at apex; 1–4 punctate with scattered setae; 5–11 setose. Pronotum: subquadrate; lateral margin straight then rounding to anterior angle, margined; anterior angle subobtuse, slightly projecting; posterior angle acute; anterior margin weakly emarginate behind head; flattened on disc; surface with scattered punctures near lateral margin and medially at base; basal impression absent; pronotal length 1.08–1.31 mm; pronotal width 1.16–1.54 mm. Scutellum: long, acutely triangular; shiny; alutaceous. Elytron: lateral margin slightly expanding apically, smooth, margined; apex rounded; sutural angle without tooth; humerus rounded, not produced; slightly constricted behind humerus; declivity beginning just behind humerus at puncture row 7 not edged with faint carina; shallowly punctate-striate, punctures obsolete laterally and apically; elytral length 4.3–5.1 mm; elytral width 1.7–2.4 mm. Venter: prosternum impunctate; meso- and metasterna impunctate medially, punctate laterally; abdominal sterna punctate, each puncture with pale seta; suture between sterna 1 and 2 complete; last sternite with apical margin emarginate medially in male, rounded, entire in female. Leg: slender; punctate; femur robust; tibia expanding apically, with fringe of setae on inner margin of apex. Total length: 5.6–6.9 mm.

##### Diagnosis.

This species is similar to *Cephaloleia nevermanni*. It can be distinguished by the elytra expanding apically.

##### Host plant.

Adults have been collected on *Heliconia* sp. (Calvert and Calvert 1917); *Heliconia imbricata* (Kuntze) Baker and *Heliconia latispatha* Benth. (Heliconiaceae) (Staines 1996).

##### Distribution.

Costa Rica, Panama.

##### Type material examined.

Lectotype: Type H. T. [white disk with red border]/ V. de Chiriqui 25–4000 ft. Champion/ B. C. A., Col. VI, 2. Cephaloleia quadrilineata, Baly/ Cephaloleia quadrilineata Baly/ Lectotype Cephaloleia quadrilineata Baly, Des. C. L. Staines 1993 [red label] (BMNH).

##### Specimens examined.

**COSTA RICA:** Alajuela- Upala Dos Ríos, 1 November 1937 (MUCR); Upala, Sector San Ramón de Dos Ríos, 1.5 km NW Hacienda Nueva Zelandia, 600–700 m (INBIO). Cartago- La Palma, 30 April 1928 (USNM); R. Dos Amigos, 1450 m, P.N. Tapantí, September 1992 (INBIO); Turrialba (DEI), 26 June 1951 (USNM); Quebrada Segunda, P. N. Tapantí, 1250 m, August 1991, 1–21 March 1992, April 1992, August 1992, September 1992 (INBIO); Turrialba, Tayutic, Grano de Oro, Chirripo, 1100–1200 m (INBIO). Guanacaste- Est. Pitilla, Volcán Orosí, 17 June 1969 (MUCR); Est. Pitilla, 700 m, 9 km S Sta. Cecilia, P. N. Guanacaste, September 1991 (INBIO); Río San Lorenzo, 1050 m, Tierras Morenas, Z. P. Tenorio, 21 April 1992, March 1993 (INBIO). Heredia- Finca La Selva nr. Puerto Viejo, 24 July 1969 (USNM), 7 March 1965 (BYUC); Río Ciruela, Perrosati, 1400 m, May 1990 (INBIO). Limón- Cerro Tortuguero, 0–120 m, P.N. Tortuguero, September 1991 (INBIO); Sector Cerro Cocorí, Fca. de E. Rojas, 150 m, September 1991, October 1991, November 1991 (INBIO); Limón, 4 February 1989, 5 February 1989 (MUCR); Oliva-Sixaola, 28 December 1980 (MUCR); Pococí, P.N. Colorado, Estación Cuatro Esquinas (INBIO); A.C. Llanuras del Tortuguero, Pococí, Río Sardinas, 0–100 m (INBIO). Puntarenas- 5 km S. Buenos Aires, 15 August 1969 (USNM); Coronado, 1400–1500 m, February 1925 (DEI); El Roble, 25 July 1929 (DEI, USNM); Est. Biol., Las Alturas, 1500 m, Coto Brus., November 1991, December 1991, January 1992, 1–21 March 1992, August 1992, 3–4 September 1992 (INBIO); Estación Boscosa, Peninsula de Osa, 15 September 1991 (INBIO); Est. Queb., Bonita, 50 m, Res. Bio. Carara, 17 March- 30 April (INBIO); Fca. Cafrosa, Est. Las Mellizas, P. N. Armistad, November 1989 (INBIO); Los Alturas, 1500 m, 23–26 May 1992 (AJGC); Monteverde, 9 July 1972 (FSCA), 26 May- 3 June 1984 (EGRC); Fca. Las Cruces, San Vito de Java, 11–14 August 1969 (USNM); F. Las Cruces, 6 mi. S. San Vito, 1200–1400 m, 21–25 August 1976 (CASC); Monteverde, 1400 m, 28 May 1979 (CMNC); Palo Seco, 31 December 1923 (DEI); Peninsula de Osa, 4 March 1968, 9 July 1968, 31 July 1968 (MUCR); Rincón de Osa, 22 July 1969 (MUCR); Río Claro, sea level, 19 August 1969 (USNM); San Vito de Java, 20 July 1972 (FSCA); San Luis, 1040 m, R.B. Monteverde, September 1992 (INBIO); San Vito Las Cruces, 20 November 1988 (INBIO); Est. Sirena, 0–100 m, P. N. Corcovado, December 1989, January 1990, November 1991, December 1991 (INBIO); Rancho Quemado, Peninsula de Osa, 200 m, October 1991, April 1992 (INBIO); 1.5 mi. S. Palmar Sur, 11 August 1969 (USNM), 5 mi. S. Palmar Sur, 15 August 1969 (USNM); Aguirre, Quepos, P.N. Manuel Antonio, 0–100 m (INBIO); 1 km SW Cerro Biolley, Sector Altamira, 1300–1400 (INBIO); Estación Pittier, 4.2 km SW Cerro Gemelo, 1600–1700 m (INBIO); Coto Brus, Sabalito, Finca Cafrosa, 2 km NW Mellizas, 1200–1300 m (INBIO); A.C.L.A.P. Coto Brus, San Vito, Las Cruces, 1200–1300 m (INBIO); Golfito, Jiménez, Albergue Cerro de Oro, 100–200 m (INBIO); A.C.O. Golfito, Reserva Ftal Golfo Dulce, Est Agujas, 200–300 m (INBIO); Guacimal, Finca Buen Amigo, Monteverde, 1000–1100 m (INBIO); Bosque Eterno de los Niños, Sector Monteverde, 1500–1600 m (INBIO); Estación La Casona, Las Torres, 1500–1600 m (INBIO). San José- Hda Tiquires, 150 m, Río Tiquires, 28 March 1988 (INBIO); San José (USNM). **PANAMA:** Chiriquí- Bugaba (AMNH); 2 km W Cerro Punta, 1720 m, 7 January 1977 (CMNC); El Valle del Nubes, 12 km NW Rovira, 4000 feet, 20 January 1960 (CDFA); Hartman's finca, St. Clara, 15–18 June 1985 (EGRC); Las Lagunas, 4 km W. Hato de Volcán, 1360 m, 24 May 1973 (EGRC); Lino (DEI); Santa Clara, 5 July 1976, 23–25 May 1980 (EGRC); 2 km N Sta. Clara, 1300 m., Hartmann’s Finca 20. May 1977 (CMNC); Tole (BMNH); V. de Chiriquí (AMNH, DEI, USNM). Total: 286.

#### Cephaloleia quinquemaculata

Taxon classificationAnimaliaColeopteraChrysomelidae

Weise, 1910

http://species-id.net/wiki/Cephaloleia_quinquemaculata

Fig. 221

Cephalolia quinquemaculata Weise 1910: 91. Weise 1911a: 9 (catalog), 1911b: 12 (catalog); Uhmann 1936b: 113 (noted), 1953d: 48 (faunal list).
Cephaloleia quinquemaculata Weise. Uhmann 1957b: 24 (catalog).

##### Description.

Moderately elongate; subparallel; subconvex; shining; pale yellowish; eyes, antennae, scutellum, elytral suture and five elytral maculae darker; venter with pro-, meso-, and metasterna reddish-yellow medially, black laterally, abdominal reddish-brown; legs reddish-yellow with tibio-femoral joint darker. Head: vertex irregularly, finely punctate, medial sulcus absent; frons not projecting; not depressed between eyes. Antenna: reaches beyond humerus; slender; antennomere 1 incrassate, elongate; 2 cylindrical, ½ length of 1; 3 longer than 2, slightly widening apically; 4–10 transverse, subequal in length, each shorter than 2; 11 2× length of 10, pointed at apex; 1–3 punctate with scattered setae; 4–11 setose. Pronotum: transverse; lateral margin straight then rounding to anterior angle, canaliculate; anterior angle rounded, not produced; posterior angle acute; anterior margin curved anteriorly; disc subconvex; surface strongly punctate except middle line impunctate; basal impression absent; pronotal length 1.3 mm; pronotal width 1.6 mm. Scutellum: pentagonal; impunctate. Elytron: lateral margin straight, smooth, narrowly margined; apex broadly rounded; sutural angle without tooth; humerus rounded, not produced; slightly constricted behind humerus; shallowly punctate-striate, rows converge and unite apically; elytral length 4.0 mm; elytral width 2.2 mm. Venter: pro-, meso-, and metasterna punctate; abdominal sterna irregularly punctate, each puncture with white seta; suture between sterna 1 and 2 obsolete medially. Leg: slender; punctate, each puncture with pale seta; tibia with fringe of setae on inner margin of apex. Total length: 5.5 mm.

##### Diagnosis.

This species is similar to *Cephaloleia kolbei* and *Cephaloleia sagittifera*. It can be distinguished by the lateral margin of the pronotum being canaliculate and straight but not divergent.

##### Distribution.

Peru.

##### Type material examined.

Syntype: Peru, Madre de Dios [green printed label]/ J. Weise det. [printed label]/ Type [salmon-colored printed label]/ Cephalolia 5maculata m [handwritten label] (ZMHB, 1).

##### Specimens examined.

**Peru:** Madre de Dios- Cocha Coshu Biol. Stn., Manu National Park, 350 m, 17–19 October 2000 (SEMC). Total: 1.

#### Cephaloleia recondita

Taxon classificationAnimaliaColeopteraChrysomelidae

Pic, 1923

http://species-id.net/wiki/Cephaloleia_recondita

Fig. 222

Cephalolia recondita Pic 1923: 9. Uhmann 1938a: 411 (noted), 1942b: 98 (noted).
Cephaloleia recondita Pic. Uhmann 1957b: 24 (catalog); Descarpentries and Villiers 1959a: 139 (types).

##### Description.

Elongate; subparallel; subdepressed; shining; pronotum, scutellum, venter, and legs yellowish-brown; antennae, vertex of head, and mouthparts black; elytra black with yellowish-brown maculae- a heart-shaped macula near scutellum reaching to puncture row 5 and extending to near middle along suture and a transverse nearly rectangular band just behind middle from suture to puncture row 5. Head: vertex with very fine punctures, with weak medial sulcus; frons not projecting; slightly depressed between eyes. Antenna: reaches to humerus; slender; antennomere 1 robust, elongate, cylindrical, 2× length 2; 2 transverse, shortest; 3–10 elongate, cylindrical, subequal in length; 11 longer than 10, acutely pointed at apex; 1–2 punctate with scattered setae; 3–11 setose. Pronotum: slightly transverse; lateral margin straight for basal 4/5 then rounding to anterior angle, margined; anterior angle rounded, not produced; posterior angle acute; anterior margin weakly emarginate behind head; disc subconvex, impunctate; surface with scattered, shallow punctures laterally and basally; basal impression absent; pronotal length 1.5–1.7 mm; pronotal width 1.7–1.9 mm. Scutellum: elongate pentagonal; impunctate. Elytron: lateral margin straight, smooth, margined; apex rounded; sutural angle without tooth; humerus rounded, slightly produced; slightly constricted behind humerus; declivity beginning just behind humerus at puncture row 7 not edged with faint carina; moderately punctate-striate, rows converge and unite apically; pygidium densely setose; elytral length 5.9–6.2 mm; elytral width 2.4–2.6 mm. Venter: pro-, meso-, and metasterna impunctate; abdominal sterna sparsely punctate, each puncture with pale seta; suture between sterna 1 and 2 obsolete medially; last sternite with apical margin emarginate medially in male, rounded in female. Leg: slender; sparsely punctate; tibia with fringe of setae on inner margin of apex. Total length: 7.8–8.2 mm.

##### Diagnosis.

This species is similar to *Cephaloleia bifasciata* and *Cephaloleia hnigrum*. It can be distinguished by the black elytra with yellowish-brown maculae.

##### Distribution.

Colombia, Ecuador.

##### Type material.

Type: Ecuador (MNHN, not seen).

##### Specimens examined.

**Colombia:** Valle de Cauca- Río Dagua (USNM). **Ecuador:** no further data (MNHN). Esmeraldas- 31.7 km NW Lita, 620 m, 23 August 1997 (CDFA, USNM). Pichincha- Chimba, 1000 feet, August 1897 (USNM). Total: 6.

#### Cephaloleia reventazonica

Taxon classificationAnimaliaColeopteraChrysomelidae

Uhmann, 1930a

http://species-id.net/wiki/Cephaloleia_reventazonica

Fig. 223

Cephalolia reventazonica Uhmann 1930a: 226.
Cephaloleia reventazonica Uhmann. Blackwelder 1946: 719 (catalog); Uhmann 1950b: 336 (type), 1957a: 24 (catalog); Papp 1953: 21 (catalog); Gaedike and Döbler 1971: 258 (types); Wilcox 1983: 137 (catalog); Staines 1996: 54 (Central America species), 1996(1997): 16 (Nicaragua species), 1997: 414 (Uhmann species list), 2004: 312 (host plants), 2011: 50 (faunal list); Staines and Staines 1997: 19 (types); Maes 1999: 1017 (faunal list); McKenna and Farrell 2005: 119 (phylogeny), 2006: 10949 (phylogeny); García–Robledo et al. 2013a: 3 (biology).

##### Description.

Narrowly elongate; subparallel; subconvex; pale yellow; antennae, except antennomeres 1 and 11 black; pronotum with black subtriangular macula which extends from anterior margin ¾ way to basal margin and anterior and basal margins black; elytra with lateral margin black, humeral macula subquadrate, from puncture row 2 to lateral margin black, medial macula from suture to lateral margin black; venter and legs pale yellow. Head: vertex impunctate, fine medial sulcus present; frons not projecting; not depressed between eyes. Antenna: reaches to humerus; slender; antennomere 1 elongate, subequal in length to 3; 2 transverse, ⅓ length of 1; 3 elongate; 4–10 transverse, subequal in length, each shorter than 3, longer than 2; 11 elongate, 2× length of 10, rounded at apex; 1–3 punctate with scattered setae; 4–11 setose. Pronotum: subquadrate; lateral margin straight for basal ¾ then rounding to anterior angle, margined; anterior angle rounded, not produced; posterior angle angulate; anterior margin straight; disc subconvex; surface nearly impunctate; basal impression absent; pronotal length 1.3–1.4 mm; pronotal width 1.6–1.9 mm. Scutellum: acutely triangular; impunctate. Elytron: lateral margin straight, smooth, margined; apex rounded; sutural angle without tooth; humerus rounded, not produced; slightly constricted behind humerus; declivity beginning just behind humerus at puncture row 7 not edged with faint carina; moderately punctate-striate, rows converge and unite apically; elytral length 3.8–4.1 mm; elytral width 1.9–2.0 mm. Venter: pro-, meso-, and metasterna impunctate; abdominal sterna punctate, each puncture with pale seta; suture between sterna 1 and 2 complete; last sternite with apical margin truncate in male, rounded in female. Leg: slender; sparsely punctate; tibia dentate at apex, with fringe of setae on inner margin of apex. Total length: 6.1–7.1 mm.

##### Diagnosis.

This species is similar to *Cephaloleia fenestrata*, *Cephaloleia histrionica*, and *Cephaloleia stainesi* sp. n. It can be distinguished by the suture between abdominal sterna 1 and 2 being complete, by the nearly impunctate pronotum, by the vertex of the head being impunctate, by antennomeres 1–4 not compressed laterally, and by the maculate pronotum.

##### Host plant.

Adults have been collected feeding on *Heliconia lathispatha* (Staines 1996); *Heliconia pogonantha* Cufod., *Calathea lutea* G. Mey., *Cephaloleia marantifolia* Standl. (Marantaceae) (García–Robledo et al. 2013a); *Heliconia psittacorum* Sw. (Heliconiaceae).

##### Distribution.

Costa Rica, Nicaragua.

##### Type material examined.

Paralectotype female: Costa Rica, Hamburg Farm [green printed label]/ Ebene Limon, 27.I.21, F. Nevermann [reversed green label]/ Paratypus [red printed label]/ Det Uhmann/ Cephalolia reventazonica Uh. 30 (DEI).

##### Specimens examined.

**COSTA RICA:** Alajuela- 1 km S Cariblanco, 20 May 1992 (CDFA); 1 km. S Cariblanco, 30 May 1992 (USNM); Res. For. San Ramón, 9 March 1990 (MUCR); Estación Eladios, 700–800 m (INBIO); Río San Lorencito, 5 km N Colonia Palmareña, 900–1000 m (INBIO); Upala, Sector San Ramón de Dos Ríos, 1.5 km NW Hacienda Nueva Zelandia, 600–700 m (INBIO). Cartago- Grano de Oro, 1120 m, Chirripo, Turrialba, 8–30 August 1992 (INBIO); Turrialba, 28 May 1951, 4–13 August 1970 (USNM). Guanacaste- Estac. Cacao, 1000–1400 m, SW side Volcán Cacao, 1988–1989 (INBIO); Río San Lorenzo, 1050 m, Tierras Morenas, Z. P. Tenorio, March 1990, 29 March- 21 April 1992, July 1992, August 1992 (INBIO); Turrialba, Santa Teresita, Monumento Nacional Guayabo, 1100–1200 (INBIO). Heredia- Est. El Ceibo, Braulio Carillo N. P., 400–600 m, November 1989 (INBIO); Est. Biol. La Selva, 50 m, 1 April 1990 (INBIO), 6 March 1965 (BYUC); Finca La Selva, Sarapiquí, June 1972 (FSCA); F. La Selva, 3 km SE P. Viejo, 30 March 1990 (USNM); Finca La Selva, 20 August 1968, 21–30 July 1976 (USNM); Fca. La Selva, nr. Puerto Viejo, 24 July 1969, 5 August 1969 (USNM). Limón- Amubri, 70 m, Talamanca, 16–31 August 1992, 12–29 November 1992 (INBIO); Bananito, 20 April 1925 (USNM); Sector Cerro Cocorí, Fca. de E. Rojas, 150 m, June 1991, October 1991, November 1991, January 1992, 31 January- 21 February 1992, March 1992, 26 March- 24 April 1992, April 1992, 28 May- 17 June 1992, June 1992, 26 June- 16 July 1992, 12–31 August 1992, 9–30 November 1992, January 1993, February 1993, March 1993, April 1993, May 1993 (INBIO); Guápiles, 17 February 1924 (DEI); Hamburg Farm, Reventazón, Ebene Limón, 15 November 1923, 12 February 1925, 12 March 1925 (USNM), 15 May 1924, 21 August 1925 (DEI), 1 January 1932 (MUCR); Est. Hitoy Cerere, 100 m, R. Cerere, Res. Biol. Hitoy Cerere, October 1990, 6–25 November 1991, 4–20 December 1991, 7–26 January 1992 19–29 April 1992, 30 June- 20 July 1992, November 1992, December 1992, 15–27 February 1993 (INBIO); Manzanillo, 0–100 m, RFNS, Gandoca y Manzanillo, 7–19 August 1992, 9 September- 13 October 1992, 22 October- 11 November 1992, 5–13 December 1992, 6–27 January 1993 (INBIO); Río Sardinas, 10 m, R.N.F.S., Barra del Colorado, September 1992 (INBIO); Salvadora Farm, Parismina, 10 September 1930 (USNM), 19–31 December 1930 (DEI); Waldeck, 22 July 1936 (USNM); Valle La Estrella, 100–200 m (INBIO); Est Miramar, R. B. Hitoy Cerere, 300–400 m (INBIO). Puntarenas- Est. Biol. Las Alturas, 1500 m, Coto Brus., June 1991, 1–21 March 1992, August 1992, 3–4 September 1992 (INBIO); Est. La Casona, 1521 m, Res. Biol. Monteverde, April 1992 (INBIO); Monteverde Cloud For., 27–31 May 1984 (EGRC); Monteverde Cloud For. Res., 1300 m, 17–20 May 1985 (EMEC); Osa Peninsula, 2.5 mi SW Rincón, 1–7 March 1967 (USNM); San Luis, 1040 m, R.B. Monteverde, October 1992, 24 August- 15 September 1992 (INBIO); Estación Altamira, 1 km S Cerro Biolley, 1400–1500 m (INBIO); Finca Cafrosa, El Embalse, 1300–1400 m (INBIO); Guacimal, Finca Buen Amigo, Monteverde, 1000–1100 m (INBIO); Reserva Bosque Eterno de los Niños, Sector Monteverde, 1500–1600 m (INBIO). **Nicaragua:** Atlantico Norte- Musawas, Waspuc River, 27 September 1955 (EMEC). Total: 278.

#### Cephaloleia rosenbergi

Taxon classificationAnimaliaColeopteraChrysomelidae

Weise, 1905b

http://species-id.net/wiki/Cephaloleia_rosenbergi

Fig. 224

Cephalolia rosenbergi Weise 1905b: 55. Weise 1911a: 9 (catalog), 1911b: 10 (catalog); Uhmann 1936b: 110 (noted), 1938a: 408 (noted).
Cephaloleia rosenbergi Weise. Uhmann 1957b: 24 (catalog); Descarpentries and Villiers 1959a: 139 (types).

##### Description.

Elongate; subparallel; depressed; yellowish with dark markings; antennae, head, medial longitudinal pronotal vitta, scutellum, elytral base and apex dark; venter with pro-, meso-, and metasterna reddish-yellow medially, dark laterally, abdominal sterna dark medially, pale laterally; legs dark except basal ¾ of femora. Head: vertex sparsely, irregularly punctate, medial sulcus present, sulcus present near eye; frons not projecting; depressed between eyes. Antenna: reaches to humerus; slender; antennomere 1 thick, transverse, shortest; 2 elongate, subequal to 4; 3 elongate, longer than 2; 2–4 triangularly produced in male, cylindrical in female; 5–10 elongate, subequal in length, each shorter than 4; 11 slightly longer than 10, pointed at apex; 1–3 punctate with scattered setae; 4–11 setose. Pronotum: transverse; lateral margin straight then rounding to anterior angle, narrowly margined; anterior angle slightly rounded, not produced; posterior angle subangulate; anterior margin straight; disc subconvex; surface sparsely, irregularly punctate; slight transverse basal impression present medially; pronotal length 1.4–1.6 mm; pronotal width 1.5–1.7 mm. Scutellum: triangular; punctate. Elytron: lateral margin straight, smooth, narrowly margined; apex rounded; sutural angle without tooth; humerus rounded, not produced; slightly constricted behind humerus; weakly punctate-striate, rows converge and unite apically; elytral length 5.6–6.0 mm; elytral width 2.1–2.4 mm. Venter: pro-, meso-, and metasterna impunctate medially, punctate laterally; abdominal sterna punctate, each puncture with pale seta; suture between sterna 1 and 2 complete; last sternite with apical margin bisinuate in male, rounded in female. Leg: slender; impunctate; tibia with fringe of setae on inner margin of apex. Total length: 7.5–8.0 mm.

**Figures 224–232.**
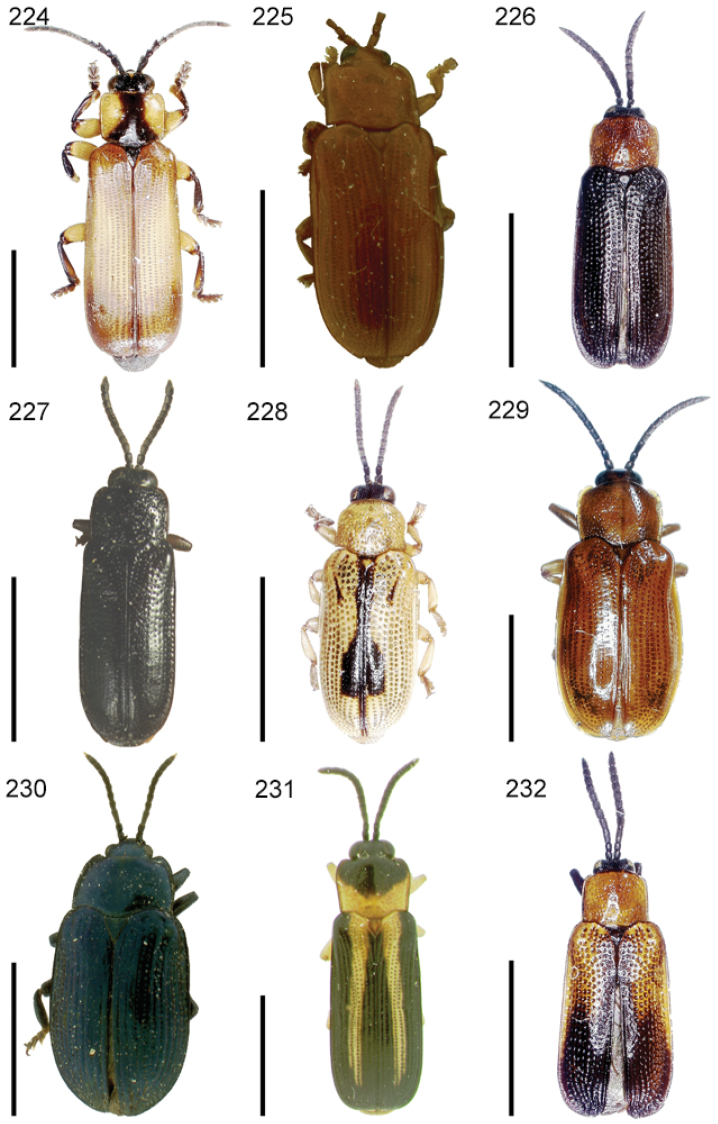
Habitus. **224** *Cephaloleia rosenbergi* **225** *Cephaloleia rubra* **226** *Cephaloleia ruficollis* **227** *Cephaloleia rufipes* **228** *Cephaloleia sagittifera* **229** *Cephaloleia sallei* **230** *Cephaloleia sandersoni* **231** *Cephaloleia saundersii* **232** *Cephaloleia schmidti*. Scale bars equal 3 mm.

##### Diagnosis.

This is the only South American species with a darker apical portion of the elytra and a black longitudinal vitta on the pronotum.

##### Distribution.

Ecuador.

##### Type material examined.

Syntype: Ecuador, Chimbo, 3000’, 8.1897, Rosenberg [printed label]/ I. Weise det. [printed label]/ Type [printed salmon-colored label]/ Cephalolia rosenbergi m. [handwritten label] (BMNH, 1).

##### Specimens examined.

**Ecuador:** no further data (USNM). Imbabama- Cachabe, January 1897 (USNM). Los Ríos- Río Palenque, 47 km S Sto. Domingo, 220 m, 26 August 1997 (CDFA, USNM). Pichincha- Chimbo (ZMHB, MNHN); above Chimbo, 3000’, August 1897 (USNM); Estación Orongo, Palmitopomba, 9 November 1999, 23 July 2001, 6 September 2002 (USNM); Reserva Maquipucuna, Cerro de Nanegal, 18 July 2001 (USNM). Total: 30.

#### Cephaloleia rubra

Taxon classificationAnimaliaColeopteraChrysomelidae

Staines, 1996

http://species-id.net/wiki/Cephaloleia_rubra

Fig. 225

Cephaloleia rubra Staines 1996: 55. Staines 2008: 2 (key), 2009a: 21 (noted).

##### Description.

Elongate; subparallel; subdepressed; reddish-brown, eyes and apex of antennae blackish. Head: vertex sparsely punctate, medial sulcus absent; strong keel present between antennal bases; frons not projecting; slightly depressed between eyes. Antenna: reaches to humerus; slender; antennomere 1 incrassate, with projection on outer apical angle; 2 transverse, ⅓ length of 1; 3 widening apically, subequal in length to 2; 4–10 transverse, subequal in length, each shorter than 3; 11 2× length of 10, pointed at apex; 1–3 punctate with scattered setae; 4–11 setose. Pronotum: transverse; lateral margin straight for basal ¾ then rounding to anterior angle, margined; anterior angle rounded, produced; posterior angle acute; anterior margin emarginate behind head; disc flattened; surface sparsely, shallowly punctate; basal impression absent; pronotal length 1.1–1.3 mm; pronotal width 1.4–1.9 mm. Scutellum: pentagonal, micropunctate. Elytron: lateral margin straight, smooth, margined; apex rounded; sutural angle without tooth; humerus rounded, not produced; slightly constricted behind humerus; shallowly punctate-striate, rows converge and unite apically; elytral length 3.7–4.4 mm; elytral width 2.1–2.3 mm. Venter: prosternum impunctate medially, rugose laterally; meso- and metasterna impunctate medially, punctate laterally; abdominal sterna punctate, each puncture with pale seta; suture between sterna 1 and 2 obsolete medially. Leg: slender; tibia with fringe of setae on inner margin of apex. Total length: 5.0–6.0 mm.

##### Diagnosis.

This species is similar to *Cephaloleia brunnea*. It can be distinguished by the sparsely punctate pronotum and by antennomere 1 being 3 times the length of 3.

##### Distribution.

Trinidad.

##### Type material examined.

Holotype: Morne Bleu, 2700', Trinidad, W. I., Aug. 15, 1969, H. and A. Howden/ Holotype Cephaloleia rubra Staines, Des. C. L. Staines 1994 [red label] (CMNC).

##### Specimens examined.

**TRINIDAD:** N. Range Arima-Blanchisseuse rd., Textel, nr. Morne Bleu, 12 May 1985 (EGRC). Total: 2.

#### Cephaloleia ruficollis

Taxon classificationAnimaliaColeopteraChrysomelidae

Baly, 1858

http://species-id.net/wiki/Cephaloleia_ruficollis

Fig. 226

Cephalolia ruficollis Baly 1858: 165. Gemminger and Harold 1876: 3602 (catalog); Donckier 1899: 551 (catalog); Weise 1905a: 132 (noted), 1911a: 9 (catalog), 1911b: 12 (catalog).
Cephaloleia ruficollis Baly. Baly 1885: 24 (distribution); Blackwelder 1946: 720 (catalog); Papp 1953: 21 (catalog); Uhmann 1957a: 25 (catalog); Wilcox 1983: 137 (catalog); Staines 1996: 56 (Central America species); Staines and Staines 1999: 524 (Baly species list); Flowers and Hanson 2003: 51 (distribution), 2011: 50 (faunal list); McKenna and Farrell 2005: 121 (phylogeny), 2006: 10949 (phylogeny); Naczi and Staines 2011: 3 (faunal list).

##### Description.

Elongate; subparallel; subconvex; head, antennae, elytra, and venter black; pronotum red. Head: vertex densely punctate, medial sulcus absent; frons not projecting; slightly depressed between eyes. Antenna: reaches to humerus; slender; antennomere 1 clavate, subequal in length to 3; 2–3 elongate, 2 shorter than 1 or 3; 4–10 transverse, decreasing in length; 11 longer than 10, rounded at apex; 1–2 punctate; 3–11 setose. Pronotum: subquadrate; lateral margin straight for basal ½ then rounding to anterior angle, narrowly canaliculate; anterior angle acute, produced; posterior angle angulate; anterior margin emarginate behind head; disc subconvex, slightly depressed behind middle; surface sparsely punctate, punctures subovate, large; pronotal length 1.0–1.1 mm; pronotal width 1.3–1.4 mm. Scutellum: pentagonal, micropunctate. Elytron: lateral margin nearly straight, smooth, margined; apex rounded, minutely serrulate, emarginate at suture; sutural angle without tooth; humerus rounded, not produced; slightly constricted behind humerus; subconvex, slightly flattened along suture; moderately punctate-striate, punctures large, punctures near apex smaller and less impressed than those on disc, rows converge and unite apically; elytral length 3.8–4.1 mm; elytral width 1.9–2.0 mm. Venter: pro-, meso-, and metasterna impunctate medially, punctate laterally; abdominal sterna 1–4 punctate laterally, each puncture with pale seta; suture between sterna 1 and 2 obsolete medially; last sternite with apical margin broadly truncate-emarginate, sinuate medially in male, truncate in female. Leg: slender; punctate, each puncture with pale seta; tibia with fringe of setae on inner margin of apex. Total length: 5.1–5.3 mm.

##### Diagnosis.

This species is similar to *Cephaloleia atriceps* and *Cephaloleia schmidti*. It can be distinguished by the unicolorous elytra, by the vertex of the head being depressed between the eyes, and by the suture between abdominal sterna 1 and 2 being obsolete medially.

##### Distribution.

Belize, Costa Rica, El Salvador, Guatemala, Honduras, Mexico, Nicaragua.

##### Type material examined.

Holotype male: Mexico, Tuxtla; San Andres, Sallé [printed label]/ Cephalolia ruficollis Baly, Mexico [blue handwritten label] (BMNH).

##### Specimens examined.

**BELIZE:** Cayo- Trek Stop (E. San José Succotz), 11–12 September 2002 (BYUC); Xunantunich, 14 August 1977 (BYUC). Orange Walk- Río Bravo Conservation Area, La Milpa, 17 July 1996 (BYUC). **COSTA RICA:** Cartago- Quebrada Segunda, 1200–1300 m (INBIO); Turrialba, 13 July 1976 (EMEC). Guanacaste- La Cruz, La Garita, Estación Los Almendros, 200–300 m (INBIO); La Cruz, Santa Elena, Cerro El Hacha, 12 km SE La Cruz, 200–300 m (INBIO). Heredia- Est. Biol. La Selva, April-May 1993 (MUCR). Limón- Llanuras del Tortuguero, Pococí, Río Sardinas, Barra del Colorado, 0–100 m (INBIO); Pococí, P.N. Colorado, Sector Cerro Cocorí, 30 km N Cariari, 100–200 m (INBIO); Talamanca, Amubri, 0–100 m (INBIO). Puntarenas- Monteverde Reserve, 1500 m, 24 May 1979, 18 August 1987, 19 August 1987, 24 August 1987, 23 August 1987, (CMNC); Monteverde area, 6–14 June 1973, 1400–1700 m (USNM); Orotina San Mateo, 29 February 1939 (MUCR); Garabito, Reserva Biol Carara, Est Quebrada Bonita, 0–100 m (INBIO); Reserva Bosque Eterno de los Niños, Sector Monteverde, 1500–1600 m (INBIO). **GUATEMALA:** Baja Verapaz- 5 km. S. San Jeronimo, 1250 m, 20 May 1991 (CMNC). Petén- Tikal, September 1959 (USNM). **HONDURAS:** Atlántida- 36 km E. Tela, 6 August 1977 (EGRC). Copán- Sta Rosa, 4000', 24 July 1974 (EGRC). **MEXICO:** no further data (DEI); at Nogales Arizona, 11 May 1976 (USNM). Chiapas- SE Trinitaria, 19 September 1981 (BYUC, SEMC); Tuxtla Gutierrez, 23 May 1952 (USNM), 11 July 1952 (EMEC). Colima- 11.3 mi. S. Colima, 20 August 1984 (FSCA). Guerrero- 9.0 km. NW. El Ocotito, 945 m, 7 July 1987 (TAMU). Jalisco- 9 mi SW Autlan, 4300 feet, hwy 8, 11 August 1982 (EGRC); 6 mi NE Barra de Navidad, 500', hwy 80, 11 August 1982 (EGRC). Morelas- Cañon de Lobos, km 19E Cuernavaca, 1220–1375 m, 3 July 1992 (EGRC). Oaxaca- Puerto Escondido, 15 July 1985 (TAMU); 3.9 mi. NE of San Gabriel Mixtepec, 16 July 1985 (TAMU); 7.6 km. S. San Gabriel Mixtepec, 762 m, 10 July 1987 (TAMU); Temescal, 6 July 1965 (EGRC); Vista Hermosa, 3 July 1982 (SMC). Oxaca- Vista Hermosa, 3 July 1982 (BYUC). Puebla- 17 mi N Zacapoaxtla, 4300 ft., 10 June 1983 (BYUC). San Luis Potosí- Tamazunchale, 20 May 1952 (AMNH). Sinaloa- 38 mi. NE Concordia nr. Loberas, 3 July 1982 (CDFA, USNM); 7 mi. E. Villa Unión, 9 August 1964 (CNC). Tampaulipas- Est. Biol. Las Cedras, Gomez-Farias, 350 m, 26–30 July 1993 (TAMU); El Cielito nr. Encino, 28–30 August 1985 (EGRC, USNM); along rd to Rancho de Cielo, 1–3 mi W Gomez Faries, 21 May 1979 (EGRC). Veracruz- Cordoba, 20 July 1966 (EMEC); Fortín de la Flores, 26–30 June 1963 (USNM), 27 June 1963 (FSCA); INEREP Botanical Gardens, Jalapa, 9 June 1983 (BYUC); Jalapa 21 May 1946, 22 May 1946 (AMNH). Yucatán- Sagil, July 1959 (USNM). **NICARAGUA:** Granda- Volcán Mombacho, Finca La Progresso, 21 June 1998 (USNM). Total: 103.

#### Cephaloleia rufipes

Taxon classificationAnimaliaColeopteraChrysomelidae

Pic, 1929b

http://species-id.net/wiki/Cephaloleia_rufipes

Fig. 227

Cephalolia rufipes Pic 1929b: 183. Bachmann and Cabrera 2010: 71 (types).
Cephaloleia rufipes Pic. Uhmann 1957b: 25 (catalog).

##### Description.

Elongate; subparallel; subdepressed; shining; black with legs reddish; venter covered with white pubescence (denser on abdomen). Head: vertex finely, densely punctate; medial sulcus absent; frons not projecting; depressed between eyes. Antenna: reaches to humerus; robust; antennomeres 1–2 subequal in length, slightly elongate; 3–5 elongate, subequal in length, each shorter than 2; 6–10 transverse, subequal in length; 11 2× length of 10, pointed at apex; 1–2 punctate with scattered setae; 3–11 setose. Pronotum: quadrate; lateral margin straight for basal ⅔ then rounding to anterior angle, narrowly margined; anterior angle rounded, not produced; posterior angle acute; anterior margin straight; disc subconvex; surface irregularly, sparsely punctate; transverse basal impression present medially; pronotal length 1.1 mm; pronotal width 1.4 mm. Scutellum: pentagonal; impunctate. Elytron: lateral margin straight, smooth, margined; apex subrounded; sutural angle without tooth; humerus rounded, not produced; slightly constricted behind humerus; strongly, irregularly punctate-striate at base, apex with punctures nearly obsolete; interspaces slightly raised; elytral length 3.8 mm; elytral width 1.6 mm. Venter: obscured by card. Leg: slender; punctate; tibia with fringe of setae on inner margin of apex. Total length: 5.0 mm.

##### Diagnosis.

This species is similar to *Cephaloleia coroicoana*, *Cephaloleia deplanata*, *Cephaloleia fiebrigi*, and *Cephaloleia marantae*. It can be distinguished by the basal impression on the pronotum and by the elytral punctures being obsolete basally.

##### Distribution.

Brazil.

##### Type material examined.

Holotype: Brazil, coll. Bruch [printed label]/ *Cephalolia* [handwritten label]/ *rufipes* sp. n. [handwritten label] (MACN).

#### Cephaloleia sagittifera

Taxon classificationAnimaliaColeopteraChrysomelidae

Uhmann, 1939

http://species-id.net/wiki/Cephaloleia_sagittifera

Fig. 228

Cephalolia sagittifera Uhmann 1939: 152.
Cephaloleia sagittifera Uhmann. Uhmann 1957b: 25 (catalog); Buck 1958: 146 (museum list); Gaedike and Döbler 1971: 359 (types); Staines 1997b: 414 (Uhmann species list); McKenna and Farrell 2005: 121 (phylogeny), 2006: 10949 (phylogeny).

##### Description.

Elongate; ovate; subconvex; shining; yellowish-brown; head, antennae (except antennomere 1 brownish) black; elytra with black sutural marking; venter with pro-, meso-, metasterna and abdominal sternite 1 yellowish-brown medially, black laterally, sterna 3–5 yellowish-brown. Head: vertex punctate, medial sulcus absent; frons not projecting; slightly depressed between eyes. Antenna: reaches to humerus; slender; antennomere 1 slightly incrassate, 2× length of 2; 2 subglobose, elongate; 3–5 cylindrical, subequal in length, each longer than 2; 6–10 transverse, subequal in length; 11 2× length of 10, broadly rounded at apex; 1–2 punctate with scattered setae; 3–11 setose. Pronotum: transverse; lateral margin sinuate at base, straight after middle then rounding to anterior angle, finely margined; anterior angle rounded, not produced; posterior angle acute; anterior margin straight; disc subconvex; surface densely punctate; basal impression absent; pronotal length 0.9–1.1 mm; pronotal width 1.2–1.4 mm. Scutellum: pentagonal; impunctate. Elytron: lateral margin straight, smooth, narrowly margined; apex broadly rounded; sutural angle without tooth; humerus rounded, not produced; slightly constricted behind humerus; moderately punctate-striate, rows converge and unite apically; elytral length 3.3–3.7 mm; elytral width 1.5–1.7 mm. Venter: pro-, meso-, and metasterna impunctate medially, punctate laterally; abdominal sterna punctate, each puncture with pale seta; suture between sterna 1 and 2 obsolete medially; last sternite with apical margin emarginate medially in male, truncate in female. Leg: slender; punctate, each puncture with pale seta; tibia with fringe of setae on inner margin of apex. Total length: 4.5–5.0 mm.

##### Diagnosis.

This species is similar to *Cephaloleia kolbei* and *Cephaloleia quinquemaculata*. It can be distinguished by the sinuate lateral margin of the pronotum.

##### Distribution.

Argentina, Brazil (Santa Catharina).

##### Type material examined.

Holotype male: Brazil, S. Catharina, Nova Teutonia, 5.X.1932, Plaumann [printed label]/ Holotypus [red printed label]/ Cephalolia sagittifera Uh., Det. E. Uhmann (DEI).

##### Specimens examined.

**Argentina:** Misiones- Dos de Mayo, November 1989 (USNM). **Brazil:** Santa Catharina- Nova Teutonia, 24 September 1932, 2 September 1935, 14 November 1935, 23 November 1935, 2 October 1936, 8 October 1936, 10 October 1936, 15 October 1936, 22 October 1936 (DEI), 6 January 1938, 17 September 1938, 19 September 1938, September 1968, October 1968 (USNM), September 1976 (EGRC). Total: 41.

#### Cephaloleia sallei

Taxon classificationAnimaliaColeopteraChrysomelidae

Baly, 1858

http://species-id.net/wiki/Cephaloleia_sallei

Fig. 229

Cephalolia sallei Baly 1858: 45. Gemminger and Harold 1876: 3602 (catalog); Donckier 1899: 551 (catalog); Weise 1910: 83 (noted), 1911a: 9 (catalog), 1911b: 11 (catalog); Uhmann 1942: 97 (noted).
Cephaloleia sallei Baly. Baly 1885: 12 (distribution); Champion 1894: 234 (distribution); Blackwelder 1946: 720 (catalog); Papp 1953: 21 (catalog); Uhmann 1957a: 25 (catalog); Wilcox 1983: 137 (catalog); Strong 1977a: 163 (host plants); Werren et al. 1995: 200 (disease); Staines 1996: 56 (Central America species), 2004: 312 (host plants), 2011: 50 (faunal list); Staines and Staines 1999: 524 (Baly species list); McKenna and Farrell 2005: 121 (phylogeny), 2006: 10949 (phylogeny); Meskins et al. 2008: 163 (host plants), 2011: 483 (food web); Descampe et al. 2008: 227 (host plants); García–Robledo et al. 2013a: 3 (biology).

##### Description.

Elongate; subparallel; subdepressed; reddish-yellow; eyes and antennae (except antennomere 1) black. Head: vertex sparsely punctate, medial sulcus present; keel present between antennal bases; frons not projecting; not depressed between eyes. Antenna: reaches to humerus; slender; antennomere 1 subincrassate, obovate, longer than 2; 2 transverse; 3–5 elongate, each subequal in length to 1; 6–10 transverse; 11 rounded at apex, 3× length of 10; 1–3 punctate with scattered setae; 4–11 setose. Pronotum: subquadrate; lateral margin nearly straight then rounding to anterior angle, margined; anterior angle obtuse, slightly produced; posterior angle acute; anterior margin weakly emarginate behind eyes; disc subconvex; surface with disc nearly impunctate, some punctures present laterally and basally; transverse basal impression present on each side of middle; pronotal length 1.1–1.4 mm; pronotal width 1.6–2.1 mm. Scutellum: elongate triangular, apex very acute; impunctate. Elytron: lateral margin straight, smooth, margined; apex obtusely rounded, smooth; sutural angle without tooth; humerus rounded, slightly produced; slightly constricted behind humerus; shallowly punctate-striate, rows converge and unite apically; elytral length 4.1–4.7 mm; elytral width 2.1–2.6 mm. Venter: pro-, meso-, and metasterna impunctate; abdominal sterna punctate, each puncture with pale seta; suture between sterna 1 and 2 complete; male with last sternite with apical margin concave, pygidium obtusely truncate; female with last sternite with apical margin bisinuate, slightly produced medially, pygidium obtuse, weakly bisinuate. Leg: slender; impunctate; femur robust; tibia dentate at apex, with fringe of setae on apex. Total length: 5.2–6.4 mm.

##### Diagnosis.

This species is similar to *Cephaloleia cylindrica* and *Cephaloleia puncticollis*. It can be distinguished by antennomeres 1 being subincrassate, by antennomere 2 being transverse, by the impunctate disc of the pronotum, and by the impunctate lateral margins of the pro-, meso-, and metasterna.

##### Host plant.

Adults have been collected on *Heliconia* sp. (Heliconiaceae) (Strong 1977a); *Renealmia strobilifera* Poepp. and Endl. (Zingiberaceae) (Staines 2004); *Heliconia irrasa* R. R. Smith (Heliconiaceae) (McKenna and Farrell 2005); *Calathea inocephala* (Kuntze), *Cephaloleia latifolia* Klotzsch, *Cephaloleia lutea* Schult., *Pleiostachya pruinosa* (W. Bull. ex. Regel) K. Schum. (Marantaceae), *Heliconia catheta* R. R. Smith, *Heliconia latispatha* Benth., *Heliconia mariae* Hook. (Meskins et al. 2008); *Heliconia vaginalis* Benth. (Heliconiaceae) (Descampe et al. 2008); *Costus laevis* Ruiz. and Pav. (Costaceae) (García–Robledo et al. 2013a); *Heliconia psittacorum* Sassy.

##### Distribution.

Costa Rica, Guatemala, Mexico, Panama.

##### Type material examined.

Syntype: Bogata [handwritten label]/, Baly coll. [printed label]/ Cephalolia sallei Baly, Bogata [blue handwritten label] (BMNH, 1).

##### Specimens examined.

**COSTA RICA:** Alajuela- R. B. San Ramón, 900–1000 m (INBIO); Río San Lorencito, 5 km N Colonia Palmareñ, 900–1000 m (INBIO); road to Arenal Lodge, 2 September 1998 (BYUC); Upala, Sector San Ramón de Dos Ríos, 1.5 km NW Hacienda Nueva Zelandia, 600–700 m (INBIO). Cartago- Quebrada Segunda, P.N. Tapantí, 1250 m, April 1992, May 1992 (INBIO); Turrialba, 4–13 August 1970 (USNM); ITCA at Turrialba, 13 March 1965 (BYUC). Guanacaste- La Pacifica, 4 km NW Cañas, 7–10 July 1973 (EMEC); Río San Lorenzo, 1050 m, Tierras Morenas, Z. P. Tenorio, November 1991, January 1992, August 1992 (INBIO); Turrialba, Tayutic, Grano de Oro, Chirripo, 1100–1200 m (INBIO). Guanacaste- Estación Pitilla, 9 km S. Santa Cecilia, 600–700 m (INBIO). Heredia- La Selva Biol. Sta., 3 km S Pto. Viejo, 16 July 1991 (USNM); La Selva, 80 m, 18 May 1993, 19 May 1993 (SEMC), 31 March 1990 (MUCR). Limón- Sector Cerro Cocorí, Fca. de E. Rojas, 150 m, November 1991 (INBIO); 7 mi N Guacimo, 22 February- 3 March 1988 (BYUC); Est Hitoy Cerere, 100, R. Cerere, Res. Biol. Hitoy Cerere, May 1991 (INBIO); Las Lomas, 3 January 1965 (MUCR); ca. 2 km W Pto Viejo, 20 m, 16 May 1993 (SEMC); Pococí, P.N. Colorado, Estación Cuarto Esquinas (INBIO); A.C. Llanuras del Tortuguero, Río Sardinas, 0–100 m (INBIO); Talamanca, Amubri, 0–100 m (INBIO); R.V.S. Gandoca Manzanillo, 0–100 m (INBIO). Puntarenas- Estación Boscosa, Peninsula de Osa, 15 September 1991 (INBIO); Las Alturas, 1400 m, 22 May 1992 (CDFA); Est Biol Las Alturas, 1500 m, Coto Brus., June 1991, December 1991, January 1992 (INBIO); Las Cruces Botanical Garden nr. San Vito, 3500 ft., 27–28 February 1985 (AMNH); Rancho Quemado, 200 m, Peninsula de Osa, 21 March- 7 April 1992, April 1992, September 1992 (INBIO); Est Queb. Bonita, 50 m, Res Biol Carara, 17 March- 30 April (INBIO); Rincón de Osa, 7 March 1969 (CASC); 3.5 mi. S. Rincón, Peninsula de Osa, 28 February- 12 March 1969 (CASC); 5 km. S. Rincón, 20 March 1973 (USNM); Est. Sirena, 0–100 m, P.N. Corcovado, October 1989, November 1989, February 1990, 21 March- 21 April 1992 (INBIO); Aguirre, Quepos, P.N. Manuel Antonio, 0–100 (INBIO); Estación Altamira, 1 km S Cerro Biolley, 1400–1500 m (INBIO); Estación Pittier, Sendero Río Canasta, 1700–1800 m (INBIO); Sendero Cerro Pittier, 1900–2000 m (INBIO); Coto Brus, Sabalito, Finca Cafrosa, 2 km NW Mellizas, 1200–1300 m (INBIO); Z.P. Las Tablas, Quebrada, Pizote, 1300–1400 m (INBIO); Z.P. Las Tablas, Coton, 1500–1600 m (INBIO); Estación Esquinas, Peninsula de Osa 0–100 m (INBIO); Alrededor del Río Corcovado, 0–100 m (INBIO); A.C.O. Golfito, Reserva Ftal Golfo Dulce, Estación Agujas, 200–300 m (INBIO); Sendero La Tarde, Cerro de Oroo, 5.6 km NW Cerro Rincón, 200–300 m (INBIO); Cerro Anguciana, Llano Bonito, Piedras Blancas, 800–900 m (INBIO); Estación Boscosa, 0–100 m (INBIO); Estero de Guerra, 0–100 m (INBIO); Bosque Eterno de los Niños, Sector Monteverde, 1500–1600 m (INBIO). **GUATEMALA:** 18 April 1932 (USNM). Alta Verapaz- Cacao Trece Aguas, 5.4 (USNM); Cacoj (USNM); Izabel, San Gil, 8 km N Las Escabuo, 800 m, 13 June 1983 (CMNC). Vera Paz- Chacoj (AMNH). **MEXICO:** Chiapas- Palenque, 9 August 1969 (CNC). Tabasco- Cardenas, 15 October 2013 (USNM). **PANAMA:** no further data (USNM). Chiriquí- Reserva Fortuna, Continental Divide Trail, 25 May 1993, 26 May 1993 (CDFA, EGRC); Santa Clara, 23–25 May 1980 (EGRC). Colón- Gamboa, 22 July 1975 (EGRC); 5 mi NW Gamboa, 27 April 1974 (EGRC); Pipeline rd., nr. Gamboa, 1 July 1976 (USNM); Pipeline Road, 8–9 July 1997 (USNM, EMEC); Skunk Hollow nr. Ft. Sherman, 28 May 1980 (EGRC); Achiote Rd., 10 km SW Gatun, 12 June 1976 (USNM, EGRC); Santa Rita Ridge, 13 June 1976 (EGRC). Panamá- Ancón, 19–21 August 1970 (USNM); Barro Colo Isld., 24 December 1928, 1 February 1929 (AMNH); Cerro Campana, 11–15 May 1980 (EGRC, USNM); near Chepo, 3 April 1974 (EGRC); Road leading to Clayton Observatory, 8 May 1971 (EGRC); Summit Gardens, 26 June 1976 (EGRC); Madden Forest, 2 November 1973, 25 June 1976 (EGRC); Madden Forest Reserve, 25 June 1976 (EGRC); Nusagandi area, I. K. U. S. A. Igar, 20 May 1993 (EGRC); Par. Nac. Soberania, Pipeline road, 23 May 1993 (EGRC). Total: 241.

#### Cephaloleia sandersoni

Taxon classificationAnimaliaColeopteraChrysomelidae

Staines, 1996

http://species-id.net/wiki/Cephaloleia_sandersoni

Fig. 230

Cephaloleia coeruleata Sanderson 1967: 137. (homoym of *Cephaloleia coeruleata*Weise 1911a (misspelling of *Cephaloleia caeruleata* Baly).
Cephaloleia sandersoni Staines 1996: 57 (replacement name for *Cephaloleia coeruleata* Sanderson). Staines 2008: 1 (key), 2009a: 21 (noted).

##### Description.

Oval; convex; metallic blue, venter and legs dark brown with bluish sheen. Head: vertex finely punctate, medial sulcus absent; frons projecting; slightly depressed between eyes. Antenna: reaches to humerus; slender; antennomere 1 clavate, subequal in length to 3; 2 transverse, ½ length of 1; 3 elongate; 4–10 elongate, subequal in length, each shorter than 3; 11 2× length of 10, subequal in length to 1 and 3, acutely pointed at apex; 1–2 punctate with scattered setae; 3–11 setose. Pronotum: transverse; lateral margin evenly arcuate from base to anterior angle, margined; anterior angle rounded, slightly produced; posterior angle acute; anterior margin emarginate behind head; disc subconvex; surface uneven, with two vague, rounded depressions between middle and lateral margin; surface sparsely, irregularly punctate, alutaceous; basal impression absent; pronotal length 1.1 mm; pronotal width 1.9 mm. Scutellum: broadly triangular; impunctate. Elytron: lateral margin slightly expanded apically, smooth, margined; apex rounded; sutural angle without tooth; humerus rounded, not produced; slightly constricted behind humerus; moderately punctate-striate, rows converge and unite apically; elytral length 3.4 mm; elytral width 2.4 mm. Venter: prosternum impunctate medially, rugose laterally; meso- and metasterna impunctate medially, punctate laterally; abdominal sterna punctate; suture between sterna 1 and 2 complete; last sternite with apical margin broadly sinuate in male, less so in female; pygidium setose. Leg: slender; punctate; tibia with fringe of golden setae at apex. Total length: 4.7–5.4 mm.

##### Diagnosis.

This species is similar to *Cephaloleia barroi*. It can be distinguished by the sparsely punctate vertex of the head, by the straight lateral margins of the pronotum, and by antennomeres 1 and 2 being transverse and subequal in length.

##### Host plant.

Adults have been collected in *Thrinax* sp. inflorescences (Arecaceae) (Sanderson 1967).

##### Distribution.

Jamaica.

##### Type material examined.

Holotype: Jamaica, Grier Mount on Mount Diablo (St. Catherine Parish), 3000 ft., February 30, 1953, R. A. Howard and G. R. Procter, ex. inflorescence sheaths of *Thrinax* (INHS).

##### Specimens examined.

**JAMAICA:** Trelawny Parish- Windsor Estate, 22 August 1955 (INHS). Total: 9.

#### Cephaloleia saundersii

Taxon classificationAnimaliaColeopteraChrysomelidae

Baly, 1858

http://species-id.net/wiki/Cephaloleia_saundersii

Fig. 231

Cephalolia saundersii Baly 1858: 57. Gemminger and Harold 1876: 3602 (catalog); Donckier 1899: 551 (catalog); Weise 1910: 90 (noted), 1911a: 9 (catalog), 1911b: 12 (catalog).
Cephaloleia saundersii Baly. Uhmann 1957b: 25 (catalog); Staines and Staines 1999: 524 (Baly species list).
Cephaloleia pulchella Baly. Waterhouse 1881: 261 (misidentification); Weise 1910: 90 (correction).

##### Description.

Elongate; subparallel; subdepressed; shining; black; basal ⅓ of pronotum and longitudinal vitta on elytra yellowish; scutellum, venter, and legs (except tarsi and tibio-femoral joint darker) yellowish. Head: vertex finely punctate, medial carina present; frons not projecting; depressed between eyes. Antenna: reaches to humerus; slender; antennomere 1 incrassate, elongate, 2× length of 2; 2 elongate, cylindrical; 3 cylindrical, subequal in length to 1; 4–5 elongate, cylindrical, decreasing in length; 6–7 transverse, decreasing in length; 8–10 transverse, subequal in length, each shorter than 7; 11 2× length of 10, rounded at apex; 1–2 punctate with scattered setae, 3–11 setose. Pronotum: subquadrate, slightly narrowed basally; lateral margin straight then rounding to anterior angle, canaliculate; anterior angle rounded, slightly produced; posterior angle acute; anterior margin emarginate behind head; disc subconvex; surface coarsely punctate; transverse basal impression present medially; pronotal length 1.5–1.7 mm; pronotal width 1.9–2.1 mm. Scutellum: pentagonal; impunctate. Elytron: lateral margin straight, smooth, narrowly margined; apex rounded, emarginate at sutural angle; sutural angle without tooth; humerus rounded, not produced; slightly constricted behind humerus; declivity beginning just behind humerus at puncture row 7 not edged with faint carina; flattened along suture; moderately punctate-striate, rows converge and unite apically; elytral length 5.1–5.4 mm; elytral width 2.1–2.3 mm. Venter: pro-, meso-, and metasterna impunctate; abdominal sterna impunctate with row of setae apically; suture between sterna 1 and 2 entirely obsolete; last sternite with apical margin broadly emarginate medially in male, bisinuate in female. Leg: slender; punctate; tibia with fringe of setae on inner margin of apex. Total length: 6.5–7.0 mm.

##### Diagnosis.

This species is similar to *Cephaloleia eximia* and *Cephaloleia pulchella*. It can be distinguished by the suture between abdominal sterna 1 and 2 being obsolete and by the pro-, meso-, and metasterna being impunctate.

##### Distribution.

Brazil, Ecuador, Peru.

##### Type material.

Holotype: northern Brazil, Saunders coll. (depository unknown, not examined).

##### Specimens examined.

**Ecuador:** Napo- Limonocha, 7 June 1977 (USNM), 300 m, 31 March 1974 (EGRC); Puyo, 960 m, 1–8 October 1970 (USNM); Sacha Lodge, 270 m, 3–13 April 1994, 23 April- 4 May 1994, 13–25 July 1994 (USNM), 2–12 February 1994, 22 February- 4 March 1994, 13–23 April 1994, 23 April- 4 May 1994, 14–24 May 1994, 3–13 July 1994, 13–25 July 1994, 16–29 August 1994, 27 August-10 September 1994, 10–20 September 1994, 20–30 September 1994 (SEMC); 9 km SE Lumbaqui, 650 m, 7–8 August 1998 (AJGC); Río Napo, Sacha Lodge, 4–14 May 1994, 3–13 June 1994 (BYUC). **Peru:** Madre de Dios- CICRA Field Station, 272 m, 12 June 2011 (SEMC); Pontiacolla Lodge, 8 km NW El Mirador Trail, Alto Madre de Dios River, 800 m, 23–26 October 2000 (SEMC); Rio Tambopata Res., 30 km SW Puerto Maldonado, 290 m, 11 November 1982, 22 October 1983 (USNM); Zona Reserva Manu Pakitza, 400 m, 13–18 February 1992 (USNM). Total: 31.

#### Cephaloleia schmidti

Taxon classificationAnimaliaColeopteraChrysomelidae

Uhmann, 1933

http://species-id.net/wiki/Cephaloleia_schmidti

Fig. 232

Cephalolia schmidti Uhmann 1933: 168.
Cephaloleia schmidti Uhmann. Blackwelder 1946: 720 (catalog); Papp 1953: 22 (catalog); Uhmann 1957a: 25 (catalog); Descarpentries and Villiers 1959a: 139 (types); Gaedike and Döbler 1971: 359 (types); Wilcox 1983: 137 (catalog); Staines 1996: 58 (Central America species), 1997: 414 (Uhmann species list); Staines and Staines 1997: 20 (types); McKenna and Farrell 2005: 121 (phylogeny), 2006: 10949 (phylogeny).

##### Description.

Narrowly elongate; subparallel; subconvex; head, antennae, scutellum, venter, and legs black, pronotum reddish, elytra with basal ½ reddish-brown with a black sutural vitta, apical ½ black. Head: vertex densely punctate, medial sulcus absent; frons not projecting; keel present between antennal bases; depressed between eyes. Antenna: reaches to humerus; slender; antennomere 1 transverse, laterally compressed; 2 transverse, shortest; 3–4 elongate, cylindrical; 3 elongate, as long as 1 and 2 combined; 4 ¾ length of 3; 5–10 transverse, subequal in length; 11 2× length of 10, pointed at apex; 1–2 punctate with scattered setae; 3–11 setose. Pronotum: transverse; lateral margin sinuate then rounding to anterior angle, canaliculate; anterior angle acute, produced; posterior angle acute; anterior margin emarginate behind head; disc subconvex; surface deeply, sparsely, irregularly punctate; basal impression absent; pronotal length 0.9–1.0 mm; pronotal width 1.1–1.4 mm. Scutellum: pentagonal; slightly punctate. Elytron: lateral margin straight, smooth, margined; apex rounded, sparsely serrate; sutural angle without tooth; humerus rounded, not produced; slightly constricted behind humerus; moderately punctate-striate, rows converge and unite apically; puncture row 10 removed from lateral margin; elytral length 3.8–4.0 mm; elytral width 1.8–2.0 mm. Venter: pro-, meso-, and metasterna impunctate medially, punctate laterally; abdominal sterna punctate, each puncture with pale seta; suture between sterna 1 and 2 complete. Leg: slender; femur punctate at apex; tibia with fringe of setae on inner margin of apex. Total length: 5.0–5.3 mm.

##### Diagnosis.

This species is similar to *Cephaloleia atriceps* and *Cephaloleia ruficollis*. It can be distinguished by the elytra being reddish-brown at the base with a black sutural vitta, by the vertex of the head being depressed between the eyes, and by the suture between abdominal sterna 1 and 2 being complete.

##### Distribution.

Costa Rica, Panama.

##### Type material examined.

Paratype: Costa Rica, F. Nevermann [green label]/ La Caja, B. San Jose, Schmidt leg. [reversed green label]/ Paratyp [red label]/ Cephalolia schmidti Uh., E. Uhmann det. 33/ Paratype No. 54638 USNM [orange label] (USNM).

##### Specimens examined.

**COSTA RICA:** Alajuela- 20 km S Upala, 22–31 May 1991 (BYUC). Cartago- Limón border, 500 m, 40 km NE Turrialba, 18 May 1979 (CMNC). Guanacaste- 3 km SE R. Naranjo, 1–5 June 199- (BYUC); Volcán Miravelles Geothermal Area, 3 July 1991 (EMEC). Puntarenas- Monteverde, 1400 m, 23 May 1979 (CMNC); 4–6 km S Sta Elena, June 4–7 1980 (EGRC); Garabito, Reserva Biol Carara, Estación Quebrada Bonita, 0–100 m (INBIO). San José- La Caja, S. San José (USNM). **PANAMA:** Panamá- Cerro Campana, 11–15 May 1980 (EGRC). Total: 12.

#### Cephaloleia scitulus

Taxon classificationAnimaliaColeopteraChrysomelidae

Staines, 1996

http://species-id.net/wiki/Cephaloleia_scitulus

Fig. 233

Cephaloleia scitulus Staines 1996: 58.

##### Description.

Elongate; subparallel; subdepressed; head, pronotum (except pale anterior and lateral margins), scutellum, and antennae black; elytra yellow with black sutural vitta from base to apex to row 2, at base includes all of row 2, narrows abruptly to interspace 1 at apex; and lateral vitta from behind humerus to apex from rows 6–9, lateral vittae unite at apex; legs yellow with femur with apex darker, tibia dark at base and apex, tarsi dark. Head: vertex punctate, medial sulcus absent; frons not projecting; slightly depressed between eyes. Antenna: nearly ½ body length; slender; antennomere 1 elongate; 2 transverse; 3 elongate, subequal in length to 1; 4–5 elongate, each shorter than 1 or 3; 6–10 transverse, subequal in length, each shorter than 5; 11 subequal in length to 1 or 3, pointed at apex; 1–3 punctate with scattered setae; 4–11 setose. Pronotum: transverse, widest at base; lateral margin sinuate then rounding to anterior angle, canaliculate; anterior angle rounded, slightly produced; posterior angle acute; anterior margin emarginate behind head; disc subconvex; surface sparsely, deeply punctate, more deeply laterally, area just behind head impunctate; transverse basal impression present medially; pronotal length 1.1–1.4 mm; pronotal width 1.4–1.7 mm. Scutellum: pentagonal; alutaceous. Elytron: lateral margin straight, smooth, margined; apex rounded; sutural angle with small tooth; humerus rounded, slightly produced; slightly constricted behind humerus; moderately punctate-striate, humerus virtually impunctate, rows converge and unite apically; elytral length 4.3–5.3 mm; elytral width 1.9–2.6 mm. Venter: pro-, meso-, and metasterna impunctate medially, punctate laterally; abdominal sternite 1–2 impunctate medially, punctate laterally, each puncture with pale seta; sterna 3–5 punctate, each puncture with pale seta; suture between sterna 1 and 2 obsolete medially; last sternite with apical margin emarginate medially in male, rounded in female. Leg: slender; femur punctate at apex; tibia with row of setae on inner margin and fringe of setae at apex. Total length: 5.4–6.9 mm.

**Figures 233–241.**
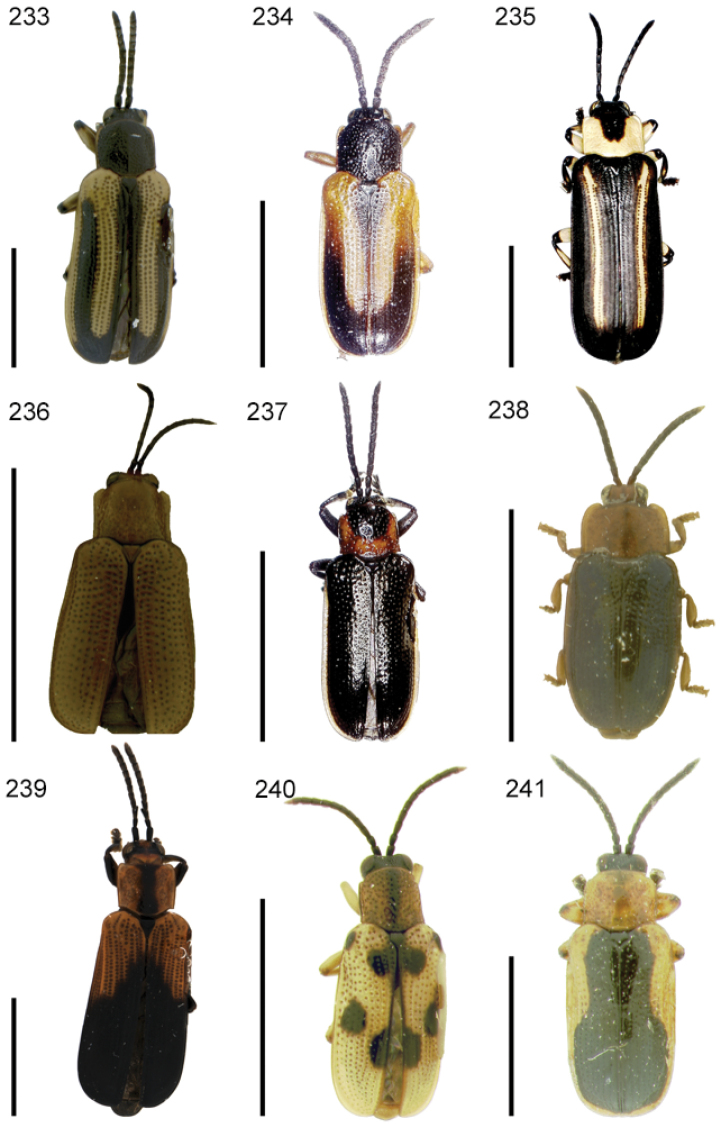
Habitus. **233** *Cephaloleia scitulus* **234** *Cephaloleia semivittata* **235** *Cephaloleia separata* **236** *Cephaloleia simplex* **237** *Cephaloleia splendida* **238** *Cephaloleia steinhauseni* **239** *Cephaloleia stenosoma* **240** *Cephaloleia stevensi* **241** *Cephaloleia strandi*. Scale bars equal 3 mm.

##### Diagnosis.

This species is similar to *Cephaloleia parenthesis*. It can be distinguished by the suture between abdominal sterna 1 and 2 being obsolete medially and by the sutural angle of the elytra with a small tooth.

##### Distribution.

Panama.

##### Type material examined.

Holotype: Panama at San Pedro, III-7–68/ Lindsay and Gillogly 68–4934/ Cephaloleia or nr d R. White/ Holotype Cephaloleia scitulus Staines, Des. C. L. Staines 1994 [red label] (USNM).

##### Specimens examined.

**PANAMA:** Chiriquí- Reserva Fortuna, Continental Divide Trail, 29 May 1993 (CDFA), 30 May 1994 (CDFA, USNM), 23 May- 9 June 1996 (USNM); Reserva La Fortuna, Hydrographic sta. trail, 28 May 1993 (EGRC). Coclé- El Valle, 20 February 1959 (FMNH); El Valle, El. 700 m, 28 July 1974 (EGRC). Panamá- Cerro Azul, Los Alto, 24 May 1994 (USNM); Cerro Jefe, 700 m, 19 June 1976 (EGRC). Total: 21.

#### Cephaloleia semivittata

Taxon classificationAnimaliaColeopteraChrysomelidae

Baly, 1885

http://species-id.net/wiki/Cephaloleia_semivittata

Fig. 234

Cephaloleia semivittata Baly 1885: 16. Blackwelder 1946: 720 (catalog); Papp 1953: 22 (catalog); Uhmann 1957a: 25 (catalog); Wilcox 1983: 137 (catalog); Staines 1996: 59 (Central America species), 2004: 312 (host plants), 2011: 50 (faunal list); Staines and Staines 1999: 524 (Baly species list); McKenna and Farrell 2005: 120 (phylogeny); García–Robledo et al. 2013a: 3 (biology).
Cephalolia semivittata Baly. Donckier 1899: 551 (catalog); Weise 1911a: 9 (catalog), 1911b: 12 (catalog).

##### Description.

Elongate; subparallel; subdepressed; head, antennae, scutellum, pronotum (except anterior margin and anterior angle margined in black), and venter (except pro-, meso-, and metasterna reddish-brown medially) black; elytra reddish-yellow on basal ½, apical ½ with black vitta from lateral margin to suture, extends between puncture rows 5–10, lateral margin reddish-yellow. Head: vertex densely punctate, medial carina present; frons finely, sparsely punctate, not projecting; not depressed between eyes. Antenna: reaches to humerus; slender; antennomeres 1 and 3 elongate, subequal in length; 2 transverse; 4–10 transverse, subequal in length, each longer than 2; 11 2× length of 10, rounded at apex; 1–3 punctate with scattered setae; 4–11 setose. Pronotum: quadrate; lateral margin straight then rounding to anterior angle, slightly canaliculate; anterior angle obtuse, produced; posterior angle angulate; anterior margin emarginate behind head; disc subconvex; surface strongly punctate, longitudinal section of disc impunctate; basal impression absent; pronotal length 0.9–1.0 mm; pronotal width 1.3 mm. Scutellum: pentagonal, impunctate. Elytron: lateral margin straight, smooth, margined; apex rounded; sutural angle without tooth; humerus rounded, slightly produced; slightly constricted behind humerus; flattened along suture; strongly punctate-striate, rows converge and unite apically; elytral length 3.4–3.6 mm; elytral width 1.7–1.9 mm. Venter: pro-, meso-, and metasterna impunctate medially, punctate laterally; abdominal sterna punctate, each puncture with pale seta; suture between sterna 1 and 2 obsolete medially; last sternite with apical margin emarginate medially in male, truncate in female. Leg: slender; punctate, each puncture with pale seta; tibia with fringe of setae on inner margin of apex. Total length: 4.6–4.8 mm.

##### Diagnosis.

This species is similar to *Cephaloleia belti*, *Cephaloleia consanguinea*, *Cephaloleia erugatus*, *Cephaloleia triangularis*, *Cephaloleia trivittata*, *Cephaloleia variabilis*, and *Cephaloleia vicina*. It can be distinguished by the elytral puncture rows being distinct apically, by antennomere 1 being subequal in length to 2 and 3, by antennomere 2 being transverse, and by the uniform pronotal punctures.

##### Host plant.

Adults have been collected on *Calathea marantifolia* Standley (Staines 1996); *Cephaloleia cleistantha* Standl., *Cephaloleia crotalifera* S. Watson, *Pleiostachya pruinosa* (W. Bull. ex. Regel) K. Schum. (Marantaceae).

##### Distribution.

Costa Rica, Panama.

##### Type material examined.

Syntype: Type H. T. [white disk with red border]/ Panama, Bugaba, Champion/ B. C. A., Col. VI, 2. Cephaloleia semivittata, Baly/ Cephaloleia/ Cephalolia semivittata Baly, Panama [blue handwritten label] (BMNH, 1).

##### Specimens examined.

**COSTA RICA:** Cartago- Turrilba, Santa Teresita, Monumento Nacional Guayabo, 1100–1200 m (INBIO). Guanacaste- Estac. Cacao, 1000–1400 m, SE side Volcán Cacao, 1988–1989, June 1990 (INBIO); Est. Pitilla, 700 m, 9 km S Sta. Cecilia, P.N. Guanacaste, December 1989, July 1991 (INBIO); Río San Lorenzo, 1050 m, Tierras Morenas, Z. P. Tenorio, 28 March- 21 April 1992, July 1992 (INBIO). Heredia- La Selva Biol. Sta., 3 km S Pto. Viejo, 23 July 1992 (USNM); Est. Biol. La Selva, 07 July 2001 (USNM); Rara Avis Biological Station, 15 November 2011 (USNM). Limón- Sector Cerro Cocorí, Fca. de E. Rojas, 150 m, November 1991, March 1992 (INBIO); Cerro Tortuguero, P.N. Tortuguero, December 1989 (INBIO); Est. Cuatro Esquinas, 0 m, P.N. Tortuguero, August 1991 (INBIO). Puntarenas- Est. Biol. Las Alturas, 1500 m, Coto Brus., November 1991 (INBIO); Coto Brus, Las Cruces Biological Station, 10 March 2012 (USNM); Rancho Quemado, Pen. Osa, February 1991, April 1991, May 1991, November 1991, 21 March- 7 April 1992, May 1992 (INBIO); Est. Sirena, 0–100 m, P.N. Corcovado, January 1990, September 1990, April 1991, October 1991, July 1991, September 1991, November 1991, December 1991 (INBIO); Aguirre, Quepos, P.N. Manuel Antonio, 0–100 m (INBIO). **PANAMA:** Colón- Porto Bello, 17 February 1911, 18 February 1911, 27 February 1911, 6 March 1911 (USNM). Total: 73.

#### Cephaloleia separata

Taxon classificationAnimaliaColeopteraChrysomelidae

Baly, 1885

http://species-id.net/wiki/Cephaloleia_separata

Fig. 235

Cephaloleia separata Baly 1885: 22. Blackwelder 1946: 720 (catalog); Papp 1953: 22 (catalog); Uhmann 1957a: 25 (catalog); Wilcox 1983: 137 (catalog); Staines 1996: 59 (Central America species); Staines and Staines 1999: 524 (Baly species list).
Cephalolia separata Baly. Donckier 1899: 551 (catalog); Weise 1905a: 131 (noted), 1911a: 9 (catalog), 1911b: 11 (catalog); Uhmann 1932b: 261 (noted).

##### Description.

Elongate; subparallel; subdepressed; head (except yellowish frons) and scutellum brownish; antennae black; pronotum yellowish with anterior margin dark behind head; elytra brownish-black with yellow vitta beginning in interspace 3 going to interspace 6; venter with prosternum yellow; mesosternum yellow, medially darker laterally, metasternum and abdominal sterna yellow or with black macula on apical margin of each sternite medially; leg with femur and tibia yellow, tarsi darker. Head: vertex punctate, medial sulcus present; frons finely punctate, not projecting; eyes long, convex, finely faceted; slightly depressed between eyes. Antenna: reaches to humerus; slender; antennomere 1 elongate, robust, as long as 2–4 combined; 2–3 triangular, 3 longer than 2; 2 shortest; 3 1½ length of 2; 4–6 transverse, subequal in length, each ¾ length of 3; 7–10 elongate, subequal in length, each slightly longer than 6; 11 slightly longer than 10, pointed at apex; 1–5 punctate with scattered setae; 6–11 setose. Pronotum: subquadrate; lateral margin straight then rounding to anterior angle, margined; anterior angle rounded, slightly produced; posterior angle angulate; anterior margin weakly emarginate behind head; disc flattened; surface sparsely punctate laterally, disc impunctate; basal impression absent; pronotal length 1.3–1.4 mm; pronotal width 1.6–1.7 mm. Scutellum: acutely triangular; micropunctate. Elytron: lateral margin straight, smooth, margined; apex rounded; sutural angle without tooth; humerus rounded, not produced; slightly constricted behind humerus; shallowly punctate-striate, punctures in vitta larger, rows converge and unite apically; elytral length 4.8 mm; elytral width 2.2–2.3 mm. Venter: pro-, meso-, and metasterna impunctate medially, punctate laterally; abdominal sterna punctate, each puncture with pale seta; suture between sterna 1 and 2 complete; last sternite with apical margin shallowly emarginate in male, truncate in female. Leg: slender; tibia sparsely punctate, each puncture with pale seta, with fringe of setae on inner margin of apex. Total length: 6.2–6.7 mm.

##### Diagnosis.

This species is similar to *Cephaloleia ornatrix* and *Cephaloleia presignis*. It can be distinguished by the elytral punctures rows being distinct to the apex.

##### Distribution.

Costa Rica, Mexico.

##### Type material examined.

Lectotype male: Sontecomapam/ Type H. T. [white disk with red border]/ Mexico, Salle coll./ male/ B. C. A., Col. VI, 2. Cephaloleia separata, Baly/ Lectotype Cephaloleia separata Baly Des. C. L. Staines 1993 [red label] (BMNH).

##### Specimens examined.

**COSTA RICA:** Heredia- El Angel falls, Vara Blanca area, 21 June 1969 (USNM). Limón- Est. Hitoy Cerere, R. Cerere, Res. Biol. Hitoy Cerere, 4–20 December 1991 (INBIO). Puntarenas- Aguirre, Quepos, P.N. Manuel Antonio, 0–100 m (INBIO). Total: 5.

#### Cephaloleia simoni

Taxon classificationAnimaliaColeopteraChrysomelidae

Pic, 1934 Incertae Sedis

http://species-id.net/wiki/Cephaloleia_simoni

Image not available

Cephalolia simoni Pic 1934: 155.
Cephaloleia simoni Pic. Bryant 1942: 205 (faunal list); Uhmann 1957b: 25 (catalog).

##### Description.

From Pic 1934: Oblong-elongate; subdepressed; shining; testaceous, pronotum with disc brownish, head darker posteriorly, elytral disc greenish-brown; venter and legs testaceous. Head: small; sparsely punctate. Antenna: slender. Pronotum: subquadrate; lateral margin straight, rounded anteriorly, slightly canaliculate; anterior angle produced; disc nearly impunctate, irregularly punctate laterally. Scutellum: large. Elytron: lateral margin narrowly margined; apex rounded; punctate-striate; with short carina behind humerus. Total length: 5.0 mm.

##### Distribution.

Venezuela.

##### Type material.

Type: Colonie Tovar (depository unknown, not examined).

##### Specimens examined.

None.

##### Remarks.

The placement of this species is uncertain since many of the species Pic described in *Cephaloleia* belong to other genera. We have not seen any specimen which can be assigned to this species based on the short description nor have I found any material identified to this species. The type is not present in the Museum National d’Historie Naturelle (Paris) (MNHN).

#### Cephaloleia simplex

Taxon classificationAnimaliaColeopteraChrysomelidae

Staines, 2008

http://species-id.net/wiki/Cephaloleia_simplex

Fig. 236

Cephaloleia simplex Staines 2008: 2. Staines 2009a: 21 (noted).

##### Description.

Small; elongate; subparallel; subdepressed; yellowish-brown; eyes and antennae (except basal antennomere) nearly black, venter brownish except pro- and mesosterna blackish; base of pronotum much narrower than base of elytra. Head: vertex punctate, alutaceous between punctures, medial sulcus present; front nearly vertical; interantennal carina absent; clypeus small, punctate; maxillary and labial palps yellowish; frons not projecting; slightly depressed between eyes. Antenna: extends beyond humerus; slender; antennomeres 3–11 elongate; 1 short; 2 2× length of 1; 3 longer than 1 and 2 combined; 4–6 subequal in length, each longer than 2; 7–10 subequal in length, each shorter than 6; 11 2× length of 10, bluntly pointed at apex; 1–2 punctate with scattered setae; 3–11 setose. Pronotum: transverse; lateral margin straight and slightly divergent for basal ⅞ then rounding to anterior angle, margined; anterior angle rounded, slightly produced; posterior angle acute; anterior margin weakly emarginate behind head; disc subconvex; surface with disc sparsely punctate, moderately coarsely punctate laterally; basal impression absent; pronotal length 0.9 mm; pronotal width 1.1 mm. Scutellum: large; pentagonal; acutely pointed at apex; alutaceous. Elytron: lateral margin straight, smooth, margined; apex rounded, smooth; sutural angle without tooth; humerus rounded, impunctate; slightly constricted behind humerus; puncture rows with few punctures, rows converge and unite apically; scutellar row reaching basal ⅓; elytral length 2.9 mm; elytral width 1.6 mm. Venter: pro-, meso-, and metasterna impunctate medially, punctate laterally; abdominal sternite 1 punctate; sterna 2–5 punctate, each puncture with white setae; suture between sterna 1 and 2 complete; last sternite broadly emarginate in male, entire, rounded in female. Leg: short, robust; coxa and femur punctate; tibia with fringe of setae on inner margin of apex. Total length: 3.0 mm.

##### Diagnosis.

This species is similar to *Cephaloleia mauliki*, *Cephaloleia placida*, and *Cephaloleia sulciceps*. It can be distinguished by the suture between abdominal sterna 1 and 2 being complete and by the small size.

##### Distribution.

Dominica, Grenada.

##### Type material examined.

Holotype: Dominica, St. Paul Parish, Mornes Trois Piton Nat’l. Pk., trail to Middleham Falls, 15°21'06" N, 61°20'06" W, el. 2200 ft., V-20-VI-2–2000/ L. Benavides, E. Chavez, J. Dye and E. Kretsch, Malaise trap, 2000–10/ Holotype Cephaloleia simplex Staines 2007 [red label] (TAMU).

##### Specimens examined.

**GRENADA:** Grand Etan NP, Mt. Qua Qua Tr., 9 September 1991 (BYUC). Total: 6.

#### Cephaloleia splendida

Taxon classificationAnimaliaColeopteraChrysomelidae

Staines, 1996

http://species-id.net/wiki/Cephaloleia_splendida

Fig. 237

Cephaloleia splendida Staines 1996: 60. McKenna and Farrell 2005: 121 (phylogeny); Staines 2011: 50 (faunal list).

##### Description.

Small; elongate; subparallel; subconvex; shining black (except elytral lateral margins yellow); pronotum reddish-yellow except black rectangular black macula from anterior margin to disc behind head; venter with prosternum black medially, reddish-yellow laterally. Head: vertex deeply, densely punctate, medial sulcus absent; frons not projecting; not depressed between eyes. Antenna: reaches beyond humerus; slender; antennomeres 1–3 elongate, 1 and 2 subequal in length, 3 longer; 4–10 transverse, subequal in length; 11 rounded at apex, subequal in length to 1 or 2; 1–2 punctate with scattered setae; 3–11 setose. Pronotum: transverse; lateral margin straight then rounding to anterior angle, canaliculate; anterior angle rounded, produced; posterior angle acute; anterior margin emarginate behind head; disc subconvex; surface sparsely, deeply punctate; transverse impression present just posterior to disc; pronotal length 0.7–0.9 mm; pronotal width 0.9–1.0 mm. Scutellum: pentagonal; alutaceous. Elytron: lateral margin straight, smooth, margined; apex rounded; sutural angle with minute tooth; humerus rounded, not produced; slightly constricted behind humerus; moderately punctate-striate, rows converge and unite apically; elytral length 2.3–3.2 mm; elytral width 1.1–1.5 mm. Venter: pro-, meso-, and metasterna impunctate medially, punctate laterally; abdominal sterna 1–2 sparsely punctate, 3–5 densely punctate, each puncture with pale seta; suture between sterna 1 and 2 obsolete medially; last sternite with apical margin emarginate medially in male, entire in female. Leg: slender; femur and tibia punctate, each punctate with pale seta; tibia with fringe of setae on inner margin of apex. Total length: 3.9–4.3 mm.

##### Diagnosis.

This species is similar to *Cephaloleia turrialbana*. It can be distinguished by antennomere 1 being subequal in length to 2 and by the basal impression on the pronotum.

##### Distribution.

Costa Rica, Nicaragua, Panama.

##### Type material examined.

Holotype: Costa Rica, Penas Blancas, 7.VII.1987, E. Cruz, FIT/ Holotype Cephaloleia splendida Staines, Des. C. L.Staines 1994 [red label] (USNM).

##### Specimens examined.

**COSTA RICA:** Alajuela- Peñas Blancas, 7 July 1987 (USNM); Upala, Sector San Ramón de Dos Ríos, 1.5 km NW Hacienda Nueva Zelandia, 600–700 m (INBIO). Cartago- no further data (ZMHB); Quebrada Segunda, Ref. Nac. Fauna Silv. Tapantí, 1250 m, April 1992 (INBIO); Quebrada Segunda, 1200–1300 m (INBIO); Turrialba, Tayutic, Grano deo Oro, Chirripo, 1100–1200 m (INBIO). Guanacaste- Estación Pitilla, 9 km S. Santa Cecilia, 600–700 m (INBIO); Liberia, Mayorga, Estación Cacao, 2 km SW Cerro Cacao, 900–1000 m (INBIO); Río San Lorenzo, Tierras Morenas, 900–1000 m (INBIO). Heredia- La Selva Bio. Station, January 1993 (BYUC). Limón- 16 km W Guápiles, 400 m, March 1989 (USNM); Est. Jalova, 0 m, P.N. Tortuguero, July 1990 (INBIO); Valle La Estrella, 0–100 m (INBIO); Sardinas, Barra del Colorado, 4 km NW Cerro Cocorí 0–100 m (INBIO). Puntarenas- R. F. Gulfo Dulce, 3 km SW Rincón, 10 m, October-December 1990 (USNM); Monte Verde Biol. Res, Camino Penas Blancas, 1400 m, 10 July 1989 (USNM); Osa, Sierpe, Rancho Quemado, 200–300 m (INBIO); Perez Zeledón Santa Elena, Las Nubes, 1200–1300 m (INBIO); 3 km SW Rincón, 10 m, March-May 1991 (USNM); 6 km S San Vito Las Cruces,1200 m, March 1988 (USNM). San José- 1 km NE Estación Santa Elena, 1300–1400 m (INBIO); Estación Bijagual, 1.5 km N Bijagual, 400–500 m (INBIO). **Nicaragua:** Matagalpa- 6 km N Matagalpa, 1350 m, 19 May 2002 (SEMC). **PANAMA:** Chiriquí- El Valle del Nubes, 12 mi NW Rovira, 4000 feet, 10 March 1960 (CDFA); Reserva La Fortuna, Continental Divide Tr., 28 May 1992 (EGRC), 25 May 1993 (CDFA), 10 May 1994 (CDFA, USNM) Panamá- Cerro Campana, 27 August 1972 (EGRC), May 11–15, 1980 (EGRC), 17 May 1993 (EGRC). Total: 98.

#### Cephaloleia stainesi

Taxon classificationAnimaliaColeopteraChrysomelidae

García-Robledo sp. n.

http://zoobank.org/9BC91A48-6DCA-4420-A61D-25C64B9AE13A

http://species-id.net/wiki/Cephaloleia_stainesis

Fig. 273

##### Description.

Elongate; parallel-sided; subdepressed; head, pronotum, scutellum, venter, and legs yellowish; antennomeres 1, 2, and apical ½ of 11 yellow, 3 to 10 and basal ½ of 11 black; elytra black with six large, oval, pale yellowish-white maculae. Head: vertex finely punctate, not depressed between eyes, medial sulcus present; interantennal keel absent; clypeus punctate, each puncture with pale seta. Antenna: reaches to humerus; antennomeres 1 to 4 compressed laterally; 5 to 11 filiform; incrassate, 2½x length of 2; 2 to 4 transverse, with projection apically; 2 ¾ length of 3; 3 and 4 subequal in length; 5 to 10 subequal in length; slightly longer than 10, pointed at apex; 1 and 2 punctate, each puncture with pale seta; 3 to 11 setose. Pronotum: transverse; lateral margin straight for basal ¾, then rounding to anterior angle, canaliculate; anterior angle obtuse, produced; anterior margin emarginate; posterior angle acute; posterior margin strongly bisinuate; surface irregularly punctate, disc nearly impunctate; pronotal length 1.6 mm; pronotal width 1.9 mm. Scutellum: pentagonal; alutaceous. Elytron: lateral margin straight, smooth; exterior apical angle and apical margin rounded, smooth; sutural angle rounded; humerus rounded, not produced; surface finely punctate-striate, puncture rows converge and unite apically; elytral length 4.6 mm; elytral width 2.4 mm. Venter: pro-, meso-, and metasterna impunctate; abdominal sterna finely punctate, each puncture with pale seta; suture between abdominal sterna 1 and 2 complete; apical margin of last sternite truncate in male. Leg: long; robust; punctate, each puncture with pale seta; tibia with fringe of setae on inner apical margin. Total length: 6.7 mm.

##### Etymology.

Named for Charles L. Staines in recognition of his many contributions to the understanding of the biology and taxonomy of Chrysomelidae in general and of the genus *Cephaloleia* in particular. Also in recognition of his mentorship to new generations of researchers. The name is a noun in apposition.

##### Diagnosis.

*Cephaloleia stainesi* sp. n. is most similar to *Cephaloleia fenestrata* Weise. It can be distinguished by the lateral margin of the pronotum being canaliculate, by the lack of a deep sulcus along the lateral margin of the pronotum, by the suture between abdominal sterna 1 and 2 being complete, and by the legs being punctate.

##### Host plant.

*Heliconia latispatha* Benth. (Heliconiaceae).

##### Distribution.

Costa Rica.

##### Type material.

Holotype male: Costa Rica: Puntarenas, Coto Brus, Carretera Costanera, 70 m near KM 34, 9°3'15.5"N, 83°38'7.04"W, 31 July 2013, Carlos García-Robledo/ 1208_CG_31_Jul_2013_1815, *Heliconia latispatha* Benth. (Heliconiaceae)/ Holotype Cephaloleia stainesi García-Robledo, des. C. García-Robledo 2014 (red label), USNM.

#### Cephaloleia steinhauseni

Taxon classificationAnimaliaColeopteraChrysomelidae

Uhmann, 1961b

http://species-id.net/wiki/Cephaloleia_steinhauseni

Fig. 238

Cephaloleia steinhauseni Uhmann 1961b: 15. Uhmann 1964a: 404 (catalog); Gaedike and Döbler 1971: 360 (types); Staines 1997b: 414 (Uhmann species list).
Cephaloleia steinhauseni musae Uhmann 1961b: 15. Uhmann 1964a: 404 (catalog); Staines 1997b: 414 (Uhmann species list).

##### Description.

Small; elongate; ovate; subdepressed; shining; reddish-yellow; antennae, except basal antennomere, black; elytra bluish except lateral margin or totally reddish-yellow. Head: vertex impunctate, medial carina absent, eyes not strongly convex; frons small, not projecting; slightly depressed between eyes. Antenna: reaches to humerus; slender; antennomeres 1–5 elongate, cylindrical; 1 2× length of 2; 3–5 subequal in length, each longer than 2; 6–10 transverse, subequal in length, each shorter than 5; 11 longest, pointed at apex; 1–2 impunctate; 3–11 setose. Pronotum: transverse; lateral margin straight then rounding to anterior angle, moderately margined; anterior angle rounded, slightly produced; posterior angle acute; anterior margin emarginate behind head; disc subconvex; surface finely, sparsely punctate; basal impression absent; pronotal length 0.6–0.8 mm; pronotal length 1.0–1.2 mm. Scutellum: pentagonal; impunctate. Elytron: lateral margin straight, smooth, margined; apex rounded; sutural angle without tooth; humerus rounded, not produced; slightly constricted behind humerus; flattened along suture; shallowly punctate-striate, punctures confused near apex; elytral length 2.3–2.6 mm; elytral width 1.2–1.4 mm. Venter: pro-, meso-, and metasterna impunctate; abdominal sterna sparsely punctate, each puncture with white seta; suture between sterna 1 and 2 complete; last sternite with apical margin strongly emarginate in male, rounded in female. Leg: slender; impunctate; tibia with fringe of setae on inner margin of apex. Total length: 3.0–3.5 mm.

##### Diagnosis.

This species has two color forms. The bicolored form is similar to *Cephaloleia abdominalis*, *Cephaloleia amazona*, *Cephaloleia princeps*, *Cephaloleia susanae* sp. n., and *Cephaloleia teutonica*. It can be distinguished by the smaller size, by the lateral margin of the pronotum being straight, and by the vertex of the head with a medial fovea, carina or sulcus.

The pale form is similar to *Cephaloleia crenulata* sp. n. It can be distinguished by the smooth lateral margins of the pronotum, by the impunctate vertex of the head, and by antennomere 1 being twice the length of 2.

##### Distribution.

Brazil (Rondonia), Colombia.

##### Type material examined.

Holotype: Colombia, Bananenzone, Tumaco, SW Kíste, 12.IV.1958, Steinhausen [printed label]/ Holotype [red printed label]/ Cephaloleia steinhauseni Uh., Det. E. Uhmann (DEI).

Holotype: Colombia, Bananenzone, Tumaco, 12.IV.1958, Steinhausen [printed label]/ Holotype [red printed label]/ Cephaloleia steinhauseni musae Uh., Det. E. Uhmann (DEI).

##### Specimens examined.

**Brazil:** Rondónia- 62 km SW Ariquemes, Fzda. Rancho Grande, 14 October 1993 (BYUC). Total: 7.

#### Cephaloleia stenosoma

Taxon classificationAnimaliaColeopteraChrysomelidae

Baly, 1885

http://species-id.net/wiki/Cephaloleia_stenosoma

Fig. 239

Cephaloleia stenosoma Baly 1885: 19. Baly 1886: 120 (noted); Blackwelder 1946: 720 (catalog); Papp 1953: 22 (catalog); Uhmann 1957a: 25 (catalog); Descarpentries and Villiers 1959a: 139 (types); Wilcox 1983: 137 (catalog); Staines 1996: 61 (Central America species), 1999: 242 (mimicry), 2004: 312 (host plants), 2010: 36 (types); Staines and Staines 1997: 21 (types), 1999: 524 (Baly species list); McKenna and Farrell 2005: 121 (phylogeny), 2006: 10949 (phylogeny).
Cephalolia stenosoma Baly. Donckier 1899: 551 (catalog); Weise 1911a: 9 (catalog), 1911b: 10 (catalog); Calvert and Calvert 1917: 394 (noted); Uhmann 1930a: 221 (faunal list), 1936b: 483 (key).
Cephalolia stenosoma biolleyi Pic 1926a: 9 (type: Costa Rica, MNHN, not seen).
Cephaloleia stenosoma biolleyi Pic. Blackwelder 1946: 720 (catalog); Papp 1953: 22 (catalog); Uhmann 1957a: 25 (catalog); Descarpentries and Villiers 1959a: 139 (types).

##### Description.

Large, elongate; subparallel; subconvex; head (except frons reddish-yellow), antennae, and scutellum black; pronotum reddish-yellow, often with variable black markings; elytra varies from entirely reddish-yellow, to variable black markings, to entirely black; venter with prosternum red; meso- and metasterna red medially, black laterally, abdominal sterna black; leg with femur pale basally, dark apically, tibiae and tarsi darker. Head: vertex impunctate, with longitudinal Y-shaped medial carina; frons finely punctate, not projecting; not depressed between eyes. Antenna: reaches to humerus; slender; antennomere 1 elongate, robust, clavate, longer than 2 and 3 combined; 2 ovate; 3 longer than 2, compressed, triangular; 4–10 transverse, decreasing in length; 11 2× length of 10, pointed at apex; 1–3 punctate with scattered setae; 4–11 setose. Pronotum: transverse; lateral margin straight then rounding to anterior angle, narrowly canaliculate; anterior angle not produced, acute; posterior angle acute; apical margin straight; disc subconvex, impunctate; surface punctate laterally; basal impression absent; pronotal length 1.3–1.5 mm; pronotal width 1.5–1.6 mm. Scutellum: acutely triangular; alutaceous. Elytron: lateral margin straight, smooth, margined; apex rounded; sutural angle without tooth; humerus rounded, not produced; slightly constricted behind humerus; flattened along suture; moderately punctate-striate, punctation obsolete at humerus, rows converge and unite apically; elytral length 5.4–6.1 mm; elytral width 1.73–2.3 mm. Venter: prosternum impunctate; meso- and metasterna impunctate medially, punctate laterally; abdominal sterna punctate, each puncture with pale seta; suture between sterna 1 and 2 complete; last sternite with apical margin sinuate medially in male, truncate in female. Leg: slender; femur and tibia sparsely punctate; tibia with fringe of setae on inner margin of apex. Total length: 6.8–7.8 mm.

##### Diagnosis.

This species is similar to *Cephaloleia instabilis*. It can be distinguished by antennomere 2 being triangular, by the pronotum being uniformly punctate, and by the elytra lacking a declivity at puncture row 7.

##### Host plant.

Adults have been collected from *Heliconia imbricata* (Kuntze) Baker, *Heliconia latispatha* Benth. (Staines 1996); *Heliconia trichocarpa* G. S. Daniels and F. G. Stiles, *Heliconia wilsonii* G. S. Daniels and F. G. Stiles (Heliconiaceae), *Calathea crotalifera* S. Watson (Marantaceae), *Musa velutina* H. Wendl. and Drude (Musaceae).

##### Distribution.

Costa Rica, Guatemala, Panama.

##### Type material examined.

Lectotype: Bugaba, 800–1,500 ft. Champion [printed label]/ B. C. A. Col. VI, 2. Cephaloleia stenosoma Baly [printed label]/ Lectotype Cephaloleia stenosoma Baly Des. C. L. Staines 1993 [printed red label] (BMNH).

##### Specimens examined.

**COSTA RICA:** no further data (MNHN). Alajuela- Río San Lorencito, 900 m, Res. For. Sn. Ramón, 5 km N Col. Palmarena, March 1990 (INBIO); Safo Dulce, Río Sandalo, Palo Seco, 10 m, 31 December 1923, 21 August 1936 (USNM). Puntarenas- Golfito, 3 July 1976 (EMEC), 22 July 1981 (FSCA); Coto Brus, Las Cruces Biological Station, 6 March 2012, 10 March 2012 (USNM); Est. Sirena, Corcovado NP, 0, 1000m, October 1989, November 1989, December 1989, January 1990, February 1990, March 1990, April 1990, October 1990, June 1991, April 1992 (INBIO); Fca. Las Cruces, San Vito de Java, 11–14 August 1969 (USNM); Est. Queb. Bonita, 50 m, Res. Biol. Carara, 4–26 January 1993 (INBIO); Rancho Quemada, Pen. Osa, November 1989, February 1991, November 1991, 21 March- 7 April 1992, October 1992, November 1992, 1–21 December 1992 (INBIO); Osa Peninsula, 2.5 mi. SW Rincón, 5 March 1967 (CMNC); 3.5 mi. S. Rincón, 28 February- 12 March 1969 (CASC); Río Claro, sea level, 19 August 1969 (USNM); Río Piedras, sea level, 15 August 1969 (USNM); San Vito de Java, 20 July 1972 (FSCA); 6 mi. S. San Vito, 27 June 1969 (USNM); Garabito, Tarcoles, Estación Quebrada Bonita, 100–200 m (INBIO); Estación Esquinas, Peninsula de Osa, 0–100 m (INBIO). **GUATEMALA:** Zacapa- 4–6 km S La Unión, 4600 feet, 2–11 June 1991 (FSCA); 3 km SE La Union, 1500 m, 23 June 1993 (CMNC). **PANAMA:** Chiriquí- Bugaba, 800–1,500 ft. (AMNH, USNM). Colón- Gamboa, 22 June 1975, 18 June 1976 (EGRC). Panamá- Ancón, August 1937 (CASC); La Pita Signal Station Rd., 6 February 1971, 27 February 1971, 18 May 1980 (EGRC); Madden Forest, February 28, 1971, 27 March 1971, 28 June 1976 (EGRC); Pedro Miguel, 17 April 1911 (USNM); Summit, November 1946 (USNM). Total: 125.

#### Cephaloleia stevensi

Taxon classificationAnimaliaColeopteraChrysomelidae

Baly, 1885

http://species-id.net/wiki/Cephaloleia_stevensi

Fig. 240

Cephaloleia stevensi Baly 1885: 26. Blackwelder 1946: 720 (catalog); Papp 1953: 22 (catalog); Uhmann 1957a: 25 (catalog); Wilcox 1983: 137 (catalog); Staines 1996: 61 (Central America species), 2004: 312 (host plants), 2011: 50 (faunal list); Staines and Staines 1999: 524 (Baly species list); Flowers and Hanson 2003: 51 (distribution); McKenna and Farrell 2005: 121 (phylogeny), 2006: 10949 (phylogeny); Meskins et al. 2008: 163 (host plants), 2011: 483 (food web); García–Robledo et al. 2013a: 3 (biology).
Cephalolia stevensi Baly. Donckier 1899: 551 (catalog); Weise 1911a: 9 (catalog), 1911b: 11 (catalog).

##### Description.

Small; elongate; subparallel; subdepressed; head and legs reddish-brown; antennomeres 1–2 reddish-brown, rest darker; pronotum yellow with variable black markings; scutellum black; elytra yellow with variable black oblong markings; venter with pro-, meso-, and metasterna reddish-brown medially, black laterally, abdominal sterna reddish-brown; legs yellowish. Head: vertex densely punctate, faint medial carina present; frons punctate, not projecting; depressed between eyes. Antenna: ½ body length; slender; antennomere 1 elongate, cylindrical longer than 3; 2 transverse, short; 3–5 elongate, cylindrical, subequal in length; 6–10 transverse, subequal in length; 11 longer than 10, pointed at apex; 1–2 punctate with scattered setae; 3–11 setose. Pronotum: transverse; lateral margin straight then rounding to anterior angle, margined; anterior angle rounded, not produced; posterior angle angulate; anterior margin straight; base slightly excavated transversely; disc subconvex; surface strongly, densely punctate; pronotal length 0.7–0.9 mm; pronotal width 0.8–1.0 mm. Scutellum: pentagonal; alutaceous. Elytron: lateral margin straight, smooth, margined; apex rounded; sutural angle without tooth; humerus rounded, not produced; slightly constricted behind humerus; strongly punctate-striate, rows slightly confused at apex; elytral length 2.6–2.7 mm; elytral width 1.2–1.4 mm. Venter: pro-, meso-, and metasterna impunctate medially, punctate laterally; abdominal sterna 1–2 impunctate medially, punctate laterally; sterna 3–5 punctate, each puncture with pale seta; suture between sterna 1 and 2 obsolete medially. Leg: slender; punctate, each puncture with pale seta; tibia with fringe of setae on inner margin of apex. Total length: 3.4–3.8 mm.

##### Diagnosis.

This distinctive species is recognized by the small size, by the vertex of the head being depressed between the eyes, by the densely punctate pronotum, and by the suture between abdominal sterna 1 and 2 being obsolete medially.

##### Host plant.

Adults have been collected on *Heliconia* sp. (Heliconiaceae) leaves (Staines 1996); *Calathea micans* (Mathieru) Koern. (McKenna and Farrell 2005); *Cephaloleia inocephala* (Kuntze), *Cephaloleia latifolia* Klotzsch, *Pleiostachya pruinosa* (W. Bull. ex. Regel) K. Schum. (Meskins et al. 2008); *Calathea venusta* H. Kenn. (Marantaceae) (García–Robledo et al. 2013a); *Tradescantia zanonia* (L.) Sw. (Commelinaceae).

##### Distribution.

Costa Rica, Panama.

##### Type material examined.

Holotype: Bugaba Panama. Champion/ Cephaloleia stevensi/ Sp. figured/ Type H.T [white disk with red border]/ Godman-Salvin Coll, Biol. Centr.-Amer./ Cephaloleia stevensi Baly, Panama (BMNH).

##### Specimens examined.

**COSTA RICA:** Heredia- La Selva, 3.2 Km SE Puerto Viejo, 100 m, 17 February 1992, 19 February 1992, 21 February 1992, 3 March 1992, 21 March 1992 (SEMC), 19 February 1980 (CMNC). Limón- Sector Cerro Cocorí, Fca. de E. Rojas, 150 m, January 1992, December 1992 (INBIO). Puntarenas- Coto Brus, Las Cruces Biological Station, 8 March 2012 (USNM); Rancho Quemado, Peninsula de Osa, 200 m, October 1991, July 1991, September 1992 (INBIO); Quepos, 80 m, P.N. Manuel Antonio, April 1992 (INBIO); Est. Sirena, 0–100 m, P.N. Corcovado, February 1990, October 1991, December 1991 (INBIO); Sirena Station, Corcovado National Park, upper Ollas Trail, 24–28 June 2000 (SEMC); Estación Biológica Las Alturas, 1400–1500 m (INBIO). **PANAMA:** Bocas del Toro- Reserva La Fortuna, 28 May 1993 (EGRC). Canal Zone- Tank Hill, near Albrook Field, 23 February 1971 (EGRC). Chiriquí- Reserva Fortuna, Continental Divide Trail, 25 May 1993 (CDFA). Coclé- Cerro Gaital, 4000 feet, 1 June 1993 (AJGC, CDFA); El Valle, 10–13 June 1985 (EGRC). Panamá- Ancón, 19–21 August 1970 (USNM); Cerro Campana, 30 May 1970, 24 April 1971, 12 March 1972, 11–15 May 1980, 17 May 1993 (EGRC, CDFA); Reserva Sobrina, Pipeline road, 23 May 1993 (CDFA). Total: 44.

#### Cephaloleia strandi

Taxon classificationAnimaliaColeopteraChrysomelidae

Uhmann, 1935b

http://species-id.net/wiki/Cephaloleia_strandi

Fig. 241

Cephalolia strandi Uhmann 1935b: 47.
Cephaloleia strandi Uhmann. Uhmann 1951b: 332 (type), 1957b: 25 (catalog); Gaedike and Döbler 1971: 360 (types); Staines 1997b: 414 (Uhmann species list).

##### Description.

Elongate; sub-obovate; subconvex; head, antennae, and scutellum black; pronotum yellowish; elytra yellowish with variable black markings- with suture black on basal ½ and rounded black macula on each elytron, or black from base reaching to puncture row 5 and expanding apically to puncture row 8, apex always pale; legs yellowish, tarsi darker; venter black, abdomen yellowish laterally. Head: vertex punctate, with medial carina present; frons not projecting; not depressed between eyes. Antenna: reaches beyond humerus; slender; antennomeres elongate, cylindrical; antennomere 1 subincrassate; 2–10 elongate; 3 longest; 2, 4–10 subequal in length; 11 pointed at apex, 2× length of 10; 1–4 punctate with scattered setae; 5–11 setose. Pronotum: transverse; much narrower than base of elytra; lateral margin straight for basal 4/5 then rounding to anterior angle, weakly margined; anterior angle rounded, not produced; posterior angle acute; anterior margin straight; disc subconvex; surface lightly punctate; weak transverse basal impression present; pronotal length 1.0–1.2 mm; pronotal width 1.2–1.4 mm. Scutellum: pentagonal; impunctate. Elytron: lateral margin slightly expanding apically, smooth, weakly margined; apex rounded; sutural angle without tooth; humerus rounded, not produced; slightly constricted behind humerus; moderately punctate-striate, rows converge and unite apically; elytral length 3.6–2.8 mm; elytral width 1.8–2.0 mm. Venter: pro, meso-, and metasterna punctate; abdominal sterna punctate, each puncture with pale seta; suture between sterna 1 and 2 complete; last sternite with apical margin weakly emarginate medially in male, truncate in female. Leg: slender; sparsely punctate; tibia with fringe of setae on inner margin of apex Total length: 4.9–5.2 mm.

##### Diagnosis.

This species is similar to *Cephaloleia dilectans*, *Cephaloleia maxima*, and *Cephaloleia ornatula*. It can be distinguished by the pronotum which is narrower than the base of the elytra with a basal impression.

##### Host plant.

According to the label data, adults have been collected in the flowers of *Butia yatay* (Mart.) Becc. (Arecaceae).

##### Distribution.

Brazil (Minas Gerais, Paraná, Río de Janiero).

##### Type material examined.

Holotype: Brazil, Est. do Río, Itatiaya, 700 m, 2.XI.1929, Zikan [green printed label]/ Holotypus [red printed label]/ Cephalolia strandi Uh., Det. E. Uhmann (DEI).

##### Specimens examined.

**BRAZIL:** Minas Gerais- Corcovado Guanabara, 700 m, 1–7 November 1968 (AMNH). Paraná- Ponta Grossa, October 1943 (USNM). Río de Janiero- Guanabara, October 1963 (USNM). Total: 3.

#### Cephaloleia striata

Taxon classificationAnimaliaColeopteraChrysomelidae

Weise, 1910

http://species-id.net/wiki/Cephaloleia_striata

Fig. 242

Cephalolia striata Weise 1910: 88. Weise 1911a: 9 (catalog), 1911b: 11 (catalog); Uhmann 1936b: 114 (noted), 1942b: 94 (noted).
Cephaloleia striata Weise. Uhmann 1957b: 25 (catalog).

##### Description.

Large; oblong; subdepressed; shining; reddish-brown with eyes and apical four antennomeres darker (except apex of antennomere 11). Head: vertex sparsely punctate, medial carina present; frons with medial carina; frons not projecting; weakly depressed between eyes. Antenna: reaches to humerus; robust; antennomere 1 large, longer than 2 and 3 combined; 2–10 transverse, subequal in length; 11 slightly longer than 10, pointed at apex; 1–2 punctate with scattered setae; 3–11 setose. Pronotum: transverse; lateral margin straight and divergent for basal ½ then rounding to anterior angle, narrowly margined; anterior angle rounded, not produced; posterior angle acute; anterior margin weakly emarginate behind head; disc subconvex; surface sparsely punctate, punctures larger and more dense laterally; basal impression absent; pronotal length 1.5–1.7 mm; pronotal width 2.5–2.7 mm. Scutellum: triangular; impunctate. Elytron: lateral margin straight, smooth, narrowly margined; apex rounded, smooth; sutural angle without tooth; humerus rounded, not produced; constricted behind humerus; moderately punctate-striate, rows 5 and 6 unite and become obsolete apically; interval 4 carinate at base; elytral length 6.0–6.2 mm; elytral width 3.1–3.5 mm. Venter: pro-, meso- and metasterna impunctate medially, punctate laterally; abdominal sterna punctate, each puncture with white seta; suture between sterna 1 and 2 complete; last sternite with apical margin emarginate medially in male, rounded, entire in female. Leg: slender; punctate; tibia with fringe of setae on inner margin of apex. Total length: 7.7–8.1 mm.

**Figures 242–250.**
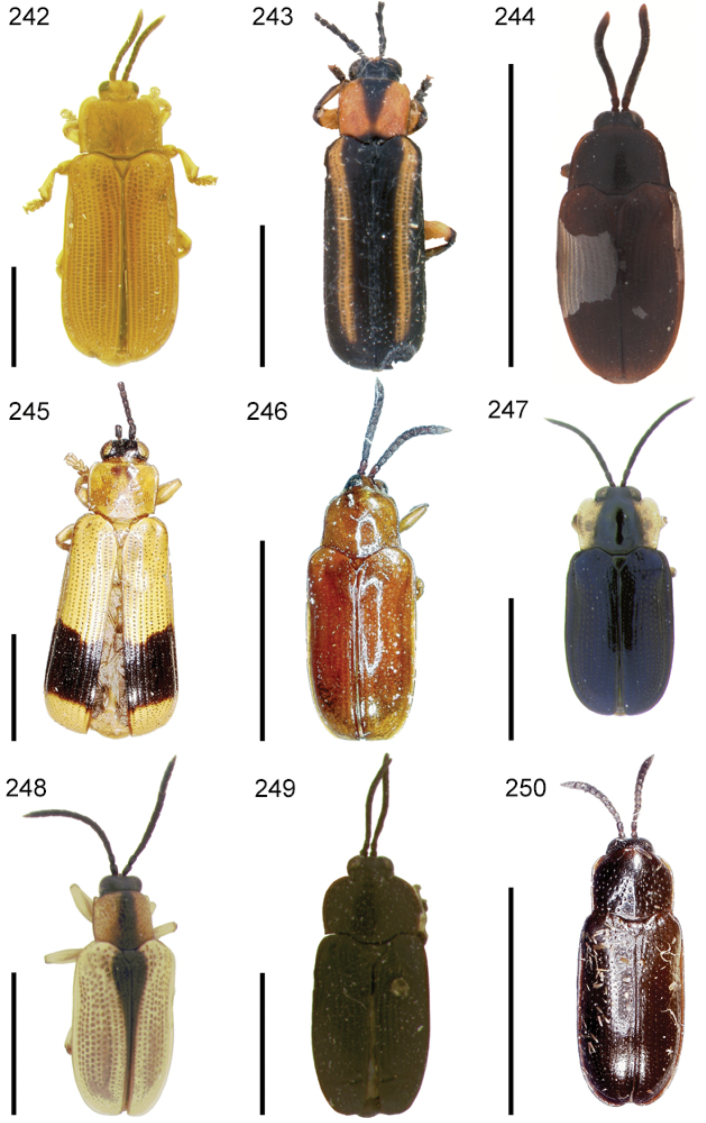
Habitus. **242** *Cephaloleia striata* **243** *Cephaloleia suaveola* **244** *Cephaloleia subdepressa* **245** *Cephaloleia succincta* **246** *Cephaloleia sulciceps* **247** *Cephaloleia susanae* sp. n. **248** *Cephaloleia suturalis* **249** *Cephaloleia tarsata* **250** *Cephaloleia tenella*. Scale bars equal 3 mm.

##### Diagnosis.

This species is similar to *Cephaloleia interstitialis*, *Cephaloleia subdepressa*, *Cephaloleia truncatipennis*, and *Cephaloleia unctula*. It can be distinguished by a transverse basal impression on the pronotum and by antennomere 1 being as long as 2 and 3 combined.

##### Distribution.

Brazil, Ecuador.

##### Type material examined.

Syntype: Südamerika [green printed label]/ J. Weise det. [printed label]/ Type [printed salmon-colored label]/ Cephalolia striata m. [handwritten label] (ZMHB, 1).

##### Specimens examined.

**Ecuador:** Napo- Limonocha, 3 June 1977 (USNM). Total: 6.

#### Cephaloleia suaveola

Taxon classificationAnimaliaColeopteraChrysomelidae

Baly, 1885

http://species-id.net/wiki/Cephaloleia_suaveola

Fig. 243

Cephaloleia suaveola Baly 1885: 23. Blackwelder 1946: 720 (catalog); Papp 1953: 22 (catalog); Uhmann 1957a: 25 (catalog); Wilcox 1983: 137; Staines 1996: 62 (Central America species), 1999: 241 (mimicry); Staines and Staines 1999: 524 (Baly species list); McKenna and Farrell 2005: 121 (phylogeny), 2006: 10949 (phylogeny).
Cephalolia suaveola Baly. Donckier 1899: 551 (catalog); Weise 1905a: 132 (noted), 1911a: 9 (catalog), 1911b: 11 (catalog); Uhmann 1936b: 485 (key).

##### Description.

Elongate; subparallel; subdepressed; head (except yellow frons), antennae, and scutellum black; pronotum yellow with small black macula on anterior margin behind head; elytra black with thin yellow vitta from base to apical 1/5 covering interspace 5 and puncture row 6; venter with pro-, meso-, and metasterna yellow medially, black laterally; abdominal sternite 1 yellow medially then black up to yellow margin, sterna 2–4 black medially, yellow laterally, sternite 5 black; leg with apex of femur, tibia, and tarsi dark. Head: vertex punctate, medial sulcus present; frons finely, densely punctate, not projecting; eyes protruding, finely faceted; slightly depressed between eyes. Antenna: reaches to humerus; slender; antennomere 1 elongate, cylindrical, robust; 2 ⅓ length of 1, elongate, cylindrical; 3 triangular, longer than 2; 4–5 elongate, cylindrical, decreasing in length; 6–8 transverse; 1–2 punctate with scattered setae; 3–8 setose. Pronotum: subquadrate; lateral margin straight then rounding to anterior angle, margined; anterior angle rounded, slightly produced; posterior angle acute; anterior margin straight; disc flattened; surface impunctate; basal impression absent; pronotal length 1.2–1.3 mm; pronotal width 1.4–1.7 mm. Scutellum: elongate triangular; alutaceous. Elytron: lateral margin straight, smooth, narrowly margined; apex rounded, smooth; sutural angle without tooth; humerus rounded, not produced; slightly constricted behind humerus; declivity beginning just behind humerus at puncture row 7 not edged with faint carina; shallowly punctate-striate, punctures in rows 5 and 6 larger than others, rows obsolete apically; elytral length 5.1–5.6 mm; elytral width 2.1–2.3 mm. Venter: pro-, meso-, and metasterna impunctate medially, punctate laterally; abdominal sterna punctate, each puncture with pale seta; suture between sterna 1 and 2 complete. Leg: slender; impunctate; tibia with fringe of setae on inner margin of apex. Total length: 6.4–7.2 mm.

##### Diagnosis.

This species is similar to *Cephaloleia nevermanni* and *Cephaloleia quadrilineata*. It can be distinguished by the elytra not expanding apically and by antennomeres 1 and 2 being elongate.

##### Host plant.

According to label data, adults have been collected in *Heliconia* sp. (Heliconiaceae).

##### Distribution.

Guatemala, Mexico.

##### Type material examined.

Holotype: Type H. T. [white disk with red border]/ Purula, Vera Paz. Champion/ B. C. A., Col. VI, 2. Cephaloleia suaveola, Baly/ Cephaloleia suaveola Baly (BMNH).

##### Specimens examined.

**MEXICO:** Veracruz- Cordoba (CASC). Total: 2.

#### Cephaloleia subdepressa

Taxon classificationAnimaliaColeopteraChrysomelidae

Baly, 1878

http://species-id.net/wiki/Cephaloleia_subdepressa

Fig. 244

Cephaloleia subdepressa Baly 1878: 41. Uhmann 1957b: 26 (catalog); Staines and Staines 1999: 524 (Baly species list).
Cephalolia subdepressa Baly. Donckier 1899: 551 (catalog); Weise 1911a: 9 (catalog), 1911b: 11 (catalog).

##### Description.

Small; elongate-oval; flattened; reddish-brown with margins of pronotum and elytra paler; head and antennae dark, except basal antennomeres. Head: vertex densely punctate, medial carina present; frons impunctate, not projecting; slightly depressed between eyes. Antenna: reaches to humerus; slender; antennomeres 1–3 cylindrical; 1–2 subequal in length; 3 longer than 1 or 2; 4 shorter than 3; 5–10 transverse, decreasing in length; 11 2× length of 10, rounded at apex; 1–3 punctate with scattered setae; 4–11 setose. Pronotum: transverse; lateral margin straight, slightly divergent at basal ½ then rounding to anterior angle, slightly canaliculate; anterior angle rounded, not produced; posterior angle acute; anterior margin curved anteriorly; basal margin biangulate; disc subconvex; surface sparsely coarsely punctate, more so laterally; basal impression absent; pronotal length 0.5–0.7 mm; pronotal width 0.7–0.9 mm. Scutellum: triangular; impunctate. Elytron: lateral margin straight, smooth, narrowly margined; apex rounded; sutural angle without tooth; humerus rounded, not produced; slightly constricted behind humerus; moderately punctate-striate, rows converge and unite apically; elytral length 1.9–2.1 mm; elytral width 0.9–1.1 mm. Venter: pro-, meso-, and metasterna punctate; abdominal sterna punctate, each puncture with pale seta; suture between sterna 1 and 2 complete; last sternite with apical margin concave-emarginate medially in male, rounded entire in female. Leg: slender; punctate, each puncture with pale seta; tibia with fringe of setae on inner margin of apex. Total length: 2.5–2.8 mm.

##### Diagnosis.

This species is similar to *Cephaloleia interstitialis*, *Cephaloleia striata*, *Cephaloleia truncatipennis*, and *Cephaloleia unctula*. It can be distinguished by the pronotum lacking a transverse basal impression and by antennomere 1 being subequal in length to 2.

##### Distribution.

Brazil (Minas Gerais).

##### Type material examined.

Syntype: Banks of the Amazon [handwritten label]/ Cephaloleia subdepressa Baly, Banks of Amazon [blue handwritten label] (BMNH, 1).

#### Cephaloleia succincta

Taxon classificationAnimaliaColeopteraChrysomelidae

Guérin-Méneville, 1844

http://species-id.net/wiki/Cephaloleia_succincta

Fig. 245

Cephaloleia succincta Guérin–Méneville 1844: 282. Baly 1858: 58 (redescription); Uhmann 1957b: 26 (catalog), 1966d: 269 (noted).
Cephalolia succincta Guérin-Méneville. Gemminger and Harold 1876: 3602 (catalog); Donckier 1899: 551 (catalog); Weise 1911a: 9 (catalog), 1911b: 11 (catalog).

##### Description.

Large; elongate; subparallel; subdepressed; yellowish; head, antennae, pronotal macula (may be absent), and transverse elytral vitta on apical ½ black. Head: vertex finely, moderately punctate, faint medial sulcus present; frons slightly projecting; slightly depressed between eyes. Antenna: reaches to humerus; slender; cylindrical; antennomere 1 incrassate, longest; 2 less than ½ length of 1; 3 elongate, 2× length 2; 4–6 transverse, decreasing in length. Pronotum: quadrate; lateral margin straight for basal ¾ then rounding to anterior angle, narrowly margined; anterior angle obtuse, not produced; posterior angle acute; anterior margin straight; disc subconvex; surface sparsely, irregularly punctate; transverse basal impression present; pronotal length 1.8 mm; pronotal width 2.0 mm. Scutellum: pentagonal; impunctate. Elytron: lateral margin straight, smooth, narrowly margined; apex rounded; sutural angle with minute tooth; humerus rounded, slightly produced; slightly constricted behind humerus; disc convex; moderately punctate-striate, punctures confused at apex; elytral length 6.4 mm; elytral width 2.7 mm. Venter: pro-, meso-, and metasterna impunctate; abdominal sterna punctate, each puncture with pale seta; suture between sterna 1 and 2 complete; last sternite with apical margin slightly concave-emarginate in male, sinuate in female. Leg: slender; sparsely punctate; tibia with fringe of setae on inner margin of apex. Total length: 8.5 mm.

##### Diagnosis.

This species is similar to *Cephaloleia alternans* and *Cephaloleia nana* sp. n. It can be distinguished by the canaliculate lateral margins of the pronotum.

##### Distribution.

Colombia.

##### Type material.

Type: Colombia (depository unknown, not examined).

##### Specimens examined.

No label data (USNM). Total: 1.

#### Cephaloleia sulciceps

Taxon classificationAnimaliaColeopteraChrysomelidae

Baly, 1885

http://species-id.net/wiki/Cephaloleia_sulciceps

Fig. 246

Cephaloleia sulciceps Baly 1885: 26. Blackwelder 1946: 720 (catalog); Papp 1953: 22 (catalog); Uhmann 1957a: 26 (catalog); Descarpentries and Villiers 1959a: 139 (types); Wilcox 1983: 138 (catalog); Staines 1996: 62 (Central America species), 2011: 51 (faunal list); Staines and Staines 1997: 21 (types), 1999: 524 (Baly species list).
Cephalolia sulciceps Baly. Donckier 1899: 551 (catalog); Weise 1911a: 9 (catalog), 1911b: 11 (catalog); Uhmann 1936a: 114 (noted).

##### Description.

Small; elongate; slightly narrowing apically; subconvex; reddish-brown, eyes darker; antennae with antennomeres 1–2 reddish-brown, 3–11 black. Head: vertex impunctate, with two deep, longitudinal sulci which reach antennal bases, longitudinal carina present between sulci; frons not projecting; depressed between eyes. Antenna: as long as head and pronotum combined; robust; antennomeres 1–2 short, subequal in length; 1 robust, clavate; 2 cylindrical; 3–10 transverse, decreasing in length, 3 shorter than 1 or 2; 2× length of 10, 11 rounded at apex; 1–2 impunctate, glabrous; 3–11 setose. Pronotum: transverse; lateral margin straight from base to beyond middle then rounding to anterior angle, canaliculate; anterior angle broadly rounded, slightly produced; posterior angle acute; apical margin straight; disc subconvex; surface sparsely punctate with shallow punctures; basal impression absent; pronotal length 0.7–1.0 mm; pronotal width 1.1–1.3 mm. Scutellum: subcordate, apex acute; impunctate. Elytron: lateral margin straight, smooth, margined; apex rounded; sutural angle without tooth; humerus rounded, not produced; slightly constricted behind humerus; shallowly punctate-striate, punctures large, rows converge and unite apically; elytral length 2.6–3.1 mm; elytral width 1.4–1.6 mm. Venter: pro- and mesosterna impunctate; metasternum impunctate medially, punctate laterally; abdominal sterna impunctate, glabrous; suture between sterna 1 and 2 obsolete medially. Leg: slender; impunctate; tibia with fringe of setae on inner margin of apex. Total length: 3.6–4.1 mm.

##### Diagnosis.

This species is similar to *Cephaloleia mauliki*, *Cephaloleia placida*, and *Cephaloleia simplex*. It can be distinguished by the suture between abdominal sterna 1 and 2 being obsolete medially and by antennomere 1 being subequal in length to 2.

##### Distribution.

Costa Rica, Panama.

##### Type material examined.

Syntype: Bugaba, 800–1500 ft., Champion/ paratipos [red label]/ F. Monros Collection 1959/ Cephaloleia sulciceps Baly, J. S. Baly det. [pink label] (USNM, 1).

##### Specimens examined.

**COSTA RICA:** Cartago- Turrialba (USNM), 4–13 August 1970 (USNM). Limón- Sector Cerro Cocorí, Fca. de E. Rojas, 150 m, November 1991 (INBIO). Puntarenas- Est. Sirena, 0–100 m, P.N. Corcovado, March-June 1991 (INBIO); Los Alturas Field Station, 20 km N San Vito de Java, 3–7 June 1991 (AMNH). **PANAMA:** Colón- Paraiso, 5 February 1911, 4 March 1911, 7 May 1911 (USNM). Panamá- Barro Colorado Is., no date (USNM); Summit, 29 November 1965 (AMNH). Total: 20.

#### Cephaloleia susanae

Taxon classificationAnimaliaColeopteraChrysomelidae

Staines sp. n.

http://zoobank.org/12569A54-87B9-49EE-B894-28C99A91713D

http://species-id.net/wiki/Cephaloleia_susanae

Fig. 247

##### Description.

Elongate; flattened; antennae, head, and elytra shining bluish-black; pronotum yellowish with wide medial longitudinal black vitta from anterior to posterior margins; venter paler, pro-, meso- and metasterna darker medially, abdomen brownish; legs yellow. Head: vertex sparsely, irregularly punctate, medial carina present; triangular projection present between antennal bases; frons not projecting; slightly depressed between eyes. Antenna: reaches to humerus; slender; elongate, cylindrical; antennomeres 1 and 2 subequal in length and width; 3 1¾ length 2; 4–10 subequal in length, each shorter than 2; 11 slightly longer than 10, rounded at apex; 1–3 punctate with scattered setae; 4–11 setose. Pronotum: transverse, lateral margin sinuate then rounding to anterior angle, serrulate; anterior angle rounded, slightly produced; posterior angle acute; anterior margin emarginate behind head; disc subconvex; surface irregularly punctate, more dense basally; basal impression absent; pronotal length 1.1 mm; pronotal width 1.4 mm. Scutellum: pentagonal; impunctate. Elytron: lateral margin straight, finely serrulate, margined; apex rounded, finely serrate; sutural angle without tooth; humerus rounded, not produced; slightly constricted behind humerus; moderately punctate-striate, rows converge and unite apically; space between row 10 and lateral margin; elytral length 3.6 mm; elytral width 2.0 mm. Venter: prosternum rugose medially, impunctate laterally; mesosternum punctate; metasternum rugose basally, rest impunctate; abdominal sterna punctate, each puncture with pale seta; suture between sterna 1 and 2 complete; last sternite with apical margin emarginate medially in male, rounded in female. Leg: slender; femur coarsely punctate basally; tibia with fringe of setae on inner margin of apex. Total length: 4.9 mm.

##### Etymology.

Named for Susan L. Staines in acknowledgement of her constant, continuing encouragement and help in taxonomic projects for the last 30 years.

##### Diagnosis.

This species is similar to *Cephaloleia abdominalis*, *Cephaloleia amazona*, *Cephaloleia princeps*, *Cephaloleia steinhauseni*, and *Cephaloleia teutonica*. It can be distinguished by the straight lateral margins of the pronotum, by the vertex of the head having a medial carina, by the pronotum with a medial black longitudinal vitta, and by the finely serrulate lateral margins of the elytra.

##### Distribution.

Brazil (Pará), Ecuador.

##### Type material.

Holotype female: Holotype- Brazil, Santarem/ F. Monros collection 1959 / Holotype Cephaloleia susanae Staines, des. C. L. Staines 2012 [red label] (USNM). Paratypes (2 males, 1 female) each with Paratype Cephaloleia susanae Staines, des. C. L. Staines 2012 [red label]) (USNM): Ecuador, Orellana, Tiputini Biodiversity Station, nr. Yasuni NP, 220–250 m, 1 July 1998; Ecuador, Orellana, 1 km S Onkone Gore Camp, Reserva Etnica Waorani, 216.3 m, 24 January 1994; Ecuador, Orellana, 1 km S Onkone Gore Camp, Reserva Etnica Waorani, 216.3 m, 26 October 1998.

#### Cephaloleia suturalis

Taxon classificationAnimaliaColeopteraChrysomelidae

Baly, 1885

http://species-id.net/wiki/Cephaloleia_suturalis

Fig. 248

Cephaloleia suturalis Baly 1885: 14. Maulik 1937: 132 (host plants); Blackwelder 1946: 720 (catalog); Papp 1953: 22 (catalog); Uhmann 1957a: 26 (catalog); Wilcox 1983: 138 (catalog); Maes and Staines 1991: 36 (faunal list); Staines 1996: 63 (Central America species), 1996(1997): 16 (Nicaragua species), 2004: 312 (host plants), 2011: 51 (faunal list); Flowers and Janzen 1997: 353 (host plant); Maes 1999: 1017 (faunal list); Staines and Staines 1999: 524 (Baly species list); McKenna and Farrell 2005: 121 (phylogeny), 2006: 10949 (phylogeny); Meskins et al. 2008: 163 (host plants), 2011: 483 (food web).
Cephalolia suturalis Baly. Donckier 1899: 551 (catalog); Weise 1911a: 9 (catalog), 1911b: 12 (catalog); Uhmann 1930a: 226 (faunal list), 1930f: 161 (museum list).
Cephaloleia histronica Baly. Strong 1977a: 163 (misidentification); Staines 2004: 312 (identification).

##### Description.

Elongate; subparallel; subconvex; head, antennae, and scutellum black; pronotum yellowish with medial black vitta from base to apex; elytra yellowish with black sutural vitta from base to ¾ length, gradually narrows to apex; venter yellowish with pro-, meso-, and metasterna dark reddish-brown medially, abdominal sterna paler reddish-brown; leg yellowish-brown. Head: vertex densely punctate, medial longitudinal carina present; frons not projecting; not depressed between eyes. Antenna: reaches to humerus; slender; antennomere 1 elongate, subclavate; 2 oblong-ovate, ⅔ length of 1, rugose; 3 elongate, cylindrical, subequal in length to 1; 4–5 elongate, cylindrical, shorter than 2 or 3; 6–10 transverse, decreasing in length; 11 2× length of 10, pointed at apex; 1–3 punctate with scattered setae; 4–11 setose. Pronotum: slightly wider than long; lateral margin straight for basal ¾ then rounding to anterior angle, slightly canaliculate anteriorly; anterior angle obtuse, produced; posterior angle acute; anterior margin straight; disc subconvex; surface covered with large, deep punctures, medial line on disc nearly impunctate; basal impression absent; pronotal length 1.0–1.3 mm; pronotal width 1.3–1.6 mm. Scutellum: pentagonal; alutaceous. Elytron: lateral margin straight, smooth, margined; apex obtusely rounded; sutural angle without tooth; humerus rounded, not produced; slightly constricted behind humerus; subconvex; strongly punctate-striate, humerus almost impunctate, rows converge and unite apically; elytral length 3.6–4.4 mm; elytral width 2.0–2.1 mm. Venter: pro-, meso-, and metasterna impunctate medially, punctate laterally; abdominal sterna punctate, each puncture with pale seta; suture between sterna 1 and 2 obsolete medially; last sternite with apical margin truncate in male, rounded, entire in female. Leg: slender; punctate, each puncture with pale seta; tibia with fringe of setae on inner margin of apex. Total length: 4.9–5.1 mm.

##### Diagnosis.

This species is similar to *Cephaloleia balyi*, *Cephaloleia deficiens*, *Cephaloleia discoidalis*, *Cephaloleia dorsalis*, and *Cephaloleia linkei*. It can be distinguished by the yellowish pronotum with a black vitta and by antennomere 1 being clavate and subequal in length to 3.

##### Host plant.

*Costus malortieanus* Wendl. (Uhmann 1930a); *Costus* sp. (Maulik 1937); *Cephaloleia pulverulentus* C. Presl. (Meskins et al. 2008); *Cephaloleia laevis* Ruiz. and Pav. (Costaceae).

##### Distribution.

Costa Rica, Guatemala, Nicaragua.

##### Type material examined.

Syntype: Type H. T. [white disk with red border]/ Guatemala: Sinanja, Sabo, Cubilguitz, Champion/ B. C. A., Col. VI, 2. Cephaloleia suturalis, Baly/ Cephaloleia/ Cephaloleia suturalis Baly, Guatemala [blue handwritten label] (BMNH, 1).

##### Specimens examined.

**COSTA RICA:** Alajuela- road to Arenal Lodge, 2 September 1998 (BYUC); Fca. San Gabriel, 600 m, 2 km SO de Dos Ríos, 14 June 1991 (INBIO); Caño Negro, 0–100 m (INBIO); Upala, Sector San Ramón de Dos Ríos, 1.5 km NW Hacienda Nueva Zelandia, 600–700 m (INBIO). Cartago- Peralta, 26 January 1933 (USNM); Quebrada Segunda Ref. Nac. Fauna Silv. Tapantí, 1250 m, March 1992, April 1992 (INBIO); Tabacon Hot Springs, 2 September 1998 (BYUC); CATIE Turrialba, 26–29 June 1986 (BYUC); Turrialba, 640 m, 10 October 1981 (CMNC), 4–13 August 1970 (USNM); 40 km NE Turrialba, 18 May 1979, 19 May 1979, 20 May 1979 (CMNC); Tuis River, 21 May 1991 (CDFA). Guanacaste- Est. Pitilla, 700 m, 9 km S Sta Cecilia, P. N. Guanacaste, February 1990, 24 August- 11 September 1992, 22 September- 14 October 1992, October- 8 November 1992, 21 March- 6 April 1993, 22 18 April- 19 May 1993 (INBIO); Río San Lorenzo, 1050 m, Tierras Morenas, Z. P. Tenorio, 23 March- 21 April 1992 (INBIO). Heredia- Est. El Ceibo, Braulio Carillo N.P., November 1989 (INBIO); Est. Biol. La Selva, 21 January 1989, 23 January 1989 (MUCR); La Selva Biol. Sta., 2 km S. Pt. Viejo, 3–5 June 1984 (EGRC); 1 km S. Pt. Viejo, 4–5 June 1984 (EGRC); Rara Avis Biological Station, 20 November 011 (USNM). Limón- Sector Cerro Cocorí, Fca. de E. Rojas, 150 m, November 1991, October 1991, January 1992, March 1992, August 1991, 31 January- 21 February 1992, 28 May- 17 June 1992, 26 June- 16 July 1992, 11 October 1992, December 1992, 12–31 August 1992, 10–30 September 1992, October 1992, 9–30 November 1992, January 1993, February 1993, March 1993, April 1993, May 1993 (INBIO); Cerro Tortuguero, 0–120 m, P. N. Tortuguero, April 1989, March 1993 (INBIO); Est. Cuatro Esquinas, P.N. Tortuguero, 0 m, September 1989, November 1989 (INBIO); 16 km W Guápiles, 400 m, May 1989 (USNM); 7 mi W Guacimo, 22 February- 3 March 1988 (BYUC); Hamburg Farm, Reventazón, Ebene Limón, 15 December 1921, 15 November 1922, 9 July 1924, 24 January 1925, July 1925, 1 August 1929, 28 January 1933, 24 February 1935, 25 May 1936, 23 January 1936, 18 December 1936, 24 July 1937 (USNM), 3 January 1925 (DEI), 1 March 1926 (MUCR); Manzanillo, 0–100 m RNFS, Gandoca y Manzanillo, May 1991, January 1992 (INBIO); Las Mercedes, 12 November 1922 (DEI); Río Sardinas, 10 m, R.N.F.S., Barra del Colorado, September 1992, 10 October 1992 (INBIO); Waldeck, 22 July 1928 (USNM). Puntarenas- Fca. Cafrosa, Est. Las Mellizas, P. N. Amistad, 1300 m, October 1989 (INBIO); Est. Pitilla, 700 m, 9 km S Sta. Cecilia, P.N. Guanacaste, 1988, March 1991, May 1991, July 1991, 3–8 October 1991, 4–25 November 1991, 4–13 December 1991, 2–9 March 1992 (INBIO); 23 km W Piedras Blancas, April-May 1989 (USNM); San Lorenzo, 1050 m, Tierras Morenas, Z. P. Tenorio, July 1991 (INBIO); Estación Biológica Las Alturas, 1400–1500 m (INBIO); Estación Esquinas, Peninsula de Osa, 0–100 m (INBIO). San José- Hacienda el Rodeo, Universidad, 800–900 m (INBIO). **GUATEMALA:** Izabel- San Gil, 3 km N Las Eschas, 11 June 1993 (CMNC). Total: 363.

#### Cephaloleia tarsata

Taxon classificationAnimaliaColeopteraChrysomelidae

Baly, 1858

Fig. 249

Cephaloleia tarsata Baly 1858: 60. Uhmann 1957b: 26 (catalog); Staines and Staines 1999: 524 (Baly species list).
Cephalolia tarsata Baly. Gemminger and Harold 1876: 3602 (catalog); Donckier 1899: 551 (catalog); Weise 1911a: 9 (catalog), 1911b: 12 (catalog).

##### Description.

Elongate; subparallel; subdepressed; dark metallic blue, pronotum paler laterally, legs paler; venter black; tarsi yellowish. Head: vertex punctate with faint medial carina; frons not projecting; depressed between eyes. Antenna: reaches to humerus; slender; antennomere 1 subincrassate, elongate, widening apically; 2 ½ length of 1; 3 elongate, cylindrical, subequal in length to 1; 4–10 elongate, cylindrical, subequal in length, each ¾ length of 3; 11 2× length of 10, rounded at apex; 1–4 punctate with scattered setae; 5–11 setose. Pronotum: transverse; lateral margin subsinuate basally then rounding to anterior angle, narrowly margined, slightly serrulate; anterior angle obtusely rounded, slightly produced; posterior angle with short obtuse tooth, produced; anterior margin emarginate behind head; disc subconvex; surface with coarse, deep punctures, punctures finer on disc; basal impression absent; pronotal length 1.3–1.4 mm; pronotal width 2.0–2.1 mm. Scutellum: pentagonal; impunctate. Elytron: lateral margin straight, narrowly margined, slightly reflexed, finely serrulate; apex obtusely rounded, emarginate at suture; sutural angle with small tooth; humerus rounded, not produced; slightly constricted behind humerus; disc flattened; moderately punctate-striate, rows converge and unite apically; pygidium narrowed from base to apex, truncate; elytral length 3.8–3.9 mm; elytral width 2.1–2.2 mm. Venter: pro-, meso-, and metasterna punctate; abdominal sterna sparsely punctate, each puncture with pale seta; suture between sterna 1 and 2 complete; last sternite with apical margin truncate in female, broadly emarginate medially in male. Leg: slender; punctate; tibia with fringe of setae on inner margin of apex. Total length: 5.5–5.6 mm.

##### Diagnosis.

This species is similar to *Cephaloleia depressa*, *Cephaloleia donckieri*, *Cephaloleia elaeidis*, and *Cephaloleia zikani*. It can be distinguished by the lack of a medial fovea on the vertex of the head, by the serrulate lateral margins of the pronotum, by the smooth apical margin of the elytra, and by antennomere 2 being obconic.

##### Distribution.

Brazil, Colombia.

##### Type material examined.

Holotype: Colombia, Baly coll. (BMNH).

##### Specimens examined.

**BRAZIL:** ?- Chapada (USNM). Total: 1.

#### Cephaloleia tenella

Taxon classificationAnimaliaColeopteraChrysomelidae

Baly, 1885

http://species-id.net/wiki/Cephaloleia_tarsata

Fig. 250

Cephaloleia tenella Baly 1885: 26. Blackwelder 1946: 720 (catalog); Papp 1953: 22 (catalog); Uhmann 1957a: 26 (catalog), 1961: 24 (noted), 1963: 148 (museum list), 1968: 248 (faunal list); Wilcox 1983: 138 (catalog); Staines 1996: 64 (Central America species), 2011: 51 (faunal list); Staines and Staines 1999: 524 (Baly species list); Flowers and Hanson 2003: 51 (distribution); McKenna and Farrell 2005: 121 (phylogeny), 2006: 10949 (phylogeny).
Cephalolia tenella Baly. Donckier 1899: 551 (catalog); Weise 1911a: 10 (catalog), 1911b: 13 (catalog); Uhmann 1930a: 230 (faunal list), 1942: 94 (noted).

##### Description.

Small, elongate; subparallel; subdepressed; black, pronotum and elytra with paler margins. Head: vertex densely punctate, medial sulcus absent; frons densely punctate, not projecting; not depressed between eyes. Antenna: slightly longer than head and pronotum combined; slender, slightly thickened apically; antennomeres 1–2 transverse, subequal in length, 1 not thickened; 3 elongate, slightly longer than 1 or 2; 4–10 transverse, subequal in length, each shorter than 2; 11 2× length of 10, pointed at apex. Pronotum: slightly wider than long; lateral margin straight then abruptly rounding to anterior angle, canaliculate; anterior angle rounded, slightly produced; posterior angle angulate; anterior margin sinuate; disc transversely subconvex; surface deeply but not densely punctate; basal impression absent; pronotal length 0.7–0.9 mm; pronotal width 1.0–1.1 mm. Scutellum: pentagonal; alutaceous. Elytron: lateral margin straight, smooth, margined; apex obtusely rounded; sutural angle without tooth; humerus rounded, not produced; slightly constricted behind humerus; shallowly punctate-striate, rows converge and unite apically; elytral length 2.6–2.7 mm; elytral width 1.3–1.4 mm. Venter: pro-, meso-, and metasterna impunctate medially, punctate laterally; abdominal sterna punctate, each puncture with pale seta; suture between sterna 1 and 2 complete; last sternite with apical margin emarginate medially in male, truncate in female. Leg: slender; punctate, each puncture with pale seta; femur robust; tibia with fringe of setae on inner margin of apex. Total length: 3.4–3.6 mm.

##### Diagnosis.

This small black species is recognized by the head not being depressed between the eyes, by the vertex of the head being densely punctate, and by the canaliculate lateral margins of the pronotum.

##### Host plant.

According to the label data, adults have been collected on palm leaves (Areaceae) (Staines 1996).

##### Distribution.

Costa Rica, El Salvador, Guatemala, Mexico, Nicaragua, Panama.

##### Type material examined.

Holotype: Type H. T. [white disk with red border]/ Guatemala, Coatepeque, Champion/ B. C. A., Col. VI, 2. Cephaloleia tenella, Baly/ Cephaloleia/ Cephaloleia tenella Baly, Guatemala [blue handwritten label] (BMNH).

##### Specimens examined.

**COSTA RICA:** ?- Bataan, 16 June 1954 (USNM). Alajuela- Peñas Blancas, 7 July 1987 (USNM); 20 km S Upala, 1–9 May 1991 (BYUC); Caño Negro, 0–100 m (INBIO); Upala, Sector San Ramón de Dos Ríos, 1.5 km NW Hacienda Nueva Zelandia, 600–700 m (INBIO). Cartago- Turrialba, 20 June 1974 (CASC). Guanacaste- Est. Cacao, 1000–1400 m, SW side Volcán Cacao, November 1989 (INBIO); 14 km S Cañas, 9–14 March 1990 (BYUC); Cerro El Hacha, 300 m, 12 km SE de La Cruz, 6 May 1991 (INBIO); Est. Maritza, 600 m, Lado oeste del Volcán Orosí, August 1990 (INBIO); Est. Pitilla, 700 m, 9 km S Sta. Cecilia, P.N. Guanacaste, September 1989, 3–18 October 1991, 4–25 November 1991, 31 March- 29 April 1992 (INBIO); 3 km SE Río Naranjo, 29 May 1992, 1–5 June 1992 (BYUC); La Cruz, P.N. Guanacaste, Agua Buena, 200–300 m (INBIO); La Cruz, La Garita, Estación Los Almendros, 200–300 m (INBIO); Estación Los Almendros, 12 km Carretera Santa Cecilia, 200–300 m (INBIO); La Cruz, Santa Elena, P.N. Santa Rosa, Estación Murciélago, 8 km SW Cuajiniquil, 0–100 m (INBIO); Sector Las Pailas, 4.5 km SW Volcán Rincón de la Vieja, 800–900 m (INBIO); Sector Santa Maria, 6 km S Volcán Santa Maria, 800–900 m (INBIO); Parque Nac Santa Rosa, Est. Biol. Pitilla, 700 m, 16 August 1991 (BYUC). Heredia- Est. Barva, P.N. Braulio Carillo, 2500 m, May 1990 (INBIO); Chilamate, 18–23 August 1988 (BYUC); F. La Selva, 3 km S Pto. Viejo, 1 September 1998 (BYUC). Limón- Sector Cerro Cocorí, Fca. de E. Rojas, 150 m, December 1990, October 1991 (INBIO); Est. Hitoy Cerere, 100 m, R. Cerere, Res. Biol. Hitoy Cerere, 27 June- 22 July 1992 (INBIO); Hamburg Farm, Reventazón, 12 November 1932 (USNM); A.C. Llanuras del Tortuguero, Pococí, Río Sardinas, 0–100 m (INBIO). **EL SALVADOR:** San Salvador, 14 June 1958, 21 June 1958 (CNC). **GUATEMALA:** Quezalten, 25, 2 km SW Zunil, 800 m, 20 June 1993 (CMNC); Retalhuleu- 4 m E Retalhuleu, 4 September 1972 (USNM); Yepocapa, 20 April 1948 (USNM). **MEXICO:** ?- Bilimek, 1871 (DEI). Chiapas- Ocosingo Rd., 76 km S. Palenque, Rt. 195, 5–29 July 1983 (CNC). Colima- 9 mi. NE Comala, 18–19 July 1983 (TAMU). Hildago- 3 mi. W Hild. and S.L.P. border on 85, 25 July 1979 (EGRC). Nayarit- San Blas, 24–26 April 1961 (CNC); Tepic, 21–24 September 1953 (CASC); 25 mi. N. Tepic, 24 April 1961 (CNC). Tamaulipas- El Cielito, nr. Encino, 28–30 August 1985 (EGRC); along rd to Rancho de Cielo, 1–3 mi W Gomez Farias, 21 May 1979 (EGRC). Veracruz- 1962–63 (DEI); 11 mi. S. Misantla, 24 July 1984 (TAMU). **NICARAGUA:** Granda- Volcán Mombacho, Finca Santa Ana, 2 June 1998 (USNM). **PANAMA:** ?- El Cermeco, April-May 1939 (USNM). Canal Zone- September 1938, December 1960 (DEI); Peristeria elata, 19 October 1938 (USNM); Tank Hill near Albrook Field, 8 April 1971 (EGRC). Chiriquí- Reserva Fortuna, Continental Divide trail, 29 May 1993 (CDFA). Coclé- El Valle, 1 July 1973 (USNM), 15 May 1980 (EGRC). Colón- Paraiso, 16 January 1911, 18 January 1911, 27 January 1911, 11 February 1911, 26 March 1911, 28 March 1911, 30 March 1911, 26 April 1911 (USNM); Frijoles, 23 October 1918 (USNM). Panamá- 12 April 1911 (USNM), 19–31 August 1937 (CASC); Ancón, 19 August 19?8 (USNM), 19–21 August 1970, 24 August 1970 (USNM), 20 March 1920 (USNM); Barro Colorado, January 1938 (USNM), April-May 1939 (USNM); Cerro Campana, 18 July 1976 (USNM), 11–15 May 1980 (EGRC); Corozal, 14 January 1953 (USNM), 3 January 1953 (DEI); Diablo Heights, 2 March 1971, 24 April 1971 (EGRC); Fort Clayton, April 1944 (CASC), 16 September 1969 (EGRC); Ft. Kobbe, 4 October 1969, 1 May 1971 (EGRC); Skunk Hollow, nr Ft. Sherman, 28 May 1980 (EGRC); La Pita Signal Station rd, 16 May 1980 (EGRC); Summit, July 1953 (USNM), August 1946 (USNM); Tabernilla, 20 June 1907 (USNM). Total: 162.

#### Cephaloleia tetraspilota

Taxon classificationAnimaliaColeopteraChrysomelidae

Guérin-Méneville, 1844

http://species-id.net/wiki/Cephaloleia_tetraspilota

Fig. 251

Cephaloleia tetraspilota Guérin–Méneville 1844: 282. Baly 1858: 63 (redescription); Uhmann 1957b: 26 (catalog), 1966d: 269 (noted); McKenna and Farrell 2005: 121 (phylogeny), 2006: 10949 (phylogeny).
Cephalolia tetraspilota Guérin-Méneville. Gemminger and Harold 1876: 3602 (catalog); Donckier 1899: 551 (catalog); Weise 1911a: 10 (catalog), 1911b: 12 (catalog); Uhmann 1931f: 219 (museum list).

##### Description.

Elongate; subparallel; subdepressed; black; antennae and legs paler; elytra with rounded ocher-yellow macula behind humeri and apical ¼ ocher-yellow; abdominal sterna yellowish; leg with base of femur brownish. Head: vertex strongly punctate, with medial carina; frons impunctate, not projecting; not depressed between eyes. Antenna: reaches beyond humerus; slender; antennomere 1 elongate, cylindrical, slightly longer than 2; 2–5 subequal in length, each ¾ length of 1, subelongate; 6–10 transverse, subequal in length, each shorter than 5; 11 2× length of 10, pointed at apex; 1–2 punctate with scattered setae; 4–11 setose. Pronotum: transverse; lateral margin straight for basal 4/5 then broadly rounded to anterior angle, narrowly margined; anterior angle rounded, produced; posterior angle acute; anterior margin weakly emarginate behind head; disc subconvex; surface strongly sparsely punctate; basal impression absent; pronotal length 1.3–1.5 mm; pronotal width 1.4–1.6 mm. Scutellum: pentagonal; impunctate. Elytron: lateral margin straight, smooth, margined; apex rounded, smooth; sutural angle without tooth; humerus rounded, not produced; slightly constricted behind humerus; shallowly punctate-striate, rows converge and unite apically; elytral length 4.0–4.4 mm; elytral width 2.2–2.5 mm. Venter: prosternum punctate; meso- and metasterna impunctate medially, punctate laterally; abdominal sterna punctate, each puncture with pale seta; suture between sterna 1 and 2 complete. Leg: slender; punctate, each puncture with pale seta; tibia with fringe of setae on inner margin of apex. Total length: 5.0–5.7 mm.

**Figures 251–259.**
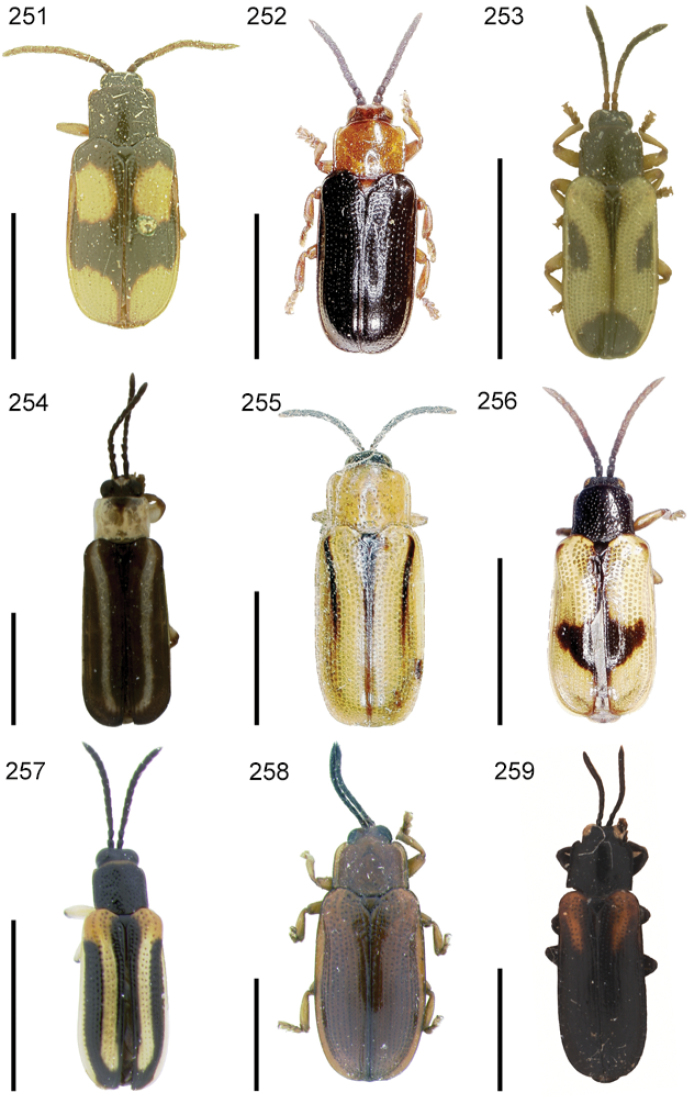
Habitus. **251** *Cephaloleia tetraspilota* **252** *Cephaloleia teutonica* **253** *Cephaloleia thiemei* **254** *Cephaloleia triangularis* **255** *Cephaloleia trilineata* **256** *Cephaloleia trimaculata* **257** *Cephaloleia trivittata* **258** *Cephaloleia truncatipennis* **259** *Cephaloleia tucumana*. Scale bars equal 3 mm.

##### Diagnosis.

This species is similar to *Cephaloleia maculipennis*. It can be distinguished by the black elytra and by the densely punctate vertex of the head.

##### Host plant.

Adults have been collected feeding on *Calathea lanata* Peterson (Marantaceae).

##### Distribution.

Colombia, Ecuador, Peru.

##### Type material.

Type: Colombia (depository unknown, not examined).

##### Specimens examined.

**Colombia:** no further data (BMNH). **Ecuador:** Pichincha- Maquipuncuna Biological Reserve, 1350 m, 2–3 August 1998 (AJGC), 1275 m, Principal Trail, 27–29 October 1999 (SEMC). Sucumbios- Shushifindi, 1200 m, 9 August 1998 (AJGC, USNM). **Peru:** Loreto- Madreselva Biol. Stn., 24 June 2005 (USNM). Total: 22.

#### Cephaloleia teutonica

Taxon classificationAnimaliaColeopteraChrysomelidae

Uhmann, 1937

http://species-id.net/wiki/Cephaloleia_teutonica

Fig. 252

Cephalolia teutonica Uhmann 1937: 153.
Cephaloleia teutonica Uhmann. Uhmann 1957b: 26 (catalog), 1964b: 5 (faunal list); Gaedike and Döbler 1971: 361 (types); Staines 1997b: 414 (Uhmann species list).

##### Description.

Elongate; subparallel; subdepressed; shining; head, pronotum, scutellum, venter, and legs yellowish-brown; antennae and elytra black. Head: vertex sparsely punctate, medial sulcus present; keel present between antennal bases; eyes convex; frons sparsely punctate, not projecting; not depressed between eyes. Antenna: reaches to humerus; robust; antennomere 1 thick, elongate in male, shorter in female; 2 transverse, ⅓ length of 1; 3–4 transverse, subequal in length, each longer than 2; 5–10 transverse, subequal in length, each shorter than 3; 11 2× length of 10, pointed at apex; 1–2 punctate with scattered setae; 3–11 setose. Pronotum: transverse; lateral margin straight then rounding to anterior angle, weakly margined; anterior angle rounded, weakly produced; posterior angle acute; anterior margin weakly emarginate behind head; disc subconvex; surface irregularly punctate; basal impression absent; pronotal length 1.0–1.2 mm; pronotal width 1.3–1.5 mm. Scutellum: pentagonal; sparsely punctate. Elytron: lateral margin straight, smooth, broadly margined; apex rounded; sutural angle with small tooth; humerus rounded, not produced; slightly constricted behind humerus; declivity beginning just behind humerus at puncture row 7 edged with faint carina; moderately punctate-striate, punctures in row 8 larger, rows converge and unite apically; elytral length 3.5–3.9 mm; elytral width 1.8–2.0 mm. Venter: prosternum impunctate; meso- and metasterna impunctate medially, punctate laterally; abdominal sterna punctate, each puncture with pale seta; suture between sterna 1 and 2 complete; last sternite with apical margin rounded and emarginate medially in male, rounded in female. Leg: slender; punctate; tibia with fringe of setae on inner margin of apex; Total length: 5.0–5.5 mm.

##### Diagnosis.

This species is similar to *Cephaloleia abdominalis*, *Cephaloleia amazona*, *Cephaloleia princeps*, *Cephaloleia steinhauseni*, and *Cephaloleia susanae* sp. n. It can be distinguished by the straight lateral margins of the pronotum, by the vertex of the head having a medial sulcus, by antennomere 3 being longer than 2, and by the immaculate pronotum.

##### Distribution.

Argentina, Brazil (Minas Gerais, Río Grande do Sul, Santa Catharina, São Paulo).

##### Type material examined.

Holotype male: Brazil, S. Catharina, Nova Teutonia, Plaumann (DEI).

##### Specimens examined.

**Brazil:** Minas Gerais- Alagoa, Serra Branca, Murici, May 1964 (USNM). Santa Catharina- Corupa, October 1944, November 1944, December 1944 (AMNH); Nova Teutonia, 14 October 1938, 23 29 October 1942, September 1948, 4 December 1954 (USNM), 29 March 1938 (AMNH); Río Natal, September 1944 (AMNH), November 1976 (EGRC). São Paulo- São Paulo, 18 October 1940 (USNM); São Paulo Botanical Garden, 13 November 1971 (USNM); Cantareira, 29 October 1939 (USNM). Total: 16.

#### Cephaloleia thiemei

Taxon classificationAnimaliaColeopteraChrysomelidae

Weise, 1910

http://species-id.net/wiki/Cephaloleia_thiemei

Fig. 253

Cephaloleia thiemei Weise 1910: 92. Weise 1911a: 10 (catalog), 1911b: 12 (catalog); Uhmann 1957b: 26 (catalog).

##### Description.

Small; subelongate; subparallel; subconvex; slender; yellowish with darker markings; legs reddish-yellow; antennae black except antennomeres 1–3 or 1–4 reddish; head, pronotum, and scutellum black; elytra with darkened suture and transverse black macula apically. Head: vertex distinctly, finely punctate, faint medial sulcus present; frons not projecting; slightly depressed between eyes. Antenna: reaches to humerus; slender; antennomere 1 incrassate, elongate; 2 robust, ½ length of 1; 3–4 elongate, cylindrical, each longer than 2; 5–6 elongate, cylindrical, decreasing in length; 7–10 transverse, decreasing in length; 11 2× length of 10, pointed at apex; 1–3 punctate with scattered setae; 4–11 setose. Pronotum: quadrate; lateral margin straight then rounding to anterior angle, slightly canaliculate; anterior angle rounded, not produced; posterior angle acute; anterior margin curved anteriorly; disc subconvex; surface distinctly, irregularly punctate; basal impression absent; pronotal length 0.8–1.0 mm; pronotal width 0.9–1.1 mm. Scutellum: pentagonal; impunctate. Elytron: lateral margin straight, smooth, narrowly margined; apex broadly rounded; sutural angle without tooth; humerus rounded, not produced; slightly constricted behind humerus; shallowly punctate-striate, rows converge and unite apically; elytral length 2.6–2.9 mm; elytral width 1.3–1.5 mm. Venter: obscured by glue and card. Leg: slender; punctate; tibia with fringe of setae on inner margin of apex. Total length: 3.8–4.2 mm.

##### Diagnosis.

This species is similar to *Cephaloleia trimaculata*. It can be distinguished by the vertex of the head having a medial sulcus.

##### Distribution.

Amazonas, Ecuador.

##### Type material examined.

Syntypes: Amazonas (ZMHB, 2).

##### Specimens examined.

**Ecuador:** Napo- Yuturi Lodge, Río Napo, 270 m, 20–21 March 1999 (SEMC). Total: 2.

#### Cephaloleia triangularis

Taxon classificationAnimaliaColeopteraChrysomelidae

Staines, 1996

http://species-id.net/wiki/Cephaloleia_triangularis

Fig. 254

Cephaloleia triangularis Staines 1996: 65.

##### Description.

Elongate; subparallel; subdepressed; head (except yellow frons) antennae, and scutellum black; pronotum reddish or yellowish with black macula on anterior margin behind head which varies from semicircular to triangular in shape; elytra black with yellow longitudinal vitta, width of vitta varies from interspace 1 to interspace 7, not narrowing; venter with pro-, meso-, and metasterna yellow medially, black laterally, abdominal sterna 1 yellow medially, black laterally, 2–5 black medially, yellow laterally; legs with femur yellowish, tibia and tarsi black. Head: vertex punctate, medial carina present; frons not projecting; not depressed between eyes. Antenna: reaches to humerus; slender; antennomere 1 incrassate, clavate, elongate; 2–3 transverse, projection on inner apical angle; 2 ¼ length of 1, shortest; 3–4 subequal; 5–10 transverse, subequal in length; 11 rounded at apex, 2× length of 10; 1–3 punctate with scattered setae; 4–11 setose. Pronotum: quadrate; lateral margin straight then rounding to anterior angle, margined; anterior angle rounded, not produced; posterior angle acute; anterior margin straight; disc flattened; surface impunctate; basal impression absent; pronotal length 1.0–1.3 mm; pronotal width 1.3–1.7 mm. Scutellum: triangular; impunctate. Elytron: lateral margin straight, smooth, margined; apex rounded; sutural angle without tooth; humerus rounded, not produced; slightly constricted behind humerus; shallowly punctate-striate, rows obsolete at apex; elytral length 4.1–5.1 mm; elytral width 1.7–2.1 mm. Venter: pro-, meso-, and metasterna impunctate medially, punctate laterally; abdominal sterna punctate, each puncture with pale seta; suture between sterna 1 and 2 complete. Leg: robust, short; femur punctate; tibia with spoon-like depression at apex, tuft setae on inner margin. Total length: 5.7–7.1 mm.

##### Diagnosis.

This species is similar to *Cephaloleia belti*, *Cephaloleia consanguinea*, *Cephaloleia erugatus*, *Cephaloleia semivittata*, *Cephaloleia variabilis*, and *Cephaloleia vicina*. It can be distinguished by the elytral puncture rows being obsolete apically, by the impunctate pronotum, and by the pro-, meso-, and metasterna being punctate laterally.

##### Distribution.

Costa Rica.

##### Type material examined.

Holotype: Est. Hitoy Cerere, 100 m, R. Cerere, Res/ Biol. Hitoy Cerere, Prov. Limon, Costa Rica, E. Lopez, 28–12 abr 1992, L-N 184200, 643300/ COSTA RICA, INBIO, CR1000, 375730 [reversed white label with bar code]/ HOLOTYPE Cephaloleia triangularis Staines, Des. C. L. Staines 1994 [red label] (INBIO).

##### Specimens examined.

**COSTA RICA:** Cartago- Ref. Nac. Fauna Silv. Tapantí, 1650 m, Represa Río Gde. de Orosí, August 1991 (INBIO). Guanacaste- La Palma, 30 April 1928 (USNM); R. Sn. Lorenzo, 1050 m, Tierras Morenas, R. F. Cord., October 1991, November 1991 (INBIO). Limón- Est. Hitoy Cerere, 100 m, R. Cerere, Res. Biol. Hitoy Cerere, May 1991, July 1991, 4–20 December 1991, 5–19 March 1992, 30 June- 20 July 1992 (INBIO, USNM); Valle La Estrella, 100–200 m (INBIO). Puntarenas- Est. Sirena, Corcovado N. P., 0, 100 m, February 1990 (INBIO). Total: 22.

#### Cephaloleia trilineata

Taxon classificationAnimaliaColeopteraChrysomelidae

Uhmann, 1942b

http://species-id.net/wiki/Cephaloleia_trilineata

Fig. 255

Cephalolia trilineata Uhmann 1942b: 101.
Cephaloleia trilineata Uhmann. Uhmann 1957b: 26 (catalog); Gaedike and Döbler 1971: 361 (types); Staines 1997b: 414 (Uhmann species list).

##### Description.

Elongate; subparallel; subconvex; shining; yellowish-brown; antennomeres 1–2 reddish-brown, 3–11 black; head with vertex black; scutellum black; elytra with sutural vitta, vitta from humerus to near apex, and apex black. Head: vertex densely punctate medially, medial sulcus absent; frons not projecting; depressed between eyes. Antenna: reaches to humerus; slender; antennomeres elongate, nearly subequal in length; 1 shortest; 11 longest, rounded at apex; 1–2 punctate with scattered setae; 3–11 setose. Pronotum: transverse; lateral margin straight then rounding to anterior angle, margined; anterior angle obtuse, not produced; posterior angle acute; anterior margin curved anteriorly; disc subconvex; surface sparsely, irregularly punctate; basal impression absent; pronotal length 1.4–1.6 mm; pronotal width 1.7–1.9 mm. Scutellum: pentagonal; impunctate. Elytron: lateral margin straight, smooth, slightly laminate; apex rounded; sutural angle weakly emarginate, with small tooth; humerus rounded, not produced; slightly constricted behind humerus; shallowly punctate-striate, puncture rows 3–4 confused at base, rows converge and unite apically; pygidium punctate; elytral length 4.3–4.5 mm; elytral width 2.1–2.2 mm. Venter: pro-, meso-, and metasterna impunctate medially, punctate laterally; abdominal sterna sparsely punctate, each puncture with pale seta; suture between sterna 1 and 2 complete; last sternite with apical margin broadly emarginate medially in female. Leg: slender; punctate; tibia with fringe of setae on inner margin of apex. Total length: 6.0–6.2 mm.

##### Diagnosis.

This species is similar to *Cephaloleia abdita* sp. n. and *Cephaloleia gemma* sp. n. It can be distinguished by the elytral puncture rows 3 and 4 being confused basally, by the sutural angle of the elytra having a small tooth, and by antennomere 1 being shorter than 2.

##### Distribution.

Brazil (Río de Janiero).

##### Type material examined.

Holotype: Brazil, Est. do Río, Itatiaya, 705 m, 30.X.1933, Zikan (DEI).

##### Specimens examined.

**Brazil:** São Paulo- Río Piracicaba, February 1885 (USNM). Total: 1.

#### Cephaloleia trimaculata

Taxon classificationAnimaliaColeopteraChrysomelidae

Baly, 1858

http://species-id.net/wiki/Cephaloleia_trimaculata

Fig. 256

Cephaloleia trimaculata Baly 1858: 166. Uhmann 1936a: 117 (comparative note), 1950b: 324 (noted), 1956: 560 (noted), 1957a: 26 (catalog), 1964a: 404 (catalog); Blackwelder 1946: 720 (catalog); Papp 1953: 23 (catalog); Staines 1996: 66 (Central America species), 2004: 313 (host plants), 2011: 51 (faunal list); Staines and Staines 1999: 524 (Baly species list); McKenna and Farrell 2005: 121 (phylogeny), 2006: 10949 (phylogeny); Meskins et al. 2008: 163 (host plants), 2011: 483 (food web); García–Robledo et al. 2013a: 3 (biology).
Cephalolia trimaculata Baly. Gemminger and Harold 1876: 3602 (catalog); Donckier 1899: 551 (catalog); Weise 1911a: 10 (catalog), 1911b: 12 (catalog), 1913: 101 (noted); Bryant 1942: 205 (faunal list).
Cephalolia trimaculata columbica Weise 1913: 101 (type: Colombia, Río Negro; Sosomoco, ZMHB, not seen). Uhmann 1936a: 117 (noted).
Cephaloleia trimaculata columbica Weise. Uhmann 1956: 561 (noted).

##### Description.

Broadly elongate; subparallel; subdepressed; shining; head, pronotum, and scutellum black; antennomeres 1–2 black, 3–11 yellow; elytra yellow with small black humeral macula and black subovate sutural macula; venter with pro-, meso-, and metasterna yellowish medially, black laterally; abdomen yellow; legs yellow. Head: vertex deeply punctate, medial sulcus absent; frons not projecting; depressed between eyes. Antenna: reaches to humerus; slender; antennomere 1 slightly incrassate, elongate, longer than 3; 2 transverse, less than ½ length of 1; 3–4 elongate, 4 longer than 3; 5–10 transverse, subequal in length; 11 2× length of 10, pointed at apex; 1–3 punctate with scattered setae; 4–11 setose. Pronotum: subquadrate, much narrower than base of elytra; lateral margin straight then rounding to anterior angle, canaliculate; anterior angle rounded, slightly produced; posterior angle angulate; anterior margin weakly emarginate behind head; disc subconvex; surface covered with large, round, dark punctures, punctures more dense laterally; basal impression absent; pronotal length 0.9–1.0 mm; pronotal width 1.1–1.3 mm. Scutellum: pentagonal; impunctate. Elytron: lateral margin straight, smooth, margined; apex obtusely rounded; sutural angle without tooth; humerus rounded, not produced; slightly constricted behind humerus; slightly flattened along suture; moderately punctate-striate, rows confused apically; elytral length 3.4–3.6 mm; elytral width 1.7–2.0 mm. Venter: pro-, meso, and metasterna impunctate medially, punctate laterally; abdominal sterna punctate, each puncture with pale seta; suture between sterna 1 and 2 complete; male with last sternite with apical margin broadly truncate, female weakly emarginate medially. Leg: slender; punctate, each puncture with pale seta; tibia with setae on apical ½. Total length: 4.6–4.8 mm.

##### Diagnosis.

This species is similar to *Cephaloleia weisei*. It can be distinguished by antennomere 1 being longer than 2, by the pronotum being more punctate laterally, and by the elytral puncture rows being confused apically.

##### Host plant.

Adults have been collected on ginger lily (Zingiberaceae) (Uhmann 1950a); *Renealmia* sp. (Zingiberaceae) (McKenna and Farrell 2005); *Costus pulverulentus*, C. Presl. (Costaceae) (Meskins et al. 2008); *Renealmia pluriplicata* Maas (García–Robledo et al. 2013a).

##### Distribution.

Colombia, Costa Rica, Ecuador, French Guiana, Panama, Venezuela.

##### Type material examined.

Holotype female: Venezuela, Caracas, Sallé (BMNH).

##### Specimens examined.

**COSTA RICA:** Limón- Sector Cerro Cocorí, Fca. de E. Rojas, 150 m, May 1991 (INBIO). **Ecuador:** Napo- Sacha Lodge, 4–14 March 1994, 13–23 April 1994, 14–24 May 1994 (USNM). **French Guiana:** 33.5 km S Cayenne, 8.4 km NW Hwy N2 on hwy D5, 29 May- 7 June 1997 (SEMC); Roura, 27.4 km SSE, 10 June 1997 (SEMC); Saul, 7 km N, 1 km NW Les Eaux Claires, along Rue de Belizon trail, 4–8 June 1997 (SEMC). **PANAMA:** Panamá- Cerro Campana, 850 M, 31 July 1970 (CMNC). **Venezuela:** Caracas 14 September 1952 (USNM). Aragua- Pontachuelo, 14 August 1988 (BYUC); Portachelo Pass, 12 July 1987 (BYUC), 23–30 June 1967 (USNM), 4 June 1998 (SEMC); Rancho Grande, 14 May 1985 (BYUC), January 1954, 5 July 1968, 22–23 February 1971, 6 January 1973, 13 April 1975, 17 January 1976, 10 June 1983, 12 July 1987, 13 May 1998 (USNM), 26 January 1989 (AMNH), 12–14 May 1998, 13 May 1998 (SEMC). Lara- Parque Nac. Yacamba, El Blanquita, 1350 m, 1–3 August 1976, 6–8 April 1981 (USNM); 17.4 km SE Sanare, Yacambu National Park, 18 May- 1 June 1998 (SEMC). Merida- Andre Bello, 6 km E La Azulita, 15 July 1986 (BYUC). Total: 55.

#### Cephaloleia trivittata

Taxon classificationAnimaliaColeopteraChrysomelidae

Baly, 1885

http://species-id.net/wiki/Cephaloleia_trivittata

Fig. 257

Cephaloleia trivittata Baly 1885: 16. Blackwelder 1946: 720 (catalog); Papp 1953: 23 (catalog); Uhmann 1957a: 26 (catalog); Wilcox 1983: 138 (catalog); Staines 1996: 66 (Central America species), 1996(1997): 17 (Nicaragua species), 2004: 313 (host plants), 2011: 51 (faunal list); Staines and Staines 1997: 22 (types), 1999: 524 (Baly species list); Maes 1999: 1017 (faunal list); Flowers and Hanson 2003: 51 (distribution); García–Robledo et al. 2010: 64 (noted), 2013a: 3 (biology); Schmitt and Frank 2013: 58 (biology).
Cephalolia trivittata Baly. Donckier 1899: 551 (catalog); Weise 1904: 438 (noted), 1911a: 10 (catalog), 1911b: 12 (catalog); Uhmann 1930a: 227 (faunal list).

##### Description.

Small; elongate; subparallel; subdepressed; shining; head, antennae pronotum (except pale anterior margin), and scutellum black; elytra yellow with three wide black vittae- medial vitta along sutural margin, slightly dilated below scutellum, lateral vittae begin below humeral callus, extend entire length of disc, incurve to join sutural vitta, humerus reddish or yellowish; venter with pro-, meso-, metasterna, and abdominal sterna 1–4 yellow medially, dark laterally, sternite 5 totally black; leg yellow except dark apex of femur, base of tibia, and tarsi. Head: vertex densely punctate, medial carina present; frons not projecting; not depressed between eyes. Antenna: reaches to humerus; slender, elongate; antennomere 1 clavate, subequal in length to 3; 2 transverse; 3 elongate, cylindrical; 4–10 transverse, decreasing in length; 11 2× length of 10, pointed at apex; 1–2 punctate with scattered setae; 3–11 setose. Pronotum: subquadrate; lateral margin straight then rounding to anterior angle, margined; anterior angle subacute, produced; posterior angle acute; anterior margin straight; disc subconvex; surface deeply but moderately punctate; basal impression absent; pronotal length 0.7–0.9 mm; pronotal width 0.9–1.0 mm. Scutellum: pentagonal; alutaceous. Elytron: lateral margin straight, smooth, margined; apex rounded; sutural angle slightly sinuate, without tooth; humerus slightly angulate, slightly produced; constricted behind humerus; subconvex; slightly flattened along suture; strongly punctate-striate, rows obsolete at apex; elytral length 2.8–3.3 mm; elytral width 1.3–1.4 mm. Venter: pro-, meso-, and metasterna, abdominal sterna 1–4 impunctate medially, punctate laterally; abdominal sterna 1–4 impunctate medially, punctate laterally, each puncture with pale seta; sternite 5 punctate, each puncture with pale seta; suture between sterna 1–2 obsolete medially; last sternite with apical margin entire, rounded in female, broadly incurved in male. Leg: slender; femur punctate, each puncture with pale seta; tibia with fringe of setae on inner margin of apex. Total length: 4.1–4.4 mm.

##### Diagnosis.

This species is similar to *Cephaloleia belti*, *Cephaloleia consanguinea*, *Cephaloleia erugatus*, *Cephaloleia semivittata*, *Cephaloleia triangularis*, *Cephaloleia variabilis*, and *Cephaloleia vicina*. It can be distinguished by the elytral punctures being obsolete apically and by antennomere 3 being subequal in length to 1.

##### Comments.

Preliminary analysis of the CO1 gene indicates that cryptic species may be present under the current application of this species name. Further work is needed to resolve this question.

##### Host plant.

Adults have been collected on *Calathea haamelii* H. Kennedy, *Cephaloleia macrosepala* K. Schum. (Staines 1996); *Cephaloleia cleistantha* Standl., *Cephaloleia gymnocarpa* H. Kenn., *Cephaloleia lutea* Schult., *Cephaloleia venusta* H. Kenn., *Cephaloleia warscewiczii* Körn., *Pleiostachya pruinosa* (W. Bull. ex. Regel) K. Schum. (Marantaceae) (García–Robledo et al. 2013a); *Cephaloleia pulverulentus* C. Presl. (Schmitt & Frank 2103); *Cephaloleia marantifolia* Standl., *Ctenanthe* sp., *Donax canniformis* K. Schum, *Marantochloa purpurea* (Ridl.) Milne-Redh., *Stromante jacquinii* (Roem. and Schult.) H. Kenn. and Nicolson, *Ischnosiphon elegans* Standl., *Alpinia purpurata* K. Schum., *Hedychium coronarium* J. Koenig, (Zingiberaceae).

##### Distribution.

Costa Rica, Nicaragua, Panama.

##### Type material examined.

Syntypes: Bugaba, Panama, Champion/ Paratipo [red label]/ F. Monros Collection 1959/ Cephaloleia trivittata Baly, J. S. Baly det. [pink label], USNM, 1; V. de Chiriqui, 25–4000 ft., Champion (USNM, 1).

##### Specimens examined.

**COSTA RICA:** Alajuela- Res. For. San Ramón, 900 m, 9 March 1990 (INBIO); San Ramón, Río San Lorencito, 800 m, 3 July 1986 (INBIO); San Ramón EB, 27 km N and 8 km W San Ramón, 8 July 2000 (SEMC). Cartago- Aquiares nr. Santa Cruz, 9 km NW Turrialba, 1500 m, 16 May 1985 (EMEC); 1.2 mi SE Tuis, 18–21 April 1992 (CDFA); Turrialba, CATIE, 19–21 May 1979, 20 May 1979 (CMNC); Turrialba (USNM), 650 m, 4–13 August 1970 (USNM); ITCA at Turrialba, 13 March 1965 (BYUC); Turrialba, Santa Teresita, Monumento Nacional Guayabo, 1100–1200 m (INBIO). Guanacaste- Estación Mengo, 1100 m, SW side Volcán Cacao, February 1989 (INBIO); Río San Lorenzo, 1050 m, Tierras Morenas, Z. P. Tenorio, 10–20 February 1992, 23 March- 21 April 1992, April 1992 (INBIO); Volcán Cacao, 9 February 1989 (MUCR); Estación Pitilla, 9 km S Santa Cecilia, 600–700 m (INBIO). Heredia- La Selva Biol. Sta., 3 km S Pto. Viejo, 3 July 2001 (USNM); Fca. La Selva nr. Puerto Viejo, 7 August 1969 (USNM); El Plastico Station, 4 July 2011 (USNM); Rara Avis Biological Station, 13 November 2011 (USNM). Limón- Sector Cerro Cocorí, Fac. de E. Rojas, 150 m, August 1991, 31 January- 21 February 1992, 26 March- 24 April 1992, October 1992, 9–30 November 1992, February 1993, March 1993 (INBIO); Cerro Tortuguero, P. N. Tortuguero, 100 m, December 1989, December 1992 (INBIO); Estación Cuatro Esquinas, 0 m, P. N. Tortugero, September 1992 (INBIO); Guápiles, 30 October 1942 (MUCR); Hamburg Farm, Reventazón, Ebene Limón, 14 November 1922 (USNM), 25 January 1926 (DEI), 1 January 1933 (MUCR); Amubri, Sendero Soki, 0–100 m (INBIO). Puntarenas- Estación Biol. Las Alturas, 1500 m, Coto Brus, 23 March- 2 May 1992 (INBIO); Coto Brus, Las Cruces Biological Station, 5 March 2012, 6 March 2012, 10 March 2012 (USNM); Parque Nacional Corcovado, Estación Sirena, 20 March 1981 (INBIO), 20 February 1981 (MUCR); Fca. Las Cruces, San Vito de Java, 27 June 1969 (USNM); Monteverde Cloud For. Res., 1300 m, 17–20 May 1985 (EMEC); Monteverde FIT, 27 February 1983 (CMNC); Monteverde Cloud For. Res., 27–31 May 1984 (EGRC, USNM); Monteverde Reserve (trail near lab), 30 May 1993 (SEMC); Rancho Quemado, 200 m, Peninsula de Osa, September 1992 (INBIO); Osa Peninsula, 8.0 mi SW Rincón, 3 August 1968 (USNM); San Luis, 1040 m, R. B. Monteverde, October 1992 (INBIO); Peninsula Osa, 23 km N. O. La Palma, October-November 1990 (MUCR); 6 mi S San Vito, June 1969 (USNM); 22 mi SW San Vito, 11 August 1969 (USNM); Estación Sirena, 0–100 m, P. N. Corcovado, February 1990, August 1991, 21 March- 21 April 1992, 9–27 July 1992, December 1992 (INBIO); Sirena Station, Corcovado National Park, Río Pavo Trail, 25–28 June 2000 (SEMC); Augirre, Quepos, P.N. Manuel Antonio, 0–100 m (INBIO); Estación Altamira, 1 km S Cerro Biolley, 1400–1500 m (INBIO); Garabito, Reserva Biológica Carara, Estación Quebrada Boniita, 0–100 m (INBIO); Garabito, Tarcoles, Estación Quebrada Bonita, 100–200 m (INBIO); Estación Esquinas, Peninsula de Osa, 0–100 m (INBIO); Golfito, Jiménez, P.N. Corcovado, Estación Sirena, 0–100 m (INBIO); Guacimal, Finca Buen Amigo, Monteverde, 1000–1100 m (INBIO); Reserva Bosque Eterno de los Niños, Sector Monteverde, 1500–1600 m (INBIO); Estación La Casona, Las Torres, 1500–1600 m (INBIO); Monteverde, Estación Casona, 1500–1600 m (INBIO). San José- Estación Las Nubes de Santa Elena, 1200–1300 m (INBIO). **Nicaragua:** Atlantico Norte- Masawas, Waspuc River, 27 September 1955 (EMEC). **PANAMA:** Chiriquí- 11.2 km E Chiriquí, 30 May 1993 (AJGC); Santa Clara, 23–25 May 1980 (EGRC). Total: 129.

#### Cephaloleia truncatipennis

Taxon classificationAnimaliaColeopteraChrysomelidae

Baly, 1869

http://species-id.net/wiki/Cephaloleia_truncatipennis

Fig. 258

Cephaloleia truncatipennis Baly 1869: 371. Uhmann 1957b: 26 (catalog); Staines and Staines 1999: 524 (Baly species list).
Cephalolia truncatipennis Baly. Gemminger and Harold 1876: 3602 (catalog); Donckier 1899: 551 (catalog); Weise 1911a: 10 (catalog), 1911b: 11 (catalog).

##### Description.

Elongate; slightly expanded apically; subdepressed; reddish-brown; antennae (except basal 3 antennomeres) and eyes darker. Head: vertex impunctate, with strong medial carina; frons impunctate, not projecting; depressed between eyes. Antenna: as long as head and pronotum combined; slender; antennomere 1 robust, subclavate, longest; 2 transverse, ⅓ length of 1; 3 elongate, cylindrical, longer than 2; 4–10 elongate, cylindrical, subequal in length, each shorter than 3; 11 2× length of 10, pointed at apex; 1–3 punctate with scattered setae; 4–11 setose. Pronotum: transverse; lateral margin straight and converging for basal ¾ then rounding to anterior angle, canaliculate, slightly laminate; anterior angle rounded, slightly produced; posterior angle acute; anterior margin emarginate behind head; disc subconvex; surface sparsely, coarsely punctate, punctures more dense basally and laterally; transverse basal impression present medially; pronotal length 1.6–1.7 mm; pronotal width 2.0–2.1 mm. Scutellum: pentagonal; impunctate. Elytron: lateral margin slightly expanding apically, smooth, margined; apex subtruncate; sutural angle without tooth; humerus rounded, not produced; slightly constricted behind humerus; disc flattened; shallowly punctate-striate, punctures obsolete apically; elytral width 5.0–5.1 mm; elytral width 2.5–2.6 mm. Venter: prosternum punctate medially, impunctate laterally; meso- and metasterna impunctate; abdominal sterna punctate, each puncture with pale seta; suture between abdominal sterna 1 and 2 complete; last sternite with apical margin obtuse-truncate in female. Leg: slender; punctate, each puncture with pale seta; tibia with fringe of setae on inner margin of apex. Total length: 6.7–6.8 mm.

##### Diagnosis.

This species is similar to *Cephaloleia interstitialis*, *Cephaloleia striata*, *Cephaloleia subdepressa*, and *Cephaloleia unctula*. It can be distinguished by the transverse basal impression on the pronotum and by antennomere 1 being longer than 2 but shorter than 2 and 3 combined.

##### Distribution.

Amazonas, Brazil.

##### Type material examined.

Holotype: Upper Amazons (BMNH).

##### Specimens examined.

**Brazil:** ?- Capella (USNM). Total: 1.

#### Cephaloleia tucumana

Taxon classificationAnimaliaColeopteraChrysomelidae

Weise, 1904b

http://species-id.net/wiki/Cephaloleia_tucumana

Fig. 259

Cephalolia tucumana Weise 1904b: 439. Weise 1906: 221 (noted), 1911a: 10 (catalog), 1911b: 12 (catalog); Bruch 1915: 375 (faunal list), 1928: 202 (faunal list), 1937: 32 (biology); Bondar 1931b: 134 (noted), 1940a: 49 (noted); Uhmann 1936b: 116 (noted), 1938b: 364 (noted); Maulik 1937: 132 (host plants); Lima 1936: 325 (faunal list), 1955: 202 (faunal list); Bosq 1943: 140 (faunal list); Monrós and Viana 1947: 163 (Argentina species); Grandi 1951: 841 (metamorphosis); Bachmann and Cabrera 2010: 74 (types).
Cephaloleia tucumana Weise. Lima 1955: 202 (faunal list); Uhmann 1957b: 26 (catalog), 1961b: 6 (noted), 1964a: 404 (catalog), 1964b: 20 (faunal list); Cox 1996: 168 (pupa); Staines 2004a: 313 (host plants).
Cephaloleia saccharina Maulik 1929: 88 (Syntypes: Brazil, Bahia, Bondar, BMNH, 4). Bruch 1937: 37 (synonymy); Maulik 1937: 132 (host plants); Staines 2004a: 312 (host plants).
Cephalolia saccharina Maulik. Lima 1930: 68 (faunal list), 1936: 325 (faunal list); Uhmann 1930c: 35 (redescription); Bondar 1931b: 135 (biology); Guérin 1953: 97 (faunal list).

##### Description.

Elongate, subparallel, subconvex; narrow; shining; black, with the basal ⅓ of elytra and elytral margin reddish-yellow; pronotal margin weakly reddish; venter with prosternum medially and mesosternum basally brownish-yellow; leg with tarsi and apex of the protibiae dark chestnut, almost black; tibio-femoral joint reddish chestnut. Head: vertex finely, sparsely punctate, with faint medial carina; eyes not strongly convex; slight keel present between antennal bases; frons not projecting; slightly depressed between eyes. Antenna: less than head and pronotum combined; slender; antennomere 1–2 subglobose, thick; 2 ¾ length of 1; 3 cylindrical, elongate, as long as 1 and 2 combined, longest; 4–10 transverse, subequal in length; 11 2× length of 10, bluntly pointed at apex; 1–2 punctate with scattered setae; 3–11 setose. Pronotum: quadrate; lateral margin straight then rounding to anterior angle, narrowly margined; anterior angle rounded, not produced; posterior angle acute; anterior margin weakly emarginate behind head; disc subconvex; surface finely, densely punctate with some larger punctures laterally and basally and medial line impunctate; basal impression absent; pronotal length 1.2–1.6 mm; pronotal width 1.3–1.8 mm. Scutellum: pentagonal; impunctate. Elytron: lateral margin straight, smooth, margined; apex rounded, slightly serrate; sutural angle without tooth; humerus rounded, not produced; slightly constricted behind humerus; moderately punctate-striate, punctation more pronounced laterally, rows converge and unite apically; scutellar row long; elytral length 4.0–4.7 mm; elytral width: 1.8–2.2 mm. Venter: pro-, meso-, and metasterna punctate; abdominal sterna punctate, each puncture with pale seta; suture between sterna 1 and 2 complete; last sternite with apical margin broadly rounded in male, truncate in female. Leg: slender; punctate, each puncture with pale seta; tibia flattened, with fringe of setae on inner margin of apex. Total length: 5.0–6.5 mm.

##### Diagnosis.

This species is similar to *Cephaloleia humeralis*. It can be distinguished by antennomere 2 being subglobose and by the pronotum lacking a medial basal impression.

##### Host plant.

*Canna* sp. (Monrós and Viana 1947), *Cephaloleia denudata* Roscoe (Bondar 1931b); *Cephaloleia glauca* L. (Bruch 1937) (Cannaceae); *Saccharum officinarum* L. (Maulik 1929); *Panicum grumosum* Nees (Monrós and Viana 1947) (Poaceae).

##### Distribution.

Argentina, Brazil (Bahia, São Paulo), Colombia.

##### Type material examined.

Syntype: Rep. Argentina, Prov. Tucuman, September 1897, C. Bruch (ZMHB, 1).

##### Specimens examined.

**Argentina:** Buenos Aires- 3 August, March 1932, 11 November 1934, January 1936, 1935, April 1939, October 1939, April 1941, May 1941, October 1943, October 1963 (USNM). Tucuman- no further data (MACN), 1942 (USNM), 16 November 1952 (USNM). Total: 55.

#### Cephaloleia turrialbana

Taxon classificationAnimaliaColeopteraChrysomelidae

Uhmann, 1930a

http://species-id.net/wiki/Cephaloleia_turrialbana

Fig. 260

Cephalolia turrialbana Uhmann 1930a: 216.
Cephaloleia turrialbana Uhmann. Blackwelder 1946: 720 (catalog); Papp 1953: 23 (catalog); Uhmann 1957a: 27 (catalog); Wilcox 1983: 138 (catalog); Staines 1996: 67 (Central America species), 1997: 414 (Uhmann species list); Staines and Staines 1997: 22 (types).

##### Description.

Elongate; subparallel; subdepressed; shining; head, antennae, pronotum, and scutellum dark; elytra reddish-brown with apical 1/5 dark and pale lateral margins; venter reddish-brown medially, dark laterally; legs yellowish. Head: vertex densely punctate, medial sulcus absent; frons not projecting; slightly depressed between eyes. Antenna: reaches to humerus; slender; antennomere 1 clavate, elongate; 2 transverse, ½ length of 1; 3 elongate, subequal in length of 1; 4–10 transverse, subequal in length; 11 pointed at apex, subequal in length to 1 or 3; 1–2 punctate with scattered setae; 3–11 setose. Pronotum: quadrate; lateral margin straight then rounding to anterior angle, canaliculate apically; anterior angle rounded, produced; posterior angle acute; anterior margin weakly emarginate behind head; disc subconvex; slight depression laterally behind middle; surface densely irregularly punctate; basal impression absent; pronotal length 1.0 mm; pronotal width 1.3 mm. Scutellum: pentagonal; impunctate. Elytron: lateral margin nearly straight, smooth, margined; apex rounded; sutural angle without tooth; humerus rounded, slightly produced; slightly constricted behind humerus; flattened along suture; shallowly punctate-striate, rows converge and unite apically; elytral length 3.7 mm; elytral width 1.8 mm. Venter: pro-, meso-, and metasterna impunctate medially, punctate laterally; abdominal sterna punctate, each puncture with pale seta; suture between sterna 1 and 2 obsolete medially. Leg: slender; femur and tibia punctate, each puncture with pale seta; tibia fringed with setae at apex. Total length: 5.0 mm.

**Figures 260–268.**
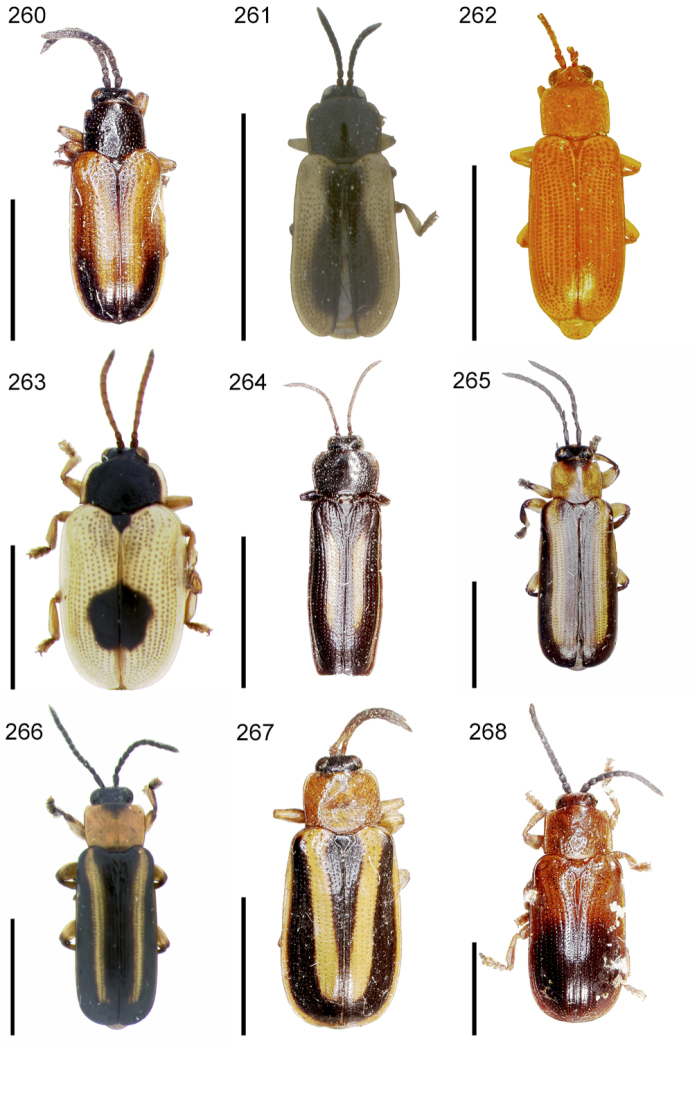
Habitus. **260** *Cephaloleia turrialbana* **261** *Cephaloleia uhmanni* **262** *Cephaloleia unctula* **263** *Cephaloleia uniguttata* **264** *Cephaloleia vagelineata* **265** *Cephaloleia variabilis* **266** *Cephaloleia vicina* **267** *Cephaloleia vittipennis* **268** *Cephaloleia waterhousei*. Scale bars equal 3 mm.

##### Diagnosis.

This species is similar to *Cephaloleia splendida*. It can be distinguished by antennomere 1 being twice as long as 2 and by the lateral impression on the pronotum.

##### Distribution.

Costa Rica, Panama.

##### Type material examines.

Holotype: Costa Rica, F. Nevermann [green label]/ Turrialba, 800 m, Slg. Schild [reversed green label]/ Type No. 54604 USNM [orange label]/ Holotype [red label]/ Det Uhmann/ Cephalolia turrialbana Uh. 30 (USNM).

##### Specimens examined.

**COSTA RICA:** Cartago- Turrialba, 500 m (USNM). **PANAMA:** ?- XX Plantation, 8 February 1930 (USNM). Total: 2.

#### Cephaloleia uhmanni

Taxon classificationAnimaliaColeopteraChrysomelidae

Staines, 1996

http://species-id.net/wiki/Cephaloleia_uhmanni

Fig. 261

Cephaloleia uhmanni Staines 1996: 67. Staines 1996(1997): 18 (Nicaragua species); Maes 1999: 1017 (faunal list); McKenna and Farrell 2005: 121 (phylogeny), 2006: 10949 (phylogeny).

##### Description.

Small; elongate; subparallel; subconvex; variable in coloration- head black; antennomeres 1–3 reddish-yellow, 4–11 black; pronotum black with paler margins; scutellum black; elytra black with reddish yellow at humerus and margins or brownish with dark suture; venter with pro-, meso-, and metasterna pale medially, dark laterally, abdominal sterna brownish-yellow; legs brownish-yellow. Head: vertex punctate, medial sulcus absent; frons not projecting; slightly depressed between eyes. Antenna: as long as head and pronotum combined; slender; antennomeres 1–2 transverse, thick, subequal in length; 3 elongate, cylindrical; 4–10 transverse, subequal in length, each ¾ length of 3; 11 2× length of 10, subequal in length to 3 pointed at apex; 1–2 punctate with scattered setae; 3–11 setose. Pronotum: transverse; lateral margin straight for basal ¾ then rounding to anterior angle, canaliculate; anterior angle rounded, not produced; posterior angle acute; anterior margin straight; disc subconvex; surface sparsely, deeply punctate, less dense and finer on disc and anterior margin; basal impression absent; pronotal length 0.6–0.7 mm; pronotal width 0.9–1.0 mm. Scutellum: pentagonal; impunctate. Elytron: lateral margin straight, smooth, margined; apex rounded; sutural angle without tooth; humerus rounded, not produced; constricted behind humerus; shallowly punctate-striate, humerus nearly impunctate, rows converge and unite apically; elytral length 2.4–3.0 mm; elytral width 1.1–1.3 mm. Venter: pro-, meso-, and metasterna impunctate medially, punctate laterally; abdominal sternite 1 punctate laterally, glabrous; 2–5 punctate, each puncture with pale seta; suture between sterna 1 and 2 obsolete medially; last sternite with apical margin emarginate medially in male, truncate in female. Leg: slender; punctate, each puncture with pale seta; femur robust; tibia with fringe of setae on inner margin of apex. Total length: 3.3–3.9 mm.

##### Diagnosis.

This species is similar to *Cephaloleia neglecta*. It can be distinguished by the smaller size and by the pronotum and elytra being the same color.

##### Distribution.

Costa Rica, Nicaragua, Panama.

##### Type material examined.

Holotype: Quepos, 80 m, P. N. Manuel Antonio, Prov. Punt., Costa Rica, G. Varela, Abr 1991, L-S-370900, 448800/ Costa Rica INBIO CR1000 484001/ Holotype Cephaloleia uhmanni Staines, des. C. L. Staines 1994 [red label] (INBIO).

##### Specimens examined.

**COSTA RICA:** Alajuela- 20 km S Upala, 11–15 May 1990, 1–5 October 1990, 11–20 April 1991, 10–21 May 1991, 22–31 May 1991, 11–20 August 1991 (BYUC). Guanacaste- 3 km SE R. Naranjo, 22–24 July 1992 (BYUC); Río San Lorenzo, 1050 m, Tierras Morenas, Z. P. Tenorio, 23 March- 21 April 1992 (INBIO); Estación Pitilla, 9 km S Santa Cecilia, 600–700 m (INBIO). Heredia- Las Cruces Field Station, 29 April 1975 (USNM); 11 km SE La Virgen, 450–550 m, 12 April 2003 (USNM). Limón- R.B. Hitoy Cerere, Sendero Toma de Agua, 0–100 m (INBIO); Amubri, Sendero Soki, 0–100 m (INBIO). Puntarenas- Estación Esquinas, 0 m, Peninsula de Osa, 8–27 November 1992 (INBIO); Golfito, 22 July 1981 (FSCA); Manuel Antonio, NB, 26 August 1986 (USNM); Quepos, P. N. Manuel Antonio, 80 m, March 1991, July 1991, October 1991, November 1991, April 1992, November 1992 (INBIO); Estación Queb., Bonita, 50 m, Res. Biol. Carara, 10–28 August 1992 February 1994 (INBIO); Estación Quebrada, R. B. Carara, 50 m, February 1994 (INBIO); Rancho Quemado, 200 m, Peninsula de Osa, April 1992 (INBIO); Sirena, Corcovado N. P., April 1989, October 1989 (INBIO); Golfito, Jiménez, Albergue Cerro de Oro, 100–200 m (INBIO); 3 km SW Rincón, 20 m, March-May 1991 (USNM). San José- Estación Bijagual, 500 m, Reserva Biológica Carara, November 1989, December 1989, January 1990, June 1993 (INBIO); La Caja, February 1929 (USNM). **NICARAGUA:** El Recrea, Zelaya, 29 November 1958 (USNM). **PANAMA:** Bocas del Toro- 6 km N Punta Peñas, 27 May 1993 (AJGC). Chiriquí- Reserva Fortuna, Continential Divide Trail, 30 May 1994 (CDFA); Santa Clara, 23–25 May 1980 (EGRC). Coclé- rd. W. Cerro Gaital, 15 May 1980 (EGRC). Colón- Pipeline Road, 10 mi NE Gamboa. 9 June 1978 (USNM). Total: 75.

#### Cephaloleia unctula

Taxon classificationAnimaliaColeopteraChrysomelidae

Pic, 1923

http://species-id.net/wiki/Cephaloleia_unctula

Fig. 262

Cephalolia unctula Pic 1923: 8. Uhmann 1938a: 409 (noted).
Cephaloleia unctula Pic. Uhmann 1957b: 27 (catalog), 1961b: 24 (noted); Descarpentries and Villiers 1959a: 139 (types); McKenna and Farrell 2005: 121 (phylogeny).

##### Description.

Small; elongate; subparallel; subconvex; reddish-brown; eyes darker. Head: vertex punctate, with medial carina; frons impunctate, not projecting; slightly depressed between eyes. Antenna: reaches to humerus; slender; antennomere 1 elongate, longer than 2 and 3 combined, slightly incrassate, apical margin produced with horn-like projection; 2–6 elongate, cylindrical; 2 longer than 3; 3 longer than 4; 4–6 elongate, subequal in length; 7–10 transverse, subequal in length, each shorter than 6; 11 pointed at apex; 1–6 punctate with scattered setae; 7–11 setose. Pronotum: transverse; lateral margin sinuate for basal ¾ then rounding to anterior angle, canaliculate; anterior angle rounded, not produced; posterior angle acute; anterior margin straight; disc flattened; surface densely, coarsely punctate; basal impression absent; pronotal length 1.0–1.2 mm; pronotal width 1.2–1.4 mm. Scutellum: triangular, impunctate. Elytron: lateral margin straight, smooth, narrowly margined; apex rounded; sutural angle without tooth; humerus rounded, not produced; slightly constricted behind humerus; shallowly punctate-striate, punctures large, slightly confused apically; elytral length 3.4–3.7 mm; elytral width 1.5–1.7 mm. Venter: pro-, meso-, and metasterna impunctate; abdominal sterna impunctate, glabrous; suture between sterna 1 and 2 complete. Leg: slender; impunctate; tibia with fringe of setae on inner margin of apex. Total length: 4.5–5.0 mm.

##### Diagnosis.

This species is similar to *Cephaloleia interstitialis*, *Cephaloleia striata*, *Cephaloleia subdepressa*, and *Cephaloleia truncatipennis*. It can be distinguished by the lack of a transverse basal impression on the prontum and by antennomere 1 being longer than 2 and 3 combined.

##### Distribution.

Colombia, Bolivia, Ecuador, Peru.

##### Type material.

Types: Ecuador (MNHN, NHRS, not seen).

##### Specimens examined.

**Bolivia:** Cochabamba- Cochabama, 117 km E Yungas, 1040 m, 1–6 February 1999 (SEMC). **Ecuador:** no further data (MNHN, NHRS). **Peru:** Lima- Lima, 2000–3000 ft. (USNM). Total: 4.

#### Cephaloleia uniguttata

Taxon classificationAnimaliaColeopteraChrysomelidae

Pic, 1923

http://species-id.net/wiki/Cephaloleia_uniguttata

Fig. 263

Cephalolia uniguttata Pic 1923: 9. Uhmann 1953d: 48 (faunal list).
Cephaloleia uniguttata Pic. Uhmann 1957b: 27 (catalog); Descarpentries and Villiers 1959a: 139 (types).

##### Description.

Suboval; subconvex; black; pronotum red laterally; elytra reddish-yellow with large rounded black sutural macula after middle; legs and abdomen red. Head: vertex sparsely punctate, medial sulcus absent; frons not projecting; slightly depressed between eyes. Antenna: reaches to humerus; slender; 1 subincrassate, elongate; 2 transverse, ½ length of 1; 3 elongate, cylindrical, 1½x of 2; 4–5 elongate, cylindrical, subequal in length, each ¾ length of 3; 5–10 transverse, subequal in length, each ¾ length of 5; 11 2× length of 10, rounded at apex; 1–2 punctate with scattered setae; 3–11 setose. Pronotum: transverse; lateral margin straight for basal ½ then rounding to anterior angle, broadly margined; anterior angle rounded, slightly produced; posterior angle acute; anterior margin weakly emarginate behind head; disc subconvex; surface irregularly, strongly punctate; transverse basal impression present medially; pronotal length 1.2 mm; pronotal width 1.6 mm. Scutellum: pentagonal; impunctate. Elytron: lateral margin straight for basal ⅔ then narrowing, smooth, margined; apex rounded; sutural angle with minute tooth; humerus rounded, not produced; slightly constricted behind humerus; moderately punctate-striate, punctures reduced at humerus, rows converge and unite apically; elytral length 3.6 mm; elytral width 2.4 mm. Venter: pro-, meso-, and metasterna punctate; abdominal sterna punctate, each puncture with pale seta; suture between sterna 1 and 2 complete; last sternite with apical margin emarginate medially in male, truncate in female. Leg: slender; punctate, each puncture with pale seta; tibia with fringe of setae on inner margin of apex. Total length: 5.0 mm.

##### Diagnosis.

This species is similar to *Cephaloleia insidiosa*. It can be distinguished by the pronotum having reddish lateral margins and by the elytra having an ovoid black medial macula on the apical ½.

##### Distribution.

Ecuador, Peru.

##### Type material examined.

Holotype: Pérou [handwritten label]/ Type [white label with red printing]/ Cephalolia uniguttata m., type [handwritten label]/ uniguttata Pic (1923) [handwritten label]/ Museum Paris Coll. M. Pic [printed blue label]/ Type [printed red label]/ Cephaloleia uniguttata Pic [printed label]/ Holotype [red printed label]/ MNHN EC 2651 [printed label] (MNHN).

##### Specimens examined.

**Ecuador:** Napo- Sacha Lodge, 270 m, 24 March- 5 April 1994, 14–24 May 1994, 13–23 June 1994, 25 July- 3 August 1994, 10–21 October 1994 (SEMC). Orellana- Estación Cientifica Yasuni, 5–10 September 1999 (EGRC). **Peru:** Loreto- 80 km NE Iquitos, Explorama Lodge, 1 km up Río Yanamono from Amazon River, at light, 1–5 September 1992 (EGRC). Total: 10.

#### Cephaloleia vagelineata

Taxon classificationAnimaliaColeopteraChrysomelidae

Pic, 1926c

http://species-id.net/wiki/Cephaloleia_vagelineata

Fig. 264

Cephalolia vagelineata Pic 1926c: 10.
Cephaloleia vagelineata Pic. Uhmann 1957b: 27 (catalog), 1964a: 404 (catalog); Descarpentries and Villiers 1959a: 139 (types); Genty et al. 1978: 332 (biology); Angel 1989: 81 (museum list); Couturier and Kahn 1992: 720 (host plants); Mariau 1999: 233 (noted), 2001: 132 (noted); Howard and Abad 2001: 100 (host plants).
Cephaloleia near *vagelineata* Pic. Sandino 1972: 77 (biology); Sandino 1972: 75 (biology), 1974: 21 (control), 1975: 20 (biology).

##### Description.

Small; narrow; elongate; depressed; shining; black; pronotum margined in red; elytra with broad Y-shaped yellow vitta on disc and lateral margins red. Head: vertex densely punctate, medial sulcus present; frons not projecting; depressed between eyes. Antenna: reaches to humerus; slender; antennomeres 1–2 thicker than others; 1 elongate, cylindrical; 2 transverse, shortest; 3–5 elongate, cylindrical; 3 2× length of 2; 4 slightly shorter than 3; 5 ¾ length of 4; 6–10 transverse, decreasing in length; 11 2× length of 10, acutely pointed at apex; 1–2 punctate with scattered setae; 3–11 setose. Pronotum: transverse; lateral margin straight and divergent for basal ¾ then rounding to anterior angle, margined, serrulate; anterior angle angulate, not produced; posterior angle acute; anterior margin straight; disc subconvex; surface densely coarsely punctate laterally, finely punctate medially; basal impression absent; pronotal length 0.9–1.0 mm; pronotal width 1.2–1.3 mm. Scutellum: pentagonal; punctate. Elytron: lateral margin straight, smooth, narrowly margined; apex strongly truncate; sutural angle without tooth; humerus rounded, not produced; constricted behind humerus; strongly punctate-striate, rows converge and unite apically; interspace 5 slightly carinate behind humerus, flattening posteriorly; elytral length 3.5–3.6 mm; elytral width 1.3–1.4 mm. Venter: pro-, meso-, and metasterna punctate; abdominal sterna sparsely punctate, each puncture with pale seta; suture between sterna 1 and 2 complete; last sternite with apical margin broadly concave in male. Leg: slender; punctate; tibia with fringe of setae on inner margin of apex. Total length: 4.9–5.0 mm.

##### Diagnosis.

This species is similar to *Cephaloleia formosus* and *Cephaloleia gracilis*. It can be distinguished by the serrulate lateral margins of the pronotum, by the angulate anterior angles of the pronotum, and by the suture between abdominal sterna 1 and 2 being complete.

##### Host plant.

*Elaeis guineensis* Jacq., *Corozo oleifera* (H.B.K.) Bailey, *Cocos nucifera* L. (Sandino 1972); *Astrocaryum chonta* Matrius (Couturier and Kahn 1992) (Arecaceae).

##### Biology.

The recently hatched larva feeds on the interior surface of the basal part of the young rachis of the palm. This damage has the appearance of superficial scrapings in the form of longitudinally lengthened maculae. These maculae are initially light (or clear) in color and darken with time. The adult beetles seek out the youngest leaves of the host palm, rasping the surface of the leaflets. This class of damage, which commonly is in the form of longitudinal bands or lines, can cause a yellowing and drying of the foliage, which can be afterwards be invaded by fungi (Sandino 1972).

The eggs are deposited individually in the internal surface of the base of the young rachis of the host palm. The larva prefers this humid medium that is protected from the direct action of the sun. Pupae are found in the internal basal part of the rachises. The adults are found in the youngest leaves of the palm and which are beginning to open. There they stay hidden among the leaflets. It is very rare to encounter the insect in old leaves or those that have already opened completely; possibly this is owing to their preference for humidity and protection from the effects of the sun, which are present on the leaves which they barely are beginning to separate their leaflet. The mechanical damage caused by the adults of *Cephaloleia vagelineata* although apparently insignificant, is important when the insects are present in abundance, in those cases, the affected areas can coalesce resulting in the withering of much of the foliage (Sandino 1972).

The eggs are yellow, flattened, ellipsoid and about 2 mm long. The larvae are whitish, flattened, and ellipsoid, with the last instar nearly 6 mm long. The pupa is similar in form and size to the larva and is distinguished by being thicker and having a cream color, which changes to black, in its central part, as it matures (Sandino 1972).

##### Distribution.

Brazil (Goiás, Matto Grosso), Colombia.

##### Type material.

Type: Brazil, Goyaz, Jatahy (MNHN, not seen).

##### Specimens examined.

**Brazil:** Goiás- Jatahy (MNHN). Matto Grosso- no further data (USNM). Total: 2.

#### Cephaloleia variabilis

Taxon classificationAnimaliaColeopteraChrysomelidae

Staines, 1996

http://species-id.net/wiki/Cephaloleia_variabilis

Fig. 265

Cephaloleia variabilis Staines 1996: 69.

##### Description.

Elongate; subparallel; subdepressed; head (except yellow frons) and scutellum black; antennae black or antennomeres 9–11 yellow; pronotum yellowish with black maculae on anterior and basal margins connected by narrow black vitta; elytra brown or black with yellow longitudinal vitta from base to apical ¼ from puncture rows 4 to 8, lateral margin black or yellow; venter with prosternum yellow, meso- and metasterna yellow medially, black laterally, abdominal sterna 1–4 black with yellow laterally, 5 totally black; leg with tibia and tarsi dark; femur yellow, dark at apex; coxae dark. Head: vertex punctate near eyes, center impunctate, medial sulcus absent; frons not projecting; not depressed between eyes. Antenna: reaches beyond humerus; slender; antennomere 1 elongate, as long as 2–4 combined, clavate, compressed laterally; 2 transverse, ½ length of 1; 3 transverse, longer than 2, projection on inner apical angle; 4–10 transverse; 4 shorter than 3; 5 subequal in length to 3; 6–10 subequal in length, each shorter than 5; 11 as long as 3 or 5, rounded at apex; 1–2 punctate with scattered setae; 3–11 setose. Pronotum: quadrate; lateral margin straight, slightly divergent then rounding to anterior angle, margined; anterior angle rounded, not produced; posterior angle acute; anterior margin straight; disc subconvex; surface sparsely punctate; basal impression absent; pronotal length 1.1–1.3 mm; pronotal width 1.4–1.6 mm. Scutellum: triangular; impunctate. Elytron: lateral margin straight, smooth, margined; apex rounded; sutural angle without tooth; humerus rounded, not produced; slightly constricted behind humerus; moderately punctate-striate, rows converge and unite apically; elytral length 4.9–5.6 mm; elytral width 2.0–2.3 mm. Venter: pro-, meso-, and metasterna impunctate medially, punctate laterally; abdominal sterna punctate, each puncture with pale seta; suture between sterna 1 and 2 complete; last sternite with apical margin sinuate in male, truncate in female. Leg: slender; impunctate; tibia with fringe of setae on inner margin of apex. Total length: 6.3–7.1 mm.

##### Diagnosis.

This species is similar to *Cephaloleia belti*, *Cephaloleia consanguinea*, *Cephaloleia erugatus*, *Cephaloleia semivittata*, *Cephaloleia triangularis*, *Cephaloleia trivittata*, and *Cephaloleia vicina*. It can be distinguished by elytral puncture rows being distinct apically, by antennomere 1 being as long as 2 to 4 combined, by the elytral puncture rows converging and uniting apically, and by the sutural angle of the elytra without a small tooth.

##### Distribution.

Colombia, Panama.

##### Type material examined.

Holotype: Colombia, Puerto Berrio Antio, Ag. 8, '38, H. Dybas/ Holotype Cephaloleia variabilis Staines, Des. C. L. Staines 1994 [red label] (USNM).

##### Specimens examined.

**COLOMBIA:** Meta- 3 mi. W. Villavicencio, 920 m, 11 March 1955 (CASC). Sartander- Puerto Berrío, Antio, 15 August 1938 (FMNH), 8 August 1938, 9 August 1938, 11 August 1938 (USNM). **PANAMA:** Panamá- Isthmus Matachin, O. Thieme S (ZMHB); Old Gamboa Road, 25 June 1994 (CDFA); El Llano-Carti rd. nr. Jct. main hwy, 18 May 1993 (EGRC). San Blas- Nusagandi area, I. K. U. S. A. Igar, 20 May 1993 (EGRC). Total: 47.

#### Cephaloleia vicina

Taxon classificationAnimaliaColeopteraChrysomelidae

Baly, 1858

http://species-id.net/wiki/Cephaloleia_vicina

Fig. 266

Cephaloleia vicina Baly 1858: 55. Baly 1885: 24 (noted); Blackwelder 1946: 720 (catalog); Papp 1953: 23 (catalog); Uhmann 1957a: 26 (catalog); Wilcox 1983: 138 (catalog); Strong 1977a: 165 (host plants), 1977b: 578 (host plants), 1981: 185 (host plants), 1982b: 1045 (host plants), 1983: 711 (host plants); Staines 1996: 70 (Central America species), 1999: 241 (mimicry), 2004: 313 (host plants), 2011: 51 (faunal list); Staines and Staines 1999: 524 (Baly species list).
Cephalolia vicina Baly. Gemminger and Harold 1876: 3602 (catalog); Donckier 1899: 551 (catalog); Weise 1905a: 131 (noted), 1910: 87 (noted), 1911a: 10 (catalog), 1911b: 10 (catalog); Uhmann 1936b: 484 (key).

##### Description.

Elongate; subparallel; depressed; head (except yellow frons), antennae, and scutellum black; pronotum yellow with triangular black macula behind head on anterior margin; elytra black with narrow yellow vittae which becomes obsolete on apical 1/5; venter with prosternum yellow, meso- and metasterna yellow medially, black laterally, abdominal sternite 1 entirely black, 2–5 yellow; legs with femur yellow with apex darker, coxae, tibiae, and tarsi darker. Head: vertex punctate, faint medial sulcus present; eyes convex, finely faceted; frons longitudinally strigose-punctate, not projecting; slightly depressed between eyes. Antenna: reaches to humerus; slender; antennomere 1 incrassate, elongate, compressed; 2 subglobose, short; 3 elongate, 2× length of 2, triangular with projecting angle; 4–10 transverse; 4 ¾ length of 3; 5 shorter than 4; 6–10 subequal in length, each shorter than 5; 11 more than 2× length of 2, pointed at apex; 1–2 punctate with scattered setae; 3–5 sparsely setose; 6–11 setose. Pronotum: quadrate; lateral margin straight then rounding to anterior angle, margined; anterior angle rounded, slightly produced; posterior angle acute; anterior margin emarginate behind head; disc flattened; surface with scattered large punctures laterally, impunctate medially; basal impression absent; pronotal length 1.15–1.27 mm; pronotal width 1.38–1.81 mm. Scutellum: elongate, acutely triangular; micropunctate. Elytron: lateral margin straight, smooth, narrowly margined; apex obtusely rounded; sutural angle without tooth; humerus rounded, not produced; constricted behind humerus; declivity beginning just behind humerus at puncture row 7 without faint carina; shallowly punctate-striate, rows converge and unite apically; elytral length 4.6–5.1 mm; elytral width 2.0–2.3 mm. Venter: prosternum impunctate; meso- and metasterna impunctate medially, punctate laterally; abdominal sterna punctate, each puncture with pale seta; suture between sterna 1 and 2 complete; last sternite with apical margin truncate in male, broadly concave in female. Leg: slender; punctate; tibia with fringe of setae on inner margin of apex. Total length: 5.9–6.7 mm.

##### Diagnosis.

This species is similar to *Cephaloleia bella*, *Cephaloleia championi*, and *Cephaloleia luctuosa*. It can be distinguished by the vertex of the head being depressed between the eyes, by the pronotum being punctate laterally, and by antennomere 2 being triangular.

##### Host plant.

*Heliconia* spp. (Strong 1977a), *Heliconia latispatha* Benth. (Strong 1977b), *Heliconia imbricata* (Kuntze) Baker (Heliconiaceae) (Strong 1981). Adults feed on. *Heliconia* spp., *Calathea* spp., *Ischnosiphon* spp. (Marantaceae) (Strong 1977a); *Heliconia psittacurum* Sassy.

##### Distribution.

Costa Rica, Guatemala, Mexico, Nicaragua, Panama.

##### Type material examined.

Lectotype female: Type/ female/ Cordova/ Mexico, Salle coll./ Cephaloleia vicina Fig. I/ Cephaloleia vicina Baly Salle coll. 1345/ B. C. A., Col. VI, 2. Cephaloleia vicina Baly/ sp. figured/ Cephaloleia vicina Baly, Mexico/ Lectotype Cephaloleia vicina Baly, Des. C. L. Staines 1993 [red label] (BMNH).

##### Specimens examined.

?- Vermachtnis, 1907 (DEI); Viessa de Zargolica (DEI). **COSTA RICA:** Alajuela- Colonia Dos Ríos, 1 November 1987 (MUCR); 2 Ríos, 1 December 1987 (MUCR); Res. For. San Ramón, 8 March 1990 (MUCR). Heredia- Estación Biol. La Selva, 22 January 1989 (USNM), 21 January 1989, 1 April 1990 (MUCR). Limón- Estación Hitoy Cerere, R. Cerere, Reserva Biológica Hitoy Cerere, 4–20 December 1991, 5–19 March 1992 (INBIO). **GUATEMALA:** Baja Verapaz- 6–9 km E Purulhá, +500', 15–24 April 1990 (FSCA). **MEXICO:** no further data (DEI). Guerrero- Ixcuinatoyac, 10 September 1943 (USNM). Jalisco- “Sierra Autlan”, 20 mi SSE Autlan, 5500’, 1 March 1953 (UMMZ). Tabasco- Cardenas, 15 October 2013 (USNM). Veracruz- Atoyac, 14 July 1941 (FMNH); Comoapan-Eyipantla, 14–27 June 1985 (EGRC); Fortín de las Flores- Sumidero, 21 May 1965 (FSCA); Motzorongo (DEI); 7 mi. S. E. Orizaba, 19–20 June 1983 (FSCA); Playa Vicente (DEI); Tezonapa, 8 August 1941 (FMNH). **NICARAGUA:** Lago- Chontales (USNM). **PANAMA:** Chiriquí- Fortuna, 20 May 1978 (USNM). Panamá- Barro Colo Isl, 3 March 1959 (FSCA). Total: 39.

#### Cephaloleia vittata

Taxon classificationAnimaliaColeopteraChrysomelidae

Staines, 1996

http://species-id.net/wiki/Cephaloleia_vittata

Image not available

Cephaloleia vittata Staines 1996: 70.

##### Description.

Small; elongate; subparallel; head, antennae, pronotum, and elytra whitish-yellow with black sutural vitta at base which reaches puncture row 2 then narrows gradually posteriorly until only suture darkened at apex; venter with yellow medially, dark laterally; legs whitish-yellow with coxae, apex of femur, base of tibia and, tarsi dark. Head: vertex densely punctate, medial sulcus absent; frons not projecting; not depressed between eyes. Antenna: reaches to humerus; slender; antennomeres 1–3 elongate, cylindrical; 1–2 subequal in length, 3 as long as 1–2 combined; 4–5 elongate, cylindrical, subequal in length; 6–10 transverse, subequal in length; 11 elongate, rounded at apex. Pronotum: transverse; lateral margin straight then rounding to anterior angle, canaliculate; anterior angle rounded, not produced; posterior angle acute; anterior margin curved anteriorly; disc subconvex; surface irregularly punctate; basal impression absent; pronotal length 0.9 mm; pronotal width 1.0 mm. Scutellum: pentagonal, impunctate. Elytron: lateral margin straight, smooth, margined; apex rounded; sutural angle without tooth; humerus rounded, not produced; slightly constricted behind humerus; moderately punctate-striate, rows converge and unite apically; elytral length 3.3 mm; elytral width 1.4 mm. Venter: pro-, meso-, metasterna, and abdominal sterna 1 and 2 punctate laterally; abdominal sterna 3–5 punctate, each puncture with pale seta; suture between sterna 1 and 2 obsolete medially. Leg: slender. Total length: 4.3 mm.

##### Diagnosis.

This species is similar to *Cephaloleia belti*, *Cephaloleia consanguinea*, *Cephaloleia erugatus*, *Cephaloleia semivittata*, *Cephaloleia triangularis*, *Cephaloleia trivittata*, and *Cephaloleia variabilis*. It can be distinguished by the elytral puncture rows being distinct apically, by antennomere 1 being subequal in length to 2 and shorter than 3, and by antennomere 2 being elongate.

##### Distribution.

Costa Rica.

##### Comments.

The holoype is apparently lost since it could not be located in the CMNC collection.

##### Type material.

Holotype: Costa Rica: Punt. S. Vito, Las Cruces, July 1982, B. Gill, 1200 m/ Flt. Intercept/ Holotype Cephaloleia vittata Staines, Des. C. L. Staines 1994 [red label] (CMNC, not seen).

#### Cephaloleia vittipennis

Taxon classificationAnimaliaColeopteraChrysomelidae

Weise, 1910

http://species-id.net/wiki/Cephaloleia_vittipennis

Fig. 267

Cephalolia vittipennis Weise 1910: 89. Weise 1911a: 10 (catalog), 1911b: 12 (catalog); Uhmann 1936b: 115 (lectotype); Guérin 1953: 97 (faunal list).
Cephaloleia vittipennis Weise. Uhmann 1957b: 27 (catalog), 1964a: 303 (catalog), 1964b: 5 (faunal list); Gaedike and Döbler 1971: 363 (type).

##### Description.

Elongate; subparallel; subconvex; reddish-yellow; antennomere 1 yellowish, 2–3 reddish-brown, 4–11 darker; head and scutellum black; elytra reddish-yellow with dark sutural vitta for basal ½ and wide black vitta from humerus curving to suture near apex, lateral and apical margins reddish-yellow; venter and legs reddish-yellow. Head: vertex finely punctate, medial sulcus present; frons not projecting; not depressed between eyes. Antenna: reaches to humerus; slender; antennomeres 1–5 elongate, cylindrical; 2 ¾ length of 1; 3 subequal to 1; 4–5 subequal, each ¾ length of 3; 6–10 transverse, subequal in length; 11 2× length of 10, pointed at apex; 1–4 punctate with scattered setae; 5–11 setose. Pronotum: transverse; lateral margin straight then rounding to anterior angle, smooth, narrowly margined; anterior angle rounded, not produced; posterior angle acute; anterior margin curved posteriorly; disc subconvex; surface distinctly punctate with impunctate medial longitudinal line; transverse impression present behind middle; pronotal length 1.3–1.5 mm; pronotal width 1.5–1.7 mm. Scutellum: pentagonal; impunctate. Elytron: lateral margin straight, smooth; narrowly margined; apex rounded; sutural angle without tooth; humerus rounded, not produced; constricted behind humerus; declivity beginning just behind humerus at puncture row 7 edged with faint carina; punctures small, fine basally, larger apically, rows converge and unite apically; scutellar row long; elytral length 4.1–4.8 mm; elytral width 2.0–2.4 mm. Venter: pro-, meso-, and metasterna impunctate medially, punctate laterally; abdominal sterna punctate, each puncture with pale seta; suture between sterna 1 and 2 complete; last sternite with apical margin rounded in male, truncate in female. Leg: slender; punctate, each puncture with pale seta; tibia with fringe of setae on inner margin of apex. Total length: 5.5–6.5 mm.

##### Diagnosis.

This species is similar to *Cephaloleia picta*. It can be distinguished by the vertex of the head with a medial sulcus and by the basal impression on the pronotum.

##### Distribution.

Argentina, Brazil (Santa Catharina, São Paulo), Peru, Venezuela.

##### Type material examined.

Syntype: [Brazil], Santos, 17–23.II.1899 (DEI, 1).

##### Specimens examined.

**Argentina:** Buenos Aires (USNM). **Brazil:** Santa Catharina- Hansa, 1934 (USNM). São Paulo- Cantareira, November 1939 (USNM); Santos, 17–25 February 1899 (ZMHB). **Peru:** Junin- Satipo, March 1945 (USNM). **Venezuela:** Aragua- Rancho Grande, 11 June 1983 (BYUC), June 1963 (USNM),12–14 May 1998 (SEMC). Total: 23.

#### Cephaloleia waterhousei

Taxon classificationAnimaliaColeopteraChrysomelidae

Baly, 1858

http://species-id.net/wiki/Cephaloleia_waterhousei

Fig. 268

Cephaloleia waterhousei Baly 1858: 48. Baly 1858: 166 (noted); Uhmann 1948b: 14 (noted), 1957b: 27 (catalog); Staines and Staines 1999: 524 (Baly species list).
Cephalolia waterhousei Baly. Gemminger and Harold 1876: 3602 (catalog); Donckier 1899: 551 (catalog); Weise 1911a: 10 (catalog), 1911b: 11 (catalog); Uhmann 1935b: 47 (faunal list).

##### Description.

Elongate; subparallel; subdepressed; shining; dark yellowish, antennae (except apex of antennomere 11 yellowish) and apical ⅔ elytra black, eyes dark. Head: vertex finely punctate, medial sulcus present; carina present between antennal bases; frons not projecting; not depressed between eyes. Antenna: reaches to humerus; robust; antennomere 1 incrassate, subclavate, elongate; 2–3 elongate, cylindrical, subequal in length, each about ¾ length 1; 4–10 transverse, subequal in length, each ¾ length of 3; 11 2× length 10, broadly rounded at apex; 1–2 punctate, with seta in each puncture; 3–11 setose. Pronotum: subquadrate; lateral margin straight then rounding to anterior angle, canaliculate; anterior angle obtusely rounded, slightly produced; posterior angle acute; anterior margin weakly emarginate behind head; disc subconvex; depressed laterally near base; surface with disc finely, sparsely punctate, punctures more dense and deeper laterally; pronotal length 1.5–1.9 mm; pronotal width 2.0–2.4 mm. Scutellum: broadly pentagonal; impunctate. Elytron: lateral margin straight, smooth, narrowly margined; apex rounded; sutural angle without tooth; humerus rounded, not produced; slightly constricted behind humerus; disc moderately convex; moderately punctate-striate, punctures becoming finer apically, rounds converge and unite apically; elytral length 5.2–5.9 mm; elytral width 2.5–2.9 mm. Venter: pro-, meso-, and metasterna impunctate medially, punctate laterally; abdominal sterna punctate, each puncture with pale seta; suture between sterna 1 and 2 obsolete medially; last sternite with apical margin broadly emarginate, slightly protruding medially in female, broadly emarginate and sinuate laterally in male. Leg: slender; sparsely punctate; tibia with fringe of setae on inner margin of apex. Total length: 7.0–7.9 mm.

##### Diagnosis.

This species is similar to *Cephaloleia basalis*. It can be distinguished by the elytral puncture rows converging and uniting apically and by antennomere 2 being subequal in length to 3.

##### Distribution.

Brazil (District Federal, Río de Janiero, Rondonia, Santa Catharina), Ecuador.

##### Type material examined.

Syntype: Brazil, Petropolis, [Baly, Fry, Clark, and Waterhouse colls.] (BMNH, 1).

##### Specimens examined.

**Brazil:** no further data (USNM). District Federal- Roceio dos Banirantes, 23 October 1945 (USNM). Río de Janiero- no further data (USNM). Rondónia- 62 km SW Ariquames, Fzda Rancho Grande, 9 November 1994 (BYUC). Santa Catharina- no further data (USNM). **Ecuador:** Napo- Sacha Lodge, 270 m, 23 April- 4 May 1994 (SEMC). Orellana- Estacion Cientifica Yasuni, 16 August 1997 (CDFA). Total: 10.

#### Cephaloleia weisei

Taxon classificationAnimaliaColeopteraChrysomelidae

Staines, 1996

http://species-id.net/wiki/Cephaloleia_weisei

Fig. 269

Cephaloleia weisei Staines 1996: 71.

##### Description.

Small; subovate; subdepressed; head, antennomeres 1–2, pronotum, and basal ½ of elytra yellowish; eyes, antennal antennomeres 3–11, and apical ½ of elytra black; venter and legs reddish-yellow. Head: vertex alutaceous, medial carina present; frons not projecting; depressed between eyes. Antenna: reaches to humerus; slender; antennomeres 1–2 elongate, cylindrical, subequal in length; 3–10 transverse, subequal in length; 11 pointed at apex, subequal in length to 1 or 2; 1–2 punctate with scattered setae; 3–11 setose. Pronotum: transverse; lateral margin straight for basal ¾ then rounding to anterior angle in male, female evenly arcuate, narrowly margined; anterior angle rounded, not produced; posterior angle acute; anterior margin straight; disc subconvex; punctures large, shallow, sparse; basal impression absent; pronotal length 0.7–0.9 mm; pronotal width 1.3–1.6 mm. Scutellum: pentagonal, alutaceous. Elytron: lateral margin slightly expanding to middle then rounding to apex, smooth, margined; apex rounded; sutural angle without tooth; humerus rounded, not produced; slightly constricted behind humerus; shallowly punctate-striate, rows obsolete on apical ⅓, row 10 removed from margin; elytral length 2.7–3.0 mm; elytral width 1.3–1.9 mm. Venter: pro-, meso-, and metasterna punctate; abdominal sterna punctate, each puncture with pale seta; suture between sterna 1 and 2 obsolete medially; last sternite with apical margin emarginate medially in male, truncate in female. Leg: slender; punctate, each puncture with pale seta; tibia with fringe of setae on inner margin of apex. Total length: 3.6–4.1 mm.

**Figures 269–273.**
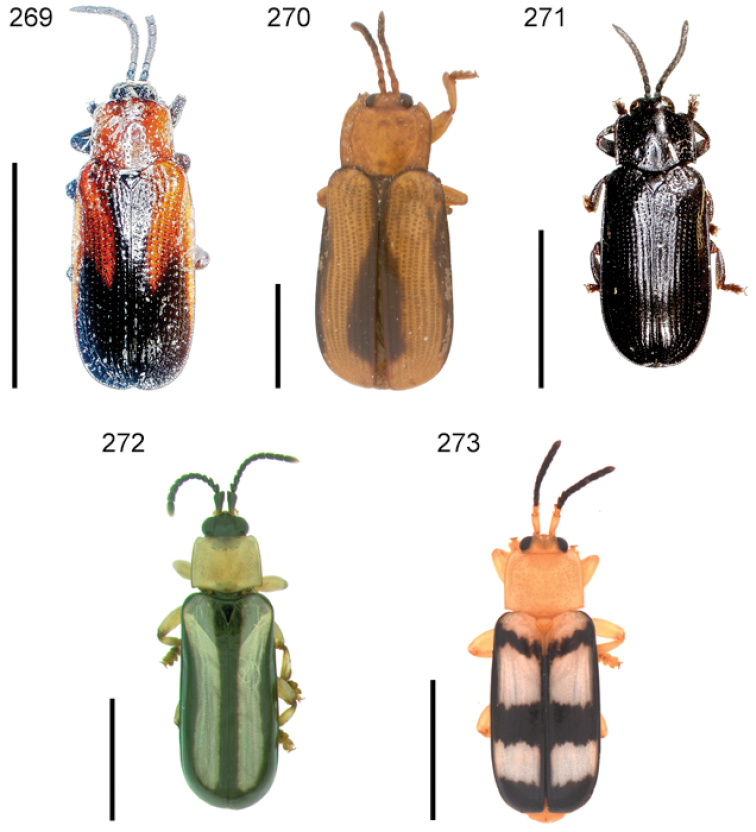
Habitus. **269** *Cephaloleia weisei* **270** *Cephaloleia whitei* **271** *Cephaloleia zikani* **272** *Cephaloleia kressi* sp. n. **273** *Cephaloleia stainesi* sp. n. Scale bars equal 3 mm.

##### Diagnosis.

This species is similar to *Cephaloleia trimaculata*. It can be distinguished by antennomeres 1 and 2 being subequal in length, by the uniformly punctate pronotum, and by the elytral puncture rows being obsolete apically.

##### Distribution.

Costa Rica, Panama.

##### Type material examined.

Holotype: Cocoli Panama C.Z., IX-2-1946/ N. L. H. Kraus/ Holotype Cephaloleia weisei Staines, Des. C. L. Staines 1994 [red label] (USNM).

##### Specimens examined.

**COSTA RICA:** Guanacaste- 3 km SE Naranjo, 24–26 May 1993 (USNM), 21–30 June 1992, 1–10 September 1992, 5 June 1993, 7 June 1993, 8–12 June 1993 (BYUC). **PANAMA:** Panamá- Barro Colorado Is., 13 August 1946 (USNM), 23–27 July 2000 (SEMC); Llano-Carti rd. at km 9, 18 May 1993 (EGRC). Total: 14.

#### Cephaloleia whitei

Taxon classificationAnimaliaColeopteraChrysomelidae

Baly, 1858

http://species-id.net/wiki/Cephaloleia_whitei

Fig. 270

Cephaloleia whitei Baly 1858: 41. Uhmann 1957b: 27 (catalog); Staines and Staines 1999: 524 (Baly species list).
Cephalolia whitei Baly. Gemminger and Harold 1876: 3602 (catalog); Donckier 1899: 551 (catalog); Weise 1910: 85 (noted), 1911a: 10 (catalog), 1911b: 10 (catalog); Uhmann 1936f: 482 (key).

##### Description.

Elongate; subparallel; subdepressed; shining; reddish-yellow; eyes and antennomeres 7–11 dark; elytra with suture and lateral vittae dark. Head: vertex impunctate, medial sulcus present; frons not projecting; depressed between eyes. Antenna: reaches to humerus; slender; antennomeres 1–3 compressed in male, 1 incrassate, 2–3 triangular, subequal in length; 1–3 not compressed in female, 1 incrassate, 2–3 elongate, cylindrical, 3 ¾ length of 2; 4–10 transverse, subequal in length, each ¾ length of 3; 11 2× length of 10, pointed at apex; 1–5 punctate with scattered setae; 6–11 setose. Pronotum: transverse; widest just before middle; lateral margin sinuate at base then rounding to anterior angle, canaliculate; anterior angle obtuse, slightly produced; posterior angle acute; anterior margin emarginate behind head; disc subconvex; surface sparsely, irregularly punctate; transverse basal impression present medially; pronotal length 2.1 mm; pronotal width 2.6 mm. Scutellum: pentagonal, apex acute; impunctate. Elytron: lateral margin straight, narrowly margined; apex rounded, emarginate at suture; sutural angle without tooth; humerus rounded, not produced; constricted behind humerus; flattened along suture; moderately punctate-striate, punctures oblong, rows converge and unite apically; elytral length 4.4 mm; elytral width 3.4 mm. Venter: last sternite with apical margin sinuate in male. Leg: slender; impunctate; tibia with fringe of setae on inner margin of apex. Total length: 9.0 mm.

##### Diagnosis.

This species is similar to *Cephaloleia felix*. It can be distinguished by the rounded, sinuate lateral margins of the pronotum and by the larger size.

##### Distribution.

Colombia.

##### Type material examined.

Holotype male: Colombia, Baly coll. (BMNH).

#### Cephaloleia zikani

Taxon classificationAnimaliaColeopteraChrysomelidae

Uhmann, 1935b

http://species-id.net/wiki/Cephaloleia_zikani

Fig. 271

Cephalolia zikani Uhmann 1935b: 48. Uhmann 1940: 114 (noted).
Cephaloleia zikani Uhmann. Uhmann 1938b: 365 (faunal list), 1957b: 27 (catalog); Gaedike and Döbler 1971: 363 (types); Staines 1997b: 414 (Uhmann species list).

##### Description.

Elongate; subparallel; subdepressed; shining; black. Head: vertex lightly punctate, with medial fovea; keel present between antennal bases; frons not projecting; slightly depressed between eyes. Antenna: as long as head and pronotum combined; slender; antennomeres 1–2 transverse, subequal in length, each ¾ length of 3; 3 elongate, cylindrical, longest; 4–10 transverse, subequal in length, each slightly longer than 1 or 2; 11 2× length of 10, rounded at apex; 1–2 punctate with scattered setae; 3–11 setose. Pronotum: transverse; lateral margin straight then rounding to anterior angle, slightly canaliculate; anterior angle rounded, slightly produced; posterior angle acute; anterior margin emarginate behind head; disc subconvex; surface sparsely punctate laterally and basally; transverse basal impression present medially; pronotal length 1.0–1.2 mm; pronotal width 1.4–1.6 mm. Scutellum: broadly pentagonal; impunctate. Elytron: lateral margin straight, smooth, narrowly margined; apex rounded, finely serrulate; sutural angle with small tooth; humerus rounded, not produced; slightly constricted behind humerus; strongly punctate-striate, rows confused near apex; elytral length 3.5–3.9 mm; elytral width 1.7–1.9 mm. Venter: pro-, meso-, and metasterna punctate; abdominal sterna punctate, each puncture with pale seta; suture between sterna 1 and 2 complete; last sternite with apical margin broadly truncate in male, emarginate medially in female. Leg: slender; punctate, each puncture with pale seta; tibia with fringe of setae on inner margin of apex. Total length: 4.9–5.3 mm.

##### Diagnosis.

This species is similar to *Cephaloleia depressa*, *Cephaloleia donckieri*, *Cephaloleia elaeidis*, and *Cephaloleia tarsata*. It can be distinguished by the medial fovea on the vertex of the head and by the smooth lateral margins of the pronotum and the smooth apical margins of the elytra.

##### Distribution.

Brazil (Rio de Janeiro, Santa Catharina).

##### Type material examined.

Holotype: Brazil, Est. do Río, Itatiaya, 1000 m, 5.XI.1931, Zikan (DEI).

##### Specimens examined.

**Brazil:** Santa Catharina- Nova Teutonia, 2 October 1936, 11 January 1937, 6 June 1940, 17 June 1940, 5 August 1949, January 1967, February 1977 (USNM), no date (AMNH), September 1976, October 1976, November 1976, December 1976, January 1977, February 1977 (EGRC). Total: 24.
